# CirPred, the first structure modeling and linker design system for circularly permuted proteins

**DOI:** 10.1186/s12859-021-04403-1

**Published:** 2021-10-12

**Authors:** Teng-Ruei Chen, Yen-Cheng Lin, Yu-Wei Huang, Chih-Chieh Chen, Wei-Cheng Lo

**Affiliations:** 1 Institute of Bioinformatics and Systems Biology, National Chiao Tung University, Hsinchu, Taiwan; 2 Institute of Bioinformatics and Systems Biology, National Yang Ming Chiao Tung University, Hsinchu, Taiwan; 3 Department of Biological Science and Technology, National Chiao Tung University, Hsinchu, Taiwan; 4 Department of Biological Science and Technology, National Yang Ming Chiao Tung University, Hsinchu, Taiwan; 5 Institute of Medical Science and Technology, National Sun Yat-sen University, Kaohsiung, Taiwan; 6 The Center for Bioinformatics Research, National Yang Ming Chiao Tung University, Hsinchu, Taiwan

**Keywords:** Circular permutation, Protein engineering, Protein structure modeling, Protein structure prediction

## Abstract

**Background:**

This work aims to help develop new protein engineering techniques based on a structural rearrangement phenomenon called circular permutation (CP), equivalent to connecting the native termini of a protein followed by creating new termini at another site. Although CP has been applied in many fields, its implementation is still costly because of inevitable trials and errors.

**Results:**

Here we present CirPred, a structure modeling and termini linker design method for circularly permuted proteins. Compared with state-of-the-art protein structure modeling methods, CirPred is the only one fully capable of both circularly-permuted modeling and traditional co-linear modeling. CirPred performs well when the permutant shares low sequence identity with the native protein and even when the permutant adopts a different conformation from the native protein because of three-dimensional (3D) domain swapping. Linker redesign experiments demonstrated that the linker design algorithm of CirPred achieved subangstrom accuracy.

**Conclusions:**

The CirPred system is capable of (1) predicting the structure of circular permutants, (2) designing termini linkers, (3) performing traditional co-linear protein structure modeling, and (4) identifying the CP-induced occurrence of 3D domain swapping. This method is supposed helpful for broadening the application of CP, and its web server is available at http://10.life.nctu.edu.tw/CirPred/ and http://lo.life.nctu.edu.tw/CirPred/.

**Supplementary Information:**

The online version contains supplementary material available at 10.1186/s12859-021-04403-1.

## Background

To facilitate the utilization of circular permutation (CP) as a protein engineering technique, we carried out this study. CP, a polypeptide backbone rearrangement, could be considered as if the native termini of a protein were linked and a new opening created elsewhere [1–3]. Studies on natural cases concluded that circular permutants (CPMs) usually retain their native structures and functions, sometimes with increased functional diversity or activity [1–3]. This property makes CP promising for bioengineering. By artificially creating CPMs, CP has been applied in various fields, such as studying the folding and function of proteins [4, 5], improving the stability, solubility, substrate affinity, substrate specificity, and activity of proteins [6–9]. It can be used to create biosensors, molecular switches, and novel bifunctional proteins [10–12]. Recently, it is also utilized to create split inteins [13, 14].

Despite being powerful, the implementation of CP poses challenges. First, CP is much more difficult, expensive, and time-consuming than traditional mutagenesis. Second, not every position is permissive for CP [7, 15]. Third, when the termini of a protein are distant, a peptide linker should be designed to connect them, or the CPMs are unlikely viable [9]. Forth, conventional modeling algorithms are inadequate for predicting the structure of circularly permuted proteins. We have previously developed a viable CP cutting site predictor [16, 17]. Nevertheless, there is still a lack of a 3D structure predictor and linker design algorithm for CP. Because of the rearrangement nature, when modeling a CPM, even state-of-the-art comparative modeling systems like the SWISS-MODEL [18], RaptorX [19], Robetta [20], and our work (PS)^2^ [21] usually generate a partially modeled structure. So far, uneconomic trials and errors are inevitable for CP bioengineering.

**Fig. 1:**
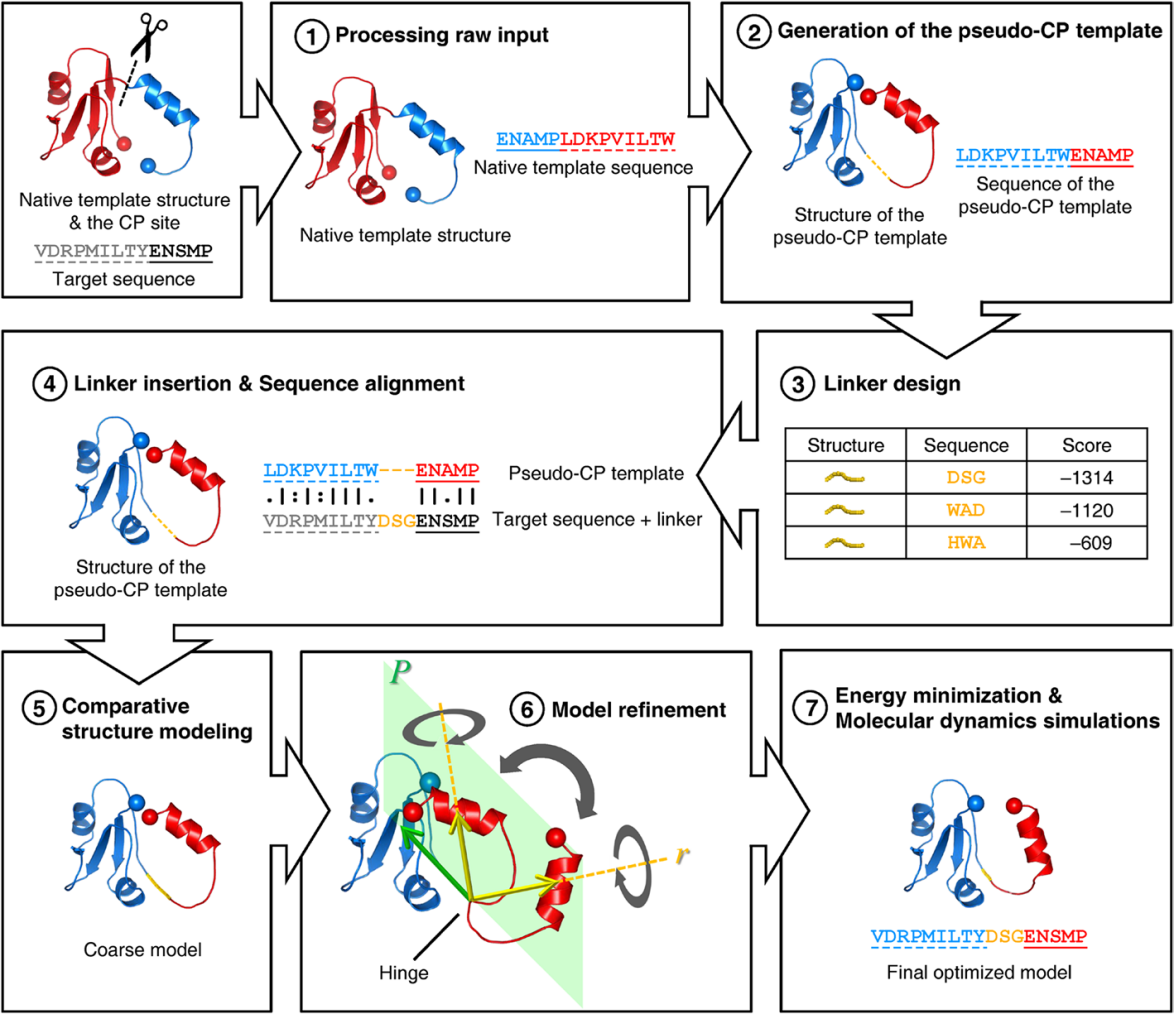
Flowchart of the CirPred method. After obtaining the input data inclusive of a native template structure, a CP site, and a target sequence, seven major steps are performed. (1) The native template is processed to restore missing atoms and obtain the native amino acid sequence. (2) A pseudo-CP template structure and sequence are created according to the CP site. (3) A linker design procedure is carried out when necessary. (4) Sequence alignment between the pseudo-CP template and the target sequence is performed with native termini connected by the designed linker. (5) A coarse model is produced by comparative structure modeling. (6) The coarse model is refined by an algorithm that uses the CP site as a hinge to find the optimal orientation of domains (see “Methods” section). Each arrow represents a vector from the hinge to the center of mass of a domain. (7) Energy minimization and MD simulations are carried out to make the optimized model. In the web implementation, the linker design algorithm is activated only when the user does not provide the target sequence. In this situation, the target sequence is directly obtained from the template structure according to the CP site. The blue and red colors represent the N- and C-terminal proportions of a protein, respectively. The solid and dotted underlines indicate sequence fragments corresponding to the N- and C-terminal proportions of the native protein

In this work, we have developed the first CP structure modeling and linker design method named CirPred (Circularly-permuted protein structure Predictor), which integrates several algorithms of protein structural computation, machine learning, and molecular dynamics (MD) simulations in a “circularly-permuted” fashion (see Fig. 1 and “Methods” section). As tested with experimentally-verified CPMs of the dihydrofolate reductase (DHFR) [15], CirPred was the only comparative modeling method capable of producing complete models. Evaluated with ~ 1600 pairs of CPMs from literature and the Circular Permutation DataBase (CPDB) [22], the average alignment ratio and root-mean-square distance (RMSD) between CirPred-modeled and actual structures were better than 90% and 2.5 Å, respectively, even for CPMs sharing ~ 20% sequence identities. On average, linkers designed by CirPred possessed 70.2% sequence similarities with native linkers. For proteins with amino (N)- and carboxyl (C)-termini closer than 10 Å, the linkers designed by CirPred achieved an accuracy of 0.26 Å. Interestingly, we found CirPred capable of detecting protein 3D domain swapping (DS) [23]. When CP and DS co-occurred, CirPred provided accurate predictions of the structure and orientation of domains.

To fulfill the aims of this work, we implemented the CirPred into a rapid web server. With the assistance of this effective structure predictor, we hope that the time and cost of CP implementation can be significantly decreased, letting this powerful technique open protein and enzyme engineering to new possibilities.

## Results

### Comparison of structural models generated by CirPred and conventional modeling methods

Since proteins related by CP have different start points of the polypeptide sequence, conventional structure modeling methods typically meet difficulties in finding suitable templates when processing a circularly permuted target protein. Even if the native protein of the target is provided as the template, they may still fail to build a full-length model. It is commonly observed that, delimited by the CP site, a part of the model is missing or predicted as extended loops/coils. In other cases, the model is predicted to possess two well-folded domains, but the predicted orientation of domains is incorrect.

**Fig. 2:**
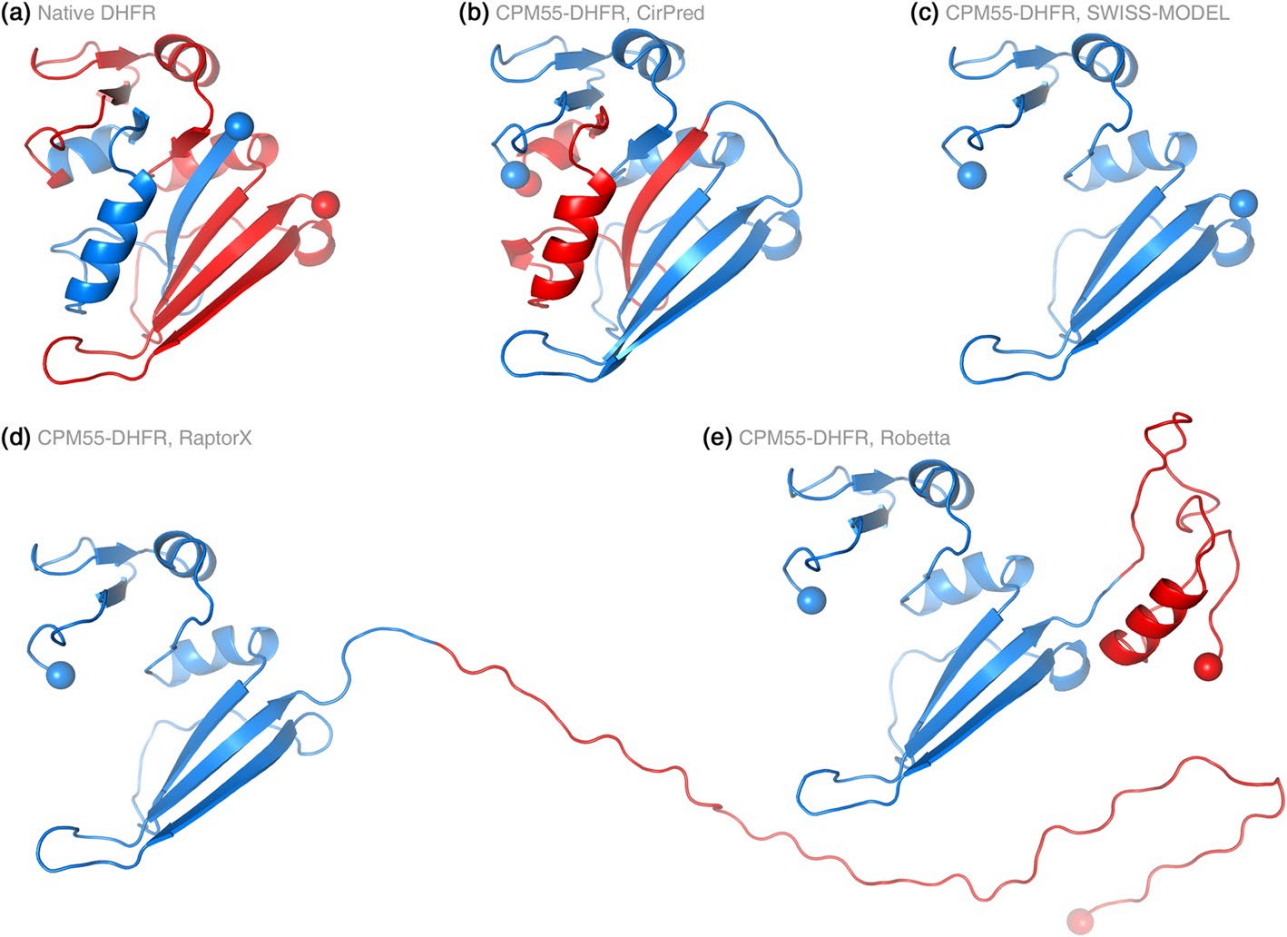
Circularly-permuted protein structure models constructed by CirPred and conventional modeling methods. The CPM55 of DHFR has been reported viable (CP site on the native DHFR: Pro55) [15]. Several comparative structure modeling methods were applied to construct the model of this CPM. Conventional methods failed to model part of the CPM (panel **c** and **d**) or produced incorrect domain orientations (panel **e**). The proposed CirPred method correctly constructed the full-length model (panel **b**). Structures shown in this figure include **a** the native DHFR (PDB 1rx4A), and the model of CPM55 constructed by **b** CirPred **c** SWISS-MODEL [18], **d** RaptorX [19], and **e** Robetta [20]. Structures are drawn in the same orientation and scale. The blue and red colors indicate the N- and C-terminal proportions, respectively, of the protein. The α-carbon atoms of termini residues are shown as spheres

The entire polypeptide of the DHFR had been scanned by CP to identify viable CP sites [15]. According to those CP sites, we generated all the permutant sequences and submitted them to state-of-the-art comparative modeling systems, including SWISS-MODEL [18], RaptorX [19], and Robetta [20] (see Additional file 1). When CP occurred at positions close to the center of the polypeptide, the quality of models built by these methods was generally good. As the CP site moved close to the N- or C-terminus, the missing, coiled, or incorrect-orientation modeling problems became increasingly serious. If a modeled permutant and the native DHFR were structurally aligned, the worst results (the lowest alignment ratio or highest RMSD) usually occurred when the CP site is situated around 1/4 or 3/4 of the sequence. The same permutant sequences were processed with the proposed CirPred method, and all the produced CPM models aligned well with the native protein. Take CP site residue 55 for example, the CPM model built by CirPred retained the correct conformation of DHFR (compare Fig. 2a, b). As for the model constructed by SWISS-MODEL, the proportion corresponding to residues 1–55 of the native DHFR was missing (Fig. 2c). The same proportion was predicted as a long coil by RaptorX (Fig. 2d). Robetta successfully modeled the two proportions delimited by the CP site, but the predicted orientation was wrong (Fig. 2e).

CirPred was the only one capable of correctly modeling all viable DHFR CPMs among the assessed methods, demonstrating its specificity to circularly permuted proteins. Additionally, it is noteworthy that CirPred is applicable to conventional co-linear modeling. By setting the CP site to be residue 1 of a protein, CirPred would create a co-linear model with comparable quality to models constructed by state-of-the-art modeling systems. See Additional file 2 for results of co-linear protein structure modeling performed using CirPred.

### Comparison of models generated by CirPred and conventional modeling methods for proteins requiring termini linkers

When the native termini of a protein for CP were close, short poly-glycine or glycine/serine-rich linkers were frequently used [9]. However, longer linkers may be required for proteins with distant termini to be successfully engineered by CP [9]. We proposed a linker design protocol (see “Methods” section), which further differentiated the CirPred from traditional modeling systems. Here a linker redesign experiment was conducted to demonstrate how the proposed protocol could design a long peptide linker for proteins having distant termini.

**Fig. 3:**
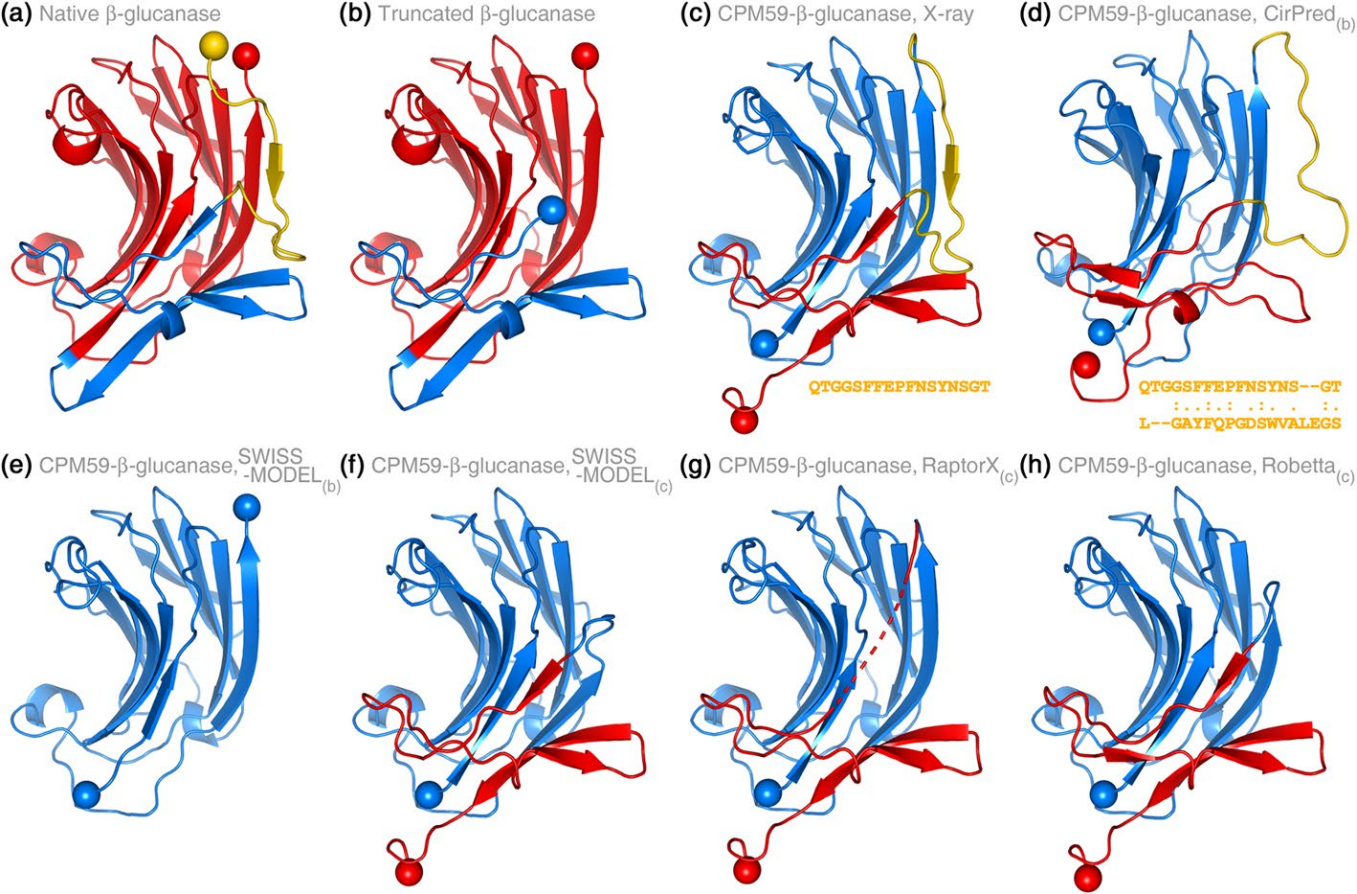
Circularly-permuted models constructed by several modeling methods for proteins requiring a long termini linker. The CPM structure of a β-glucanase has been determined [24]. In this experiment, the 17 N-terminal residues of the native β-glucanase were removed to test the linker design capability of CirPred. The model constructed by CirPred was complete, and the designed linker was similar to the removed 17-residue fragment both in structure and sequence. Conventional modeling methods either twisted the structure to connect the native termini or failed to fill the gap in between. **a** Crystal structure of the native 1,3–1,4-β-glucanase (PDB 2ayhA). The yellow fragment corresponds to the 17 N-terminal residues for truncation. **b** The N-terminus truncated β-glucanase (denoted β-glucanase_∆1–17_). **c** Crystal structure of the CPM59 of the β-glucanase (PDB 1cpmA). The yellow fragment (and sequence) is equivalent to the truncated part of the native protein and was the target linker to be redesigned. **d** Model constructed by CirPred using **b** as the template. The yellow fragment is the redesigned linker, whose sequence similarity to the native linker is 70.6% (aligned by Stretcher [32]). **e** Model constructed by SWISS-MODEL using **b** as the template. The C-terminal proportion of the structure was missing. **f** Model constructed by SWISS-MODEL using **c** as the template. The longest main-frame β-strand was twisted to connect the native termini. **g** Model constructed by RaptorX using **c** as the template. As the dotted line indicates, an unreasonable gap remained between the native termini. **h** Model constructed by Robetta using **c** as the template. The direct connection between the native termini twisted the longest main-frame β-strand

The 1,3–1,4-β-glucanase from Bacillus with Protein Data Bank (PDB) entry 2ayhA (Fig. 3a) had been engineered by CP using Phe59 as the CP site, and the permuted structure had been determined (denoted as CPM59-β-glucanase; Fig. 3c) [24]. We deleted residues 1–17 from the native β-glucanase structure 2ayhA to create a new N-terminus 21.97 Å away from the C-terminus. The truncated β-glucanase (denoted as β-glucanase_∆1–17_; Fig. 3b) simulated a protein that possessed distant termini, and a long linker (17 residues) should be added for CP bioengineering. With β-glucanase_∆1–17_ as the template, we constructed the model of CPM59-β-glucanase using several methods. If a method could properly design a linker for β-glucanase_∆1–17_, the designed linker should likely be similar to the fragment in the actual structure of CPM59-β-glucanase (the yellow part in Fig. 3c) that is corresponding to the deleted residues in the native β-glucanase (the yellow part in Fig. 3a). The result showed that CirPred successfully designed a linker similar to the deleted residues in structure and sequence (see Fig. 3d). The last 58 residues of CPM59-β-glucanase were missing in the model constructed by SWISS-MODEL (Fig. 3e). As for RaptorX and Robetta, the long-coil and incorrect-orientation predictions exhibited in Fig. 2 persisted. We had then utilized the actual structure of CPM59-β-glucanase as the template to construct the model of CPM59 itself using SWISS-MODEL, RaptorX, and Robetta. These algorithms either directly connected the native termini of β-glucanase by a twist (Fig. 3f, h) or simply formed a big gap between the termini (Fig. 3g). It should be noted that only CirPred was equipped with a linker design protocol; therefore, the above comparisons were not made to compete with conventional modeling methods for performance but only to indicate the novelty of CirPred. See “Performance of linker design” section for large-scale evaluations of the proposed linker design protocol.

### Performance on engineered CPs

Artificially engineered circular permutations are suitable materials for assessing the performance of a CP structure predictor because their parent proteins, CP sites, and additional mutations were well defined. The engineered CPMs collected in [25] were used to assess CirPred. The sequence of each engineered CPM was the target, which was subjected to CirPred modeling with its parent protein used as the template. Since the structure of all applied CPMs had been known, the performance of CirPred could be evaluated by aligning the known structures with constructed models. As shown in Table 1, the structural alignment qualities were high. The average alignment ratio and RMSD were 99.2% and 1.59 Å, respectively.

**Table 1:** Structural alignment qualities between models constructed by CirPred and the actual structures of engineered CPMs

Target: engineered CPM (size: residues)	Template: native protein (size: residues)	CP site on the template^a^	Identity^b^ (%)	Alignment ratio^c^ (%)	RMSD^c^ (Å)
1ajkA (212)	2ayhA (214)	84	90.2	97.6	1.441
1ajoA (212)	2ayhA (214)	129	97.2	100.0	1.026
1cpmA (214)	2ayhA (214)	59	98.6	100.0	1.001
1cpnA (208)	2ayhA (214)	59	92.5	100.0	1.050
1alqA (259)	3blmA (257)	223	98.8	100.0	0.548
1fw8A (416)	3pgkA (416)	72	72.8	98.1	3.155
1n02A (102)	2ezmA (101)	50	90.1	100.0	1.316
1un2A (186)	1a2jA (188)	100	96.3	100.0	1.156
1bd7A (176)	1blbC (187)	98	85.6	94.9	6.501
1g2bA (62)	1shgA (57)	47	86.0	100.0	1.056
1tucA (61)	1shgA (57)	20	86.0	96.7	1.609
1tudA (60)	1shgA (57)	48	89.5	100.0	0.833
1swfA (116)	1stpA (121)	51	87.9	100.0	1.256
1swgA (112)	1stpA (121)	51	86.6	100.0	0.657
1p5cA (166)	1lw9A (164)	12	98.1	100.0	1.310

^a^Numbered according to the order of residues in the PDB file

^b^Sequence identity between the ***template*** protein and the target CPM (computed by the circularly-permuted structure alignment algorithm CPSARST [25])

^c^These were the structural alignment ratio and RMSD values between the ***model*** and the actual structure of target CPMs. The alignment ratio was defined as the number of aligned residues divided by the target’s size

### Large-scale assessments for various sequence identity levels

To examine how the modeling quality of CirPred would be influenced by the sequence identity between the target CPM and the template, we conducted an extensive test using CPDB, the largest dataset of manually-verified pairs of CPMs [22]. The 1568 CP pairs of CPDB with sequence identities ≥ 10% (Additional file 3) were grouped into subsets of decreasing identities. For each pair of CPMs, one was used as the template for modeling the other. The results are listed in Table 2. The quality of models remained high until the identity was lower than 20%. For those CP pairs with identities ≥ 20%, the average alignment ratios between models and actual structures were all > 90%, and the average RMSDs were generally < 2.50 Å. Even for those with identities < 20%, the average alignment ratio and RMSD still reached 74.0% and 3.90 Å, respectively. For instance (see Additional file 4), a RIM2 C_2_A-domain (PDB 2bwqA) and a calcium-phospholipid binding domain (PDB 1rlwA) shared only 19.5% identity. The model made based on 2bwqA as the template (CP site: residue 16) achieved a structural alignment ratio of 95.2% and RMSD 2.75 Å when aligned with the known structure of the target CPM, i.e., PDB 1rlwA. In summary, the performance of CirPred would decrease as the sequence identity between template and target lowers, but the quality of the constructed model remains high as long as the identity is not far below 20%.

**Table 2:** Effects of sequence identity on the performance of CirPred

Sequence identity^a^ (%)	Alignment ratio^b^ (%)	RMSD^b^ (Å)	Number of CP pairs
90–100	97.6	1.236	79
80–90	95.7	1.673	39
70–80	94.7	1.504	8
60–70	95.1	3.124	10
50–60	96.7	1.910	31
40–50	98.7	2.002	236
30–40	98.1	2.026	351
20–30	90.9	2.496	161
10–20	74.0	3.904	653

In this experiment, for each pair of circular permutants, one protein was utilized as the template to create the model of the other protein, i.e., the target

^a^Sequence identities between the ***template*** and target proteins

^b^Alignment ratio and RMSD values between the ***model*** and known structure of target proteins

In addition to sequence identity, we had examined to what extent the performance of CirPred would be influenced by protein size or the location of CP sites. Additional file 5: Tables S1 and S2 demonstrate that protein size exerted little influence on the modeling quality; however, the performance decreased slightly for proteins with CP sites close to the termini (see also Additional file 4 for examples of CirPred modeling for CPMs of different sizes).

### Performance of linker design

Connecting the native termini of a protein is a formidable challenge for successful CP engineering [9]. A direct connection may be appropriate when the native termini are close in 3D space; otherwise, a well-designed peptide linker is required. There is still no general method for designing CP linkers in addition to short glycine/serine-rich peptides [9]. In “Methods” section, a linker design protocol for CP is proposed. This subsection provides performance assessments of the proposed protocol.

#### Evaluation with known circular permutants

A CPDB linker dataset (Additional file 6) containing all non-redundant linkers of CPMs from the CP pair dataset was prepared to evaluate the linker design algorithm of CirPred by performing a strict 500-round independent test described in “Methods” section. Statistical analyses showed that 99.0% of the CPM structures with linkers designed by our algorithm exhibited equivalent or lower potential energy than their original structures. The average sequence similarity between the designed and the original linkers was 68.6% (see Additional file 7 for raw data and Additional file 8: Table S3 for statistics). Since all the original linkers were obtained from naturally occurring CPMs, these data indicated that the proposed algorithm could design linkers with structural stabilities and sequence compositions analogous to linkers that evolved naturally.

To dissect the influence of the distance between native termini on the performance of linker design, we further analyzed the sequence similarities and RMSDs between the designed and original linkers. As the distance between termini increased, the similarity lowered, and RMSD rose, indicating a decline in performance (see Additional file 8: Fig. S3 and Table S3). However, since the output of the CirPred linker design procedure for a given protein was a set of candidate linkers ordered by potential energy, retrieving more candidates helped find better results. Take proteins with a termini distance ≤ 10 Å for instance, if the top 5 candidates were retrieved, the average optimal sequence similarity between the designed and original linkers was 83.7%, and the RMSD was 0.32 Å; if the top 10 candidates were retrieved, those values became 90.7% and 0.26 Å, respectively. Before this work, the most accurate linker design method accomplished a 0.50 Å RMSD out of 6 candidates [26]. The CirPred has made a noticeable advance in the linker design for CP engineering.

#### Evaluation with in silico circular permutants

To perform a thorough assessment of the linker design algorithm, we prepared an in silico synthetic CPM dataset, namely, the Dataset S (Additional file 9), in which every protein shared < 25% identity with any protein either from itself or from the CPDB linker dataset. There were 2141 CPMs in Dataset S, each with a known native protein and a predefined missing linker. After the machine-learning predictor of CirPred’s linker design module was trained with the CPDB linker dataset, every CPM in Dataset S was processed by CirPred modeling to redesign its missing linker. The potential energy of CirPred models was compared with that of the actual CPM structures. The results showed that 98.4% of the CPMs with linkers redesigned by CirPred had equivalent or lower potential energy than their original structures, and the average sequence similarity between the redesigned and original linkers was 71.7%. Since Dataset S was highly non-redundant and very different from the CPDB linker dataset (i.e., the training data for machine learning), this independent test demonstrated that the proposed linker design protocol was stable in performance.

### Ability to model 3D domain-swapped proteins induced by CP

As we evaluated the CirPred with engineered CP cases, a very interesting situation was recognized for the CPMs of βB2-crystallin with PDB entries 1bd7A and 1blbC. The crystallin 1bd7A was an artificial CPM of 1blbC with residue Glu87 as the CP site [27]. Crystallin 1blbC, before the CP, was a homo-tetramer composed of two dimers, each of which was still a dimer comprising two intertwined subunits with an open conformation. The CP converted the **inter**molecular pairing between subunits into **intra**molecular pairing and thus disentangled the intertwined subunits into two side-by-side monomers with a closed conformation [27]. The conversion between intertwined dimeric “open-form” and disentangled “closed-form” is an example of 3D domain swapping (DS) [23].

**Fig. 4:**
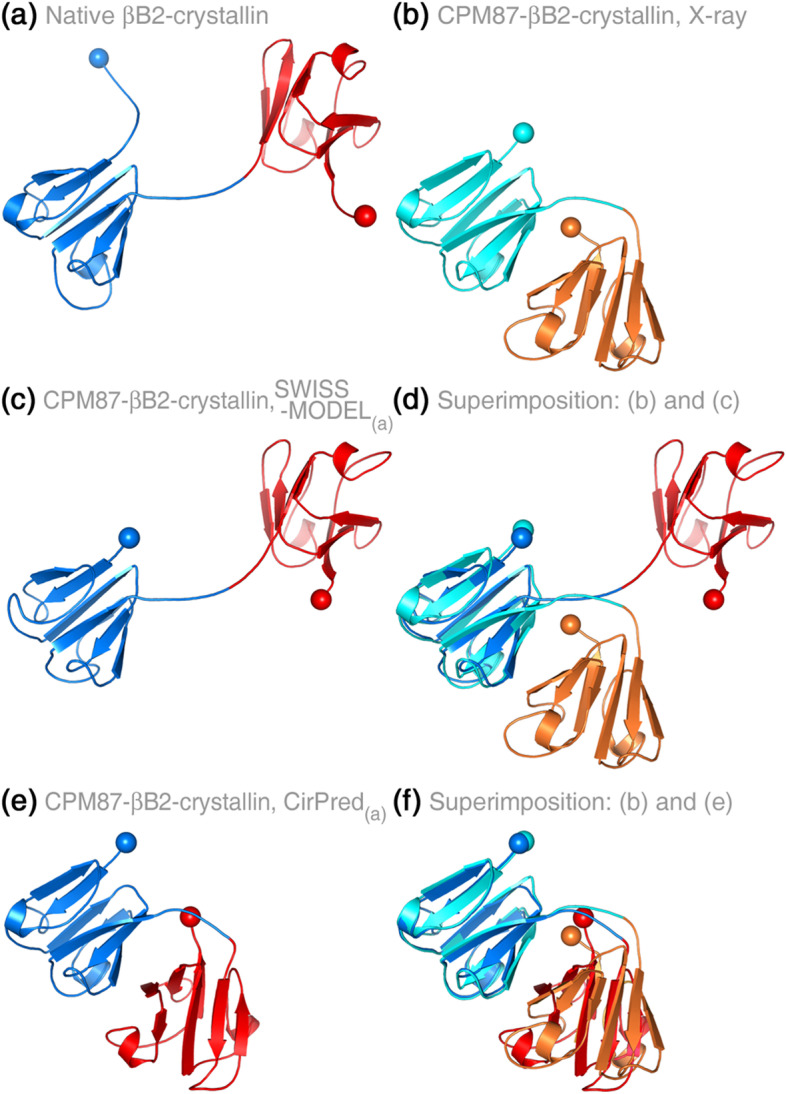
Modeling of proteins with 3D domain swapping phenomenon induced by CP. The structures of **a** the native βB2-crystallin (PDB 1blbC) and **b** the engineered CPM87 βB2-crystallin (PDB 1bd7A; CP site on the native crystallin: Glu87) have both been solved by X-ray crystallography. This pair of circular permutants is also an example of the 3D domain-swapping phenomenon. Since this crystallin had a two-fold rotational symmetric structure and the CP site was at the middle of the subunit, conventional comparative modeling methods might be able to construct the full model of the CPM by mimicking the template structure, such as **c** the model of CPM87 βB2-crystallin constructed by SWISS-MODEL using **a** as the template. However, the conformation of the models constructed by conventional methods was very different from that of the actual CPM structure, as shown by **d** the superimposition between **b** and **c**. Interestingly, **e** the model of CPM87 βB2-crystallin constructed by CirPred was highly similar to the actual CPM conformation, as shown by **f** the superimposition between **b** and **e**

DS-related homologs are difficult for sequence or structure alignment methods to identify because of the dramatic conformational difference [28]. The identification of CP is also not straightforward [25]. When CP and DS co-occurred, the situation would become too complicated for conventional modeling systems to construct a model of correct conformation, even if a proper native template was provided. One native βB2-crystallin subunit (182 residues; PDB 1blbC) contains two homologous tandem-repeat domains. Since the CP site 87 (which created crystallin 1bd7A, or the CPM87-crystallin) was situated close to the middle of the subunit, it would be expectable that conventional modeling systems construct a model of CPM87-crystallin that is similar to the native crystallin. Indeed, as Fig. 4 demonstrated, when the CPM87-crystallin sequence was input as the target and the native crystallin (Fig. 4a) as the template, SWISS-MODEL [18] built a model very similar to the native structure (Fig. 4c). However, the truth was complicated. The actual structure of the CPM87-crystallin (PDB 1bd7A; Fig. 4b) had a very different domain orientation from the native crystallin. Thus, superimposition between the model built by SWISS-MODEL and the actual CPM87-crystallin structure showed a low alignment ratio (51.7%) and a large spatial displacement in the unaligned region (Fig. 4d). Contrarily, the CirPred model of CPM87-crystallin showed a very high alignment ratio (99.4%) and a small RMSD (3.61 Å) with the actual CPM structure (Fig. 4e, f). The fact that CirPred could correctly model a 3D domain-swapped CPM implied that it has the potential of detecting the DS phenomenon induced by CP.

### Web server of CirPred

The CirPred algorithm has been implemented as a web server providing three working modes, (1) structure modeling for a primary CP, (2) structure modeling for a highly modified CPM, and (3) linker design. On the input page, the user will be asked to provide a structure of the native protein and a CP site. If the linker had been designed, using Mode 1 is appropriate. If there are other sequence modifications (substitutions, insertions, and deletions) on the native protein in addition to the CP and the linker, Mode 2 can be a great help. If a linker is necessary and the user needs assistance in designing it, Mode 3 is applicable. CirPred will provide 30 candidate linkers with their CPM models ordered by the DOPE score. The final choice should depend on the user’s knowledge of the protein to be engineered. Distributed computation techniques [29] are applied to speed up the execution of CirPred algorithms. If the optional MD simulation step is not enabled, a typical query of any mode requires < 3 min.

## Discussion

As a novel comparative modeling method, CirPred is fully capable of modeling circularly permuted proteins, even if the permutant shared a low sequence identity with the native protein. When a polypeptide linker is required to connect the termini before CP, CirPred helps design the linker. Besides, it could be applied to the design of 3D domain-swapped proteins by CP.

The novelty of CirPred was established on three bases. First, a pseudo-CP template was made before modeling. Second, CPDB [22] provided valuable data for establishing the machine learning predictor of the linker design protocol. Third, the CP-site-hinged model refinement procedure (Fig. 1 and “Methods” section) helped overcome the problems met by comparative modeling systems when dealing with circularly permuted proteins (e.g., incorrect domain orientation and expensive time cost). It also enabled CirPred to model domain-swapped CPMs.

### On the performance for low identity circular permutants

The quality of sequence alignment between the target and template is crucial to the accuracy of comparative modeling, especially when their sequence identity is low. It has been reported that for cases with target-template identity < 40%, an error of ~ 4 Å would be introduced into the model by just a single-residue misalignment [30]. Using CirPred, even when the identity fell to 20–40%, the RMSDs between the constructed models and the known structures of target permutants were < 2.5 Å, and the proportions of structurally well-aligned residues between them were > 90% (Table 2). CirPred performed well at low identity because it utilized three global sequence alignment methods [21, 31, 32] to find the optimal target-template alignment according to the produced sequence similarity. Among those methods, (PS)^2^ [21] took the predicted secondary structure of the target and the known secondary structure of the template into consideration and was particularly suitable for making accurate target-template alignments. Notwithstanding its high alignment quality, the current implementation of (PS)^2^ is dependent on a secondary structure predictor developed two decades ago and a traditional gap penalty scheme. If new secondary structure prediction strategies like [33–35] could be applied, or gaps could be dynamically suppressed within regions of regular secondary structure, buried core, or straight segments, the sequence alignment quality would be significantly improved and make CirPred more accurate for CPMs sharing < 20% sequence identities.

### On the accuracy of linker design

We proposed a linker design algorithm to provide bioengineers with energetically-favored choices in addition to glycine-rich linkers. The performance of this algorithm should be owed to its “predict-and-refine” strategy. Even if the accuracy of the initial machine learning prediction of amino acid for each residue position of a linker was only 67.5%, the subsequent probability-guided random modeling and energy screening greatly increased the chance of finding suitable linker sequences. Nevertheless, if the absolute prediction accuracy could be improved, the quality of designed linkers might be enhanced. At least, the algorithm’s efficiency could be enhanced by reducing the number of random modeling (see Additional file 10 for advanced discussions).

### On 3D domain-swapped circular permutants

DS is a quaternary structural phenomenon defined as proteins exchanging equivalent parts to form oligomers [23]. Understanding DS may help find new treatments for protein deposition diseases [36, 37] and develop bioengineering technology to modify enzyme activities or create auto-assembling biopolymers [38, 39]. Since DS enables homologous proteins to have dramatically different conformations (open *versus* closed) and CP changes the location of N-/C-termini, if they occurred concurrently, obtaining a reliable model by conventional modeling methods would be improbable. The CP-site-hinge model refinement step of CirPred seemed to create a shortcut to overcome the difficulty in modeling DS-related proteins caused by CP. The combination of naturally occurring CP and DS has been reported [9, 40, 41], and the βB2-crystallin we met in this study was an engineered case. These interesting examples implied that it is possible to create “circularly-permuted domain swapping” proteins to accomplish novel functions like molecular switches and auto-assembling biomaterials. CirPred is supposed useful for this combined type of protein engineering (Additional file 10).

### Other applications

Previously, we developed a protein viable CP site predictor abbreviated “CPred” [16], the machine learning model of which was constructed when CP (in)viability data were rare. In addition to reconstructing the model with the rapidly increasing data of recent years, integrating the proposed CirPred algorithm into the CPred pipeline may help improve the accuracy of CP site prediction. A preliminary test based on the CP viability data of [42] demonstrated that the energy scores computed according to CirPred models helped calibrate CPred predictions. Details of this preliminary test and discussions about future applications of CirPred in template identification, CP study, and complex structure modeling are also available in Additional file 10.

## Conclusions

We proposed a comparative modeling method for circularly permuted proteins to facilitate and broaden the application of CP in protein engineering. This CirPred method could accurately construct the model of a circular permutant at low sequence identity, indicating that it is promising for predicting the structure of a protein engineered by CP even if many other mutations are introduced. CirPred can design polypeptide linkers similar to the linkers of naturally occurring circular permutants. This ability helps ensure the viability of an engineered permutant that requires additional residues to connect the native termini. The model refinement procedure not only accelerated the search for energetically stable conformation for the domains delimited by the CP site but enabled CirPred to identify 3D domain swapping induced by CP. Thus, CirPred may assist in engineering “CP + DS” proteins with different biological properties, fusion sites, and oligomeric states from native proteins. Regular mutagenesis has enabled researchers to manipulate enzymes, antibodies, signal transmitters, cellular structural proteins, etc., for various applications. Circular permutation has brought about many novel engineered proteins that were difficult to create by regular mutations. We believe that many more protein engineering possibilities will be achieved by combining circular permutation with regular mutagenesis. As the first computational method capable of circularly-permuted structure modeling with linker design and co-linear structure modeling, CirPred shall help move forward various fields requiring protein engineering. The CirPred web server is available at http://10.life.nctu.edu.tw/CirPred/ (main server) and http://lo.life.nctu.edu.tw/CirPred/.

## Methods

### Software

In addition to the algorithms and software developed in our previous works, such as the CPSARST [25], (PS)^2^ [21], and an integrated machine learning and optimization server [16, 17], several third party software packages were applied in this study, inclusive of the sequence alignment program Stretcher (vEMBOSS:6.6.0.0) [32], the comparative modeling software Modeller (v9.19) [43] and the molecular dynamics simulation package GROMACS (v2016.4) [44]. Many steps of the proposed CirPred method were computationally expensive. The distributed computation technique we developed for the *i*SARST protein structural similarity search server [29] was extensively used to speed up research progress and the CirPred web server. All protein structures shown in this report were rendered using PyMOL [45].

### Datasets

#### The dataset of CP pairs from CPDB

Pairs of circular permutants sharing sequence identities ≥ 10% were downloaded from the CPDB [22]. Since the alignment data of CPMs provided by CPDB were computed by an old version of CPSARST [25], the structural alignment measures (e.g., alignment ratio and RMSD) and CP sites of these CP pairs were updated by the current CPSARST implemented in the *i*SARST server [29]. This dataset of 1568 CP pairs (Additional file 3) was used to perform large-scale evaluations of CirPred.

#### The CPDB linker dataset

**Fig. 5:**
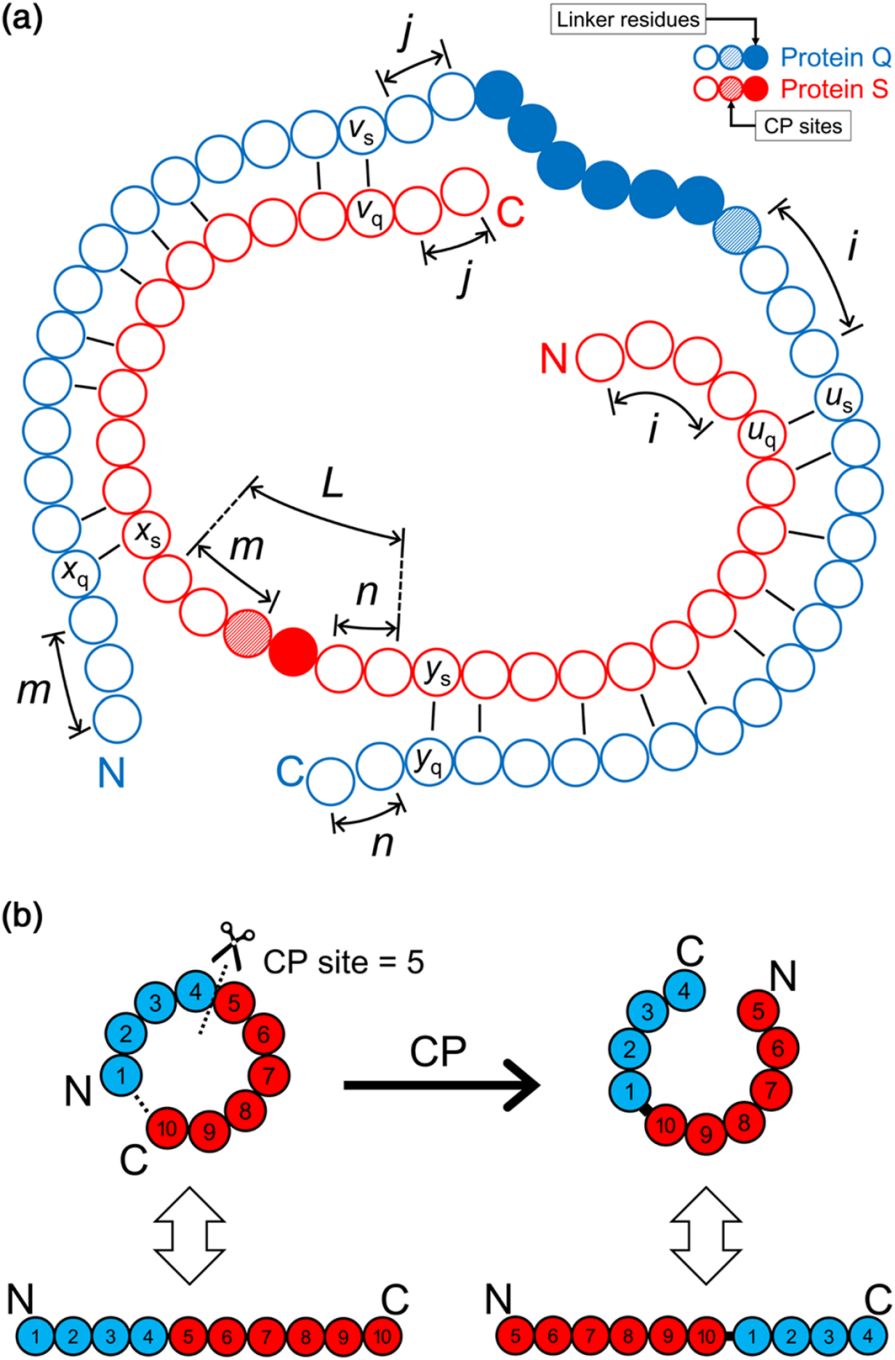
Determination of termini linkers and, in silico circular permutation. **a** Algorithm for determining the termini linkers for a pair of CPMs. In the implementation, the circularly-permuted structure alignment between CPMs was performed by CPSARST [25]. **b** The in silico circular permutation. The circular permutation is, in essence, a sequence interchange between the N- and C-terminal proportions

CP-based structural alignments of CP pairs from CPDB were performed by CPSARST to establish this dataset. For each CP pair, as illustrated in Fig. 5, two linkers were obtained based on the algorithm stated below,

1. Let Q and S represent the two proteins of a CP pair.
2. Let *x*_q_ represents the first residue of Q aligned with S, and *x*_s_ represents the residue of S aligned with *x*_q_. Similarly, let *y*_q_ be the last aligned residue of Q and its equivalent residue on S be *y*_s_.
3. The candidate linker for Q was computed as the residues between *y*_s_ and *x*_s_ on S (excluding *y*_s_ and *x*_s_). Let *l* be the number of residues of this candidate linker.
4. Let *m* be the number of unaligned residues on the N-terminus of Q in front of *x*_q_ and *n* be the number of unaligned residues on the C-terminus of Q after *y*_q_.
  (i) If *m* + *n* ≥ *l*, no linker was required to connect the native termini of Q.
  (ii) Otherwise, the linker for Q would be refined as the residues between *y*_s_ + *n* and *x*_s_ − *m* on S (excluding *y*_s_ + *n* and *x*_s_ − *m*).
5. Referring to Fig. 5a, let *x*, *y*, *m*, and *n* be replaced with *u*, *v*, *i*, and *j*, respectively. Repeat steps 2–4 to find out the linker for protein S.

The CPDB termini linker dataset is available in Additional file 6. If a protein has more than one permutant, it may have multiple linkers in this dataset.

#### Dataset S, a synthetic termini linker dataset

This dataset consisted of in silico circularly permuted proteins, each with a surface polypeptide fragment removed to serve as the known missing linker. The following steps were taken to establish this dataset.

1. Let the position of a residue be represented by its α-carbon (Cα).
2. Using the 100% sequence identity non-redundant subset of the PDB (snapshot date: Dec. 25th, 2016), calculate the average distance, denoted by *d*, between two consecutive residues in a protein. The result was *d* = 3.36 (Å).
3. Compute the distance between the N- and C-terminal residues, denoted by *D*, for each protein in the above PDB subset (73,073 proteins) and filter out any protein with *D* > 3*d*. Only 3027 proteins remained in the dataset, in which every protein possessed a short distance between termini, i.e., within 3 residues on average.
4. Proteins sharing ≥ 25% sequence identity with any protein in the CPDB termini linker dataset (Additional file 6) were further discarded. The resultant dataset (1802 proteins) was thus suitable, as an independent dataset, for assessing the machine learning predictor trained with the CPDB linker dataset.
5. For each protein remaining in this dataset, a CPM was created as stated below.
  (i) Since CP tends to occur at residues exposed to the solvent [17], each residue’s relative solvent accessible surface area (RSA) was computed.
  (ii) Consecutive residues with RSA > 20% were considered an exposed fragment on the surface of the protein. A protein might possess several exposed fragments.
  (iii) Randomly select one exposed fragment, set the CP site at the carboxyl-end of the fragment.
  (iv) Since the native N- and C-termini of the protein were close, they were directly connected in silico. New termini were created at the selected CP site. This step was performed by renumbering residues in the PDB file in a way described in the next subsection.
  (v) Remove the selected exposed fragment from the PDB file.
  (vi) Refine the structure of this CPM by energy minimization using GROMACS [44].

The exposed peptide fragments removed in the above procedure were the known linkers for these in silico synthetic CPMs. A complete list of the PDB entries, CP sites, and linker sequences of this Dataset S is available in Additional file 9.

### Generating the pseudo-circularly-permuted template

Because a CPM usually folded into a structure similar to the native structure [1, 9], the native structure would be a good template when modeling a CPM. However, since the termini of the CPM had changed, the native structure should be manipulated first. CP of a protein structure is actually an amino acid sequence rearrangement such that the N- and C-terminal proportions of the sequence were interchanged. As illustrated in Fig. 5b, making a circularly permuted protein in silico was equivalent to cutting the N-terminal proportion and attaching it to the end of the C-terminal proportion. At the sequence level, this manipulation was a simple text string rearrangement. At the structure level with a PDB file, as described below, it was more complicated.

1. To ensure that the PDB file’s residues appeared in the same order in the template and model structures, starting with 1, assign serial numbers to the residues from the top of the file.
2. If the CP site was *n*, move *n* − 1 residues from the top of the file to the bottom.
3. Starting with 1, renumber all residues and atoms from the top of the file.
4. In case there were missing or structurally undetermined atoms in the PDB file, the reduce [46] and teLeap [47] programs were utilized to add/restore them.

### Comparative structure modeling

The comparative modeling procedure of CirPred consisted of three major steps. First, the target CPM sequence was generated in the way stated above, unless provided by the user. For instance, Mode 2 of the CirPred web server required the user to input the sequence of CPM. Second, the global sequence alignment between the target CPM and template (the pseudo-CP template) was obtained using three methods: the algorithm combining amino acid sequence and secondary structure information that we developed for (PS)^2^ [21], the Smith-Waterman algorithm [31], and the Stretcher program [32]. Among the alignment results, the one with the largest number of aligned residues was selected. Third, comparative structure modeling was performed using the (PS)^2^ procedure according to the sequence alignment. In addition to these three pipelined steps, according to the requirements of experiments or web users, the linker design procedure might be integrated into the pipeline (Fig. 1); and, at the end of the pipeline, model refinement and MD simulation procedures might be activated.

### Linker design

#### Overview of the linker design protocol

The linker design algorithm of CirPred utilized several machine learning and structural modeling methods. The basic idea was to coarsely determine the position of residues of a linker at first and then predict for each residue position the amino acid. The whole protocol is outlined as follows,

1. *Determination of the coarse residue positions of a linker.*
  (i) Let *l* denote the number of residues of the linker.
  (ii) Randomly make *t* temporary linkers according to amino acid propensities of known CPM linkers (Additional file 11).
  (iii) Before making sequence alignment between the target CPM and the pseudo-CP template, insert each temporary linker into the target CPM.
  (iv) Based on the sequence alignment with each temporary linker inserted, generate a coarse model of the target CPM using Modeller, which also computed its DOPE (discrete optimized protein energy) score [43].
  (v) Pick up *t'* from the *t* coarse models with the lowest energy scores for the next step. In this study, *t* and *t'* were set to be 200 and 20, respectively.
2. *Prediction of the amino acid composition of the linker.*
  (i) Take one coarse model of the target CPM, for each residue position of the linker, compute the feature values required to perform predictions.
  (ii) For each residue position, predict the occurrence probabilities of 20 amino acids by machine learning. See later subsections for algorithms of the feature set and machine learning techniques we applied.
3. *Amino acid sequence assignments of the linker.* According to the occurrence probabilities of amino acids, randomly assign an amino acid to each residue position of the linker. For instance, if the probabilities of valine and leucine at position 1 were 70% and 30%, respectively, the chance that position 1 was assigned with a valine was 70%, while the chance of leucine was 30%. Repeat this step for *k* times, creating *k* candidate linker sequences (*k* = 10 in this study).
4. *Generation of a candidate linker sequence pool*. Repeat steps 2 and 3 until all coarse models were applied. There would be *t'* × *k* candidate linker sequences generated to form the pool.
5. *Computation of energy score for linkers in the pool.*
  (i) Take one candidate linker, insert it into the target CPM sequence, and make a sequence alignment with the pseudo-CP template.
  (ii) Based on the alignment, generate *m* models of the target CPM, each with a DOPE score using Modeller [43] (*m* = 10 in this study).
  (iii) Select the model with the lowest DOPE to represent the quality of this candidate linker.
  (iv) Repeat (i) to (iii) until all candidate linkers in the pool were processed.
6. *Selection of the final candidate linker(s).* Find the candidate linker(s) with the lowest energy score(s) to be the final designed linker(s). For all experiments of this study, except the ones of Additional file 8, only one best-designed linker was taken. In our web implementation, several candidate linkers with low energy scores are reported to the user.

### The feature set for prediction

We speculated that if the position of a residue of interest is known in a protein structure, information (or “features”) obtained from its surroundings could be utilized to predict what amino acid the residue is. For a known linker, its residue positions were readily available in the PDB file. For a linker to be designed, residue positions could be coarsely simulated as described above. Features obtained from known linkers were used to train the machine learning kernels for predicting the amino acid composition of a linker to be designed. The feature set used in this study comprised 20 features computed according to this equation,

1 $$F_{A} (i) = \sum\limits_{a = 1}^{{n_{A} (i,d_{r} )}} {\frac{1}{{d_{ia}^{2} }}}$$

where *i* denotes the residue of interest, *A* represents the type of amino acid, *F*_*A*_(*i*) means the feature value of amino acid *A* for *i*, *a* stood for a residue of *A* surrounding *i*, and *d*_*ia*_ is the distance between residues *i* and *a*. The *n*_*A*_(*i*, *d*_*r*_) is the number of *A* residues located within the radius of *d*_*r*_ (which was set as 20 Å in this work) from *i*. The position of a residue is represented by its Cα.

For a given *A*, this feature describes how many and how close this type of amino acids appeared near the residue of interest. A high feature value means that many such amino acids are nearby, or the distances between them and the residue of interest are short. Because the sequence of a linker to be designed is unknown, there is no prior knowledge of the amino acids adjacent to each other. Therefore, when computing the feature values, no matter for a known linker or a linker to be designed, five adjacent residues before and five after the residue of interest are discarded.

### Establishing the predictor by machine learning methods

For any residue in a known linker, in addition to feature values, the “answer” must be provided for machine learning. The answer in the case of linker design should be the residue’s amino acid, and thus there should be 20 candidate answers for learning and prediction. The computation loading of most machine learning algorithms would increase significantly as the number of candidate answers increases. Since 20 answers were beyond our hardware’s computation capacity, we classified 20 amino acids into three types (hydrophilic, hydrophobic, and neutral) and reduced the number of candidate answers to 3.

Previously as we studied viable CP sites, we developed an artificial intelligence system that integrated several machine learning, random sampling, and parameter optimization algorithms [16, 17]. This system was applied in this work, and the recruited algorithms included bootstrap sampling, decision tree, and artificial neural network. After obtaining the answers and feature values from a set of known linkers, 250 and 50 bootstrap samples were made to train minor predictors of decision tree and artificial neural network, respectively. The final predictor was then formed by collecting the minor ones, which made predictions by vote. With this procedure, the probabilities of candidate answers for a given case could be estimated as the proportions of votes the answers received.

For preliminarily assessing the performance of the feature set we designed, tenfold cross-validation was performed using the CPDB linker dataset. The average accuracy (rate of correct predictions) was 67.5%. In this preliminary test, for each testing case, only the best-voted candidate answer was considered. However, in the actual application, the probabilities of candidate answers played a prominent role. This procedure constituted just a part of the CirPred linker design protocol (see the previous subsection). A thorough assessment of the complete linker design protocol had been carried out, and the performance is stated in “Results” section.

### Tuning the probability estimate of amino acids in a designed linker

Since the 20 amino acids were reduced to 3 classes for machine learning, the predicted amino acid probability estimates were sketchy. Before these estimates could be used in the actual linker design protocol, they should be restored to 20 amino acids.

Linkers of the training dataset had been analyzed, such that for each class, proportions of amino acids were known. After the “class-level” prediction, the occurrence probabilities of 20 amino acids at a given residue were finely estimated using the equation,

2 $$pe\left(A\right)=pe(C)\times p(A|C)$$

where *pe*() and *p*() stand for the probability estimate and proportion, respectively, and *A* and *C* denote amino acid and class. For example, if the probability estimate of the hydrophilic class for a residue is 0.80 and the proportion of aspartic acid in this class is 0.25, the probability estimate of aspartic acid at this position is 0.80 × 0.25 = 0.20.

### Length estimate of the linker

In our experiments, the length of a linker to be designed could be obtained from the alignment between CPMs. However, before using the CirPred server for linker design, the user might not know how many residues there should be in the linker. Hence, we proposed an algorithm to estimate the length of the linker to be designed. For a protein with a distance of N- and C-termini (represented by Cα atoms) < 20 Å, the length of the linker would be estimated using this equation,

3 $$l=\mathrm{Round}\left(21.8\times \mathrm{ln}\left(b\right)-52.5\right);\,\,\, l\ge 0$$

where *l* and *b* denote the number of residues of the linker and the distance of termini, respectively. This equation was established according to CPDB. The 20 Å cutoff was determined based on the fact that the length of known linkers (*l*) and the distance of the termini they bridged (*b*) fit the equation within it and that for termini more distant than it, there seemed no rule between *l* and *b* (Additional file 12). For proteins with a long distance between the termini, we proposed the following algorithm to estimate the length of the linker,

1. Starting with *l* = 20 (residues), make *t* temporary linkers of length *l* based on the amino acid propensities of the CPDB linker dataset (Additional file 11).
2. Add each temporary linker to the target CPM; according to the sequence alignment between the target CPM and the pseudo-CP template, generate a model of the CPM and compute its energy score.
3. Among the *t* temporary linkers, find the one with the lowest energy score to represent linkers of length *l*.
4. Increase *l* by five residues and repeat steps 1–3 until *l* reaches a given maximum. We empirically suggest the maximum be 1/5 of the size of the target protein.
5. Find the value of *l* producing the lowest energy score. Scan the length range from *l* − 4 to *l* + 4 and compute these lengths’ energy score by repeating steps 1–3.
6. The length of the linker to be designed is estimated as the length of temporary linkers achieving the lowest energy score.

For evaluating this algorithm, proteins of the CPDB linker dataset with linker length ≥ 20 residues were tested with the DOPE energy score. The average difference in length between the known and designed linkers was 20.3%. The performance of this algorithm was acceptable; it was very time-consuming, however. In the CirPred web server, Eq. (3) is applied by default unless the user changes it.

### Evaluation of the linker design protocol by multiple rounds of independent test

Using the CPDB linker dataset (Additional file 6), we performed an independent test of 500 rounds to evaluate the proposed linker design protocol. This procedure was an improved tenfold cross-validation test. It ensured the independence between the training and testing data for each round and reduced the imprecision of evaluation. The training datasets, independent test datasets, and results of each round are available in Additional file 7. The procedure of this test is provided below,

1. Since one protein might have multiple CPMs in CPDB, all CPDB linkers were grouped according to their PDB entries.
2. Repeat the following steps 500 times,
  (i) Randomly divide the grouped linkers into a training Dataset T and an independent test Dataset I possessing 90% and 10% of the proteins.
  (ii) For ensuring that Dataset I was highly different from Dataset T, any protein in Dataset I sharing ≥ 15% sequence identity with any protein from Dataset T was discarded.
  (iii) A machine-learning linker predictor was established using Dataset T as the training data based on the CirPred linker design protocol.
  (iv) For each case in Dataset I, remove the known linker from the protein, make a pseudo CPM, and then input this pseudo CPM to the CirPred system to redesign the linker using the machine-learning linker predictor established in the previous step. Sequence similarities and the difference of potential energy scores between the designed and known linkers were computed using the BLOSUM45 matrix [48] and GROMACS [44].
3. Statistically analyze the sequence similarity and energy data obtained from the 500 rounds.

### Refinement of the generated model

As shown in Fig. 1, a protein is virtually divided by the CP site (equivalent to the middle point of the linker of the native protein), i.e., the hinge, into two proportions. If the proportions have the same size, take the C-terminal proportion to be the small one. An axis *r* is formed between the hinge and the center of mass of the small proportion. Besides, a plane *P* is defined by these two points and the center of mass of the large proportion. Then, two kinds of movements are made to the small proportion. First, with the hinge fixed, rotate the small proportion on plane *P* with a pause per 20 degrees. Second, at each pause, rotate it around axis *r* with a snapshot per 20 degrees. There are 162 snapshots in total (180°/20° × 360°/20°). Finally, compute the energy score of these snapshots and select the one with the lowest energy to be the refined model of the CPM.

### Molecular dynamics simulations

Molecular dynamics simulations were applied as a final optimization of the model. The model was submerged in a box filled with water molecules. When necessary, a suitable amount of Na^+^ or Cl^–^ ions were added into the box to neutralize the system’s charge. The neutralized system was first energy minimized before the full MD simulations, in which two rounds of annealing, each with temperature points 298 K, 320 K, and 298 K, were performed. Without applying the CP-site-hinge model refinement described above, the number of steps made in MD was set as 5 million, and the step size was two femtoseconds (total simulation time = 10 ns). If the model refinement was applied, we found that 0.5 million steps were enough to produce results with equivalent quality in terms of the alignment ratio and RMSD between the actual and modeled structures. In all modeling experiments performed in this study, both 5 and 0.5 million time steps were tested, and the reported data were based on the 0.5-million-step results. To reduce server machines’ loading, we set the default number of time steps as 0.1 million in the implemented CirPred server, and the user could change the setting.

## Supplementary Information

**Additional file 1: Data S1.** Modeling results of SWISS-MODEL, RaptorX, and Robetta for viable circular permutants of the DHFR.

**Additional file 2: Fig. S1.** Co-linear modeling quality of the CirPred and several state-of-the-art modeling methods.

**Additional file 3: Data S2.** The CP pair dataset.

**Additional file 4: Fig. S2.** Models constructed by CirPred for circular permutants with high identity, low identity, or large sizes.

**Additional file 5: Tables S1, S2.** Performance of CirPred for proteins of various sizes and various CP site positions.

**Additional file 6: Data S3.** The CPDB linker dataset. A full list of termini linkers of all circular permutants from the CP pair dataset (Additional file 3).

**Additional file 7: Data S4.** The training datasets, independent datasets, and results of the 500-round independent test for the linker design protocol of CirPred.

**Additional file 8: Fig. S3, Table S3.** Performance of the linker design algorithm of CirPred for proteins with various termini distances.

**Additional file 9: Data S5.** Dataset S. A dataset of 2,141 in silico synthetic circular permutants, each with a known native protein and a predefined missing linker. Every protein in this dataset shares <25% sequence identity with any other protein either from itself or the CPDB linker dataset (Additional file 6).

**Additional file 10: Discussion.** Advanced discussions on the utilization, limitation, and future developments of the CirPred method.

**Additional file 11: Table S4.** Amino acid propensities of the CPDB linker dataset.

**Additional file 12: Fig. S4.** Relation between the length of linkers and the distance of the termini they bridged.

## Data Availability

All data generated or analyzed during this study are included in this published article and its supplementary information files.

## Abbreviations

CPM: Circular permutant. A circularly permuted protein variant (artificially engineered) or a circularly permuted homolog (engineered or naturally occurring).
CP pair: A pair of proteins related by a circular permutation. Note that in a CP pair, one protein is the CPM of the other
