# Supplementary material for: CirPred, the first structure modeling and linker design system for circularly permuted proteins
Source: BMC Bioinformatics. 2021 Oct 12;22(Suppl 10):494. doi: 10.1186/s12859-021-04403-1 (PMC8513176; doi:10.1186/s12859-021-04403-1)

Models constructed by SWISS-MODEL for viable circular permutants of the dihydrofolate reductase (PDB:1rx4)

- References

  - SWISS-MODEL

    - Benkert P, Biasini M, Schwede T: **Toward the estimation of the absolute quality of individual protein structure models.** *Bioinformatics* 2011, **27:**343-350.
    - Bertoni M, Kiefer F, Biasini M, Bordoli L, Schwede T: **Modeling protein quaternary structure of homo- and hetero-oligomers beyond binary interactions by homology.** *Sci Rep* 2017, **7:**10480.
    - Bienert S, Waterhouse A, de Beer TA, Tauriello G, Studer G, Bordoli L, Schwede T: **The SWISS-MODEL Repository-new features and functionality.** *Nucleic Acids Res* 2017, **45:**D313-D319.
    - Guex N, Peitsch MC, Schwede T: **Automated comparative protein structure modeling with SWISS-MODEL and Swiss-PdbViewer: a historical perspective.** *Electrophoresis* 2009, **30 Suppl 1:**S162-173.
    - Waterhouse A, Bertoni M, Bienert S, Studer G, Tauriello G, Gumienny R, Heer FT, de Beer TAP, Rempfer C, Bordoli L, et al: **SWISS-MODEL: homology modelling of protein structures and complexes.** *Nucleic Acids Res* 2018, **46:**W296-W303.

  - BLAST

    - Camacho C, Coulouris G, Avagyan V, Ma N, Papadopoulos J, Bealer K, Madden TL: **BLAST+: architecture and applications.** *BMC Bioinformatics* 2009, **10:**421.

  - HHblits

    - Remmert M, Biegert A, Hauser A, Soding J: **HHblits: lightning-fast iterative protein sequence searching by HMM-HMM alignment.** *Nat Methods* 2011, **9:**173-175.

Template: PDB 1rx4A

CP site: Met1

Target sequence:

MISLIAALAVDRVIGMENAMPWNLPADLAWFKRNTLDKPVIMGRHTWESIGRPLPGRKNI
ILSSQPGTDDRVTWVKSVDEAIAACGDVPEIMVIGGGRVYEQFLPKAQKLYLTHIDAEVE
GDTHFPDYEPDDWESVFSEFHDADAQNSHSYCFEILERRGGGGG

Model #01	File	Built with	Oligo-State	Ligands	GMQE	QMEAN
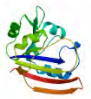	PDB	ProMod3 Version 1.2.0.	monomer	None	0.99	0.02

QMEAN	0.02	
Cβ	-0.15	
All Atom	0.33	
Solvation	-0.14	
Torsion	0.05	

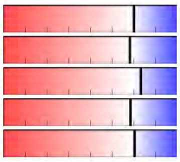

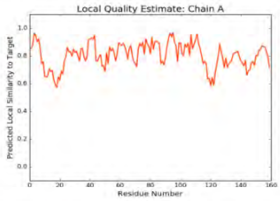

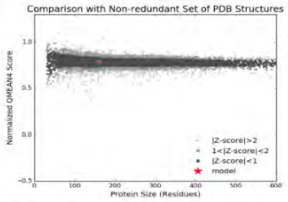

Template	Seq Identity	Oligo-state	Found by	Method	Resolution	Seq Similarity	Range	Coverage	Description
template_upload.1.A	100.00	monomer	HHblits	Unknown	NA	0.62	1 - 159	0.97	Polypeptide

The template contained no ligands.

```
Target                  MISLIAALAVDRVIGMENAMPWNLPADLAWFKRNTLDKPVIMGRHTWESIGRPLP
template_upload.1.A     MISLIAALAVDRVIGMENAMPWNLPADLAWFKRNTLDKPVIMGRHTWESIGRPLP

Target                  GRKNIILSSQPGTDDRVTWVKSVDEAIAACGDVPEIMVIGGGRVYEQFLPKAQKL
template_upload.1.A     GRKNIILSSQPGTDDRVTWVKSVDEAIAACGDVPEIMVIGGGRVYEQFLPKAQKL

Target                  YLTHIDAEVEGDTHFPDYEPDDWESVFSEFHDADAQNSHSYCFEILERRGGGGG
template_upload.1.A     YLTHIDAEVEGDTHFPDYEPDDWESVFSEFHDADAQNSHSYCFEILERR-----
```

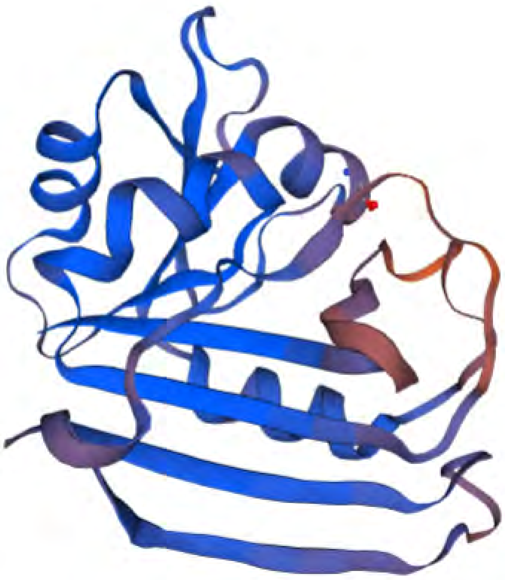

Template: PDB 1rx4A

CP site: Ile2

Target sequence:

```
ISLIAALAVDRVIGMENAMPWNLPADLAWFKRNTLDKPVIMGRHTWESIGRPLPGRKNII
LSSQPGTDDRVTWVKSVDEAIAACGDVPEIMVIGGGRVYEQFLPKAQKLYLTHIDAEVEG
DTHFPDYEPDDWESVFSEFHDADAQNSHSYCFEILERRGGGGGM
```

Model #01	File	Built with	Oligo-State	Ligands	GMQE	QMEAN
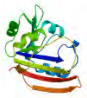	PDB	ProMod3 Version 1.2.0.	monomer	None	0.99	0.16

QMEAN	0.16	
Cβ	-0.09	
All Atom	0.42	
Solvation	-0.01	
Torsion	0.15	

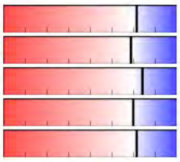

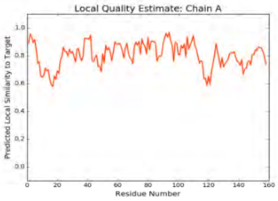

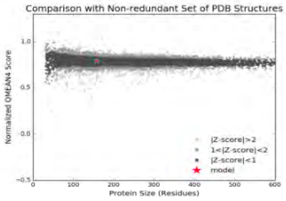

Template	Seq Identity	Oligo-state	Found by	Method	Resolution	Seq Similarity	Range	Coverage	Description
template_upload.1.A	100.00	monomer	HHblits	Unknown	NA	0.62	1 - 158	0.96	Polypeptide

The template contained no ligands.

```
Target                      ISLIAALAVDRVIGMENAMPWNLPADLAWFKRNTLDKPVIMGRHTWESIGRPLPG
template_upload.1.A         ISLIAALAVDRVIGMENAMPWNLPADLAWFKRNTLDKPVIMGRHTWESIGRPLPG

Target                      RKNIILSSQPGTDDRVTWVKSVDEAIAACGDVPEIMVIGGGRVYEQFLPKAQKLY
template_upload.1.A         RKNIILSSQPGTDDRVTWVKSVDEAIAACGDVPEIMVIGGGRVYEQFLPKAQKLY

Target                      LTHIDAEVEGDTHFPDYEPDDWESVFSEFHDADAQNSHSYCFEILERRGGGGGM
template_upload.1.A         LTHIDAEVEGDTHFPDYEPDDWESVFSEFHDADAQNSHSYCFEILERR------
```

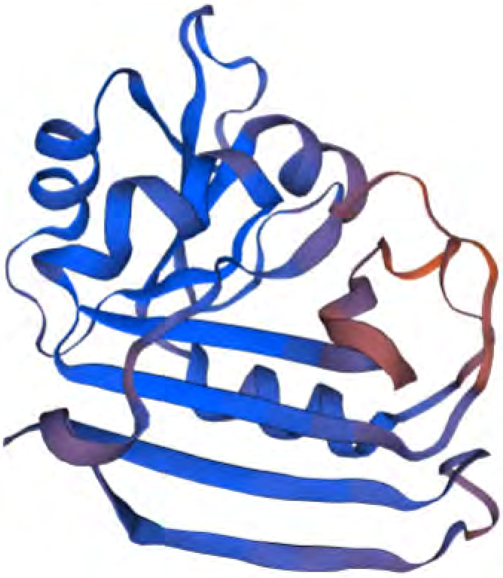

Template: PDB 1rx4A

CP site: Gly15

Target sequence:

```
GMENAMPWNLPADLAWFKRNTLDKPVIMGRHTWESIGRPLPGRKNIILSSQPGTDDRVTW
VKSVDEAIAACGDVPEIMVIGGGRVYEQFLPKAQKLYLTHIDAEVEGDTHFPDYEPDDWE
SVFSEFHDADAQNSHSYCFEILERRGGGGGMISLIAALAVDRVI
```

Model #01	File	Built with	Oligo-State	Ligands	GMQE	QMEAN
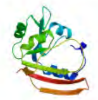	PDB	ProMod3 Version 1.2.0.	monomer	None	0.95	0.64

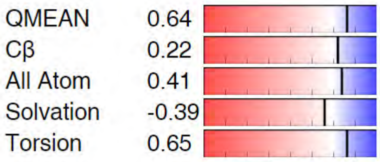

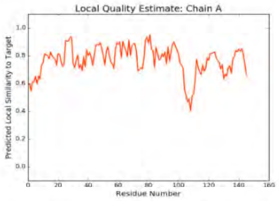

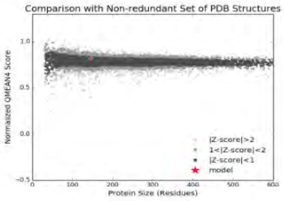

Template	Seq Identity	Oligo-state	Found by	Method	Resolution	Seq Similarity	Range	Coverage	Description
template_upload.1.A	100.00	monomer	HHblits	Unknown	NA	0.63	1 - 145	0.88	Polypeptide

The template contained no ligands.

```
Target                 GMENAMPWNLPADLAWFKRNTLDKPVIMGRHTWESIGRPLPGRKNIILSSQPGTD
template_upload.1.A    GMENAMPWNLPADLAWFKRNTLDKPVIMGRHTWESIGRPLPGRKNIILSSQPGTD

Target                 DRVTWVKSVDEAIAACGDVPEIMVIGGGRVYEQFLPKAQKLYLTHIDAEVEGDTH
template_upload.1.A    DRVTWVKSVDEAIAACGDVPEIMVIGGGRVYEQFLPKAQKLYLTHIDAEVEGDTH

Target                 FPDYEPDDWESVFSEFHDADAQNSHSYCFEILERRGGGGGMISLIAALAVDRVI
template_upload.1.A    FPDYEPDDWESVFSEFHDADAQNSHSYCFEILERR-------------------
```

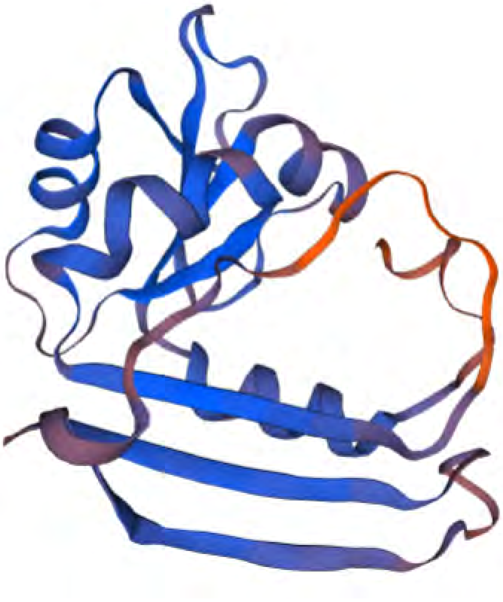

Template: PDB 1rx4A

CP site: Met16

Target sequence:

MENAMPWNLPADLAWFKRNTLDKPVIMGRHTWESIGRPLPGRKNIILSSQPGTDDRVTWV
KSVDEAIAACGDVPEIMVIGGGRVYEQFLPKAQKLYLTHIDAEVEGDTHFPDYEPDDWES
VFSEFHDADAQNSHSYCFEILERRGGGGGMISLIAALAVDRVIG

Model #01	File	Built with	Oligo-State	Ligands	GMQE	QMEAN
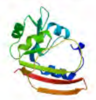	PDB	ProMod3 Version 1.2.0.	monomer	None	0.93	0.64

QMEAN	0.64
Cβ	0.04
All Atom	0.41
Solvation	-0.23
Torsion	0.65

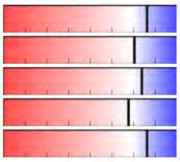

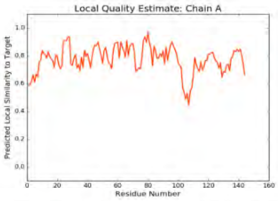

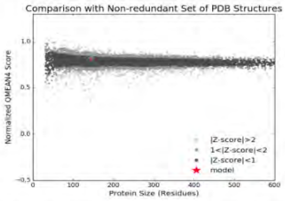

Template	Seq Identity	Oligo-state	Found by	Method	Resolution	Seq Similarity	Range	Coverage	Description
template_upload.1.A	100.00	monomer	HHblits	Unknown	NA	0.63	1 - 144	0.88	Polypeptide

The template contained no ligands.

```
Target                  MENAMPWNLPADLAWFKRNTLDKPVIMGRHTWESIGRPLPGRKNIILSSQPGTDD
template_upload.1.A     MENAMPWNLPADLAWFKRNTLDKPVIMGRHTWESIGRPLPGRKNIILSSQPGTDD

Target                  RVTWVKSVDEAIAACGDVPEIMVIGGGRVYEQFLPKAQKLYLTHIDAEVEGDTHF
template_upload.1.A     RVTWVKSVDEAIAACGDVPEIMVIGGGRVYEQFLPKAQKLYLTHIDAEVEGDTHF

Target                  PDYEPDDWESVFSEFHDADAQNSHSYCFEILERRGGGGGMISLIAALAVDRVIG
template_upload.1.A     PDYEPDDWESVFSEFHDADAQNSHSYCFEILERR--------------------
```

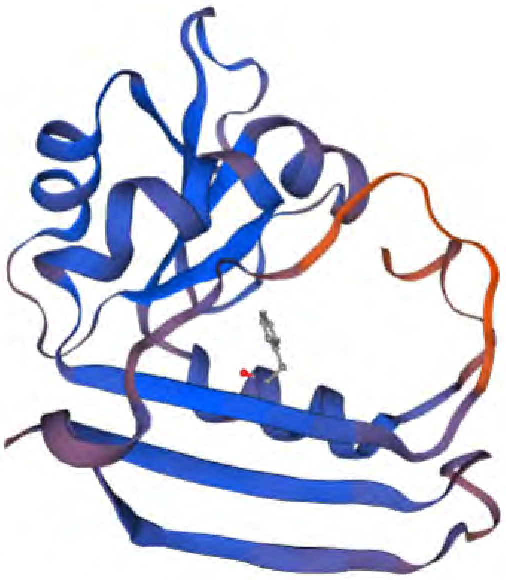

Template: PDB 1rx4A

CP site: Glu17

Target sequence:

ENAMPWNLPADLAWFKRNTLDKPVIMGRHTWESIGRPLPGRKNIILSSQPGTDDRVTWVK
SVDEAIAACGDVPEIMVIGGGRVYEQFLPKAQKLYLTHIDAEVEGDTHFPDYEPDDWESV
FSEFHDADAQNSHSYCFEILERRGGGGGMISLIAALAVDRVIGM

Model #01	File	Built with	Oligo-State	Ligands	GMQE	QMEAN
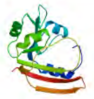	PDB	ProMod3 Version 1.2.0.	monomer	None	0.90	0.66

QMEAN	0.66
Cβ	0.08
All Atom	0.41
Solvation	-0.29
Torsion	0.68

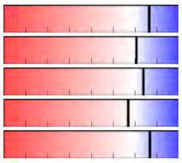

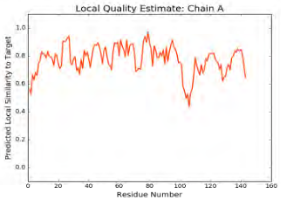

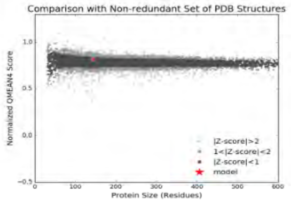

Template	Seq Identity	Oligo-state	Found by	Method	Resolution	Seq Similarity	Range	Coverage	Description
template_upload.1.A	100.00	monomer	HHblits	Unknown	NA	0.63	1 - 143	0.87	Polypeptide

The template contained no ligands.

```
Target                    ENAMPWNLPADLAWFKRNTLDKPVIMGRHTWESIGRPLPGRKNIILSSQPGTDDR
template_upload.1.A       ENAMPWNLPADLAWFKRNTLDKPVIMGRHTWESIGRPLPGRKNIILSSQPGTDDR

Target                    VTWVKSVDEAIAACGDVPEIMVIGGGRVYEQFLPKAQKLYLTHIDAEVEGDTHFP
template_upload.1.A       VTWVKSVDEAIAACGDVPEIMVIGGGRVYEQFLPKAQKLYLTHIDAEVEGDTHFP

Target                    DYEPDDWESVFSEFHDADAQNSHSYCFEILERRGGGGGMISLIAALAVDRVIGM
template_upload.1.A       DYEPDDWESVFSEFHDADAQNSHSYCFEILERR---------------------
```

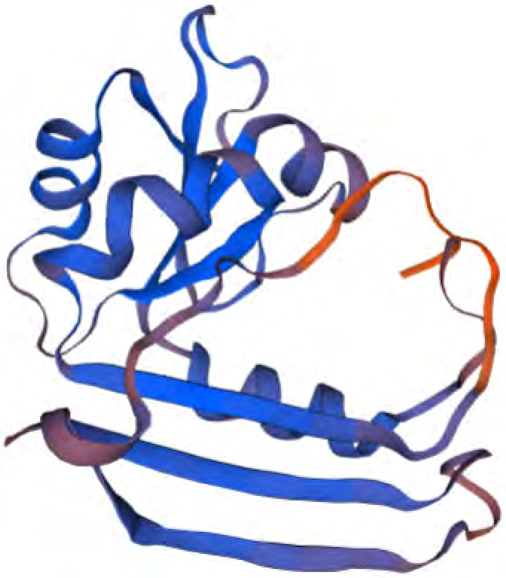

Template: PDB 1rx4A

CP site: Asn18

Target sequence:

NAMPWNLPADLAWFKRNTLDKPVIMGRHTWESIGRPLPGRKNIILSSQPGTDDRVTWVKS
VDEAIAACGDVPEIMVIGGGRVYEQFLPKAQKLYLTHIDAEVEGDTHFPDYEPDDWESVF
SEFHDADAQNSHSYCFEILERRGGGGGMISLIAALAVDRVIGME

Model #01	File	Built with	Oligo-State	Ligands	GMQE	QMEAN
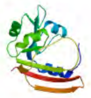	PDB	ProMod3 Version 1.2.0.	monomer	None	0.88	0.52

QMEAN 0.52
Cβ 0.05
All Atom 0.46
Solvation -0.35
Torsion 0.55

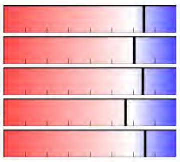

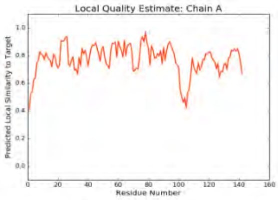

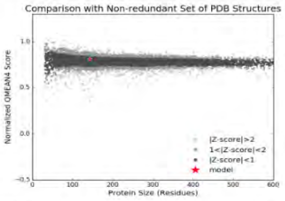

Template	Seq Identity	Oligo-state	Found by	Method	Resolution	Seq Similarity	Range	Coverage	Description
template_upload.1.A	100.00	monomer	HHblits	Unknown	NA	0.63	1 - 142	0.87	Polypeptide

The template contained no ligands.

```
Target                 NAMPWNLPADLAWFKRNTLDKPVIMGRHTWESIGRPLPGRKNIILSSQPGTDDRV
template_upload.1.A    NAMPWNLPADLAWFKRNTLDKPVIMGRHTWESIGRPLPGRKNIILSSQPGTDDRV

Target                 TWVKSVDEAIAACGDVPEIMVIGGGRVYEQFLPKAQKLYLTHIDAEVEGDTHFPD
template_upload.1.A    TWVKSVDEAIAACGDVPEIMVIGGGRVYEQFLPKAQKLYLTHIDAEVEGDTHFPD

Target                 YEPDDWESVFSEFHDADAQNSHSYCFEILERRGGGGGMISLIAALAVDRVIGME
template_upload.1.A    YEPDDWESVFSEFHDADAQNSHSYCFEILERR----------------------
```

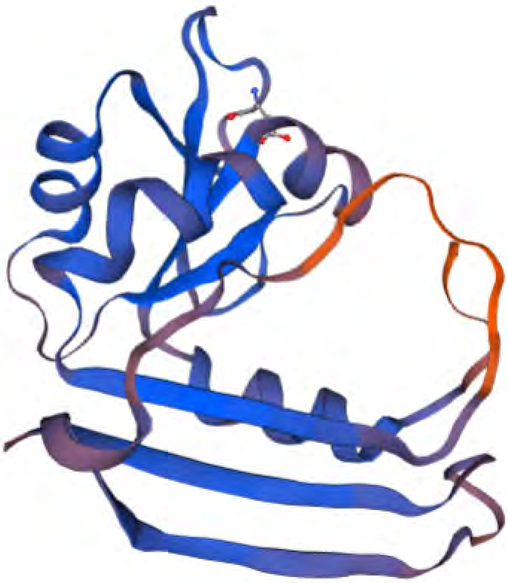

Template: PDB 1rx4A

CP site: Ala19

Target sequence:

AMPWNLPADLAWFKRNTLDKPVIMGRHTWESIGRPLPGRKNIILSSQPGTDDRVTWVKSV
DEAIAACGDVPEIMVIGGGRVYEQFLPKAQKLYLTHIDAEVEGDTHFPDYEPDDWESVFS
EFHDADAQNSHSYCFEILERRGGGGGMISLIAALAVDRVIGMEN

Model #01	File	Built with	Oligo-State	Ligands	GMQE	QMEAN
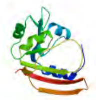	PDB	ProMod3 Version 1.2.0.	monomer	None	0.87	0.63

QMEAN	0.63	
Cβ	0.13	
All Atom	0.48	
Solvation	-0.31	
Torsion	0.63	

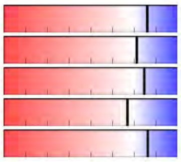

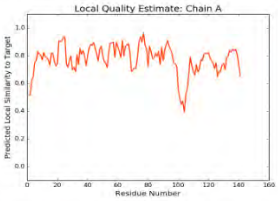

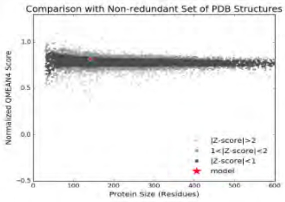

Template	Seq Identity	Oligo-state	Found by	Method	Resolution	Seq Similarity	Range	Coverage	Description
template_upload.1.A	100.00	monomer	HHblits	Unknown	NA	0.63	1 - 141	0.86	Polypeptide

The template contained no ligands.

```
Target                 AMPWNLPADLAWFKRNTLDKPVIMGRHTWESIGRPLPGRKNIILSSQPGTDDRVT
template_upload.1.A    AMPWNLPADLAWFKRNTLDKPVIMGRHTWESIGRPLPGRKNIILSSQPGTDDRVT

Target                 WVKSVDEAIAACGDVPEIMVIGGGRVYEQFLPKAQKLYLTHIDAEVEGDTHFPDY
template_upload.1.A    WVKSVDEAIAACGDVPEIMVIGGGRVYEQFLPKAQKLYLTHIDAEVEGDTHFPDY

Target                 EPDDWESVFSEFHDADAQNSHSYCFEILERRGGGGGMISLIAALAVDRVIGMEN
template_upload.1.A    EPDDWESVFSEFHDADAQNSHSYCFEILERR-----------------------
```

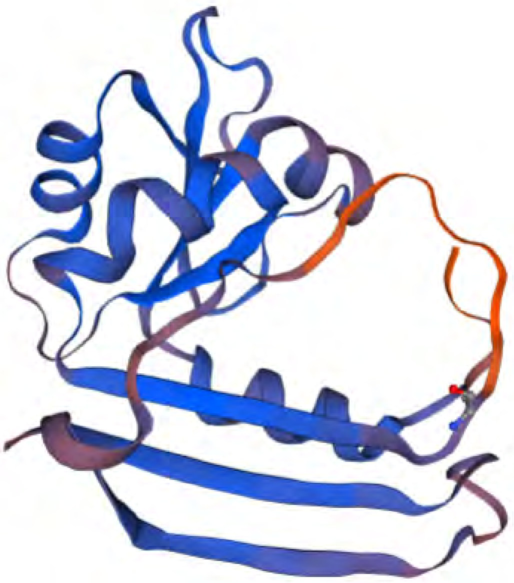

Template: PDB 1rx4A

CP site: Met20

Target sequence:

```
MPWNLPADLAWFKRNTLDKPVIMGRHTWESIGRPLPGRKNIILSSQPGTDDRVTWVKSVD
EAIAACGDVPEIMVIGGGRVYEQFLPKAQKLYLTHIDAEVEGDTHFPDYEPDDWESVFSE
FHDADAQNSHSYCFEILERRGGGGGMISLIAALAVDRVIGMENA
```

Model #01	File	Built with	Oligo-State	Ligands	GMQE	QMEAN
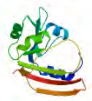	PDB	ProMod3 Version 1.2.0.	monomer	None	0.85	0.66

QMEAN	0.66
Cβ	0.16
All Atom	0.49
Solvation	-0.19
Torsion	0.64

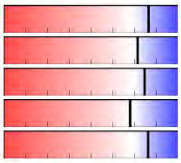

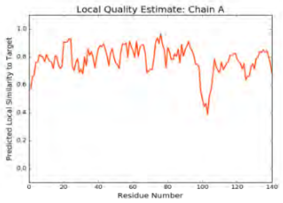

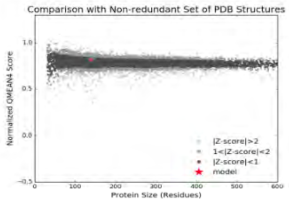

Template	Seq Identity	Oligo-state	Found by	Method	Resolution	Seq Similarity	Range	Coverage	Description
template_upload.1.A	100.00	monomer	HHblits	Unknown	NA	0.63	1 - 140	0.85	Polypeptide

The template contained no ligands.

```
Target                  MPWNLPADLAWFKRNTLDKPVIMGRHTWESIGRPLPGRKNIILSSQPGTDDRVTW
template_upload.1.A     MPWNLPADLAWFKRNTLDKPVIMGRHTWESIGRPLPGRKNIILSSQPGTDDRVTW

Target                  VKSVDEAIAACGDVPEIMVIGGGRVYEQFLPKAQKLYLTHIDAEVEGDTHFPDYE
template_upload.1.A     VKSVDEAIAACGDVPEIMVIGGGRVYEQFLPKAQKLYLTHIDAEVEGDTHFPDYE

Target                  PDDWESVFSEFHDADAQNSHSYCFEILERRGGGGGMISLIAALAVDRVIGMENA
template_upload.1.A     PDDWESVFSEFHDADAQNSHSYCFEILERR------------------------
```

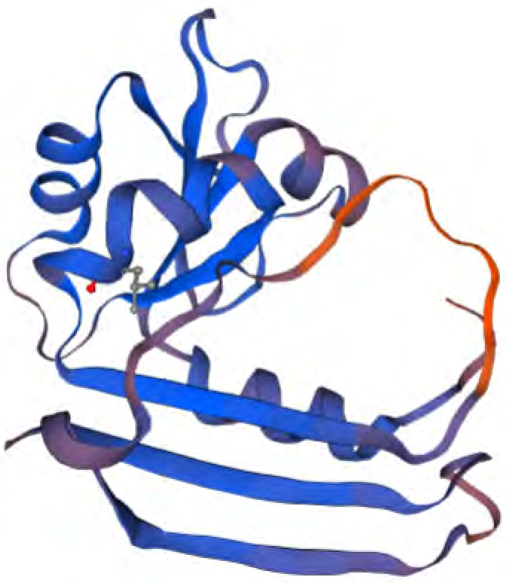

Template: PDB 1rx4A

CP site: Pro21

Target sequence:

```
PWNLPADLAWFKRNTLDKPVIMGRHTWESIGRPLPGRKNIILSSQPGTDDRVTWVKSVDE
AIAACGDVPEIMVIGGGRVYEQFLPKAQKLYLTHIDAEVEGDTHFPDYEPDDWESVFSEF
HDADAQNSHSYCFEILERRGGGGGMISLIAALAVDRVIGMENAM
```

Model #01	File	Built with	Oligo-State	Ligands	GMQE	QMEAN
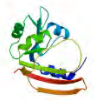	PDB	ProMod3 Version 1.2.0.	monomer	None	0.83	0.62

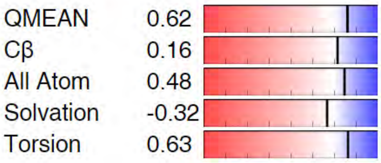

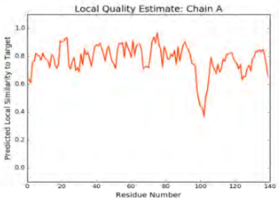

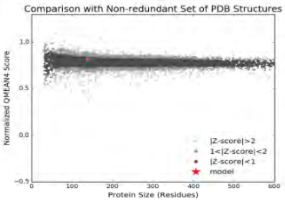

Template	Seq Identity	Oligo-state	Found by	Method	Resolution	Seq Similarity	Range	Coverage	Description
template_upload.1.A	100.00	monomer	HHblits	Unknown	NA	0.63	1 - 139	0.85	Polypeptide

The template contained no ligands.

```
Target                  PWNLPADLAWFKRNTLDKPVIMGRHTWESIGRPLPGRKNIILSSQPGTDDRVTWV
template_upload.1.A     PWNLPADLAWFKRNTLDKPVIMGRHTWESIGRPLPGRKNIILSSQPGTDDRVTWV

Target                  KSVDEAIAACGDVPEIMVIGGGRVYEQFLPKAQKLYLTHIDAEVEGDTHFPDYEP
template_upload.1.A     KSVDEAIAACGDVPEIMVIGGGRVYEQFLPKAQKLYLTHIDAEVEGDTHFPDYEP

Target                  DDWESVFSEFHDADAQNSHSYCFEILERRGGGGGMISLIAALAVDRVIGMENAM
template_upload.1.A     DDWESVFSEFHDADAQNSHSYCFEILERR-------------------------
```

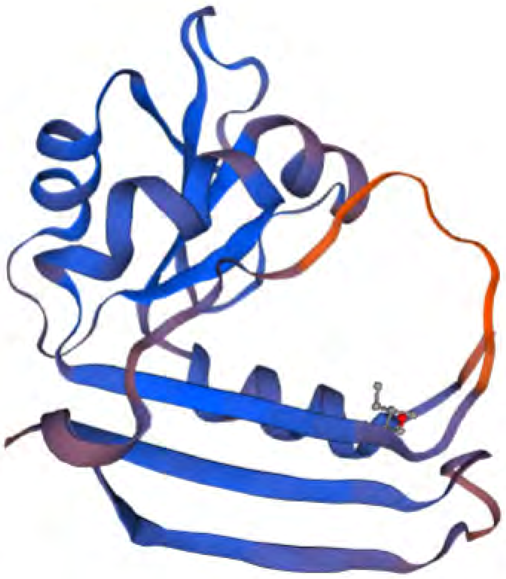

Template: PDB 1rx4A

CP site: Trp22

Target sequence:

```
WNLPADLAWFKRNTLDKPVIMGRHTWESIGRPLPGRKNIILSSQPGTDDRVTWVKSVDEA
IAACGDVPEIMVIGGGRVYEQFLPKAQKLYLTHIDAEVEGDTHFPDYEPDDWESVFSEFH
DADAQNSHSYCFEILERRGGGGGMISLIAALAVDRVIGMENAMP
```

Model #01	File	Built with	Oligo-State	Ligands	GMQE	QMEAN
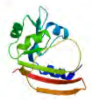	PDB	ProMod3 Version 1.2.0.	monomer	None	0.83	0.60

QMEAN	0.60	
Cβ	0.10	
All Atom	0.44	
Solvation	-0.21	
Torsion	0.60	

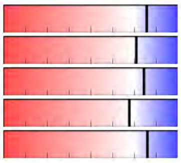

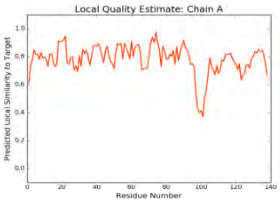

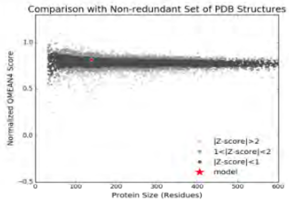

Template	Seq Identity	Oligo-state	Found by	Method	Resolution	Seq Similarity	Range	Coverage	Description
template_upload.1.A	100.00	monomer	HHblits	Unknown	NA	0.63	1 - 138	0.84	Polypeptide

The template contained no ligands.

```
Target                  WNLPADLAWFKRNTLDKPVIMGRHTWESIGRPLPGRKNIILSSQPGTDDRVTWVK
template_upload.1.A     WNLPADLAWFKRNTLDKPVIMGRHTWESIGRPLPGRKNIILSSQPGTDDRVTWVK

Target                  SVDEAIAACGDVPEIMVIGGGRVYEQFLPKAQKLYLTHIDAEVEGDTHFPDYEPD
template_upload.1.A     SVDEAIAACGDVPEIMVIGGGRVYEQFLPKAQKLYLTHIDAEVEGDTHFPDYEPD

Target                  DWESVFSEFHDADAQNSHSYCFEILERRGGGGGMISLIAALAVDRVIGMENAMP
template_upload.1.A     DWESVFSEFHDADAQNSHSYCFEILERR--------------------------
```

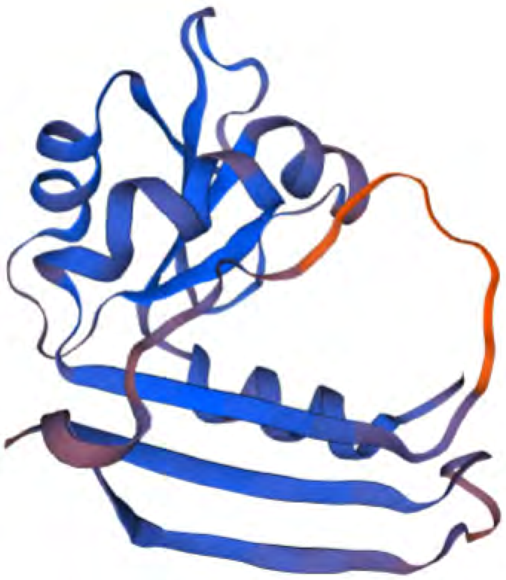

Template: PDB 1rx4A

CP site: Asn23

Target sequence:

```
NLPADLAWFKRNTLDKPVIMGRHTWESIGRPLPGRKNIILSSQPGTDDRVTWVKSVDEAI
AACGDVPEIMVIGGGRVYEQFLPKAQKLYLTHIDAEVEGDTHFPDYEPDDWESVFSEFHD
ADAQNSHSYCFEILERRGGGGGMISLIAALAVDRVIGMENAMPW
```

Model #01	File	Built with	Oligo-State	Ligands	GMQE	QMEAN
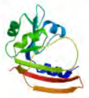	PDB	ProMod3 Version 1.2.0.	monomer	None	0.80	0.42

QMEAN	0.42	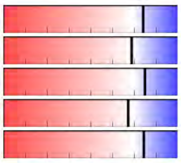
Cβ	-0.11	
All Atom	0.52	
Solvation	-0.27	
Torsion	0.46	

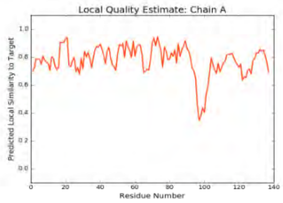

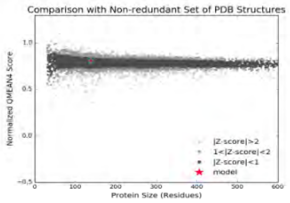

Template	Seq Identity	Oligo-state	Found by	Method	Resolution	Seq Similarity	Range	Coverage	Description
template_upload.1.A	100.00	monomer	HHblits	Unknown	NA	0.63	1 - 137	0.84	Polypeptide

The template contained no ligands.

```
Target                    NLPADLAWFKRNTLDKPVIMGRHTWESIGRPLPGRKNIILSSQPGTDDRVTWVKS
template_upload.1.A       NLPADLAWFKRNTLDKPVIMGRHTWESIGRPLPGRKNIILSSQPGTDDRVTWVKS

Target                    VDEAIAACGDVPEIMVIGGGRVYEQFLPKAQKLYLTHIDAEVEGDTHFPDYEPDD
template_upload.1.A       VDEAIAACGDVPEIMVIGGGRVYEQFLPKAQKLYLTHIDAEVEGDTHFPDYEPDD

Target                    WESVFSEFHDADAQNSHSYCFEILERRGGGGGMISLIAALAVDRVIGMENAMPW
template_upload.1.A       WESVFSEFHDADAQNSHSYCFEILERR---------------------------
```

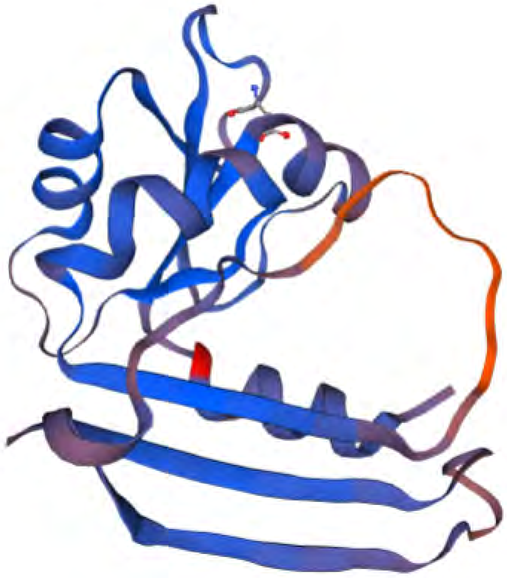

Template: PDB 1rx4A

CP site: Leu24

Target sequence:

LPADLAWFKRNTLDKPVIMGRHTWESIGRPLPGRKNIILSSQPGTDDRVTWVKSVDEAIA
ACGDVPEIMVIGGGRVYEQFLPKAQKLYLTHIDAEVEGDTHFPDYEPDDWESVFSEFHDA
DAQNSHSYCFEILERRGGGGGMISLIAALAVDRVIGMENAMPWN

Model #01	File	Built with	Oligo-State	Ligands	GMQE	QMEAN
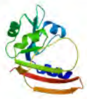	PDB	ProMod3 Version 1.2.0.	monomer	None	0.77	0.53

QMEAN	0.53	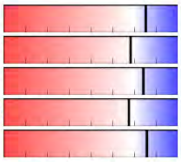
Cβ	-0.17	
All Atom	0.43	
Solvation	-0.25	
Torsion	0.59	

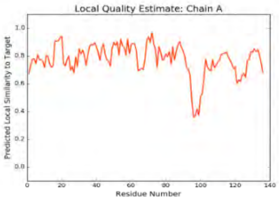

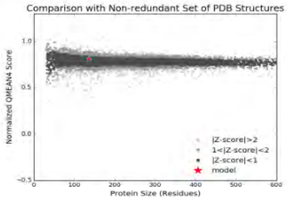

Template	Seq Identity	Oligo-state	Found by	Method	Resolution	Seq Similarity	Range	Coverage	Description
template_upload.1.A	100.00	monomer	HHblits	Unknown	NA	0.62	1 - 136	0.83	Polypeptide

The template contained no ligands.

```
Target                LPADLAWFKRNTLDKPVIMGRHTWESIGRPLPGRKNIILSSQPGTDDRVTWVKSV
template_upload.1.A   LPADLAWFKRNTLDKPVIMGRHTWESIGRPLPGRKNIILSSQPGTDDRVTWVKSV

Target                DEAIAACGDVPEIMVIGGGRVYEQFLPKAQKLYLTHIDAEVEGDTHFPDYEPDDW
template_upload.1.A   DEAIAACGDVPEIMVIGGGRVYEQFLPKAQKLYLTHIDAEVEGDTHFPDYEPDDW

Target                ESVFSEFHDADAQNSHSYCFEILERRGGGGGMISLIAALAVDRVIGMENAMPWN
template_upload.1.A   ESVFSEFHDADAQNSHSYCFEILERR----------------------------
```

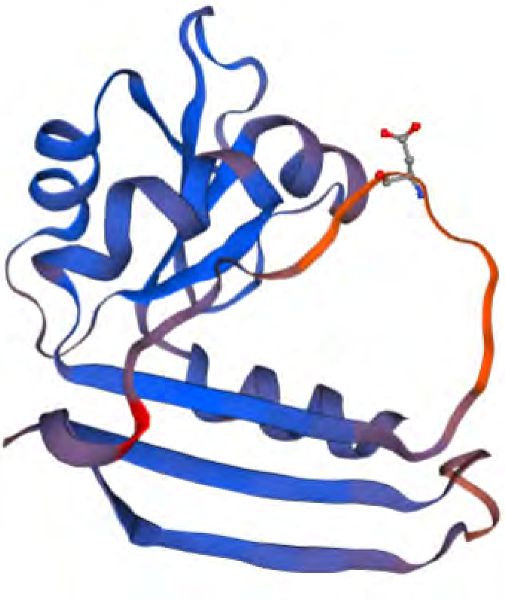

Template: PDB 1rx4A

CP site: Pro25

Target sequence:

PADLAWFKRNTLDKPVIMGRHTWESIGRPLPGRKNIILSSQPGTDDRVTWVKSVDEAIAA
CGDVPEIMVIGGGRVYEQFLPKAQKLYLTHIDAEVEGDTHFPDYEPDDWESVFSEFHDAD
AQNSHSYCFEILERRGGGGGMISLIAALAVDRVIGMENAMPWNL

Model #01	File	Built with	Oligo-State	Ligands	GMQE	QMEAN
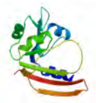	PDB	ProMod3 Version 1.2.0.	monomer	None	0.76	0.46

QMEAN	0.46	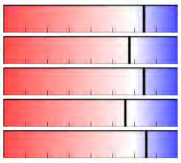
Cβ	-0.20	
All Atom	0.44	
Solvation	-0.40	
Torsion	0.55	

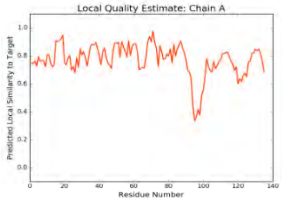

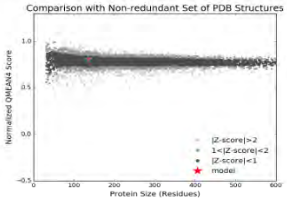

Template	Seq Identity	Oligo-state	Found by	Method	Resolution	Seq Similarity	Range	Coverage	Description
template_upload.1.A	100.00	monomer	HHblits	Unknown	NA	0.63	1 - 135	0.82	Polypeptide

The template contained no ligands.

```
Target                      PADLAWFKRNTLDKPVIMGRHTWESIGRPLPGRKNIILSSQPGTDDRVTWVKSVD
template_upload.1.A         PADLAWFKRNTLDKPVIMGRHTWESIGRPLPGRKNIILSSQPGTDDRVTWVKSVD

Target                      EAIAACGDVPEIMVIGGGRVYEQFLPKAQKLYLTHIDAEVEGDTHFPDYEPDDWE
template_upload.1.A         EAIAACGDVPEIMVIGGGRVYEQFLPKAQKLYLTHIDAEVEGDTHFPDYEPDDWE

Target                      SVFSEFHDADAQNSHSYCFEILERRGGGGGMISLIAALAVDRVIGMENAMPWNL
template_upload.1.A         SVFSEFHDADAQNSHSYCFEILERR-----------------------------
```

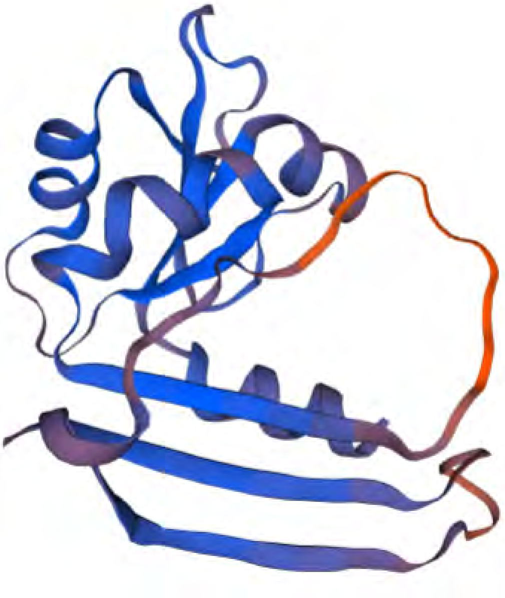

Template: PDB 1rx4A

CP site: Ala26

Target sequence:

ADLAWFKRNTLDKPVIMGRHTWESIGRPLPGRKNIILSSQPGTDDRVTWVKSVDEAIAAC
GDVPEIMVIGGGRVYEQFLPKAQKLYLTHIDAEVEGDTHFPDYEPDDWESVFSEFHDADA
QNSHSYCFEILERRGGGGGMISLIAALAVDRVIGMENAMPWNLP

Model #01	File	Built with	Oligo-State	Ligands	GMQE	QMEAN
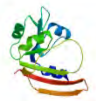	PDB	ProMod3 Version 1.2.0.	monomer	None	0.74	0.43

QMEAN	0.43	
Cβ	-0.25	
All Atom	0.42	
Solvation	-0.44	
Torsion	0.54	

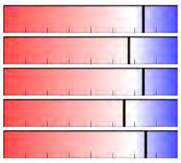

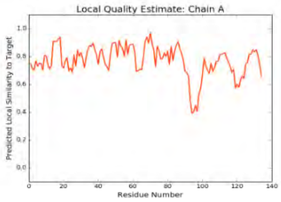

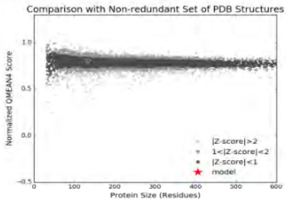

Template	Seq Identity	Oligo-state	Found by	Method	Resolution	Seq Similarity	Range	Coverage	Description
template_upload.1.A	100.00	monomer	HHblits	Unknown	NA	0.63	1 - 134	0.82	Polypeptide

The template contained no ligands.

```
Target                   ADLAWFKRNTLDKPVIMGRHTWESIGRPLPGRKNIILSSQPGTDDRVTWVKSVDE
template_upload.1.A      ADLAWFKRNTLDKPVIMGRHTWESIGRPLPGRKNIILSSQPGTDDRVTWVKSVDE

Target                   AIAACGDVPEIMVIGGGRVYEQFLPKAQKLYLTHIDAEVEGDTHFPDYEPDDWES
template_upload.1.A      AIAACGDVPEIMVIGGGRVYEQFLPKAQKLYLTHIDAEVEGDTHFPDYEPDDWES

Target                   VFSEFHDADAQNSHSYCFEILERRGGGGGMISLIAALAVDRVIGMENAMPWNLP
template_upload.1.A      VFSEFHDADAQNSHSYCFEILERR------------------------------
```

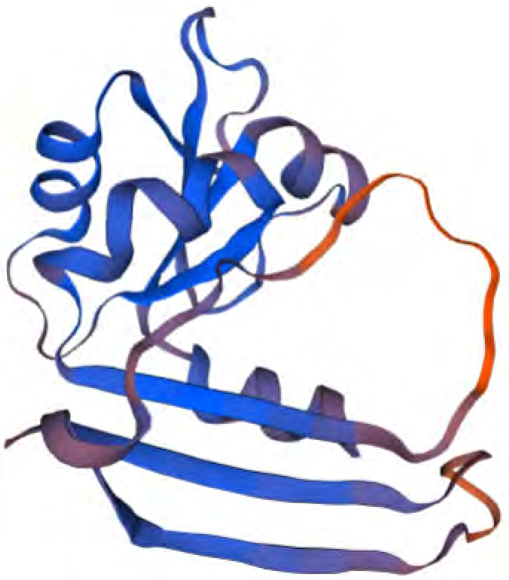

Template: PDB 1rx4A

CP site: Asp27

Target sequence:

DLAWFKRNTLDKPVIMGRHTWESIGRPLPGRKNIILSSQPGTDDRVTWVKSVDEAIAACG
DVPEIMVIGGGRVYEQFLPKAQKLYLTHIDAEVEGDTHFPDYEPDDWESVFSEFHDADAQ
NSHSYCFEILERRGGGGGMISLIAALAVDRVIGMENAMPWNLPA

Model #01	File	Built with	Oligo-State	Ligands	GMQE	QMEAN
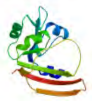	PDB	ProMod3 Version 1.2.0.	monomer	None	0.75	0.48

QMEAN	0.48
Cβ	0.04
All Atom	0.38
Solvation	-0.38
Torsion	0.52

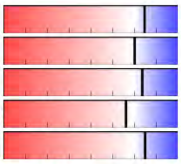

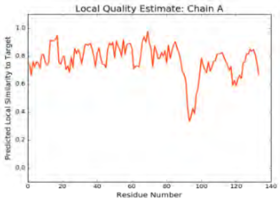

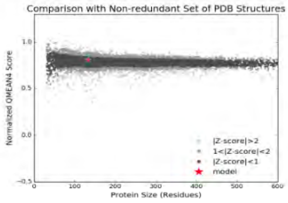

Template	Seq Identity	Oligo-state	Found by	Method	Resolution	Seq Similarity	Range	Coverage	Description
template_upload.1.A	100.00	monomer	HHblits	Unknown	NA	0.63	1 - 133	0.81	Polypeptide

The template contained no ligands.

```
Target                  DLAWFKRNTLDKPVIMGRHTWESIGRPLPGRKNIILSSQPGTDDRVTWVKSVDEA
template_upload.1.A     DLAWFKRNTLDKPVIMGRHTWESIGRPLPGRKNIILSSQPGTDDRVTWVKSVDEA

Target                  IAACGDVPEIMVIGGGRVYEQFLPKAQKLYLTHIDAEVEGDTHFPDYEPDDWESV
template_upload.1.A     IAACGDVPEIMVIGGGRVYEQFLPKAQKLYLTHIDAEVEGDTHFPDYEPDDWESV

Target                  FSEFHDADAQNSHSYCFEILERRGGGGGMISLIAALAVDRVIGMENAMPWNLPA
template_upload.1.A     FSEFHDADAQNSHSYCFEILERR-------------------------------
```

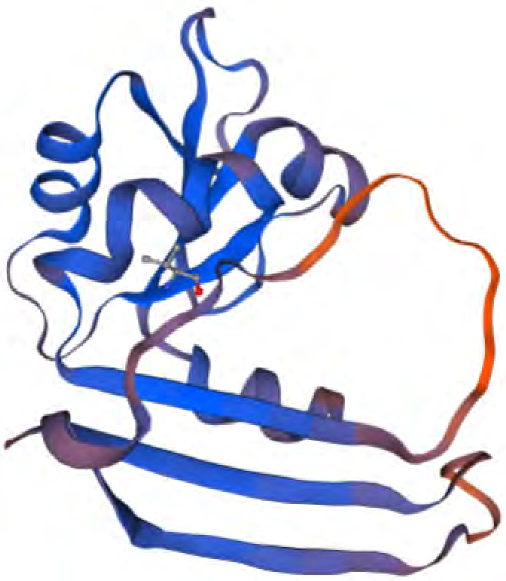

Template: PDB 1rx4A

CP site: Leu28

Target sequence:

```
LAWFKRNTLDKPVIMGRHTWESIGRPLPGRKNIILSSQPGTDDRVTWVKSVDEAIAACGD
VPEIMVIGGGRVYEQFLPKAQKLYLTHIDAEVEGDTHFPDYEPDDWESVFSEFHDADAQN
SHSYCFEILERRGGGGGMISLIAALAVDRVIGMENAMPWNLPAD
```

Model #01	File	Built with	Oligo-State	Ligands	GMQE	QMEAN
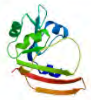	PDB	ProMod3 Version 1.2.0.	monomer	None	0.73	0.54

QMEAN	0.54	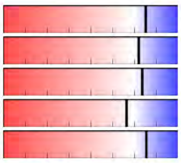
Cβ	0.22	
All Atom	0.38	
Solvation	-0.35	
Torsion	0.55	

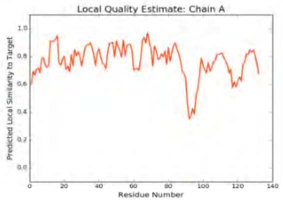

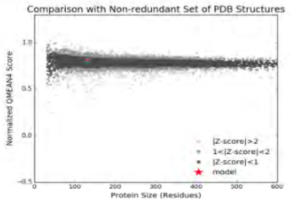

Template	Seq Identity	Oligo-state	Found by	Method	Resolution	Seq Similarity	Range	Coverage	Description
template_upload.1.A	100.00	monomer	HHblits	Unknown	NA	0.63	1 - 132	0.80	Polypeptide

The template contained no ligands.

```
Target                 LAWFKRNTLDKPVIMGRHTWESIGRPLPGRKNIILSSQPGTDDRVTWVKSVDEAI
template_upload.1.A    LAWFKRNTLDKPVIMGRHTWESIGRPLPGRKNIILSSQPGTDDRVTWVKSVDEAI

Target                 AACGDVPEIMVIGGGRVYEQFLPKAQKLYLTHIDAEVEGDTHFPDYEPDDWESVF
template_upload.1.A    AACGDVPEIMVIGGGRVYEQFLPKAQKLYLTHIDAEVEGDTHFPDYEPDDWESVF

Target                 SEFHDADAQNSHSYCFEILERRGGGGGMISLIAALAVDRVIGMENAMPWNLPAD
template_upload.1.A    SEFHDADAQNSHSYCFEILERR--------------------------------
```

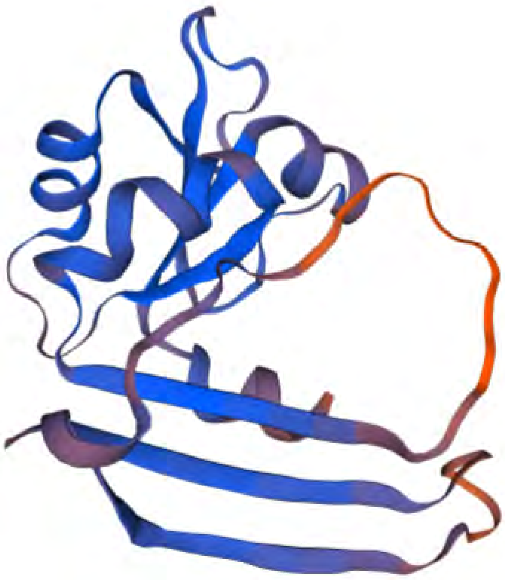

Template: PDB 1rx4A

CP site: Ala29

Target sequence:

```
AWFKRNTLDKPVIMGRHTWESIGRPLPGRKNIILSSQPGTDDRVTWVKSVDEAIAACGDV
PEIMVIGGGRVYEQFLPKAQKLYLTHIDAEVEGDTHFPDYEPDDWESVFSEFHDADAQNS
HSYCFEILERRGGGGGMISLIAALAVDRVIGMENAMPWNLPADL
```

Model #01	File	Built with	Oligo-State	Ligands	GMQE	QMEAN
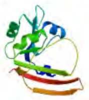	PDB	ProMod3 Version 1.2.0.	monomer	None	0.73	0.60

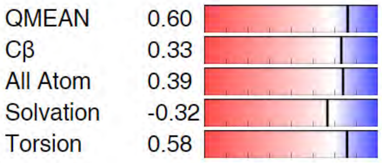

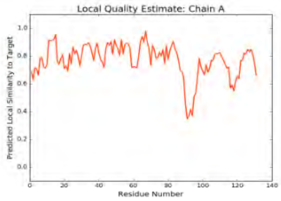

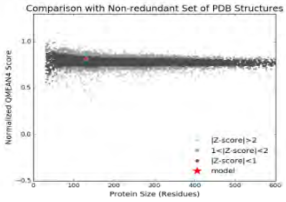

Template	Seq Identity	Oligo-state	Found by	Method	Resolution	Seq Similarity	Range	Coverage	Description
template_upload.1.A	100.00	monomer	HHblits	Unknown	NA	0.63	1 - 131	0.80	Polypeptide

The template contained no ligands.

```
Target                  AWFKRNTLDKPVIMGRHTWESIGRPLPGRKNIILSSQPGTDDRVTWVKSVDEAIA
template_upload.1.A     AWFKRNTLDKPVIMGRHTWESIGRPLPGRKNIILSSQPGTDDRVTWVKSVDEAIA

Target                  ACGDVPEIMVIGGGRVYEQFLPKAQKLYLTHIDAEVEGDTHFPDYEPDDWESVFS
template_upload.1.A     ACGDVPEIMVIGGGRVYEQFLPKAQKLYLTHIDAEVEGDTHFPDYEPDDWESVFS

Target                  EFHDADAQNSHSYCFEILERRGGGGGMISLIAALAVDRVIGMENAMPWNLPADL
template_upload.1.A     EFHDADAQNSHSYCFEILERR---------------------------------
```

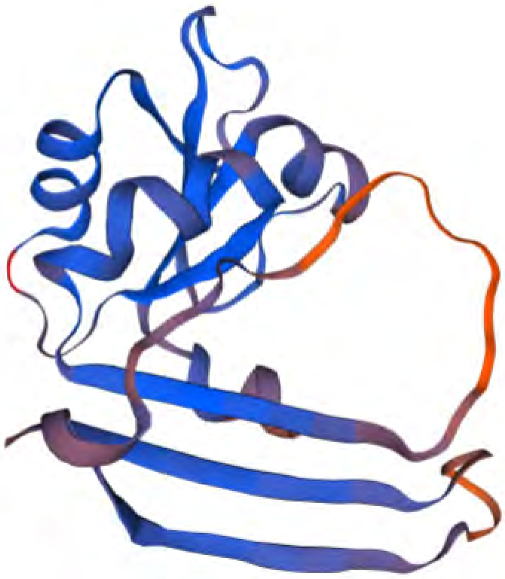

Template: PDB 1rx4A

CP site: Asp37

Target sequence:

DKPVIMGRHTWESIGRPLPGRKNIILSSQPGTDDRVTWVKSVDEAIAACGDVPEIMVIGG
GRVYEQFLPKAQKLYLTHIDAEVEGDTHFPDYEPDDWESVFSEFHDADAQNSHSYCFEIL
ERRGGGGGMISLIAALAVDRVIGMENAMPWNLPADLAWFKRNTL

Model #01	File	Built with	Oligo-State	Ligands	GMQE	QMEAN
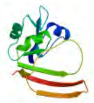	PDB	ProMod3 Version 1.2.0.	monomer	None	0.69	0.69

QMEAN	0.69
Cβ	0.23
All Atom	0.61
Solvation	-0.35
Torsion	0.68

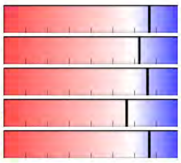

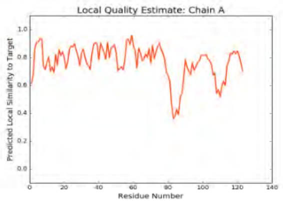

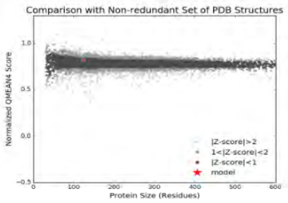

Template	Seq Identity	Oligo-state	Found by	Method	Resolution	Seq Similarity	Range	Coverage	Description
template_upload.1.A	100.00	monomer	HHblits	Unknown	NA	0.63	1 - 123	0.75	Polypeptide

The template contained no ligands.

```
Target                  DKPVIMGRHTWESIGRPLPGRKNIILSSQPGTDDRVTWVKSVDEAIAACGDVPEI
template_upload.1.A     DKPVIMGRHTWESIGRPLPGRKNIILSSQPGTDDRVTWVKSVDEAIAACGDVPEI

Target                  MVIGGGRVYEQFLPKAQKLYLTHIDAEVEGDTHFPDYEPDDWESVFSEFHDADAQ
template_upload.1.A     MVIGGGRVYEQFLPKAQKLYLTHIDAEVEGDTHFPDYEPDDWESVFSEFHDADAQ

Target                  NSHSYCFEILERRGGGGGMISLIAALAVDRVIGMENAMPWNLPADLAWFKRNTL
template_upload.1.A     NSHSYCFEILERR-----------------------------------------
```

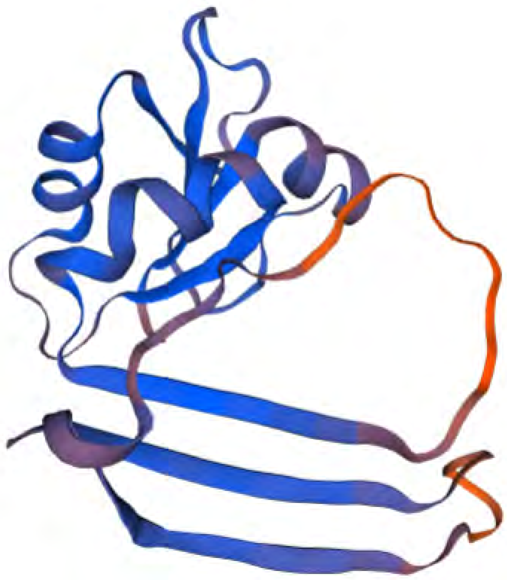

Template: PDB 1rx4A

CP site: Lys38

Target sequence:

```
KPVIMGRHTWESIGRPLPGRKNIILSSQPGTDDRVTWVKSVDEAIAACGDVPEIMVIGGG
RVYEQFLPKAQKLYLTHIDAEVEGDTHFPDYEPDDWESVFSEFHDADAQNSHSYCFEILE
RRGGGGGMISLIAALAVDRVIGMENAMPWNLPADLAWFKRNTLD
```

Model #01	File	Built with	Oligo-State	Ligands	GMQE	QMEAN
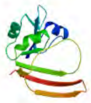	PDB	ProMod3 Version 1.2.0.	monomer	None	0.70	0.64

QMEAN	0.64	
Cβ	0.20	
All Atom	0.59	
Solvation	-0.28	
Torsion	0.62	

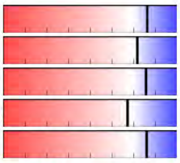

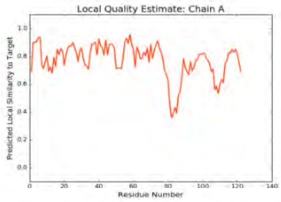

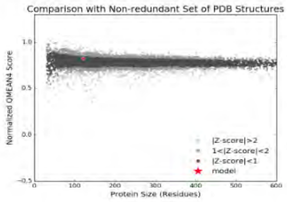

Template	Seq Identity	Oligo-state	Found by	Method	Resolution	Seq Similarity	Range	Coverage	Description
template_upload.1.A	100.00	monomer	HHblits	Unknown	NA	0.63	1 - 122	0.74	Polypeptide

The template contained no ligands.

```
Target                 KPVIMGRHTWESIGRPLPGRKNIILSSQPGTDDRVTWVKSVDEAIAACGDVPEIM
template_upload.1.A    KPVIMGRHTWESIGRPLPGRKNIILSSQPGTDDRVTWVKSVDEAIAACGDVPEIM

Target                 VIGGGRVYEQFLPKAQKLYLTHIDAEVEGDTHFPDYEPDDWESVFSEFHDADAQN
template_upload.1.A    VIGGGRVYEQFLPKAQKLYLTHIDAEVEGDTHFPDYEPDDWESVFSEFHDADAQN

Target                 SHSYCFEILERRGGGGGMISLIAALAVDRVIGMENAMPWNLPADLAWFKRNTLD
template_upload.1.A    SHSYCFEILERR-------------------------------------------
```

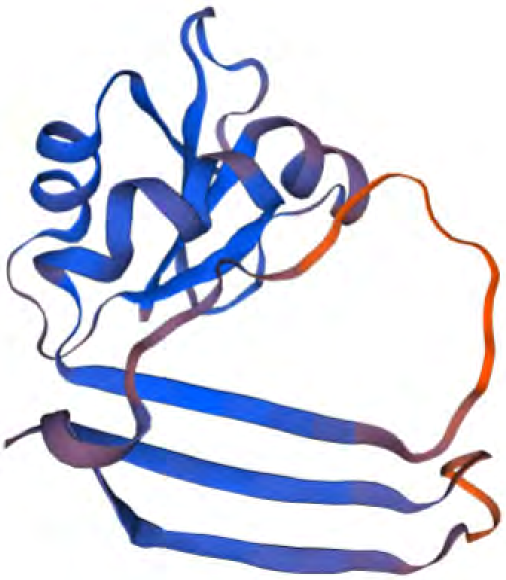

Template: PDB 1rx4A

CP site: Pro39

Target sequence:

PVIMGRHTWESIGRPLPGRKNIILSSQPGTDDRVTWVKSVDEAIAACGDVPEIMVIGGGR
VYEQFLPKAQKLYLTHIDAEVEGDTHFPDYEPDDWESVFSEFHDADAQNSHSYCFEILER
RGGGGGMISLIAALAVDRVIGMENAMPWNLPADLAWFKRNTLDK

Model #01	File	Built with	Oligo-State	Ligands	GMQE	QMEAN
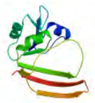	PDB	ProMod3 Version 1.2.0.	monomer	None	0.68	0.71

QMEAN	0.71
Cβ	0.41
All Atom	0.61
Solvation	-0.33
Torsion	0.66

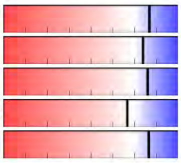

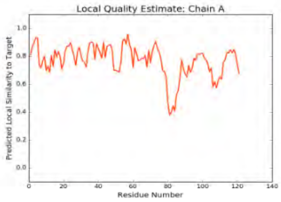

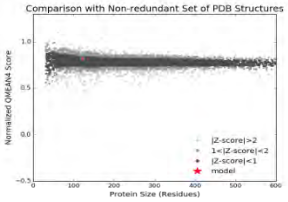

Template	Seq Identity	Oligo-state	Found by	Method	Resolution	Seq Similarity	Range	Coverage	Description
template_upload.1.A	100.00	monomer	HHblits	Unknown	NA	0.63	1 - 121	0.74	Polypeptide

The template contained no ligands.

```
Target                 PVIMGRHTWESIGRPLPGRKNIILSSQPGTDDRVTWVKSVDEAIAACGDVPEIMV
template_upload.1.A    PVIMGRHTWESIGRPLPGRKNIILSSQPGTDDRVTWVKSVDEAIAACGDVPEIMV

Target                 IGGGRVYEQFLPKAQKLYLTHIDAEVEGDTHFPDYEPDDWESVFSEFHDADAQNS
template_upload.1.A    IGGGRVYEQFLPKAQKLYLTHIDAEVEGDTHFPDYEPDDWESVFSEFHDADAQNS

Target                 HSYCFEILERRGGGGGMISLIAALAVDRVIGMENAMPWNLPADLAWFKRNTLDK
template_upload.1.A    HSYCFEILERR-------------------------------------------
```

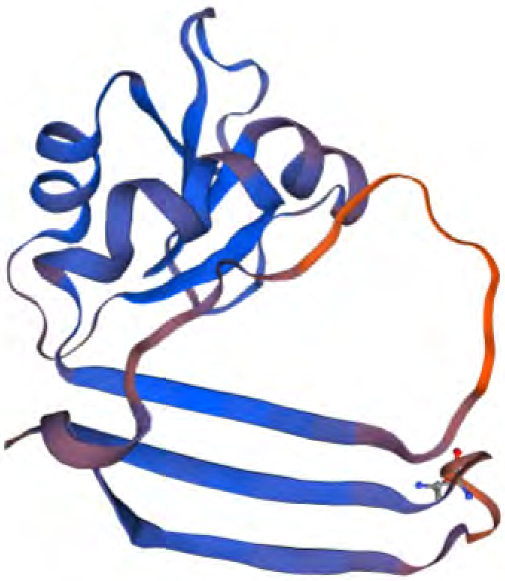

Template: PDB 1rx4A

CP site: Ile50

Target sequence:

```
IGRPLPGRKNIILSSQPGTDDRVTWVKSVDEAIAACGDVPEIMVIGGGRVYEQFLPKAQK
LYLTHIDAEVEGDTHFPDYEPDDWESVFSEFHDADAQNSHSYCFEILERRGGGGGMISLI
AALAVDRVIGMENAMPWNLPADLAWFKRNTLDKPVIMGRHTWES
```

Model #01	File	Built with	Oligo-State	Ligands	GMQE	QMEAN
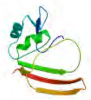	PDB	ProMod3 Version 1.2.0.	monomer	None	0.69	0.57

QMEAN	0.57
Cβ	0.10
All Atom	0.25
Solvation	-0.58
Torsion	0.67

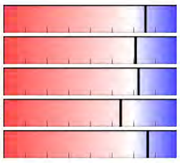

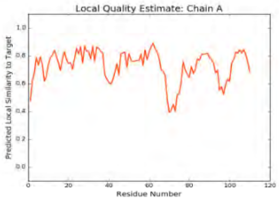

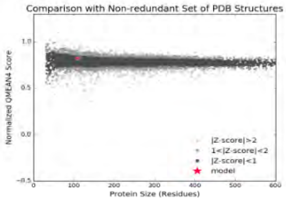

Template	Seq Identity	Oligo-state	Found by	Method	Resolution	Seq Similarity	Range	Coverage	Description
template_upload.1.A	100.00	monomer	BLAST	Unknown	NA	0.62	1 - 110	0.67	Polypeptide

The template contained no ligands.

```
Target                 IGRPLPGRKNIILSSQPGTDDRVTWVKSVDEAIAACGDVPEIMVIGGGRVYEQFL
template_upload.1.A    IGRPLPGRKNIILSSQPGTDDRVTWVKSVDEAIAACGDVPEIMVIGGGRVYEQFL

Target                 PKAQKLYLTHIDAEVEGDTHFPDYEPDDWESVFSEFHDADAQNSHSYCFEILERR
template_upload.1.A    PKAQKLYLTHIDAEVEGDTHFPDYEPDDWESVFSEFHDADAQNSHSYCFEILERR

Target                 GGGGGMISLIAALAVDRVIGMENAMPWNLPADLAWFKRNTLDKPVIMGRHTWES
template_upload.1.A    ------------------------------------------------------
```

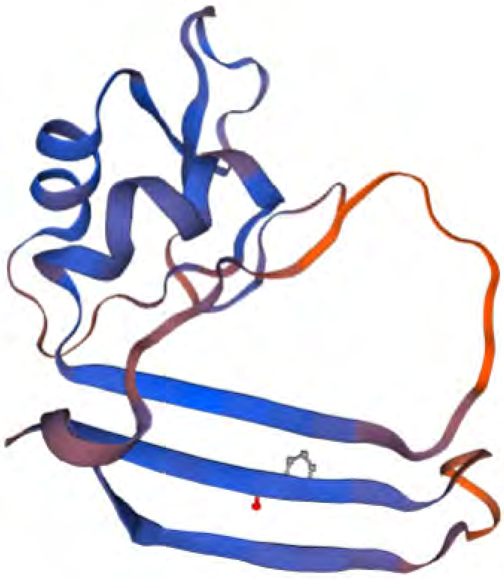

Template: PDB 1rx4A

CP site: Gly51

Target sequence:

GRPLPGRKNIILSSQPGTDDRVTWVKSVDEAIAACGDVPEIMVIGGGRVYEQFLPKAQKL
YLTHIDAEVEGDTHFPDYEPDDWESVFSEFHDADAQNSHSYCFEILERRGGGGGMISLIA
ALAVDRVIGMENAMPWNLPADLAWFKRNTLDKPVIMGRHTWESI

Model #01	File	Built with	Oligo-State	Ligands	GMQE	QMEAN
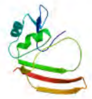	PDB	ProMod3 Version 1.2.0.	monomer	None	0.69	0.45

QMEAN	0.45	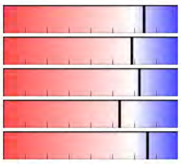
Cβ	-0.10	
All Atom	0.25	
Solvation	-0.67	
Torsion	0.61	

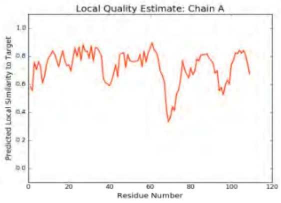

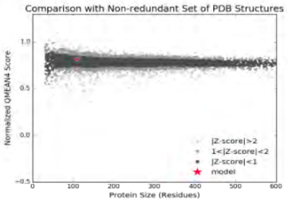

Template	Seq Identity	Oligo-state	Found by	Method	Resolution	Seq Similarity	Range	Coverage	Description
template_upload.1.A	100.00	monomer	BLAST	Unknown	NA	0.62	1 - 109	0.66	Polypeptide

The template contained no ligands.

```
Target                   GRPLPGRKNIILSSQPGTDDRVTWVKSVDEAIAACGDVPEIMVIGGGRVYEQFLP
template_upload.1.A      GRPLPGRKNIILSSQPGTDDRVTWVKSVDEAIAACGDVPEIMVIGGGRVYEQFLP

Target                   KAQKLYLTHIDAEVEGDTHFPDYEPDDWESVFSEFHDADAQNSHSYCFEILERRG
template_upload.1.A      KAQKLYLTHIDAEVEGDTHFPDYEPDDWESVFSEFHDADAQNSHSYCFEILERR-

Target                   GGGGMISLIAALAVDRVIGMENAMPWNLPADLAWFKRNTLDKPVIMGRHTWESI
template_upload.1.A      ------------------------------------------------------
```

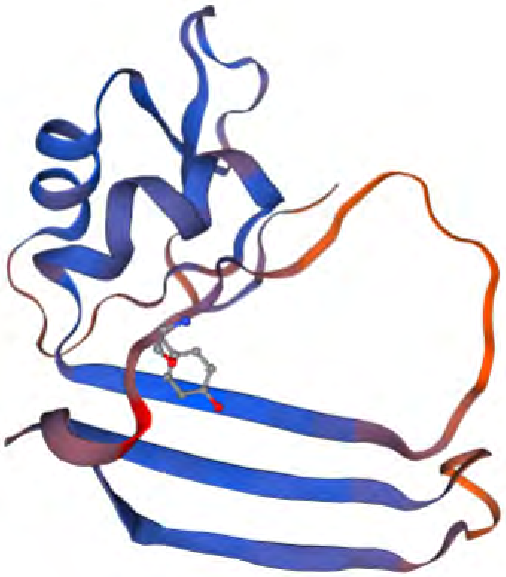

Template: PDB 1rx4A

CP site: Arg52

Target sequence:

RPLPGRKNIILSSQPGTDDRVTWVKSVDEAIAACGDVPEIMVIGGGRVYEQFLPKAQKLY
LTHIDAEVEGDTHFPDYEPDDWESVFSEFHDADAQNSHSYCFEILERRGGGGGMISLIAA
LAVDRVIGMENAMPWNLPADLAWFKRNTLDKPVIMGRHTWESIG

Model #01	File	Built with	Oligo-State	Ligands	GMQE	QMEAN
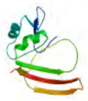	PDB	ProMod3 Version 1.2.0.	monomer	None	0.62	0.46

QMEAN	0.46	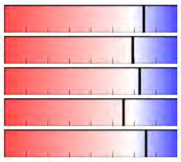
Cβ	-0.06	
All Atom	0.27	
Solvation	-0.48	
Torsion	0.57	

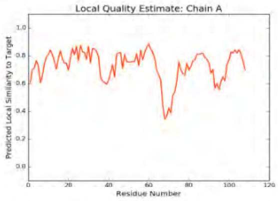

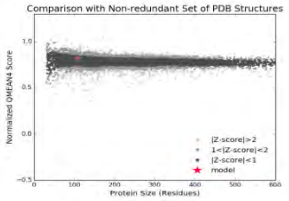

Template	Seq Identity	Oligo-state	Found by	Method	Resolution	Seq Similarity	Range	Coverage	Description
template_upload.1.A	100.00	monomer	HHblits	Unknown	NA	0.62	1 - 108	0.66	Polypeptide

The template contained no ligands.

```
Target                RPLPGRKNIILSSQPGTDDRVTWVKSVDEAIAACGDVPEIMVIGGGRVYEQFLPK
template_upload.1.A   RPLPGRKNIILSSQPGTDDRVTWVKSVDEAIAACGDVPEIMVIGGGRVYEQFLPK

Target                AQKLYLTHIDAEVEGDTHFPDYEPDDWESVFSEFHDADAQNSHSYCFEILERRGG
template_upload.1.A   AQKLYLTHIDAEVEGDTHFPDYEPDDWESVFSEFHDADAQNSHSYCFEILERR--

Target                GGGMISLIAALAVDRVIGMENAMPWNLPADLAWFKRNTLDKPVIMGRHTWESIG
template_upload.1.A   ------------------------------------------------------
```

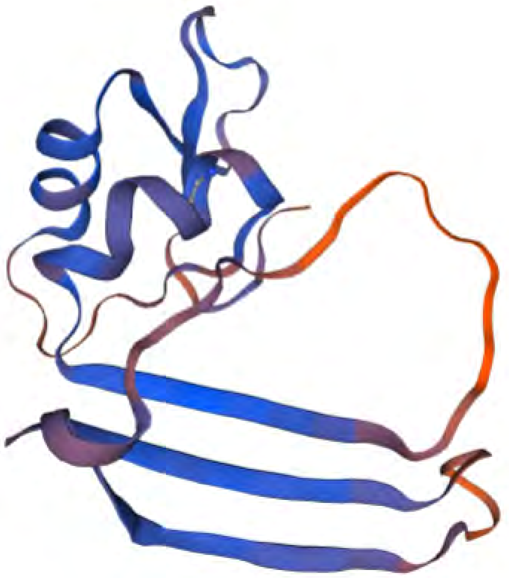

Template: PDB 1rx4A

CP site: Pro53

Target sequence:

```
PLPGRKNIILSSQPGTDDRVTWVKSVDEAIAACGDVPEIMVIGGGRVYEQFLPKAQKLYL
THIDAEVEGDTHFPDYEPDDWESVFSEFHDADAQNSHSYCFEILERRGGGGGMISLIAAL
AVDRVIGMENAMPWNLPADLAWFKRNTLDKPVIMGRHTWESIGR
```

Model #01	File	Built with	Oligo-State	Ligands	GMQE	QMEAN
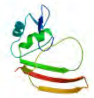	PDB	ProMod3 Version 1.2.0.	monomer	None	0.63	0.41

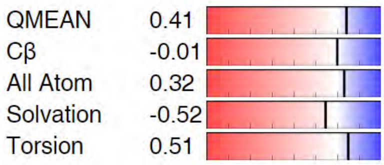

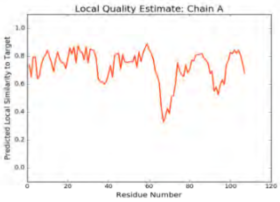

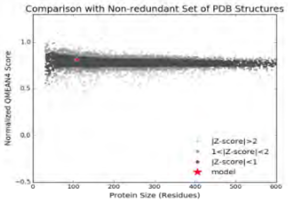

Template	Seq Identity	Oligo-state	Found by	Method	Resolution	Seq Similarity	Range	Coverage	Description
template_upload.1.A	100.00	monomer	HHblits	Unknown	NA	0.62	1 - 107	0.65	Polypeptide

The template contained no ligands.

```
Target                  PLPGRKNIILSSQPGTDDRVTWVKSVDEAIAACGDVPEIMVIGGGRVYEQFLPKA
template_upload.1.A     PLPGRKNIILSSQPGTDDRVTWVKSVDEAIAACGDVPEIMVIGGGRVYEQFLPKA

Target                  QKLYLTHIDAEVEGDTHFPDYEPDDWESVFSEFHDADAQNSHSYCFEILERRGGG
template_upload.1.A     QKLYLTHIDAEVEGDTHFPDYEPDDWESVFSEFHDADAQNSHSYCFEILERR---

Target                  GGMISLIAALAVDRVIGMENAMPWNLPADLAWFKRNTLDKPVIMGRHTWESIGR
template_upload.1.A     ------------------------------------------------------
```

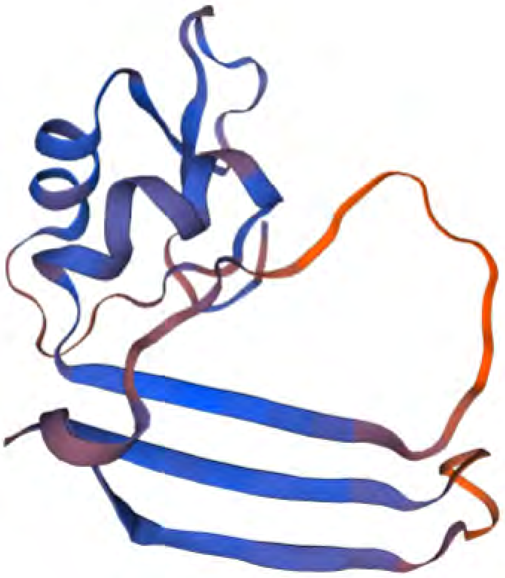

Template: PDB 1rx4A

CP site: Leu54

Target sequence:

```
LPGRKNIILSSQPGTDDRVTWVKSVDEAIAACGDVPEIMVIGGGRVYEQFLPKAQKLYLT
HIDAEVEGDTHFPDYEPDDWESVFSEFHDADAQNSHSYCFEILERRGGGGGMISLIAALA
VDRVIGMENAMPWNLPADLAWFKRNTLDKPVIMGRHTWESIGRP
```

Model #01	File	Built with	Oligo-State	Ligands	GMQE	QMEAN
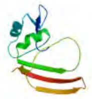	PDB	ProMod3 Version 1.2.0.	monomer	None	0.62	0.29

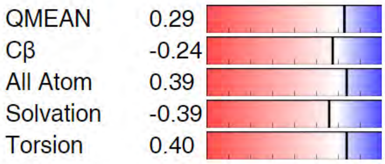

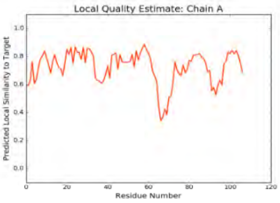

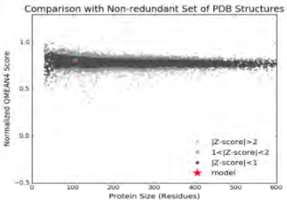

Template	Seq Identity	Oligo-state	Found by	Method	Resolution	Seq Similarity	Range	Coverage	Description
template_upload.1.A	100.00	monomer	HHblits	Unknown	NA	0.62	1 - 106	0.65	Polypeptide

The template contained no ligands.

```
Target                LPGRKNIILSSQPGTDDRVTWVKSVDEAIAACGDVPEIMVIGGGRVYEQFLPKAQ
template_upload.1.A   LPGRKNIILSSQPGTDDRVTWVKSVDEAIAACGDVPEIMVIGGGRVYEQFLPKAQ

Target                KLYLTHIDAEVEGDTHFPDYEPDDWESVFSEFHDADAQNSHSYCFEILERRGGGG
template_upload.1.A   KLYLTHIDAEVEGDTHFPDYEPDDWESVFSEFHDADAQNSHSYCFEILERR----

Target                GMISLIAALAVDRVIGMENAMPWNLPADLAWFKRNTLDKPVIMGRHTWESIGRP
template_upload.1.A   ------------------------------------------------------
```

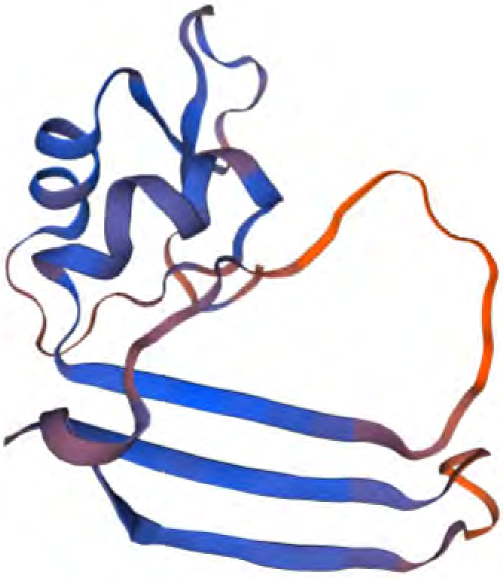

Template: PDB 1rx4A

CP site: Pro55

Target sequence:

PGRKNIILSSQPGTDDRVTWVKSVDEAIAACGDVPEIMVIGGGRVYEQFLPKAQKLYLTH
IDAEVEGDTHFPDYEPDDWESVFSEFHDADAQNSHSYCFEILERRGGGGGMISLIAALAV
DRVIGMENAMPWNLPADLAWFKRNTLDKPVIMGRHTWESIGRPL

Model #01	File	Built with	Oligo-State	Ligands	GMQE	QMEAN
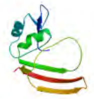	PDB	ProMod3 Version 1.2.0.	monomer	None	0.61	0.11

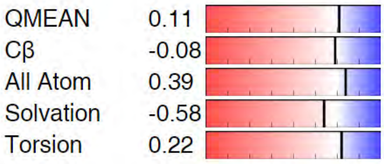

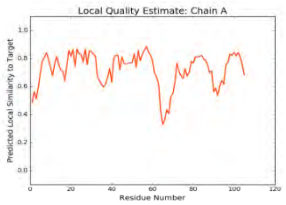

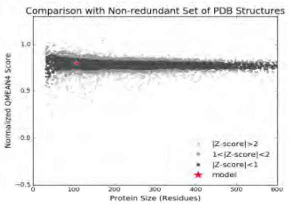

Template	Seq Identity	Oligo-state	Found by	Method	Resolution	Seq Similarity	Range	Coverage	Description
template_upload.1.A	100.00	monomer	HHblits	Unknown	NA	0.62	1 - 105	0.64	Polypeptide

The template contained no ligands.

```
Target                  PGRKNIILSSQPGTDDRVTWVKSVDEAIAACGDVPEIMVIGGGRVYEQFLPKAQK
template_upload.1.A     PGRKNIILSSQPGTDDRVTWVKSVDEAIAACGDVPEIMVIGGGRVYEQFLPKAQK

Target                  LYLTHIDAEVEGDTHFPDYEPDDWESVFSEFHDADAQNSHSYCFEILERRGGGGG
template_upload.1.A     LYLTHIDAEVEGDTHFPDYEPDDWESVFSEFHDADAQNSHSYCFEILERR-----

Target                  MISLIAALAVDRVIGMENAMPWNLPADLAWFKRNTLDKPVIMGRHTWESIGRPL
template_upload.1.A     ------------------------------------------------------
```

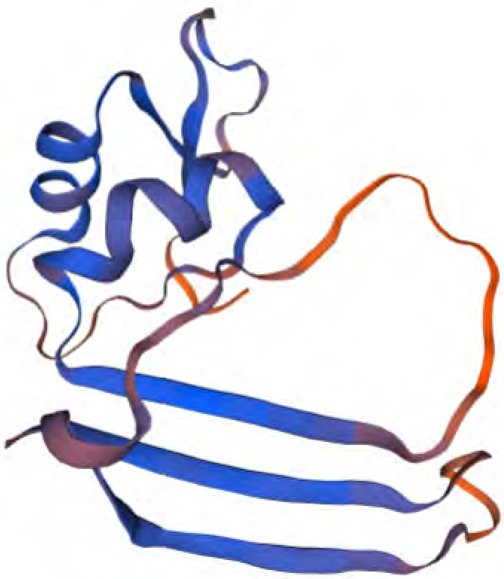

Template: PDB 1rx4A

CP site: Gly56

Target sequence:

```
GRKNIILSSQPGTDDRVTWVKSVDEAIAACGDVPEIMVIGGGRVYEQFLPKAQKLYLTHI
DAEVEGDTHFPDYEPDDWESVFSEFHDADAQNSHSYCFEILERRGGGGGMISLIAALAVD
RVIGMENAMPWNLPADLAWFKRNTLDKPVIMGRHTWESIGRPLP
```

Model #01	File	Built with	Oligo-State	Ligands	GMQE	QMEAN
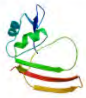	PDB	ProMod3 Version 1.2.0.	monomer	None	0.60	0.11

QMEAN	0.11
Cβ	-0.15
All Atom	0.38
Solvation	-0.49
Torsion	0.21

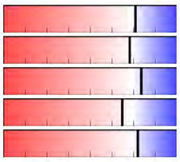

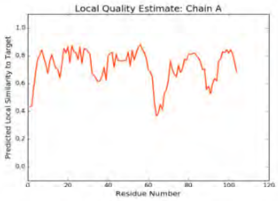

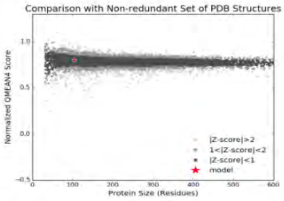

Template	Seq Identity	Oligo-state	Found by	Method	Resolution	Seq Similarity	Range	Coverage	Description
template_upload.1.A	100.00	monomer	HHblits	Unknown	NA	0.62	1 - 104	0.63	Polypeptide

The template contained no ligands.

```
Target               GRKNIILSSQPGTDDRVTWVKSVDEAIAACGDVPEIMVIGGGRVYEQFLPKAQKL
template_upload.1.A  GRKNIILSSQPGTDDRVTWVKSVDEAIAACGDVPEIMVIGGGRVYEQFLPKAQKL

Target               YLTHIDAEVEGDTHFPDYEPDDWESVFSEFHDADAQNSHSYCFEILERRGGGGGM
template_upload.1.A  YLTHIDAEVEGDTHFPDYEPDDWESVFSEFHDADAQNSHSYCFEILERR------

Target               ISLIAALAVDRVIGMENAMPWNLPADLAWFKRNTLDKPVIMGRHTWESIGRPLP
template_upload.1.A  ------------------------------------------------------
```

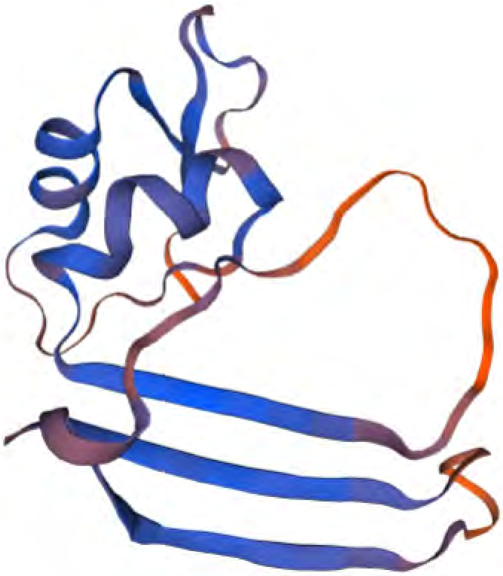

Template: PDB 1rx4A

CP site: Ser64

Target sequence:

SQPGTDDRVTWVKSVDEAIAACGDVPEIMVIGGGRVYEQFLPKAQKLYLTHIDAEVEGDT
HFPDYEPDDWESVFSEFHDADAQNSHSYCFEILERRGGGGGMISLIAALAVDRVIGMENA
MPWNLPADLAWFKRNTLDKPVIMGRHTWESIGRPLPGRKNIILS

Model #01	File	Built with	Oligo-State	Ligands	GMQE	QMEAN
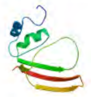	PDB	ProMod3 Version 1.2.0.	monomer	None	0.65	0.06

QMEAN	0.06
Cβ	0.05
All Atom	0.11
Solvation	-0.72
Torsion	0.18

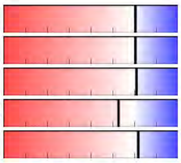

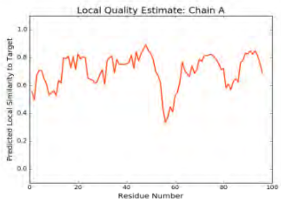

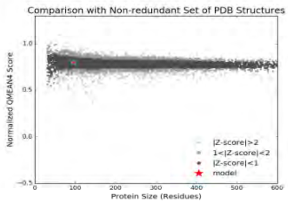

Template	Seq Identity	Oligo-state	Found by	Method	Resolution	Seq Similarity	Range	Coverage	Description
template_upload.1.A	100.00	monomer	BLAST	Unknown	NA	0.63	1 - 96	0.59	Polypeptide

The template contained no ligands.

```
Target                SQPGTDDRVTWVKSVDEAIAACGDVPEIMVIGGGRVYEQFLPKAQKLYLTHIDAE
template_upload.1.A   SQPGTDDRVTWVKSVDEAIAACGDVPEIMVIGGGRVYEQFLPKAQKLYLTHIDAE

Target                VEGDTHFPDYEPDDWESVFSEFHDADAQNSHSYCFEILERRGGGGGMISLIAALA
template_upload.1.A   VEGDTHFPDYEPDDWESVFSEFHDADAQNSHSYCFEILERR--------------

Target                VDRVIGMENAMPWNLPADLAWFKRNTLDKPVIMGRHTWESIGRPLPGRKNIILS
template_upload.1.A   ------------------------------------------------------
```

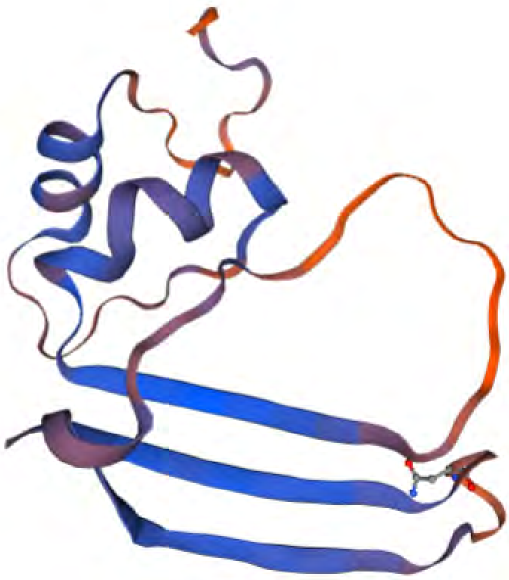

Template: PDB 1rx4A

CP site: Gln65

Target sequence:

```
QPGTDDRVTWVKSVDEAIAACGDVPEIMVIGGGRVYEQFLPKAQKLYLTHIDAEVEGDTH
FPDYEPDDWESVFSEFHDADAQNSHSYCFEILERRGGGGGMISLIAALAVDRVIGMENAM
PWNLPADLAWFKRNTLDKPVIMGRHTWESIGRPLPGRKNIILSS
```

Model #01	File	Built with	Oligo-State	Ligands	GMQE	QMEAN
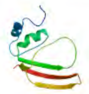	PDB	ProMod3 Version 1.2.0.	monomer	None	0.64	0.01

QMEAN	0.01	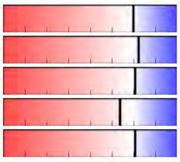
Cβ	0.23	
All Atom	0.08	
Solvation	-0.61	
Torsion	0.07	

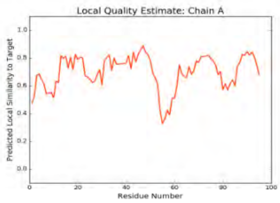

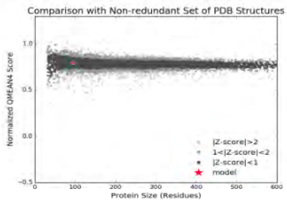

Template	Seq Identity	Oligo-state	Found by	Method	Resolution	Seq Similarity	Range	Coverage	Description
template_upload.1.A	100.00	monomer	BLAST	Unknown	NA	0.63	1 - 95	0.58	Polypeptide

The template contained no ligands.

```
Target               QPGTDDRVTWVKSVDEAIAACGDVPEIMVIGGGRVYEQFLPKAQKLYLTHIDAEV
template_upload.1.A  QPGTDDRVTWVKSVDEAIAACGDVPEIMVIGGGRVYEQFLPKAQKLYLTHIDAEV

Target               EGDTHFPDYEPDDWESVFSEFHDADAQNSHSYCFEILERRGGGGGMISLIAALAV
template_upload.1.A  EGDTHFPDYEPDDWESVFSEFHDADAQNSHSYCFEILERR---------------

Target               DRVIGMENAMPWNLPADLAWFKRNTLDKPVIMGRHTWESIGRPLPGRKNIILSS
template_upload.1.A  ------------------------------------------------------
```

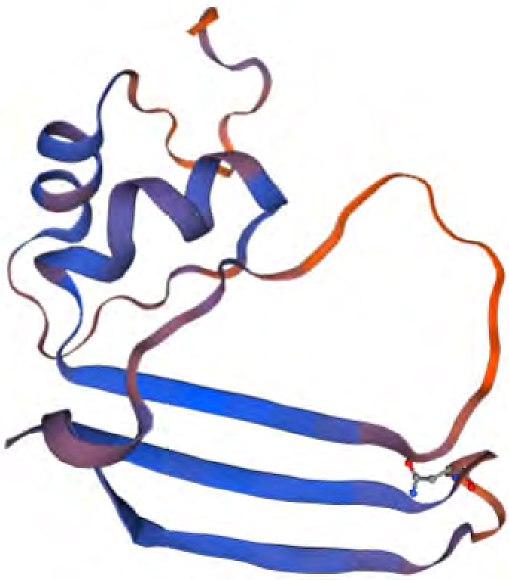

Template: PDB 1rx4A

CP site: Pro66

Target sequence:

PGTDDRVTWVKSVDEAIAACGDVPEIMVIGGGRVYEQFLPKAQKLYLTHIDAEVEGDTHF
PDYEPDDWESVFSEFHDADAQNSHSYCFEILERRGGGGGMISLIAALAVDRVIGMENAMP
WNLPADLAWFKRNTLDKPVIMGRHTWESIGRPLPGRKNIILSSQ

Model #01	File	Built with	Oligo-State	Ligands	GMQE	QMEAN
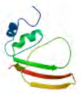	PDB	ProMod3 Version 1.2.0.	monomer	None	0.63	-0.04

QMEAN	-0.04	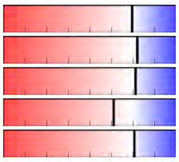
Cβ	0.21	
All Atom	0.13	
Solvation	-0.89	
Torsion	0.07	

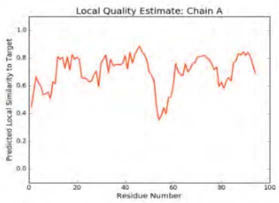

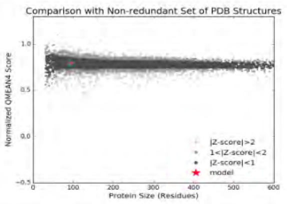

Template	Seq Identity	Oligo-state	Found by	Method	Resolution	Seq Similarity	Range	Coverage	Description
template_upload.1.A	100.00	monomer	BLAST	Unknown	NA	0.63	1 - 94	0.57	Polypeptide

The template contained no ligands.

```
Target                PGTDDRVTWVKSVDEAIAACGDVPEIMVIGGGRVYEQFLPKAQKLYLTHIDAEVE
template_upload.1.A   PGTDDRVTWVKSVDEAIAACGDVPEIMVIGGGRVYEQFLPKAQKLYLTHIDAEVE

Target                GDTHFPDYEPDDWESVFSEFHDADAQNSHSYCFEILERRGGGGGMISLIAALAVD
template_upload.1.A   GDTHFPDYEPDDWESVFSEFHDADAQNSHSYCFEILERR----------------

Target                RVIGMENAMPWNLPADLAWFKRNTLDKPVIMGRHTWESIGRPLPGRKNIILSSQ
template_upload.1.A   ------------------------------------------------------
```

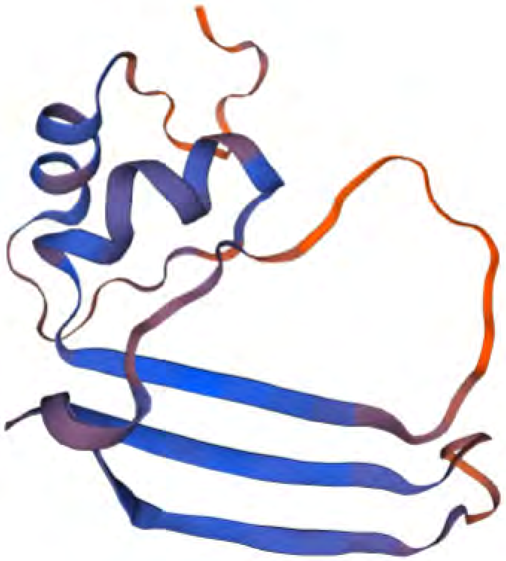

Template: PDB 1rx4A

CP site: Gly67

Target sequence:

GTDDRVTWVKSVDEAIAACGDVPEIMVIGGGRVYEQFLPKAQKLYLTHIDAEVEGDTHFP
DYEPDDWESVFSEFHDADAQNSHSYCFEILERRGGGGGMISLIAALAVDRVIGMENAMPW
NLPADLAWFKRNTLDKPVIMGRHTWESIGRPLPGRKNIILSSQP

Model #01	File	Built with	Oligo-State	Ligands	GMQE	QMEAN
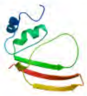	PDB	ProMod3 Version 1.2.0.	monomer	None	0.62	-0.08

QMEAN	-0.08	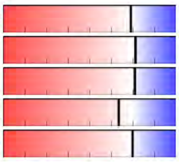
Cβ	0.14	
All Atom	0.11	
Solvation	-0.64	
Torsion	-0.00	

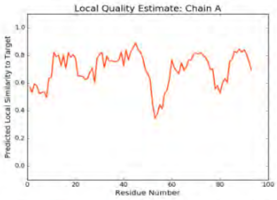

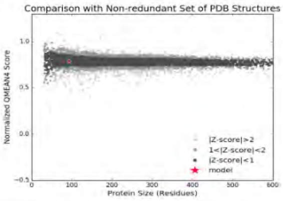

Template	Seq Identity	Oligo-state	Found by	Method	Resolution	Seq Similarity	Range	Coverage	Description
template_upload.1.A	100.00	monomer	BLAST	Unknown	NA	0.63	1 - 93	0.57	Polypeptide

The template contained no ligands.

```
Target                  GTDDRVTWVKSVDEAIAACGDVPEIMVIGGGRVYEQFLPKAQKLYLTHIDAEVEG
template_upload.1.A     GTDDRVTWVKSVDEAIAACGDVPEIMVIGGGRVYEQFLPKAQKLYLTHIDAEVEG

Target                  DTHFPDYEPDDWESVFSEFHDADAQNSHSYCFEILERRGGGGGMISLIAALAVDR
template_upload.1.A     DTHFPDYEPDDWESVFSEFHDADAQNSHSYCFEILERR-----------------

Target                  VIGMENAMPWNLPADLAWFKRNTLDKPVIMGRHTWESIGRPLPGRKNIILSSQP
template_upload.1.A     ------------------------------------------------------
```

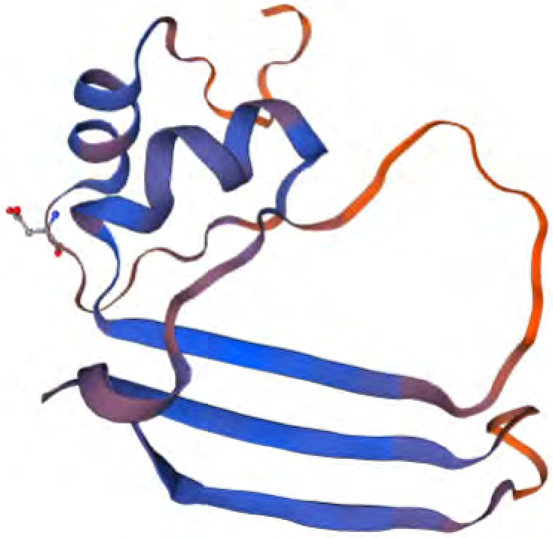

Template: PDB 1rx4A

CP site: Thr68

Target sequence:

TDDRVTWVKSVDEAIAACGDVPEIMVIGGGRVYEQFLPKAQKLYLTHIDAEVEGDTHFPD
YEPDDWESVFSEFHDADAQNSHSYCFEILERRGGGGGMISLIAALAVDRVIGMENAMPWN
LPADLAWFKRNTLDKPVIMGRHTWESIGRPLPGRKNIILSSQPG

Model #01	File	Built with	Oligo-State	Ligands	GMQE	QMEAN
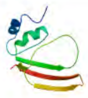	PDB	ProMod3 Version 1.2.0.	monomer	None	0.61	-0.23

QMEAN	-0.23	
Cβ	-0.24	
All Atom	0.21	
Solvation	-0.66	
Torsion	-0.07	

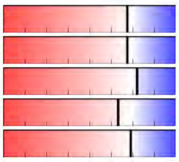

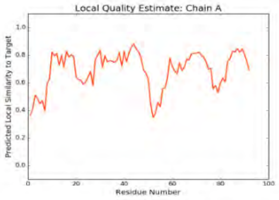

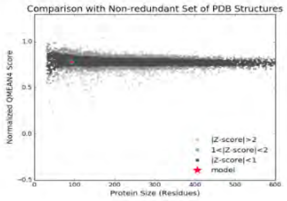

Template	Seq Identity	Oligo-state	Found by	Method	Resolution	Seq Similarity	Range	Coverage	Description
template_upload.1.A	100.00	monomer	BLAST	Unknown	NA	0.63	1 - 92	0.56	Polypeptide

The template contained no ligands.

```
Target                TDDRVTWVKSVDEAIAACGDVPEIMVIGGGRVYEQFLPKAQKLYLTHIDAEVEGD
template_upload.1.A   TDDRVTWVKSVDEAIAACGDVPEIMVIGGGRVYEQFLPKAQKLYLTHIDAEVEGD

Target                THFPDYEPDDWESVFSEFHDADAQNSHSYCFEILERRGGGGGMISLIAALAVDRV
template_upload.1.A   THFPDYEPDDWESVFSEFHDADAQNSHSYCFEILERR------------------

Target                IGMENAMPWNLPADLAWFKRNTLDKPVIMGRHTWESIGRPLPGRKNIILSSQPG
template_upload.1.A   ------------------------------------------------------
```

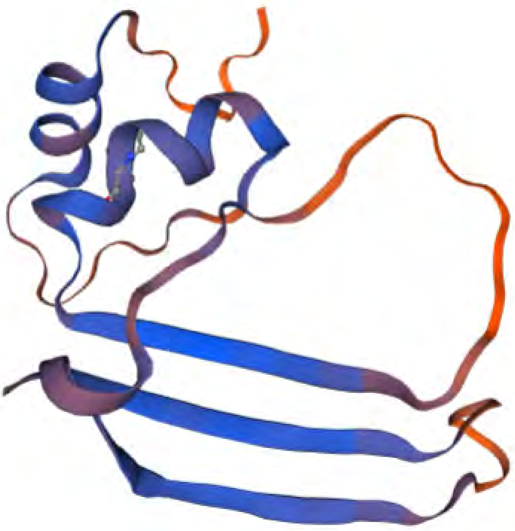

Template: PDB 1rx4A

CP site: Asp69

Target sequence:

DDRVTWVKSVDEAIAACGDVPEIMVIGGGRVYEQFLPKAQKLYLTHIDAEVEGDTHFPDY
EPDDWESVFSEFHDADAQNSHSYCFEILERRGGGGGMISLIAALAVDRVIGMENAMPWNL
PADLAWFKRNTLDKPVIMGRHTWESIGRPLPGRKNIILSSQPGT

Model #01	File	Built with	Oligo-State	Ligands	GMQE	QMEAN
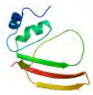	PDB	ProMod3 Version 1.2.0.	monomer	None	0.60	-0.21

QMEAN	-0.21	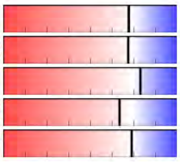
Cβ	-0.23	
All Atom	0.32	
Solvation	-0.65	
Torsion	-0.07	

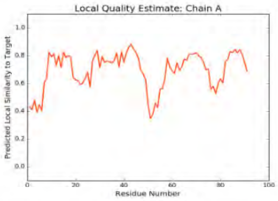

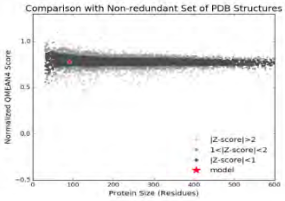

Template	Seq Identity	Oligo-state	Found by	Method	Resolution	Seq Similarity	Range	Coverage	Description
template_upload.1.A	100.00	monomer	BLAST	Unknown	NA	0.63	1 - 91	0.55	Polypeptide

The template contained no ligands.

```
Target                    DDRVTWVKSVDEAIAACGDVPEIMVIGGGRVYEQFLPKAQKLYLTHIDAEVEGDT
template_upload.1.A       DDRVTWVKSVDEAIAACGDVPEIMVIGGGRVYEQFLPKAQKLYLTHIDAEVEGDT

Target                    HFPDYEPDDWESVFSEFHDADAQNSHSYCFEILERRGGGGGMISLIAALAVDRVI
template_upload.1.A       HFPDYEPDDWESVFSEFHDADAQNSHSYCFEILERR-------------------

Target                    GMENAMPWNLPADLAWFKRNTLDKPVIMGRHTWESIGRPLPGRKNIILSSQPGT
template_upload.1.A       ------------------------------------------------------
```

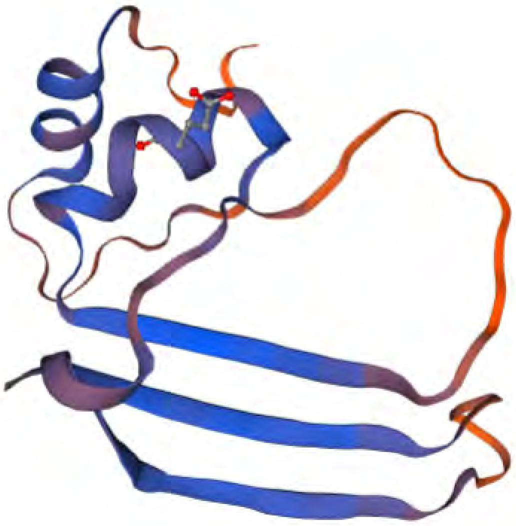

Template: PDB 1rx4A

CP site: Asp70

Target sequence:

```
DRVTWVKSVDEAIAACGDVPEIMVIGGGRVYEQFLPKAQKLYLTHIDAEVEGDTHFPDYE
PDDWESVFSEFHDADAQNSHSYCFEILERRGGGGGMISLIAALAVDRVIGMENAMPWNLP
ADLAWFKRNTLDKPVIMGRHTWESIGRPLPGRKNIILSSQPGTD
```

Model #01	File	Built with	Oligo-State	Ligands	GMQE	QMEAN
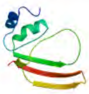	PDB	ProMod3 Version 1.2.0.	monomer	None	0.46	-0.21

QMEAN	-0.21	
Cβ	-0.40	
All Atom	0.31	
Solvation	-0.61	
Torsion	-0.03	

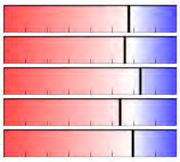

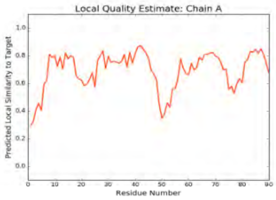

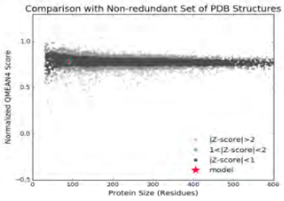

Template	Seq Identity	Oligo-state	Found by	Method	Resolution	Seq Similarity	Range	Coverage	Description
template_upload.1.A	100.00	monomer	HHblits	Unknown	NA	0.63	1 - 90	0.55	Polypeptide

The template contained no ligands.

```
Target                    DRVTWVKSVDEAIAACGDVPEIMVIGGGRVYEQFLPKAQKLYLTHIDAEVEGDTH
template_upload.1.A       DRVTWVKSVDEAIAACGDVPEIMVIGGGRVYEQFLPKAQKLYLTHIDAEVEGDTH

Target                    FPDYEPDDWESVFSEFHDADAQNSHSYCFEILERRGGGGGMISLIAALAVDRVIG
template_upload.1.A       FPDYEPDDWESVFSEFHDADAQNSHSYCFEILERR--------------------

Target                    MENAMPWNLPADLAWFKRNTLDKPVIMGRHTWESIGRPLPGRKNIILSSQPGTD
template_upload.1.A       ------------------------------------------------------
```

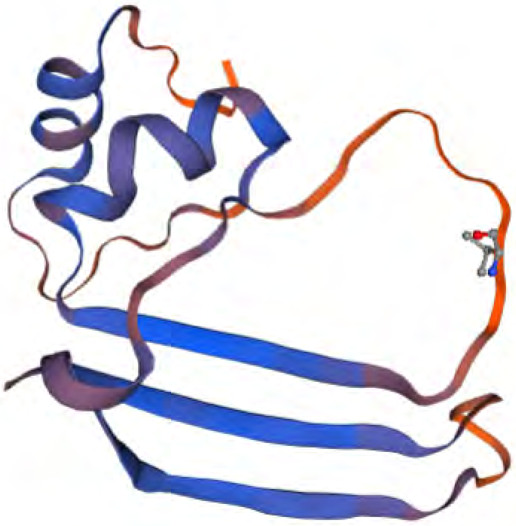

Template: PDB 1rx4A

CP site: Arg71

Target sequence:

RVTWVKSVDEAIAACGDVPEIMVIGGGRVYEQFLPKAQKLYLTHIDAEVEGDTHFPDYEP
DDWESVFSEFHDADAQNSHSYCFEILERRGGGGGMISLIAALAVDRVIGMENAMPWNLPA
DLAWFKRNTLDKPVIMGRHTWESIGRPLPGRKNIILSSQPGTDD

Model #01	File	Built with	Oligo-State	Ligands	GMQE	QMEAN
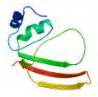	PDB	ProMod3 Version 1.2.0.	monomer	None	0.57	-0.26

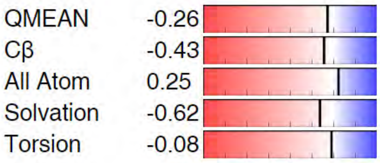

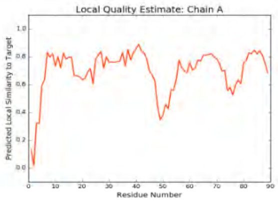

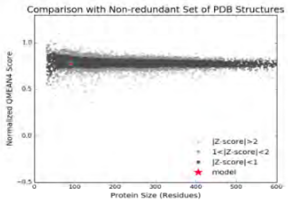

Template	Seq Identity	Oligo-state	Found by	Method	Resolution	Seq Similarity	Range	Coverage	Description
template_upload.1.A	100.00	monomer	BLAST	Unknown	NA	0.63	1 - 89	0.54	Polypeptide

The template contained no ligands.

```
Target                RVTWVKSVDEAIAACGDVPEIMVIGGGRVYEQFLPKAQKLYLTHIDAEVEGDTHF
template_upload.1.A   RVTWVKSVDEAIAACGDVPEIMVIGGGRVYEQFLPKAQKLYLTHIDAEVEGDTHF

Target                PDYEPDDWESVFSEFHDADAQNSHSYCFEILERRGGGGGMISLIAALAVDRVIGM
template_upload.1.A   PDYEPDDWESVFSEFHDADAQNSHSYCFEILERR---------------------

Target                ENAMPWNLPADLAWFKRNTLDKPVIMGRHTWESIGRPLPGRKNIILSSQPGTDD
template_upload.1.A   ------------------------------------------------------
```

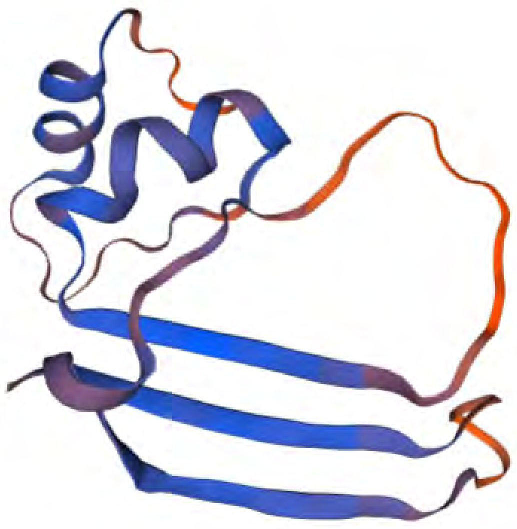

Template: PDB 1rx4A

CP site: Val72

Target sequence:

VTWVKSVDEAIAACGDVPEIMVIGGGRVYEQFLPKAQKLYLTHIDAEVEGDTHFPDYEPD
DWESVFSEFHDADAQNSHSYCFEILERRGGGGGMISLIAALAVDRVIGMENAMPWNLPAD
LAWFKRNTLDKPVIMGRHTWESIGRPLPGRKNIILSSQPGTDDR

Model #01	File	Built with	Oligo-State	Ligands	GMQE	QMEAN
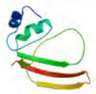	PDB	ProMod3 Version 1.2.0.	monomer	None	0.56	-0.31

QMEAN	-0.31
Cβ	-0.42
All Atom	0.14
Solvation	-0.65
Torsion	-0.10

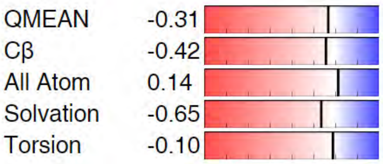

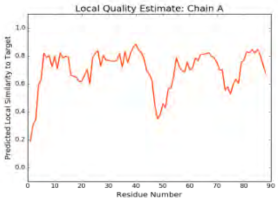

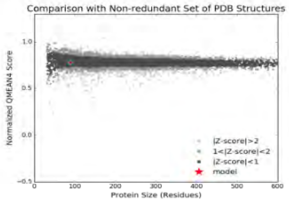

Template	Seq Identity	Oligo-state	Found by	Method	Resolution	Seq Similarity	Range	Coverage	Description
template_upload.1.A	100.00	monomer	BLAST	Unknown	NA	0.63	1 - 88	0.54	Polypeptide

The template contained no ligands.

```
Target               VTWVKSVDEAIAACGDVPEIMVIGGGRVYEQFLPKAQKLYLTHIDAEVEGDTHFP
template_upload.1.A  VTWVKSVDEAIAACGDVPEIMVIGGGRVYEQFLPKAQKLYLTHIDAEVEGDTHFP

Target               DYEPDDWESVFSEFHDADAQNSHSYCFEILERRGGGGGMISLIAALAVDRVIGME
template_upload.1.A  DYEPDDWESVFSEFHDADAQNSHSYCFEILERR----------------------

Target               NAMPWNLPADLAWFKRNTLDKPVIMGRHTWESIGRPLPGRKNIILSSQPGTDDR
template_upload.1.A  ------------------------------------------------------
```

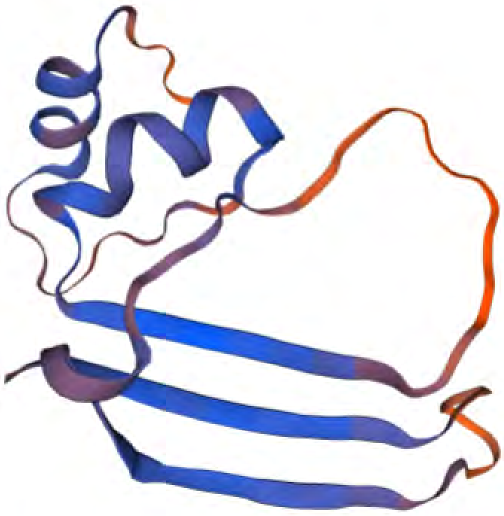

Template: PDB 1rx4A

CP site: Thr73

Target sequence:

```
TWVKSVDEAIAACGDVPEIMVIGGGRVYEQFLPKAQKLYLTHIDAEVEGDTHFPDYEPDD
WESVFSEFHDADAQNSHSYCFEILERRGGGGGMISLIAALAVDRVIGMENAMPWNLPADL
AWFKRNTLDKPVIMGRHTWESIGRPLPGRKNIILSSQPGTDDRV
```

Model #01	File	Built with	Oligo-State	Ligands	GMQE	QMEAN
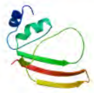	PDB	ProMod3 Version 1.2.0.	monomer	None	0.57	-0.25

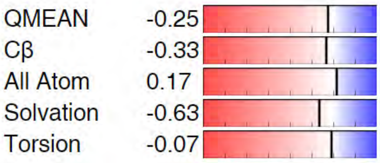

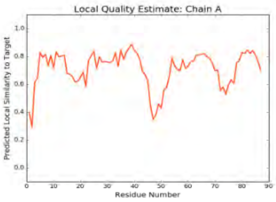

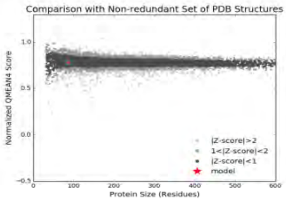

Template	Seq Identity	Oligo-state	Found by	Method	Resolution	Seq Similarity	Range	Coverage	Description
template_upload.1.A	100.00	monomer	BLAST	Unknown	NA	0.63	1 - 87	0.53	Polypeptide

The template contained no ligands.

```
Target               TWVKSVDEAIAACGDVPEIMVIGGGRVYEQFLPKAQKLYLTHIDAEVEGDTHFPD
template_upload.1.A  TWVKSVDEAIAACGDVPEIMVIGGGRVYEQFLPKAQKLYLTHIDAEVEGDTHFPD

Target               YEPDDWESVFSEFHDADAQNSHSYCFEILERRGGGGGMISLIAALAVDRVIGMEN
template_upload.1.A  YEPDDWESVFSEFHDADAQNSHSYCFEILERR-----------------------

Target               AMPWNLPADLAWFKRNTLDKPVIMGRHTWESIGRPLPGRKNIILSSQPGTDDRV
template_upload.1.A  ------------------------------------------------------
```

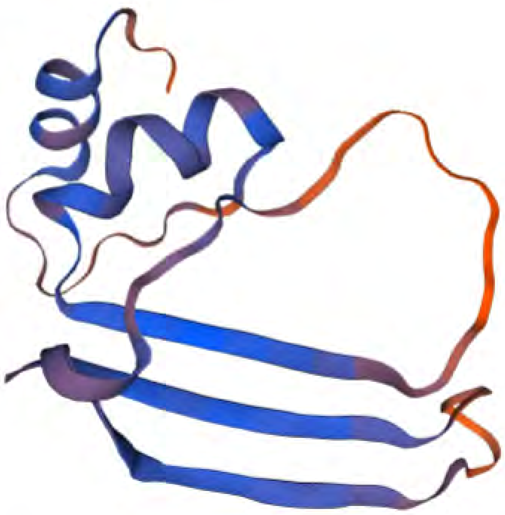

Template: PDB 1rx4A

CP site: Trp74

Target sequence:

WVKSVDEAIAACGDVPEIMVIGGGRVYEQFLPKAQKLYLTHIDAEVEGDTHFPDYEPDDW
ESVFSEFHDADAQNSHSYCFEILERRGGGGGMISLIAALAVDRVIGMENAMPWNLPADLA
WFKRNTLDKPVIMGRHTWESIGRPLPGRKNIILSSQPGTDDRVT

Model #01	File	Built with	Oligo-State	Ligands	GMQE	QMEAN
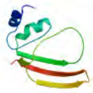	PDB	ProMod3 Version 1.2.0.	monomer	None	0.56	-0.24

QMEAN	-0.24	
Cβ	-0.36	
All Atom	0.30	
Solvation	-0.59	
Torsion	-0.06	

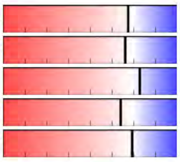

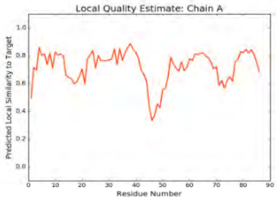

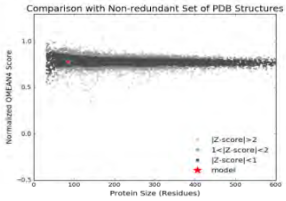

Template	Seq Identity	Oligo-state	Found by	Method	Resolution	Seq Similarity	Range	Coverage	Description
template_upload.1.A	100.00	monomer	BLAST	Unknown	NA	0.63	1 - 86	0.52	Polypeptide

The template contained no ligands.

```
Target                    WVKSVDEAIAACGDVPEIMVIGGGRVYEQFLPKAQKLYLTHIDAEVEGDTHFPDY
template_upload.1.A       WVKSVDEAIAACGDVPEIMVIGGGRVYEQFLPKAQKLYLTHIDAEVEGDTHFPDY

Target                    EPDDWESVFSEFHDADAQNSHSYCFEILERRGGGGGMISLIAALAVDRVIGMENA
template_upload.1.A       EPDDWESVFSEFHDADAQNSHSYCFEILERR------------------------

Target                    MPWNLPADLAWFKRNTLDKPVIMGRHTWESIGRPLPGRKNIILSSQPGTDDRVT
template_upload.1.A       ------------------------------------------------------
```

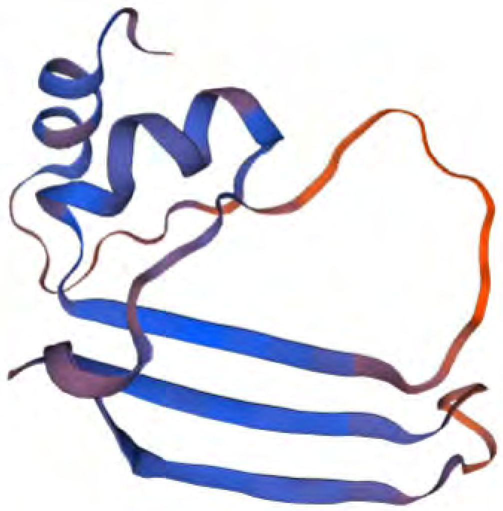

Template: PDB 1rx4A

CP site: Val75

Target sequence:

```
VKSVDEAIAACGDVPEIMVIGGGRVYEQFLPKAQKLYLTHIDAEVEGDTHFPDYEPDDWE
SVFSEFHDADAQNSHSYCFEILERRGGGGGMISLIAALAVDRVIGMENAMPWNLPADLAW
FKRNTLDKPVIMGRHTWESIGRPLPGRKNIILSSQPGTDDRVTW
```

Model #01	File	Built with	Oligo-State	Ligands	GMQE	QMEAN
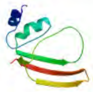	PDB	ProMod3 Version 1.2.0.	monomer	None	0.54	-0.20

QMEAN	-0.20	
Cβ	-0.43	
All Atom	0.35	
Solvation	-0.48	
Torsion	-0.04	

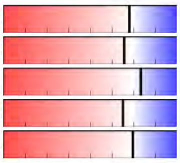

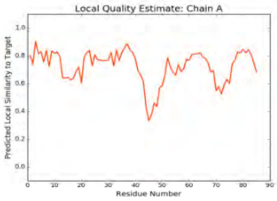

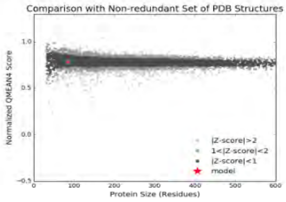

Template	Seq Identity	Oligo-state	Found by	Method	Resolution	Seq Similarity	Range	Coverage	Description
template_upload.1.A	100.00	monomer	BLAST	Unknown	NA	0.62	1 - 85	0.52	Polypeptide

The template contained no ligands.

```
Target              VKSVDEAIAACGDVPEIMVIGGGRVYEQFLPKAQKLYLTHIDAEVEGDTHFPDYE
template_upload.1.A VKSVDEAIAACGDVPEIMVIGGGRVYEQFLPKAQKLYLTHIDAEVEGDTHFPDYE

Target              PDDWESVFSEFHDADAQNSHSYCFEILERRGGGGGMISLIAALAVDRVIGMENAM
template_upload.1.A PDDWESVFSEFHDADAQNSHSYCFEILERR-------------------------

Target              PWNLPADLAWFKRNTLDKPVIMGRHTWESIGRPLPGRKNIILSSQPGTDDRVTW
template_upload.1.A ------------------------------------------------------
```

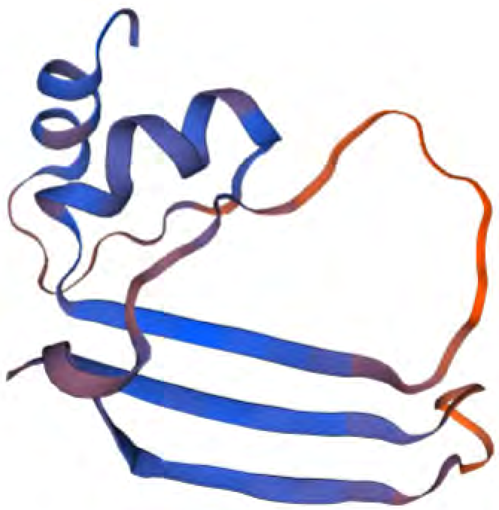

Template: PDB 1rx4A

CP site: Lys76

Target sequence:

KSVDEAIAACGDVPEIMVIGGGRVYEQFLPKAQKLYLTHIDAEVEGDTHFPDYEPDDWES
VFSEFHDADAQNSHSYCFEILERRGGGGGMISLIAALAVDRVIGMENAMPWNLPADLAWF
KRNTLDKPVIMGRHTWESIGRPLPGRKNIILSSQPGTDDRVTWV

Model #01	File	Built with	Oligo-State	Ligands	GMQE	QMEAN
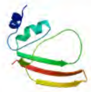	PDB	ProMod3 Version 1.2.0.	monomer	None	0.45	-0.30

QMEAN	-0.30
Cβ	-0.67
All Atom	0.43
Solvation	-0.56
Torsion	-0.07

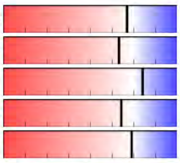

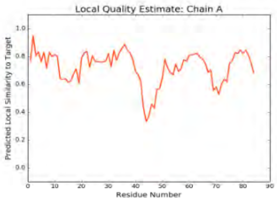

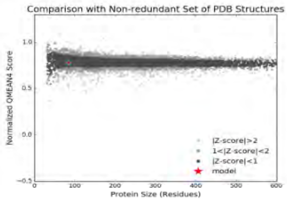

Template	Seq Identity	Oligo-state	Found by	Method	Resolution	Seq Similarity	Range	Coverage	Description
template_upload.1.A	100.00	monomer	HHblits	Unknown	NA	0.62	1 - 84	0.51	Polypeptide

The template contained no ligands.

```
Target                 KSVDEAIAACGDVPEIMVIGGGRVYEQFLPKAQKLYLTHIDAEVEGDTHFPDYEP
template_upload.1.A    KSVDEAIAACGDVPEIMVIGGGRVYEQFLPKAQKLYLTHIDAEVEGDTHFPDYEP

Target                 DDWESVFSEFHDADAQNSHSYCFEILERRGGGGGMISLIAALAVDRVIGMENAMP
template_upload.1.A    DDWESVFSEFHDADAQNSHSYCFEILERR--------------------------

Target                 WNLPADLAWFKRNTLDKPVIMGRHTWESIGRPLPGRKNIILSSQPGTDDRVTWV
template_upload.1.A    ------------------------------------------------------
```

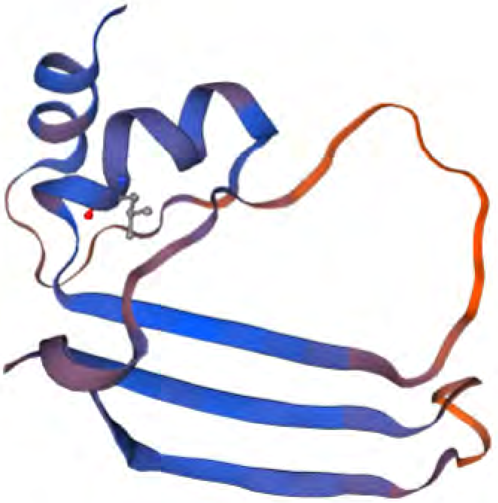

Template: PDB 1rx4A

CP site: Ser77

Target sequence:

SVDEAIAACGDVPEIMVIGGGRVYEQFLPKAQKLYLTHIDAEVEGDTHFPDYEPDDWESV
FSEFHDADAQNSHSYCFEILERRGGGGGMISLIAALAVDRVIGMENAMPWNLPADLAWFK
RNTLDKPVIMGRHTWESIGRPLPGRKNIILSSQPGTDDRVTWVK

Model #01	File	Built with	Oligo-State	Ligands	GMQE	QMEAN
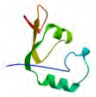	PDB	ProMod3 Version 1.2.0.	monomer	None	0.46	0.30

QMEAN	0.30	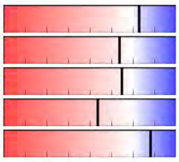
Cβ	-0.62	
All Atom	-0.52	
Solvation	-1.62	
Torsion	0.82	

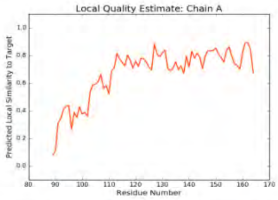

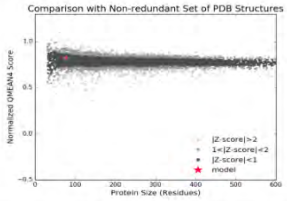

Template	Seq Identity	Oligo-state	Found by	Method	Resolution	Seq Similarity	Range	Coverage	Description
template_upload.1.A	100.00	monomer	BLAST	Unknown	NA	0.62	89 - 164	0.46	Polypeptide

The template contained no ligands.

```
Target                    SVDEAIAACGDVPEIMVIGGGRVYEQFLPKAQKLYLTHIDAEVEGDTHFPDYEPD
template_upload.1.A       -------------------------------------------------------

Target                    DWESVFSEFHDADAQNSHSYCFEILERRGGGGGMISLIAALAVDRVIGMENAMPW
template_upload.1.A       ---------------------------------MISLIAALAVDRVIGMENAMPW

Target                    NLPADLAWFKRNTLDKPVIMGRHTWESIGRPLPGRKNIILSSQPGTDDRVTWVK
template_upload.1.A       NLPADLAWFKRNTLDKPVIMGRHTWESIGRPLPGRKNIILSSQPGTDDRVTWVK
```

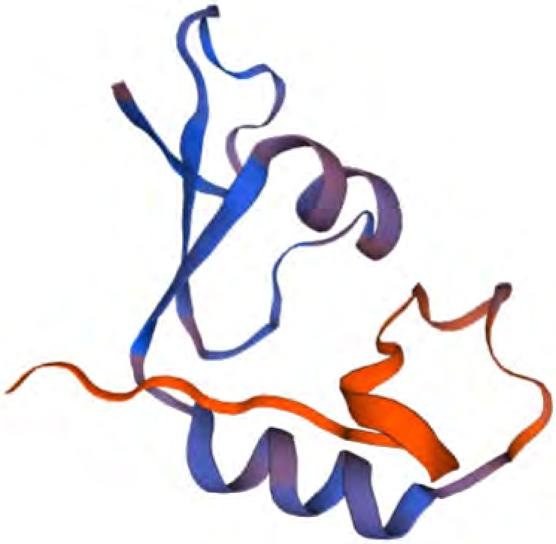

Template: PDB 1rx4A

CP site: Val78

Target sequence:

VDEAIAACGDVPEIMVIGGGRVYEQFLPKAQKLYLTHIDAEVEGDTHFPDYEPDDWESVF
SEFHDADAQNSHSYCFEILERRGGGGGMISLIAALAVDRVIGMENAMPWNLPADLAWFKR
NTLDKPVIMGRHTWESIGRPLPGRKNIILSSQPGTDDRVTWVKS

Model #01	File	Built with	Oligo-State	Ligands	GMQE	QMEAN
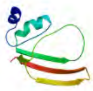	PDB	ProMod3 Version 1.2.0.	monomer	None	0.50	-0.20

QMEAN	-0.20	
Cβ	-0.68	
All Atom	0.33	
Solvation	-0.75	
Torsion	0.07	

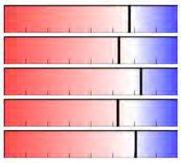

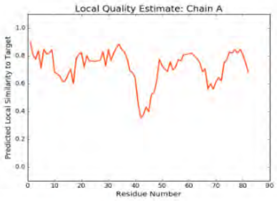

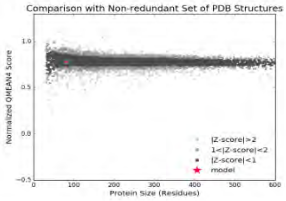

Template	Seq Identity	Oligo-state	Found by	Method	Resolution	Seq Similarity	Range	Coverage	Description
template_upload.1.A	100.00	monomer	BLAST	Unknown	NA	0.63	1 - 82	0.50	Polypeptide

The template contained no ligands.

```
Target               VDEAIAACGDVPEIMVIGGGRVYEQFLPKAQKLYLTHIDAEVEGDTHFPDYEPDD
template_upload.1.A  VDEAIAACGDVPEIMVIGGGRVYEQFLPKAQKLYLTHIDAEVEGDTHFPDYEPDD

Target               WESVFSEFHDADAQNSHSYCFEILERRGGGGGMISLIAALAVDRVIGMENAMPWN
template_upload.1.A  WESVFSEFHDADAQNSHSYCFEILERR----------------------------

Target               LPADLAWFKRNTLDKPVIMGRHTWESIGRPLPGRKNIILSSQPGTDDRVTWVKS
template_upload.1.A  ------------------------------------------------------
```

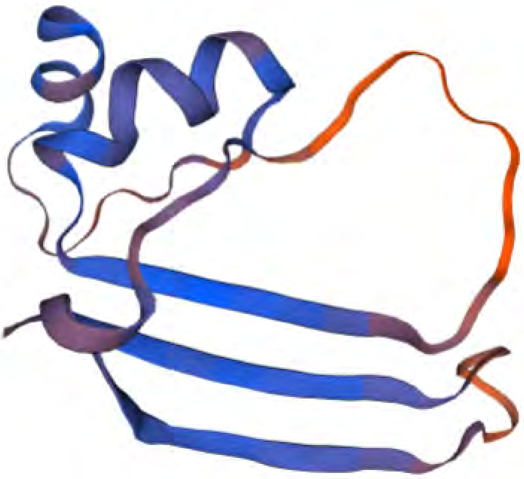

Template: PDB 1rx4A

CP site: Asp79

Target sequence:

```
DEAIAACGDVPEIMVIGGGRVYEQFLPKAQKLYLTHIDAEVEGDTHFPDYEPDDWESVFS
EFHDADAQNSHSYCFEILERRGGGGGMISLIAALAVDRVIGMENAMPWNLPADLAWFKRN
TLDKPVIMGRHTWESIGRPLPGRKNIILSSQPGTDDRVTWVKSV
```

Model #01	File	Built with	Oligo-State	Ligands	GMQE	QMEAN
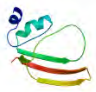	PDB	ProMod3 Version 1.2.0.	monomer	None	0.48	-0.39

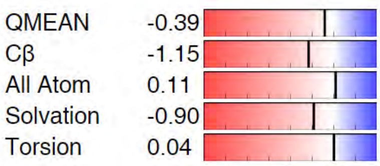

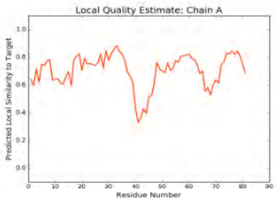

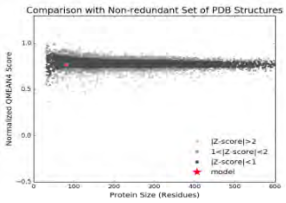

Template	Seq Identity	Oligo-state	Found by	Method	Resolution	Seq Similarity	Range	Coverage	Description
template_upload.1.A	100.00	monomer	BLAST	Unknown	NA	0.63	1 - 81	0.49	Polypeptide

The template contained no ligands.

```
Target                  DEAIAACGDVPEIMVIGGGRVYEQFLPKAQKLYLTHIDAEVEGDTHFPDYEPDDW
template_upload.1.A     DEAIAACGDVPEIMVIGGGRVYEQFLPKAQKLYLTHIDAEVEGDTHFPDYEPDDW

Target                  ESVFSEFHDADAQNSHSYCFEILERRGGGGGMISLIAALAVDRVIGMENAMPWNL
template_upload.1.A     ESVFSEFHDADAQNSHSYCFEILERR-----------------------------

Target                  PADLAWFKRNTLDKPVIMGRHTWESIGRPLPGRKNIILSSQPGTDDRVTWVKSV
template_upload.1.A     ------------------------------------------------------
```

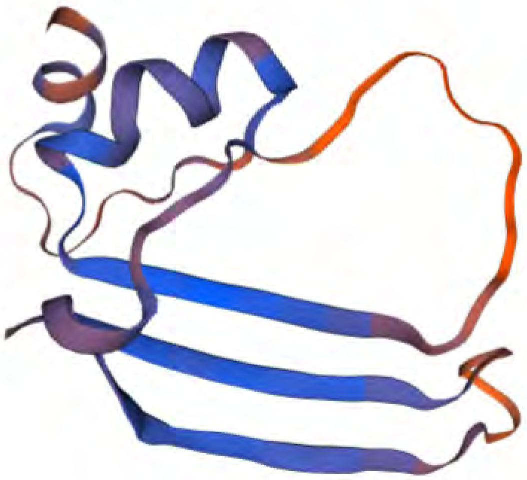

Template: PDB 1rx4A

CP site: Ala84

Target sequence:

ACGDVPEIMVIGGGRVYEQFLPKAQKLYLTHIDAEVEGDTHFPDYEPDDWESVFSEFHDA
DAQNSHSYCFEILERRGGGGGMISLIAALAVDRVIGMENAMPWNLPADLAWFKRNTLDKP
VIMGRHTWESIGRPLPGRKNIILSSQPGTDDRVTWVKSVDEAIA

Model #01	File	Built with	Oligo-State	Ligands	GMQE	QMEAN
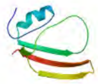	PDB	ProMod3 Version 1.2.0.	monomer	None	0.47	-0.36

QMEAN	-0.36	
Cβ	-0.91	
All Atom	-0.11	
Solvation	-1.03	
Torsion	0.07	

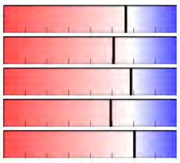

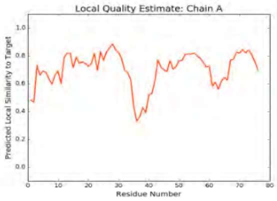

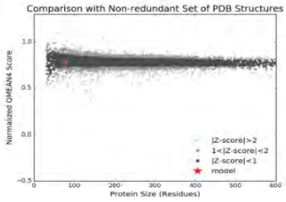

Template	Seq Identity	Oligo-state	Found by	Method	Resolution	Seq Similarity	Range	Coverage	Description
template_upload.1.A	100.00	monomer	BLAST	Unknown	NA	0.63	1 - 76	0.46	Polypeptide

The template contained no ligands.

```
Target                    ACGDVPEIMVIGGGRVYEQFLPKAQKLYLTHIDAEVEGDTHFPDYEPDDWESVFS
template_upload.1.A       ACGDVPEIMVIGGGRVYEQFLPKAQKLYLTHIDAEVEGDTHFPDYEPDDWESVFS

Target                    EFHDADAQNSHSYCFEILERRGGGGGMISLIAALAVDRVIGMENAMPWNLPADLA
template_upload.1.A       EFHDADAQNSHSYCFEILERR----------------------------------

Target                    WFKRNTLDKPVIMGRHTWESIGRPLPGRKNIILSSQPGTDDRVTWVKSVDEAIA
template_upload.1.A       ------------------------------------------------------
```

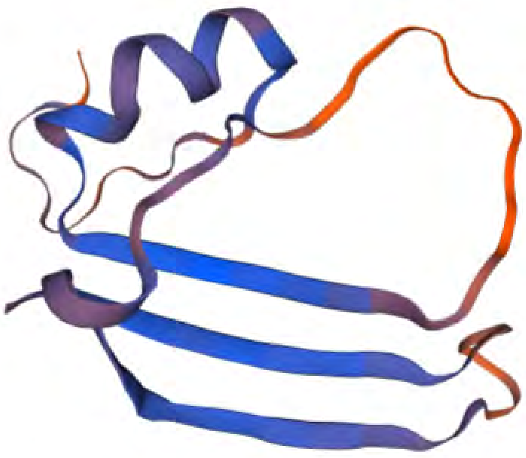

Template: PDB 1rx4A

CP site: Cys85

Target sequence:

```
CGDVPEIMVIGGGRVYEQFLPKAQKLYLTHIDAEVEGDTHFPDYEPDDWESVFSEFHDAD
AQNSHSYCFEILERRGGGGGMISLIAALAVDRVIGMENAMPWNLPADLAWFKRNTLDKPV
IMGRHTWESIGRPLPGRKNIILSSQPGTDDRVTWVKSVDEAIAA
```

Model #01	File	Built with	Oligo-State	Ligands	GMQE	QMEAN
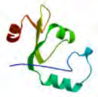	PDB	ProMod3 Version 1.2.0.	monomer	None	0.51	-0.05

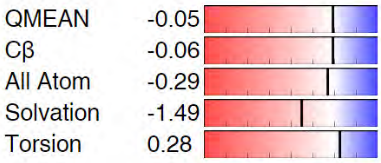

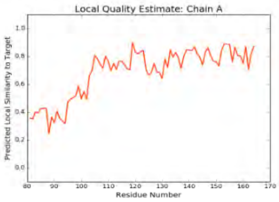

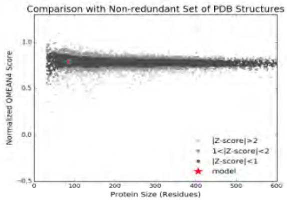

Template	Seq Identity	Oligo-state	Found by	Method	Resolution	Seq Similarity	Range	Coverage	Description
template_upload.1.A	100.00	monomer	BLAST	Unknown	NA	0.61	81 - 164	0.51	Polypeptide

The template contained no ligands.

```
Target                  CGDVPEIMVIGGGRVYEQFLPKAQKLYLTHIDAEVEGDTHFPDYEPDDWESVFSE
template_upload.1.A     -------------------------------------------------------

Target                  FHDADAQNSHSYCFEILERRGGGGGMISLIAALAVDRVIGMENAMPWNLPADLAW
template_upload.1.A     -------------------------MISLIAALAVDRVIGMENAMPWNLPADLAW

Target                  FKRNTLDKPVIMGRHTWESIGRPLPGRKNIILSSQPGTDDRVTWVKSVDEAIAA
template_upload.1.A     FKRNTLDKPVIMGRHTWESIGRPLPGRKNIILSSQPGTDDRVTWVKSVDEAIAA
```

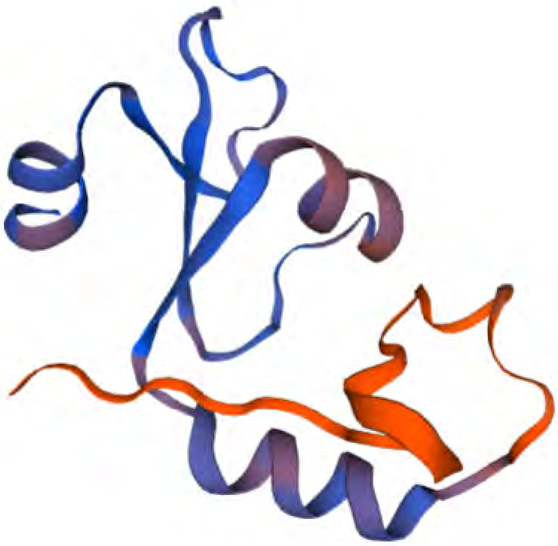

Template: PDB 1rx4A

CP site: Gly86

Target sequence:

GDVPEIMVIGGGRVYEQFLPKAQKLYLTHIDAEVEGDTHFPDYEPDDWESVFSEFHDADA
QNSHSYCFEILERRGGGGGMISLIAALAVDRVIGMENAMPWNLPADLAWFKRNTLDKPVI
MGRHTWESIGRPLPGRKNIILSSQPGTDDRVTWVKSVDEAIAAC

Model #01	File	Built with	Oligo-State	Ligands	GMQE	QMEAN
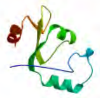	PDB	ProMod3 Version 1.2.0.	monomer	None	0.54	0.37

QMEAN	0.37	
Cβ	-0.16	
All Atom	-0.22	
Solvation	-1.47	
Torsion	0.73	

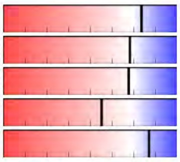

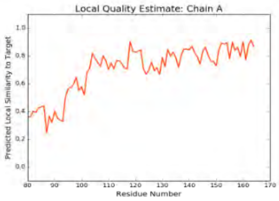

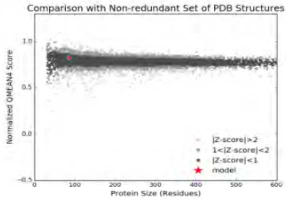

Template	Seq Identity	Oligo-state	Found by	Method	Resolution	Seq Similarity	Range	Coverage	Description
template_upload.1.A	100.00	monomer	BLAST	Unknown	NA	0.61	80 - 164	0.52	Polypeptide

The template contained no ligands.

```
Target                GDVPEIMVIGGGRVYEQFLPKAQKLYLTHIDAEVEGDTHFPDYEPDDWESVFSEF
template_upload.1.A   -------------------------------------------------------

Target                HDADAQNSHSYCFEILERRGGGGGMISLIAALAVDRVIGMENAMPWNLPADLAWF
template_upload.1.A   ------------------------MISLIAALAVDRVIGMENAMPWNLPADLAWF

Target                KRNTLDKPVIMGRHTWESIGRPLPGRKNIILSSQPGTDDRVTWVKSVDEAIAAC
template_upload.1.A   KRNTLDKPVIMGRHTWESIGRPLPGRKNIILSSQPGTDDRVTWVKSVDEAIAAC
```

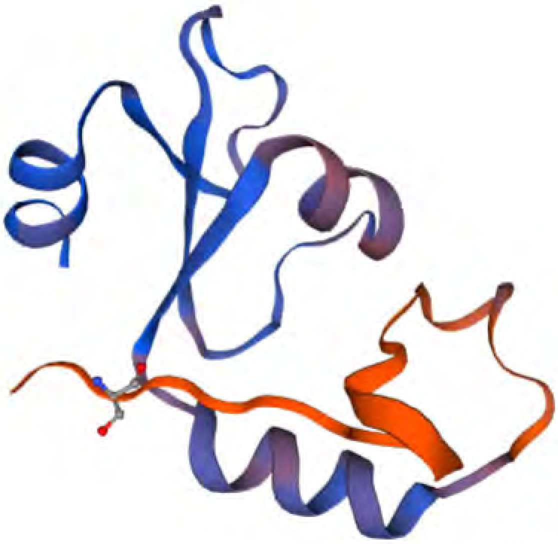

Template: PDB 1rx4A

CP site: Asp87

Target sequence:

```
DVPEIMVIGGGRVYEQFLPKAQKLYLTHIDAEVEGDTHFPDYEPDDWESVFSEFHDADAQ
NSHSYCFEILERRGGGGGMISLIAALAVDRVIGMENAMPWNLPADLAWFKRNTLDKPVIM
GRHTWESIGRPLPGRKNIILSSQPGTDDRVTWVKSVDEAIAACG
```

Model #01	File	Built with	Oligo-State	Ligands	GMQE	QMEAN
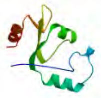	PDB	ProMod3 Version 1.2.0.	monomer	None	0.54	0.01

QMEAN	0.01	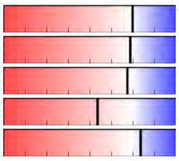
Cβ	-0.11	
All Atom	-0.25	
Solvation	-1.62	
Torsion	0.37	

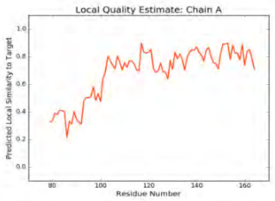

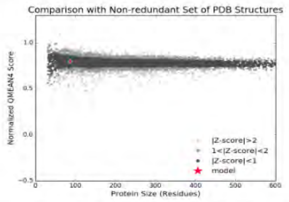

Template	Seq Identity	Oligo-state	Found by	Method	Resolution	Seq Similarity	Range	Coverage	Description
template_upload.1.A	100.00	monomer	BLAST	Unknown	NA	0.61	79 - 164	0.52	Polypeptide

The template contained no ligands.

```
Target                DVPEIMVIGGGRVYEQFLPKAQKLYLTHIDAEVEGDTHFPDYEPDDWESVFSEFH
template_upload.1.A   -------------------------------------------------------

Target                DADAQNSHSYCFEILERRGGGGGMISLIAALAVDRVIGMENAMPWNLPADLAWFK
template_upload.1.A   -----------------------MISLIAALAVDRVIGMENAMPWNLPADLAWFK

Target                RNTLDKPVIMGRHTWESIGRPLPGRKNIILSSQPGTDDRVTWVKSVDEAIAACG
template_upload.1.A   RNTLDKPVIMGRHTWESIGRPLPGRKNIILSSQPGTDDRVTWVKSVDEAIAACG
```

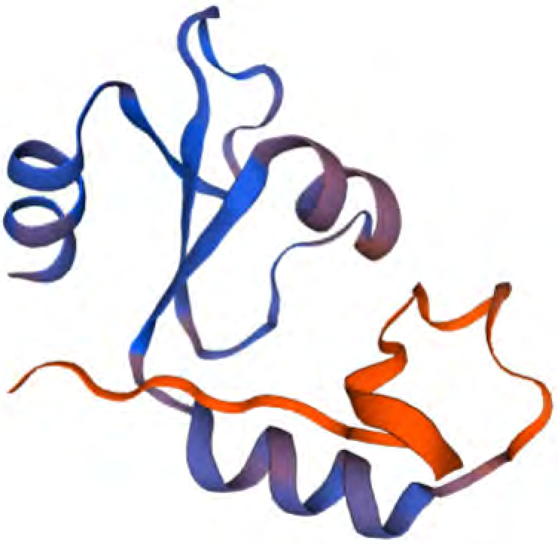

Template: PDB 1rx4A

CP site: Val88

Target sequence:

VPEIMVIGGGRVYEQFLPKAQKLYLTHIDAEVEGDTHFPDYEPDDWESVFSEFHDADAQN
SHSYCFEILERRGGGGGMISLIAALAVDRVIGMENAMPWNLPADLAWFKRNTLDKPVIMG
RHTWESIGRPLPGRKNIILSSQPGTDDRVTWVKSVDEAIAACGD

Model #01	File	Built with	Oligo-State	Ligands	GMQE	QMEAN
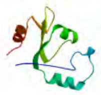	PDB	ProMod3 Version 1.2.0.	monomer	None	0.55	-0.03

QMEAN	-0.03	
Cβ	0.06	
All Atom	-0.21	
Solvation	-1.51	
Torsion	0.26	

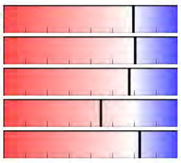

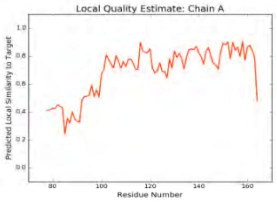

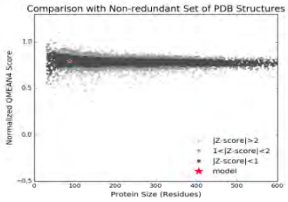

Template	Seq Identity	Oligo-state	Found by	Method	Resolution	Seq Similarity	Range	Coverage	Description
template_upload.1.A	100.00	monomer	BLAST	Unknown	NA	0.61	78 - 164	0.53	Polypeptide

The template contained no ligands.

```
Target               VPEIMVIGGGRVYEQFLPKAQKLYLTHIDAEVEGDTHFPDYEPDDWESVFSEFHD
template_upload.1.A  -------------------------------------------------------

Target               ADAQNSHSYCFEILERRGGGGGMISLIAALAVDRVIGMENAMPWNLPADLAWFKR
template_upload.1.A  ----------------------MISLIAALAVDRVIGMENAMPWNLPADLAWFKR

Target               NTLDKPVIMGRHTWESIGRPLPGRKNIILSSQPGTDDRVTWVKSVDEAIAACGD
template_upload.1.A  NTLDKPVIMGRHTWESIGRPLPGRKNIILSSQPGTDDRVTWVKSVDEAIAACGD
```

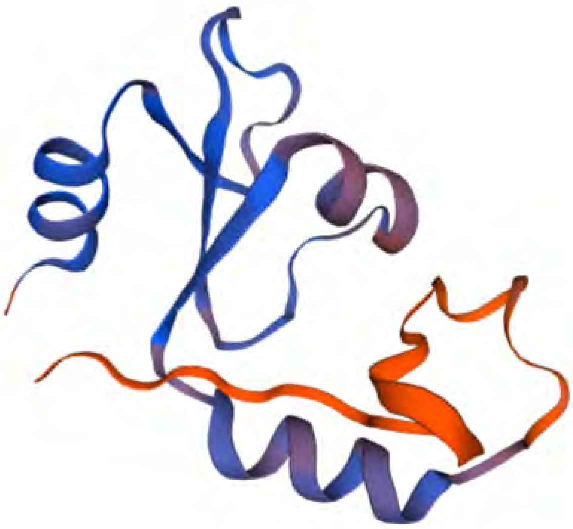

Template: PDB 1rx4A

CP site: Pro89

Target sequence:

PEIMVIGGGRVYEQFLPKAQKLYLTHIDAEVEGDTHFPDYEPDDWESVFSEFHDADAQNS
HSYCFEILERRGGGGGMISLIAALAVDRVIGMENAMPWNLPADLAWFKRNTLDKPVIMGR
HTWESIGRPLPGRKNIILSSQPGTDDRVTWVKSVDEAIAACGDV

Model #01	File	Built with	Oligo-State	Ligands	GMQE	QMEAN
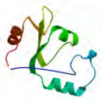	PDB	ProMod3 Version 1.2.0.	monomer	None	0.56	0.14

QMEAN	0.14	
Cβ	0.07	
All Atom	-0.27	
Solvation	-1.48	
Torsion	0.43	

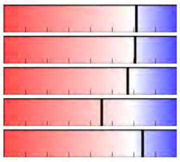

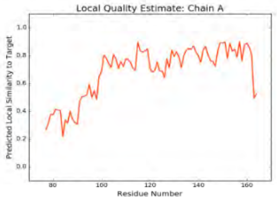

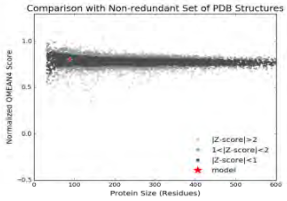

Template	Seq Identity	Oligo-state	Found by	Method	Resolution	Seq Similarity	Range	Coverage	Description
template_upload.1.A	100.00	monomer	BLAST	Unknown	NA	0.61	77 - 164	0.54	Polypeptide

The template contained no ligands.

```
Target                    PEIMVIGGGRVYEQFLPKAQKLYLTHIDAEVEGDTHFPDYEPDDWESVFSEFHDA
template_upload.1.A       -------------------------------------------------------

Target                    DAQNSHSYCFEILERRGGGGGMISLIAALAVDRVIGMENAMPWNLPADLAWFKRN
template_upload.1.A       ---------------------MISLIAALAVDRVIGMENAMPWNLPADLAWFKRN

Target                    TLDKPVIMGRHTWESIGRPLPGRKNIILSSQPGTDDRVTWVKSVDEAIAACGDV
template_upload.1.A       TLDKPVIMGRHTWESIGRPLPGRKNIILSSQPGTDDRVTWVKSVDEAIAACGDV
```

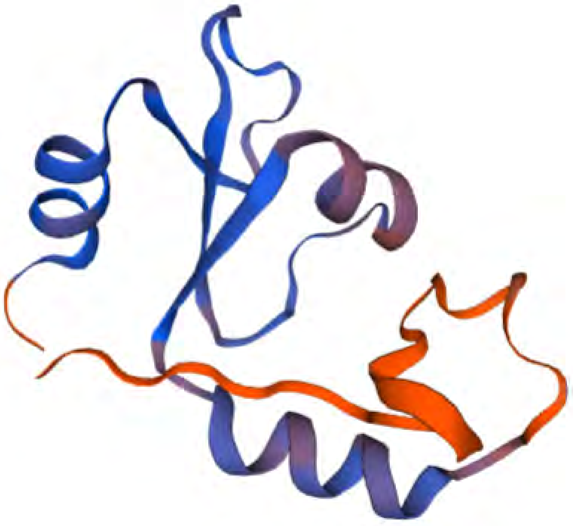

Template: PDB 1rx4A

CP site: Glu90

Target sequence:

```
EIMVIGGGRVYEQFLPKAQKLYLTHIDAEVEGDTHFPDYEPDDWESVFSEFHDADAQNSH
SYCFEILERRGGGGGMISLIAALAVDRVIGMENAMPWNLPADLAWFKRNTLDKPVIMGRH
TWESIGRPLPGRKNIILSSQPGTDDRVTWVKSVDEAIAACGDVP
```

Model #01	File	Built with	Oligo-State	Ligands	GMQE	QMEAN
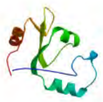	PDB	ProMod3 Version 1.2.0.	monomer	None	0.56	0.16

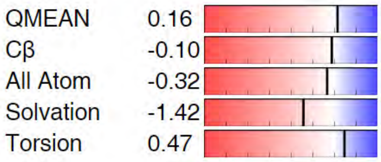

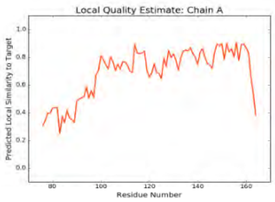

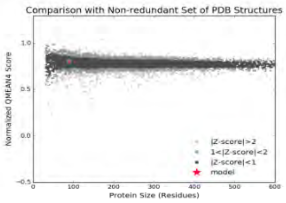

Template	Seq Identity	Oligo-state	Found by	Method	Resolution	Seq Similarity	Range	Coverage	Description
template_upload.1.A	100.00	monomer	BLAST	Unknown	NA	0.61	76 - 164	0.54	Polypeptide

The template contained no ligands.

```
Target                EIMVIGGGRVYEQFLPKAQKLYLTHIDAEVEGDTHFPDYEPDDWESVFSEFHDAD
template_upload.1.A   -------------------------------------------------------

Target                AQNSHSYCFEILERRGGGGGMISLIAALAVDRVIGMENAMPWNLPADLAWFKRNT
template_upload.1.A   --------------------MISLIAALAVDRVIGMENAMPWNLPADLAWFKRNT

Target                LDKPVIMGRHTWESIGRPLPGRKNIILSSQPGTDDRVTWVKSVDEAIAACGDVP
template_upload.1.A   LDKPVIMGRHTWESIGRPLPGRKNIILSSQPGTDDRVTWVKSVDEAIAACGDVP
```

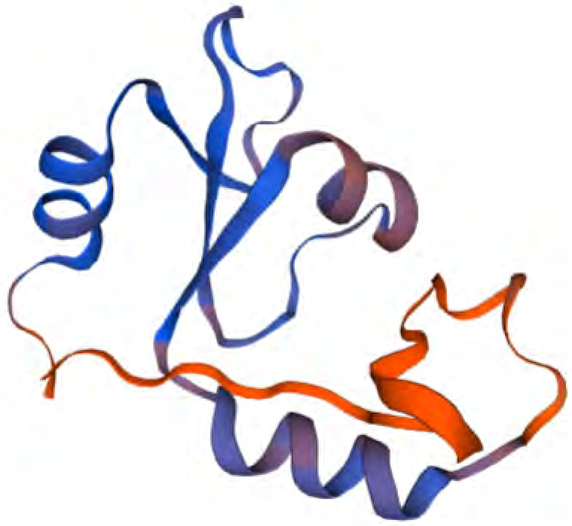

Template: PDB 1rx4A

CP site: Pro105

Target sequence:

PKAQKLYLTHIDAEVEGDTHFPDYEPDDWESVFSEFHDADAQNSHSYCFEILERRGGGGG
MISLIAALAVDRVIGMENAMPWNLPADLAWFKRNTLDKPVIMGRHTWESIGRPLPGRKNI
ILSSQPGTDDRVTWVKSVDEAIAACGDVPEIMVIGGGRVYEQFL

Model #01	File	Built with	Oligo-State	Ligands	GMQE	QMEAN
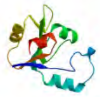	PDB	ProMod3 Version 1.2.0.	monomer	None	0.65	0.97

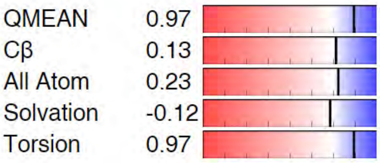

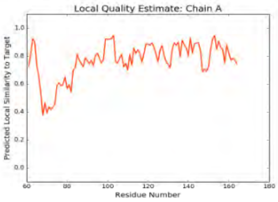

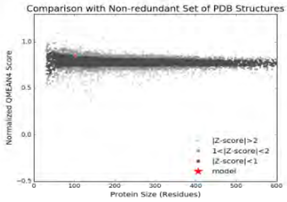

Template	Seq Identity	Oligo-state	Found by	Method	Resolution	Seq Similarity	Range	Coverage	Description
template_upload.1.A	100.00	monomer	HHblits	Unknown	NA	0.61	61 - 164	0.63	Polypeptide

The template contained no ligands.

```
Target               PKAQKLYLTHIDAEVEGDTHFPDYEPDDWESVFSEFHDADAQNSHSYCFEILERR
template_upload.1.A  -------------------------------------------------------

Target               GGGGGMISLIAALAVDRVIGMENAMPWNLPADLAWFKRNTLDKPVIMGRHTWESI
template_upload.1.A  -----MISLIAALAVDRVIGMENAMPWNLPADLAWFKRNTLDKPVIMGRHTWESI

Target               GRPLPGRKNIILSSQPGTDDRVTWVKSVDEAIAACGDVPEIMVIGGGRVYEQFL
template_upload.1.A  GRPLPGRKNIILSSQPGTDDRVTWVKSVDEAIAACGDVPEIMVIGGGRVYEQFL
```

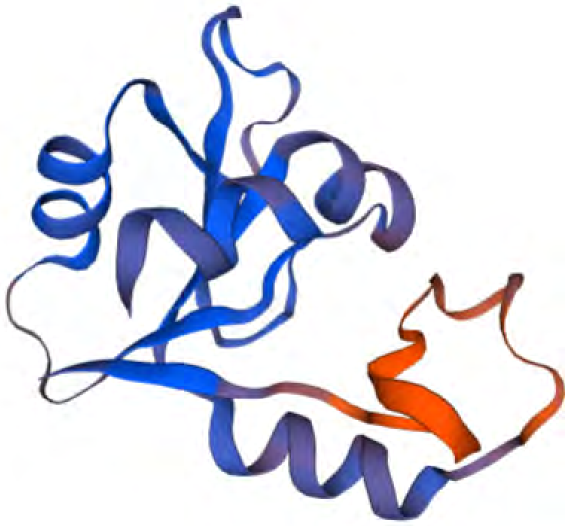

Template: PDB 1rx4A

CP site: Lys106

Target sequence:

KAQKLYLTHIDAEVEGDTHFPDYEPDDWESVFSEFHDADAQNSHSYCFEILERRGGGGGM
ISLIAALAVDRVIGMENAMPWNLPADLAWFKRNTLDKPVIMGRHTWESIGRPLPGRKNII
LSSQPGTDDRVTWVKSVDEAIAACGDVPEIMVIGGGRVYEQFLP

Model #01	File	Built with	Oligo-State	Ligands	GMQE	QMEAN
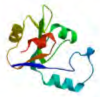	PDB	ProMod3 Version 1.2.0.	monomer	None	0.66	0.91

QMEAN	0.91	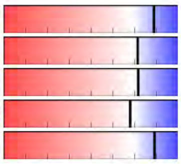
Cβ	0.15	
All Atom	0.18	
Solvation	-0.18	
Torsion	0.92	

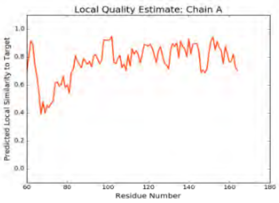

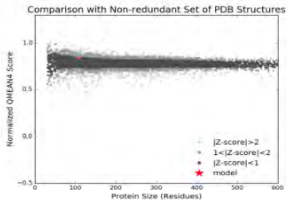

Template	Seq Identity	Oligo-state	Found by	Method	Resolution	Seq Similarity	Range	Coverage	Description
template_upload.1.A	100.00	monomer	HHblits	Unknown	NA	0.61	60 - 164	0.64	Polypeptide

The template contained no ligands.

```
Target                  KAQKLYLTHIDAEVEGDTHFPDYEPDDWESVFSEFHDADAQNSHSYCFEILERRG
template_upload.1.A     -------------------------------------------------------

Target                  GGGGMISLIAALAVDRVIGMENAMPWNLPADLAWFKRNTLDKPVIMGRHTWESIG
template_upload.1.A     ----MISLIAALAVDRVIGMENAMPWNLPADLAWFKRNTLDKPVIMGRHTWESIG

Target                  RPLPGRKNIILSSQPGTDDRVTWVKSVDEAIAACGDVPEIMVIGGGRVYEQFLP
template_upload.1.A     RPLPGRKNIILSSQPGTDDRVTWVKSVDEAIAACGDVPEIMVIGGGRVYEQFLP
```

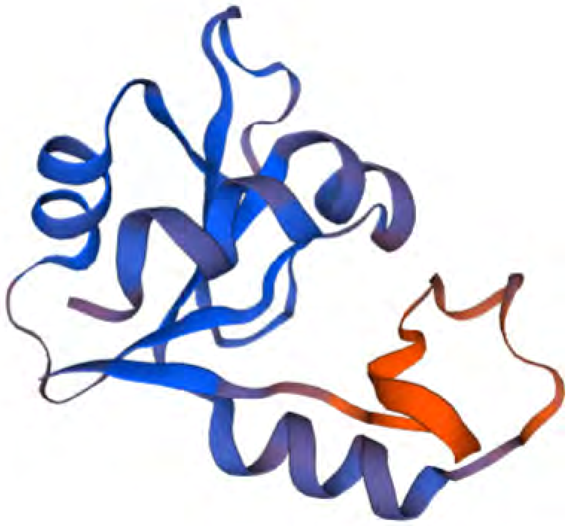

Template: PDB 1rx4A

CP site: Val119

Target sequence:

```
VEGDTHFPDYEPDDWESVFSEFHDADAQNSHSYCFEILERRGGGGGMISLIAALAVDRVI
GMENAMPWNLPADLAWFKRNTLDKPVIMGRHTWESIGRPLPGRKNIILSSQPGTDDRVTW
VKSVDEAIAACGDVPEIMVIGGGRVYEQFLPKAQKLYLTHIDAE
```

Model #01	File	Built with	Oligo-State	Ligands	GMQE	QMEAN
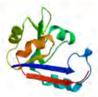	PDB	ProMod3 Version 1.2.0.	monomer	None	0.73	0.76

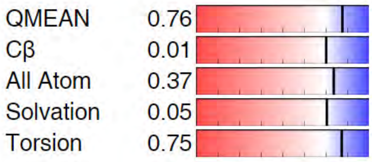

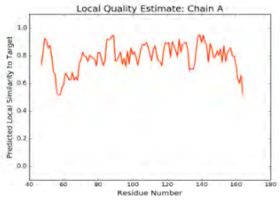

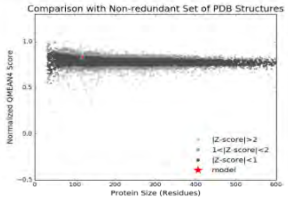

Template	Seq Identity	Oligo-state	Found by	Method	Resolution	Seq Similarity	Range	Coverage	Description
template_upload.1.A	100.00	monomer	BLAST	Unknown	NA	0.61	47 - 164	0.72	Polypeptide

The template contained no ligands.

```
Target                VEGDTHFPDYEPDDWESVFSEFHDADAQNSHSYCFEILERRGGGGGMISLIAALA
template_upload.1.A   ----------------------------------------------MISLIAALA

Target                VDRVIGMENAMPWNLPADLAWFKRNTLDKPVIMGRHTWESIGRPLPGRKNIILSS
template_upload.1.A   VDRVIGMENAMPWNLPADLAWFKRNTLDKPVIMGRHTWESIGRPLPGRKNIILSS

Target                QPGTDDRVTWVKSVDEAIAACGDVPEIMVIGGGRVYEQFLPKAQKLYLTHIDAE
template_upload.1.A   QPGTDDRVTWVKSVDEAIAACGDVPEIMVIGGGRVYEQFLPKAQKLYLTHIDAE
```

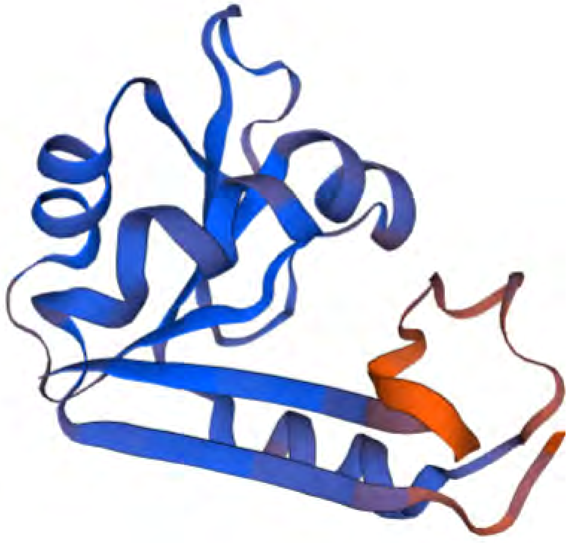

Template: PDB 1rx4A

CP site: Glu120

Target sequence:

EGDTHFPDYEPDDWESVFSEFHDADAQNSHSYCFEILERRGGGGGMISLIAALAVDRVIG
MENAMPWNLPADLAWFKRNTLDKPVIMGRHTWESIGRPLPGRKNIILSSQPGTDDRVTWV
KSVDEAIAACGDVPEIMVIGGGRVYEQFLPKAQKLYLTHIDAEV

Model #01	File	Built with	Oligo-State	Ligands	GMQE	QMEAN
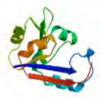	PDB	ProMod3 Version 1.2.0.	monomer	None	0.74	0.71

QMEAN	0.71	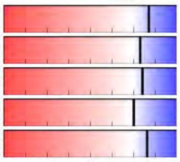
Cβ	0.32	
All Atom	0.43	
Solvation	0.02	
Torsion	0.63	

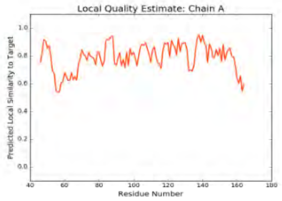

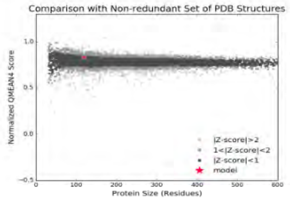

Template	Seq Identity	Oligo-state	Found by	Method	Resolution	Seq Similarity	Range	Coverage	Description
template_upload.1.A	100.00	monomer	BLAST	Unknown	NA	0.61	46 - 164	0.73	Polypeptide

The template contained no ligands.

```
Target                EGDTHFPDYEPDDWESVFSEFHDADAQNSHSYCFEILERRGGGGGMISLIAALAV
template_upload.1.A   ---------------------------------------------MISLIAALAV

Target                DRVIGMENAMPWNLPADLAWFKRNTLDKPVIMGRHTWESIGRPLPGRKNIILSSQ
template_upload.1.A   DRVIGMENAMPWNLPADLAWFKRNTLDKPVIMGRHTWESIGRPLPGRKNIILSSQ

Target                PGTDDRVTWVKSVDEAIAACGDVPEIMVIGGGRVYEQFLPKAQKLYLTHIDAEV
template_upload.1.A   PGTDDRVTWVKSVDEAIAACGDVPEIMVIGGGRVYEQFLPKAQKLYLTHIDAEV
```

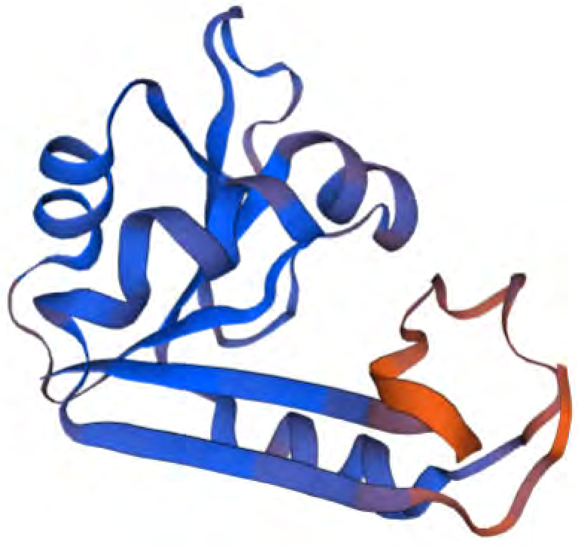

Template: PDB 1rx4A

CP site: Gly121

Target sequence:

```
GDTHFPDYEPDDWESVFSEFHDADAQNSHSYCFEILERRGGGGGMISLIAALAVDRVIGM
ENAMPWNLPADLAWFKRNTLDKPVIMGRHTWESIGRPLPGRKNIILSSQPGTDDRVTWVK
SVDEAIAACGDVPEIMVIGGGRVYEQFLPKAQKLYLTHIDAEVE
```

Model #01	File	Built with	Oligo-State	Ligands	GMQE	QMEAN
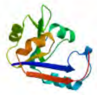	PDB	ProMod3 Version 1.2.0.	monomer	None	0.74	0.83

QMEAN	0.83	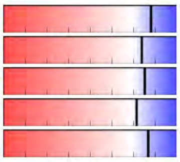
Cβ	0.39	
All Atom	0.52	
Solvation	0.17	
Torsion	0.70	

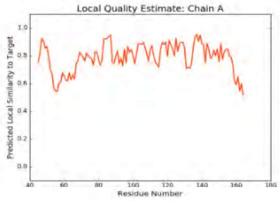

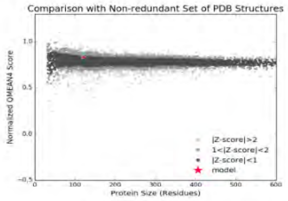

Template	Seq Identity	Oligo-state	Found by	Method	Resolution	Seq Similarity	Range	Coverage	Description
template_upload.1.A	100.00	monomer	BLAST	Unknown	NA	0.61	45 - 164	0.73	Polypeptide

The template contained no ligands.

```
Target                  GDTHFPDYEPDDWESVFSEFHDADAQNSHSYCFEILERRGGGGGMISLIAALAVD
template_upload.1.A     --------------------------------------------MISLIAALAVD

Target                  RVIGMENAMPWNLPADLAWFKRNTLDKPVIMGRHTWESIGRPLPGRKNIILSSQP
template_upload.1.A     RVIGMENAMPWNLPADLAWFKRNTLDKPVIMGRHTWESIGRPLPGRKNIILSSQP

Target                  GTDDRVTWVKSVDEAIAACGDVPEIMVIGGGRVYEQFLPKAQKLYLTHIDAEVE
template_upload.1.A     GTDDRVTWVKSVDEAIAACGDVPEIMVIGGGRVYEQFLPKAQKLYLTHIDAEVE
```

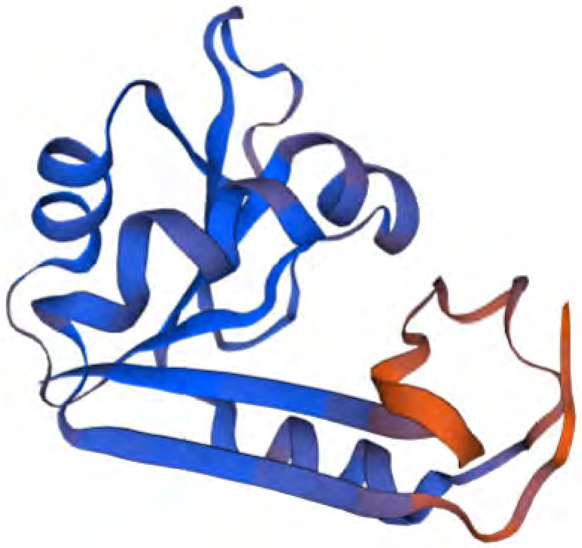

Template: PDB 1rx4A

CP site: Asp122

Target sequence:

DTHFPDYEPDDWESVFSEFHDADAQNSHSYCFEILERRGGGGGMISLIAALAVDRVIGME
NAMPWNLPADLAWFKRNTLDKPVIMGRHTWESIGRPLPGRKNIILSSQPGTDDRVTWVKS
VDEAIAACGDVPEIMVIGGGRVYEQFLPKAQKLYLTHIDAEVEG

Model #01	File	Built with	Oligo-State	Ligands	GMQE	QMEAN
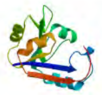	PDB	ProMod3 Version 1.2.0.	monomer	None	0.71	0.75

QMEAN	0.75	
Cβ	0.30	
All Atom	0.45	
Solvation	-0.00	
Torsion	0.68	

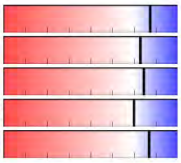

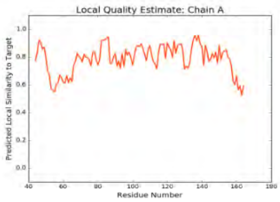

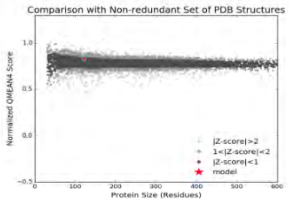

Template	Seq Identity	Oligo-state	Found by	Method	Resolution	Seq Similarity	Range	Coverage	Description
template_upload.1.A	100.00	monomer	HHblits	Unknown	NA	0.61	44 - 164	0.74	Polypeptide

The template contained no ligands.

```
Target                DTHFPDYEPDDWESVFSEFHDADAQNSHSYCFEILERRGGGGGMISLIAALAVDR
template_upload.1.A   -------------------------------------------MISLIAALAVDR

Target                VIGMENAMPWNLPADLAWFKRNTLDKPVIMGRHTWESIGRPLPGRKNIILSSQPG
template_upload.1.A   VIGMENAMPWNLPADLAWFKRNTLDKPVIMGRHTWESIGRPLPGRKNIILSSQPG

Target                TDDRVTWVKSVDEAIAACGDVPEIMVIGGGRVYEQFLPKAQKLYLTHIDAEVEG
template_upload.1.A   TDDRVTWVKSVDEAIAACGDVPEIMVIGGGRVYEQFLPKAQKLYLTHIDAEVEG
```

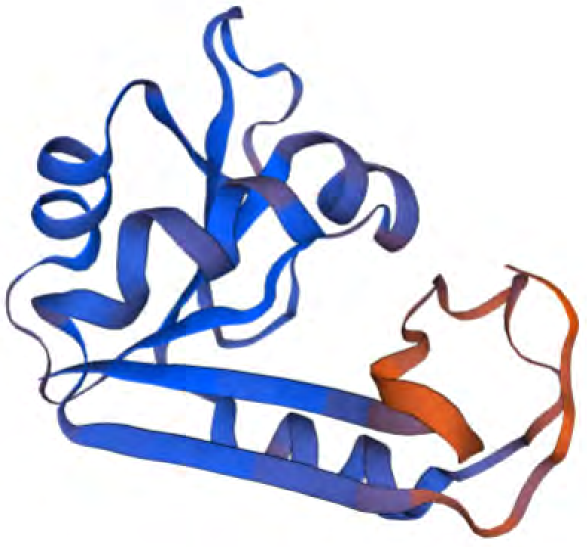

Template: PDB 1rx4A

CP site: Thr123

Target sequence:

THFPDYEPDDWESVFSEFHDADAQNSHSYCFEILERRGGGGGMISLIAALAVDRVIGMEN
AMPWNLPADLAWFKRNTLDKPVIMGRHTWESIGRPLPGRKNIILSSQPGTDDRVTWVKSV
DEAIAACGDVPEIMVIGGGRVYEQFLPKAQKLYLTHIDAEVEGD

Model #01	File	Built with	Oligo-State	Ligands	GMQE	QMEAN
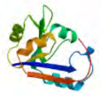	PDB	ProMod3 Version 1.2.0.	monomer	None	0.71	0.72

QMEAN	0.72	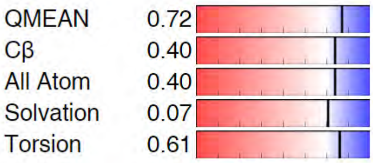
Cβ	0.40	
All Atom	0.40	
Solvation	0.07	
Torsion	0.61	

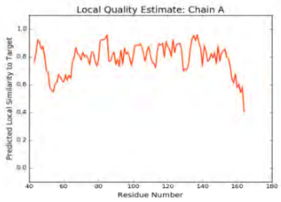

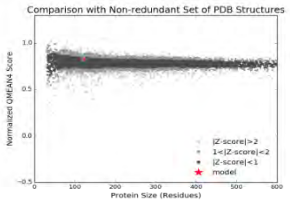

Template	Seq Identity	Oligo-state	Found by	Method	Resolution	Seq Similarity	Range	Coverage	Description
template_upload.1.A	100.00	monomer	HHblits	Unknown	NA	0.61	43 - 164	0.74	Polypeptide

The template contained no ligands.

```
Target                 THFPDYEPDDWESVFSEFHDADAQNSHSYCFEILERRGGGGGMISLIAALAVDRV
template_upload.1.A    ------------------------------------------MISLIAALAVDRV

Target                 IGMENAMPWNLPADLAWFKRNTLDKPVIMGRHTWESIGRPLPGRKNIILSSQPGT
template_upload.1.A    IGMENAMPWNLPADLAWFKRNTLDKPVIMGRHTWESIGRPLPGRKNIILSSQPGT

Target                 DDRVTWVKSVDEAIAACGDVPEIMVIGGGRVYEQFLPKAQKLYLTHIDAEVEGD
template_upload.1.A    DDRVTWVKSVDEAIAACGDVPEIMVIGGGRVYEQFLPKAQKLYLTHIDAEVEGD
```

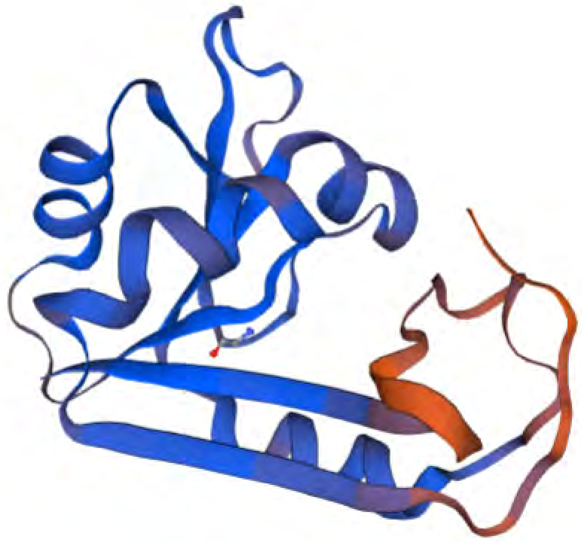

Template: PDB 1rx4A

CP site: Pro126

Target sequence:

PDYEPDDWESVFSEFHDADAQNSHSYCFEILERRGGGGGMISLIAALAVDRVIGMENAMP
WNLPADLAWFKRNTLDKPVIMGRHTWESIGRPLPGRKNIILSSQPGTDDRVTWVKSVDEA
IAACGDVPEIMVIGGGRVYEQFLPKAQKLYLTHIDAEVEGDTHF

Model #01	File	Built with	Oligo-State	Ligands	GMQE	QMEAN
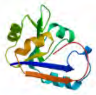	PDB	ProMod3 Version 1.2.0.	monomer	None	0.71	0.47

QMEAN	0.47	
Cβ	0.16	
All Atom	0.31	
Solvation	0.02	
Torsion	0.42	

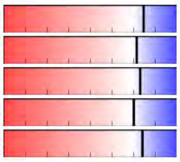

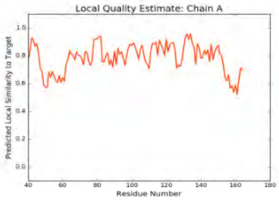

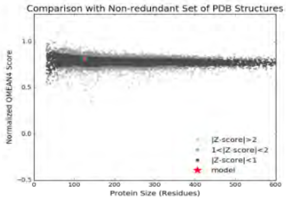

Template	Seq Identity	Oligo-state	Found by	Method	Resolution	Seq Similarity	Range	Coverage	Description
template_upload.1.A	100.00	monomer	HHblits	Unknown	NA	0.61	40 - 164	0.76	Polypeptide

The template contained no ligands.

```
Target                 PDYEPDDWESVFSEFHDADAQNSHSYCFEILERRGGGGGMISLIAALAVDRVIGM
template_upload.1.A    ---------------------------------------MISLIAALAVDRVIGM

Target                 ENAMPWNLPADLAWFKRNTLDKPVIMGRHTWESIGRPLPGRKNIILSSQPGTDDR
template_upload.1.A    ENAMPWNLPADLAWFKRNTLDKPVIMGRHTWESIGRPLPGRKNIILSSQPGTDDR

Target                 VTWVKSVDEAIAACGDVPEIMVIGGGRVYEQFLPKAQKLYLTHIDAEVEGDTHF
template_upload.1.A    VTWVKSVDEAIAACGDVPEIMVIGGGRVYEQFLPKAQKLYLTHIDAEVEGDTHF
```

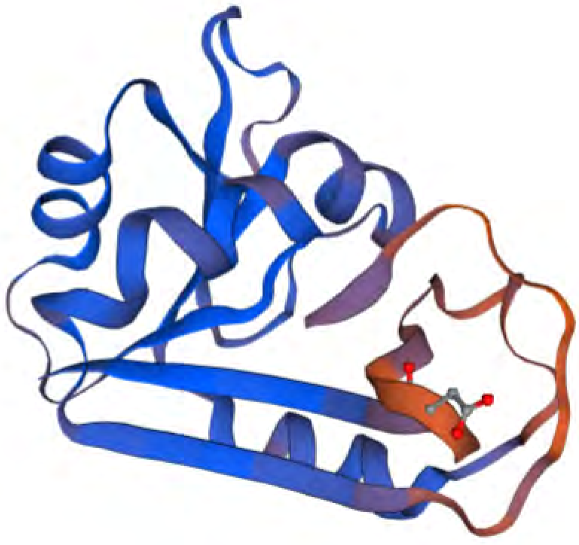

Template: PDB 1rx4A

CP site: Asp127

Target sequence:

```
DYEPDDWESVFSEFHDADAQNSHSYCFEILERRGGGGGMISLIAALAVDRVIGMENAMPW
NLPADLAWFKRNTLDKPVIMGRHTWESIGRPLPGRKNIILSSQPGTDDRVTWVKSVDEAI
AACGDVPEIMVIGGGRVYEQFLPKAQKLYLTHIDAEVEGDTHFP
```

Model #01	File	Built with	Oligo-State	Ligands	GMQE	QMEAN
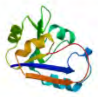	PDB	ProMod3 Version 1.2.0.	monomer	None	0.72	0.14

QMEAN	0.14	
Cβ	0.25	
All Atom	0.26	
Solvation	-0.01	
Torsion	0.07	

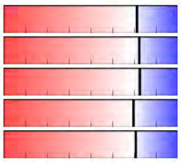

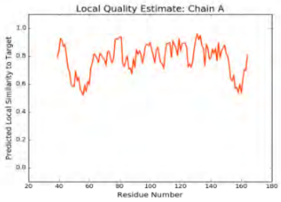

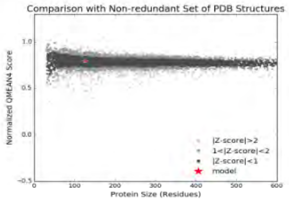

Template	Seq Identity	Oligo-state	Found by	Method	Resolution	Seq Similarity	Range	Coverage	Description
template_upload.1.A	100.00	monomer	HHblits	Unknown	NA	0.61	39 - 164	0.77	Polypeptide

The template contained no ligands.

```
Target               DYEPDDWESVFSEFHDADAQNSHSYCFEILERRGGGGGMISLIAALAVDRVIGME
template_upload.1.A  --------------------------------------MISLIAALAVDRVIGME

Target               NAMPWNLPADLAWFKRNTLDKPVIMGRHTWESIGRPLPGRKNIILSSQPGTDDRV
template_upload.1.A  NAMPWNLPADLAWFKRNTLDKPVIMGRHTWESIGRPLPGRKNIILSSQPGTDDRV

Target               TWVKSVDEAIAACGDVPEIMVIGGGRVYEQFLPKAQKLYLTHIDAEVEGDTHFP
template_upload.1.A  TWVKSVDEAIAACGDVPEIMVIGGGRVYEQFLPKAQKLYLTHIDAEVEGDTHFP
```

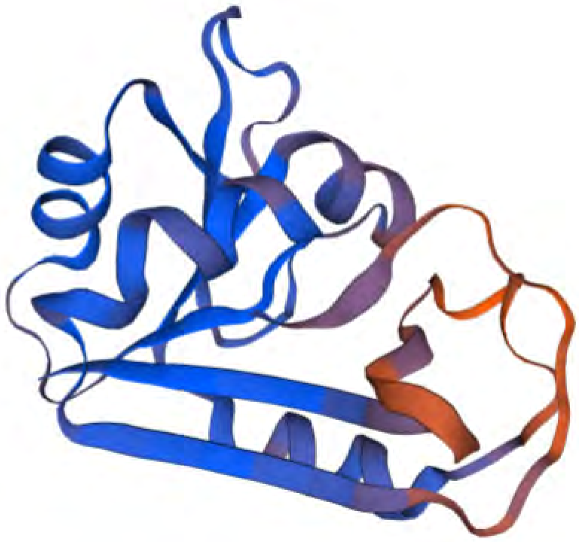

Template: PDB 1rx4A

CP site: Tyr128

Target sequence:

YEPDDWESVFSEFHDADAQNSHSYCFEILERRGGGGGMISLIAALAVDRVIGMENAMPWN
LPADLAWFKRNTLDKPVIMGRHTWESIGRPLPGRKNIILSSQPGTDDRVTWVKSVDEAIA
ACGDVPEIMVIGGGRVYEQFLPKAQKLYLTHIDAEVEGDTHFPD

Model #01	File	Built with	Oligo-State	Ligands	GMQE	QMEAN
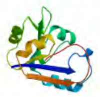	PDB	ProMod3 Version 1.2.0.	monomer	None	0.73	0.21

QMEAN	0.21
Cβ	0.43
All Atom	0.39
Solvation	-0.01
Torsion	0.09

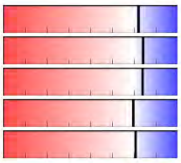

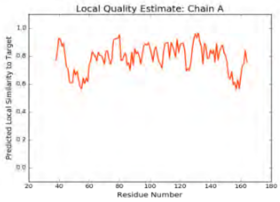

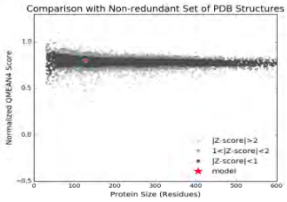

Template	Seq Identity	Oligo-state	Found by	Method	Resolution	Seq Similarity	Range	Coverage	Description
template_upload.1.A	100.00	monomer	HHblits	Unknown	NA	0.62	38 - 164	0.77	Polypeptide

The template contained no ligands.

```
Target                  YEPDDWESVFSEFHDADAQNSHSYCFEILERRGGGGGMISLIAALAVDRVIGMEN
template_upload.1.A     -------------------------------------MISLIAALAVDRVIGMEN

Target                  AMPWNLPADLAWFKRNTLDKPVIMGRHTWESIGRPLPGRKNIILSSQPGTDDRVT
template_upload.1.A     AMPWNLPADLAWFKRNTLDKPVIMGRHTWESIGRPLPGRKNIILSSQPGTDDRVT

Target                  WVKSVDEAIAACGDVPEIMVIGGGRVYEQFLPKAQKLYLTHIDAEVEGDTHFPD
template_upload.1.A     WVKSVDEAIAACGDVPEIMVIGGGRVYEQFLPKAQKLYLTHIDAEVEGDTHFPD
```

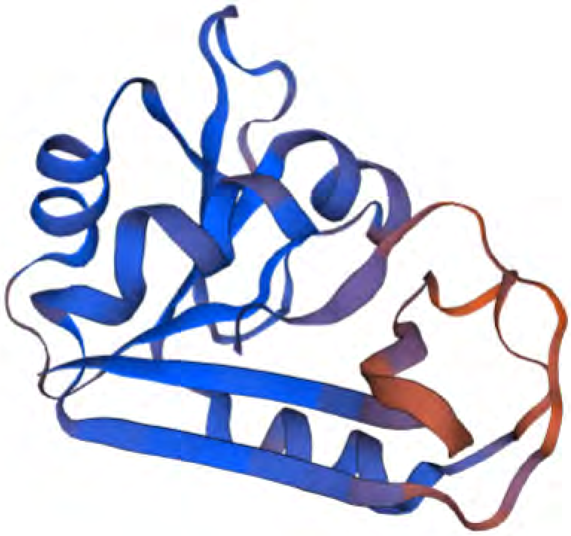

Template: PDB 1rx4A

CP site: Glu129

Target sequence:

```
EPDDWESVFSEFHDADAQNSHSYCFEILERRGGGGGMISLIAALAVDRVIGMENAMPWNL
PADLAWFKRNTLDKPVIMGRHTWESIGRPLPGRKNIILSSQPGTDDRVTWVKSVDEAIAA
CGDVPEIMVIGGGRVYEQFLPKAQKLYLTHIDAEVEGDTHFPDY
```

Model #01	File	Built with	Oligo-State	Ligands	GMQE	QMEAN
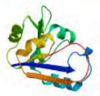	PDB	ProMod3 Version 1.2.0.	monomer	None	0.71	0.26

QMEAN	0.26	
Cβ	0.38	
All Atom	0.33	
Solvation	-0.03	
Torsion	0.17	

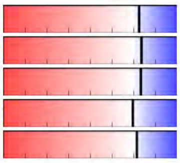

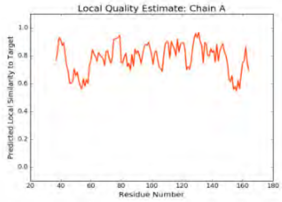

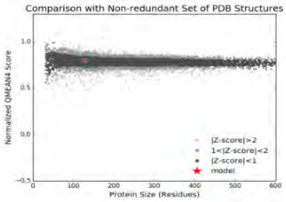

Template	Seq Identity	Oligo-state	Found by	Method	Resolution	Seq Similarity	Range	Coverage	Description
template_upload.1.A	100.00	monomer	HHblits	Unknown	NA	0.62	37 - 164	0.78	Polypeptide

The template contained no ligands.

```
Target                  EPDDWESVFSEFHDADAQNSHSYCFEILERRGGGGGMISLIAALAVDRVIGMENA
template_upload.1.A     ------------------------------------MISLIAALAVDRVIGMENA

Target                  MPWNLPADLAWFKRNTLDKPVIMGRHTWESIGRPLPGRKNIILSSQPGTDDRVTW
template_upload.1.A     MPWNLPADLAWFKRNTLDKPVIMGRHTWESIGRPLPGRKNIILSSQPGTDDRVTW

Target                  VKSVDEAIAACGDVPEIMVIGGGRVYEQFLPKAQKLYLTHIDAEVEGDTHFPDY
template_upload.1.A     VKSVDEAIAACGDVPEIMVIGGGRVYEQFLPKAQKLYLTHIDAEVEGDTHFPDY
```

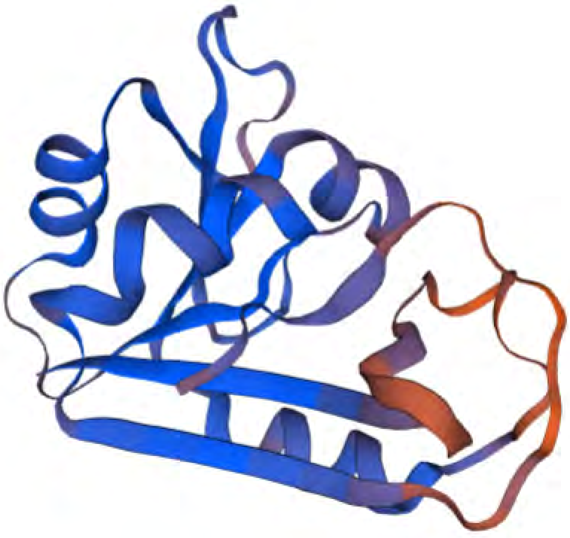

Template: PDB 1rx4A

CP site: Pro130

Target sequence:

PDDWESVFSEFHDADAQNSHSYCFEILERRGGGGGMISLIAALAVDRVIGMENAMPWNLP
ADLAWFKRNTLDKPVIMGRHTWESIGRPLPGRKNIILSSQPGTDDRVTWVKSVDEAIAAC
GDVPEIMVIGGGRVYEQFLPKAQKLYLTHIDAEVEGDTHFPDYE

Model #01	File	Built with	Oligo-State	Ligands	GMQE	QMEAN
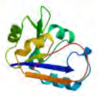	PDB	ProMod3 Version 1.2.0.	monomer	None	0.73	0.31

QMEAN	0.31	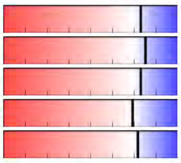
Cβ	0.54	
All Atom	0.33	
Solvation	-0.05	
Torsion	0.20	

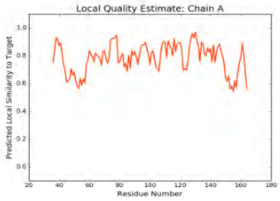

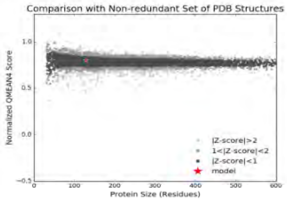

Template	Seq Identity	Oligo-state	Found by	Method	Resolution	Seq Similarity	Range	Coverage	Description
template_upload.1.A	100.00	monomer	HHblits	Unknown	NA	0.62	36 - 164	0.79	Polypeptide

The template contained no ligands.

```
Target                    PDDWESVFSEFHDADAQNSHSYCFEILERRGGGGGMISLIAALAVDRVIGMENAM
template_upload.1.A       -----------------------------------MISLIAALAVDRVIGMENAM

Target                    PWNLPADLAWFKRNTLDKPVIMGRHTWESIGRPLPGRKNIILSSQPGTDDRVTWV
template_upload.1.A       PWNLPADLAWFKRNTLDKPVIMGRHTWESIGRPLPGRKNIILSSQPGTDDRVTWV

Target                    KSVDEAIAACGDVPEIMVIGGGRVYEQFLPKAQKLYLTHIDAEVEGDTHFPDYE
template_upload.1.A       KSVDEAIAACGDVPEIMVIGGGRVYEQFLPKAQKLYLTHIDAEVEGDTHFPDYE
```

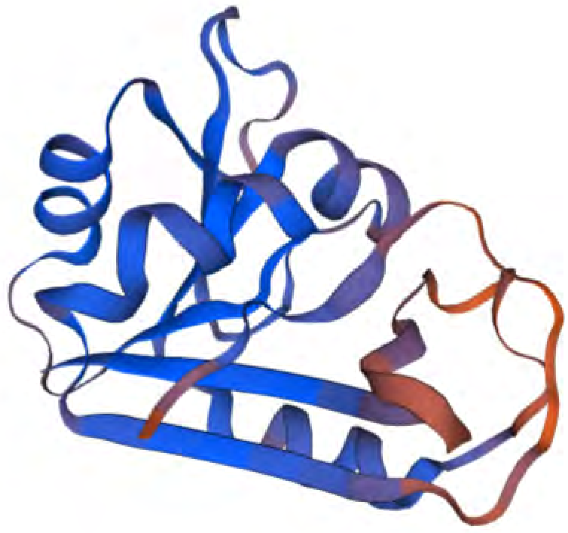

Template: PDB 1rx4A

CP site: Asp131

Target sequence:

```
DDWESVFSEFHDADAQNSHSYCFEILERRGGGGGMISLIAALAVDRVIGMENAMPWNLPA
DLAWFKRNTLDKPVIMGRHTWESIGRPLPGRKNIILSSQPGTDDRVTWVKSVDEAIAACG
DVPEIMVIGGGRVYEQFLPKAQKLYLTHIDAEVEGDTHFPDYEP
```

Model #01	File	Built with	Oligo-State	Ligands	GMQE	QMEAN
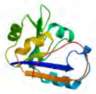	PDB	ProMod3 Version 1.2.0.	monomer	None	0.79	0.30

QMEAN	0.30	
Cβ	0.52	
All Atom	0.24	
Solvation	-0.09	
Torsion	0.20	

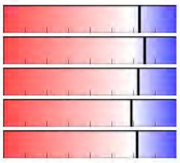

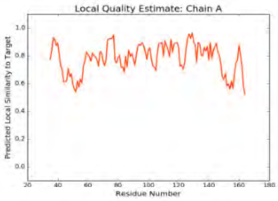

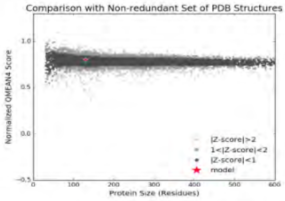

Template	Seq Identity	Oligo-state	Found by	Method	Resolution	Seq Similarity	Range	Coverage	Description
template_upload.1.A	100.00	monomer	BLAST	Unknown	NA	0.62	35 - 164	0.79	Polypeptide

The template contained no ligands.

```
Target                DDWESVFSEFHDADAQNSHSYCFEILERRGGGGGMISLIAALAVDRVIGMENAMP
template_upload.1.A   ----------------------------------MISLIAALAVDRVIGMENAMP

Target                WNLPADLAWFKRNTLDKPVIMGRHTWESIGRPLPGRKNIILSSQPGTDDRVTWVK
template_upload.1.A   WNLPADLAWFKRNTLDKPVIMGRHTWESIGRPLPGRKNIILSSQPGTDDRVTWVK

Target                SVDEAIAACGDVPEIMVIGGGRVYEQFLPKAQKLYLTHIDAEVEGDTHFPDYEP
template_upload.1.A   SVDEAIAACGDVPEIMVIGGGRVYEQFLPKAQKLYLTHIDAEVEGDTHFPDYEP
```

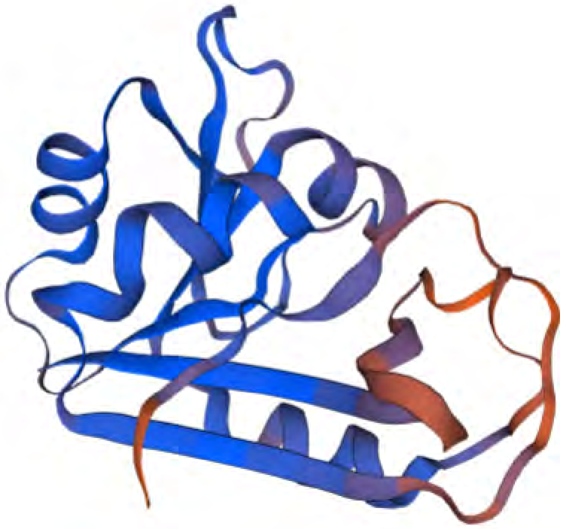

Template: PDB 1rx4A

CP site: Asp132

Target sequence:

```
DWESVFSEFHDADAQNSHSYCFEILERRGGGGGMISLIAALAVDRVIGMENAMPWNLPAD
LAWFKRNTLDKPVIMGRHTWESIGRPLPGRKNIILSSQPGTDDRVTWVKSVDEAIAACGD
VPEIMVIGGGRVYEQFLPKAQKLYLTHIDAEVEGDTHFPDYEPD
```

Model #01	File	Built with	Oligo-State	Ligands	GMQE	QMEAN
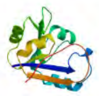	PDB	ProMod3 Version 1.2.0.	monomer	None	0.79	0.34

QMEAN	0.34	
Cβ	0.60	
All Atom	0.30	
Solvation	-0.08	
Torsion	0.22	

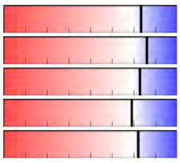

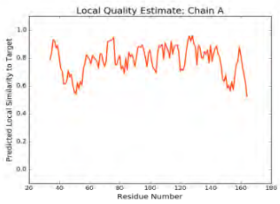

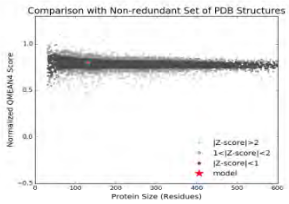

Template	Seq Identity	Oligo-state	Found by	Method	Resolution	Seq Similarity	Range	Coverage	Description
template_upload.1.A	100.00	monomer	BLAST	Unknown	NA	0.62	34 - 164	0.80	Polypeptide

The template contained no ligands.

```
Target               DWESVFSEFHDADAQNSHSYCFEILERRGGGGGMISLIAALAVDRVIGMENAMPW
template_upload.1.A  ---------------------------------MISLIAALAVDRVIGMENAMPW

Target               NLPADLAWFKRNTLDKPVIMGRHTWESIGRPLPGRKNIILSSQPGTDDRVTWVKS
template_upload.1.A  NLPADLAWFKRNTLDKPVIMGRHTWESIGRPLPGRKNIILSSQPGTDDRVTWVKS

Target               VDEAIAACGDVPEIMVIGGGRVYEQFLPKAQKLYLTHIDAEVEGDTHFPDYEPD
template_upload.1.A  VDEAIAACGDVPEIMVIGGGRVYEQFLPKAQKLYLTHIDAEVEGDTHFPDYEPD
```

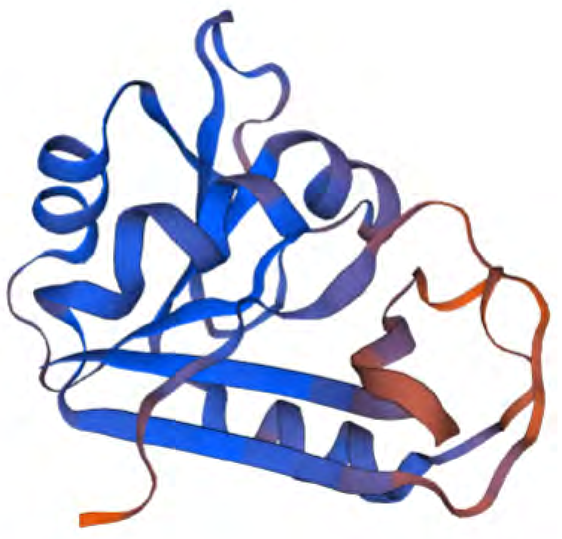

Template: PDB 1rx4A

CP site: Trp133

Target sequence:

WESVFSEFHDADAQNSHSYCFEILERRGGGGGMISLIAALAVDRVIGMENAMPWNLPADL
AWFKRNTLDKPVIMGRHTWESIGRPLPGRKNIILSSQPGTDDRVTWVKSVDEAIAACGDV
PEIMVIGGGRVYEQFLPKAQKLYLTHIDAEVEGDTHFPDYEPDD

Model #01	File	Built with	Oligo-State	Ligands	GMQE	QMEAN
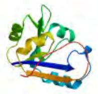	PDB	ProMod3 Version 1.2.0.	monomer	None	0.80	0.31

QMEAN	0.31
Cβ	0.60
All Atom	0.31
Solvation	-0.06
Torsion	0.19

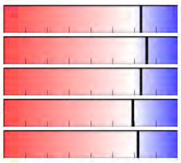

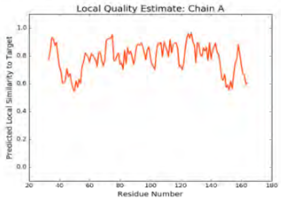

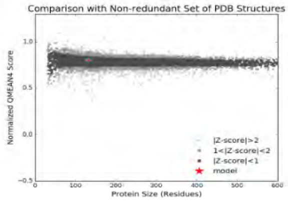

Template	Seq Identity	Oligo-state	Found by	Method	Resolution	Seq Similarity	Range	Coverage	Description
template_upload.1.A	100.00	monomer	BLAST	Unknown	NA	0.62	33 - 164	0.80	Polypeptide

The template contained no ligands.

```
Target                  WESVFSEFHDADAQNSHSYCFEILERRGGGGGMISLIAALAVDRVIGMENAMPWN
template_upload.1.A     --------------------------------MISLIAALAVDRVIGMENAMPWN

Target                  LPADLAWFKRNTLDKPVIMGRHTWESIGRPLPGRKNIILSSQPGTDDRVTWVKSV
template_upload.1.A     LPADLAWFKRNTLDKPVIMGRHTWESIGRPLPGRKNIILSSQPGTDDRVTWVKSV

Target                  DEAIAACGDVPEIMVIGGGRVYEQFLPKAQKLYLTHIDAEVEGDTHFPDYEPDD
template_upload.1.A     DEAIAACGDVPEIMVIGGGRVYEQFLPKAQKLYLTHIDAEVEGDTHFPDYEPDD
```

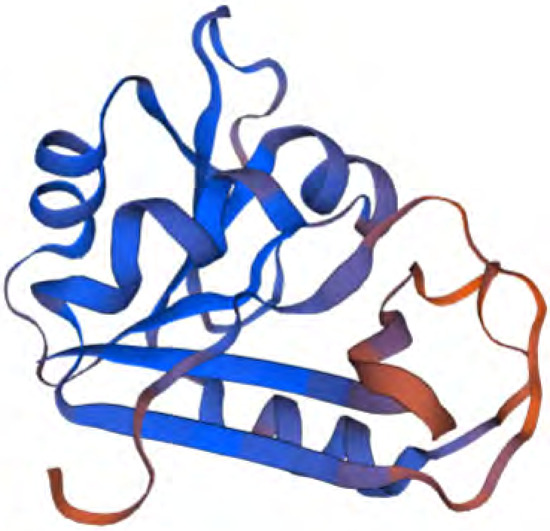

Template: PDB 1rx4A

CP site: Glu134

Target sequence:

```
ESVFSEFHDADAQNSHSYCFEILERRGGGGGMISLIAALAVDRVIGMENAMPWNLPADLA
WFKRNTLDKPVIMGRHTWESIGRPLPGRKNIILSSQPGTDDRVTWVKSVDEAIAACGDVP
EIMVIGGGRVYEQFLPKAQKLYLTHIDAEVEGDTHFPDYEPDDW
```

Model #01	File	Built with	Oligo-State	Ligands	GMQE	QMEAN
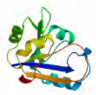	PDB	ProMod3 Version 1.2.0.	monomer	None	0.77	0.38

QMEAN	0.38	
Cβ	0.23	
All Atom	0.25	
Solvation	0.02	
Torsion	0.32	

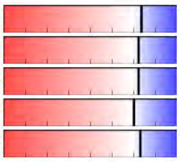

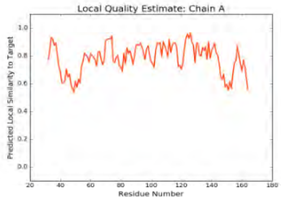

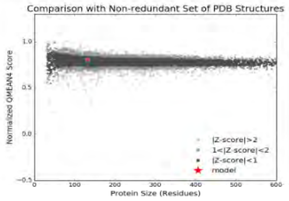

Template	Seq Identity	Oligo-state	Found by	Method	Resolution	Seq Similarity	Range	Coverage	Description
template_upload.1.A	100.00	monomer	HHblits	Unknown	NA	0.62	32 - 164	0.81	Polypeptide

The template contained no ligands.

```
Target                ESVFSEFHDADAQNSHSYCFEILERRGGGGGMISLIAALAVDRVIGMENAMPWNL
template_upload.1.A   -------------------------------MISLIAALAVDRVIGMENAMPWNL

Target                PADLAWFKRNTLDKPVIMGRHTWESIGRPLPGRKNIILSSQPGTDDRVTWVKSVD
template_upload.1.A   PADLAWFKRNTLDKPVIMGRHTWESIGRPLPGRKNIILSSQPGTDDRVTWVKSVD

Target                EAIAACGDVPEIMVIGGGRVYEQFLPKAQKLYLTHIDAEVEGDTHFPDYEPDDW
template_upload.1.A   EAIAACGDVPEIMVIGGGRVYEQFLPKAQKLYLTHIDAEVEGDTHFPDYEPDDW
```

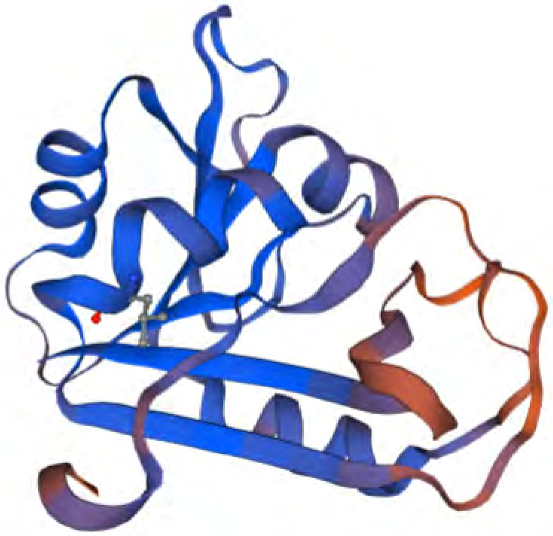

Template: PDB 1rx4A

CP site: Ser135

Target sequence:

```
SVFSEFHDADAQNSHSYCFEILERRGGGGGMISLIAALAVDRVIGMENAMPWNLPADLAW
FKRNTLDKPVIMGRHTWESIGRPLPGRKNIILSSQPGTDDRVTWVKSVDEAIAACGDVPE
IMVIGGGRVYEQFLPKAQKLYLTHIDAEVEGDTHFPDYEPDDWE
```

Model #01	File	Built with	Oligo-State	Ligands	GMQE	QMEAN
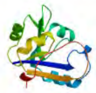	PDB	ProMod3 Version 1.2.0.	monomer	None	0.77	0.34

QMEAN	0.34	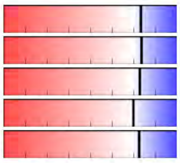
Cβ	0.39	
All Atom	0.30	
Solvation	-0.01	
Torsion	0.25	

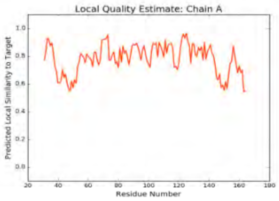

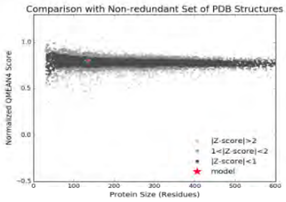

Template	Seq Identity	Oligo-state	Found by	Method	Resolution	Seq Similarity	Range	Coverage	Description
template_upload.1.A	100.00	monomer	HHblits	Unknown	NA	0.62	31 - 164	0.82	Polypeptide

The template contained no ligands.

```
Target                    SVFSEFHDADAQNSHSYCFEILERRGGGGGMISLIAALAVDRVIGMENAMPWNLP
template_upload.1.A       ------------------------------MISLIAALAVDRVIGMENAMPWNLP

Target                    ADLAWFKRNTLDKPVIMGRHTWESIGRPLPGRKNIILSSQPGTDDRVTWVKSVDE
template_upload.1.A       ADLAWFKRNTLDKPVIMGRHTWESIGRPLPGRKNIILSSQPGTDDRVTWVKSVDE

Target                    AIAACGDVPEIMVIGGGRVYEQFLPKAQKLYLTHIDAEVEGDTHFPDYEPDDWE
template_upload.1.A       AIAACGDVPEIMVIGGGRVYEQFLPKAQKLYLTHIDAEVEGDTHFPDYEPDDWE
```

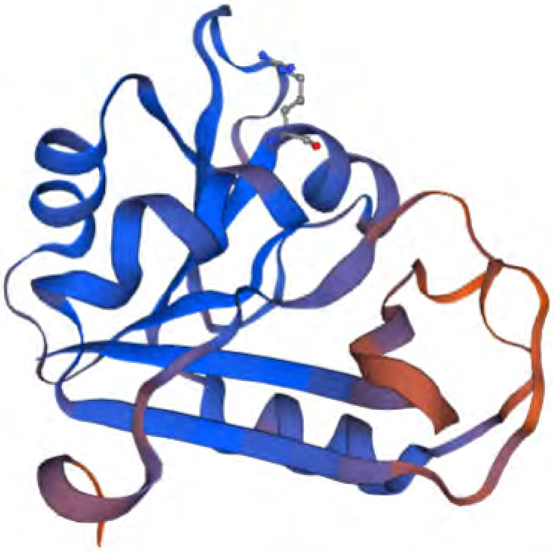

Template: PDB 1rx4A

CP site: Glu139

Target sequence:

EFHDADAQNSHSYCFEILERRGGGGGMISLIAALAVDRVIGMENAMPWNLPADLAWFKRN
TLDKPVIMGRHTWESIGRPLPGRKNIILSSQPGTDDRVTWVKSVDEAIAACGDVPEIMVI
GGGRVYEQFLPKAQKLYLTHIDAEVEGDTHFPDYEPDDWESVFS

Model #01	File	Built with	Oligo-State	Ligands	GMQE	QMEAN
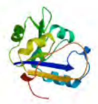	PDB	ProMod3 Version 1.2.0.	monomer	None	0.88	0.17

QMEAN	0.17	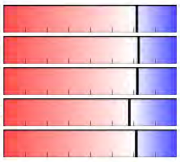
Cβ	0.25	
All Atom	0.19	
Solvation	-0.18	
Torsion	0.14	

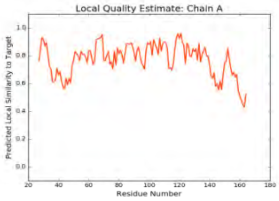

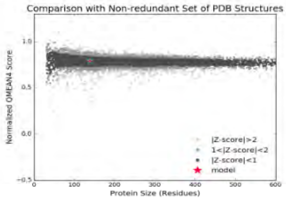

Template	Seq Identity	Oligo-state	Found by	Method	Resolution	Seq Similarity	Range	Coverage	Description
template_upload.1.A	100.00	monomer	BLAST	Unknown	NA	0.62	27 - 164	0.84	Polypeptide

The template contained no ligands.

```
Target                  EFHDADAQNSHSYCFEILERRGGGGGMISLIAALAVDRVIGMENAMPWNLPADLA
template_upload.1.A     --------------------------MISLIAALAVDRVIGMENAMPWNLPADLA

Target                  WFKRNTLDKPVIMGRHTWESIGRPLPGRKNIILSSQPGTDDRVTWVKSVDEAIAA
template_upload.1.A     WFKRNTLDKPVIMGRHTWESIGRPLPGRKNIILSSQPGTDDRVTWVKSVDEAIAA

Target                  CGDVPEIMVIGGGRVYEQFLPKAQKLYLTHIDAEVEGDTHFPDYEPDDWESVFS
template_upload.1.A     CGDVPEIMVIGGGRVYEQFLPKAQKLYLTHIDAEVEGDTHFPDYEPDDWESVFS
```

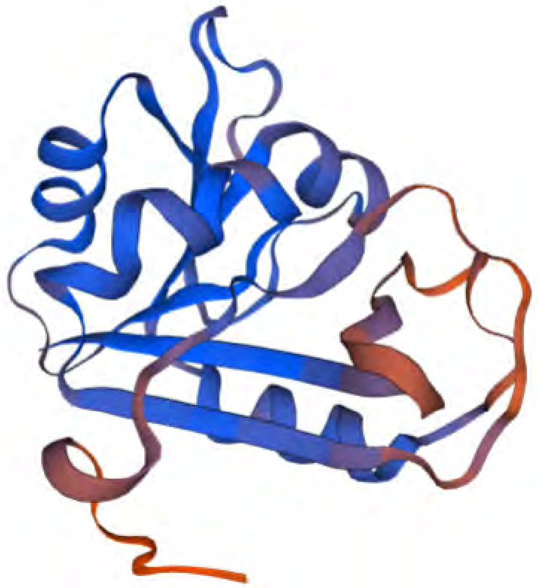

Template: PDB 1rx4A

CP site: Phe140

Target sequence:

```
FHDADAQNSHSYCFEILERRGGGGGMISLIAALAVDRVIGMENAMPWNLPADLAWFKRNT
LDKPVIMGRHTWESIGRPLPGRKNIILSSQPGTDDRVTWVKSVDEAIAACGDVPEIMVIG
GGRVYEQFLPKAQKLYLTHIDAEVEGDTHFPDYEPDDWESVFSE
```

Model #01	File	Built with	Oligo-State	Ligands	GMQE	QMEAN
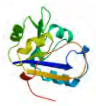	PDB	ProMod3 Version 1.2.0.	monomer	None	0.89	0.18

QMEAN	0.18
Cβ	0.22
All Atom	0.16
Solvation	-0.23
Torsion	0.18

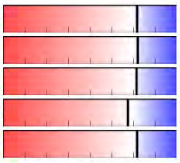

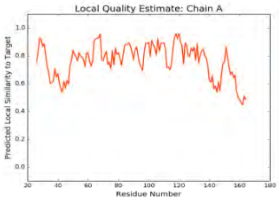

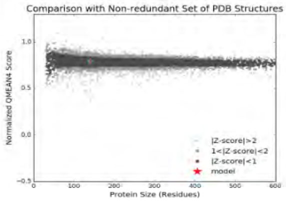

Template	Seq Identity	Oligo-state	Found by	Method	Resolution	Seq Similarity	Range	Coverage	Description
template_upload.1.A	100.00	monomer	BLAST	Unknown	NA	0.62	26 - 164	0.85	Polypeptide

The template contained no ligands.

```
Target                  FHDADAQNSHSYCFEILERRGGGGGMISLIAALAVDRVIGMENAMPWNLPADLAW
template_upload.1.A     -------------------------MISLIAALAVDRVIGMENAMPWNLPADLAW

Target                  FKRNTLDKPVIMGRHTWESIGRPLPGRKNIILSSQPGTDDRVTWVKSVDEAIAAC
template_upload.1.A     FKRNTLDKPVIMGRHTWESIGRPLPGRKNIILSSQPGTDDRVTWVKSVDEAIAAC

Target                  GDVPEIMVIGGGRVYEQFLPKAQKLYLTHIDAEVEGDTHFPDYEPDDWESVFSE
template_upload.1.A     GDVPEIMVIGGGRVYEQFLPKAQKLYLTHIDAEVEGDTHFPDYEPDDWESVFSE
```

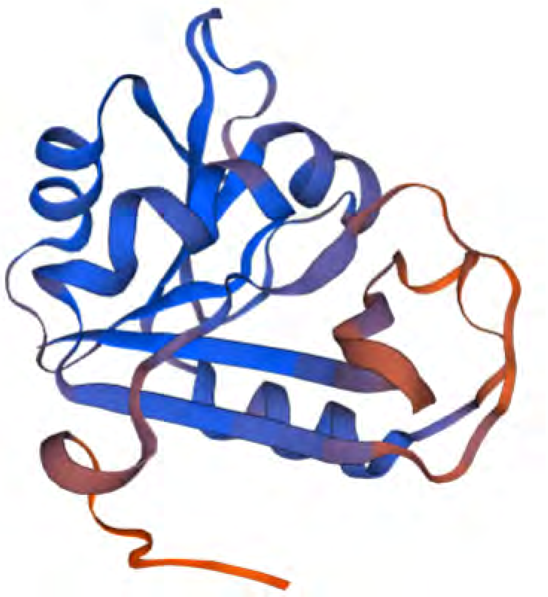

Template: PDB 1rx4A

CP site: His141

Target sequence:

HDADAQNSHSYCFEILERRGGGGGMISLIAALAVDRVIGMENAMPWNLPADLAWFKRNTL
DKPVIMGRHTWESIGRPLPGRKNIILSSQPGTDDRVTWVKSVDEAIAACGDVPEIMVIGG
GRVYEQFLPKAQKLYLTHIDAEVEGDTHFPDYEPDDWESVFSEF

Model #01	File	Built with	Oligo-State	Ligands	GMQE	QMEAN
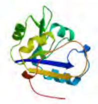	PDB	ProMod3 Version 1.2.0.	monomer	None	0.91	0.10

QMEAN	0.10	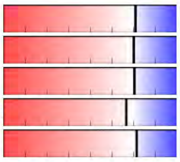
Cβ	0.02	
All Atom	0.04	
Solvation	-0.33	
Torsion	0.16	

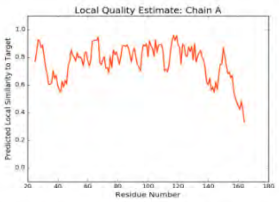

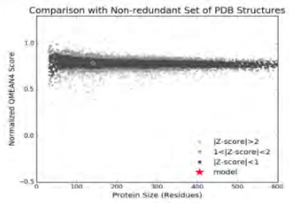

Template	Seq Identity	Oligo-state	Found by	Method	Resolution	Seq Similarity	Range	Coverage	Description
template_upload.1.A	100.00	monomer	BLAST	Unknown	NA	0.62	25 - 164	0.85	Polypeptide

The template contained no ligands.

```
Target                   HDADAQNSHSYCFEILERRGGGGGMISLIAALAVDRVIGMENAMPWNLPADLAWF
template_upload.1.A      ------------------------MISLIAALAVDRVIGMENAMPWNLPADLAWF

Target                   KRNTLDKPVIMGRHTWESIGRPLPGRKNIILSSQPGTDDRVTWVKSVDEAIAACG
template_upload.1.A      KRNTLDKPVIMGRHTWESIGRPLPGRKNIILSSQPGTDDRVTWVKSVDEAIAACG

Target                   DVPEIMVIGGGRVYEQFLPKAQKLYLTHIDAEVEGDTHFPDYEPDDWESVFSEF
template_upload.1.A      DVPEIMVIGGGRVYEQFLPKAQKLYLTHIDAEVEGDTHFPDYEPDDWESVFSEF
```

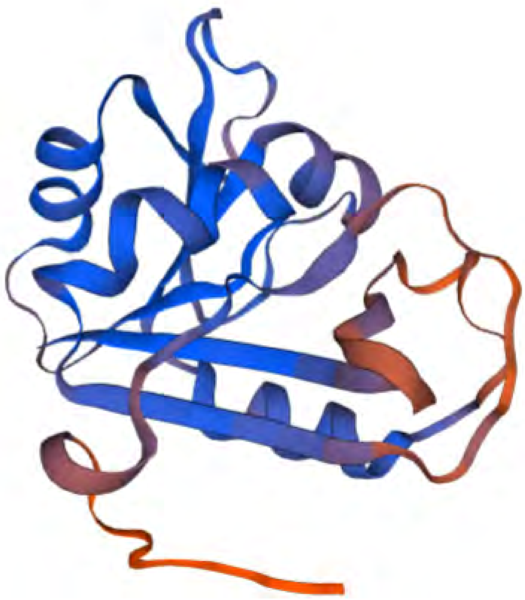

Template: PDB 1rx4A

CP site: Asp142

Target sequence:

```
DADAQNSHSYCFEILERRGGGGGMISLIAALAVDRVIGMENAMPWNLPADLAWFKRNTLD
KPVIMGRHTWESIGRPLPGRKNIILSSQPGTDDRVTWVKSVDEAIAACGDVPEIMVIGGG
RVYEQFLPKAQKLYLTHIDAEVEGDTHFPDYEPDDWESVFSEFH
```

Model #01	File	Built with	Oligo-State	Ligands	GMQE	QMEAN
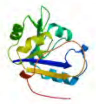	PDB	ProMod3 Version 1.2.0.	monomer	None	0.91	0.04

QMEAN	0.04	
Cβ	0.11	
All Atom	-0.04	
Solvation	-0.30	
Torsion	0.08	

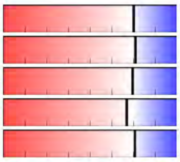

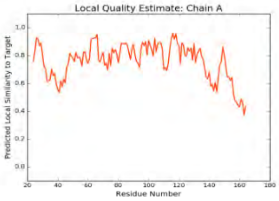

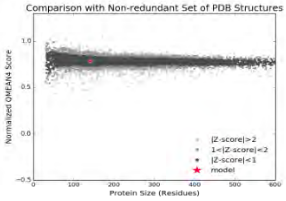

Template	Seq Identity	Oligo-state	Found by	Method	Resolution	Seq Similarity	Range	Coverage	Description
template_upload.1.A	100.00	monomer	BLAST	Unknown	NA	0.62	24 - 164	0.86	Polypeptide

The template contained no ligands.

```
Target                  DADAQNSHSYCFEILERRGGGGGMISLIAALAVDRVIGMENAMPWNLPADLAWFK
template_upload.1.A     -----------------------MISLIAALAVDRVIGMENAMPWNLPADLAWFK

Target                  RNTLDKPVIMGRHTWESIGRPLPGRKNIILSSQPGTDDRVTWVKSVDEAIAACGD
template_upload.1.A     RNTLDKPVIMGRHTWESIGRPLPGRKNIILSSQPGTDDRVTWVKSVDEAIAACGD

Target                  VPEIMVIGGGRVYEQFLPKAQKLYLTHIDAEVEGDTHFPDYEPDDWESVFSEFH
template_upload.1.A     VPEIMVIGGGRVYEQFLPKAQKLYLTHIDAEVEGDTHFPDYEPDDWESVFSEFH
```

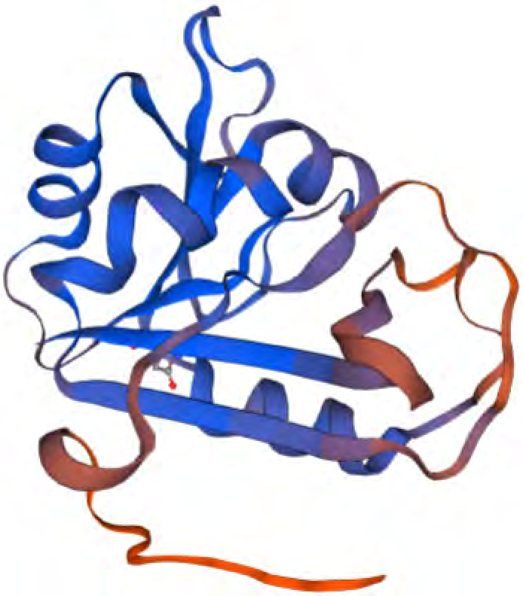

Template: PDB 1rx4A

CP site: Ala143

Target sequence:

```
ADAQNSHSYCFEILERRGGGGGMISLIAALAVDRVIGMENAMPWNLPADLAWFKRNTLDK
PVIMGRHTWESIGRPLPGRKNIILSSQPGTDDRVTWVKSVDEAIAACGDVPEIMVIGGGR
VYEQFLPKAQKLYLTHIDAEVEGDTHFPDYEPDDWESVFSEFHD
```

Model #01	File	Built with	Oligo-State	Ligands	GMQE	QMEAN
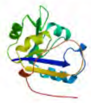	PDB	ProMod3 Version 1.2.0.	monomer	None	0.92	0.03

QMEAN	0.03	
Cβ	0.10	
All Atom	0.02	
Solvation	-0.40	
Torsion	0.08	

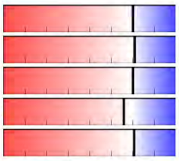

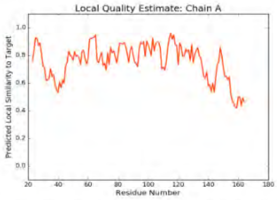

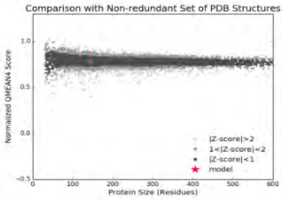

Template	Seq Identity	Oligo-state	Found by	Method	Resolution	Seq Similarity	Range	Coverage	Description
template_upload.1.A	100.00	monomer	BLAST	Unknown	NA	0.62	23 - 164	0.87	Polypeptide

The template contained no ligands.

```
Target                  ADAQNSHSYCFEILERRGGGGGMISLIAALAVDRVIGMENAMPWNLPADLAWFKR
template_upload.1.A     ----------------------MISLIAALAVDRVIGMENAMPWNLPADLAWFKR

Target                  NTLDKPVIMGRHTWESIGRPLPGRKNIILSSQPGTDDRVTWVKSVDEAIAACGDV
template_upload.1.A     NTLDKPVIMGRHTWESIGRPLPGRKNIILSSQPGTDDRVTWVKSVDEAIAACGDV

Target                  PEIMVIGGGRVYEQFLPKAQKLYLTHIDAEVEGDTHFPDYEPDDWESVFSEFHD
template_upload.1.A     PEIMVIGGGRVYEQFLPKAQKLYLTHIDAEVEGDTHFPDYEPDDWESVFSEFHD
```

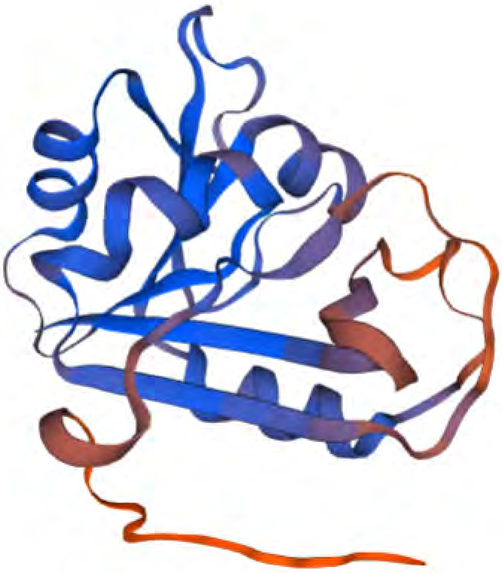

Template: PDB 1rx4A

CP site: Asp144

Target sequence:

DAQNSHSYCFEILERRGGGGGMISLIAALAVDRVIGMENAMPWNLPADLAWFKRNTLDKP
VIMGRHTWESIGRPLPGRKNIILSSQPGTDDRVTWVKSVDEAIAACGDVPEIMVIGGGRV
YEQFLPKAQKLYLTHIDAEVEGDTHFPDYEPDDWESVFSEFHDA

Model #01	File	Built with	Oligo-State	Ligands	GMQE	QMEAN
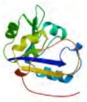	PDB	ProMod3 Version 1.2.0.	monomer	None	0.93	-0.03

QMEAN	-0.03
Cβ	0.02
All Atom	-0.04
Solvation	-0.46
Torsion	0.05

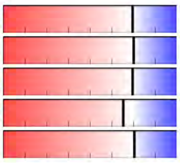

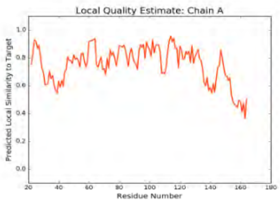

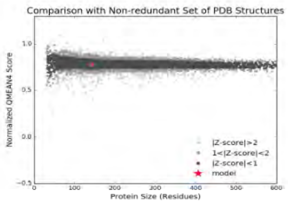

Template	Seq Identity	Oligo-state	Found by	Method	Resolution	Seq Similarity	Range	Coverage	Description
template_upload.1.A	100.00	monomer	BLAST	Unknown	NA	0.62	22 - 164	0.87	Polypeptide

The template contained no ligands.

```
Target                DAQNSHSYCFEILERRGGGGGMISLIAALAVDRVIGMENAMPWNLPADLAWFKRN
template_upload.1.A   ---------------------MISLIAALAVDRVIGMENAMPWNLPADLAWFKRN

Target                TLDKPVIMGRHTWESIGRPLPGRKNIILSSQPGTDDRVTWVKSVDEAIAACGDVP
template_upload.1.A   TLDKPVIMGRHTWESIGRPLPGRKNIILSSQPGTDDRVTWVKSVDEAIAACGDVP

Target                EIMVIGGGRVYEQFLPKAQKLYLTHIDAEVEGDTHFPDYEPDDWESVFSEFHDA
template_upload.1.A   EIMVIGGGRVYEQFLPKAQKLYLTHIDAEVEGDTHFPDYEPDDWESVFSEFHDA
```

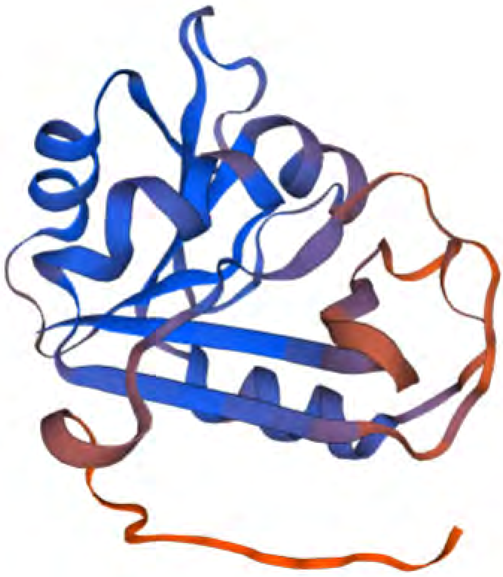

Template: PDB 1rx4A

CP site: Ala145

Target sequence:

AQNSHSYCFEILERRGGGGGMISLIAALAVDRVIGMENAMPWNLPADLAWFKRNTLDKPV
IMGRHTWESIGRPLPGRKNIILSSQPGTDDRVTWVKSVDEAIAACGDVPEIMVIGGGRVY
EQFLPKAQKLYLTHIDAEVEGDTHFPDYEPDDWESVFSEFHDAD

Model #01	File	Built with	Oligo-State	Ligands	GMQE	QMEAN
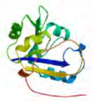	PDB	ProMod3 Version 1.2.0.	monomer	None	0.94	-0.02

QMEAN	-0.02	
Cβ	0.02	
All Atom	0.06	
Solvation	-0.44	
Torsion	0.05	

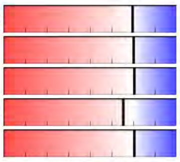

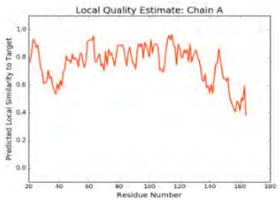

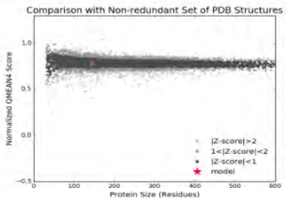

Template	Seq Identity	Oligo-state	Found by	Method	Resolution	Seq Similarity	Range	Coverage	Description
template_upload.1.A	100.00	monomer	BLAST	Unknown	NA	0.62	21 - 164	0.88	Polypeptide

The template contained no ligands.

```
Target                AQNSHSYCFEILERRGGGGGMISLIAALAVDRVIGMENAMPWNLPADLAWFKRNT
template_upload.1.A   --------------------MISLIAALAVDRVIGMENAMPWNLPADLAWFKRNT

Target                LDKPVIMGRHTWESIGRPLPGRKNIILSSQPGTDDRVTWVKSVDEAIAACGDVPE
template_upload.1.A   LDKPVIMGRHTWESIGRPLPGRKNIILSSQPGTDDRVTWVKSVDEAIAACGDVPE

Target                IMVIGGGRVYEQFLPKAQKLYLTHIDAEVEGDTHFPDYEPDDWESVFSEFHDAD
template_upload.1.A   IMVIGGGRVYEQFLPKAQKLYLTHIDAEVEGDTHFPDYEPDDWESVFSEFHDAD
```

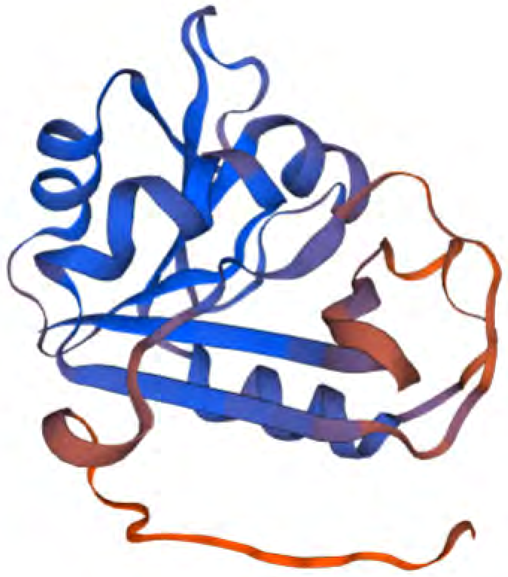

Template: PDB 1rx4A

CP site: Gln146

Target sequence:

QNSHSYCFEILERRGGGGGMISLIAALAVDRVIGMENAMPWNLPADLAWFKRNTLDKPVI
MGRHTWESIGRPLPGRKNIILSSQPGTDDRVTWVKSVDEAIAACGDVPEIMVIGGGRVYE
QFLPKAQKLYLTHIDAEVEGDTHFPDYEPDDWESVFSEFHDADA

Model #01	File	Built with	Oligo-State	Ligands	GMQE	QMEAN
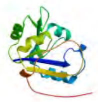	PDB	ProMod3 Version 1.2.0.	monomer	None	0.96	0.17

QMEAN	0.17	
Cβ	-0.14	
All Atom	-0.01	
Solvation	-0.49	
Torsion	0.29	

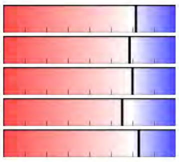

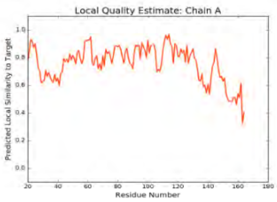

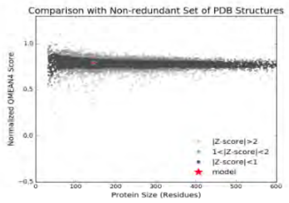

Template	Seq Identity	Oligo-state	Found by	Method	Resolution	Seq Similarity	Range	Coverage	Description
template_upload.1.A	100.00	monomer	BLAST	Unknown	NA	0.62	20 - 164	0.88	Polypeptide

The template contained no ligands.

```
Target                   QNSHSYCFEILERRGGGGGMISLIAALAVDRVIGMENAMPWNLPADLAWFKRNTL
template_upload.1.A      -------------------MISLIAALAVDRVIGMENAMPWNLPADLAWFKRNTL

Target                   DKPVIMGRHTWESIGRPLPGRKNIILSSQPGTDDRVTWVKSVDEAIAACGDVPEI
template_upload.1.A      DKPVIMGRHTWESIGRPLPGRKNIILSSQPGTDDRVTWVKSVDEAIAACGDVPEI

Target                   MVIGGGRVYEQFLPKAQKLYLTHIDAEVEGDTHFPDYEPDDWESVFSEFHDADA
template_upload.1.A      MVIGGGRVYEQFLPKAQKLYLTHIDAEVEGDTHFPDYEPDDWESVFSEFHDADA
```

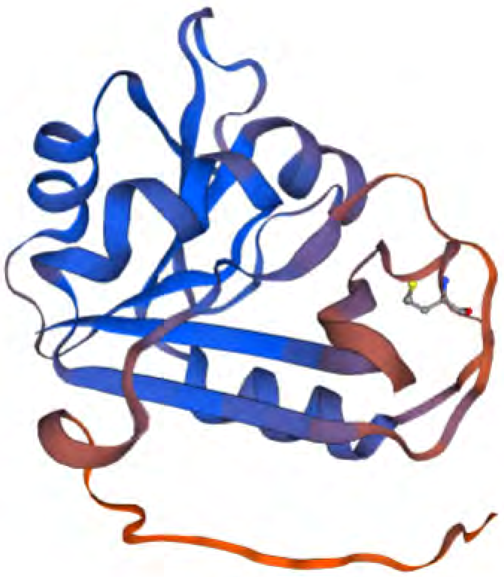

Template: PDB 1rx4A

CP site: Asn147

Target sequence:

```
NSHSYCFEILERRGGGGGMISLIAALAVDRVIGMENAMPWNLPADLAWFKRNTLDKPVIM
GRHTWESIGRPLPGRKNIILSSQPGTDDRVTWVKSVDEAIAACGDVPEIMVIGGGRVYEQ
FLPKAQKLYLTHIDAEVEGDTHFPDYEPDDWESVFSEFHDADAQ
```

Model #01	File	Built with	Oligo-State	Ligands	GMQE	QMEAN
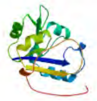	PDB	ProMod3 Version 1.2.0.	monomer	None	0.96	-0.07

QMEAN	-0.07	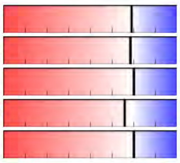
Cβ	-0.14	
All Atom	0.02	
Solvation	-0.39	
Torsion	0.02	

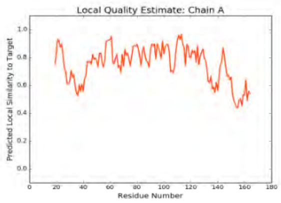

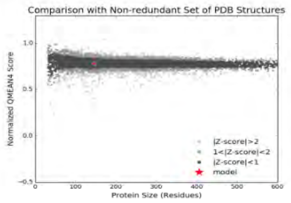

Template	Seq Identity	Oligo-state	Found by	Method	Resolution	Seq Similarity	Range	Coverage	Description
template_upload.1.A	100.00	monomer	BLAST	Unknown	NA	0.62	19 - 164	0.89	Polypeptide

The template contained no ligands.

```
Target               NSHSYCFEILERRGGGGGMISLIAALAVDRVIGMENAMPWNLPADLAWFKRNTLD
template_upload.1.A  ------------------MISLIAALAVDRVIGMENAMPWNLPADLAWFKRNTLD

Target               KPVIMGRHTWESIGRPLPGRKNIILSSQPGTDDRVTWVKSVDEAIAACGDVPEIM
template_upload.1.A  KPVIMGRHTWESIGRPLPGRKNIILSSQPGTDDRVTWVKSVDEAIAACGDVPEIM

Target               VIGGGRVYEQFLPKAQKLYLTHIDAEVEGDTHFPDYEPDDWESVFSEFHDADAQ
template_upload.1.A  VIGGGRVYEQFLPKAQKLYLTHIDAEVEGDTHFPDYEPDDWESVFSEFHDADAQ
```

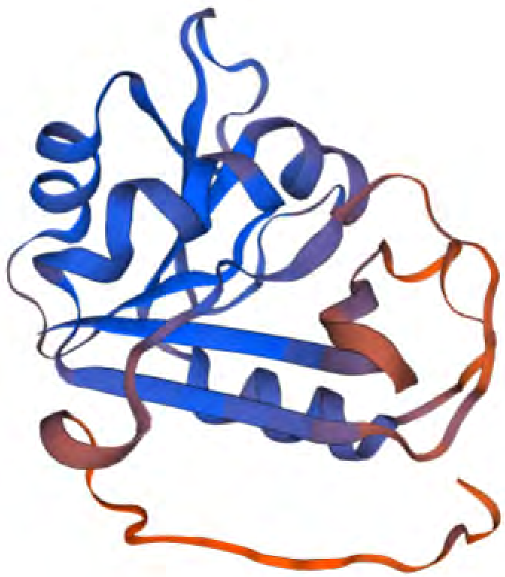

Template: PDB 1rx4A

CP site: Ser148

Target sequence:

```
SHSYCFEILERRGGGGGMISLIAALAVDRVIGMENAMPWNLPADLAWFKRNTLDKPVIMG
RHTWESIGRPLPGRKNIILSSQPGTDDRVTWVKSVDEAIAACGDVPEIMVIGGGRVYEQF
LPKAQKLYLTHIDAEVEGDTHFPDYEPDDWESVFSEFHDADAQN
```

Model #01	File	Built with	Oligo-State	Ligands	GMQE	QMEAN
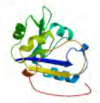	PDB	ProMod3 Version 1.2.0.	monomer	None	0.97	-0.06

QMEAN -0.06
Cβ -0.32
All Atom -0.03
Solvation -0.31
Torsion 0.06

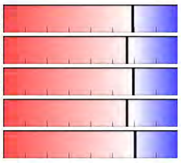

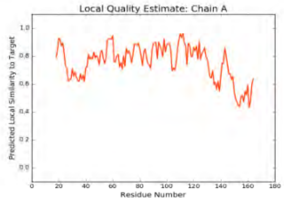

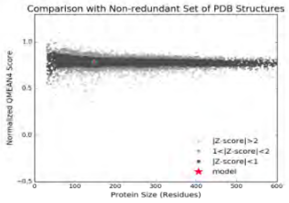

Template	Seq Identity	Oligo-state	Found by	Method	Resolution	Seq Similarity	Range	Coverage	Description
template_upload.1.A	100.00	monomer	BLAST	Unknown	NA	0.62	18 - 164	0.90	Polypeptide

The template contained no ligands.

```
Target                   SHSYCFEILERRGGGGGMISLIAALAVDRVIGMENAMPWNLPADLAWFKRNTLDK
template_upload.1.A      -----------------MISLIAALAVDRVIGMENAMPWNLPADLAWFKRNTLDK

Target                   PVIMGRHTWESIGRPLPGRKNIILSSQPGTDDRVTWVKSVDEAIAACGDVPEIMV
template_upload.1.A      PVIMGRHTWESIGRPLPGRKNIILSSQPGTDDRVTWVKSVDEAIAACGDVPEIMV

Target                   IGGGRVYEQFLPKAQKLYLTHIDAEVEGDTHFPDYEPDDWESVFSEFHDADAQN
template_upload.1.A      IGGGRVYEQFLPKAQKLYLTHIDAEVEGDTHFPDYEPDDWESVFSEFHDADAQN
```

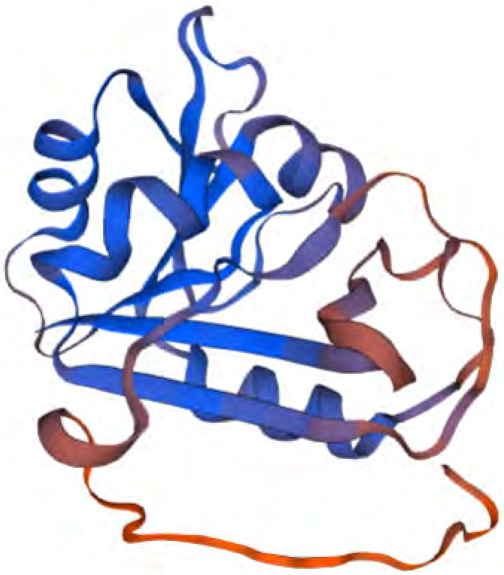

Template: PDB 1rx4A

CP site: His149

Target sequence:

HSYCFEILERRGGGGGMISLIAALAVDRVIGMENAMPWNLPADLAWFKRNTLDKPVIMGR
HTWESIGRPLPGRKNIILSSQPGTDDRVTWVKSVDEAIAACGDVPEIMVIGGGRVYEQFL
PKAQKLYLTHIDAEVEGDTHFPDYEPDDWESVFSEFHDADAQNS

Model #01	File	Built with	Oligo-State	Ligands	GMQE	QMEAN
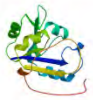	PDB	ProMod3 Version 1.2.0.	monomer	None	0.97	-0.04

QMEAN	-0.04	
Cβ	-0.26	
All Atom	-0.00	
Solvation	-0.36	
Torsion	0.08	

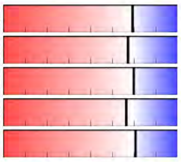

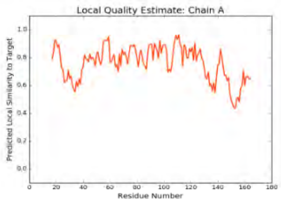

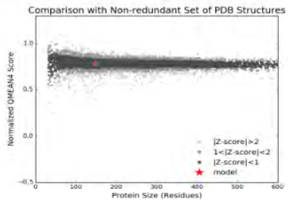

Template	Seq Identity	Oligo-state	Found by	Method	Resolution	Seq Similarity	Range	Coverage	Description
template_upload.1.A	100.00	monomer	BLAST	Unknown	NA	0.62	17 - 164	0.90	Polypeptide

The template contained no ligands.

```
Target                  HSYCFEILERRGGGGGMISLIAALAVDRVIGMENAMPWNLPADLAWFKRNTLDKP
template_upload.1.A     ----------------MISLIAALAVDRVIGMENAMPWNLPADLAWFKRNTLDKP

Target                  VIMGRHTWESIGRPLPGRKNIILSSQPGTDDRVTWVKSVDEAIAACGDVPEIMVI
template_upload.1.A     VIMGRHTWESIGRPLPGRKNIILSSQPGTDDRVTWVKSVDEAIAACGDVPEIMVI

Target                  GGGRVYEQFLPKAQKLYLTHIDAEVEGDTHFPDYEPDDWESVFSEFHDADAQNS
template_upload.1.A     GGGRVYEQFLPKAQKLYLTHIDAEVEGDTHFPDYEPDDWESVFSEFHDADAQNS
```

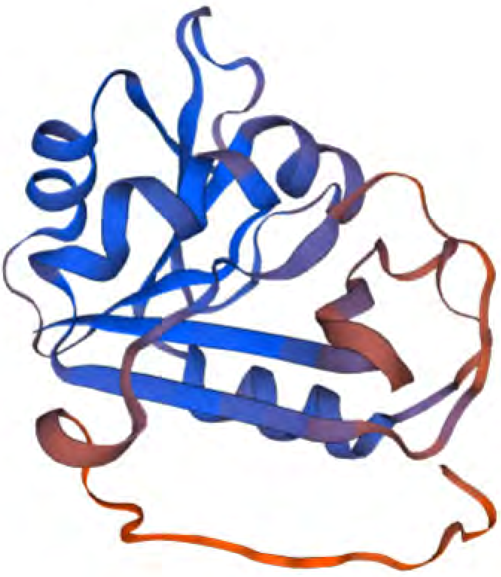

Template: PDB 1rx4A

CP site: Ser150

Target sequence:

SYCFEILERRGGGGGMISLIAALAVDRVIGMENAMPWNLPADLAWFKRNTLDKPVIMGRH
TWESIGRPLPGRKNIILSSQPGTDDRVTWVKSVDEAIAACGDVPEIMVIGGGRVYEQFLP
KAQKLYLTHIDAEVEGDTHFPDYEPDDWESVFSEFHDADAQNSH

Model #01	File	Built with	Oligo-State	Ligands	GMQE	QMEAN
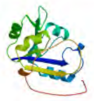	PDB	ProMod3 Version 1.2.0.	monomer	None	0.98	-0.04

QMEAN	-0.04
Cβ	-0.27
All Atom	-0.03
Solvation	-0.32
Torsion	0.07

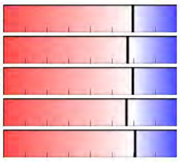

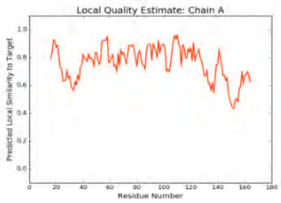

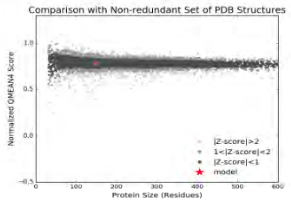

Template	Seq Identity	Oligo-state	Found by	Method	Resolution	Seq Similarity	Range	Coverage	Description
template_upload.1.A	100.00	monomer	BLAST	Unknown	NA	0.62	16 - 164	0.91	Polypeptide

The template contained no ligands.

```
Target                   SYCFEILERRGGGGGMISLIAALAVDRVIGMENAMPWNLPADLAWFKRNTLDKPV
template_upload.1.A      ---------------MISLIAALAVDRVIGMENAMPWNLPADLAWFKRNTLDKPV

Target                   IMGRHTWESIGRPLPGRKNIILSSQPGTDDRVTWVKSVDEAIAACGDVPEIMVIG
template_upload.1.A      IMGRHTWESIGRPLPGRKNIILSSQPGTDDRVTWVKSVDEAIAACGDVPEIMVIG

Target                   GGRVYEQFLPKAQKLYLTHIDAEVEGDTHFPDYEPDDWESVFSEFHDADAQNSH
template_upload.1.A      GGRVYEQFLPKAQKLYLTHIDAEVEGDTHFPDYEPDDWESVFSEFHDADAQNSH
```

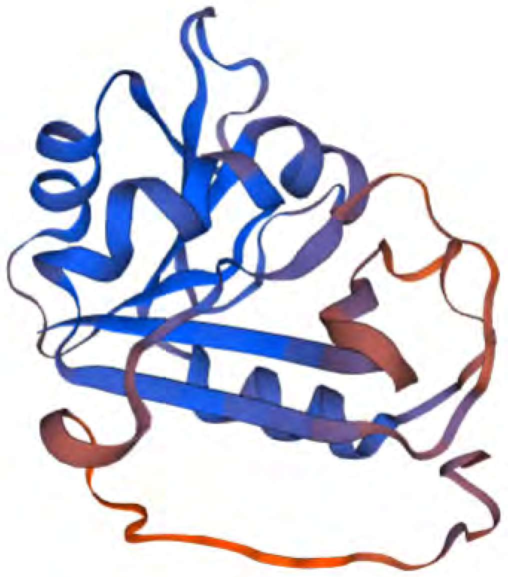

Template: PDB 1rx4A

CP site: Tyr151

Target sequence:

```
YCFEILERRGGGGGMISLIAALAVDRVIGMENAMPWNLPADLAWFKRNTLDKPVIMGRHT
WESIGRPLPGRKNIILSSQPGTDDRVTWVKSVDEAIAACGDVPEIMVIGGGRVYEQFLPK
AQKLYLTHIDAEVEGDTHFPDYEPDDWESVFSEFHDADAQNSHS
```

Model #01	File	Built with	Oligo-State	Ligands	GMQE	QMEAN
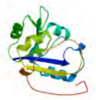	PDB	ProMod3 Version 1.2.0.	monomer	None	0.98	0.01

QMEAN	0.01	
Cβ	-0.08	
All Atom	-0.02	
Solvation	-0.36	
Torsion	0.09	

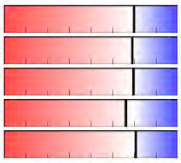

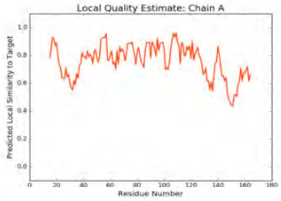

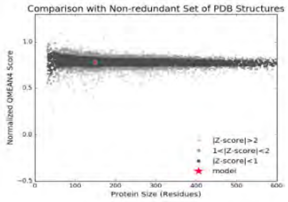

Template	Seq Identity	Oligo-state	Found by	Method	Resolution	Seq Similarity	Range	Coverage	Description
template_upload.1.A	100.00	monomer	BLAST	Unknown	NA	0.62	15 - 164	0.91	Polypeptide

The template contained no ligands.

```
Target                YCFEILERRGGGGGMISLIAALAVDRVIGMENAMPWNLPADLAWFKRNTLDKPVI
template_upload.1.A   --------------MISLIAALAVDRVIGMENAMPWNLPADLAWFKRNTLDKPVI

Target                MGRHTWESIGRPLPGRKNIILSSQPGTDDRVTWVKSVDEAIAACGDVPEIMVIGG
template_upload.1.A   MGRHTWESIGRPLPGRKNIILSSQPGTDDRVTWVKSVDEAIAACGDVPEIMVIGG

Target                GRVYEQFLPKAQKLYLTHIDAEVEGDTHFPDYEPDDWESVFSEFHDADAQNSHS
template_upload.1.A   GRVYEQFLPKAQKLYLTHIDAEVEGDTHFPDYEPDDWESVFSEFHDADAQNSHS
```

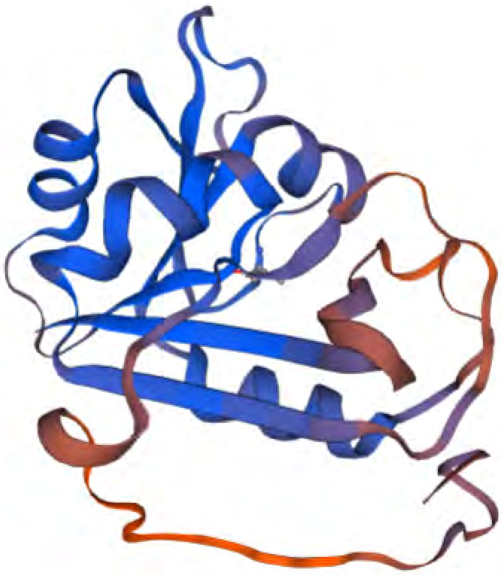

Template: PDB 1rx4A

CP site: Cys152

Target sequence:

CFEILERRGGGGGMISLIAALAVDRVIGMENAMPWNLPADLAWFKRNTLDKPVIMGRHTW
ESIGRPLPGRKNIILSSQPGTDDRVTWVKSVDEAIAACGDVPEIMVIGGGRVYEQFLPKA
QKLYLTHIDAEVEGDTHFPDYEPDDWESVFSEFHDADAQNSHSY

Model #01	File	Built with	Oligo-State	Ligands	GMQE	QMEAN
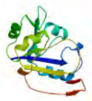	PDB	ProMod3 Version 1.2.0.	monomer	None	0.97	-0.03

QMEAN	-0.03	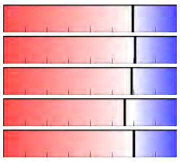
Cβ	0.07	
All Atom	-0.07	
Solvation	-0.39	
Torsion	0.04	

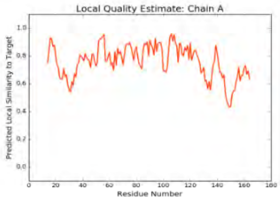

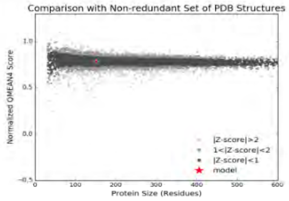

Template	Seq Identity	Oligo-state	Found by	Method	Resolution	Seq Similarity	Range	Coverage	Description
template_upload.1.A	100.00	monomer	HHblits	Unknown	NA	0.62	14 - 164	0.92	Polypeptide

The template contained no ligands.

```
Target                  CFEILERRGGGGGMISLIAALAVDRVIGMENAMPWNLPADLAWFKRNTLDKPVIM
template_upload.1.A     -------------MISLIAALAVDRVIGMENAMPWNLPADLAWFKRNTLDKPVIM

Target                  GRHTWESIGRPLPGRKNIILSSQPGTDDRVTWVKSVDEAIAACGDVPEIMVIGGG
template_upload.1.A     GRHTWESIGRPLPGRKNIILSSQPGTDDRVTWVKSVDEAIAACGDVPEIMVIGGG

Target                  RVYEQFLPKAQKLYLTHIDAEVEGDTHFPDYEPDDWESVFSEFHDADAQNSHSY
template_upload.1.A     RVYEQFLPKAQKLYLTHIDAEVEGDTHFPDYEPDDWESVFSEFHDADAQNSHSY
```

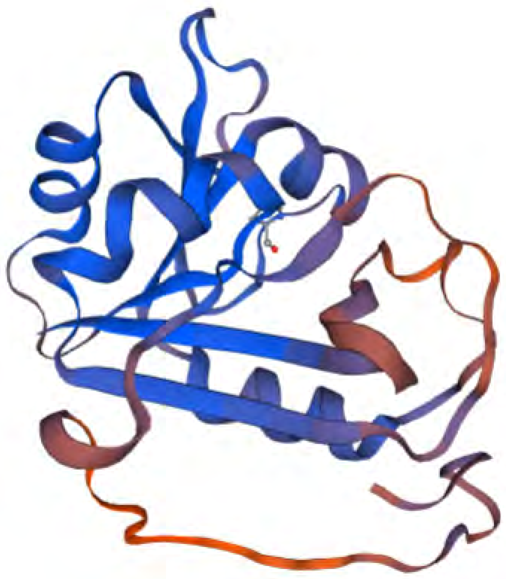

Template: PDB 1rx4A

CP site: Phe153

Target sequence:

FEILERRGGGGGMISLIAALAVDRVIGMENAMPWNLPADLAWFKRNTLDKPVIMGRHTWE
SIGRPLPGRKNIILSSQPGTDDRVTWVKSVDEAIAACGDVPEIMVIGGGRVYEQFLPKAQ
KLYLTHIDAEVEGDTHFPDYEPDDWESVFSEFHDADAQNSHSYC

Model #01	File	Built with	Oligo-State	Ligands	GMQE	QMEAN
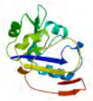	PDB	ProMod3 Version 1.2.0.	monomer	None	0.99	0.02

QMEAN	0.02	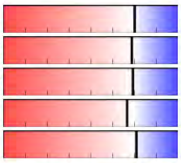
Cβ	-0.10	
All Atom	-0.05	
Solvation	-0.31	
Torsion	0.10	

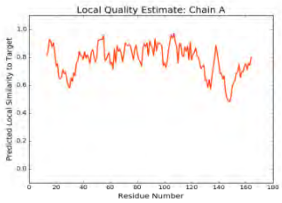

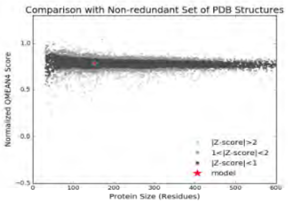

Template	Seq Identity	Oligo-state	Found by	Method	Resolution	Seq Similarity	Range	Coverage	Description
template_upload.1.A	100.00	monomer	BLAST	Unknown	NA	0.62	13 - 164	0.93	Polypeptide

The template contained no ligands.

```
Target                FEILERRGGGGGMISLIAALAVDRVIGMENAMPWNLPADLAWFKRNTLDKPVIMG
template_upload.1.A   ------------MISLIAALAVDRVIGMENAMPWNLPADLAWFKRNTLDKPVIMG

Target                RHTWESIGRPLPGRKNIILSSQPGTDDRVTWVKSVDEAIAACGDVPEIMVIGGGR
template_upload.1.A   RHTWESIGRPLPGRKNIILSSQPGTDDRVTWVKSVDEAIAACGDVPEIMVIGGGR

Target                VYEQFLPKAQKLYLTHIDAEVEGDTHFPDYEPDDWESVFSEFHDADAQNSHSYC
template_upload.1.A   VYEQFLPKAQKLYLTHIDAEVEGDTHFPDYEPDDWESVFSEFHDADAQNSHSYC
```

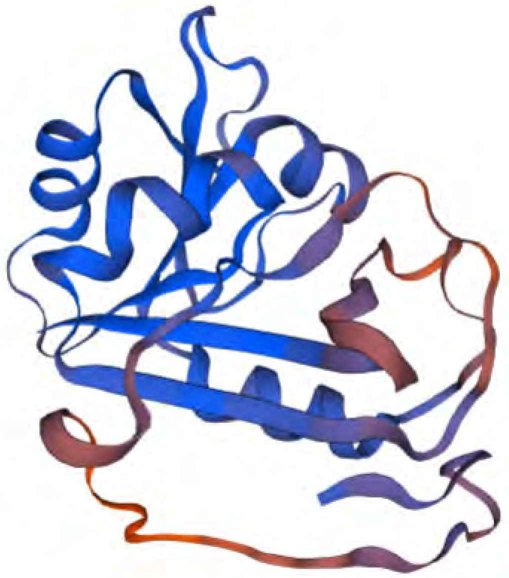

Template: PDB 1rx4A

CP site: Leu156

Target sequence:

LERRGGGGGMISLIAALAVDRVIGMENAMPWNLPADLAWFKRNTLDKPVIMGRHTWESIG
RPLPGRKNIILSSQPGTDDRVTWVKSVDEAIAACGDVPEIMVIGGGRVYEQFLPKAQKLY
LTHIDAEVEGDTHFPDYEPDDWESVFSEFHDADAQNSHSYCFEI

Model #01	File	Built with	Oligo-State	Ligands	GMQE	QMEAN
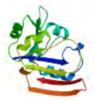	PDB	ProMod3 Version 1.2.0.	monomer	None	0.98	0.06

QMEAN	0.06
Cβ	-0.30
All Atom	0.16
Solvation	-0.21
Torsion	0.15

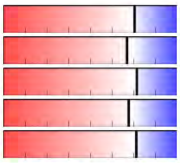

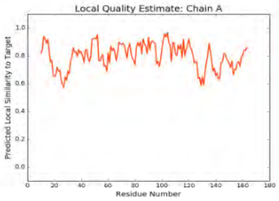

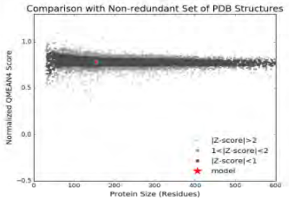

Template	Seq Identity	Oligo-state	Found by	Method	Resolution	Seq Similarity	Range	Coverage	Description
template_upload.1.A	100.00	monomer	HHblits	Unknown	NA	0.62	10 - 164	0.95	Polypeptide

The template contained no ligands.

```
Target                 LERRGGGGGMISLIAALAVDRVIGMENAMPWNLPADLAWFKRNTLDKPVIMGRHT
template_upload.1.A    ---------MISLIAALAVDRVIGMENAMPWNLPADLAWFKRNTLDKPVIMGRHT

Target                 WESIGRPLPGRKNIILSSQPGTDDRVTWVKSVDEAIAACGDVPEIMVIGGGRVYE
template_upload.1.A    WESIGRPLPGRKNIILSSQPGTDDRVTWVKSVDEAIAACGDVPEIMVIGGGRVYE

Target                 QFLPKAQKLYLTHIDAEVEGDTHFPDYEPDDWESVFSEFHDADAQNSHSYCFEI
template_upload.1.A    QFLPKAQKLYLTHIDAEVEGDTHFPDYEPDDWESVFSEFHDADAQNSHSYCFEI
```

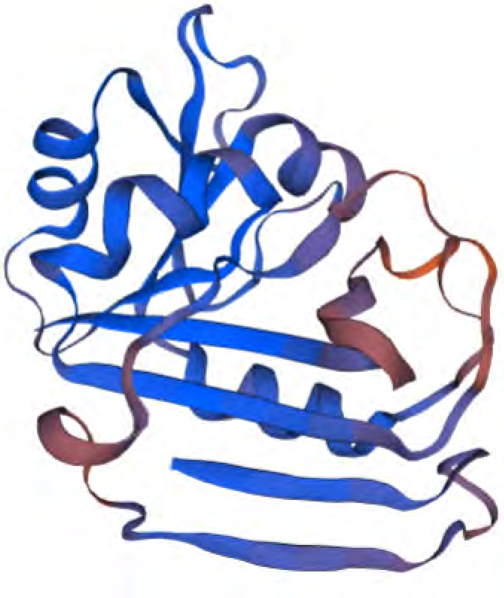

Template: PDB 1rx4A

CP site: Glu157

Target sequence:

```
ERRGGGGGMISLIAALAVDRVIGMENAMPWNLPADLAWFKRNTLDKPVIMGRHTWESIGR
PLPGRKNIILSSQPGTDDRVTWVKSVDEAIAACGDVPEIMVIGGGRVYEQFLPKAQKLYL
THIDAEVEGDTHFPDYEPDDWESVFSEFHDADAQNSHSYCFEIL
```

Model #01	File	Built with	Oligo-State	Ligands	GMQE	QMEAN
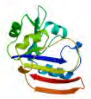	PDB	ProMod3 Version 1.2.0.	monomer	None	0.99	0.11

QMEAN	0.11
Cβ	0.03
All Atom	0.24
Solvation	-0.19
Torsion	0.13

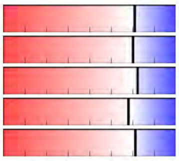

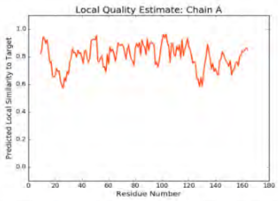

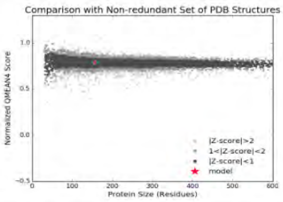

Template	Seq Identity	Oligo-state	Found by	Method	Resolution	Seq Similarity	Range	Coverage	Description
template_upload.1.A	100.00	monomer	HHblits	Unknown	NA	0.62	9 - 164	0.95	Polypeptide

The template contained no ligands.

```
Target                  ERRGGGGGMISLIAALAVDRVIGMENAMPWNLPADLAWFKRNTLDKPVIMGRHTW
template_upload.1.A     --------MISLIAALAVDRVIGMENAMPWNLPADLAWFKRNTLDKPVIMGRHTW

Target                  ESIGRPLPGRKNIILSSQPGTDDRVTWVKSVDEAIAACGDVPEIMVIGGGRVYEQ
template_upload.1.A     ESIGRPLPGRKNIILSSQPGTDDRVTWVKSVDEAIAACGDVPEIMVIGGGRVYEQ

Target                  FLPKAQKLYLTHIDAEVEGDTHFPDYEPDDWESVFSEFHDADAQNSHSYCFEIL
template_upload.1.A     FLPKAQKLYLTHIDAEVEGDTHFPDYEPDDWESVFSEFHDADAQNSHSYCFEIL
```

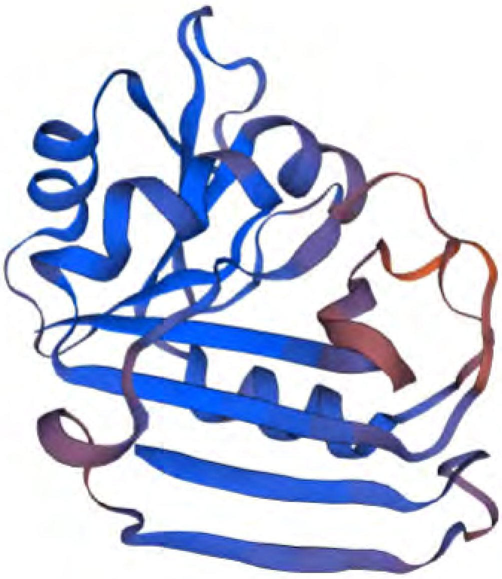

Template: PDB 1rx4A

CP site: Arg158

Target sequence:

RRGGGGGMISLIAALAVDRVIGMENAMPWNLPADLAWFKRNTLDKPVIMGRHTWESIGRP
LPGRKNIILSSQPGTDDRVTWVKSVDEAIAACGDVPEIMVIGGGRVYEQFLPKAQKLYLT
HIDAEVEGDTHFPDYEPDDWESVFSEFHDADAQNSHSYCFEILE

Model #01	File	Built with	Oligo-State	Ligands	GMQE	QMEAN
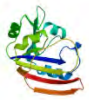	PDB	ProMod3 Version 1.2.0.	monomer	None	0.99	0.05

QMEAN	0.05	
Cβ	-0.18	
All Atom	0.26	
Solvation	-0.14	
Torsion	0.09	

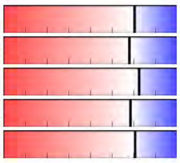

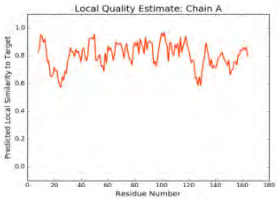

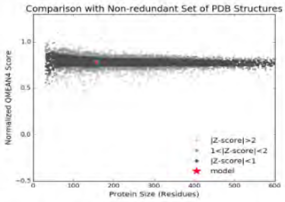

Template	Seq Identity	Oligo-state	Found by	Method	Resolution	Seq Similarity	Range	Coverage	Description
template_upload.1.A	100.00	monomer	HHblits	Unknown	NA	0.62	8 - 164	0.96	Polypeptide

The template contained no ligands.

```
Target                RRGGGGGMISLIAALAVDRVIGMENAMPWNLPADLAWFKRNTLDKPVIMGRHTWE
template_upload.1.A   -------MISLIAALAVDRVIGMENAMPWNLPADLAWFKRNTLDKPVIMGRHTWE

Target                SIGRPLPGRKNIILSSQPGTDDRVTWVKSVDEAIAACGDVPEIMVIGGGRVYEQF
template_upload.1.A   SIGRPLPGRKNIILSSQPGTDDRVTWVKSVDEAIAACGDVPEIMVIGGGRVYEQF

Target                LPKAQKLYLTHIDAEVEGDTHFPDYEPDDWESVFSEFHDADAQNSHSYCFEILE
template_upload.1.A   LPKAQKLYLTHIDAEVEGDTHFPDYEPDDWESVFSEFHDADAQNSHSYCFEILE
```

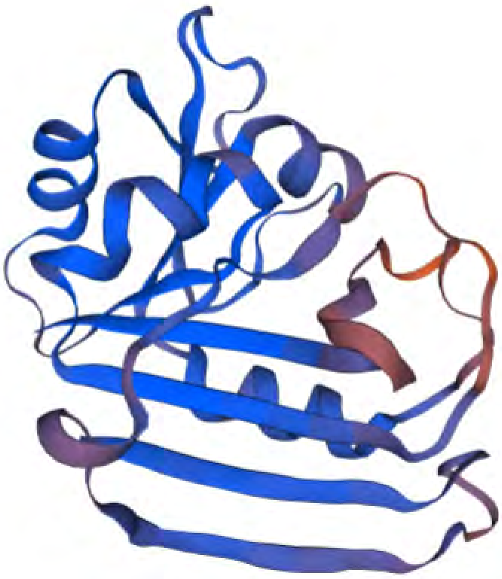

Template: PDB 1rx4A

CP site: Arg159

Target sequence:

```
RGGGGGMISLIAALAVDRVIGMENAMPWNLPADLAWFKRNTLDKPVIMGRHTWESIGRPL
PGRKNIILSSQPGTDDRVTWVKSVDEAIAACGDVPEIMVIGGGRVYEQFLPKAQKLYLTH
IDAEVEGDTHFPDYEPDDWESVFSEFHDADAQNSHSYCFEILER
```

Model #01	File	Built with	Oligo-State	Ligands	GMQE	QMEAN
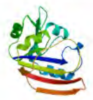	PDB	ProMod3 Version 1.2.0.	monomer	None	0.99	0.02

QMEAN	0.02
Cβ	-0.33
All Atom	0.33
Solvation	-0.09
Torsion	0.08

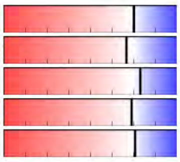

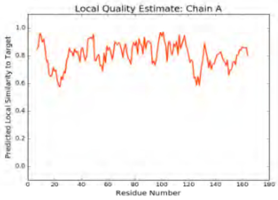

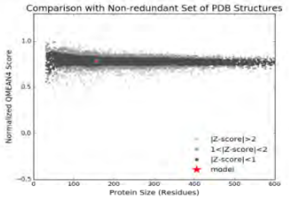

Template	Seq Identity	Oligo-state	Found by	Method	Resolution	Seq Similarity	Range	Coverage	Description
template_upload.1.A	100.00	monomer	HHblits	Unknown	NA	0.62	7 - 164	0.96	Polypeptide

The template contained no ligands.

```
Target                RGGGGGMISLIAALAVDRVIGMENAMPWNLPADLAWFKRNTLDKPVIMGRHTWES
template_upload.1.A   ------MISLIAALAVDRVIGMENAMPWNLPADLAWFKRNTLDKPVIMGRHTWES

Target                IGRPLPGRKNIILSSQPGTDDRVTWVKSVDEAIAACGDVPEIMVIGGGRVYEQFL
template_upload.1.A   IGRPLPGRKNIILSSQPGTDDRVTWVKSVDEAIAACGDVPEIMVIGGGRVYEQFL

Target                PKAQKLYLTHIDAEVEGDTHFPDYEPDDWESVFSEFHDADAQNSHSYCFEILER
template_upload.1.A   PKAQKLYLTHIDAEVEGDTHFPDYEPDDWESVFSEFHDADAQNSHSYCFEILER
```

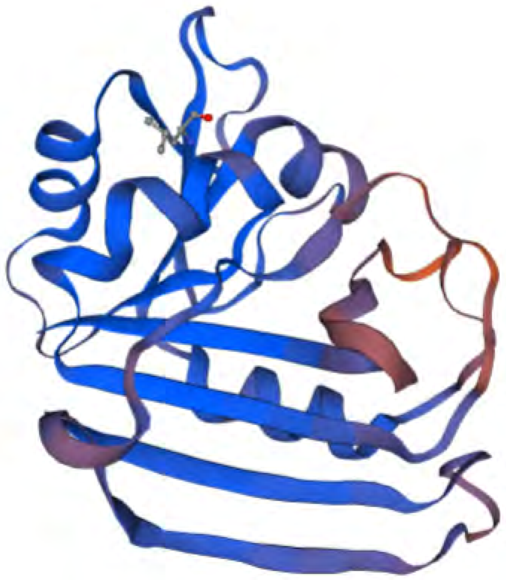

Models constructed by RaptorX for viable circular permutants of the dihydrofolate reductase (PDB:1rx4)

- References

  - RaptorX

    - Benkert P, Biasini M, Schwede T: **Toward the estimation of the absolute quality of individual protein structure models.** *Bioinformatics* 2011, **27:**343-350.
    - Bertoni M, Kiefer F, Biasini M, Bordoli L, Schwede T: **Modeling protein quaternary structure of homo- and hetero-oligomers beyond binary interactions by homology.** *Sci Rep* 2017, **7:**10480.
    - Bienert S, Waterhouse A, de Beer TA, Tauriello G, Studer G, Bordoli L, Schwede T: **The SWISS-MODEL Repository-new features and functionality.** *Nucleic Acids Res* 2017, **45:**D313-D319.
    - Guex N, Peitsch MC, Schwede T: **Automated comparative protein structure modeling with SWISS-MODEL and Swiss-PdbViewer: a historical perspective.** *Electrophoresis* 2009, **30 Suppl 1:**S162-173.
    - Waterhouse A, Bertoni M, Bienert S, Studer G, Tauriello G, Gumienny R, Heer FT, de Beer TAP, Rempfer C, Bordoli L, et al: **SWISS-MODEL: homology modelling of protein structures and complexes.** *Nucleic Acids Res* 2018, **46:**W296-W303.

Template: PDB 1rx4A

CP site: Met1

Target sequence:

MISLIAALAVDRVIGMENAMPWNLPADLAWFKRNTLDKPVIMGRHTWESIGRPLPGRKNI
ILSSQPGTDDRVTWVKSVDEAIAACGDVPEIMVIGGGRVYEQFLPKAQKLYLTHIDAEVE
GDTHFPDYEPDDWESVFSEFHDADAQNSHSYCFEILERRGGGGG

Summary

- The input predicted as **1** domain(s)
- Best template: **1dreA**, p-value **5.21e-10**
- Overall uGDT (GDT): **162** (**99**)
- **164**(**100%**) residues are modeled
- **5**(**3%**) positions predicted as disordered
- Secondary struct: **18%**H, **31%**E, **49%**C
- Solvent access: **33%**E, **35%**M, **31%**B

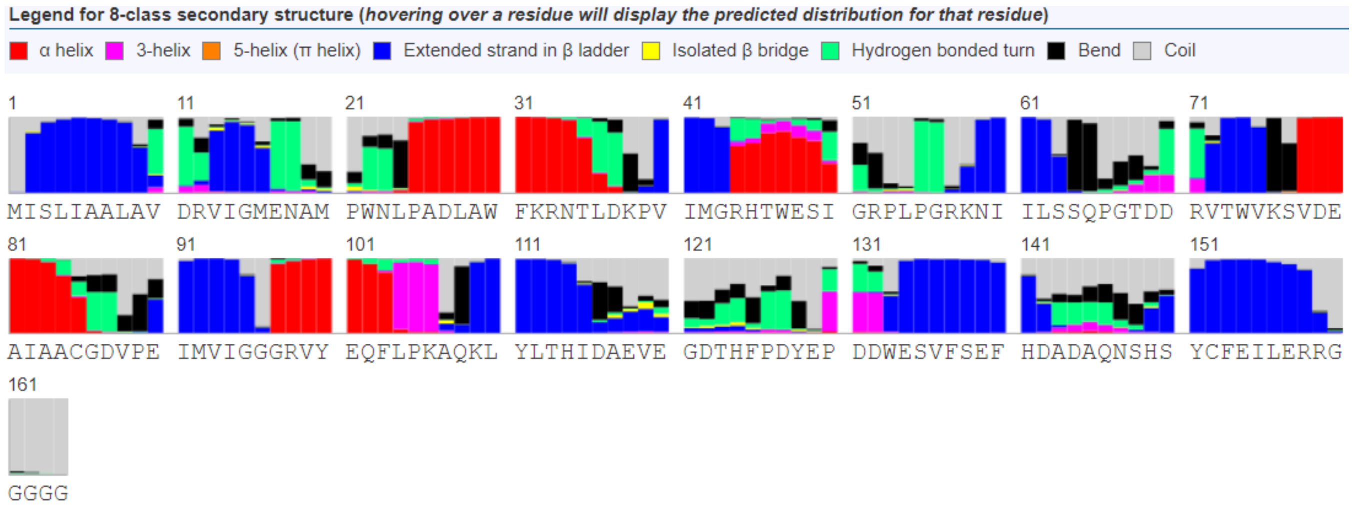

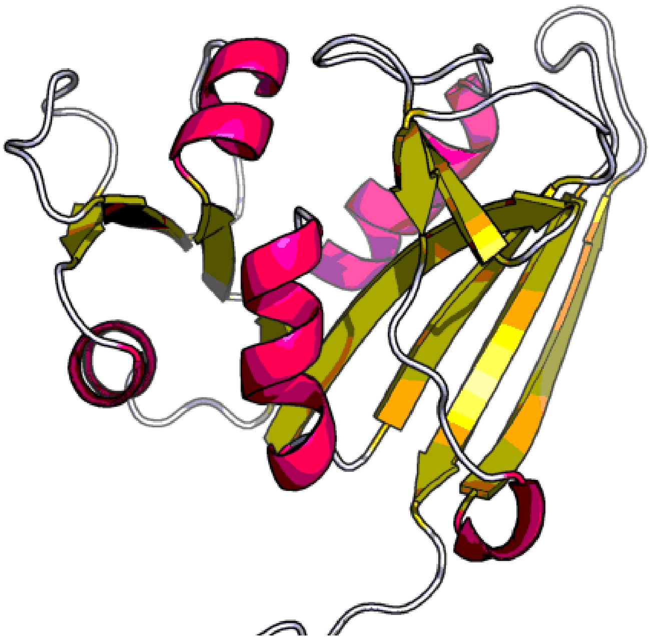

Template: PDB 1rx4A

CP site: Ile2

Target sequence:

ISLIAALAVDRVIGMENAMPWNLPADLAWFKRNTLDKPVIMGRHTWESIGRPLPGRKNII
LSSQPGTDDRVTWVKSVDEAIAACGDVPEIMVIGGGRVYEQFLPKAQKLYLTHIDAEVEG
DTHFPDYEPDDWESVFSEFHDADAQNSHSYCFEILERRGGGGGM

Summary

- The input predicted as **1** domain(s)
- Best template: **1dreA**, p-value **4.06e-10**
- Overall uGDT (GDT): **161** (**98**)
- **164**(**100%**) residues are modeled
- **9**(**5%**) positions predicted as disordered
- Secondary struct: **17%**H, **31%**E, **51%**C
- Solvent access: **35%**E, **33%**M, **30%**B

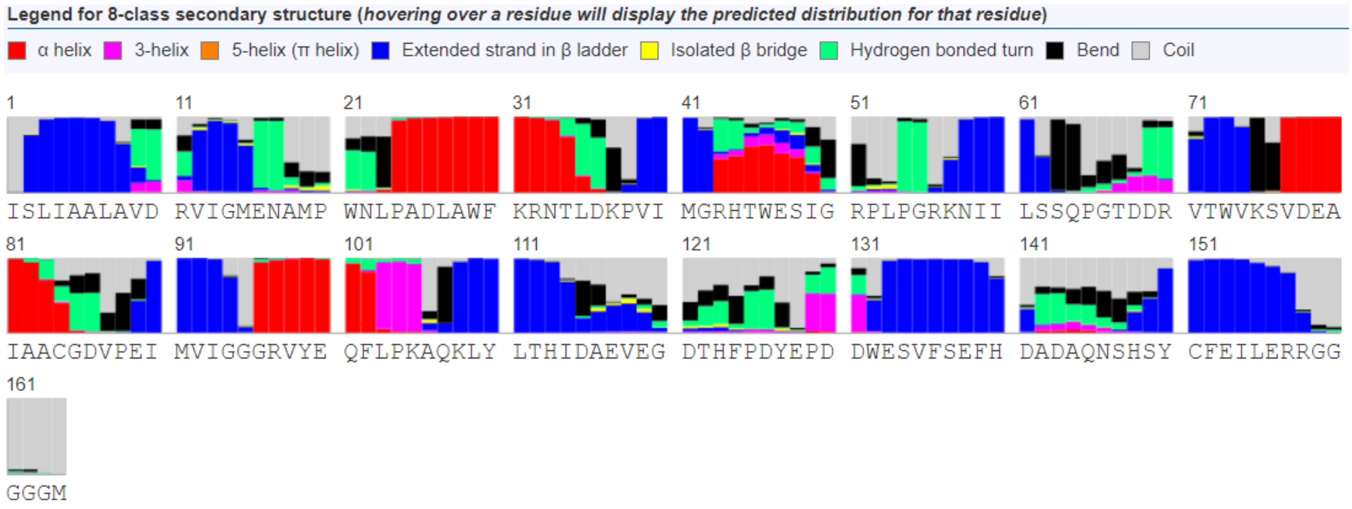

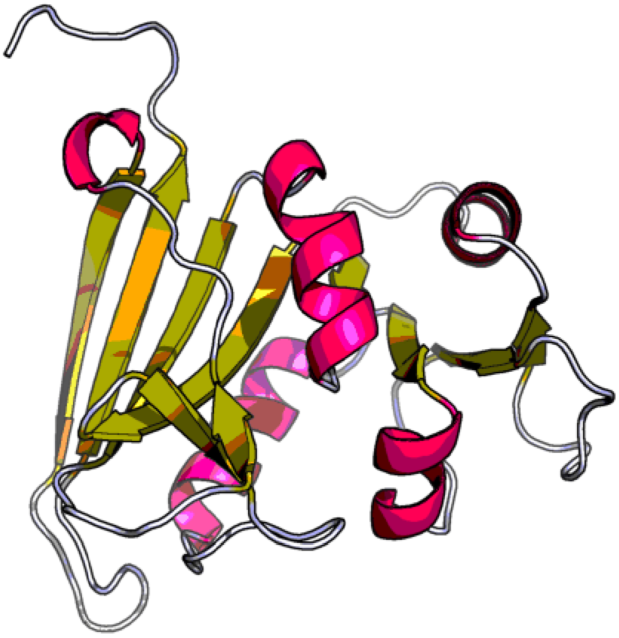

Template: PDB 1rx4A

CP site: Gly15

Target sequence:

GMENAMPWNLPADLAWFKRNTLDKPVIMGRHTWESIGRPLPGRKNIILSSQPGTDDRVTW
VKSVDEAIAACGDVPEIMVIGGGRVYEQFLPKAQKLYLTHIDAEVEGDTHFPDYEPDDWE
SVFSEFHDADAQNSHSYCFEILERRGGGGGMISLIAALAVDRVI

Summary

- The input predicted as **1** domain(s)
- Best template: **1dreA**, p-value **4.06e-09**
- Overall uGDT (GDT): **144** (**88**)
- **164**(**100%**) residues are modeled
- **7**(**4%**) positions predicted as disordered
- Secondary struct: **18%**H, **29%**E, **51%**C
- Solvent access: **37%**E, **34%**M, **28%**B

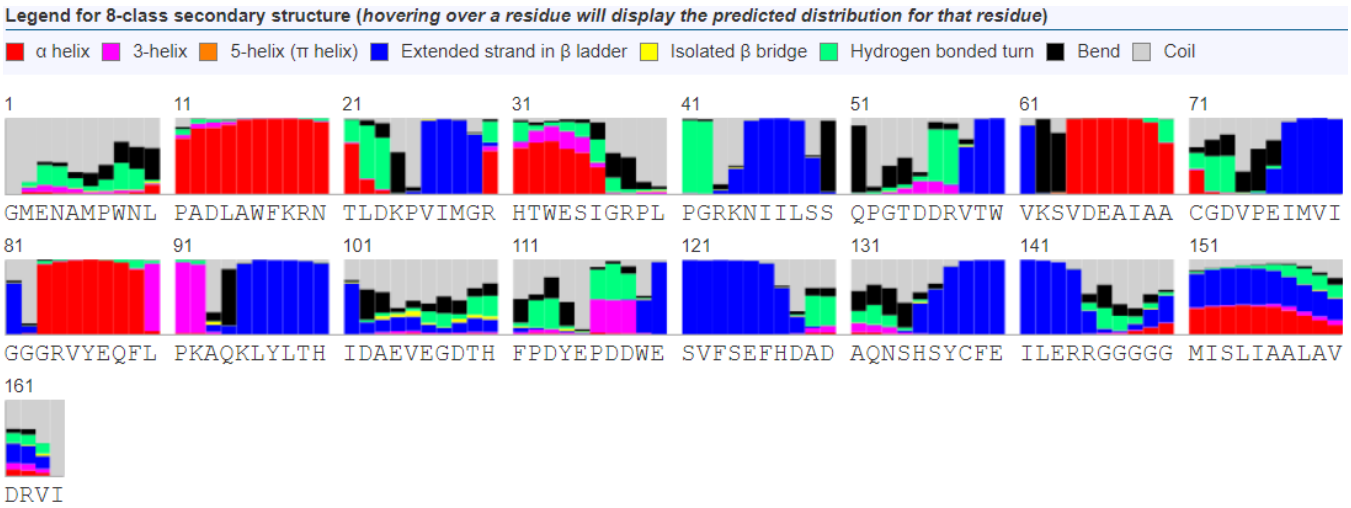

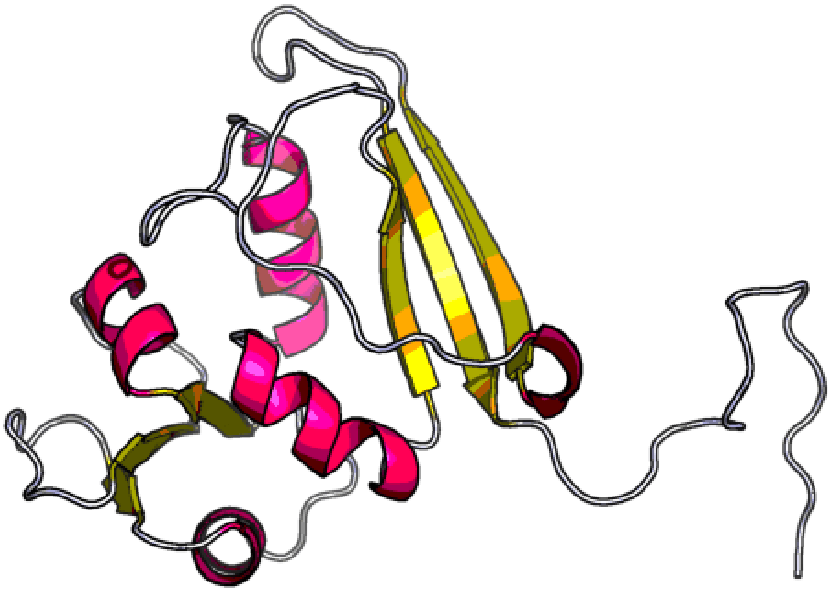

Template: PDB 1rx4A

CP site: Met16

Target sequence:

MENAMPWNLPADLAWFKRNTLDKPVIMGRHTWESIGRPLPGRKNIILSSQPGTDDRVTWV
KSVDEAIAACGDVPEIMVIGGGRVYEQFLPKAQKLYLTHIDAEVEGDTHFPDYEPDDWES
VFSEFHDADAQNSHSYCFEILERRGGGGGMISLIAALAVDRVIG

Summary

- The input predicted as **1** domain(s)
- Best template: **1dreA**, p-value **6.14e-09**
- Overall uGDT (GDT): **144** (**88**)
- **164**(**100%**) residues are modeled
- **9**(**5%**) positions predicted as disordered
- Secondary struct: **18%**H, **29%**E, **51%**C
- Solvent access: **35%**E, **35%**M, **28%**B

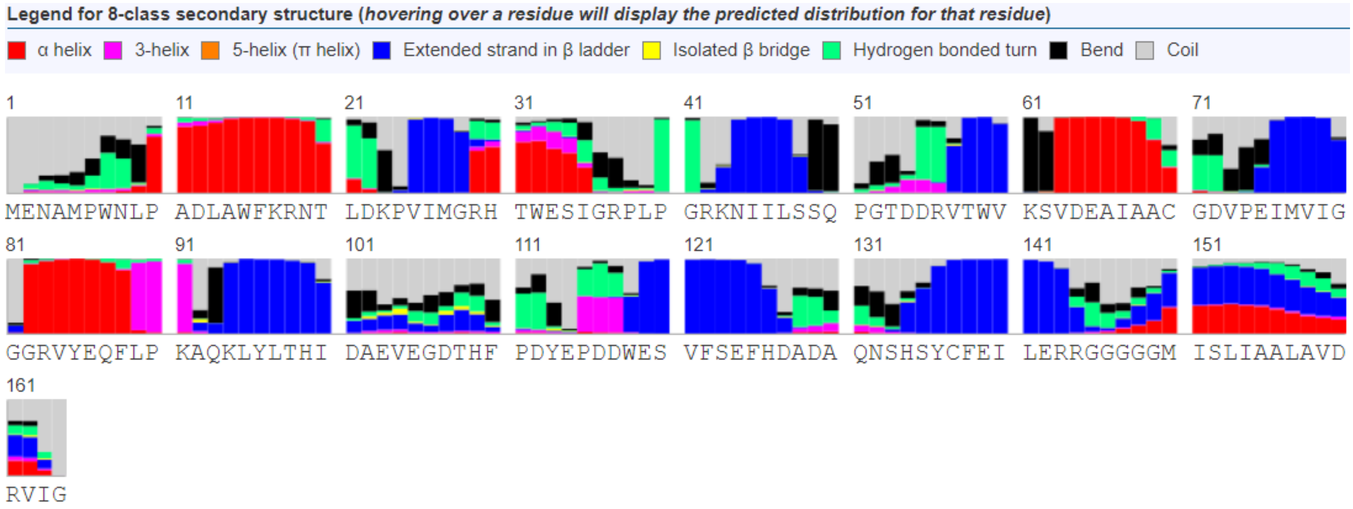

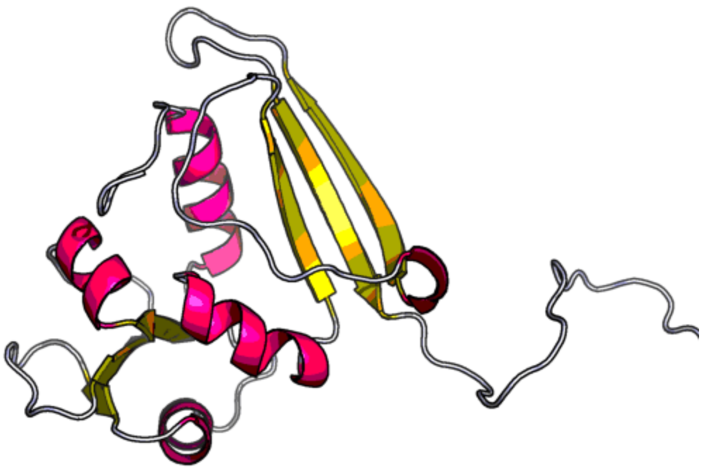

Template: PDB 1rx4A

CP site: Glu17

Target sequence:

```
ENAMPWNLPADLAWFKRNTLDKPVIMGRHTWESIGRPLPGRKNIILSSQPGTDDRVTWVK
SVDEAIAACGDVPEIMVIGGGRVYEQFLPKAQKLYLTHIDAEVEGDTHFPDYEPDDWESV
FSEFHDADAQNSHSYCFEILERRGGGGGMISLIAALAVDRVIGM
```

Summary

- The input predicted as **1** domain(s)
- Best template: **1dreA**, p-value **7.35e-09**
- Overall uGDT (GDT): **142** (**86**)
- **164**(**100%**) residues are modeled
- **6**(**3%**) positions predicted as disordered
- Secondary struct: **27%**H, **25%**E, **46%**C
- Solvent access: **35%**E, **36%**M, **28%**B

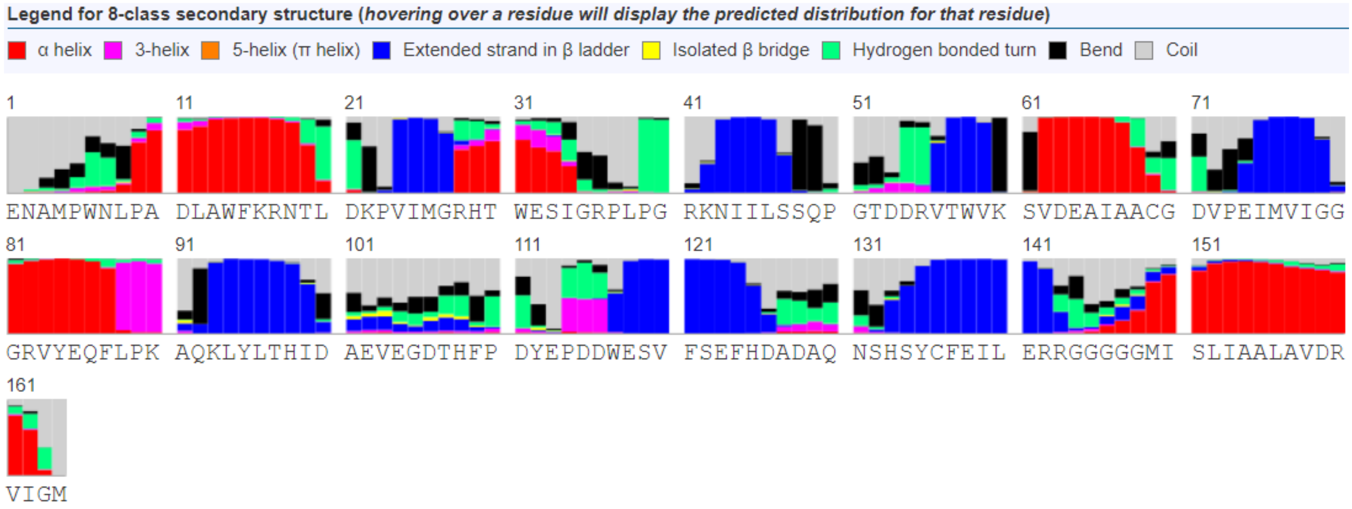

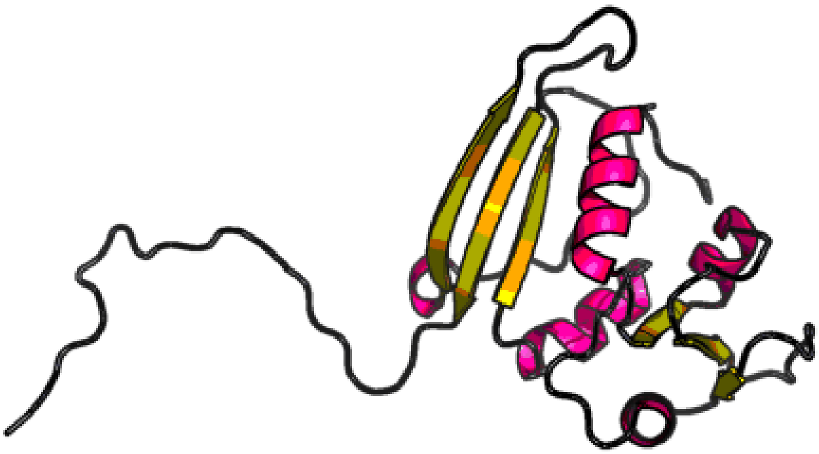

Template: PDB 1rx4A

CP site: Asn18

Target sequence:

NAMPWNLPADLAWFKRNTLDKPVIMGRHTWESIGRPLPGRKNIILSSQPGTDDRVTWVKS
VDEAIAACGDVPEIMVIGGGRVYEQFLPKAQKLYLTHIDAEVEGDTHFPDYEPDDWESVF
SEFHDADAQNSHSYCFEILERRGGGGGMISLIAALAVDRVIGME

Summary

- The input predicted as **1** domain(s)
- Best template: **1dreA**, p-value **4.48e-09**
- Overall uGDT (GDT): **140** (**85**)
- **164**(**100%**) residues are modeled
- **9**(**5%**) positions predicted as disordered
- Secondary struct: **25%**H, **25%**E, **50%**C
- Solvent access: **37%**E, **34%**M, **27%**B

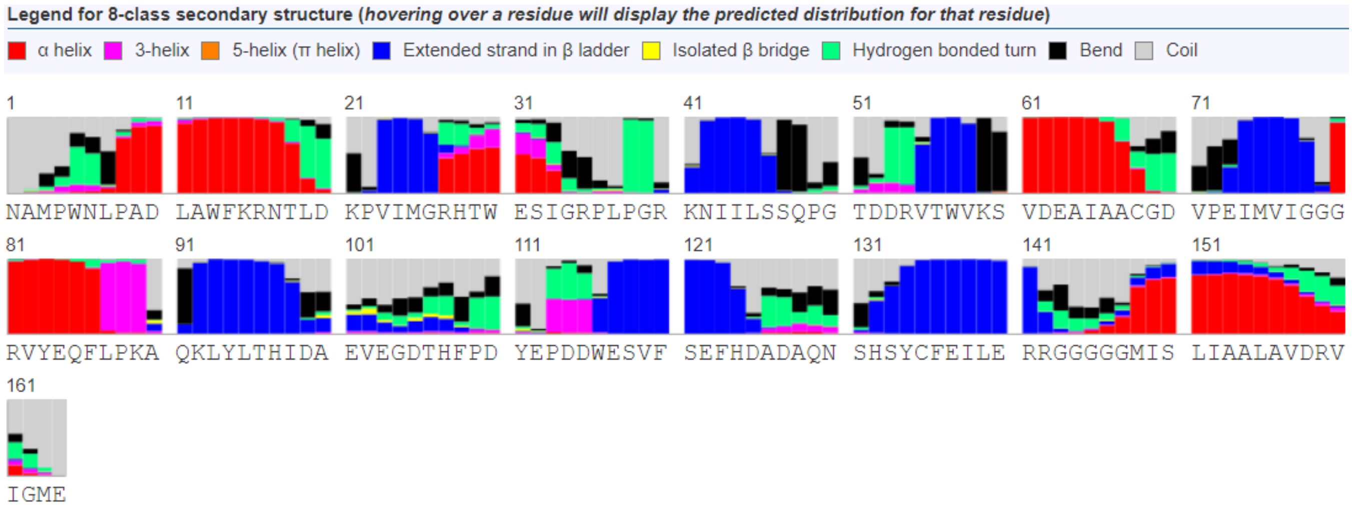

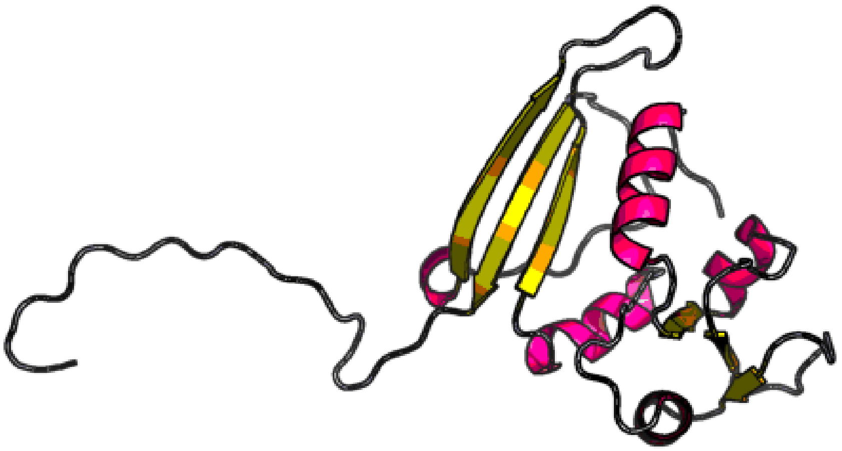

Template: PDB 1rx4A

CP site: Ala19

Target sequence:

AMPWNLPADLAWFKRNTLDKPVIMGRHTWESIGRPLPGRKNIILSSQPGTDDRVTWVKSV
DEAIAACGDVPEIMVIGGGRVYEQFLPKAQKLYLTHIDAEVEGDTHFPDYEPDDWESVFS
EFHDADAQNSHSYCFEILERRGGGGGMISLIAALAVDRVIGMEN

Summary

•The input predicted as **1** domain(s)
•Best template: **1dreA**, p-value **8.03e-09**
•Overall uGDT (GDT): **139** (**85**)
•**164**(**100%**) residues are modeled
•**9**(**5%**) positions predicted as disordered
•Secondary struct: **25%**H, **25%**E, **48%**C
•Solvent access: **37%**E, **35%**M, **26%**B

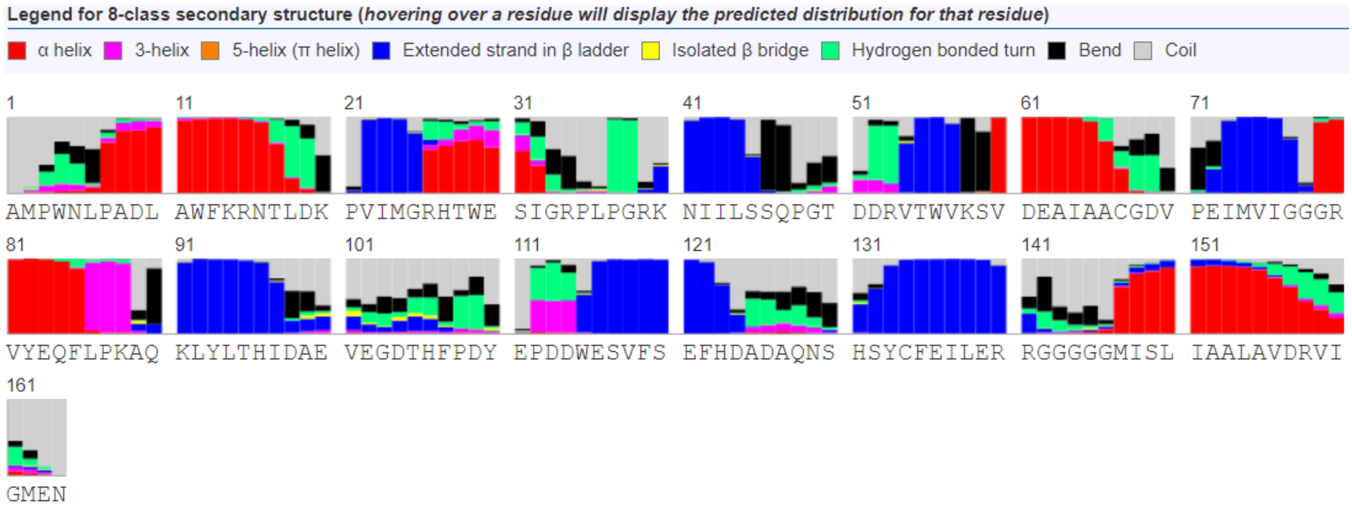

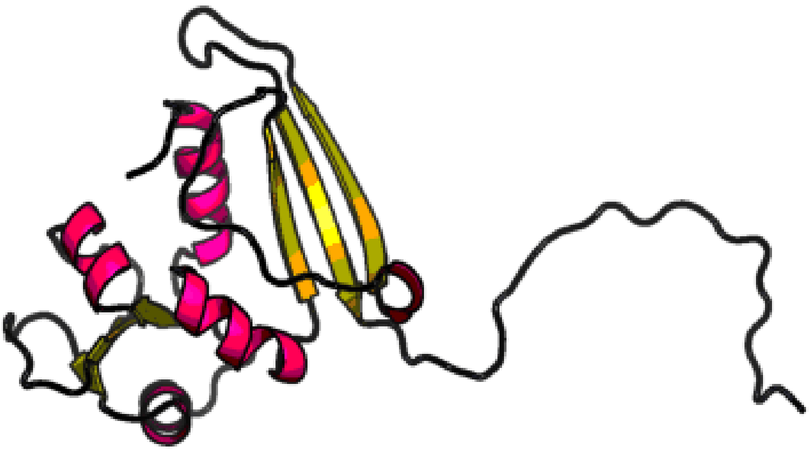

Template: PDB 1rx4A

CP site: Met20

Target sequence:

MPWNLPADLAWFKRNTLDKPVIMGRHTWESIGRPLPGRKNIILSSQPGTDDRVTWVKSVD
EAIAACGDVPEIMVIGGGRVYEQFLPKAQKLYLTHIDAEVEGDTHFPDYEPDDWESVFSE
FHDADAQNSHSYCFEILERRGGGGGMISLIAALAVDRVIGMENA

Summary

- The input predicted as **1** domain(s)
- Best template: **1dreA**, p-value **1.25e-08**
- Overall uGDT (GDT): **137** (**83**)
- **164**(**100%**) residues are modeled
- **10**(**6%**) positions predicted as disordered
- Secondary struct: **26%**H, **25%**E, **48%**C
- Solvent access: **37%**E, **34%**M, **28%**B

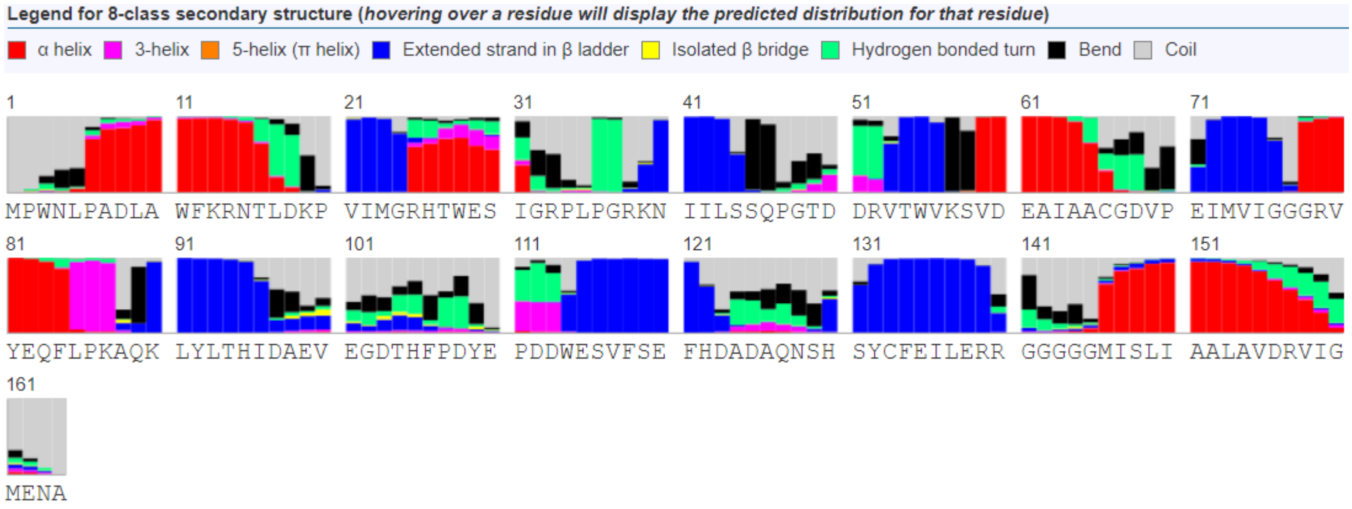

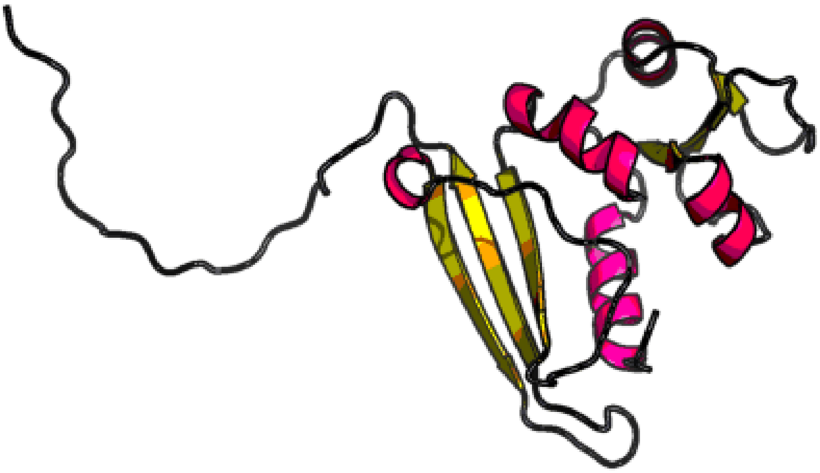

Template: PDB 1rx4A

CP site: Pro21

Target sequence:

PWNLPADLAWFKRNTLDKPVIMGRHTWESIGRPLPGRKNIILSSQPGTDDRVTWVKSVDE
AIAACGDVPEIMVIGGGRVYEQFLPKAQKLYLTHIDAEVEGDTHFPDYEPDDWESVFSEF
HDADAQNSHSYCFEILERRGGGGGMISLIAALAVDRVIGMENAM

Summary

- The input predicted as **1** domain(s)
- Best template: **1dreA**, p-value **8.43e-09**
- Overall uGDT (GDT): **136** (**83**)
- **164**(**100%**) residues are modeled
- **7**(**4%**) positions predicted as disordered
- Secondary struct: **26%**H, **25%**E, **48%**C
- Solvent access: **37%**E, **35%**M, **26%**B

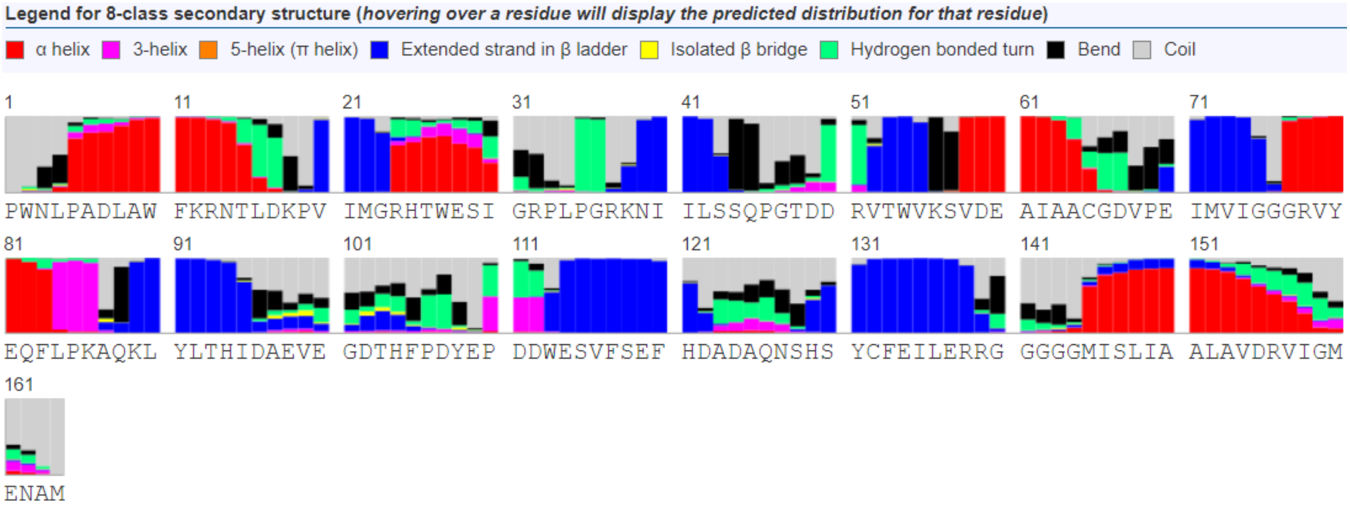

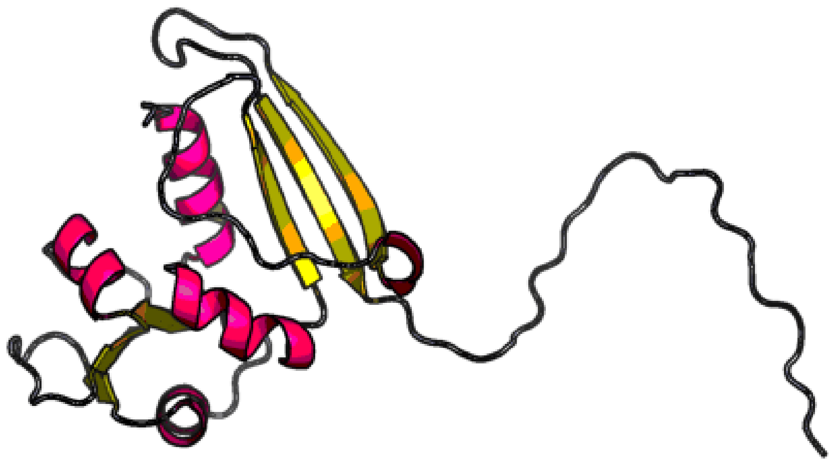

Template: PDB 1rx4A

CP site: Trp22

Target sequence:

WNLPADLAWFKRNTLDKPVIMGRHTWESIGRPLPGRKNIILSSQPGTDDRVTWVKSVDEA
IAACGDVPEIMVIGGGRVYEQFLPKAQKLYLTHIDAEVEGDTHFPDYEPDDWESVFSEFH
DADAQNSHSYCFEILERRGGGGGMISLIAALAVDRVIGMENAMP

Summary

- The input predicted as **1** domain(s)
- Best template: **1dreA**, p-value **8.78e-09**
- Overall uGDT (GDT): **137** (**83**)
- **164**(**100%**) residues are modeled
- **15**(**9%**) positions predicted as disordered
- Secondary struct: **23%**H, **25%**E, **51%**C
- Solvent access: **38%**E, **34%**M, **26%**B

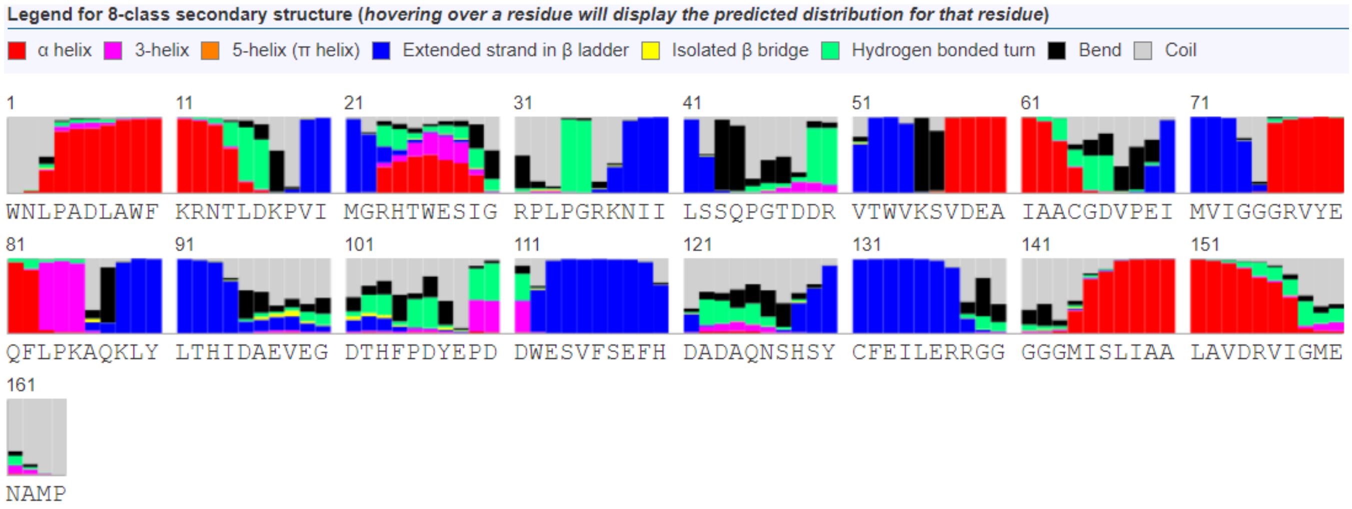

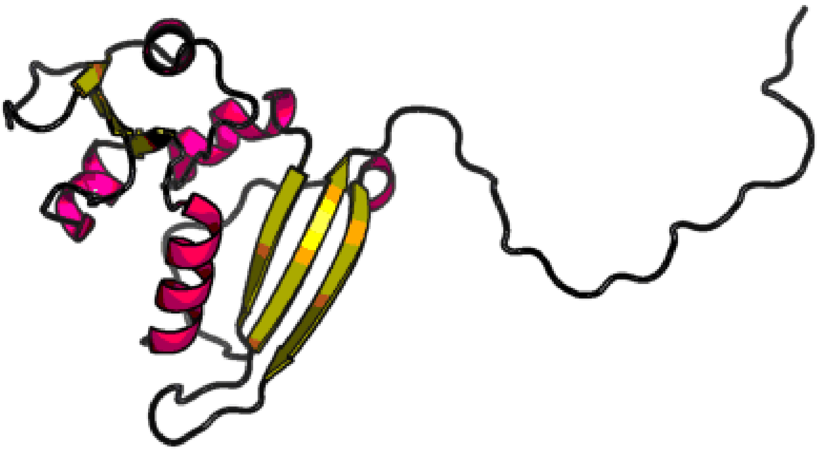

Template: PDB 1rx4A

CP site: Asn23

Target sequence:

NLPADLAWFKRNTLDKPVIMGRHTWESIGRPLPGRKNIILSSQPGTDDRVTWVKSVDEAI
AACGDVPEIMVIGGGRVYEQFLPKAQKLYLTHIDAEVEGDTHFPDYEPDDWESVFSEFHD
ADAQNSHSYCFEILERRGGGGGMISLIAALAVDRVIGMENAMPW

Summary

- The input predicted as **1** domain(s)
- Best template: **2drcA**, p-value **5.49e-09**
- Overall uGDT (GDT): **136** (**82**)
- **164**(**100%**) residues are modeled
- **13**(**7%**) positions predicted as disordered
- Secondary struct: **23%**H, **26%**E, **50%**C
- Solvent access: **37%**E, **37%**M, **25%**B

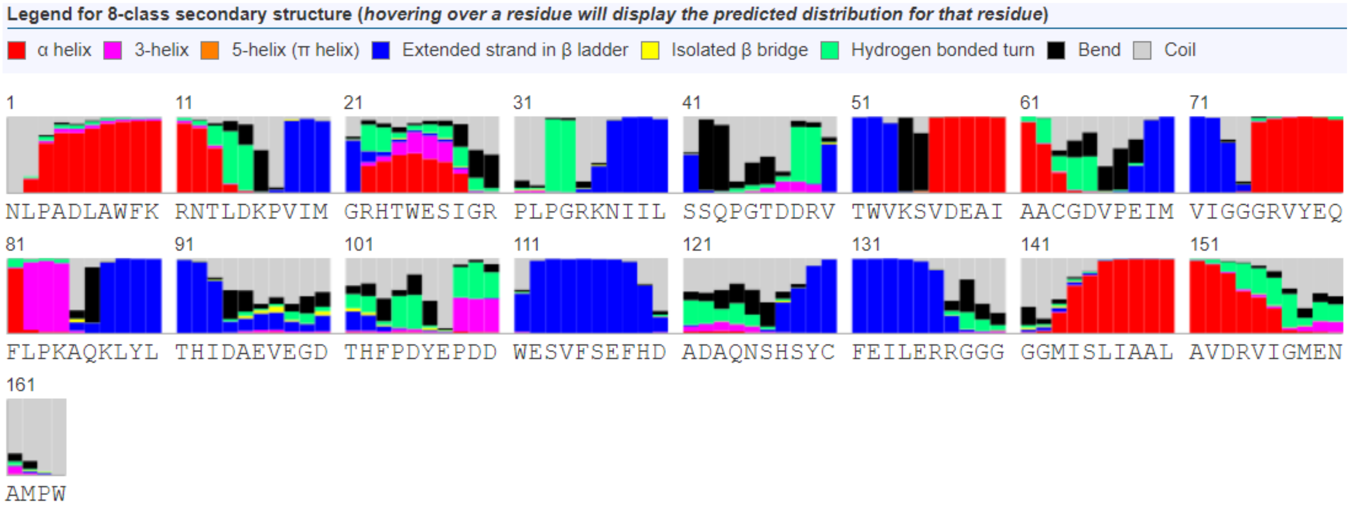

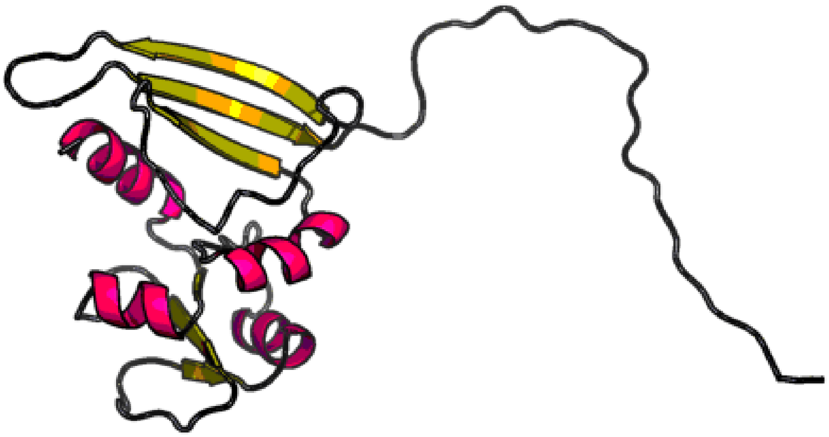

Template: PDB 1rx4A

CP site: Leu24

Target sequence:

LPADLAWFKRNTLDKPVIMGRHTWESIGRPLPGRKNIILSSQPGTDDRVTWVKSVDEAIA
ACGDVPEIMVIGGGRVYEQFLPKAQKLYLTHIDAEVEGDTHFPDYEPDDWESVFSEFHDA
DAQNSHSYCFEILERRGGGGGMISLIAALAVDRVIGMENAMPWN

Summary

- The input predicted as **1** domain(s)
- Best template: **2drcA**, p-value **5.30e-09**
- Overall uGDT (GDT): **135** (**82**)
- **164**(**100%**) residues are modeled
- **10**(**6%**) positions predicted as disordered
- Secondary struct: **22%**H, **25%**E, **52%**C
- Solvent access: **36%**E, **36%**M, **26%**B

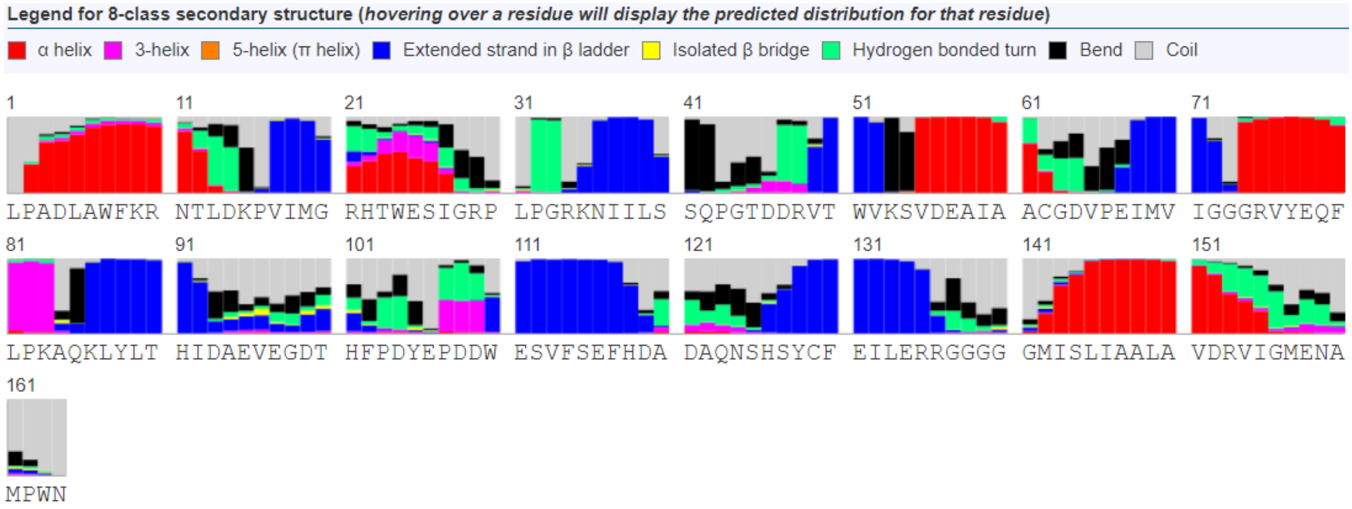

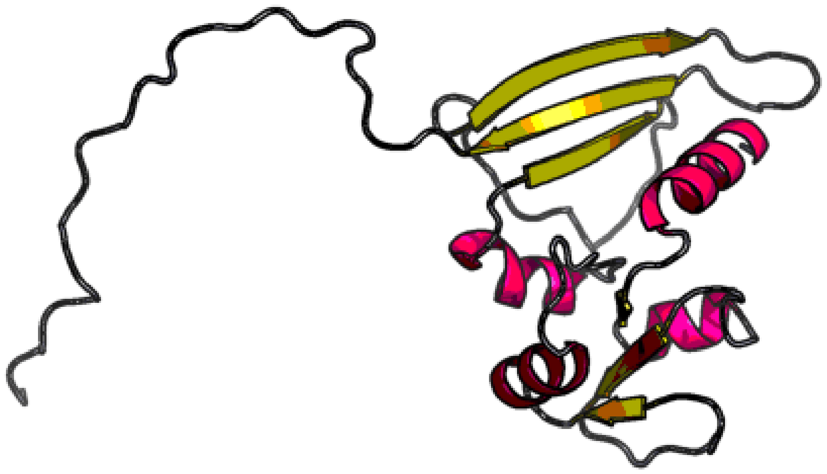

Template: PDB 1rx4A

CP site: Pro25

Target sequence:

PADLAWFKRNTLDKPVIMGRHTWESIGRPLPGRKNIILSSQPGTDDRVTWVKSVDEAIAA
CGDVPEIMVIGGGRVYEQFLPKAQKLYLTHIDAEVEGDTHFPDYEPDDWESVFSEFHDAD
AQNSHSYCFEILERRGGGGGMISLIAALAVDRVIGMENAMPWNL

Summary

- The input predicted as **1** domain(s)
- Best template: **2drcA**, p-value **1.03e-08**
- Overall uGDT (GDT): **134** (**82**)
- **164**(**100%**) residues are modeled
- **10**(**6%**) positions predicted as disordered
- Secondary struct: **23%**H, **25%**E, **51%**C
- Solvent access: **34%**E, **38%**M, **26%**B

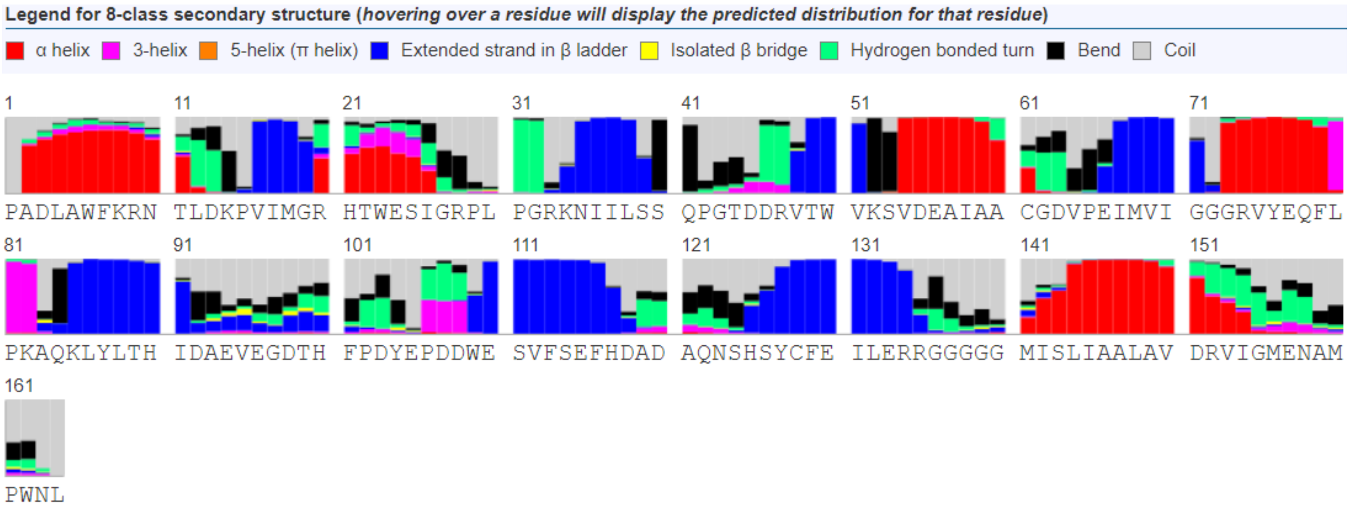

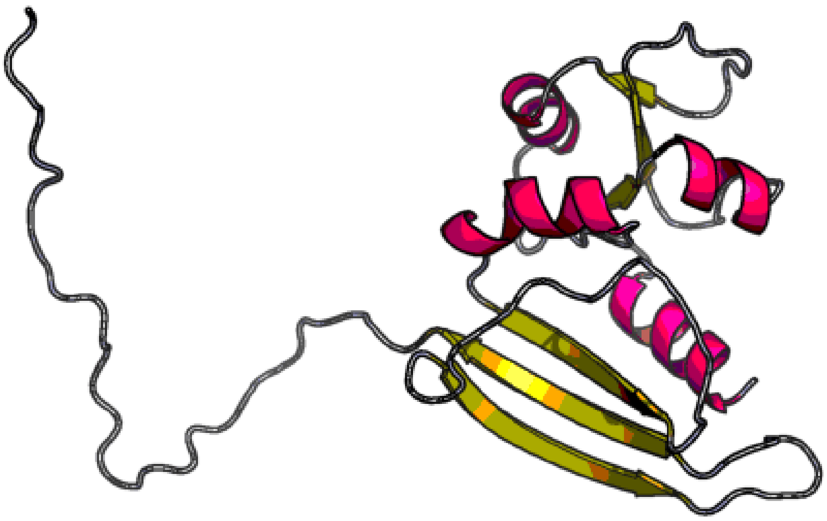

Template: PDB 1rx4A

CP site: Ala26

Target sequence:

ADLAWFKRNTLDKPVIMGRHTWESIGRPLPGRKNIILSSQPGTDDRVTWVKSVDEAIAAC
GDVPEIMVIGGGRVYEQFLPKAQKLYLTHIDAEVEGDTHFPDYEPDDWESVFSEFHDADA
QNSHSYCFEILERRGGGGGMISLIAALAVDRVIGMENAMPWNLP

Summary

- The input predicted as **1** domain(s)
- Best template: **2drcA**, p-value **1.57e-08**
- Overall uGDT (GDT): **133** (**81**)
- **164**(**100%**) residues are modeled
- **6**(**3%**) positions predicted as disordered
- Secondary struct: **16%**H, **32%**E, **51%**C
- Solvent access: **35%**E, **34%**M, **29%**B

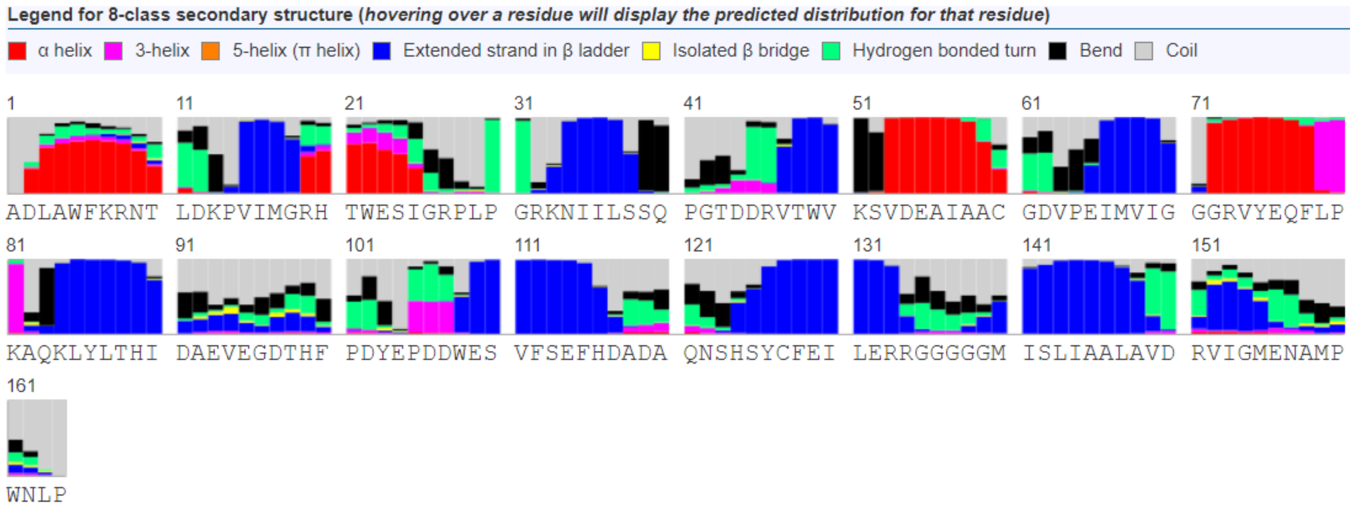

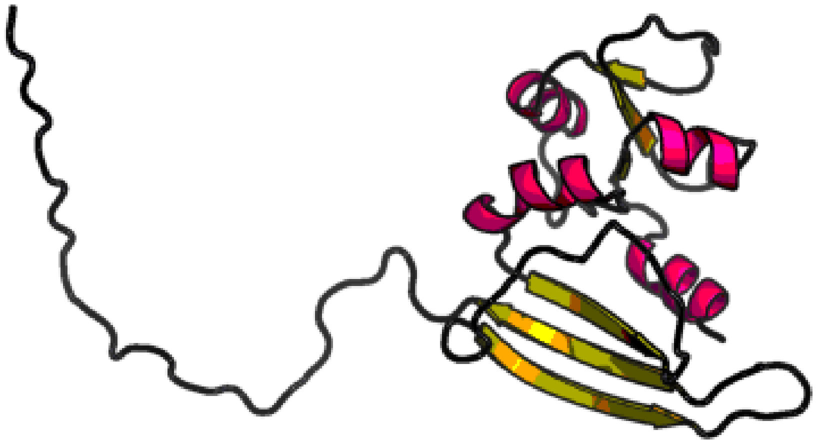

Template: PDB 1rx4A

CP site: Asp27

Target sequence:

DLAWFKRNTLDKPVIMGRHTWESIGRPLPGRKNIILSSQPGTDDRVTWVKSVDEAIAACG
DVPEIMVIGGGRVYEQFLPKAQKLYLTHIDAEVEGDTHFPDYEPDDWESVFSEFHDADAQ
NSHSYCFEILERRGGGGGMISLIAALAVDRVIGMENAMPWNLPA

Summary

- The input predicted as **1** domain(s)
- Best template: **2drcA**, p-value **1.91e-08**
- Overall uGDT (GDT): **133** (**81**)
- **164**(**100%**) residues are modeled
- **5**(**3%**) positions predicted as disordered
- Secondary struct: **13%**H, **31%**E, **54%**C
- Solvent access: **35%**E, **32%**M, **31%**B

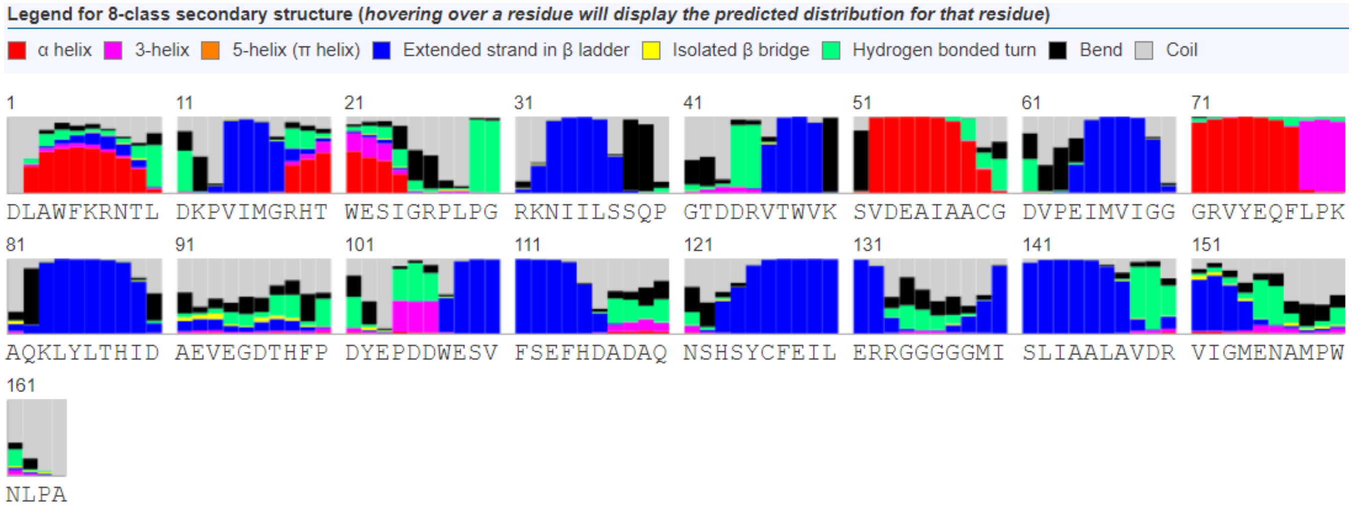

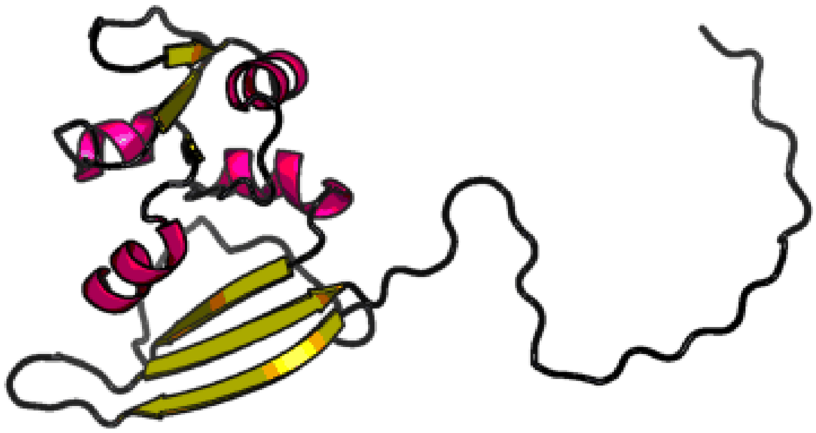

Template: PDB 1rx4A

CP site: Leu28

Target sequence:

LAWFKRNTLDKPVIMGRHTWESIGRPLPGRKNIILSSQPGTDDRVTWVKSVDEAIAACGD
VPEIMVIGGGRVYEQFLPKAQKLYLTHIDAEVEGDTHFPDYEPDDWESVFSEFHDADAQN
SHSYCFEILERRGGGGGMISLIAALAVDRVIGMENAMPWNLPAD

Summary

- The input predicted as **1** domain(s)
- Best template: **2drcA**, p-value **3.36e-08**
- Overall uGDT (GDT): **131** (**80**)
- **164**(**100%**) residues are modeled
- **8**(**4%**) positions predicted as disordered
- Secondary struct: **10%**H, **35%**E, **53%**C
- Solvent access: **35%**E, **32%**M, **31%**B

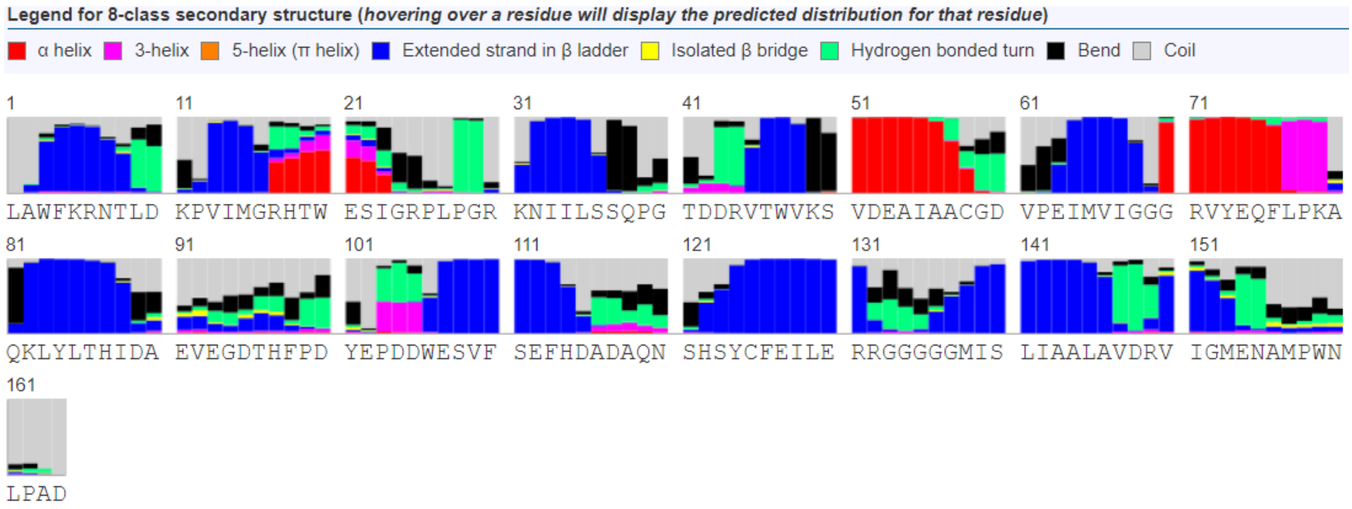

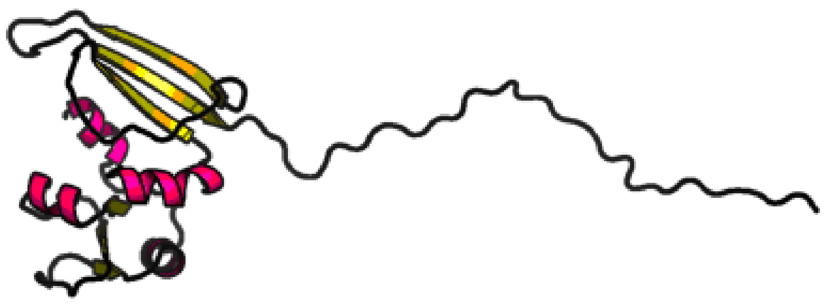

Template: PDB 1rx4A

CP site: Ala29

Target sequence:

AWFKRNTLDKPVIMGRHTWESIGRPLPGRKNIILSSQPGTDDRVTWVKSVDEAIAACGDV
PEIMVIGGGRVYEQFLPKAQKLYLTHIDAEVEGDTHFPDYEPDDWESVFSEFHDADAQNS
HSYCFEILERRGGGGGMISLIAALAVDRVIGMENAMPWNLPADL

Summary

- The input predicted as **1** domain(s)
- Best template: **2drcA**, p-value **3.00e-08**
- Overall uGDT (GDT): **130** (**79**)
- **164**(**100%**) residues are modeled
- **5**(**3%**) positions predicted as disordered
- Secondary struct: **11%**H, **34%**E, **53%**C
- Solvent access: **34%**E, **32%**M, **32%**B

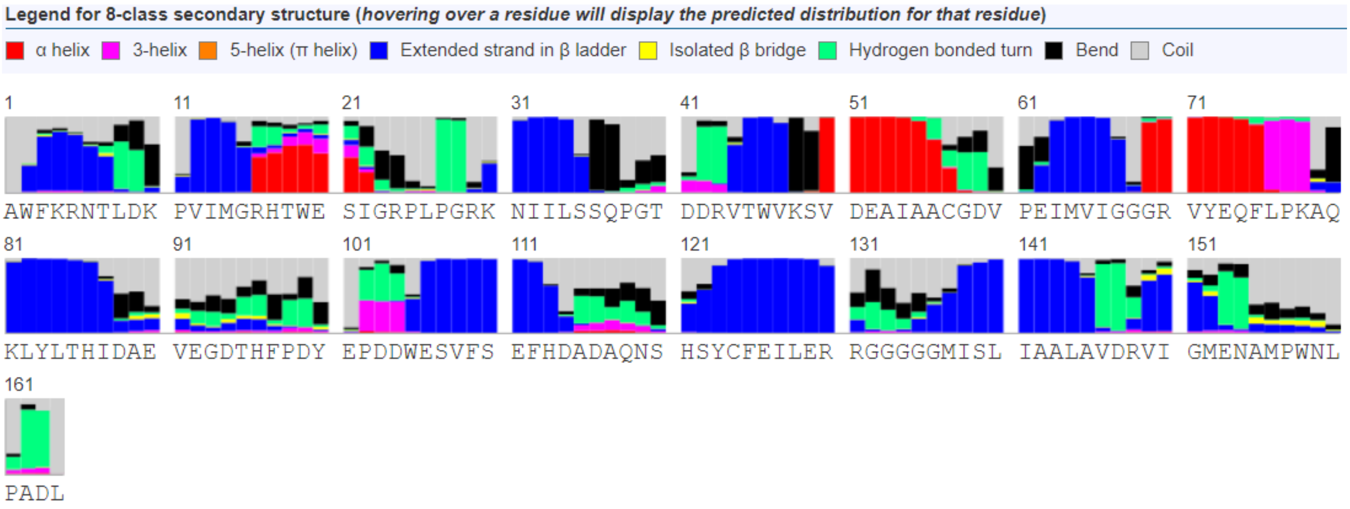

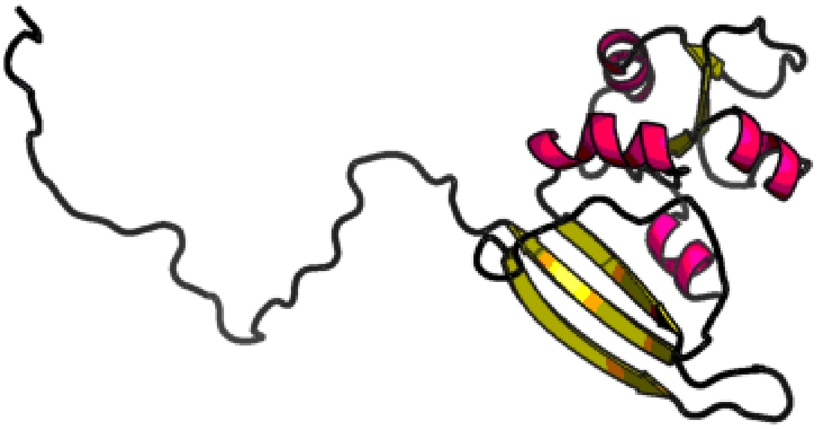

Template: PDB 1rx4A

CP site: Ala37

Target sequence:

DKPVIMGRHTWESIGRPLPGRKNIILSSQPGTDDRVTWVKSVDEAIAACGDVPEIMVIGG
GRVYEQFLPKAQKLYLTHIDAEVEGDTHFPDYEPDDWESVFSEFHDADAQNSHSYCFEIL
ERRGGGGGMISLIAALAVDRVIGMENAMPWNLPADLAWFKRNTL

Summary

- The input predicted as **1** domain(s)
- Best template: **2drcA**, p-value **3.35e-08**
- Overall uGDT (GDT): **124** (**76**)
- **164**(**100%**) residues are modeled
- **4**(**2%**) positions predicted as disordered
- Secondary struct: **17%**H, **34%**E, **48%**C
- Solvent access: **33%**E, **34%**M, **32%**B

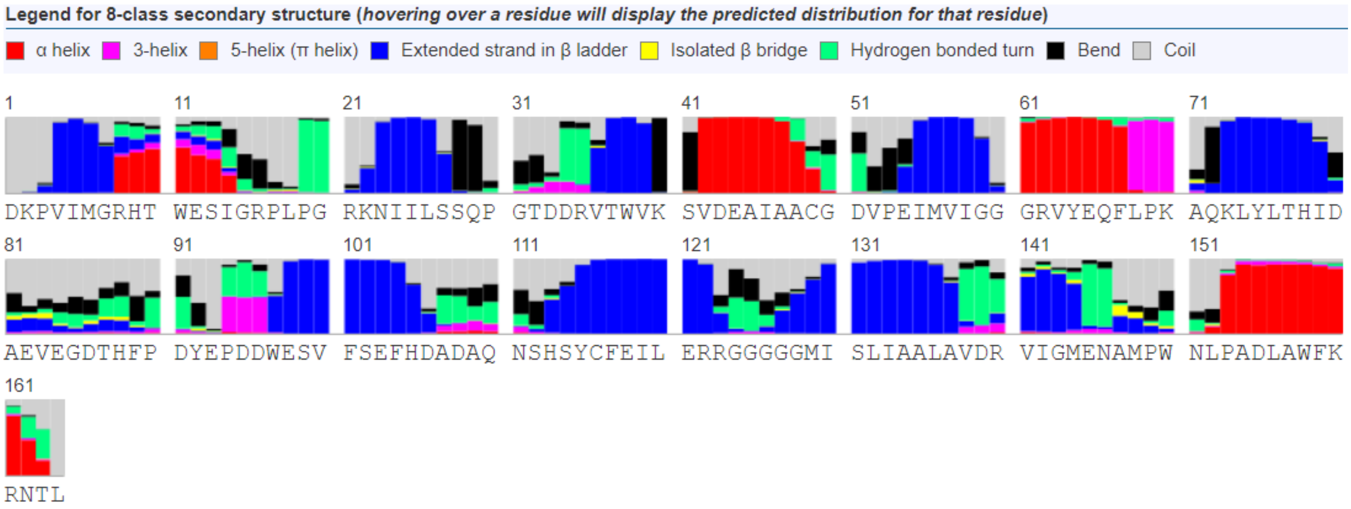

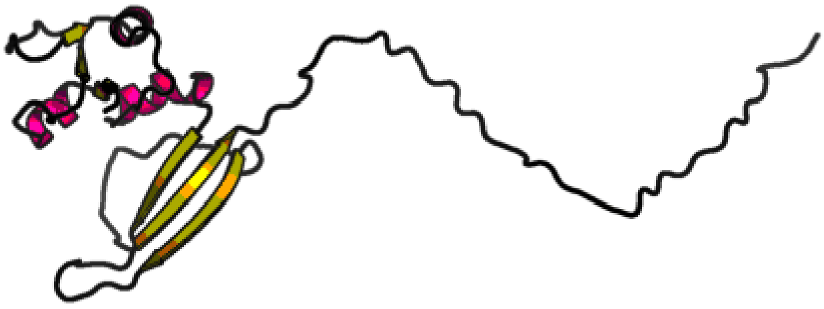

Template: PDB 1rx4A

CP site: Lys38

Target sequence:

KPVIMGRHTWESIGRPLPGRKNIILSSQPGTDDRVTWVKSVDEAIAACGDVPEIMVIGGG
RVYEQFLPKAQKLYLTHIDAEVEGDTHFPDYEPDDWESVFSEFHDADAQNSHSYCFEILE
RRGGGGGMISLIAALAVDRVIGMENAMPWNLPADLAWFKRNTLD

Summary

- The input predicted as **1** domain(s)
- Best template: **2drcA**, p-value **2.77e-08**
- Overall uGDT (GDT): **125** (**76**)
- **164**(**100%**) residues are modeled
- **6**(**3%**) positions predicted as disordered
- Secondary struct: **14%**H, **33%**E, **51%**C
- Solvent access: **32%**E, **35%**M, **31%**B

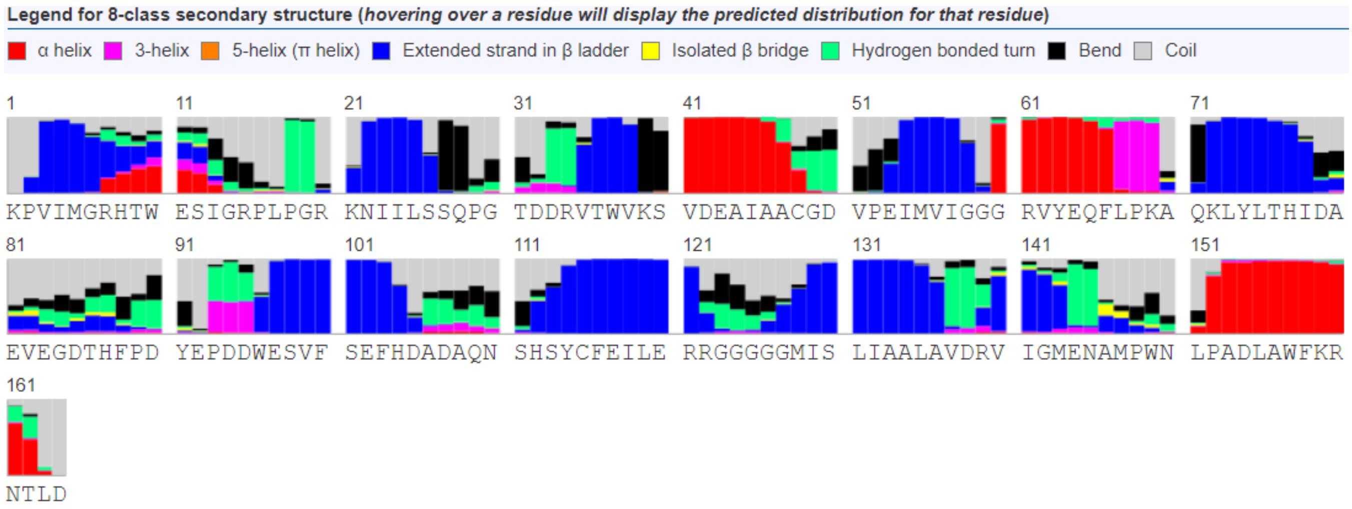

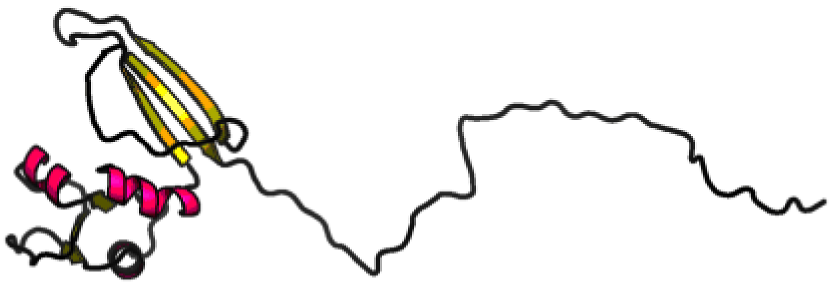

Template: PDB 1rx4A

CP site: Pro39

Target sequence:

PVIMGRHTWESIGRPLPGRKNIILSSQPGTDDRVTWVKSVDEAIAACGDVPEIMVIGGGR
VYEQFLPKAQKLYLTHIDAEVEGDTHFPDYEPDDWESVFSEFHDADAQNSHSYCFEILER
RGGGGGMISLIAALAVDRVIGMENAMPWNLPADLAWFKRNTLDK

Summary

- The input predicted as **1** domain(s)
- Best template: **2drcA**, p-value **2.50e-08**
- Overall uGDT (GDT): **123** (**75**)
- **164**(**100%**) residues are modeled
- **6**(**3%**) positions predicted as disordered
- Secondary struct: **15%**H, **34%**E, **50%**C
- Solvent access: **32%**E, **35%**M, **31%**B

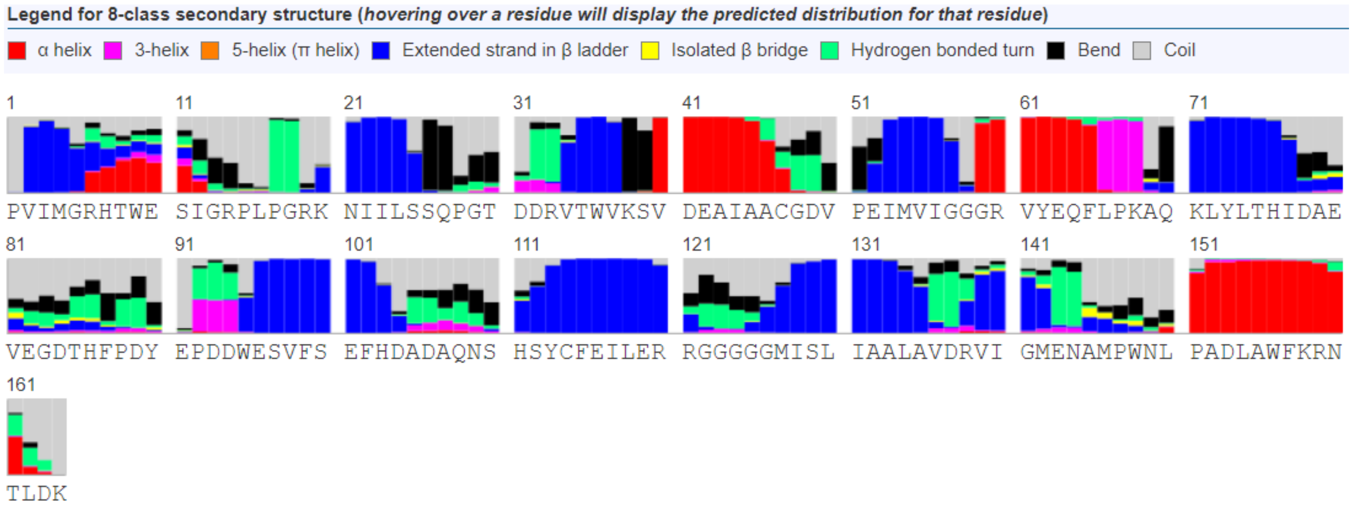

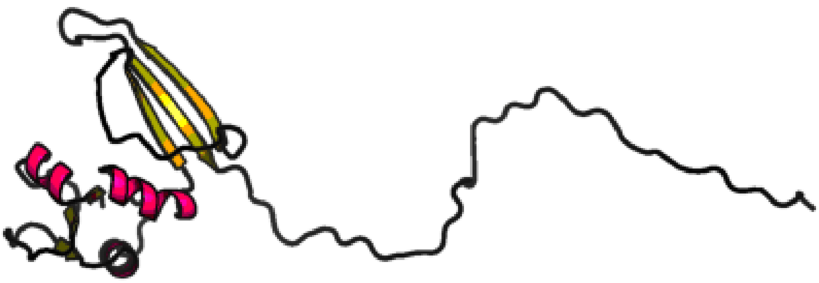

Template: PDB 1rx4A

CP site: Ile50

Target sequence:

IGRPLPGRKNIILSSQPGTDDRVTWVKSVDEAIAACGDVPEIMVIGGGRVYEQFLPKAQK
LYLTHIDAEVEGDTHFPDYEPDDWESVFSEFHDADAQNSHSYCFEILERRGGGGGMISLI
AALAVDRVIGMENAMPWNLPADLAWFKRNTLDKPVIMGRHTWES

Summary

- The input predicted as **1** domain(s)
- Best template: **5uioA**, p-value **2.12e-07**
- Overall uGDT (GDT): **105** (**64**)
- **164**(**100%**) residues are modeled
- **9**(**5%**) positions predicted as disordered
- Secondary struct: **14%**H, **32%**E, **53%**C
- Solvent access: **34%**E, **35%**M, **29%**B

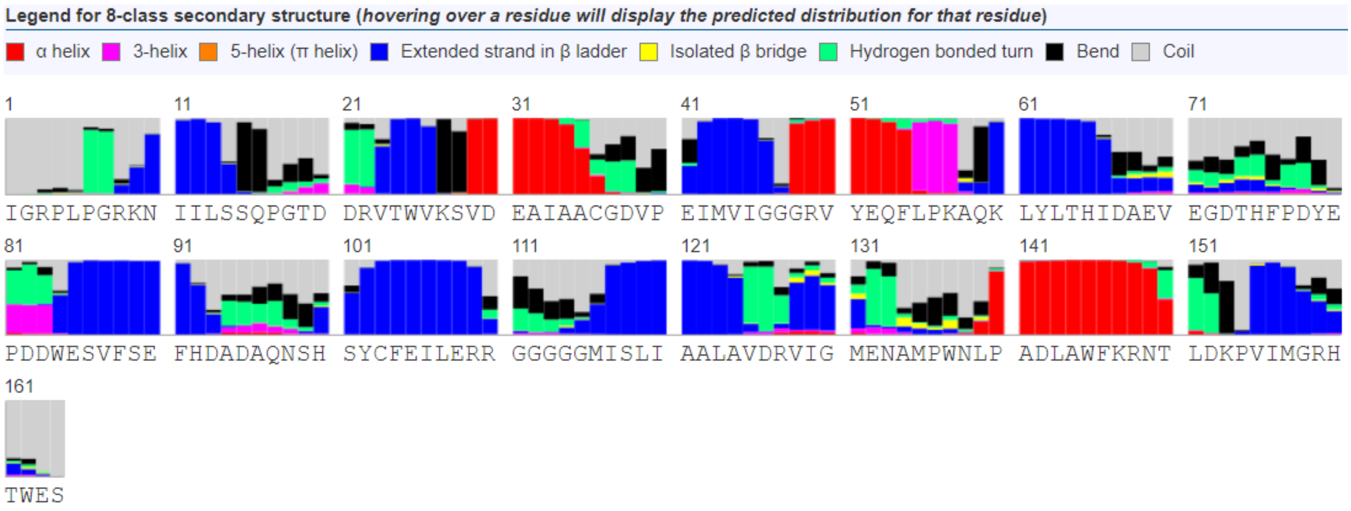

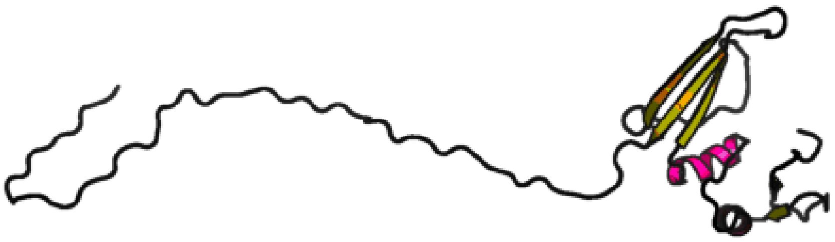

Template: PDB 1rx4A

CP site: Gly51

Target sequence:

GRPLPGRKNIILSSQPGTDDRVTWVKSVDEAIAACGDVPEIMVIGGGRVYEQFLPKAQKL
YLTHIDAEVEGDTHFPDYEPDDWESVFSEFHDADAQNSHSYCFEILERRGGGGGMISLIA
ALAVDRVIGMENAMPWNLPADLAWFKRNTLDKPVIMGRHTWESI

Summary

- The input predicted as **1** domain(s)
- Best template: **5uioA**, p-value **2.94e-07**
- Overall uGDT (GDT): **106** (**64**)
- **114**(**69%**) residues are modeled
- **6**(**3%**) positions predicted as disordered
- Secondary struct: **14%**H, **32%**E, **53%**C
- Solvent access: **34%**E, **35%**M, **30%**B

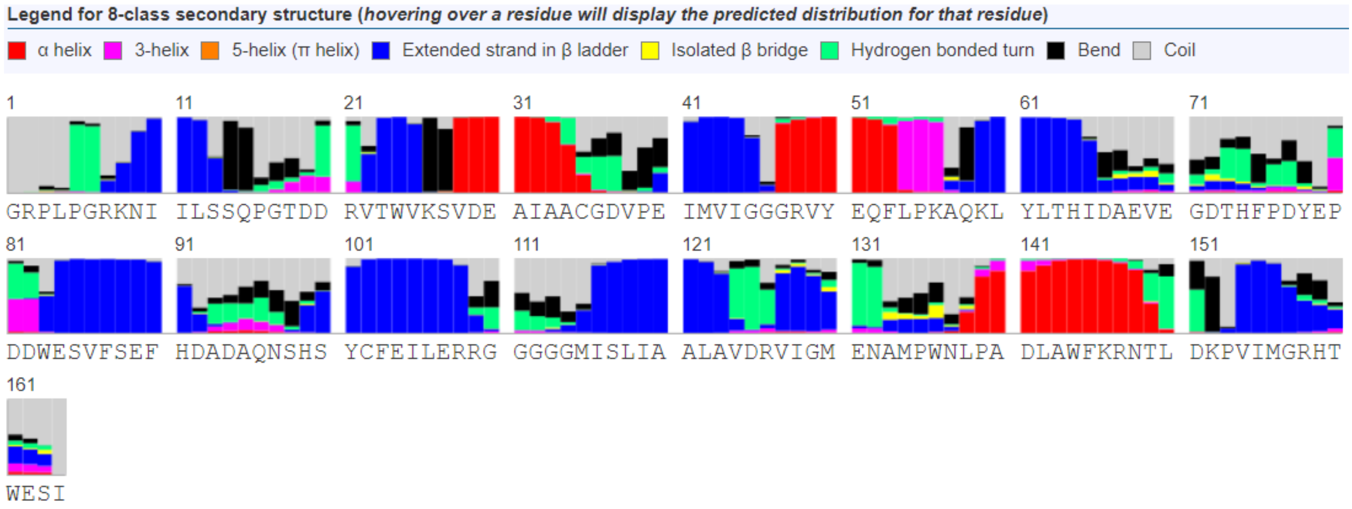

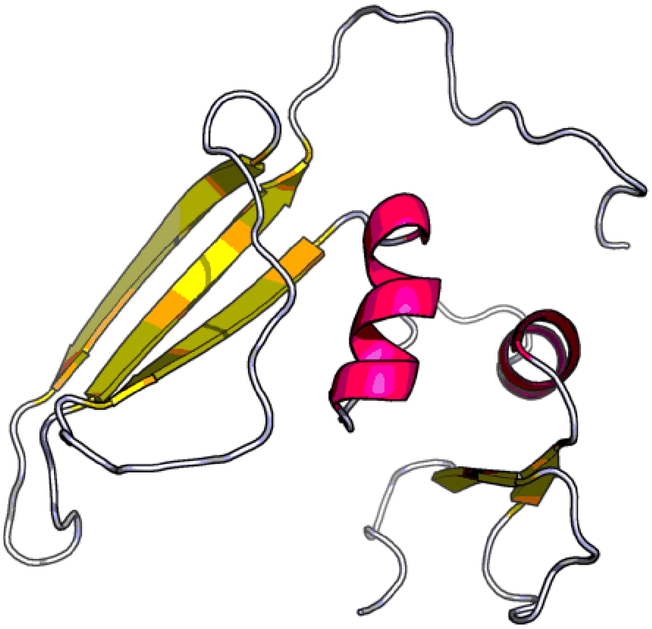

Template: PDB 1rx4A

CP site: Arg52

Target sequence:

RPLPGRKNIILSSQPGTDDRVTWVKSVDEAIAACGDVPEIMVIGGGRVYEQFLPKAQKLY
LTHIDAEVEGDTHFPDYEPDDWESVFSEFHDADAQNSHSYCFEILERRGGGGGMISLIAA
LAVDRVIGMENAMPWNLPADLAWFKRNTLDKPVIMGRHTWESIG

Summary

•The input predicted as **2** domain(s)
•Best template: **5uioA**, p-value **3.43e-07**
•Overall uGDT (GDT): **166** (**101**)
•**164**(**100%**) residues are modeled
•**8**(**4%**) positions predicted as disordered
•Secondary struct: **14%**H, **32%**E, **53%**C
•Solvent access: **32%**E, **34%**M, **32%**B

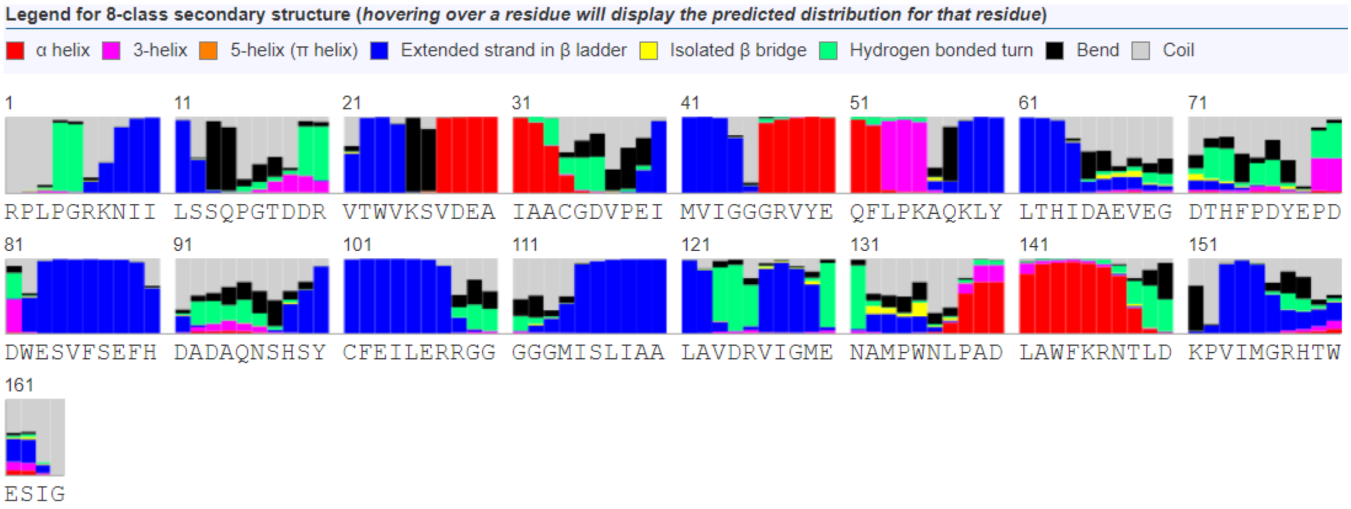

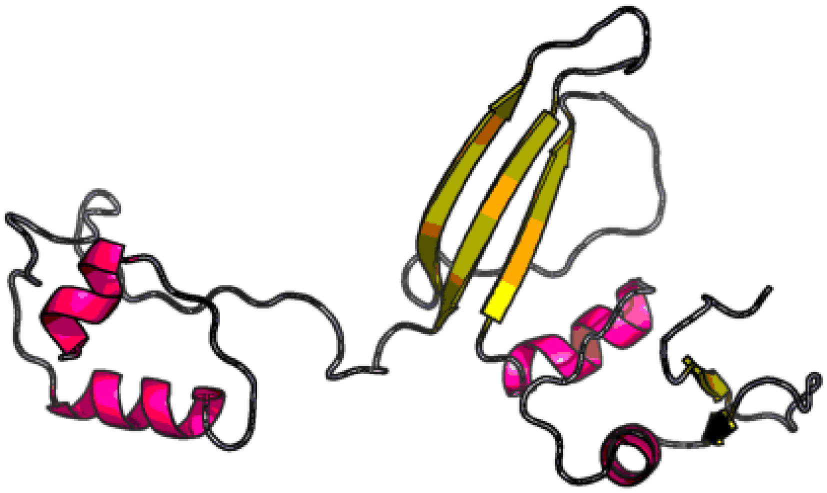

Template: PDB 1rx4A

CP site: Pro53

Target sequence:

PLPGRKNIILSSQPGTDDRVTWVKSVDEAIAACGDVPEIMVIGGGRVYEQFLPKAQKLYL
THIDAEVEGDTHFPDYEPDDWESVFSEFHDADAQNSHSYCFEILERRGGGGGMISLIAAL
AVDRVIGMENAMPWNLPADLAWFKRNTLDKPVIMGRHTWESIGR

Summary

- The input predicted as **2** domain(s)
- Best template: **5uioA**, p-value **4.00e-07**
- Overall uGDT (GDT): **167** (**102**)
- **164**(**100%**) residues are modeled
- **9**(**5%**) positions predicted as disordered
- Secondary struct: **15%**H, **32%**E, **52%**C
- Solvent access: **32%**E, **34%**M, **32%**B

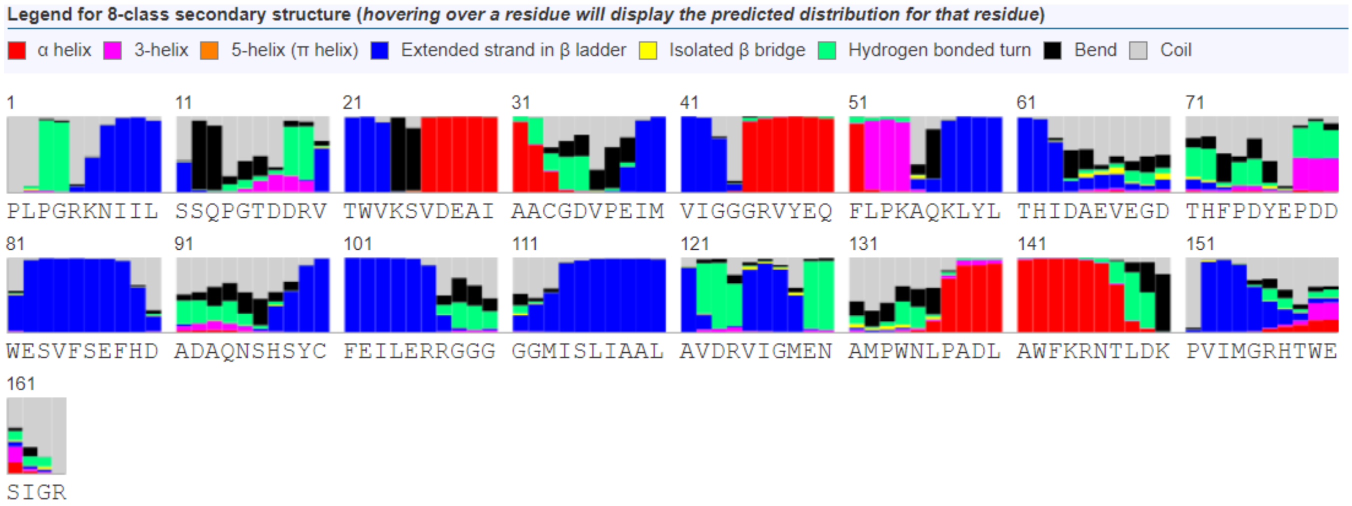

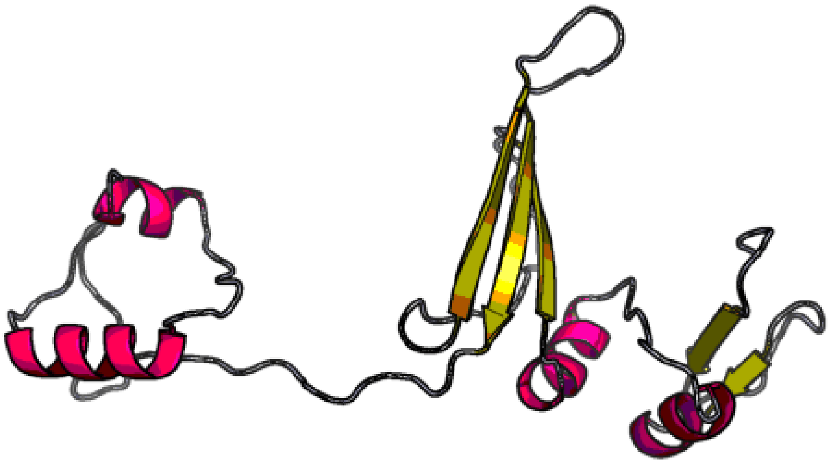

Template: PDB 1rx4A

CP site: Leu54

Target sequence:

LPGRKNIILSSQPGTDDRVTWVKSVDEAIAACGDVPEIMVIGGGRVYEQFLPKAQKLYLT
HIDAEVEGDTHFPDYEPDDWESVFSEFHDADAQNSHSYCFEILERRGGGGGMISLIAALA
VDRVIGMENAMPWNLPADLAWFKRNTLDKPVIMGRHTWESIGRP

Summary

- The input predicted as **2** domain(s)
- Best template: **5uioA**, p-value **7.46e-07**
- Overall uGDT (GDT): **165** (**101**)
- **164**(**100%**) residues are modeled
- **10**(**6%**) positions predicted as disordered
- Secondary struct: **15%**H, **32%**E, **51%**C
- Solvent access: **32%**E, **35%**M, **31%**B

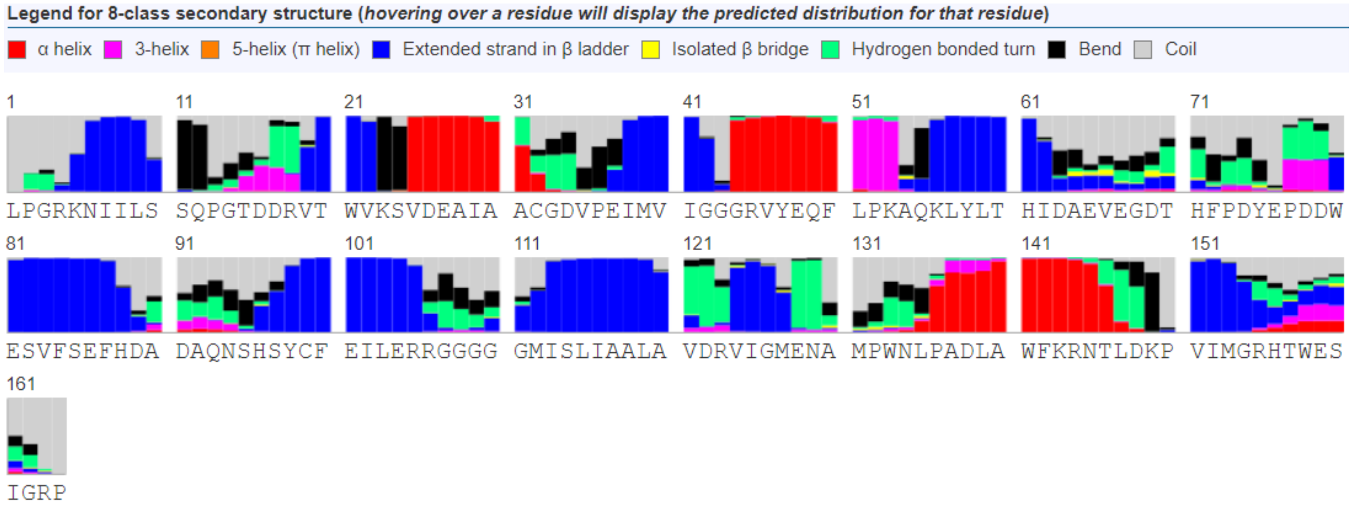

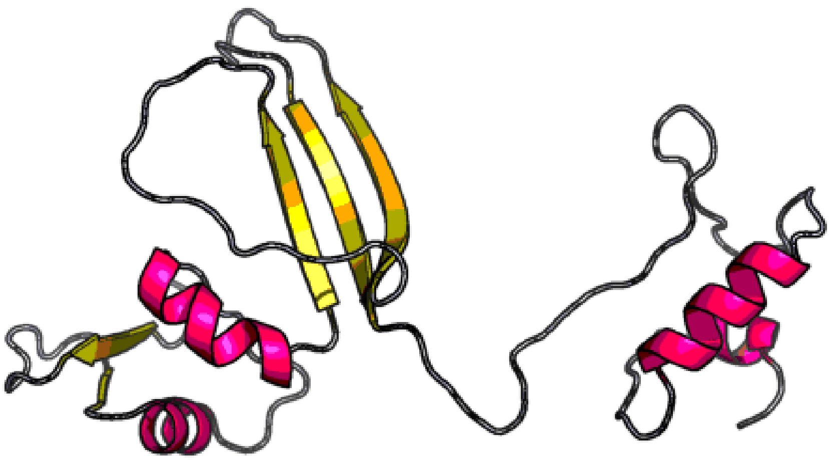

Template: PDB 1rx4A

CP site: Pro55

Target sequence:

PGRKNIILSSQPGTDDRVTWVKSVDEAIAACGDVPEIMVIGGGRVYEQFLPKAQKLYLTH
IDAEVEGDTHFPDYEPDDWESVFSEFHDADAQNSHSYCFEILERRGGGGGMISLIAALAV
DRVIGMENAMPWNLPADLAWFKRNTLDKPVIMGRHTWESIGRPL

Summary

- The input predicted as **2** domain(s)
- Best template: **5uioA**, p-value **5.03e-07**
- Overall uGDT (GDT): **163** (**99**)
- **164**(**100%**) residues are modeled
- **7**(**4%**) positions predicted as disordered
- Secondary struct: **15%**H, **32%**E, **51%**C
- Solvent access: **33%**E, **34%**M, **31%**B

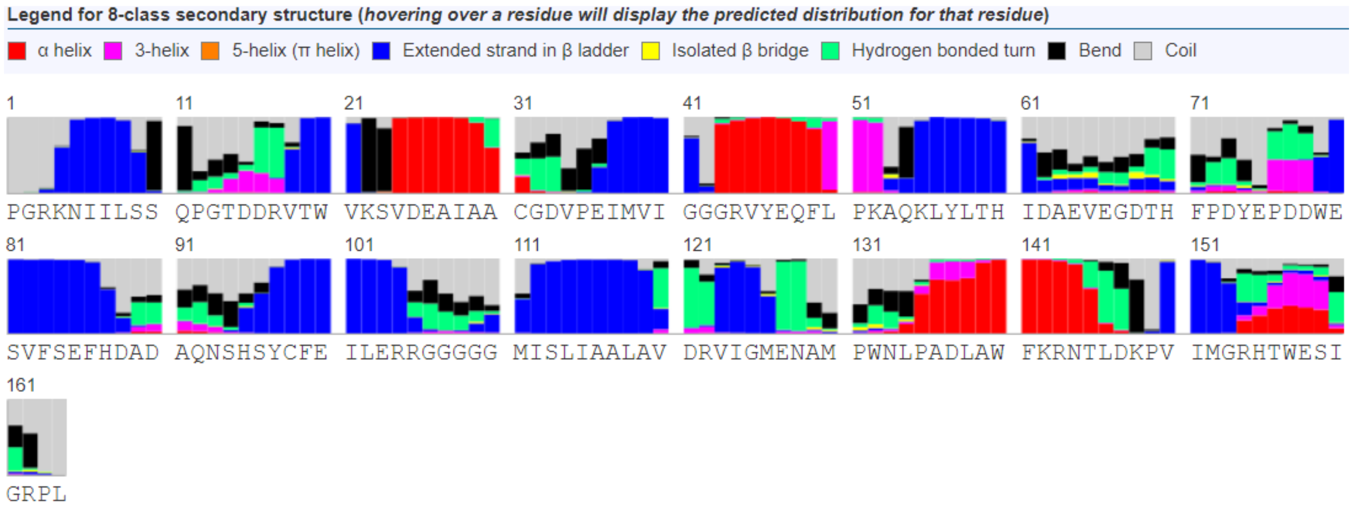

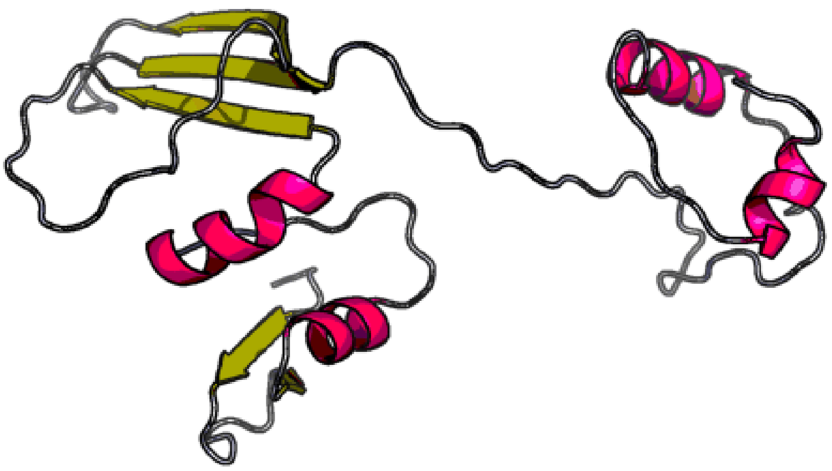

Template: PDB 1rx4A

CP site: Gly56

Target sequence:

GRKNIILSSQPGTDDRVTWVKSVDEAIAACGDVPEIMVIGGGRVYEQFLPKAQKLYLTHI
DAEVEGDTHFPDYEPDDWESVFSEFHDADAQNSHSYCFEILERRGGGGGMISLIAALAVD
RVIGMENAMPWNLPADLAWFKRNTLDKPVIMGRHTWESIGRPLP

Summary

- The input predicted as **2** domain(s)
- Best template: **5uioA**, p-value **5.95e-07**
- Overall uGDT (GDT): **162** (**99**)
- **164**(**100%**) residues are modeled
- **5**(**3%**) positions predicted as disordered
- Secondary struct: **15%**H, **34%**E, **50%**C
- Solvent access: **33%**E, **34%**M, **31%**B

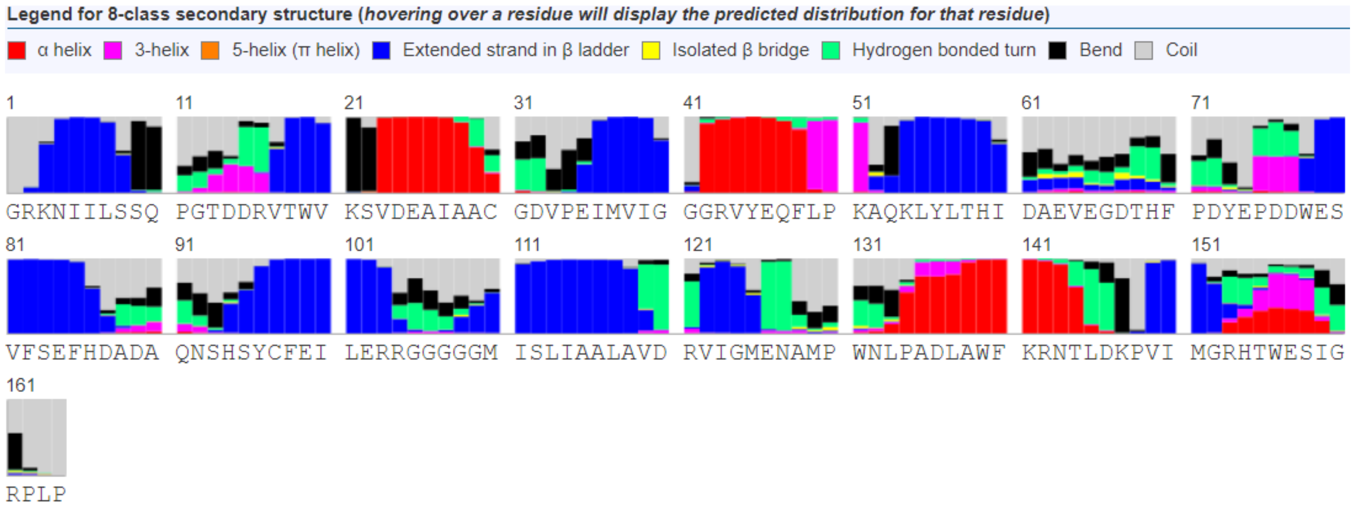

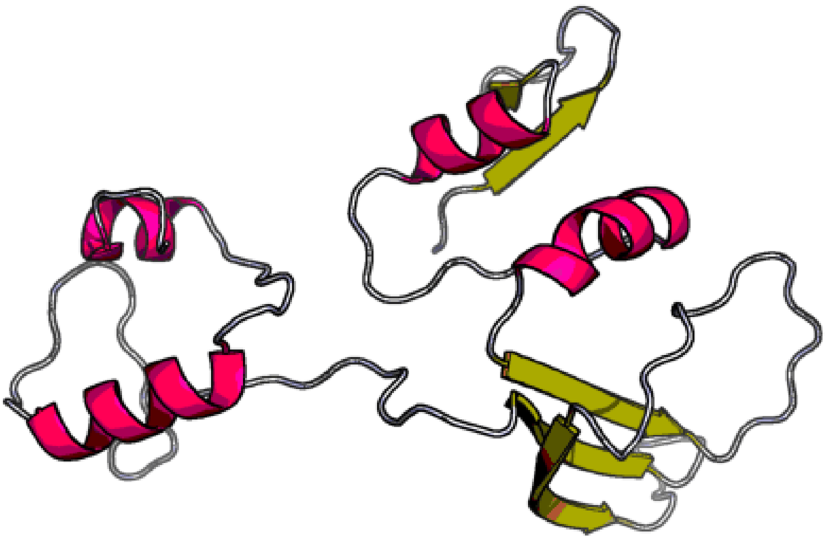

Template: PDB 1rx4A

CP site: Ser64

Target sequence:

SQPGTDDRVTWVKSVDEAIAACGDVPEIMVIGGGRVYEQFLPKAQKLYLTHIDAEVEGDT
HFPDYEPDDWESVFSEFHDADAQNSHSYCFEILERRGGGGGMISLIAALAVDRVIGMENA
MPWNLPADLAWFKRNTLDKPVIMGRHTWESIGRPLPGRKNIILS

Summary

- •The input predicted as **2** domain(s)
- •Best template: **5uioA**, p-value **1.19e-06**
- •Overall uGDT (GDT): **165** (**100**)
- •**164**(**100%**) residues are modeled
- •**10**(**6%**) positions predicted as disordered
- •Secondary struct: **15%**H, **31%**E, **53%**C
- •Solvent access: **34%**E, **36%**M, **28%**B

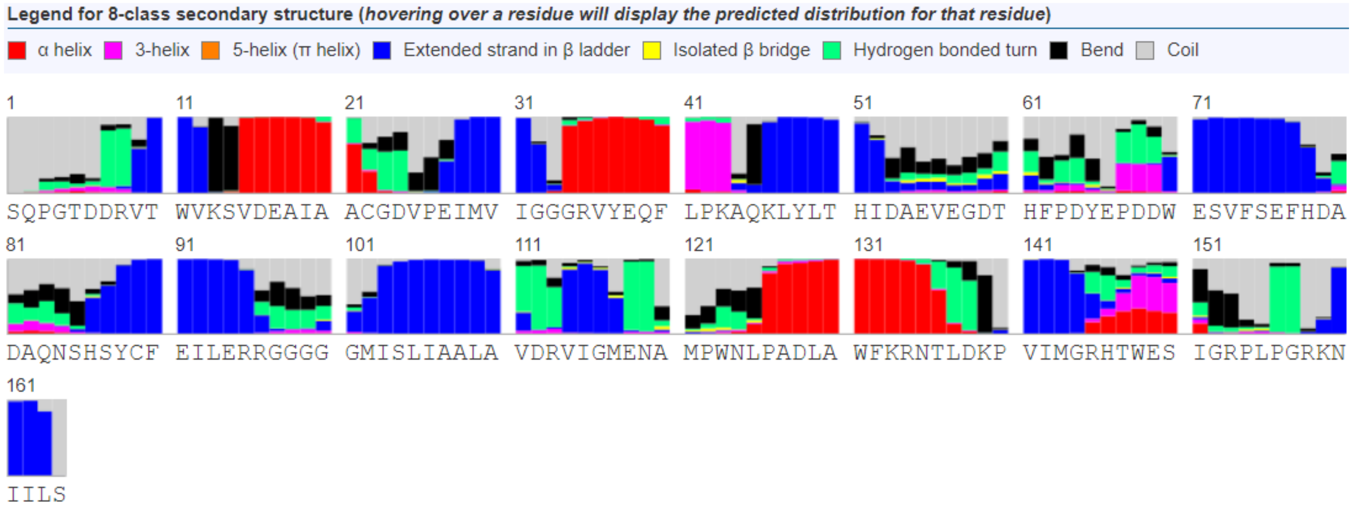

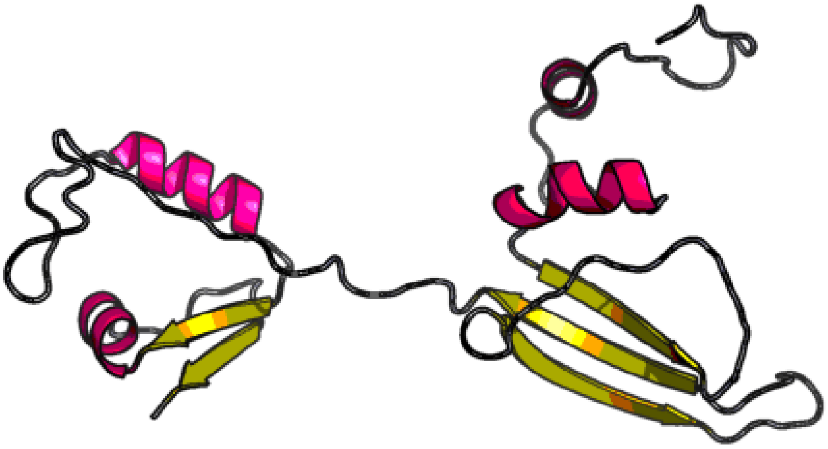

Template: PDB 1rx4A

CP site: Gln65

Target sequence:

QPGTDDRVTWVKSVDEAIAACGDVPEIMVIGGGRVYEQFLPKAQKLYLTHIDAEVEGDTH
FPDYEPDDWESVFSEFHDADAQNSHSYCFEILERRGGGGGMISLIAALAVDRVIGMENAM
PWNLPADLAWFKRNTLDKPVIMGRHTWESIGRPLPGRKNIILSS

Summary

- The input predicted as **2** domain(s)
- Best template: **5uioA**, p-value **9.81e-07**
- Overall uGDT (GDT): **166** (**101**)
- **164**(**100%**) residues are modeled
- **9**(**5%**) positions predicted as disordered
- Secondary struct: **15%**H, **31%**E, **53%**C
- Solvent access: **34%**E, **34%**M, **31%**B

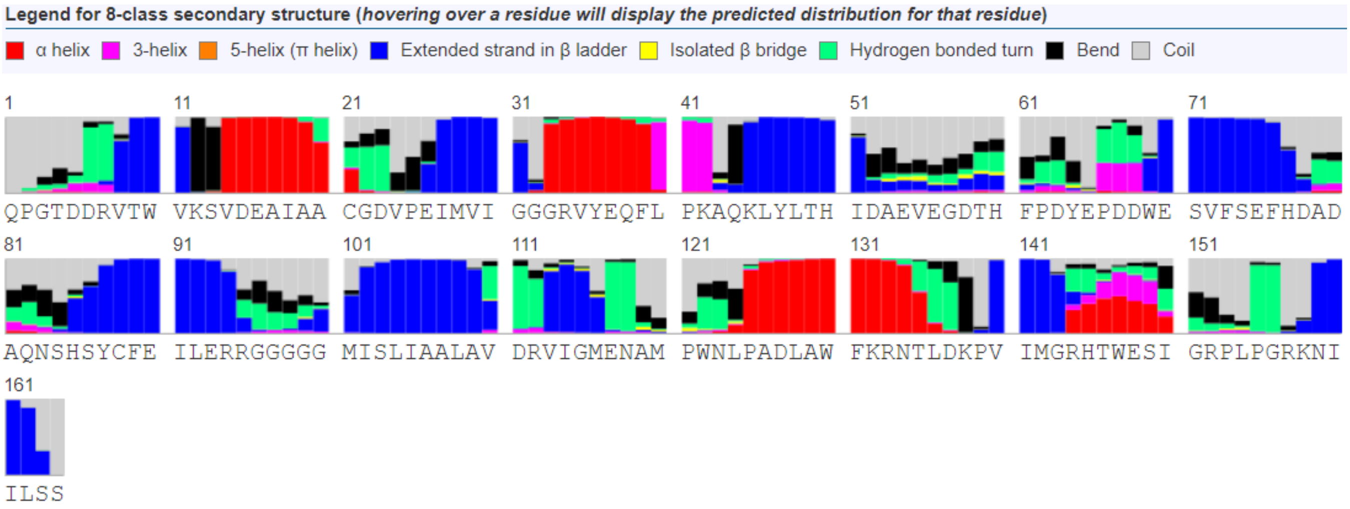

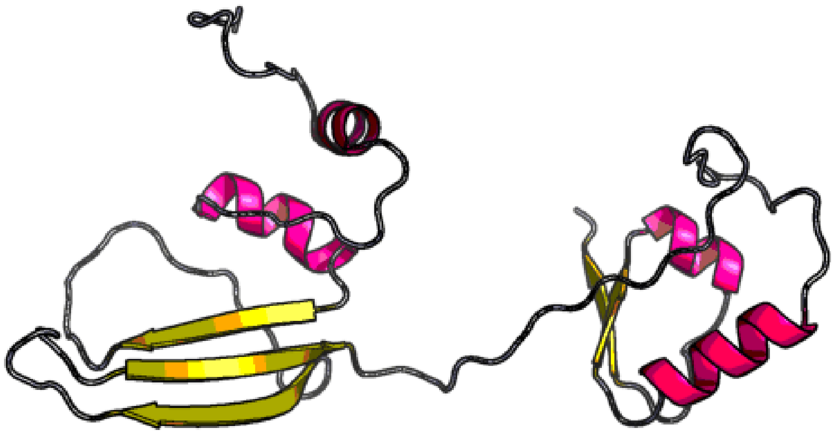

Template: PDB 1rx4A

CP site: Pro66

Target sequence:

PGTDDRVTWVKSVDEAIAACGDVPEIMVIGGGRVYEQFLPKAQKLYLTHIDAEVEGDTHF
PDYEPDDWESVFSEFHDADAQNSHSYCFEILERRGGGGGMISLIAALAVDRVIGMENAMP
WNLPADLAWFKRNTLDKPVIMGRHTWESIGRPLPGRKNIILSSQ

Summary

- The input predicted as **2** domain(s)
- Best template: **5uioA**, p-value **1.60e-06**
- Overall uGDT (GDT): **166** (**101**)
- **164**(**100%**) residues are modeled
- **8**(**4%**) positions predicted as disordered
- Secondary struct: **15%**H, **31%**E, **53%**C
- Solvent access: **32%**E, **35%**M, **31%**B

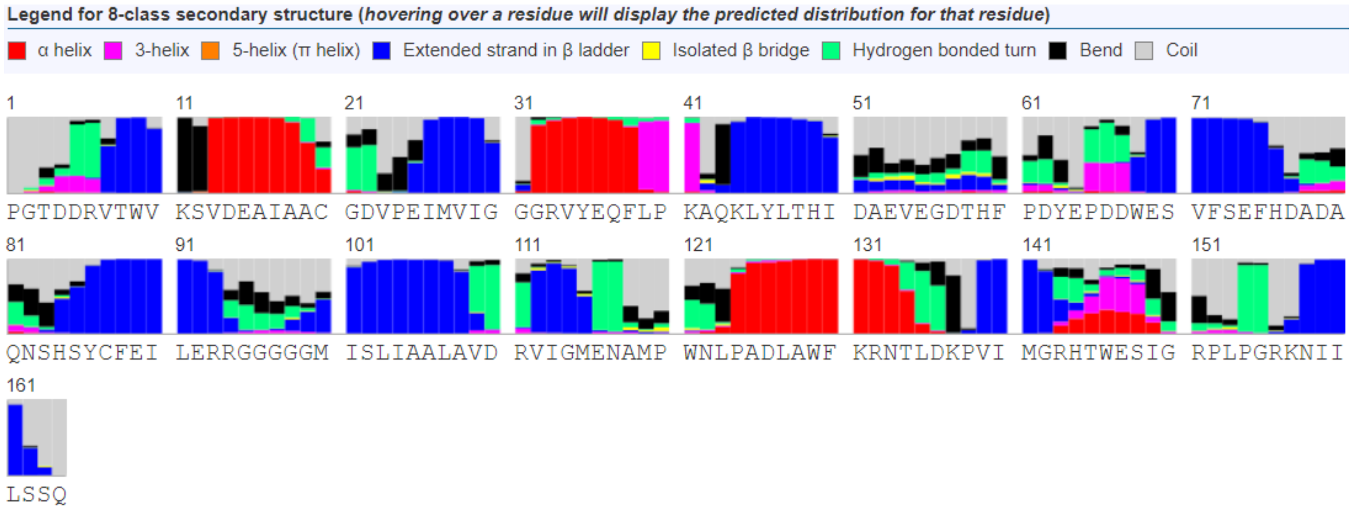

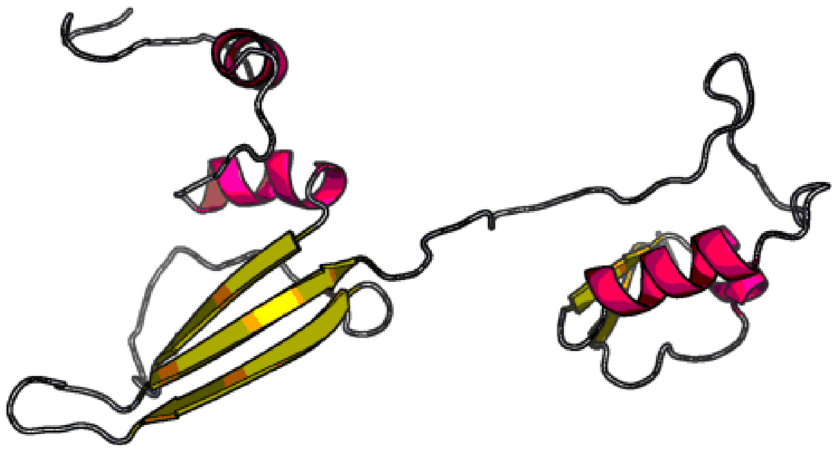

Template: PDB 1rx4A

CP site: Gly67

Target sequence:

GTDDRVTWVKSVDEAIAACGDVPEIMVIGGGRVYEQFLPKAQKLYLTHIDAEVEGDTHFP
DYEPDDWESVFSEFHDADAQNSHSYCFEILERRGGGGGMISLIAALAVDRVIGMENAMPW
NLPADLAWFKRNTLDKPVIMGRHTWESIGRPLPGRKNIILSSQP

Summary

- The input predicted as **2** domain(s)
- Best template: **5uioA**, p-value **2.95e-06**
- Overall uGDT (GDT): **166** (**101**)
- **164**(**100%**) residues are modeled
- **9**(**5%**) positions predicted as disordered
- Secondary struct: **17%**H, **32%**E, **50%**C
- Solvent access: **33%**E, **32%**M, **33%**B

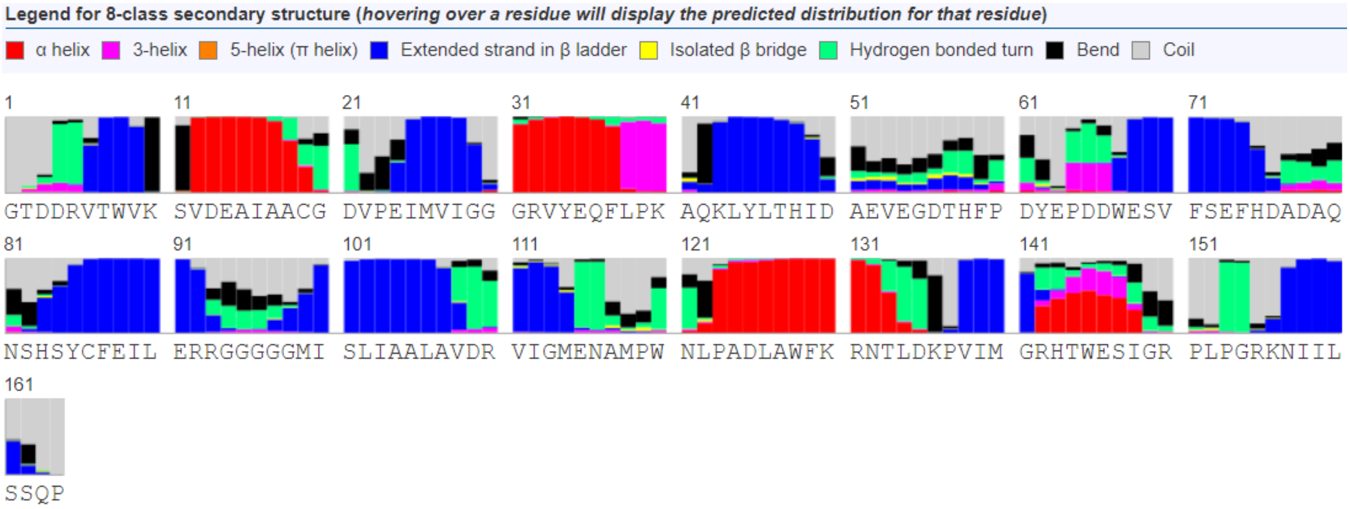

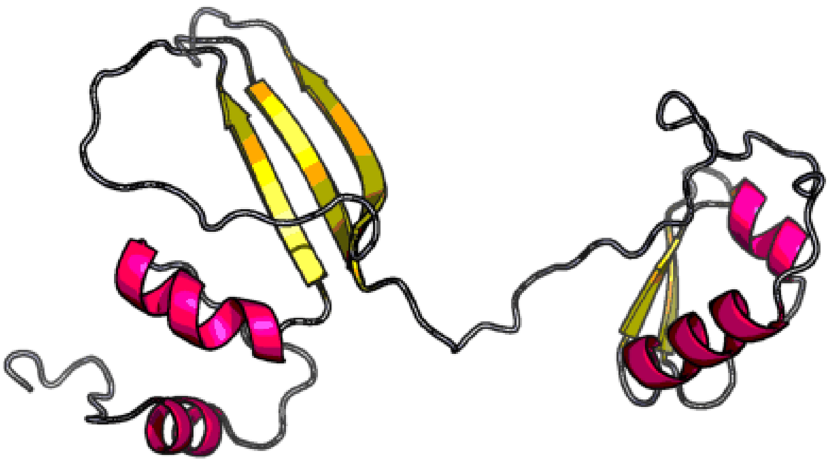

Template: PDB 1rx4A

CP site: Thr68

Target sequence:

TDDRVTWVKSVDEAIAACGDVPEIMVIGGGRVYEQFLPKAQKLYLTHIDAEVEGDTHFPD
YEPDDWESVFSEFHDADAQNSHSYCFEILERRGGGGGMISLIAALAVDRVIGMENAMPWN
LPADLAWFKRNTLDKPVIMGRHTWESIGRPLPGRKNIILSSQPG

Summary

- The input predicted as **2** domain(s)
- Best template: **5uioA**, p-value **3.44e-06**
- Overall uGDT (GDT): **165** (**100**)
- **164**(**100%**) residues are modeled
- **6**(**3%**) positions predicted as disordered
- Secondary struct: **15%**H, **32%**E, **51%**C
- Solvent access: **32%**E, **33%**M, **34%**B

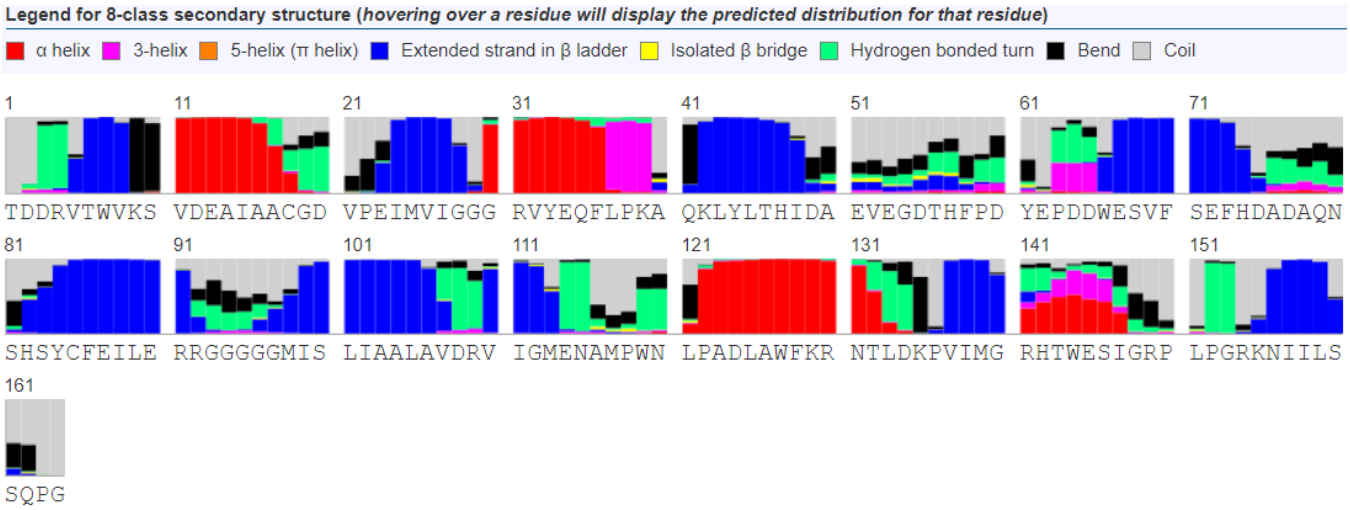

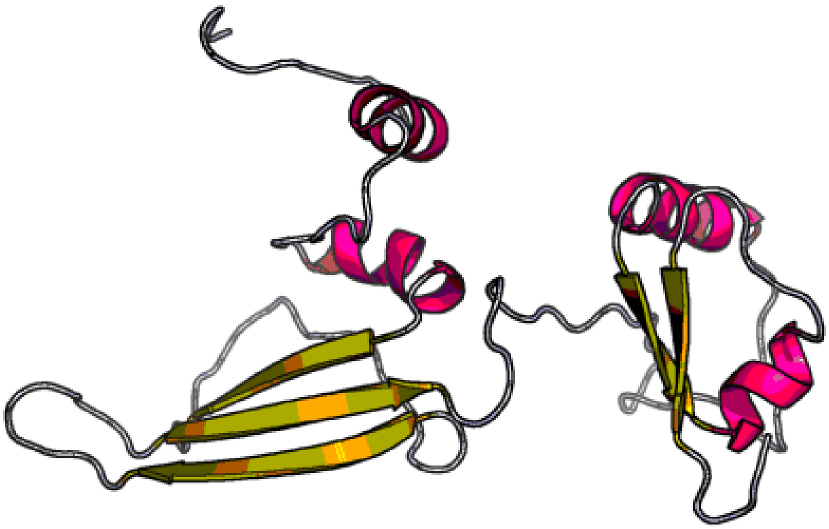

Template: PDB 1rx4A

CP site: Asp69

Target sequence:

DDRVTWVKSVDEAIAACGDVPEIMVIGGGRVYEQFLPKAQKLYLTHIDAEVEGDTHFPDY
EPDDWESVFSEFHDADAQNSHSYCFEILERRGGGGGMISLIAALAVDRVIGMENAMPWNL
PADLAWFKRNTLDKPVIMGRHTWESIGRPLPGRKNIILSSQPGT

Summary

•The input predicted as **2** domain(s)
•Best template: **1dreA**, p-value **2.06e-06**
•Overall uGDT (GDT): **165** (**100**)
•**164**(**100%**) residues are modeled
•**7**(**4%**) positions predicted as disordered
•Secondary struct: **18%**H, **32%**E, **49%**C
•Solvent access: **34%**E, **33%**M, **32%**B

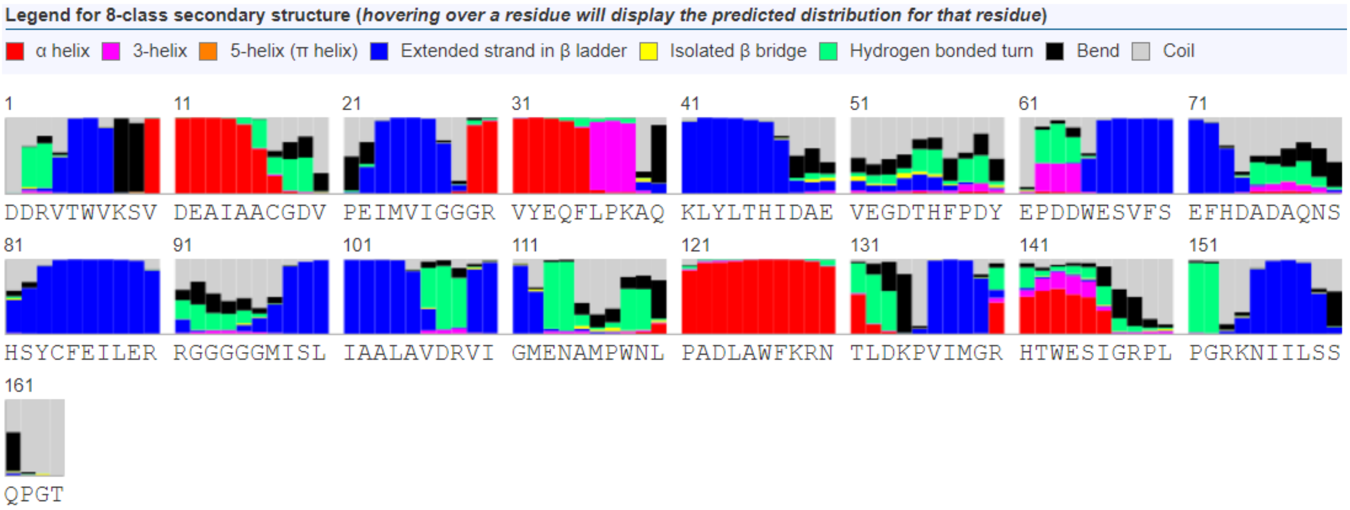

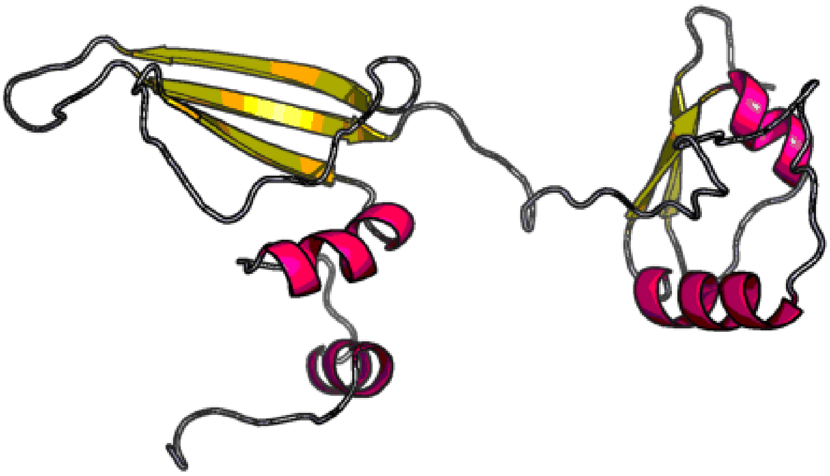

Template: PDB 1rx4A

CP site: Asp70

Target sequence:

DRVTWVKSVDEAIAACGDVPEIMVIGGGRVYEQFLPKAQKLYLTHIDAEVEGDTHFPDYE
PDDWESVFSEFHDADAQNSHSYCFEILERRGGGGGMISLIAALAVDRVIGMENAMPWNLP
ADLAWFKRNTLDKPVIMGRHTWESIGRPLPGRKNIILSSQPGTD

Summary

•The input predicted as **2** domain(s)
•Best template: **1dreA**, p-value **1.89e-06**
•Overall uGDT (GDT): **164** (**100**)
•**164**(**100%**) residues are modeled
•**8**(**4%**) positions predicted as disordered
•Secondary struct: **15%**H, **32%**E, **52%**C
•Solvent access: **32%**E, **36%**M, **31%**B

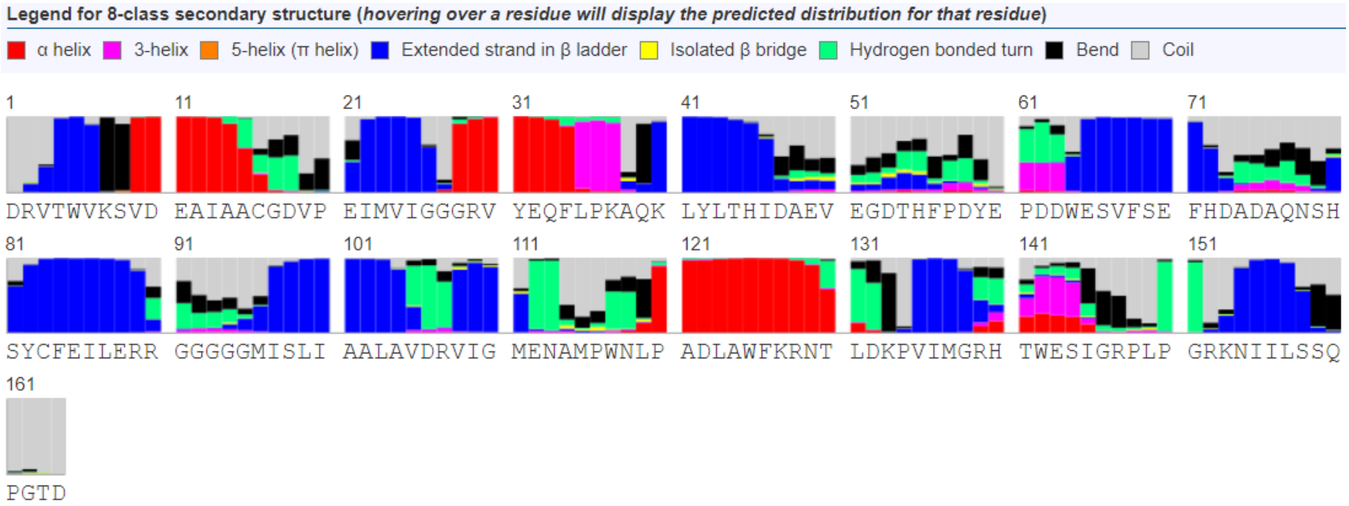

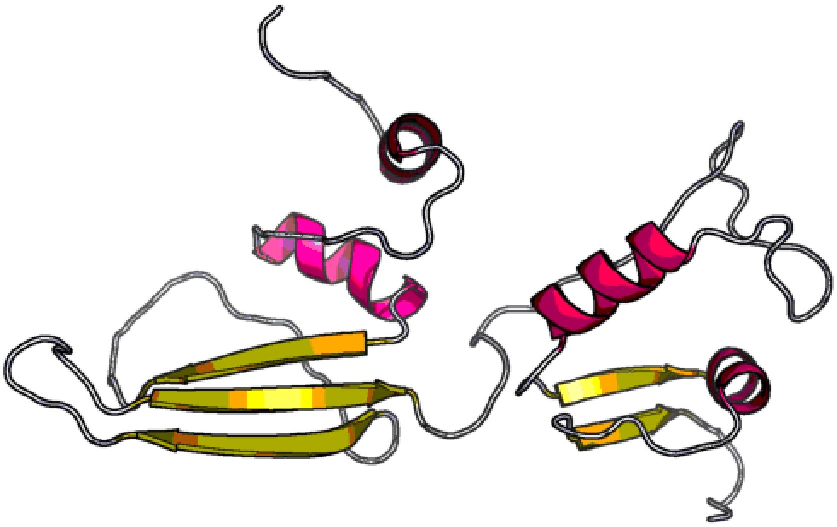

Template: PDB 1rx4A

CP site: Arg71

Target sequence:

RVTWVKSVDEAIAACGDVPEIMVIGGGRVYEQFLPKAQKLYLTHIDAEVEGDTHFPDYEP
DDWESVFSEFHDADAQNSHSYCFEILERRGGGGGMISLIAALAVDRVIGMENAMPWNLPA
DLAWFKRNTLDKPVIMGRHTWESIGRPLPGRKNIILSSQPGTDD

Summary

- The input predicted as **2** domain(s)
- Best template: **1dreA**, p-value **1.79e-06**
- Overall uGDT (GDT): **164** (**100**)
- **164**(**100%**) residues are modeled
- **8**(**4%**) positions predicted as disordered
- Secondary struct: **15%**H, **31%**E, **53%**C
- Solvent access: **33%**E, **34%**M, **32%**B

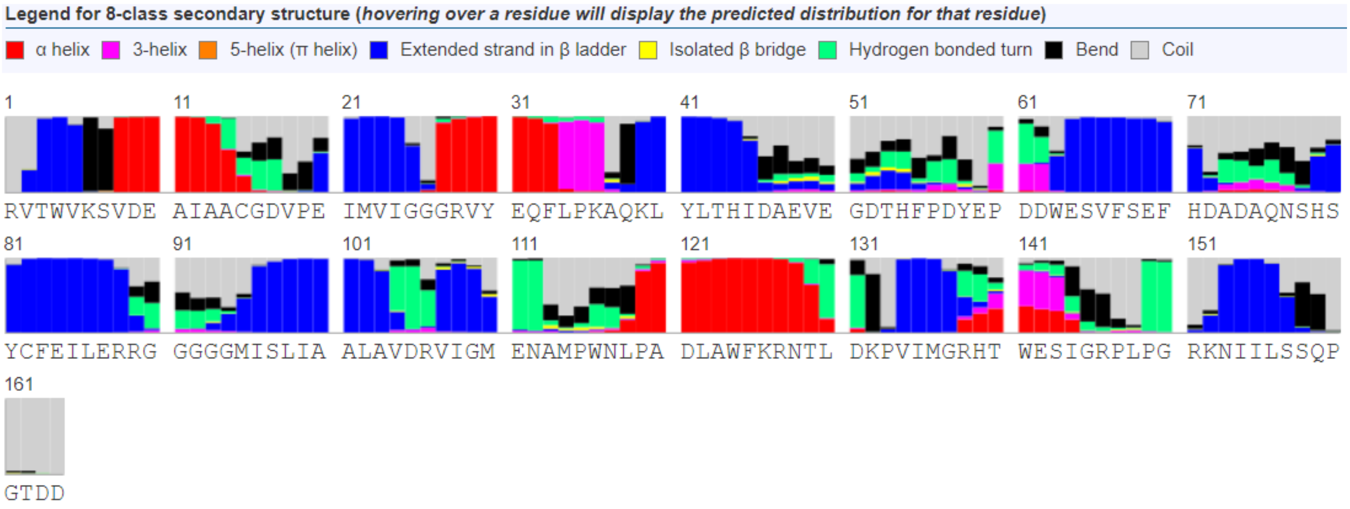

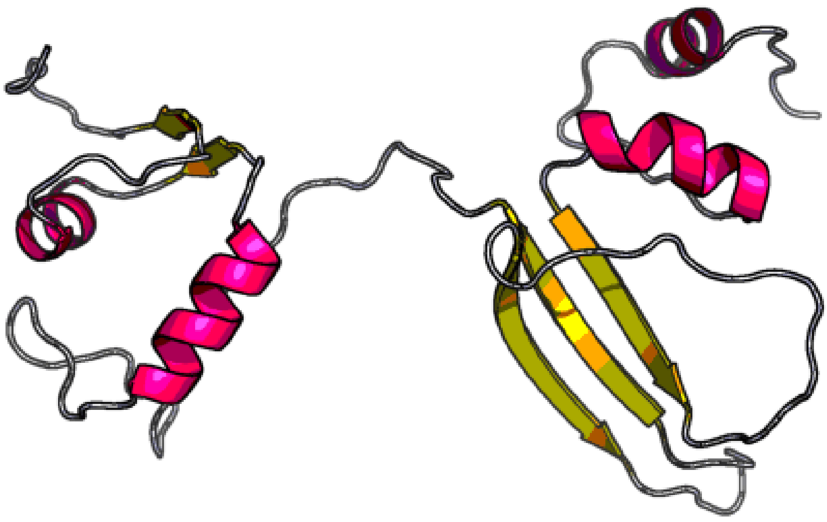

Template: PDB 1rx4A

CP site: Val72

Target sequence:

VTWVKSVDEAIAACGDVPEIMVIGGGRVYEQFLPKAQKLYLTHIDAEVEGDTHFPDYEPD
DWESVFSEFHDADAQNSHSYCFEILERRGGGGGMISLIAALAVDRVIGMENAMPWNLPAD
LAWFKRNTLDKPVIMGRHTWESIGRPLPGRKNIILSSQPGTDDR

Summary

- The input predicted as **2** domain(s)
- Best template: **1tdrA**, p-value **2.32e-06**
- Overall uGDT (GDT): **163** (**99**)
- **164**(**100%**) residues are modeled
- **10**(**6%**) positions predicted as disordered
- Secondary struct: **15%**H, **32%**E, **52%**C
- Solvent access: **34%**E, **31%**M, **33%**B

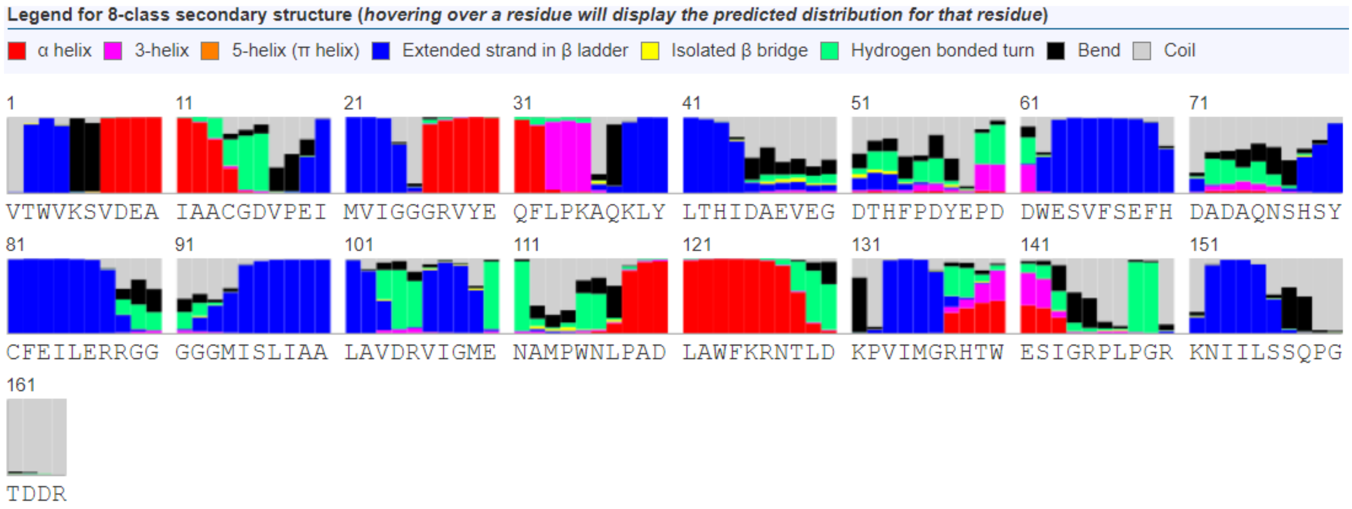

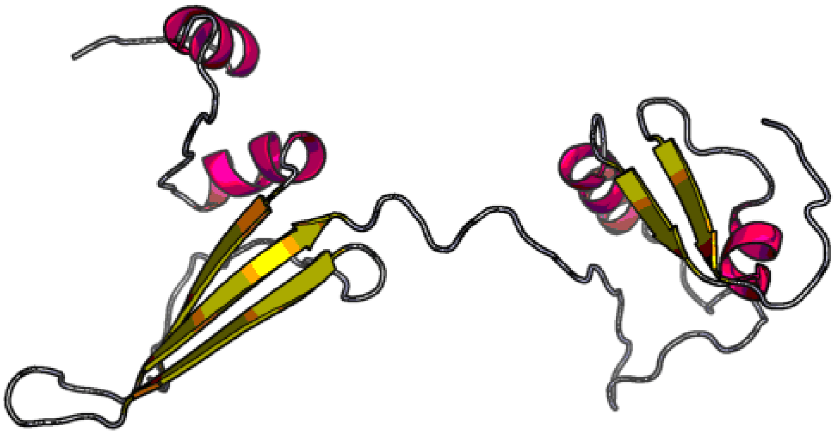

Template: PDB 1rx4A

CP site: Thr73

Target sequence:

TWVKSVDEAIAACGDVPEIMVIGGGRVYEQFLPKAQKLYLTHIDAEVEGDTHFPDYEPDD
WESVFSEFHDADAQNSHSYCFEILERRGGGGGMISLIAALAVDRVIGMENAMPWNLPADL
AWFKRNTLDKPVIMGRHTWESIGRPLPGRKNIILSSQPGTDDRV

Summary

- The input predicted as **2** domain(s)
- Best template: **1dreA**, p-value **2.06e-06**
- Overall uGDT (GDT): **163** (**99**)
- **164**(**100%**) residues are modeled
- **11**(**6%**) positions predicted as disordered
- Secondary struct: **15%**H, **30%**E, **54%**C
- Solvent access: **36%**E, **32%**M, **30%**B

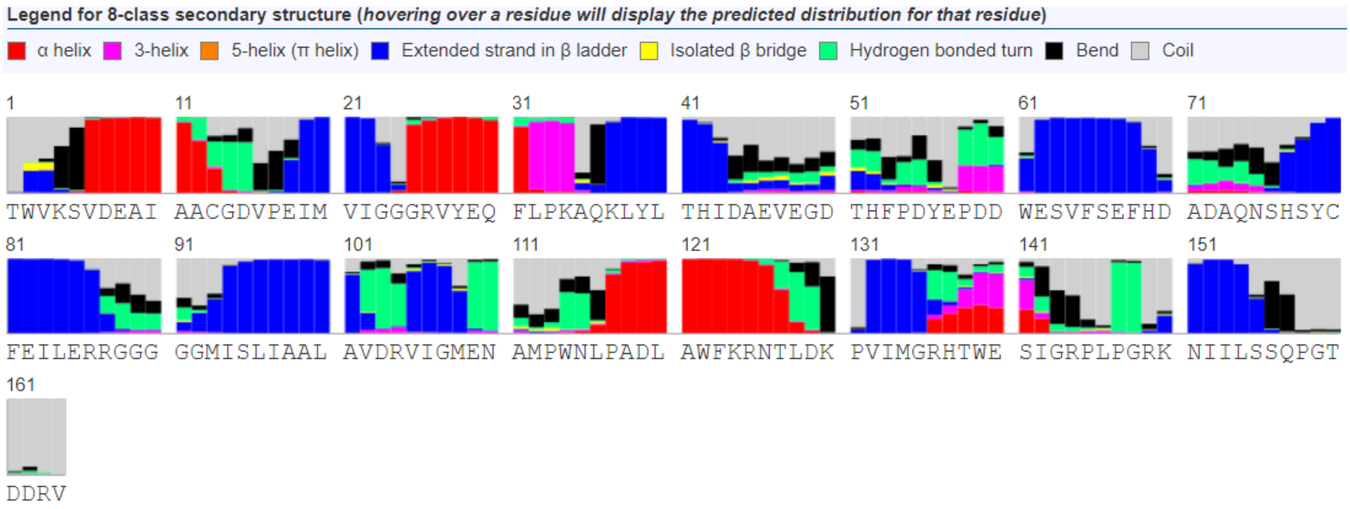

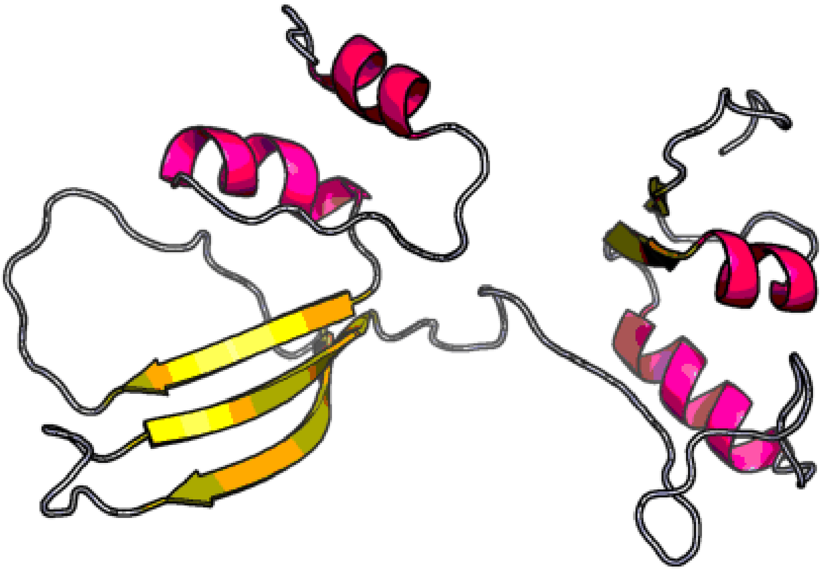

Template: PDB 1rx4A

CP site: Trp74

Target sequence:

```
WVKSVDEAIAACGDVPEIMVIGGGRVYEQFLPKAQKLYLTHIDAEVEGDTHFPDYEPDDW
ESVFSEFHDADAQNSHSYCFEILERRGGGGGMISLIAALAVDRVIGMENAMPWNLPADLA
WFKRNTLDKPVIMGRHTWESIGRPLPGRKNIILSSQPGTDDRVT
```

Summary

- The input predicted as **2** domain(s)
- Best template: **1tdrA**, p-value **3.20e-06**
- Overall uGDT (GDT): **163** (**99**)
- **164**(**100%**) residues are modeled
- **11**(**6%**) positions predicted as disordered
- Secondary struct: **15%**H, **31%**E, **53%**C
- Solvent access: **35%**E, **32%**M, **32%**B

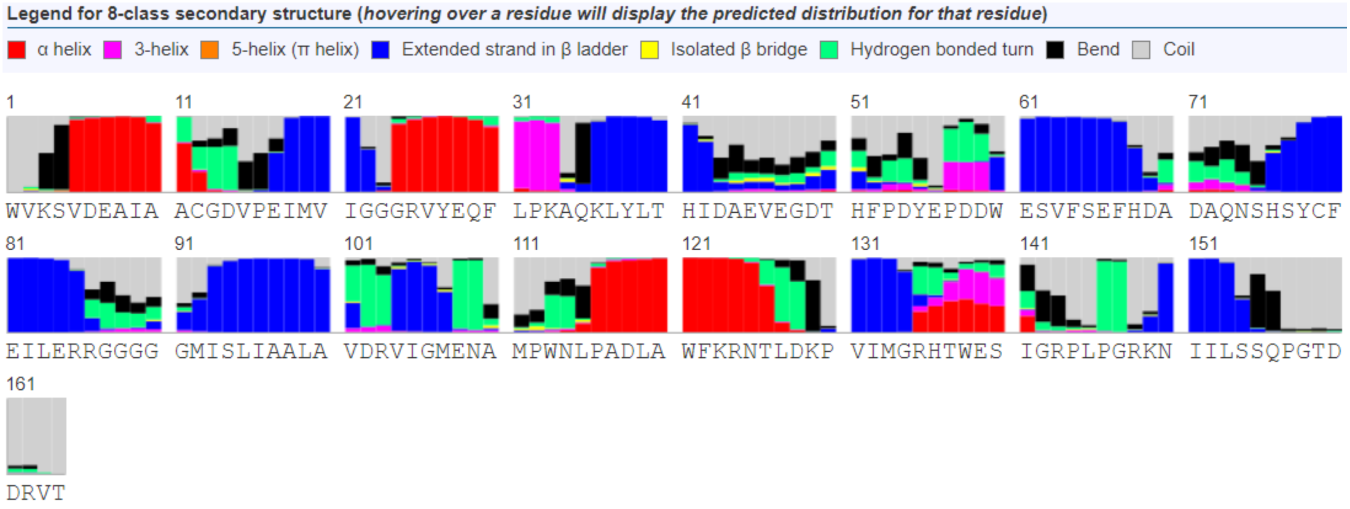

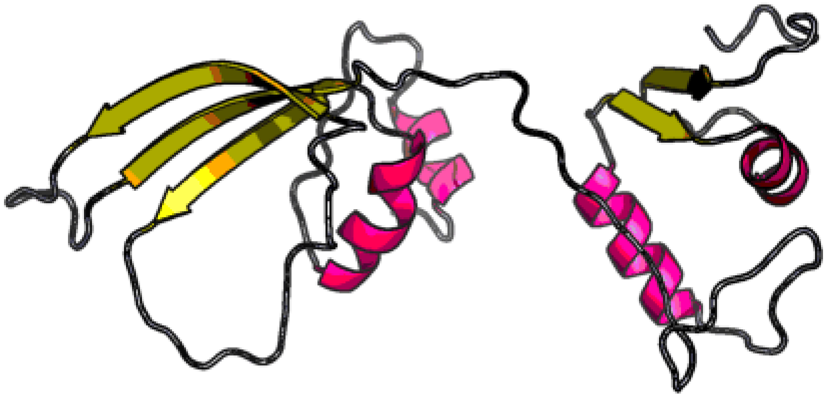

Template: PDB 1rx4A

CP site: Val75

Target sequence:

VKSVDEAIAACGDVPEIMVIGGGRVYEQFLPKAQKLYLTHIDAEVEGDTHFPDYEPDDWE
SVFSEFHDADAQNSHSYCFEILERRGGGGGMISLIAALAVDRVIGMENAMPWNLPADLAW
FKRNTLDKPVIMGRHTWESIGRPLPGRKNIILSSQPGTDDRVTW

Summary

- The input predicted as **2** domain(s)
- Best template: **1tdrA**, p-value **1.60e-06**
- Overall uGDT (GDT): **163** (**99**)
- **164**(**100%**) residues are modeled
- **11**(**6%**) positions predicted as disordered
- Secondary struct: **17%**H, **32%**E, **50%**C
- Solvent access: **34%**E, **32%**M, **32%**B

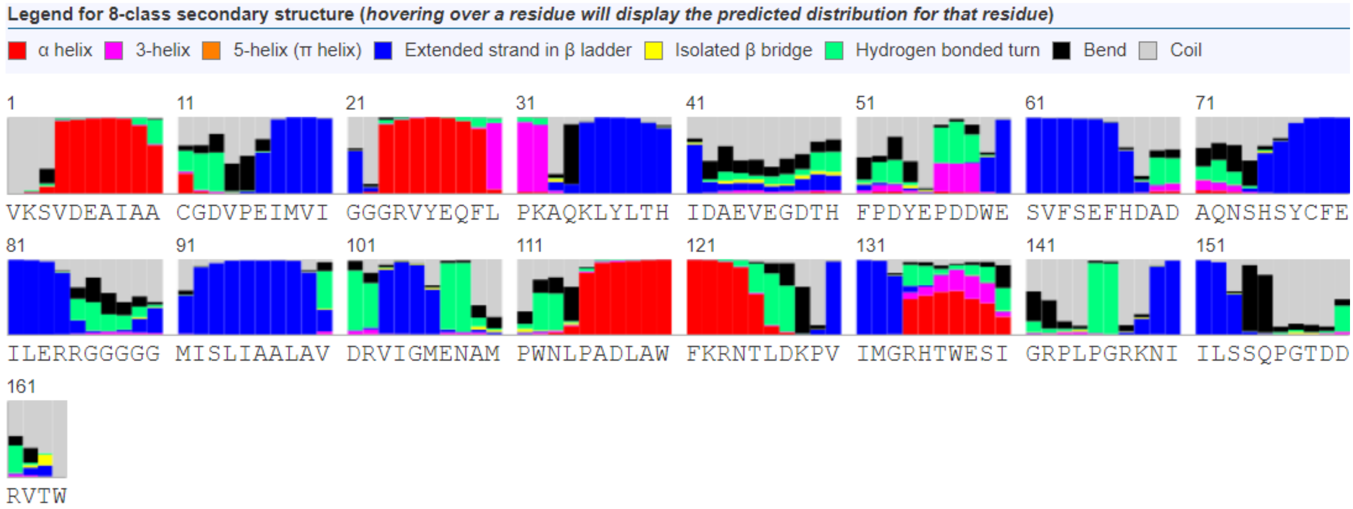

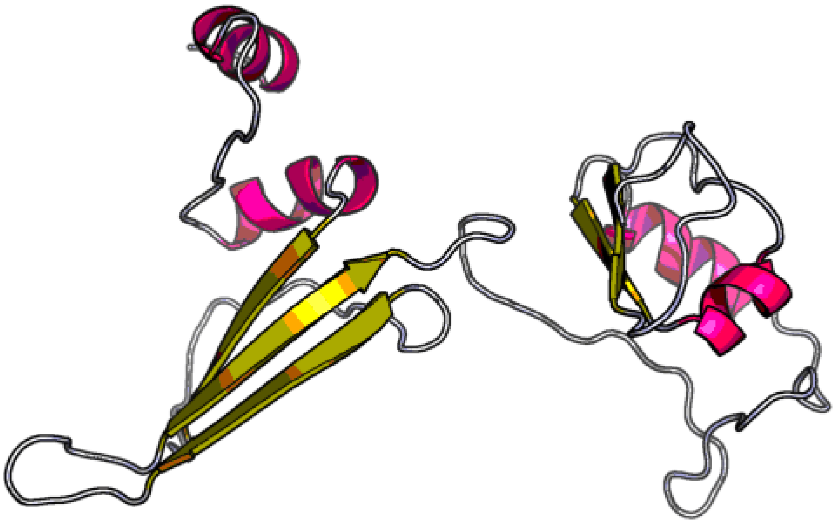

Template: PDB 1rx4A

CP site: Lys76

Target sequence:

```
KSVDEAIAACGDVPEIMVIGGGRVYEQFLPKAQKLYLTHIDAEVEGDTHFPDYEPDDWES
VFSEFHDADAQNSHSYCFEILERRGGGGGMISLIAALAVDRVIGMENAMPWNLPADLAWF
KRNTLDKPVIMGRHTWESIGRPLPGRKNIILSSQPGTDDRVTWV
```

Summary

- The input predicted as **2** domain(s)
- Best template: **1dreA**, p-value **8.70e-07**
- Overall uGDT (GDT): **165** (**101**)
- **164**(**100%**) residues are modeled
- **3**(**1%**) positions predicted as disordered
- Secondary struct: **16%**H, **32%**E, **50%**C
- Solvent access: **32%**E, **34%**M, **32%**B

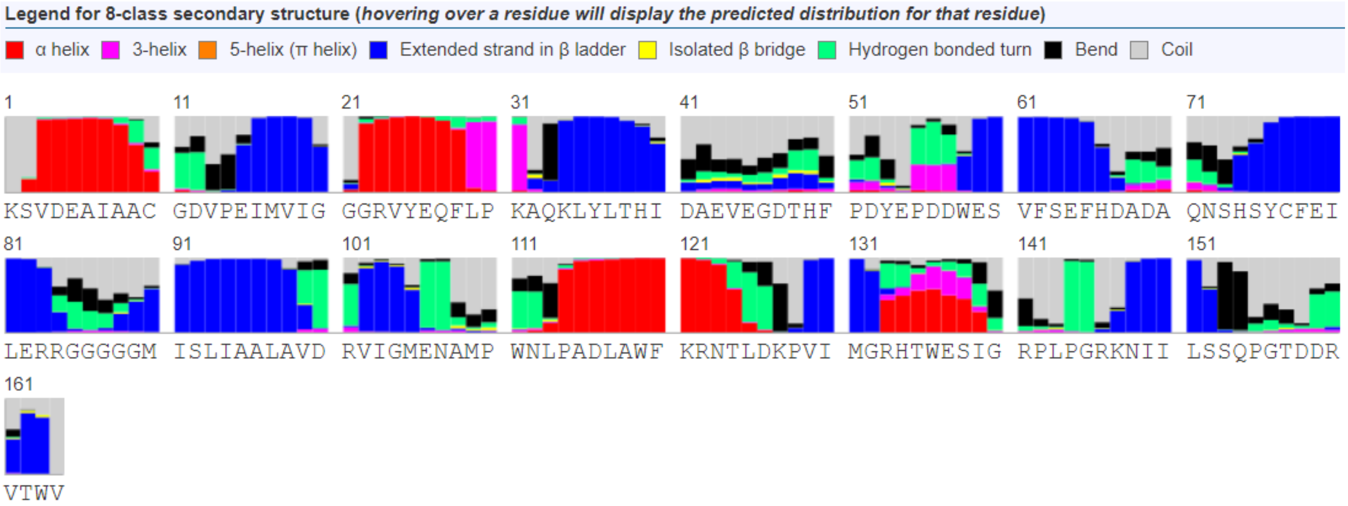

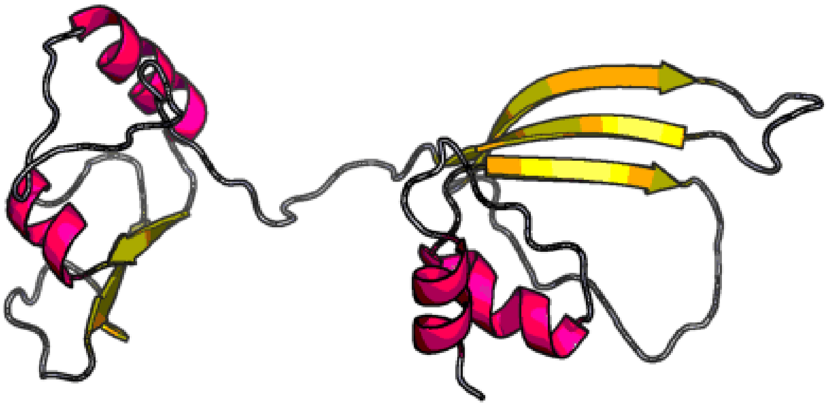

Template: PDB 1rx4A

CP site: Ser77

Target sequence:

SVDEAIAACGDVPEIMVIGGGRVYEQFLPKAQKLYLTHIDAEVEGDTHFPDYEPDDWESV
FSEFHDADAQNSHSYCFEILERRGGGGGMISLIAALAVDRVIGMENAMPWNLPADLAWFK
RNTLDKPVIMGRHTWESIGRPLPGRKNIILSSQPGTDDRVTWVK

Summary

- The input predicted as **2** domain(s)
- Best template: **1dreA**, p-value **6.20e-07**
- Overall uGDT (GDT): **166** (**101**)
- **164**(**100%**) residues are modeled
- **3**(**1%**) positions predicted as disordered
- Secondary struct: **17%**H, **33%**E, **48%**C
- Solvent access: **32%**E, **35%**M, **32%**B

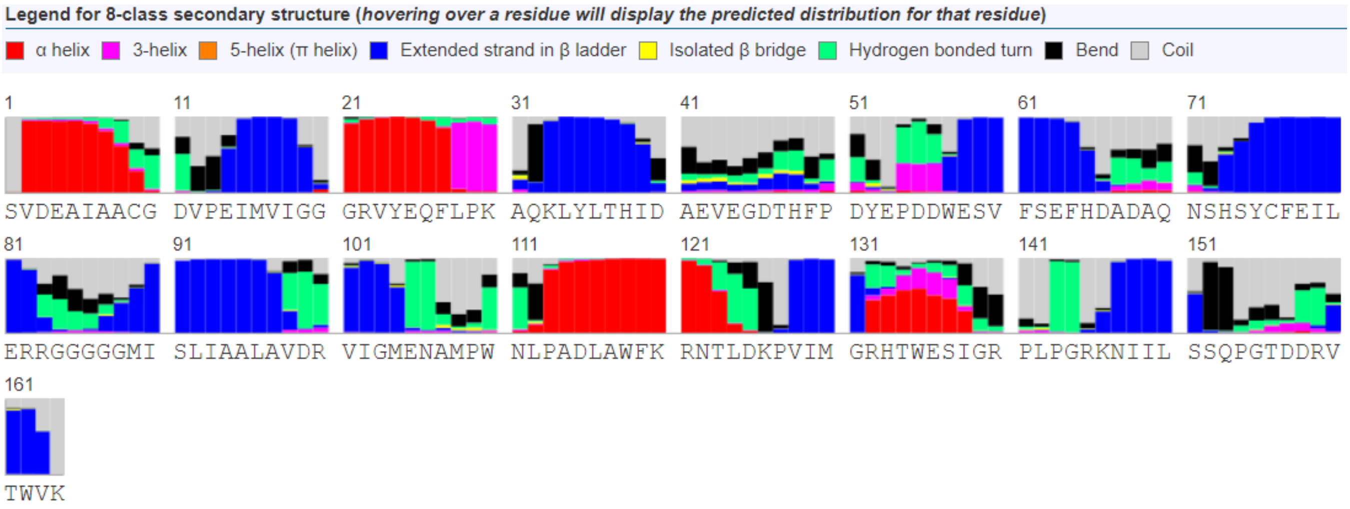

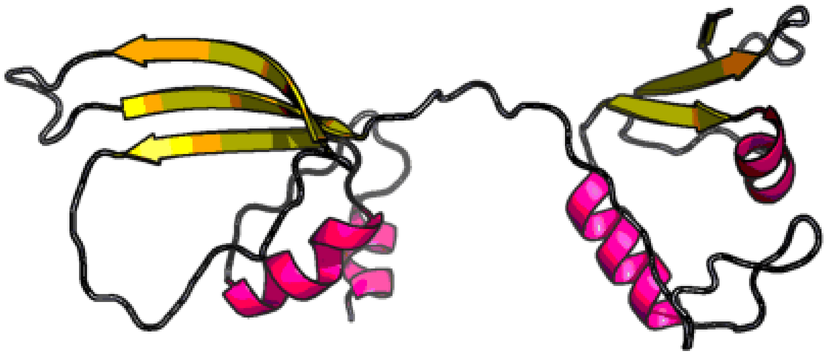

Template: PDB 1rx4A

CP site: Val78

Target sequence:

VDEAIAACGDVPEIMVIGGGRVYEQFLPKAQKLYLTHIDAEVEGDTHFPDYEPDDWESVF
SEFHDADAQNSHSYCFEILERRGGGGGMISLIAALAVDRVIGMENAMPWNLPADLAWFKR
NTLDKPVIMGRHTWESIGRPLPGRKNIILSSQPGTDDRVTWVKS

Summary

- The input predicted as **2** domain(s)
- Best template: **1dreA**, p-value **7.66e-07**
- Overall uGDT (GDT): **169** (**103**)
- **164**(**100%**) residues are modeled
- **3**(**1%**) positions predicted as disordered
- Secondary struct: **17%**H, **34%**E, **48%**C
- Solvent access: **33%**E, **35%**M, **31%**B

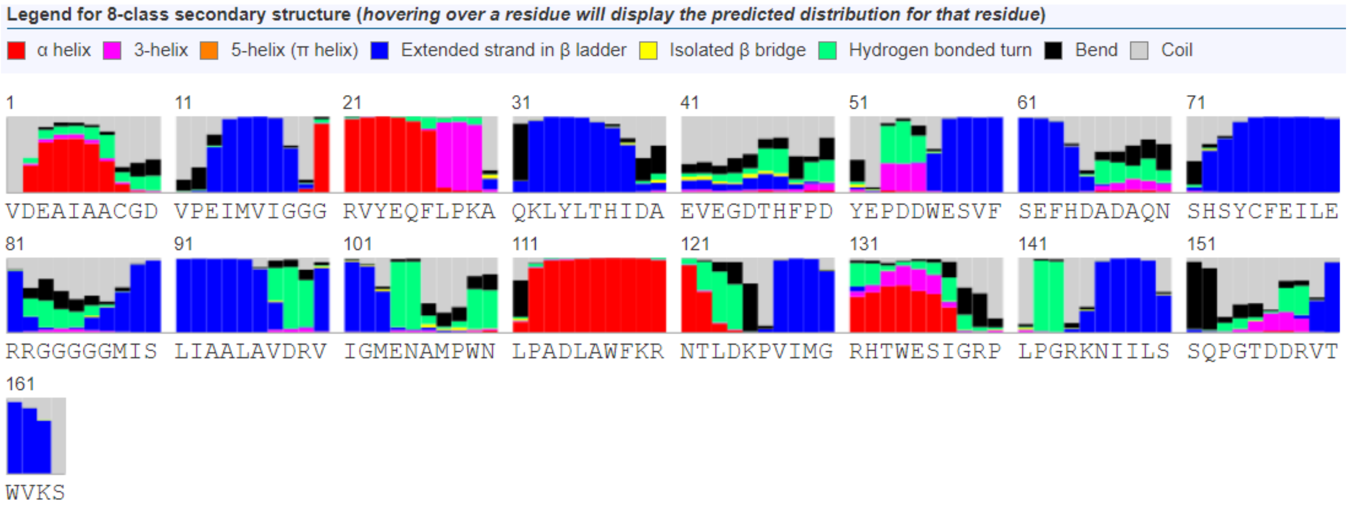

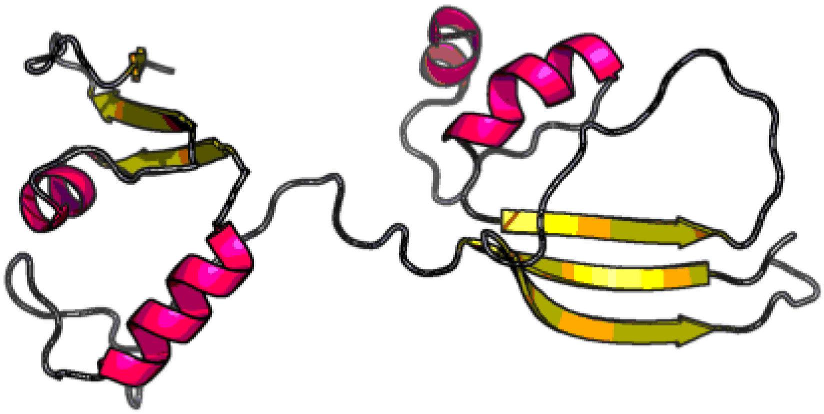

Template: PDB 1rx4A

CP site: Asp79

Target sequence:

DEAIAACGDVPEIMVIGGGRVYEQFLPKAQKLYLTHIDAEVEGDTHFPDYEPDDWESVFS
EFHDADAQNSHSYCFEILERRGGGGGMISLIAALAVDRVIGMENAMPWNLPADLAWFKRN
TLDKPVIMGRHTWESIGRPLPGRKNIILSSQPGTDDRVTWVKSV

Summary

- The input predicted as **2** domain(s)
- Best template: **5uioA**, p-value **2.73e-07**
- Overall uGDT (GDT): **162** (**98**)
- **164**(**100%**) residues are modeled
- **6**(**3%**) positions predicted as disordered
- Secondary struct: **16%**H, **35%**E, **48%**C
- Solvent access: **34%**E, **34%**M, **31%**B

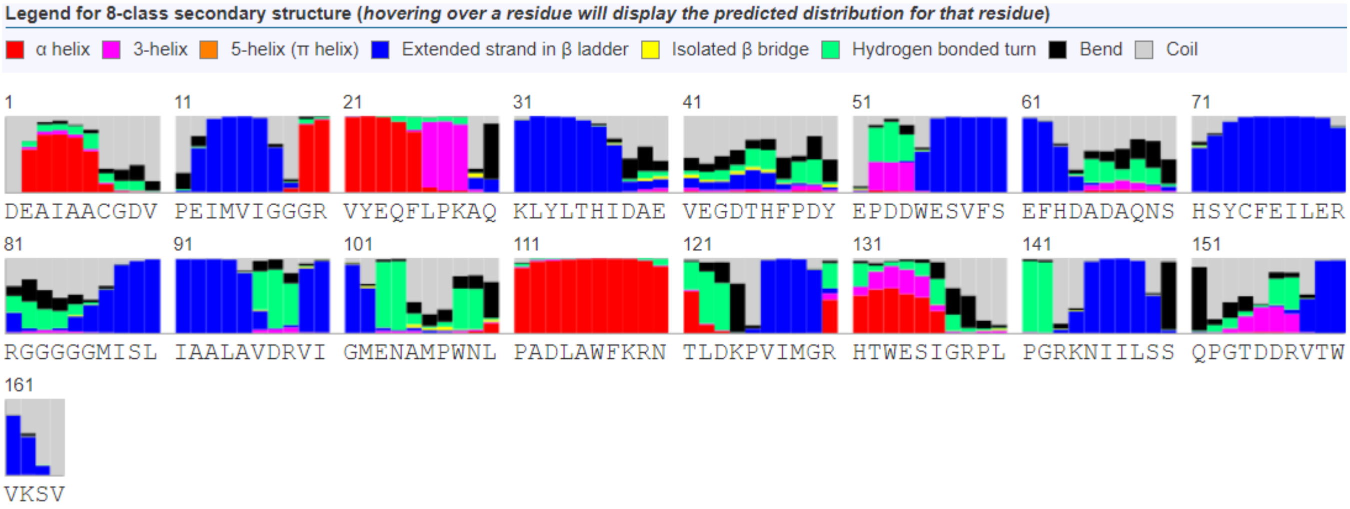

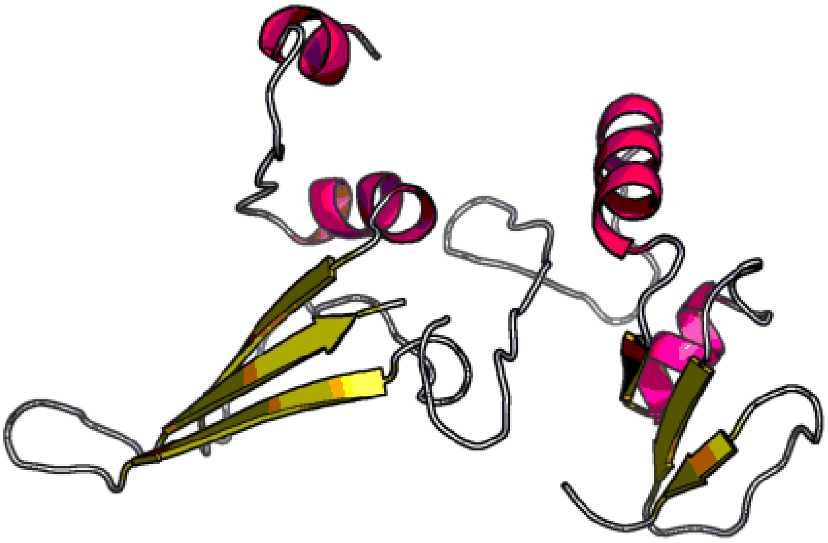

Template: PDB 1rx4A

CP site: Ala84

Target sequence:

```
ACGDVPEIMVIGGGRVYEQFLPKAQKLYLTHIDAEVEGDTHFPDYEPDDWESVFSEFHDA
DAQNSHSYCFEILERRGGGGGMISLIAALAVDRVIGMENAMPWNLPADLAWFKRNTLDKP
VIMGRHTWESIGRPLPGRKNIILSSQPGTDDRVTWVKSVDEAIA
```

Summary

- The input predicted as **2** domain(s)
- Best template: **5uioA**, p-value **7.88e-07**
- Overall uGDT (GDT): **166** (**101**)
- **164**(**100%**) residues are modeled
- **6**(**3%**) positions predicted as disordered
- Secondary struct: **17%**H, **34%**E, **48%**C
- Solvent access: **32%**E, **36%**M, **30%**B

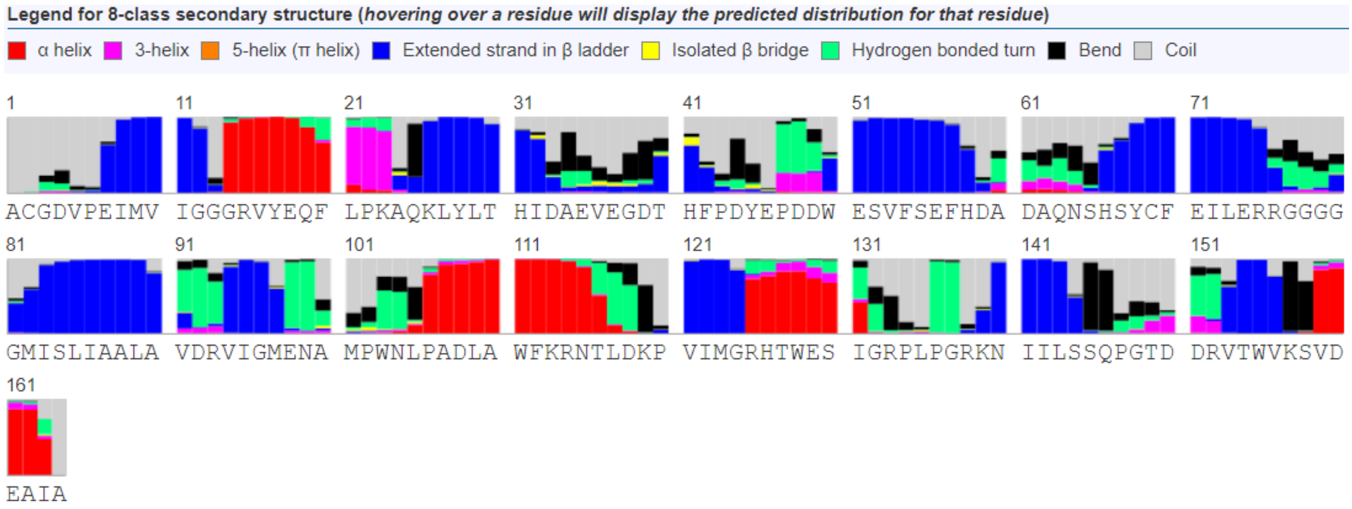

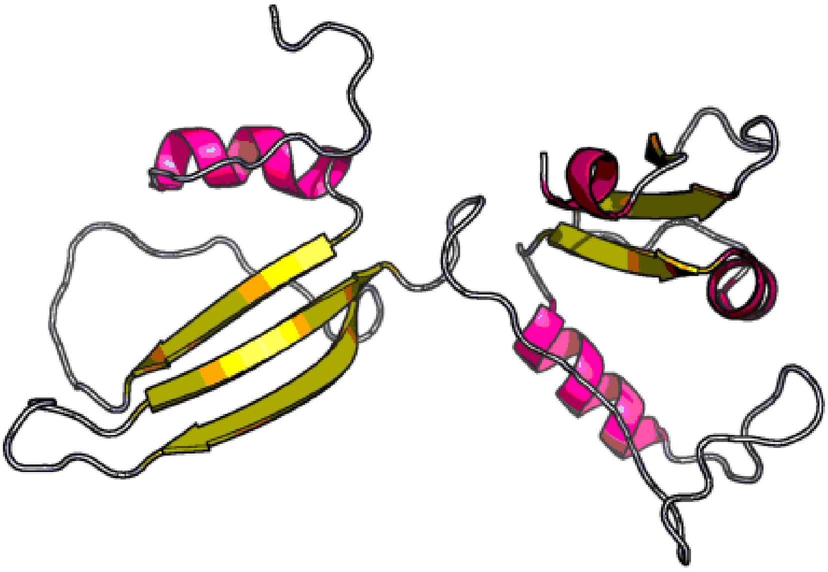

Template: PDB 1rx4A

CP site: Cys85

Target sequence:

CGDVPEIMVIGGGRVYEQFLPKAQKLYLTHIDAEVEGDTHFPDYEPDDWESVFSEFHDAD
AQNSHSYCFEILERRGGGGGMISLIAALAVDRVIGMENAMPWNLPADLAWFKRNTLDKPV
IMGRHTWESIGRPLPGRKNIILSSQPGTDDRVTWVKSVDEAIAA

Summary

- The input predicted as **2** domain(s)
- Best template: **5uioA**, p-value **6.16e-07**
- Overall uGDT (GDT): **165** (**101**)
- **164**(**100%**) residues are modeled
- **4**(**2%**) positions predicted as disordered
- Secondary struct: **18%**H, **35%**E, **46%**C
- Solvent access: **34%**E, **33%**M, **32%**B

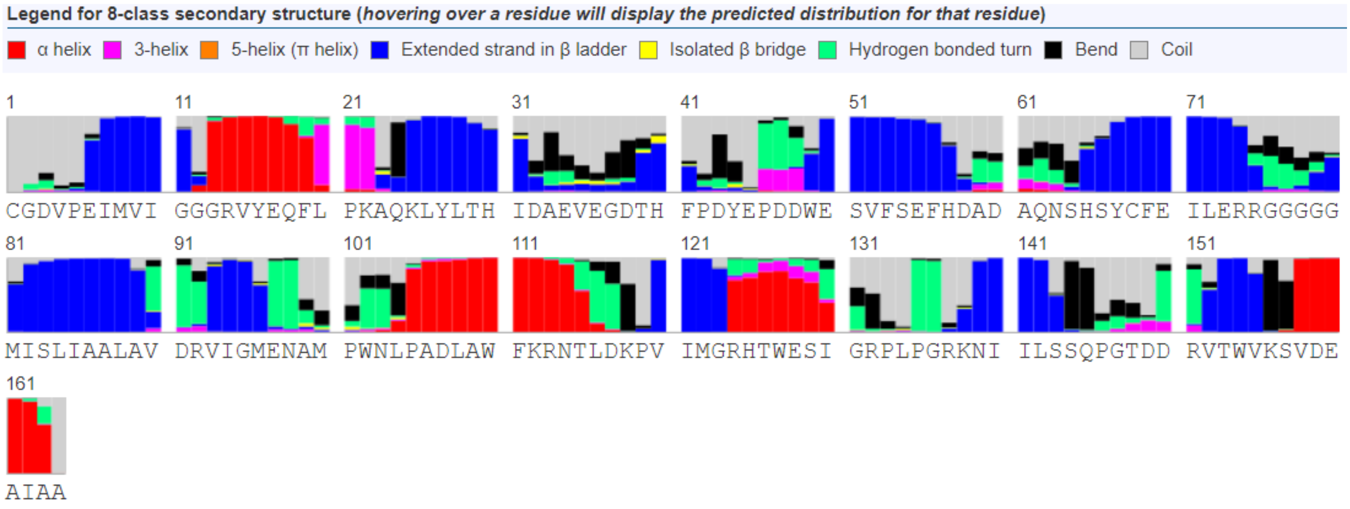

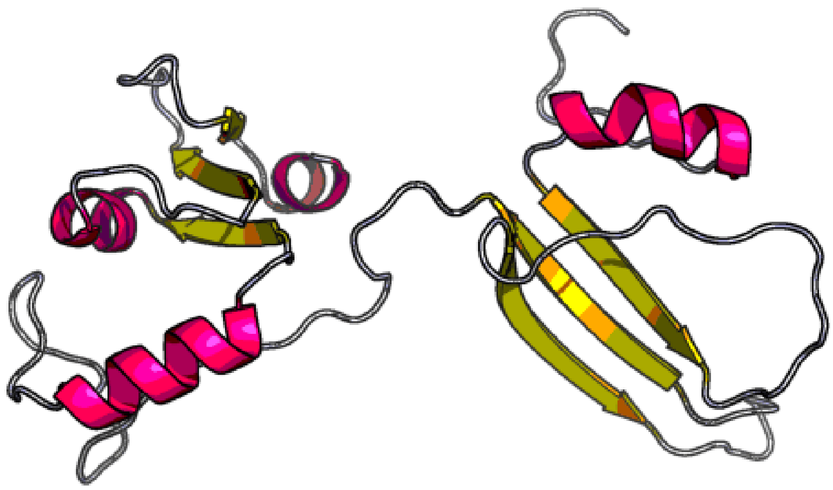

Template: PDB 1rx4A

CP site: Gly86

Target sequence:

GDVPEIMVIGGGRVYEQFLPKAQKLYLTHIDAEVEGDTHFPDYEPDDWESVFSEFHDADA
QNSHSYCFEILERRGGGGGMISLIAALAVDRVIGMENAMPWNLPADLAWFKRNTLDKPVI
MGRHTWESIGRPLPGRKNIILSSQPGTDDRVTWVKSVDEAIAAC

Summary

•The input predicted as **2** domain(s)
•Best template: **5uioA**, p-value **3.76e-07**
•Overall uGDT (GDT): **166** (**101**)
•**164**(**100%**) residues are modeled
•**6**(**3%**) positions predicted as disordered
•Secondary struct: **18%**H, **34%**E, **46%**C
•Solvent access: **32%**E, **33%**M, **33%**B

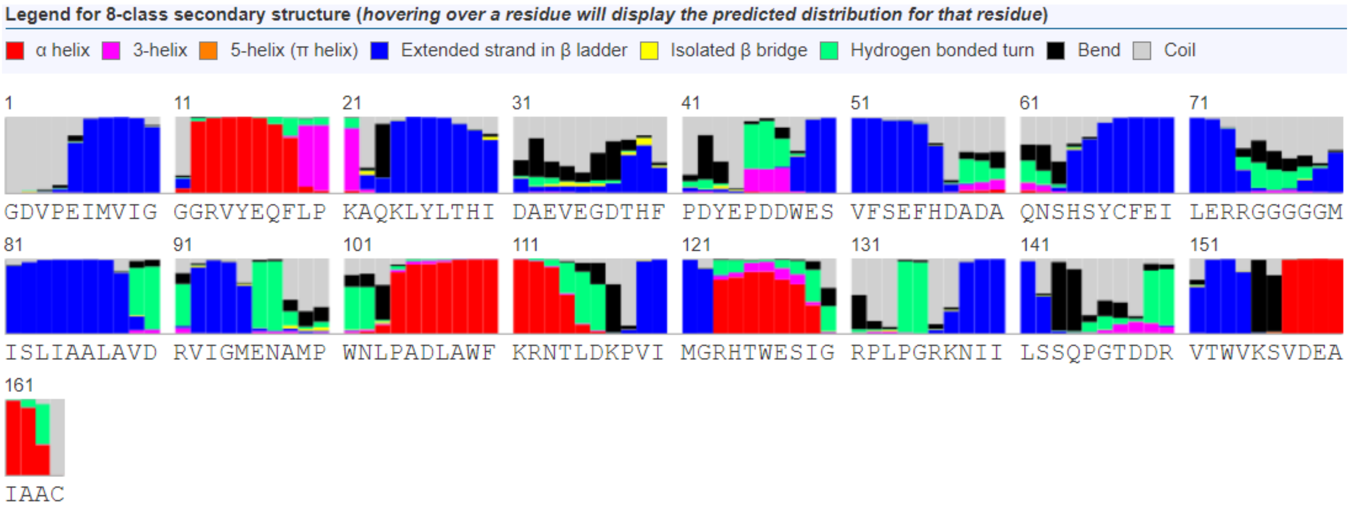

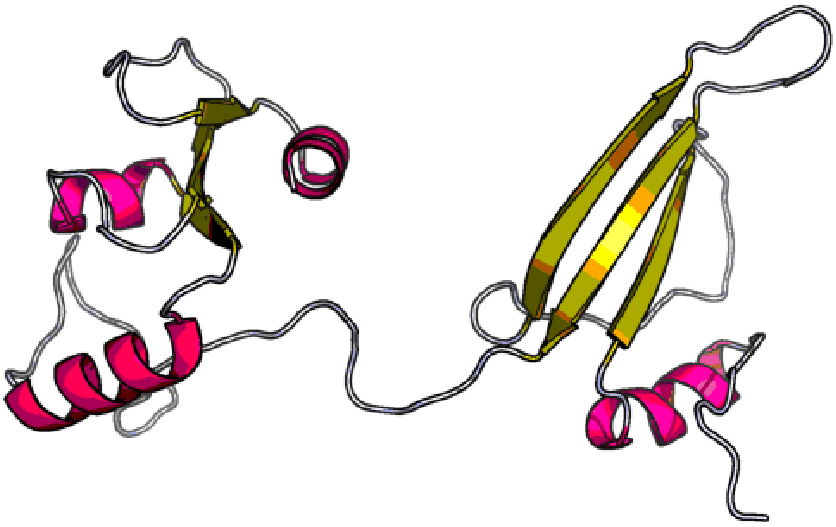

Template: PDB 1rx4A

CP site: Asp87

Target sequence:

DVPEIMVIGGGRVYEQFLPKAQKLYLTHIDAEVEGDTHFPDYEPDDWESVFSEFHDADAQ
NSHSYCFEILERRGGGGGMISLIAALAVDRVIGMENAMPWNLPADLAWFKRNTLDKPVIM
GRHTWESIGRPLPGRKNIILSSQPGTDDRVTWVKSVDEAIAACG

Summary

- The input predicted as **2** domain(s)
- Best template: **4qleA**, p-value **5.03e-06**
- Overall uGDT (GDT): **164** (**100**)
- **164**(**100%**) residues are modeled
- **2**(**1%**) positions predicted as disordered
- Secondary struct: **18%**H, **34%**E, **46%**C
- Solvent access: **33%**E, **33%**M, **32%**B

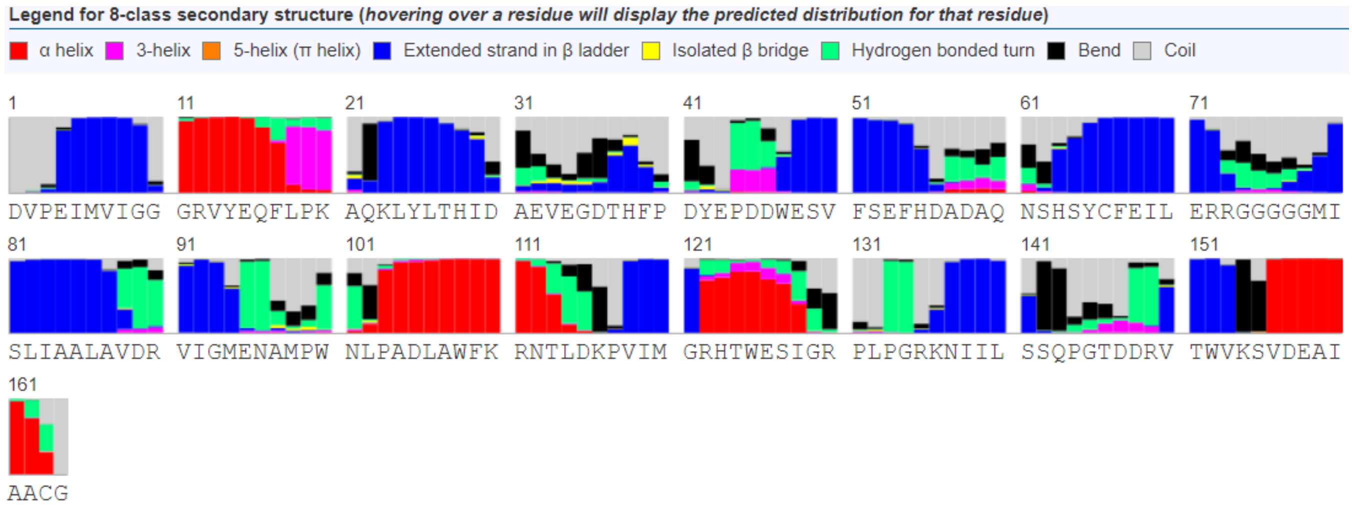

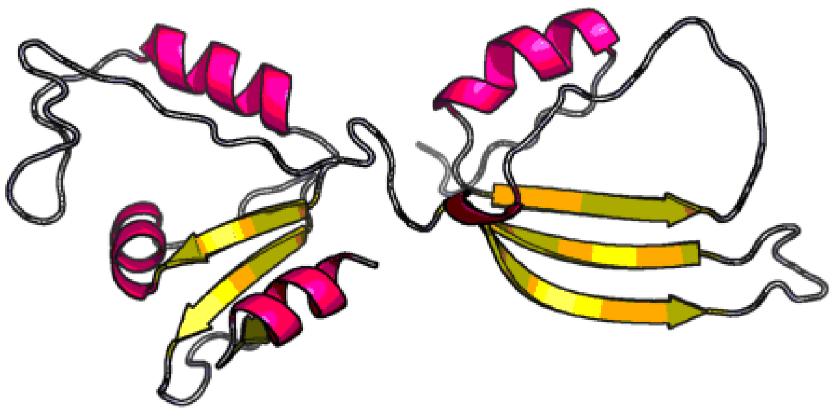

Template: PDB 1rx4A

CP site: Val88

Target sequence:

VPEIMVIGGGRVYEQFLPKAQKLYLTHIDAEVEGDTHFPDYEPDDWESVFSEFHDADAQN
SHSYCFEILERRGGGGGMISLIAALAVDRVIGMENAMPWNLPADLAWFKRNTLDKPVIMG
RHTWESIGRPLPGRKNIILSSQPGTDDRVTWVKSVDEAIAACGD

Summary

•The input predicted as **2** domain(s)
•Best template: **4qleA**, p-value **4.13e-06**
•Overall uGDT (GDT): **166** (**101**)
•**164**(**100%**) residues are modeled
•**5**(**3%**) positions predicted as disordered
•Secondary struct: **18%**H, **35%**E, **45%**C
•Solvent access: **32%**E, **34%**M, **32%**B

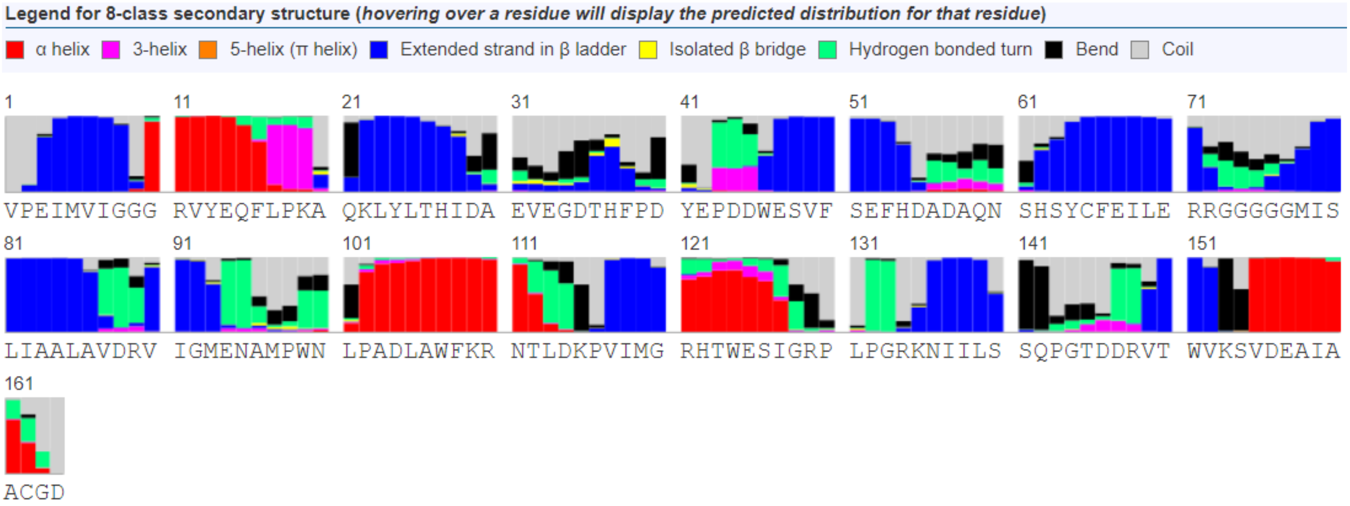

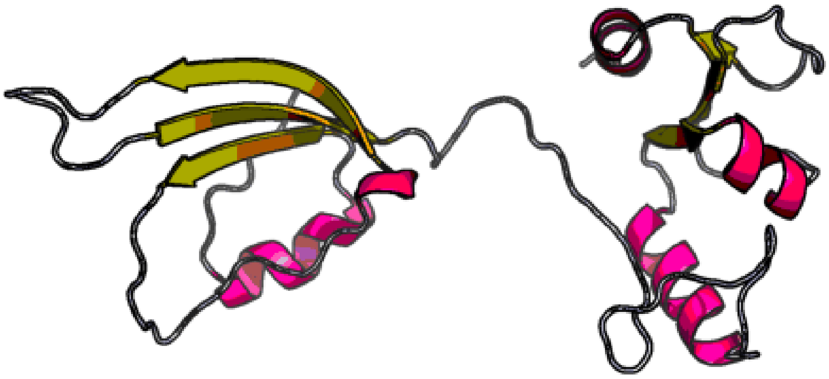

Template: PDB 1rx4A

CP site: Pro89

Target sequence:

PEIMVIGGGRVYEQFLPKAQKLYLTHIDAEVEGDTHFPDYEPDDWESVFSEFHDADAQNS
HSYCFEILERRGGGGGMISLIAALAVDRVIGMENAMPWNLPADLAWFKRNTLDKPVIMGR
HTWESIGRPLPGRKNIILSSQPGTDDRVTWVKSVDEAIAACGDV

Summary

- The input predicted as **2** domain(s)
- Best template: **5uioA**, p-value **4.14e-07**
- Overall uGDT (GDT): **164** (**100**)
- **164**(**100%**) residues are modeled
- **5**(**3%**) positions predicted as disordered
- Secondary struct: **19%**H, **35%**E, **44%**C
- Solvent access: **34%**E, **31%**M, **34%**B

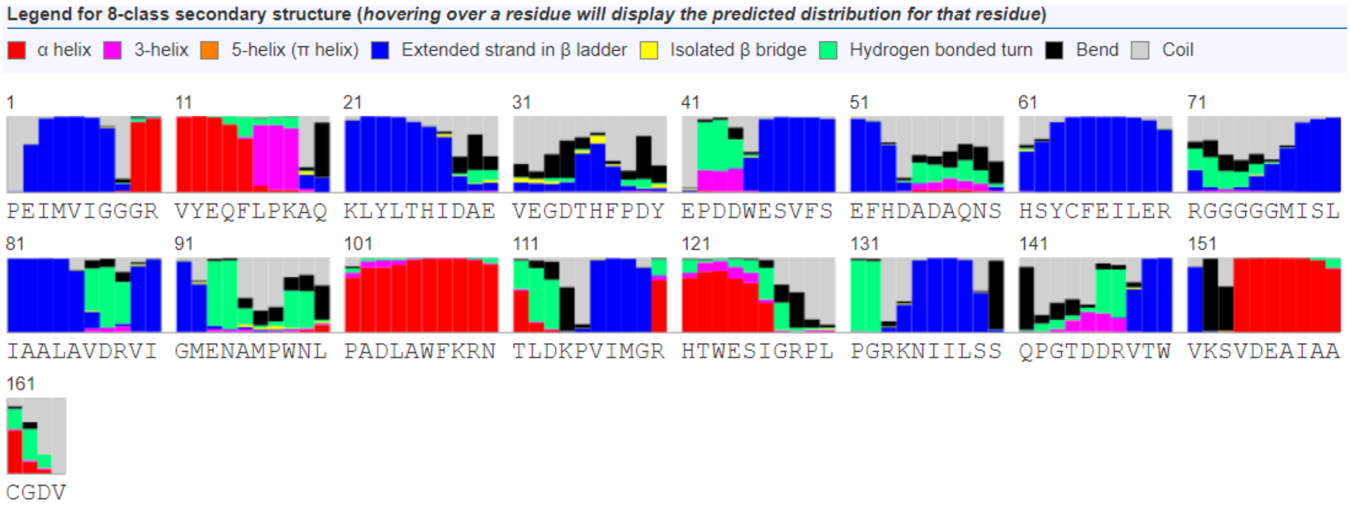

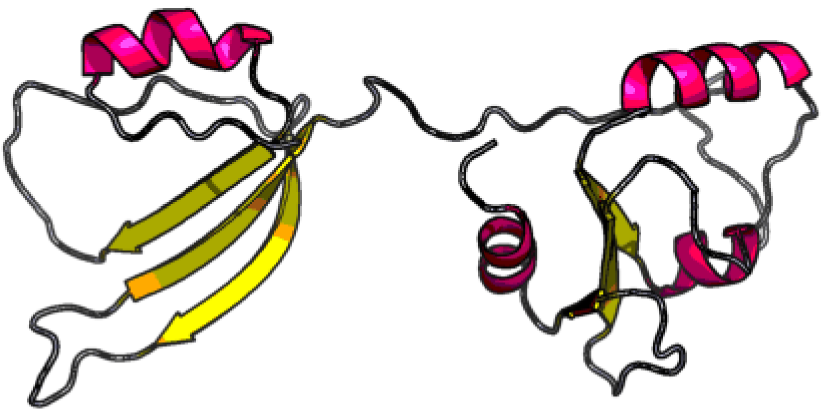

Template: PDB 1rx4A

CP site: Glu90

Target sequence:

EIMVIGGGRVYEQFLPKAQKLYLTHIDAEVEGDTHFPDYEPDDWESVFSEFHDADAQNSH
SYCFEILERRGGGGGMISLIAALAVDRVIGMENAMPWNLPADLAWFKRNTLDKPVIMGRH
TWESIGRPLPGRKNIILSSQPGTDDRVTWVKSVDEAIAACGDVP

Summary

- The input predicted as **2** domain(s)
- Best template: **5uioA**, p-value **2.58e-07**
- Overall uGDT (GDT): **164** (**100**)
- **164**(**100%**) residues are modeled
- **8**(**4%**) positions predicted as disordered
- Secondary struct: **19%**H, **33%**E, **46%**C
- Solvent access: **32%**E, **34%**M, **32%**B

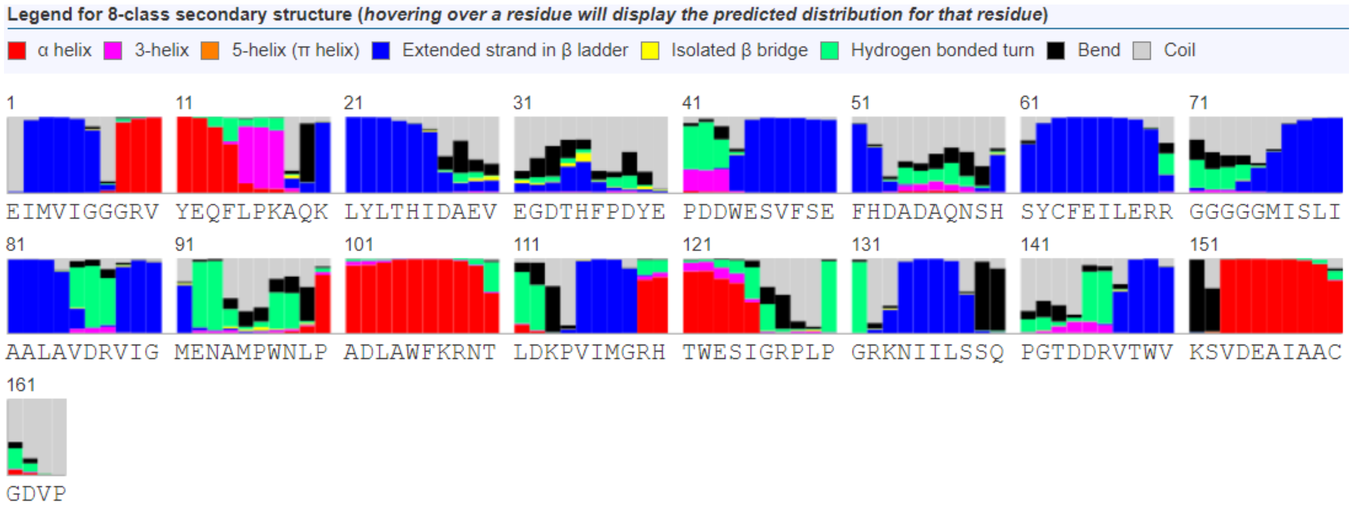

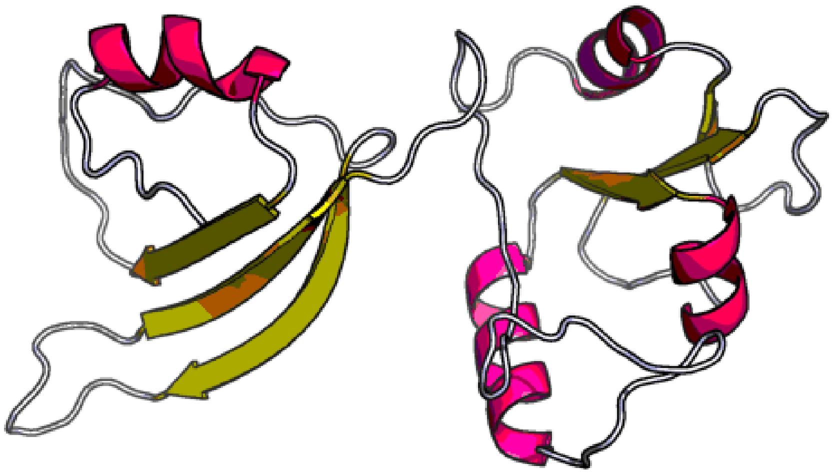

Template: PDB 1rx4A

CP site: Pro105

Target sequence:

```
PKAQKLYLTHIDAEVEGDTHFPDYEPDDWESVFSEFHDADAQNSHSYCFEILERRGGGGG
MISLIAALAVDRVIGMENAMPWNLPADLAWFKRNTLDKPVIMGRHTWESIGRPLPGRKNI
ILSSQPGTDDRVTWVKSVDEAIAACGDVPEIMVIGGGRVYEQFL
```

Summary

- The input predicted as **2** domain(s)
- Best template: **1dreA**, p-value **8.22e-07**
- Overall uGDT (GDT): **165** (**100**)
- **164**(**100%**) residues are modeled
- **5**(**3%**) positions predicted as disordered
- Secondary struct: **18%**H, **32%**E, **48%**C
- Solvent access: **35%**E, **34%**M, **29%**B

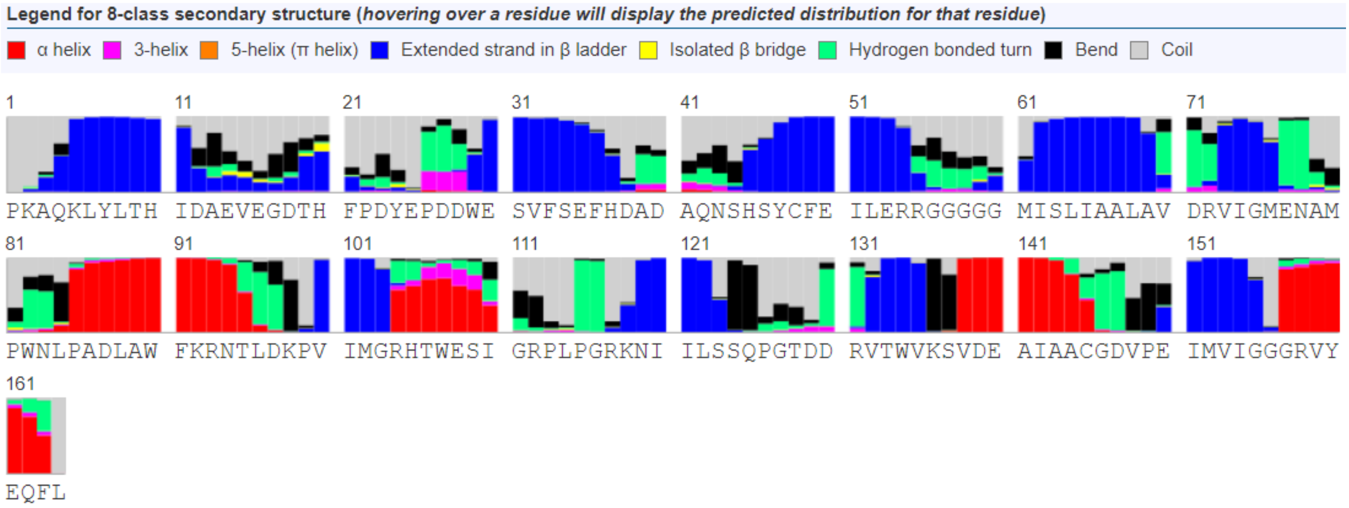

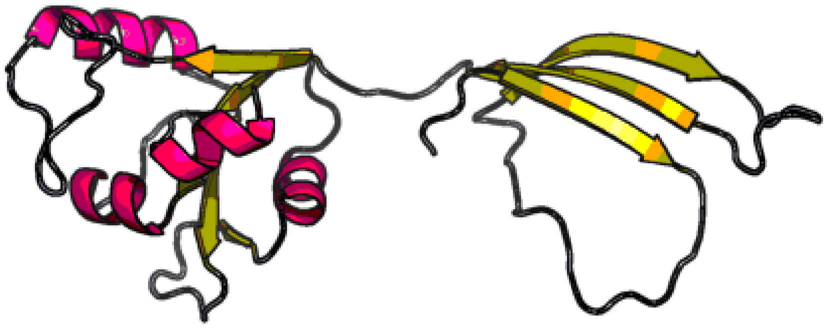

Template: PDB 1rx4A

CP site: Lys106

Target sequence:

```
KAQKLYLTHIDAEVEGDTHFPDYEPDDWESVFSEFHDADAQNSHSYCFEILERRGGGGGM
ISLIAALAVDRVIGMENAMPWNLPADLAWFKRNTLDKPVIMGRHTWESIGRPLPGRKNII
LSSQPGTDDRVTWVKSVDEAIAACGDVPEIMVIGGGRVYEQFLP
```

Summary

- The input predicted as **2** domain(s)
- Best template: **1dreA**, p-value **1.46e-06**
- Overall uGDT (GDT): **166** (**101**)
- **164**(**100%**) residues are modeled
- **4**(**2%**) positions predicted as disordered
- Secondary struct: **18%**H, **32%**E, **48%**C
- Solvent access: **34%**E, **32%**M, **32%**B

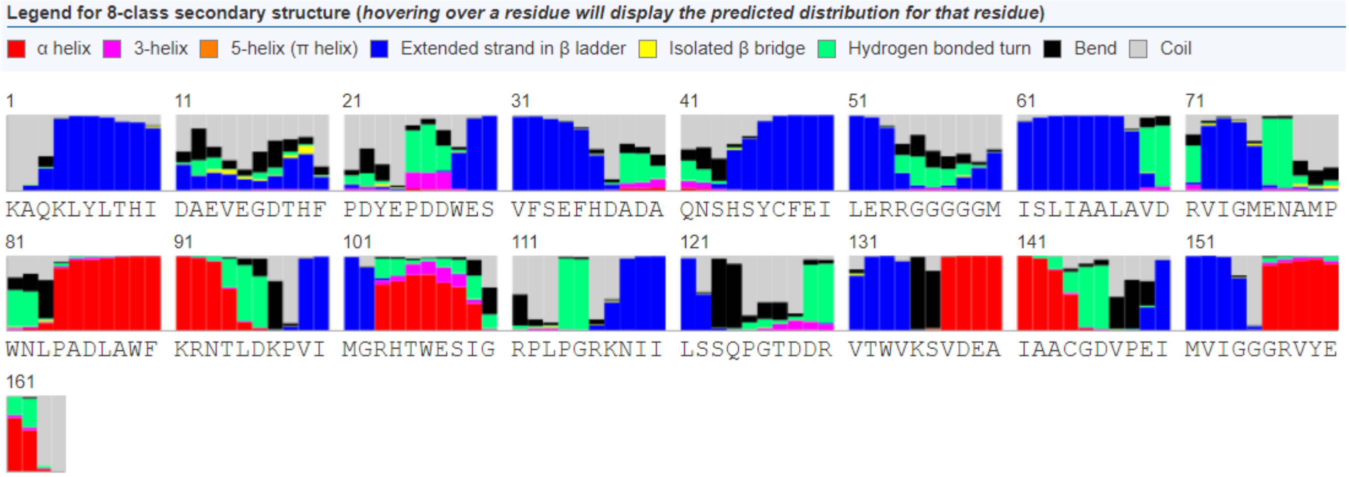

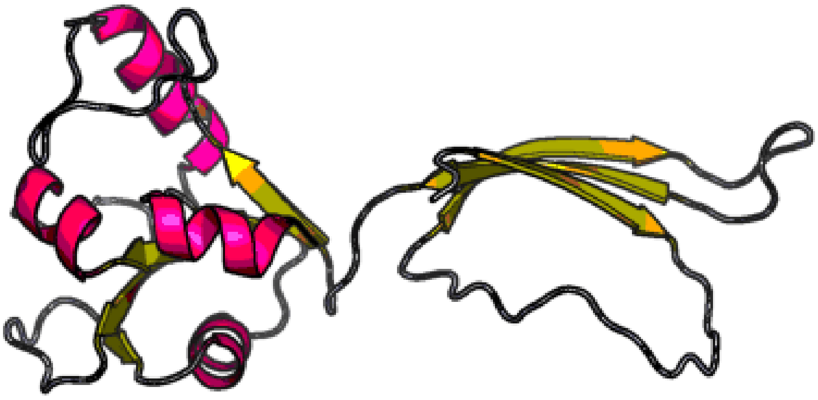

Template: PDB 1rx4A

CP site: Val119

Target sequence:

VEGDTHFPDYEPDDWESVFSEFHDADAQNSHSYCFEILERRGGGGGMISLIAALAVDRVI
GMENAMPWNLPADLAWFKRNTLDKPVIMGRHTWESIGRPLPGRKNIILSSQPGTDDRVTW
VKSVDEAIAACGDVPEIMVIGGGRVYEQFLPKAQKLYLTHIDAE

Summary

- The input predicted as **1** domain(s)
- Best template: **1dreA**, p-value **2.88e-07**
- Overall uGDT (GDT): **121** (**74**)
- **164**(**100%**) residues are modeled
- **6**(**3%**) positions predicted as disordered
- Secondary struct: **18%**H, **32%**E, **48%**C
- Solvent access: **36%**E, **31%**M, **32%**B

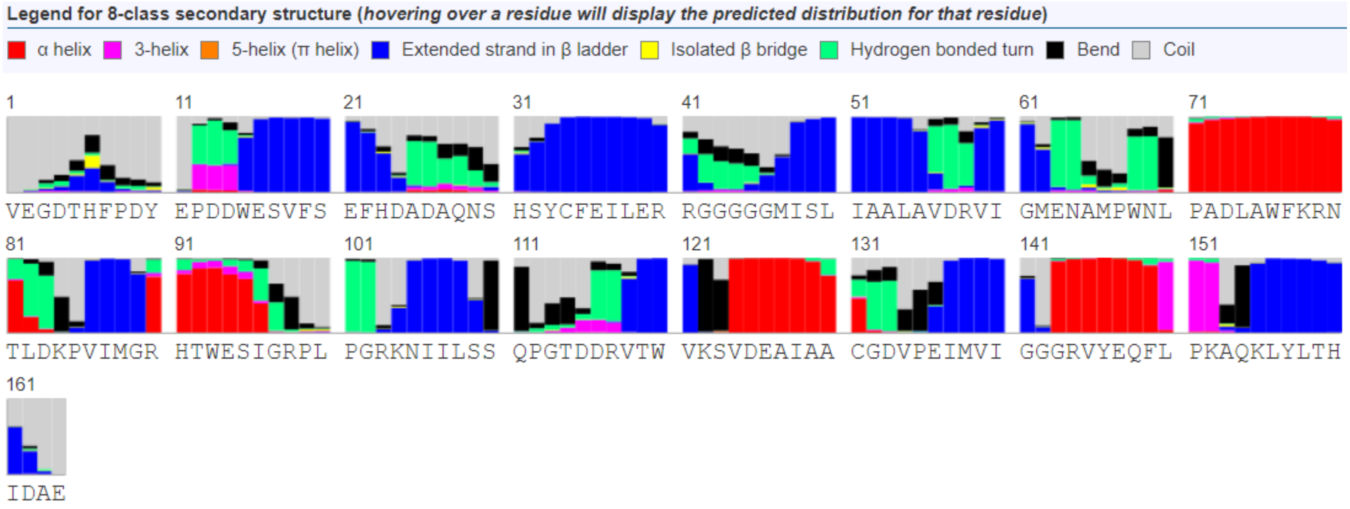

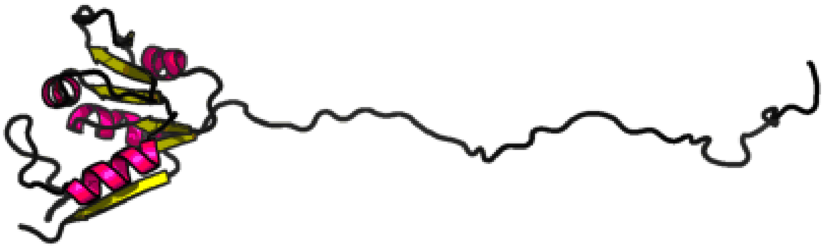

Template: PDB 1rx4A

CP site: Glu120

Target sequence:

```
EGDTHFPDYEPDDWESVFSEFHDADAQNSHSYCFEILERRGGGGGMISLIAALAVDRVIG
MENAMPWNLPADLAWFKRNTLDKPVIMGRHTWESIGRPLPGRKNIILSSQPGTDDRVTWV
KSVDEAIAACGDVPEIMVIGGGRVYEQFLPKAQKLYLTHIDAEV
```

Summary

- The input predicted as **1** domain(s)
- Best template: **1dreA**, p-value **1.49e-07**
- Overall uGDT (GDT): **122** (**74**)
- **164**(**100%**) residues are modeled
- **6**(**3%**) positions predicted as disordered
- Secondary struct: **18%**H, **31%**E, **49%**C
- Solvent access: **40%**E, **28%**M, **31%**B

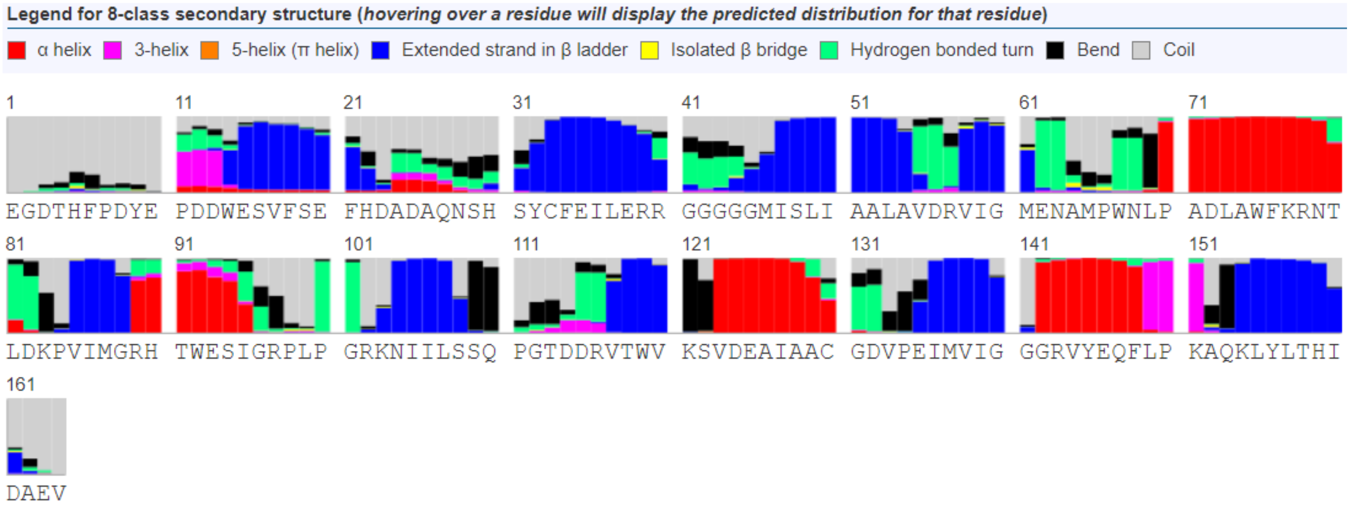

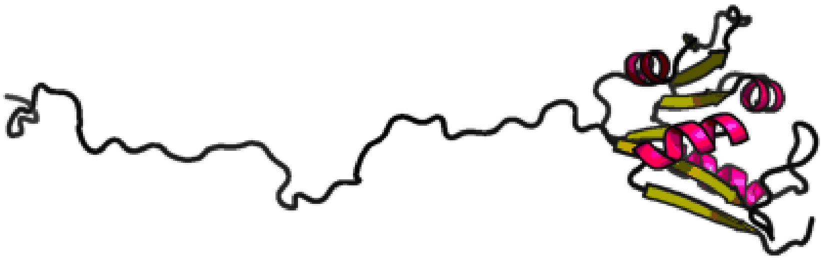

Template: PDB 1rx4A

CP site: Gly121

Target sequence:

GDTHFPDYEPDDWESVFSEFHDADAQNSHSYCFEILERRGGGGGMISLIAALAVDRVIGM
ENAMPWNLPADLAWFKRNTLDKPVIMGRHTWESIGRPLPGRKNIILSSQPGTDDRVTWVK
SVDEAIAACGDVPEIMVIGGGRVYEQFLPKAQKLYLTHIDAEVE

Summary

- The input predicted as **1** domain(s)
- Best template: **1dreA**, p-value **1.72e-07**
- Overall uGDT (GDT): **121** (**74**)
- **164**(**100%**) residues are modeled
- **6**(**3%**) positions predicted as disordered
- Secondary struct: **18%**H, **31%**E, **49%**C
- Solvent access: **39%**E, **29%**M, **30%**B

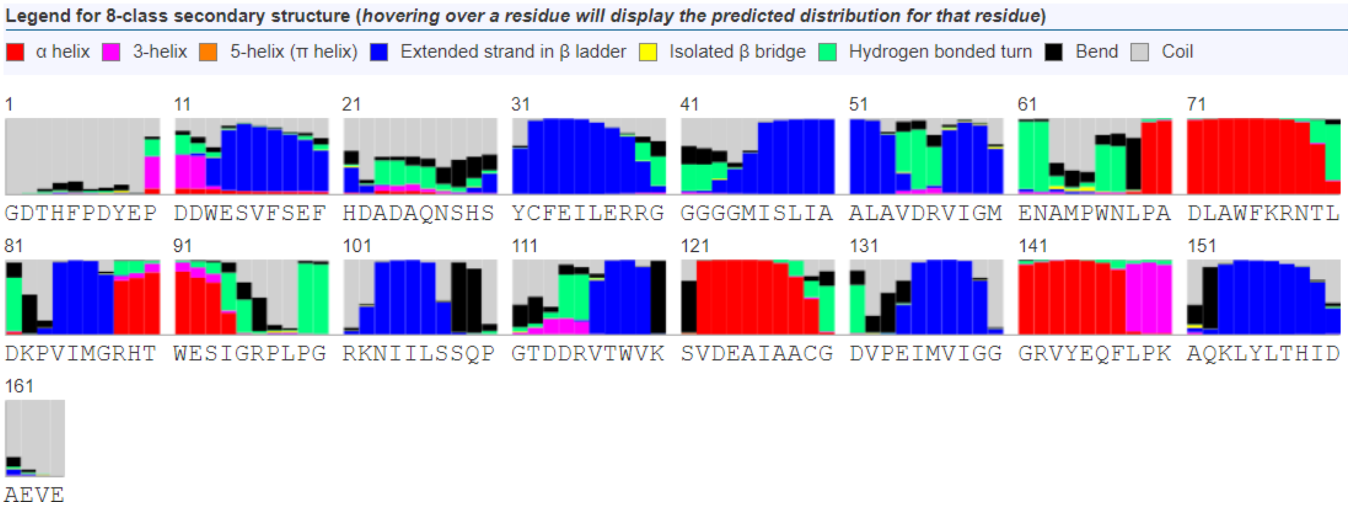

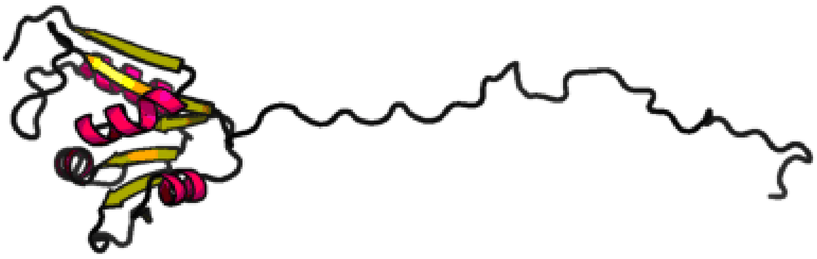

Template: PDB 1rx4A

CP site: Asp122

Target sequence:

DTHFPDYEPDDWESVFSEFHDADAQNSHSYCFEILERRGGGGGMISLIAALAVDRVIGME
NAMPWNLPADLAWFKRNTLDKPVIMGRHTWESIGRPLPGRKNIILSSQPGTDDRVTWVKS
VDEAIAACGDVPEIMVIGGGRVYEQFLPKAQKLYLTHIDAEVEG

Summary

- The input predicted as **1** domain(s)
- Best template: **1dreA**, p-value **1.31e-07**
- Overall uGDT (GDT): **123** (**75**)
- **164**(**100%**) residues are modeled
- **19**(**11%**) positions predicted as disordered
- Secondary struct: **18%**H, **29%**E, **51%**C
- Solvent access: **39%**E, **29%**M, **31%**B

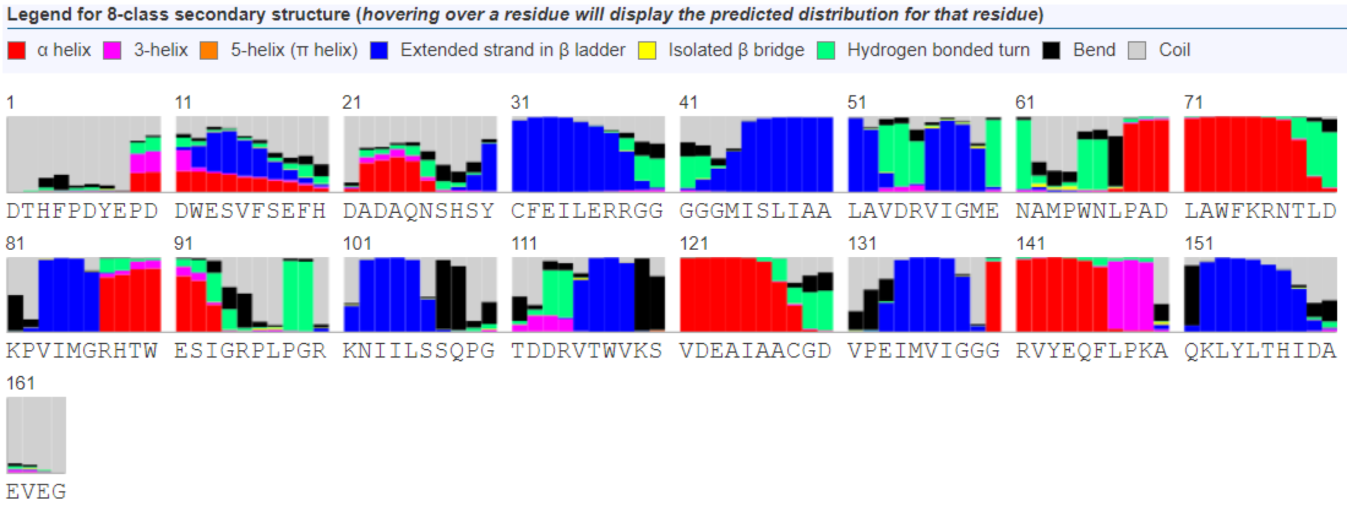

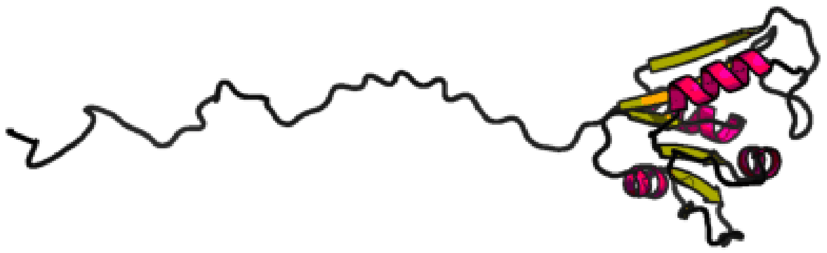

Template: PDB 1rx4A

CP site: Thr123

Target sequence:

THFPDYEPDDWESVFSEFHDADAQNSHSYCFEILERRGGGGGMISLIAALAVDRVIGMEN
AMPWNLPADLAWFKRNTLDKPVIMGRHTWESIGRPLPGRKNIILSSQPGTDDRVTWVKSV
DEAIAACGDVPEIMVIGGGRVYEQFLPKAQKLYLTHIDAEVEGD

Summary

- The input predicted as **1** domain(s)
- Best template: **1dreA**, p-value **1.19e-07**
- Overall uGDT (GDT): **123** (**75**)
- **164**(**100%**) residues are modeled
- **12**(**7%**) positions predicted as disordered
- Secondary struct: **23%**H, **25%**E, **51%**C
- Solvent access: **39%**E, **26%**M, **34%**B

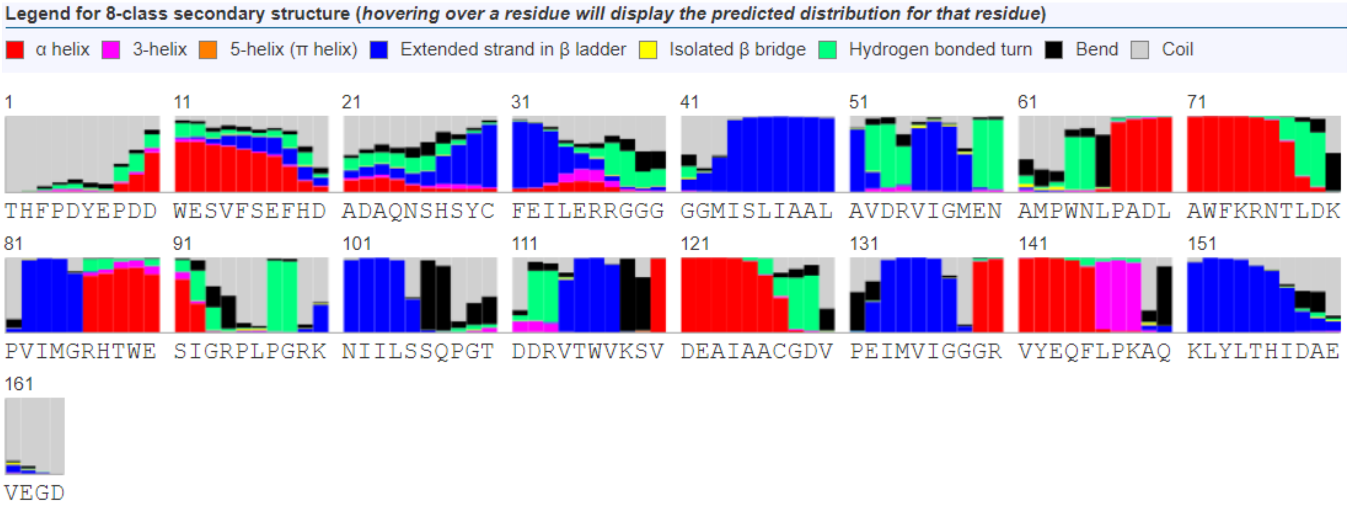

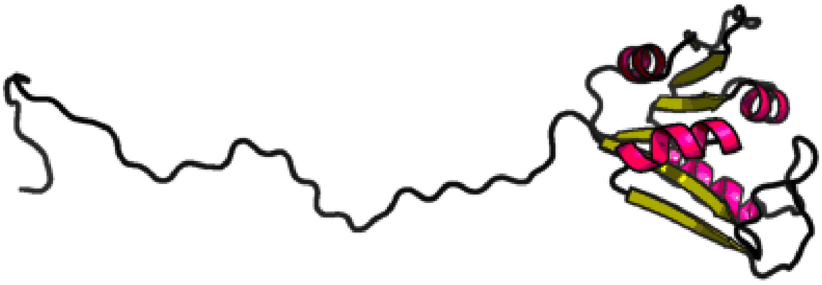

Template: PDB 1rx4A

CP site: Pro126

Target sequence:

PDYEPDDWESVFSEFHDADAQNSHSYCFEILERRGGGGGMISLIAALAVDRVIGMENAMP
WNLPADLAWFKRNTLDKPVIMGRHTWESIGRPLPGRKNIILSSQPGTDDRVTWVKSVDEA
IAACGDVPEIMVIGGGRVYEQFLPKAQKLYLTHIDAEVEGDTHF

Summary

- The input predicted as **1** domain(s)
- Best template: **1dreA**, p-value **5.29e-08**
- Overall uGDT (GDT): **127** (**77**)
- **164**(**100%**) residues are modeled
- **33**(**20%**) positions predicted as disordered
- Secondary struct: **23%**H, **25%**E, **50%**C
- Solvent access: **42%**E, **26%**M, **31%**B

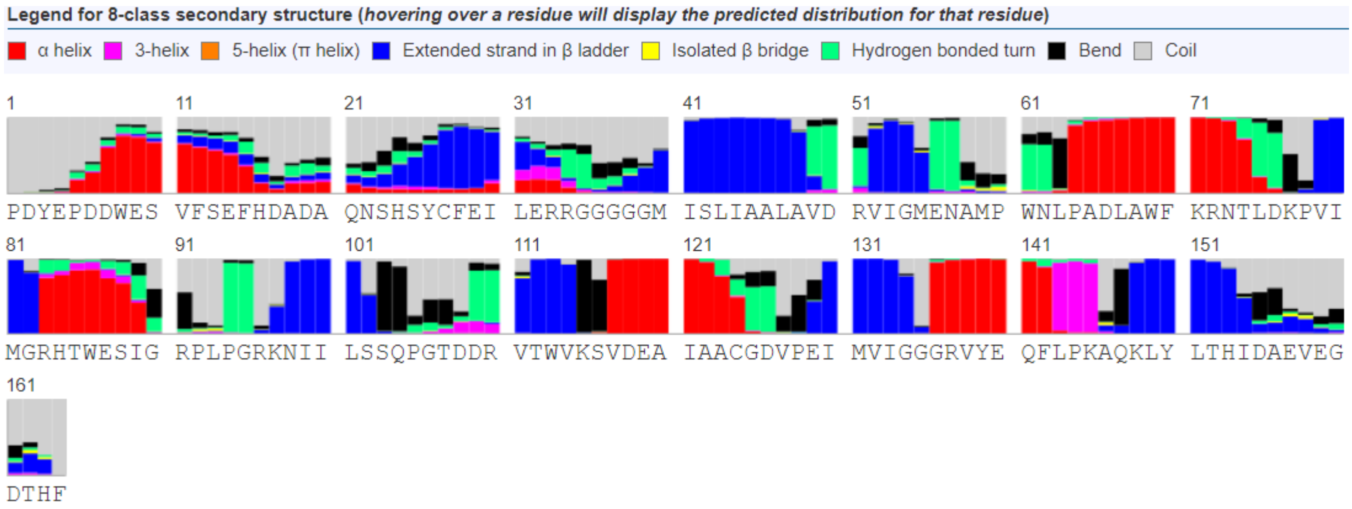

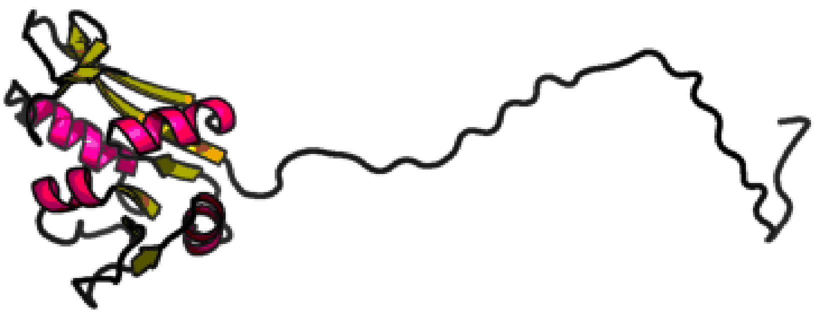

Template: PDB 1rx4A

CP site: Asp127

Target sequence:

DYEPDDWESVFSEFHDADAQNSHSYCFEILERRGGGGGMISLIAALAVDRVIGMENAMPW
NLPADLAWFKRNTLDKPVIMGRHTWESIGRPLPGRKNIILSSQPGTDDRVTWVKSVDEAI
AACGDVPEIMVIGGGRVYEQFLPKAQKLYLTHIDAEVEGDTHFP

Summary

- The input predicted as **1** domain(s)
- Best template: **1dreA**, p-value **8.02e-08**
- Overall uGDT (GDT): **129** (**79**)
- **164**(**100%**) residues are modeled
- **29**(**17%**) positions predicted as disordered
- Secondary struct: **23%**H, **25%**E, **51%**C
- Solvent access: **40%**E, **25%**M, **34%**B

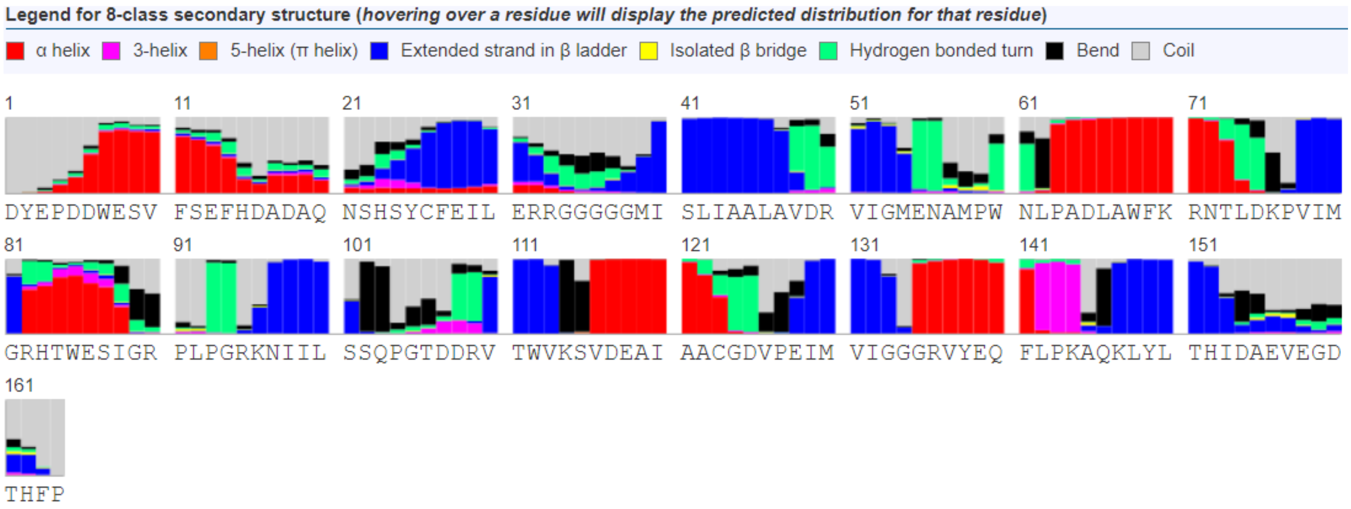

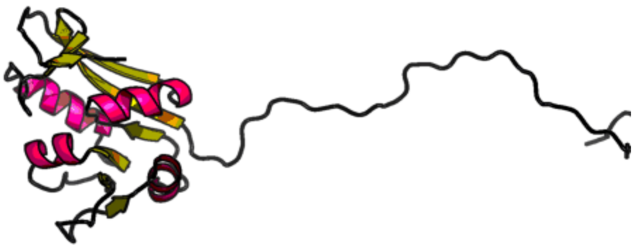

Template: PDB 1rx4A

CP site: Tyr128

Target sequence:

YEPDDWESVFSEFHDADAQNSHSYCFEILERRGGGGGMISLIAALAVDRVIGMENAMPWN
LPADLAWFKRNTLDKPVIMGRHTWESIGRPLPGRKNIILSSQPGTDDRVTWVKSVDEAIA
ACGDVPEIMVIGGGRVYEQFLPKAQKLYLTHIDAEVEGDTHFPD

Summary

- The input predicted as **1** domain(s)
- Best template: **1dreA**, p-value **1.12e-07**
- Overall uGDT (GDT): **129** (**78**)
- **164**(**100%**) residues are modeled
- **28**(**17%**) positions predicted as disordered
- Secondary struct: **23%**H, **26%**E, **50%**C
- Solvent access: **40%**E, **25%**M, **34%**B

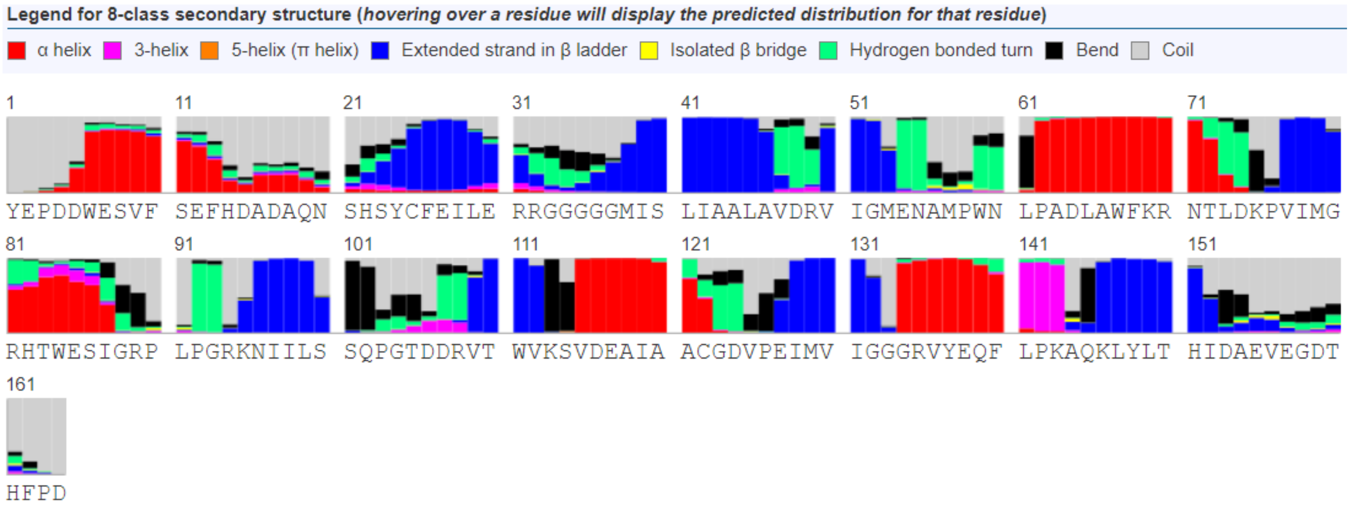

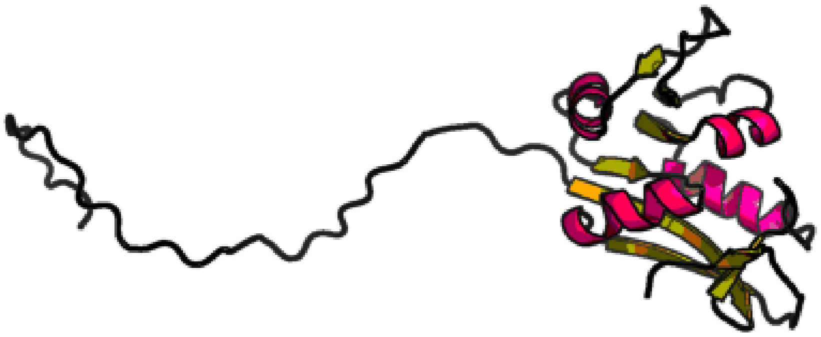

Template: PDB 1rx4A

CP site: Glu129

Target sequence:

EPDDWESVFSEFHDADAQNSHSYCFEILERRGGGGGMISLIAALAVDRVIGMENAMPWNL
PADLAWFKRNTLDKPVIMGRHTWESIGRPLPGRKNIILSSQPGTDDRVTWVKSVDEAIAA
CGDVPEIMVIGGGRVYEQFLPKAQKLYLTHIDAEVEGDTHFPDY

Summary

- The input predicted as **1** domain(s)
- Best template: **1dreA**, p-value **4.80e-08**
- Overall uGDT (GDT): **131** (**79**)
- **164**(**100%**) residues are modeled
- **28**(**17%**) positions predicted as disordered
- Secondary struct: **23%**H, **26%**E, **50%**C
- Solvent access: **40%**E, **26%**M, **32%**B

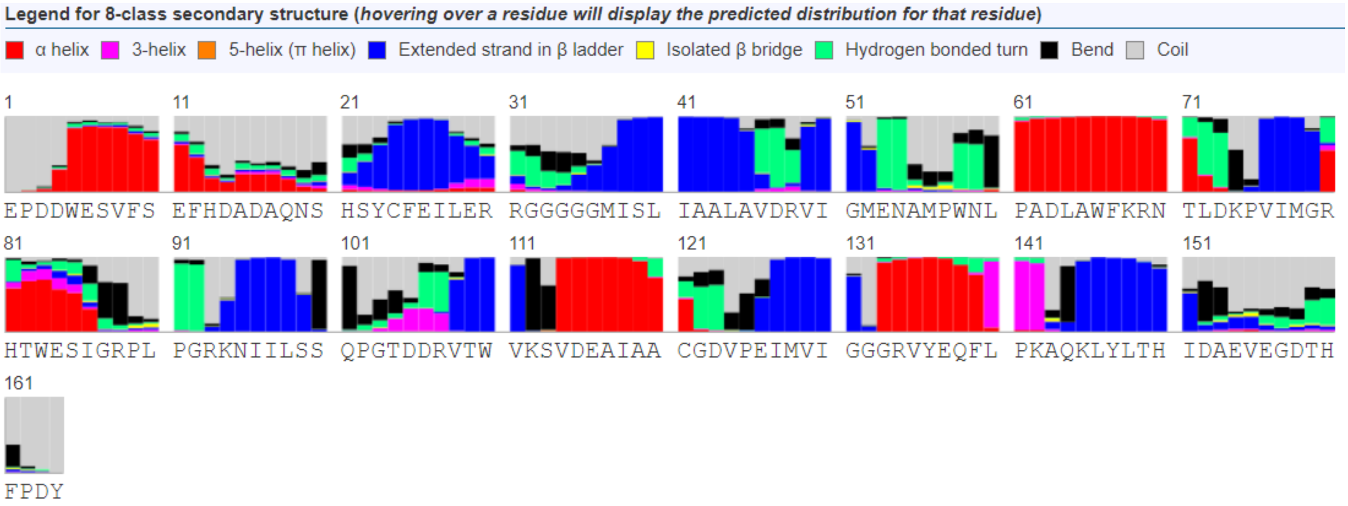

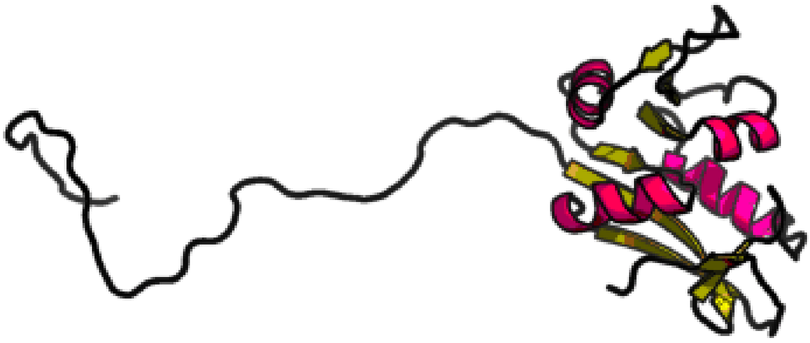

Template: PDB 1rx4A

CP site: Pro130

Target sequence:

PDDWESVFSEFHDADAQNSHSYCFEILERRGGGGGMISLIAALAVDRVIGMENAMPWNLP
ADLAWFKRNTLDKPVIMGRHTWESIGRPLPGRKNIILSSQPGTDDRVTWVKSVDEAIAAC
GDVPEIMVIGGGRVYEQFLPKAQKLYLTHIDAEVEGDTHFPDYE

Summary

- The input predicted as **1** domain(s)
- Best template: **1dreA**, p-value **6.72e-08**
- Overall uGDT (GDT): **132** (**80**)
- **164**(**100%**) residues are modeled
- **27**(**16%**) positions predicted as disordered
- Secondary struct: **23%**H, **26%**E, **50%**C
- Solvent access: **40%**E, **28%**M, **31%**B

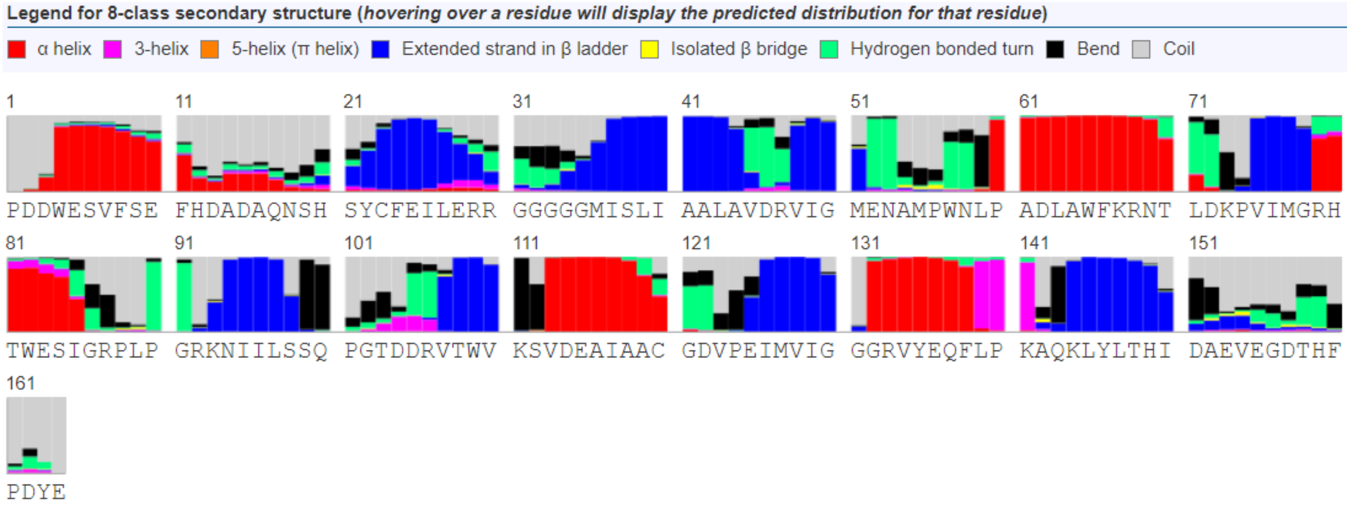

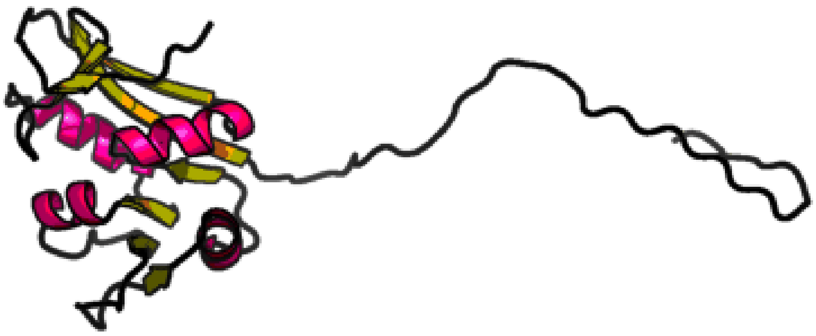

Template: PDB 1rx4A

CP site: Asp131

Target sequence:

DDWESVFSEFHDADAQNSHSYCFEILERRGGGGGMISLIAALAVDRVIGMENAMPWNLPA
DLAWFKRNTLDKPVIMGRHTWESIGRPLPGRKNIILSSQPGTDDRVTWVKSVDEAIAACG
DVPEIMVIGGGRVYEQFLPKAQKLYLTHIDAEVEGDTHFPDYEP

Summary

- The input predicted as **1** domain(s)
- Best template: **1dreA**, p-value **6.32e-08**
- Overall uGDT (GDT): **131** (**80**)
- **164**(**100%**) residues are modeled
- **26**(**15%**) positions predicted as disordered
- Secondary struct: **23%**H, **26%**E, **49%**C
- Solvent access: **40%**E, **27%**M, **31%**B

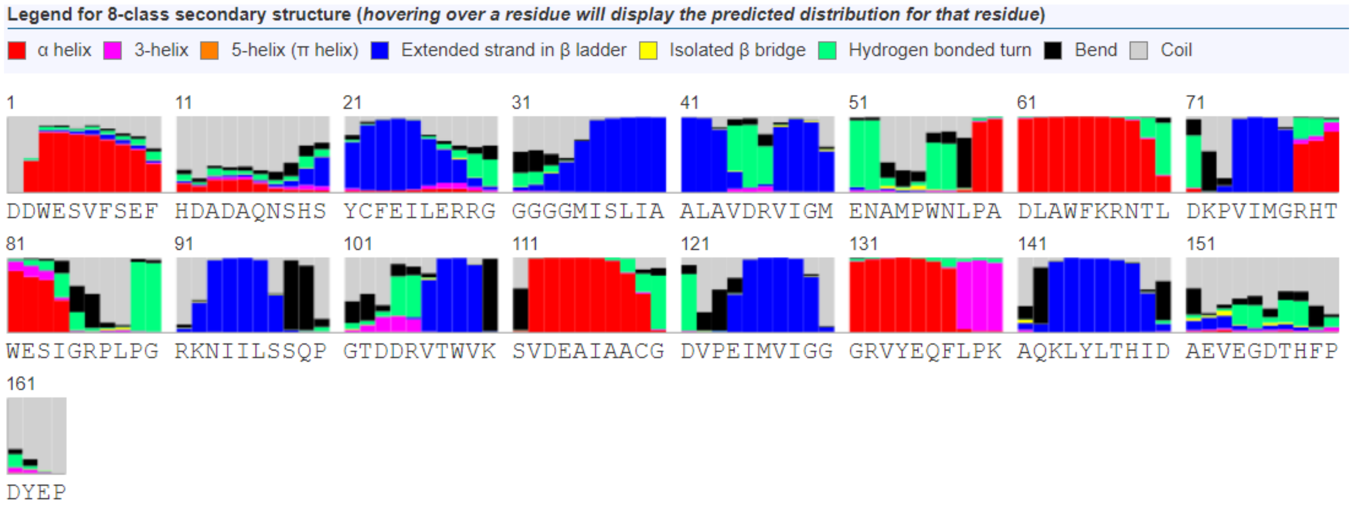

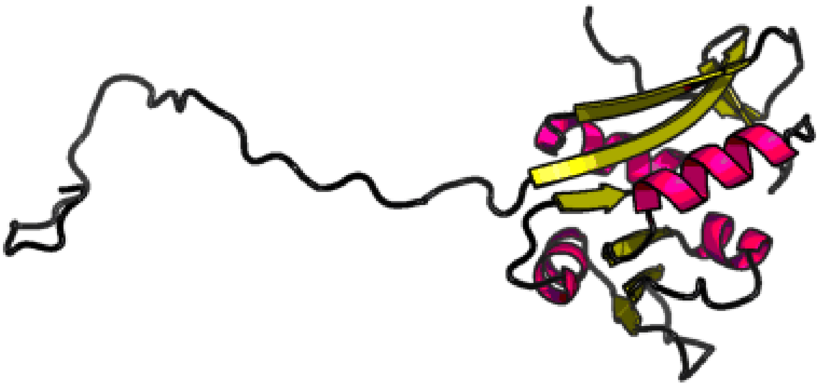

Template: PDB 1rx4A

CP site: Asp132

Target sequence:

DWESVFSEFHDADAQNSHSYCFEILERRGGGGGMISLIAALAVDRVIGMENAMPWNLPAD
LAWFKRNTLDKPVIMGRHTWESIGRPLPGRKNIILSSQPGTDDRVTWVKSVDEAIAACGD
VPEIMVIGGGRVYEQFLPKAQKLYLTHIDAEVEGDTHFPDYEPD

Summary

- The input predicted as **1** domain(s)
- Best template: **1dreA**, p-value **3.12e-08**
- Overall uGDT (GDT): **133** (**81**)
- **164**(**100%**) residues are modeled
- **26**(**15%**) positions predicted as disordered
- Secondary struct: **23%**H, **26%**E, **50%**C
- Solvent access: **40%**E, **26%**M, **32%**B

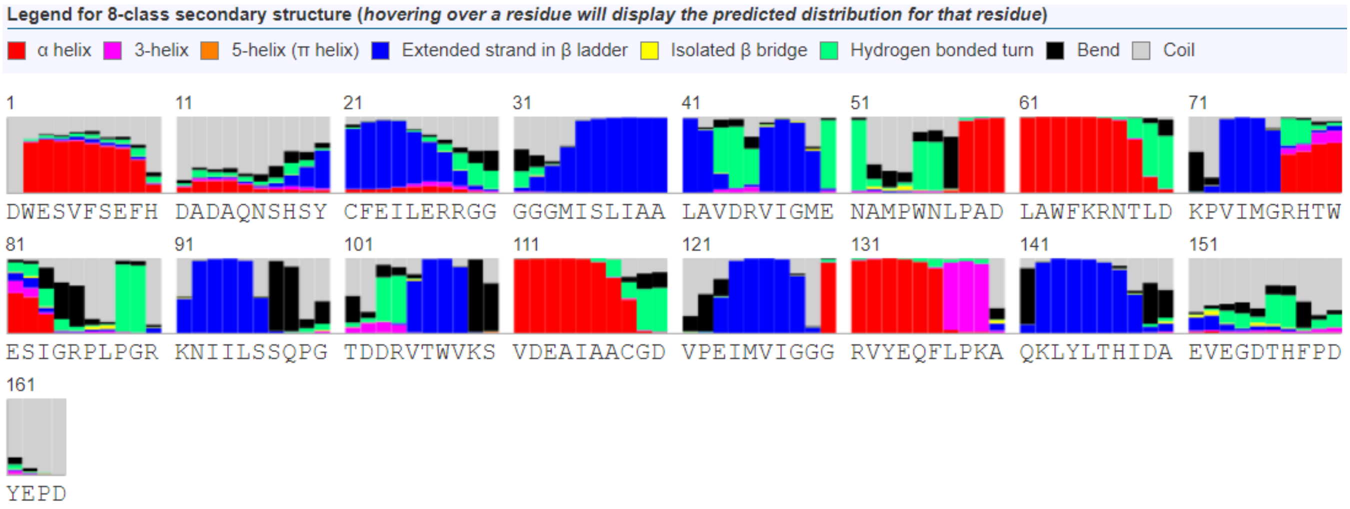

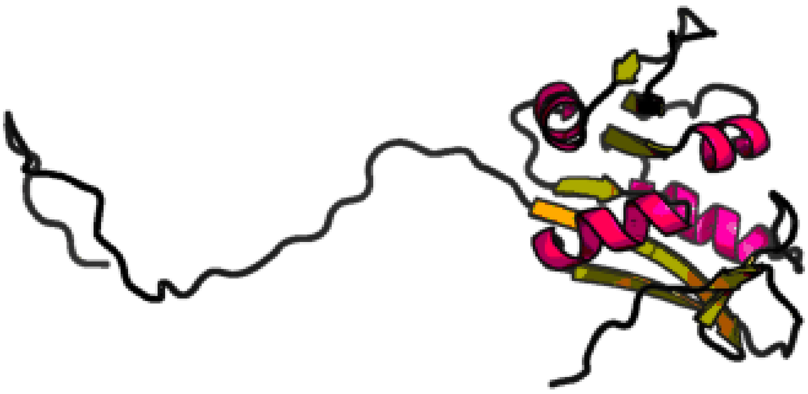

Template: PDB 1rx4A

CP site: Trp133

Target sequence:

WESVFSEFHDADAQNSHSYCFEILERRGGGGGMISLIAALAVDRVIGMENAMPWNLPADL
AWFKRNTLDKPVIMGRHTWESIGRPLPGRKNIILSSQPGTDDRVTWVKSVDEAIAACGDV
PEIMVIGGGRVYEQFLPKAQKLYLTHIDAEVEGDTHFPDYEPDD

Summary

- The input predicted as **1** domain(s)
- Best template: **1dreA**, p-value **4.26e-08**
- Overall uGDT (GDT): **132** (**81**)
- **164**(**100%**) residues are modeled
- **28**(**17%**) positions predicted as disordered
- Secondary struct: **18%**H, **26%**E, **54%**C
- Solvent access: **37%**E, **29%**M, **32%**B

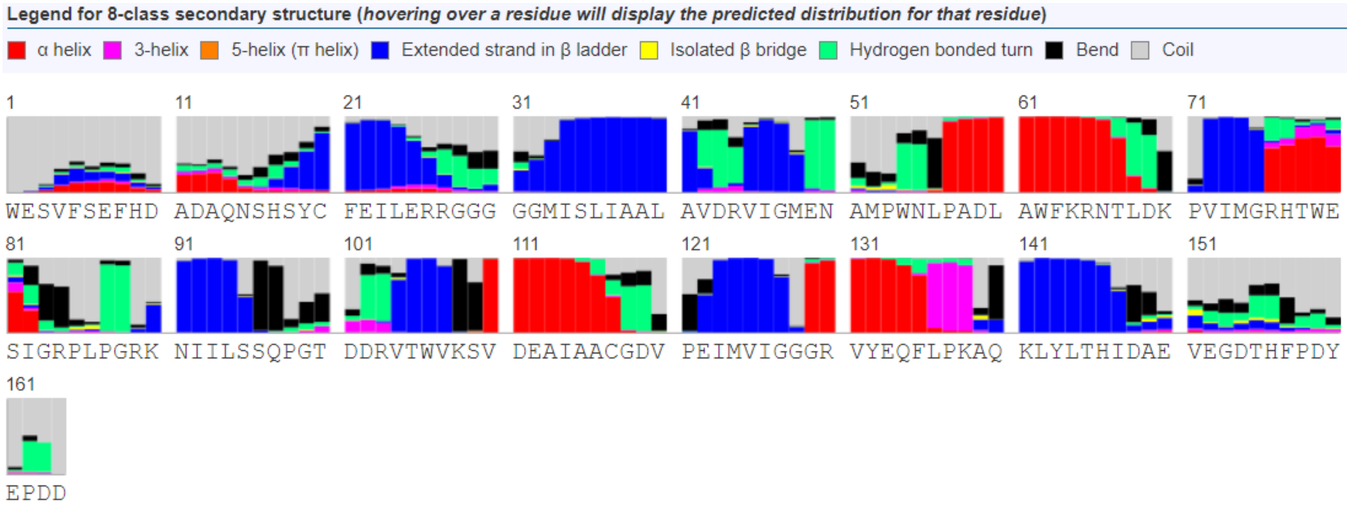

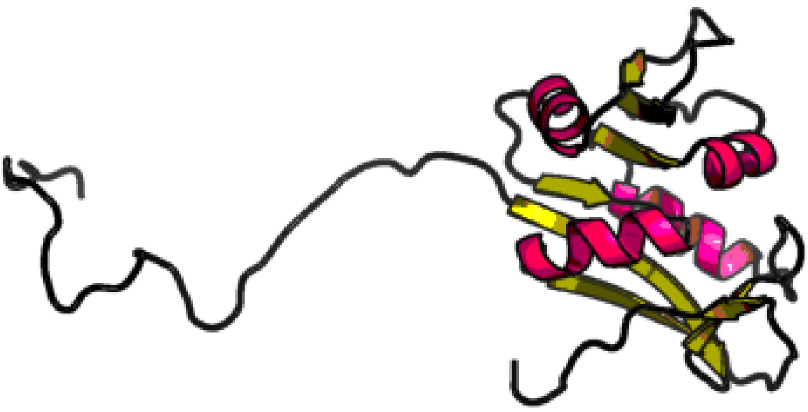

Template: PDB 1rx4A

CP site: Glu134

Target sequence:

ESVFSEFHDADAQNSHSYCFEILERRGGGGGMISLIAALAVDRVIGMENAMPWNLPADLA
WFKRNTLDKPVIMGRHTWESIGRPLPGRKNIILSSQPGTDDRVTWVKSVDEAIAACGDVP
EIMVIGGGRVYEQFLPKAQKLYLTHIDAEVEGDTHFPDYEPDDW

Summary

- The input predicted as **1** domain(s)
- Best template: **1dreA**, p-value **3.49e-08**
- Overall uGDT (GDT): **132** (**80**)
- **164**(**100%**) residues are modeled
- **33**(**20%**) positions predicted as disordered
- Secondary struct: **21%**H, **26%**E, **51%**C
- Solvent access: **39%**E, **28%**M, **32%**B

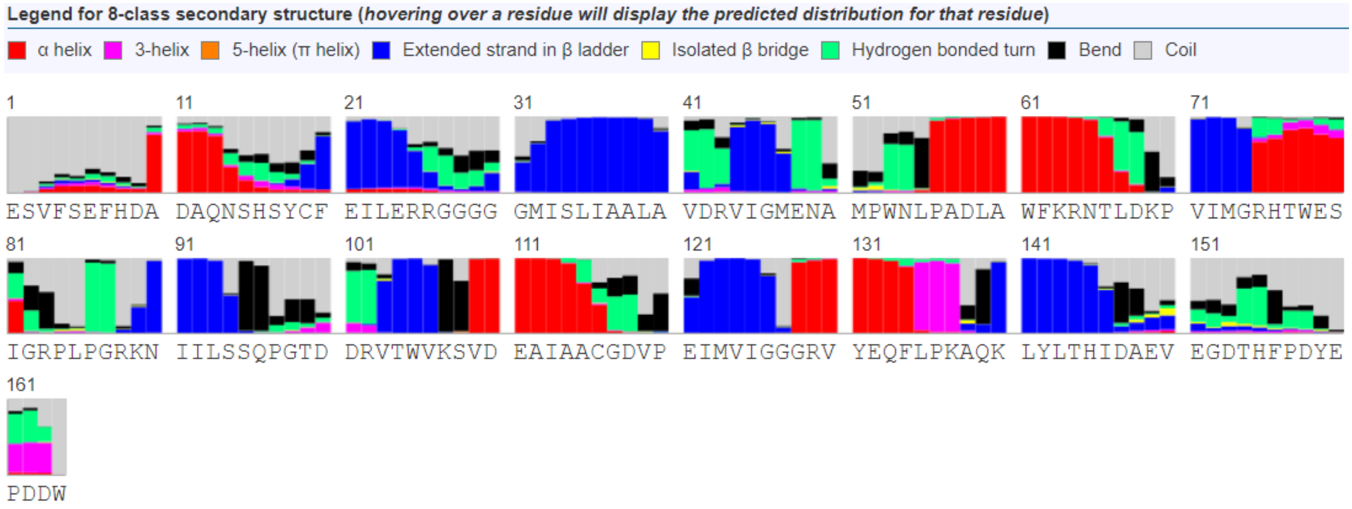

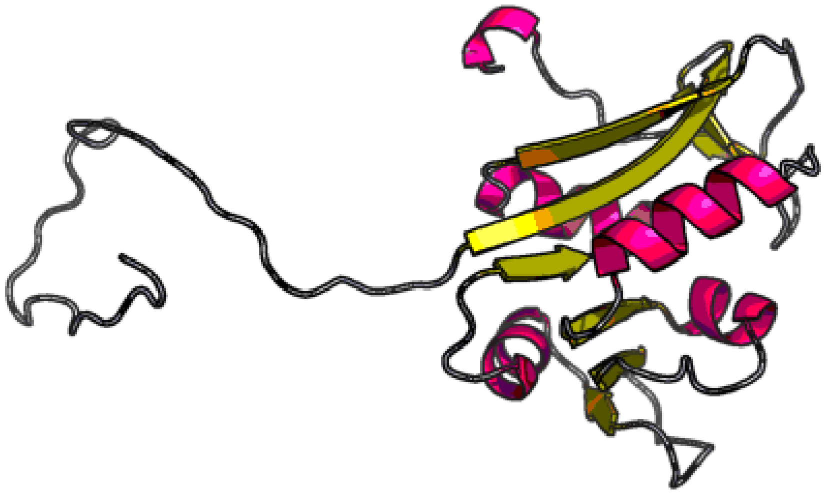

Template: PDB 1rx4A

CP site: Ser135

Target sequence:

SVFSEFHDADAQNSHSYCFEILERRGGGGGMISLIAALAVDRVIGMENAMPWNLPADLA
WFKRNTLDKPVIMGRHTWESIGRPLPGRKNIILSSQPGTDDRVTWVKSVDEAIAACGDV
PEIMVIGGGRVYEQFLPKAQKLYLTHIDAEVEGDTHFPDYEPDDWE

Summary

- The input predicted as **1** domain(s)
- Best template: **1tdrA**, p-value **4.94e-08**
- Overall uGDT (GDT): **135** (**82**)
- **164**(**100%**) residues are modeled
- **24**(**14%**) positions predicted as disordered
- Secondary struct: **18%**H, **25%**E, **55%**C
- Solvent access: **39%**E, **28%**M, **32%**B

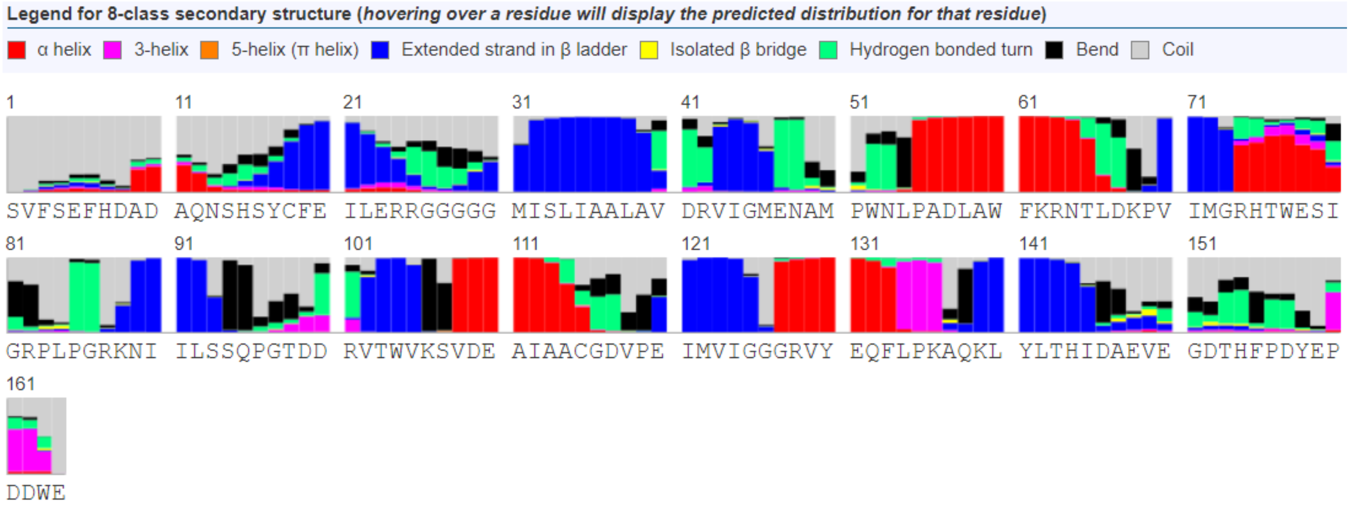

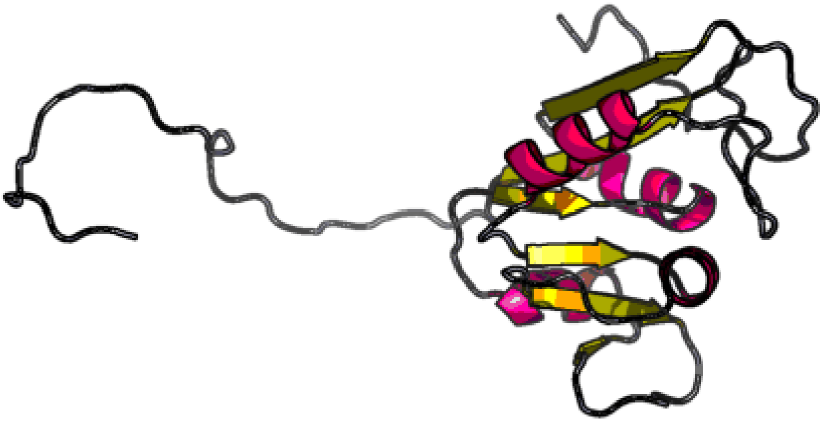

Template: PDB 1rx4A

CP site: Glu139

Target sequence:

EFHDADAQNSHSYCFEILERRGGGGGMISLIAALAVDRVIGMENAMPWNLPADLAWFK
RNTLDKPVIMGRHTWESIGRPLPGRKNIILSSQPGTDDRVTWVKSVDEAIAACGDVPEIM
VIGGGRVYEQFLPKAQKLYLTHIDAEVEGDTHFPDYEPDDWESVFS

Summary

- The input predicted as **1** domain(s)
- Best template: **1tdrA**, p-value **2.23e-08**
- Overall uGDT (GDT): **138** (**84**)
- **164**(**100%**) residues are modeled
- **19**(**11%**) positions predicted as disordered
- Secondary struct: **20%**H, **27%**E, **51%**C
- Solvent access: **37%**E, **28%**M, **34%**B

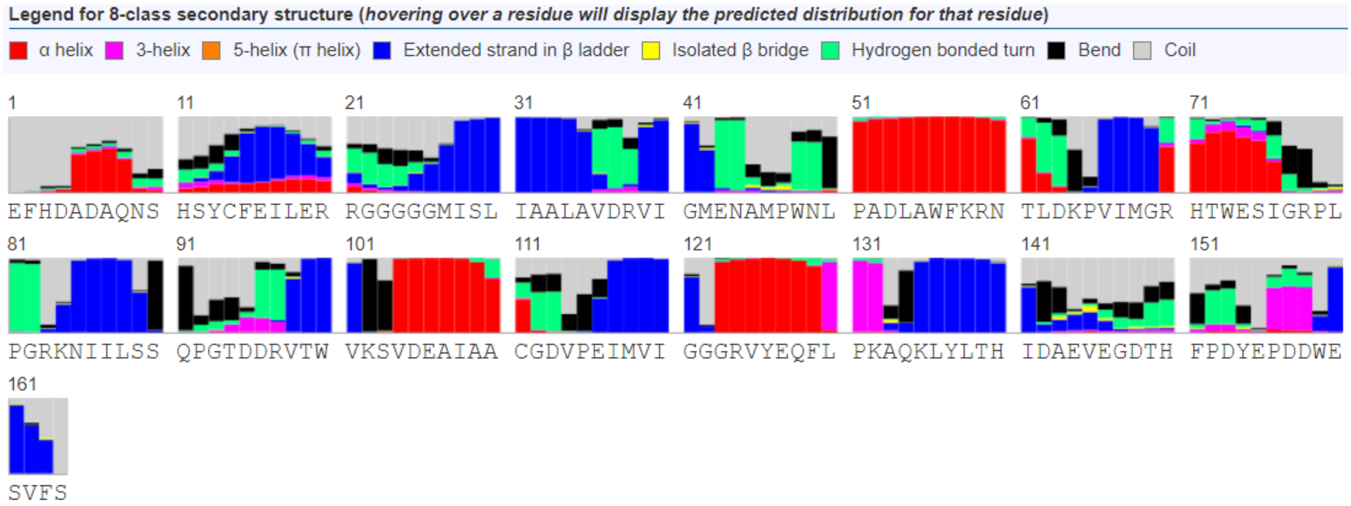

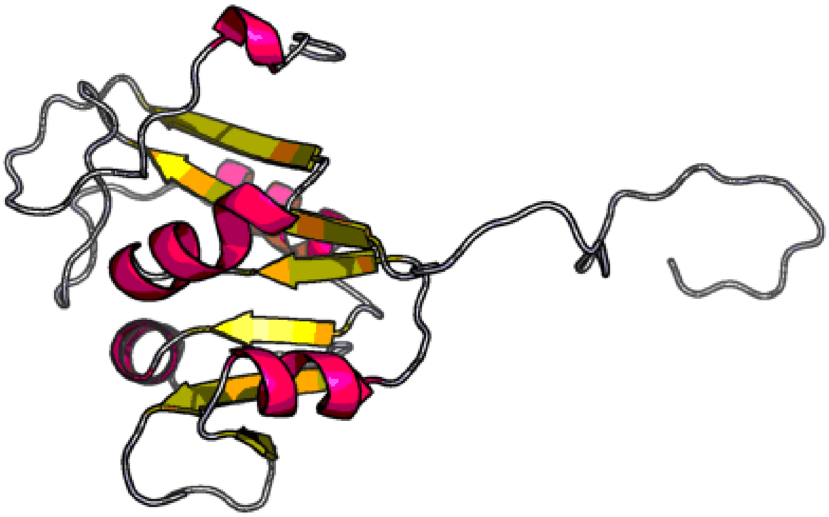

Template: PDB 1rx4A

CP site: Phe140

Target sequence:

FHDADAQNSHSYCFEILERRGGGGGMISLIAALAVDRVIGMENAMPWNLPADLAWFKR
NTLDKPVIMGRHTWESIGRPLPGRKNIILSSQPGTDDRVTWVKSVDEAIAACGDVPEIMV
IGGGRVYEQFLPKAQKLYLTHIDAEVEGDTHFPDYEPDDWESVFSE

Summary

- The input predicted as **1** domain(s)
- Best template: **1tdrA**, p-value **2.42e-08**
- Overall uGDT (GDT): **141** (**86**)
- **164**(**100%**) residues are modeled
- **22**(**13%**) positions predicted as disordered
- Secondary struct: **18%**H, **28%**E, **53%**C
- Solvent access: **37%**E, **31%**M, **31%**B

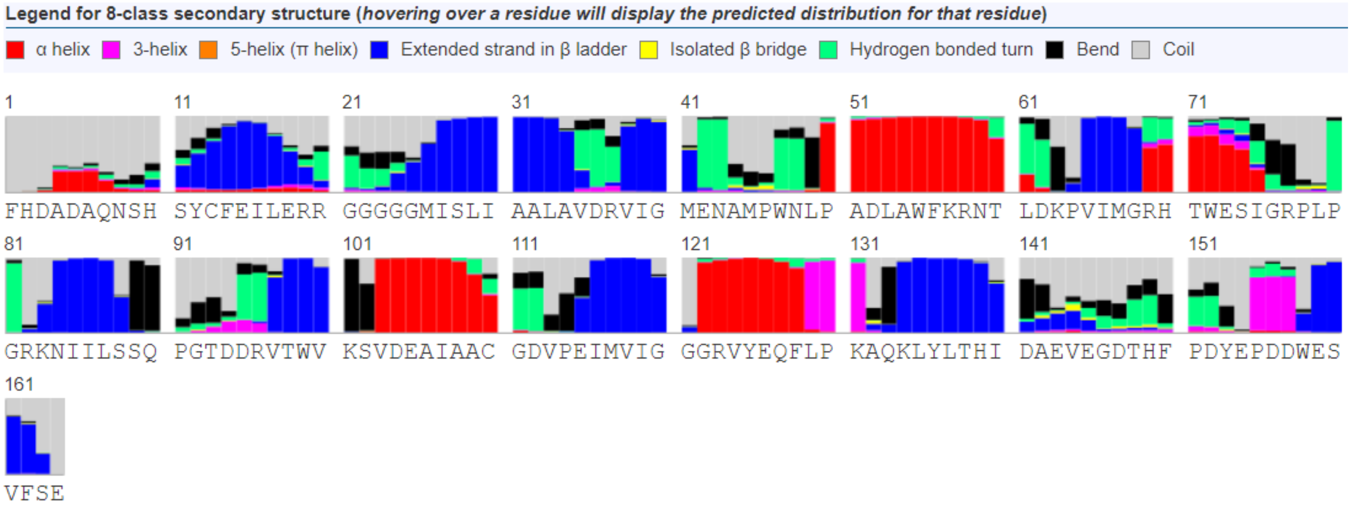

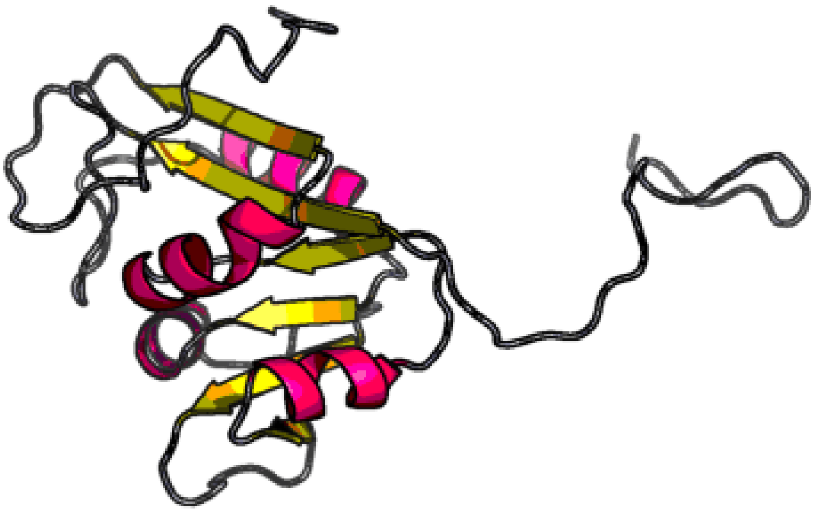

Template: PDB 1rx4A

CP site: His141

Target sequence:

HDADAQNSHSYCFEILERRGGGGGMISLIAALAVDRVIGMENAMPWNLPADLAWFKRN
TLDKPVIMGRHTWESIGRPLPGRKNIILSSQPGTDDRVTWVKSVDEAIAACGDVPEIMVI
GGGRVYEQFLPKAQKLYLTHIDAEVEGDTHFPDYEPDDWESVFSEF

Summary

- The input predicted as **1** domain(s)
- Best template: **1tdrA**, p-value **2.69e-08**
- Overall uGDT (GDT): **142** (**87**)
- **164**(**100%**) residues are modeled
- **25**(**15%**) positions predicted as disordered
- Secondary struct: **18%**H, **29%**E, **51%**C
- Solvent access: **38%**E, **29%**M, **32%**B

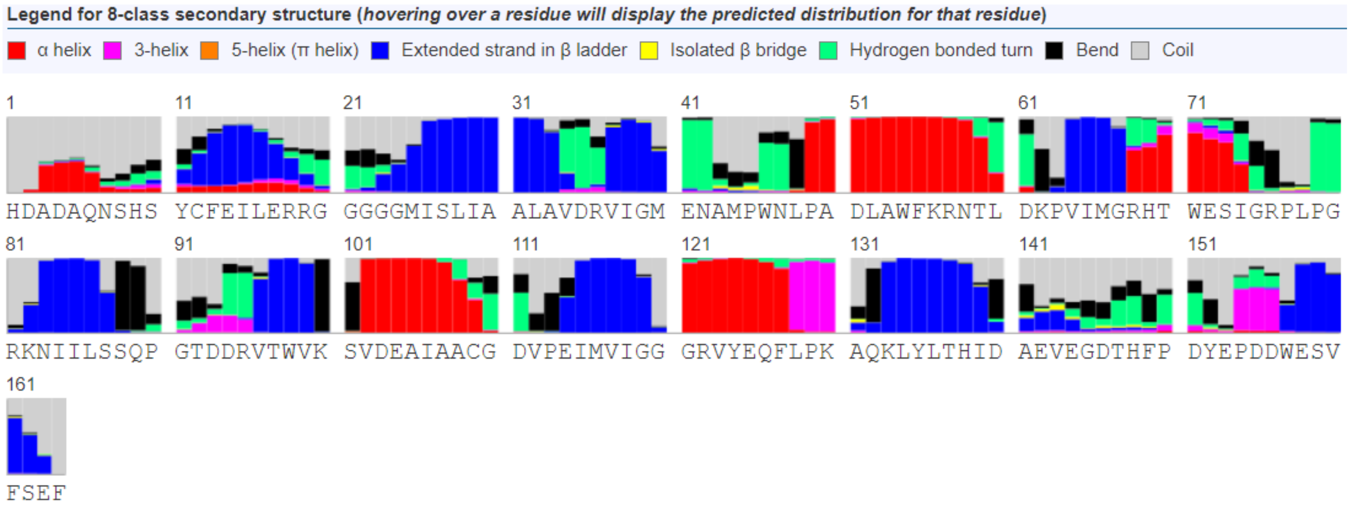

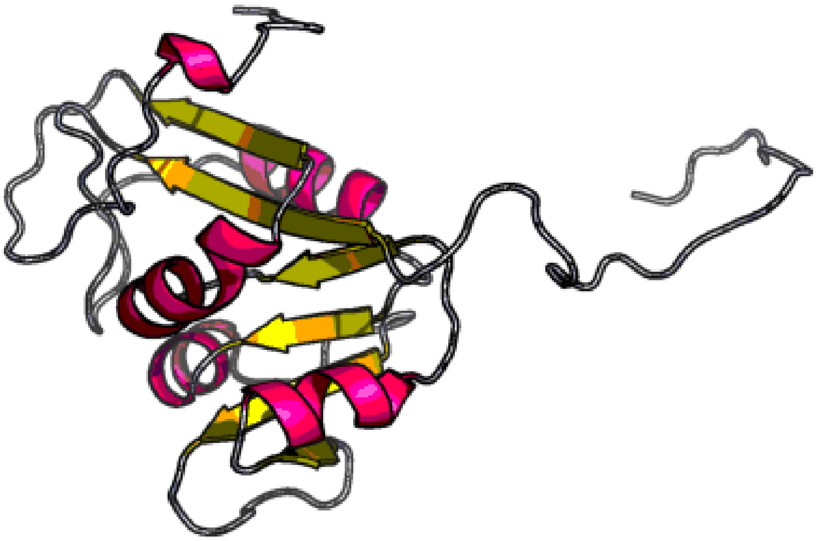

Template: PDB 1rx4A

CP site: Asp142

Target sequence:

DADAQNSHSYCFEILERRGGGGGMISLIAALAVDRVIGMENAMPWNLPADLAWFKRNT
LDKPVIMGRHTWESIGRPLPGRKNIILSSQPGTDDRVTWVKSVDEAIAACGDVPEIMVIG
GGRVYEQFLPKAQKLYLTHIDAEVEGDTHFPDYEPDDWESVFSEFH

Summary

- The input predicted as **1** domain(s)
- Best template: **1tdrA**, p-value **2.28e-08**
- Overall uGDT (GDT): **143** (**87**)
- **164**(**100%**) residues are modeled
- **19**(**11%**) positions predicted as disordered
- Secondary struct: **18%**H, **29%**E, **51%**C
- Solvent access: **37%**E, **31%**M, **31%**B

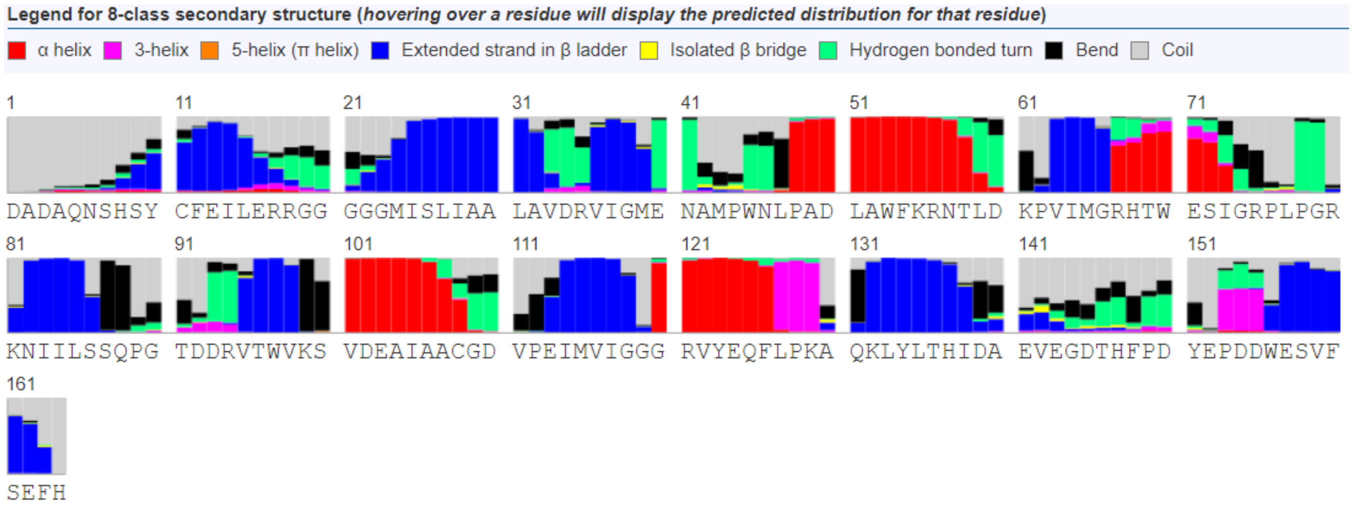

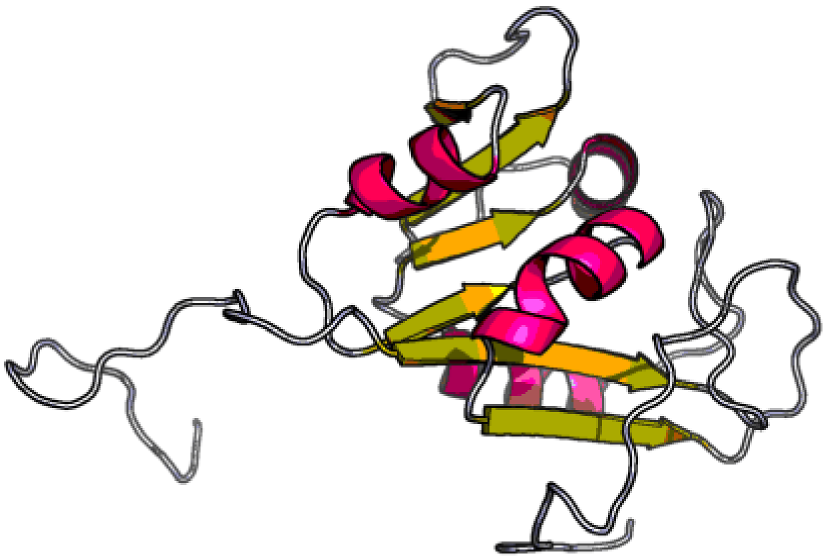

Template: PDB 1rx4A

CP site: Ala143

Target sequence:

ADAQNSHSYCFEILERRGGGGGMISLIAALAVDRVIGMENAMPWNLPADLAWFKRNTL
DKPVIMGRHTWESIGRPLPGRKNIILSSQPGTDDRVTWVKSVDEAIAACGDVPEIMVIGG
GRVYEQFLPKAQKLYLTHIDAEVEGDTHFPDYEPDDWESVFSEFHD

Summary

•The input predicted as **1** domain(s)
•Best template: **1tdrA**, p-value **1.28e-08**
•Overall uGDT (GDT): **144** (**88**)
•**164**(**100%**) residues are modeled
•**16**(**9%**) positions predicted as disordered
•Secondary struct: **18%**H, **30%**E, **50%**C
•Solvent access: **37%**E, **31%**M, **31%**B

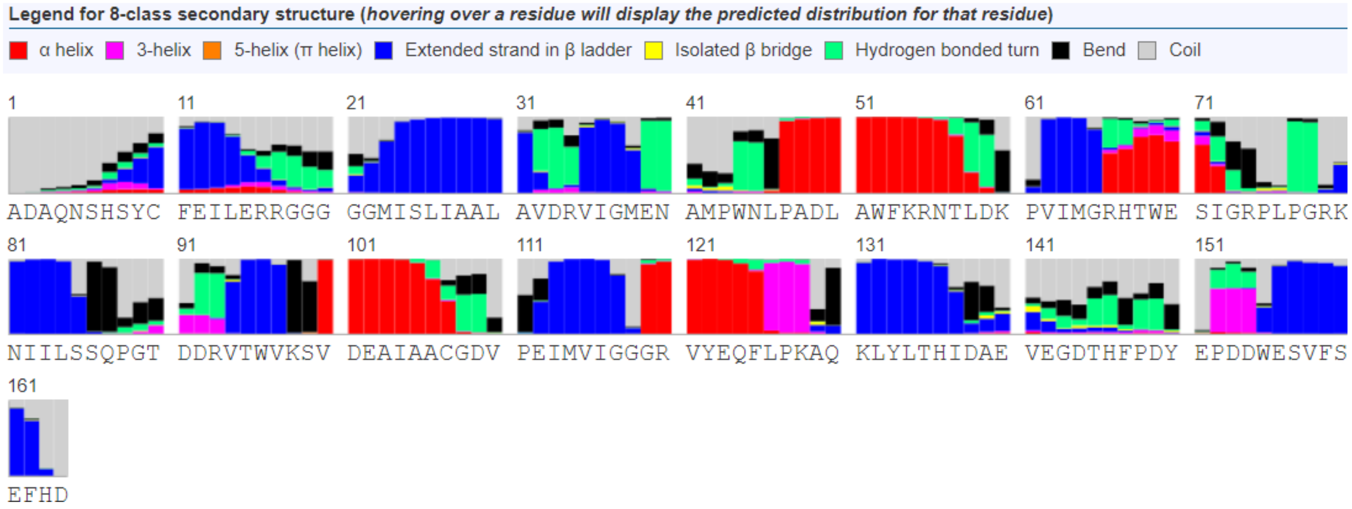

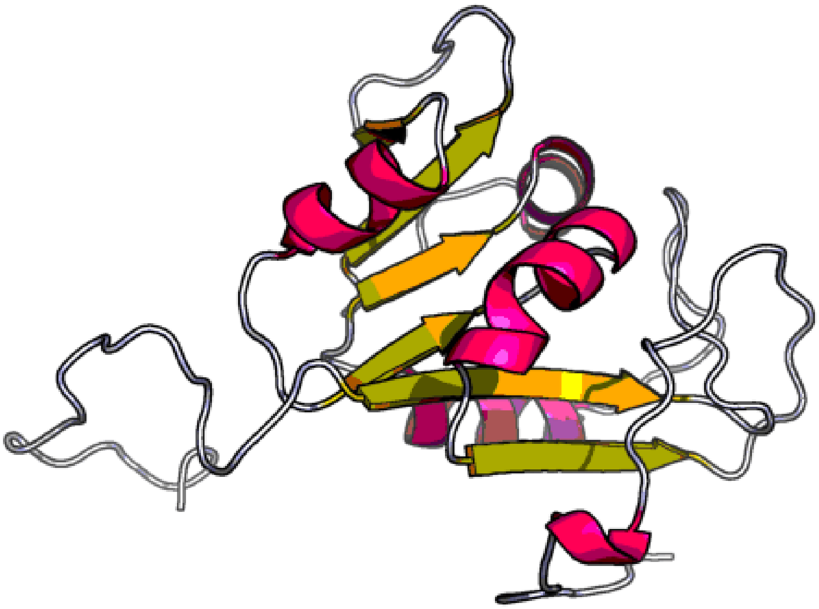

Template: PDB 1rx4A

CP site: Asp144

Target sequence:

DAQNSHSYCFEILERRGGGGGMISLIAALAVDRVIGMENAMPWNLPADLAWFKRNTLD
KPVIMGRHTWESIGRPLPGRKNIILSSQPGTDDRVTWVKSVDEAIAACGDVPEIMVIGGG
RVYEQFLPKAQKLYLTHIDAEVEGDTHFPDYEPDDWESVFSEFHDA

Summary

- The input predicted as **1** domain(s)
- Best template: **1tdrA**, p-value **1.33e-08**
- Overall uGDT (GDT): **146** (**89**)
- **164**(**100%**) residues are modeled
- **14**(**8%**) positions predicted as disordered
- Secondary struct: **18%**H, **29%**E, **51%**C
- Solvent access: **36%**E, **31%**M, **31%**B

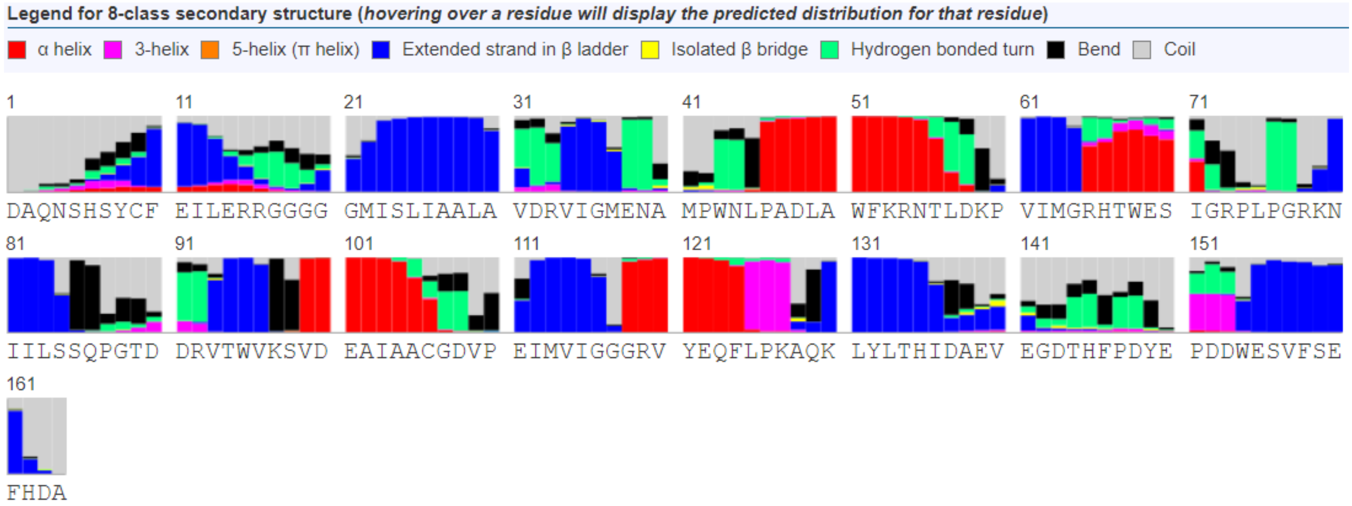

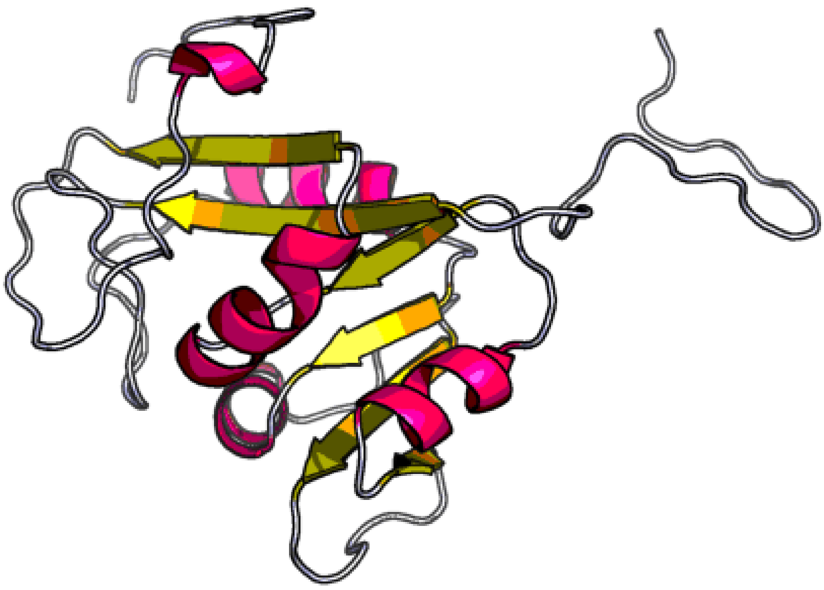

Template: PDB 1rx4A

CP site: Ala145

Target sequence:

AQNSHSYCFEILERRGGGGGMISLIAALAVDRVIGMENAMPWNLPADLAWFKRNTLDK
PVIMGRHTWESIGRPLPGRKNIILSSQPGTDDRVTWVKSVDEAIAACGDVPEIMVIGGGR
VYEQFLPKAQKLYLTHIDAEVEGDTHFPDYEPDDWESVFSEFHDAD

Summary

- The input predicted as **1** domain(s)
- Best template: **1tdrA**, p-value **7.12e-09**
- Overall uGDT (GDT): **146** (**89**)
- **164**(**100%**) residues are modeled
- **20**(**12%**) positions predicted as disordered
- Secondary struct: **17%**H, **29%**E, **53%**C
- Solvent access: **37%**E, **29%**M, **32%**B

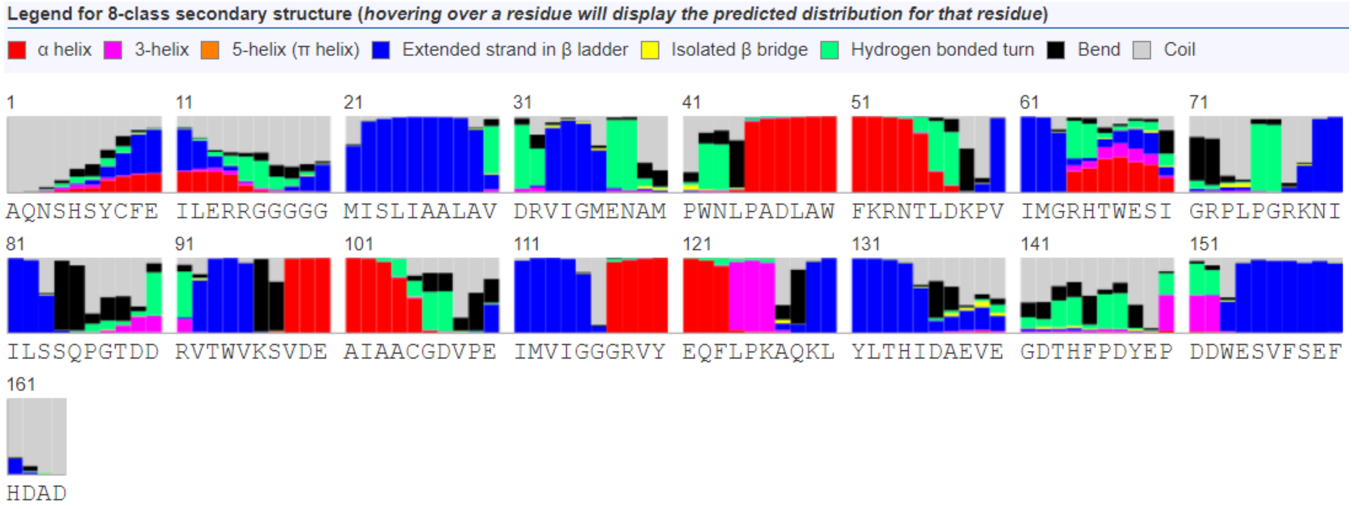

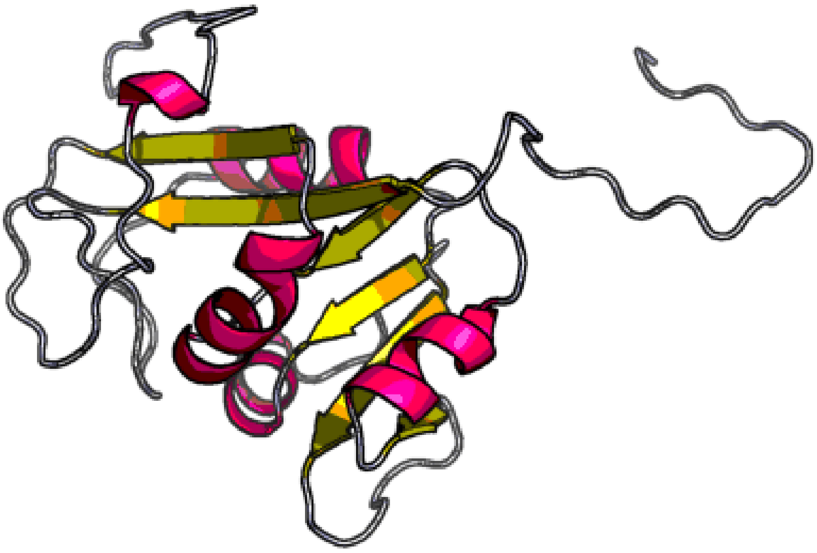

Template: PDB 1rx4A

CP site: Gln146

Target sequence:

QNSHSYCFEILERRGGGGGMISLIAALAVDRVIGMENAMPWNLPADLAWFKRNTLDKP
VIMGRHTWESIGRPLPGRKNIILSSQPGTDDRVTWVKSVDEAIAACGDVPEIMVIGGGR
VYEQFLPKAQKLYLTHIDAEVEGDTHFPDYEPDDWESVFSEFHDADA

Summary

- The input predicted as **1** domain(s)
- Best template: **1tdrA**, p-value **1.00e-08**
- Overall uGDT (GDT): **146** (**89**)
- **164**(**100%**) residues are modeled
- **23**(**14%**) positions predicted as disordered
- Secondary struct: **15%**H, **28%**E, **56%**C
- Solvent access: **39%**E, **29%**M, **31%**B

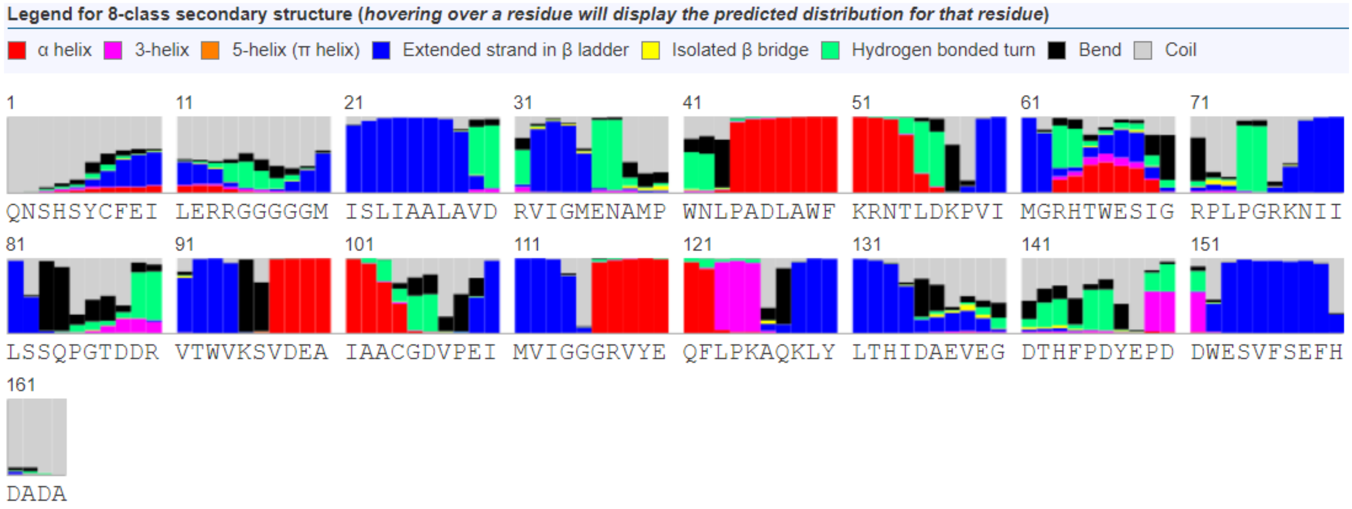

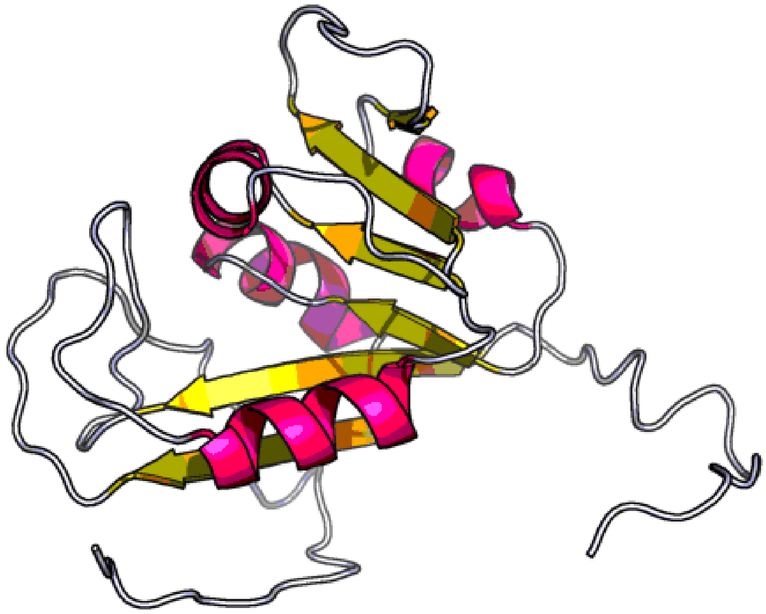

Template: PDB 1rx4A

CP site: Asn147

Target sequence:

NSHSYCFEILERRGGGGGMISLIAALAVDRVIGMENAMPWNLPADLAWFKRNTLDKPVI
MGRHTWESIGRPLPGRKNIILSSQPGTDDRVTWVKSVDEAIAACGDVPEIMVIGGGRVY
EQFLPKAQKLYLTHIDAEVEGDTHFPDYEPDDWESVFSEFHDADAQ

Summary

- The input predicted as **1** domain(s)
- Best template: **1tdrA**, p-value **1.00e-08**
- Overall uGDT (GDT): **146** (**89**)
- **164**(**100%**) residues are modeled
- **23**(**14%**) positions predicted as disordered
- Secondary struct: **15%**H, **28%**E, **56%**C
- Solvent access: **39%**E, **29%**M, **31%**B

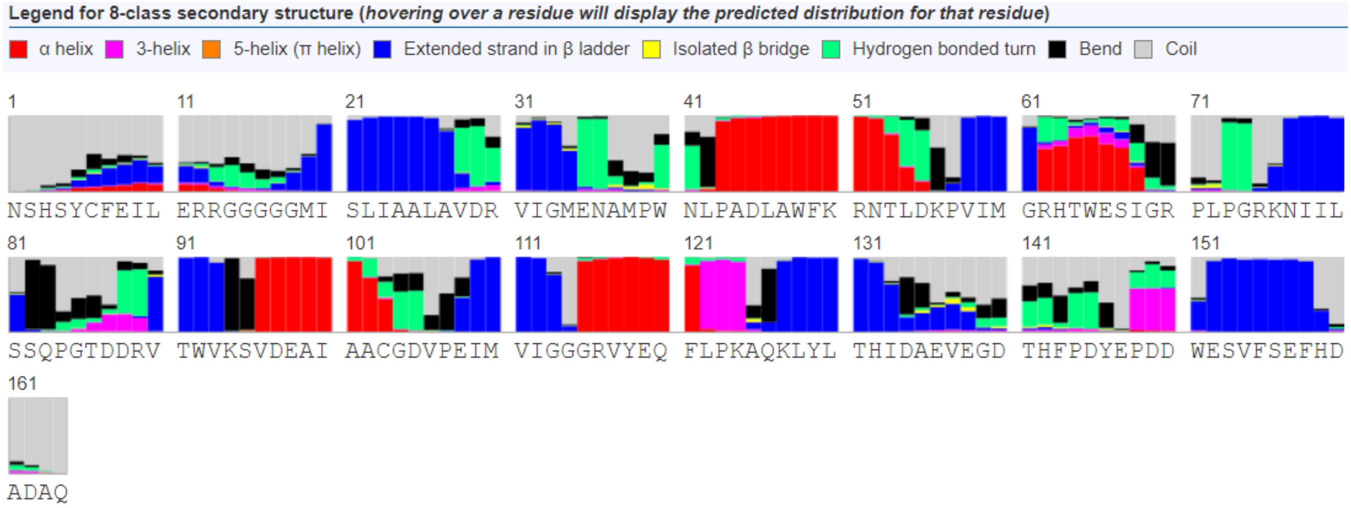

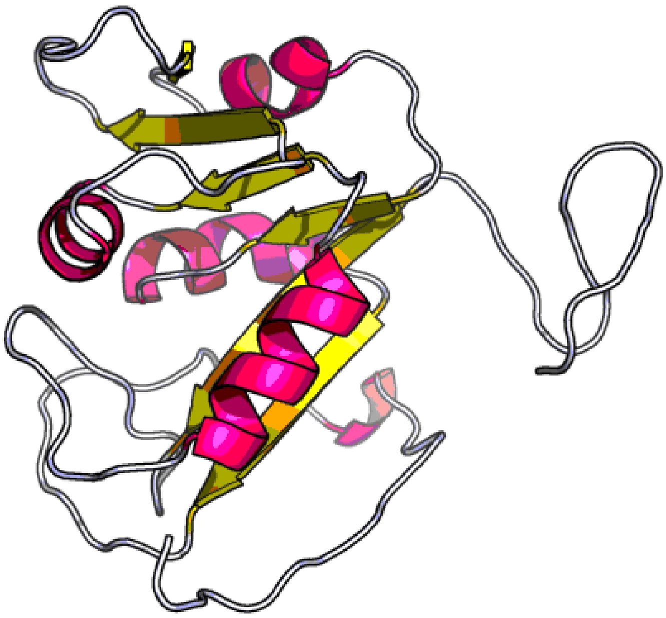

Template: PDB 1rx4A

CP site: Ser148

Target sequence:

SHSYCFEILERRGGGGGMISLIAALAVDRVIGMENAMPWNLPADLAWFKRNTLDKPVIM
GRHTWESIGRPLPGRKNIILSSQPGTDDRVTWVKSVDEAIAACGDVPEIMVIGGGRVYEQ
FLPKAQKLYLTHIDAEVEGDTHFPDYEPDDWESVFSEFHDADAQN

Summary

•The input predicted as **1** domain(s)
•Best template: **1tdrA**, p-value **1.16e-08**
•Overall uGDT (GDT): **148** (**90**)
•**164**(**100%**) residues are modeled
•**23**(**14%**) positions predicted as disordered
•Secondary struct: **17%**H, **26%**E, **56%**C
•Solvent access: **40%**E, **29%**M, **30%**B

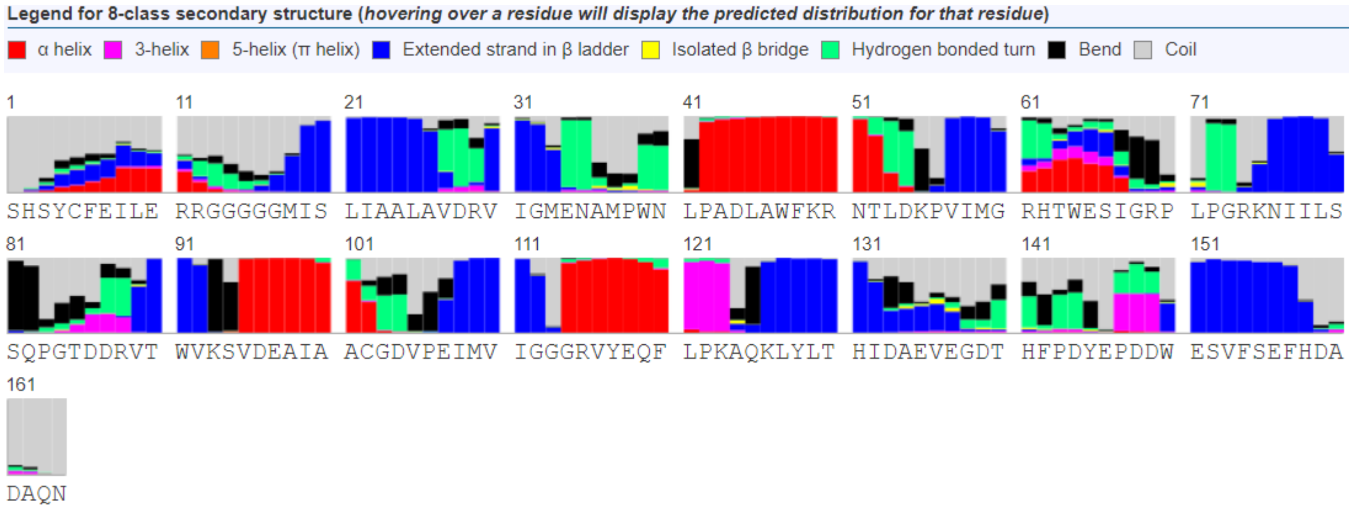

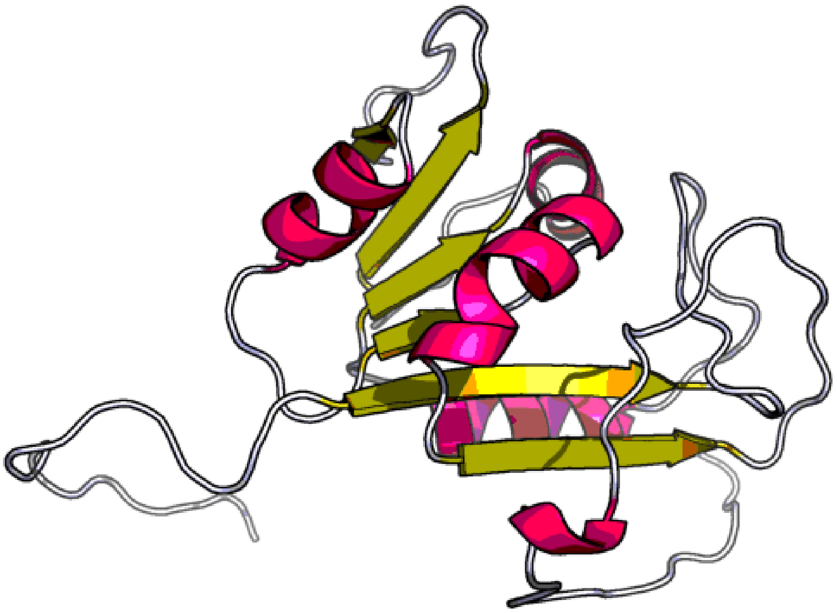

Template: PDB 1rx4A

CP site: His149

Target sequence:

HSYCFEILERRGGGGGMISLIAALAVDRVIGMENAMPWNLPADLAWFKRNTLDKPVIM
GRHTWESIGRPLPGRKNIILSSQPGTDDRVTWVKSVDEAIAACGDVPEIMVIGGGRVYEQ
FLPKAQKLYLTHIDAEVEGDTHFPDYEPDDWESVFSEFHDADAQNS

Summary

- The input predicted as **1** domain(s)
- Best template: **1tdrA**, p-value **1.19e-08**
- Overall uGDT (GDT): **148** (**90**)
- **164**(**100%**) residues are modeled
- **14**(**8%**) positions predicted as disordered
- Secondary struct: **18%**H, **29%**E, **52%**C
- Solvent access: **38%**E, **31%**M, **30%**B

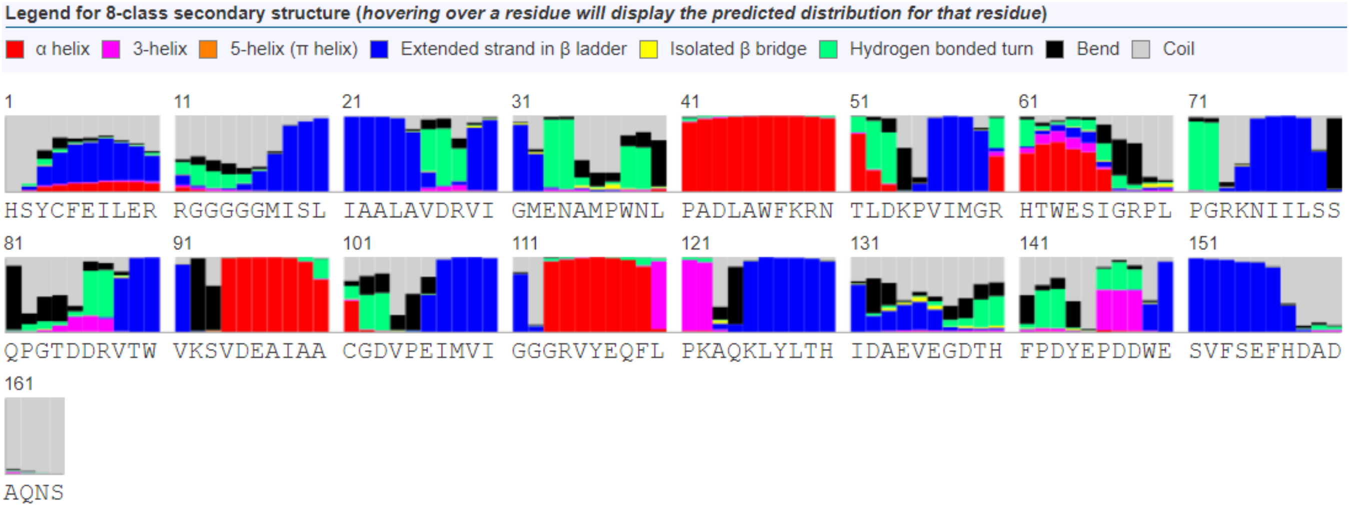

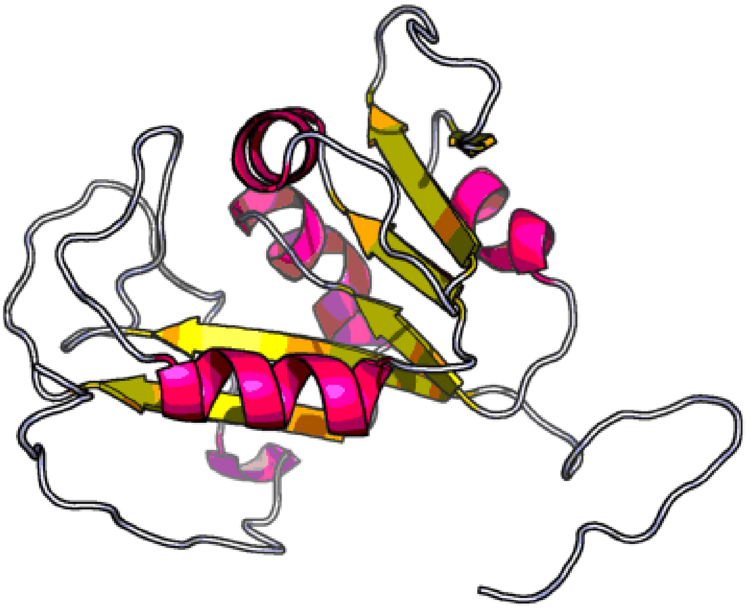

Template: PDB 1rx4A

CP site: Ser150

Target sequence:

SYCFEILERRGGGGGMISLIAALAVDRVIGMENAMPWNLPADLAWFKRNTLDKPVIMGR
HTWESIGRPLPGRKNIILSSQPGTDDRVTWVKSVDEAIAACGDVPEIMVIGGGRVYEQFL
PKAQKLYLTHIDAEVEGDTHFPDYEPDDWESVFSEFHDADAQNSH

Summary

- The input predicted as **1** domain(s)
- Best template: **1tdrA**, p-value **1.13e-08**
- Overall uGDT (GDT): **145** (**88**)
- **164**(**100%**) residues are modeled
- **14**(**8%**) positions predicted as disordered
- Secondary struct: **18%**H, **29%**E, **51%**C
- Solvent access: **41%**E, **28%**M, **30%**B

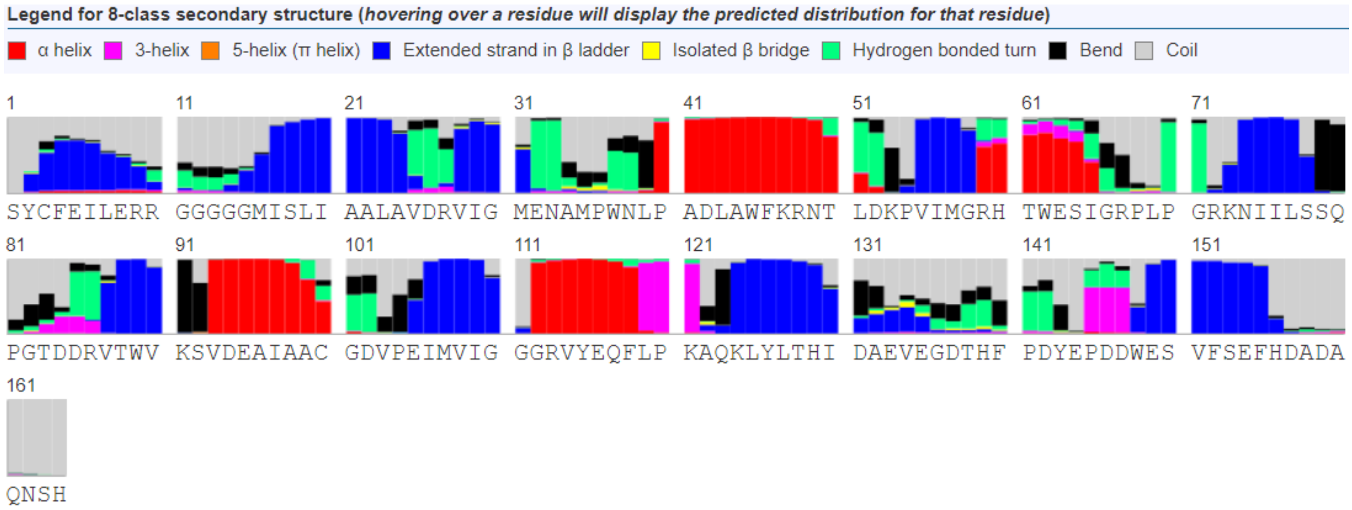

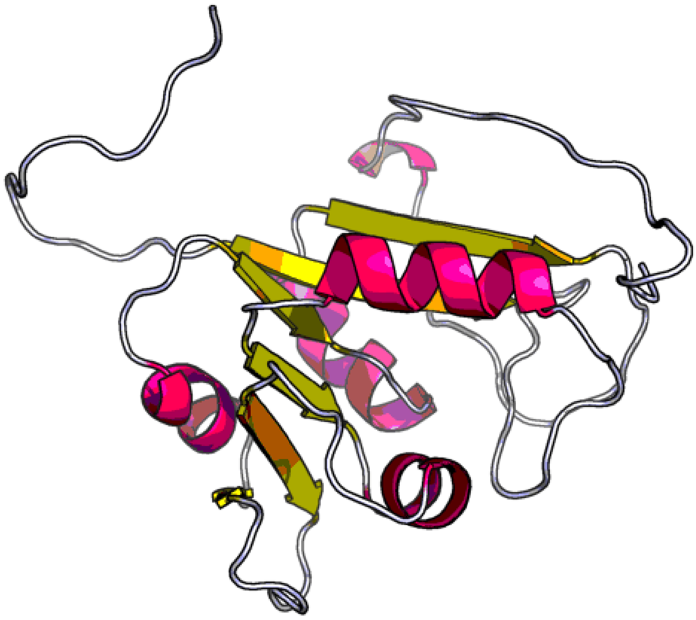

Template: PDB 1rx4A

CP site: Tyr151

Target sequence:

YCFEILERRGGGGGMISLIAALAVDRVIGMENAMPWNLPADLAWFKRNTLDKPVIMGR
HTWESIGRPLPGRKNIILSSQPGTDDRVTWVKSVDEAIAACGDVPEIMVIGGGRVYEQFL
PKAQKLYLTHIDAEVEGDTHFPDYEPDDWESVFSEFHDADAQNSHS

Summary

- The input predicted as **1** domain(s)
- Best template: **1tdrA**, p-value **8.37e-09**
- Overall uGDT (GDT): **147** (**90**)
- **164**(**100%**) residues are modeled
- **25**(**15%**) positions predicted as disordered
- Secondary struct: **15%**H, **29%**E, **54%**C
- Solvent access: **40%**E, **27%**M, **31%**B

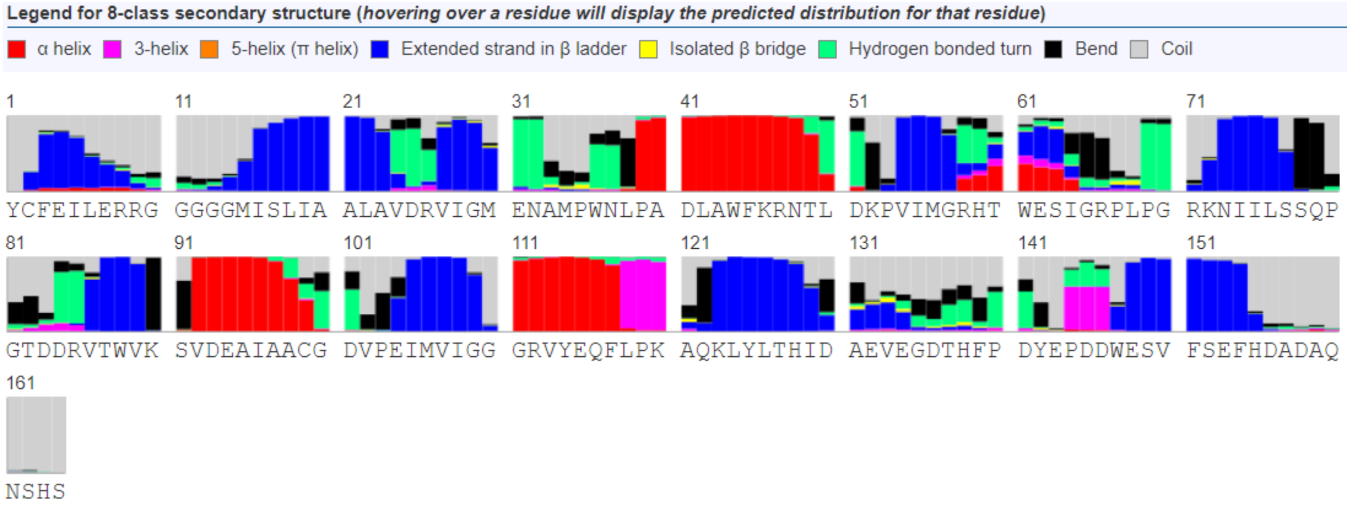

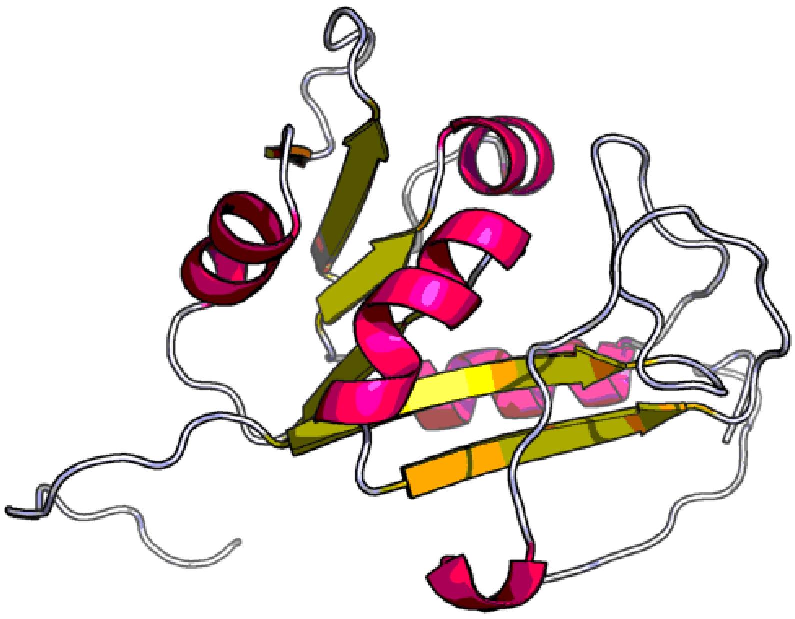

Template: PDB 1rx4A

CP site: Cys152

Target sequence:

CFEILERRGGGGGMISLIAALAVDRVIGMENAMPWNLPADLAWFKRNTLDKPVIMGRH
TWESIGRPLPGRKNIILSSQPGTDDRVTWVKSVDEAIAACGDVPEIMVIGGGRVYEQFLP
KAQKLYLTHIDAEVEGDTHFPDYEPDDWESVFSEFHDADAQNSHSY

Summary

•The input predicted as **1** domain(s)
•Best template: **1tdrA**, p-value **1.52e-08**
•Overall uGDT (GDT): **147** (**90**)
•**164**(**100%**) residues are modeled
•**26**(**15%**) positions predicted as disordered
•Secondary struct: **18%**H, **27%**E, **53%**C
•Solvent access: **40%**E, **29%**M, **29%**B

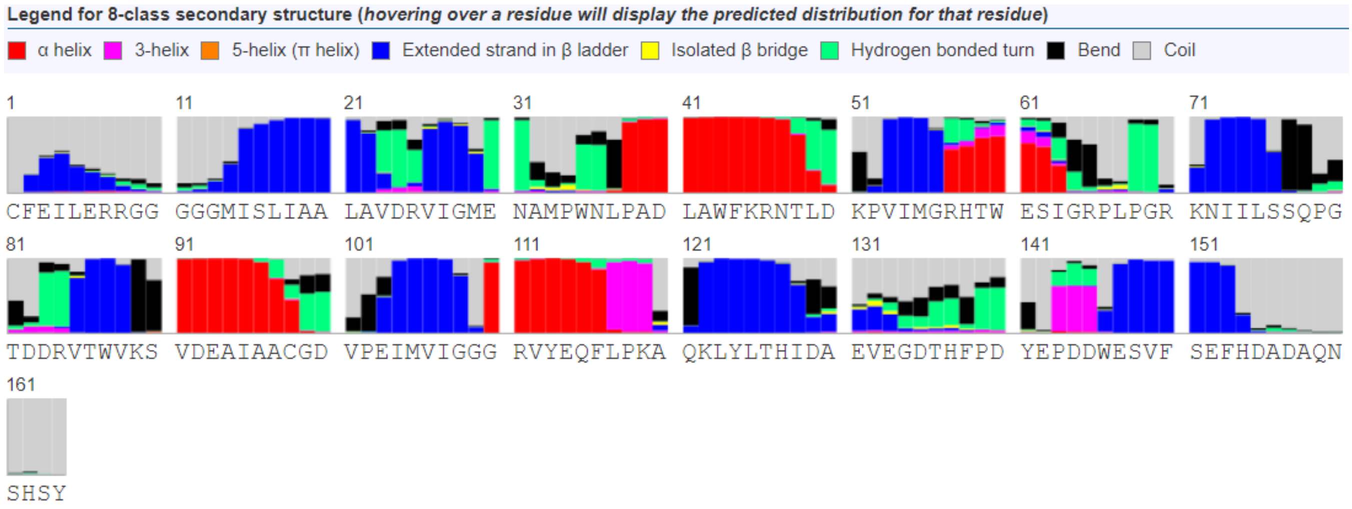

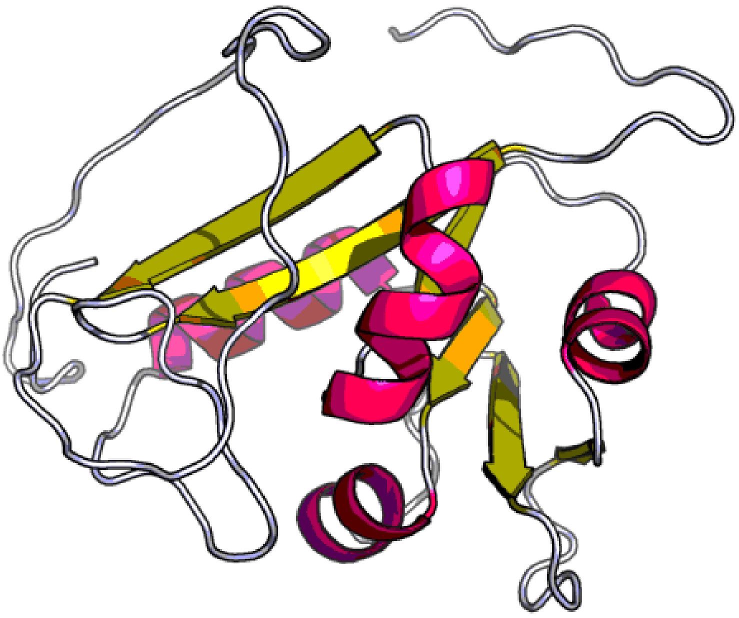

Template: PDB 1rx4A

CP site: Phe153

Target sequence:

FEILERRGGGGMISLIAALAVDRVIGMENAMPWNLPADLAWFKRNTLDKPVIMGRHT
WESIGRPLPGRKNIILSSQPGTDDRVTWVKSVDEAIAACGDVPEIMVIGGGRVYEQFLPK
AQKLYLTHIDAEVEGDTHFPDYEPDDWESVFSEFHDADAQNSHSYC

Summary

- The input predicted as **1** domain(s)
- Best template: **1tdrA**, p-value **8.33e-09**
- Overall uGDT (GDT): **148** (**90**)
- **164**(**100%**) residues are modeled
- **26**(**15%**) positions predicted as disordered
- Secondary struct: **18%**H, **26%**E, **54%**C
- Solvent access: **40%**E, **28%**M, **30%**B

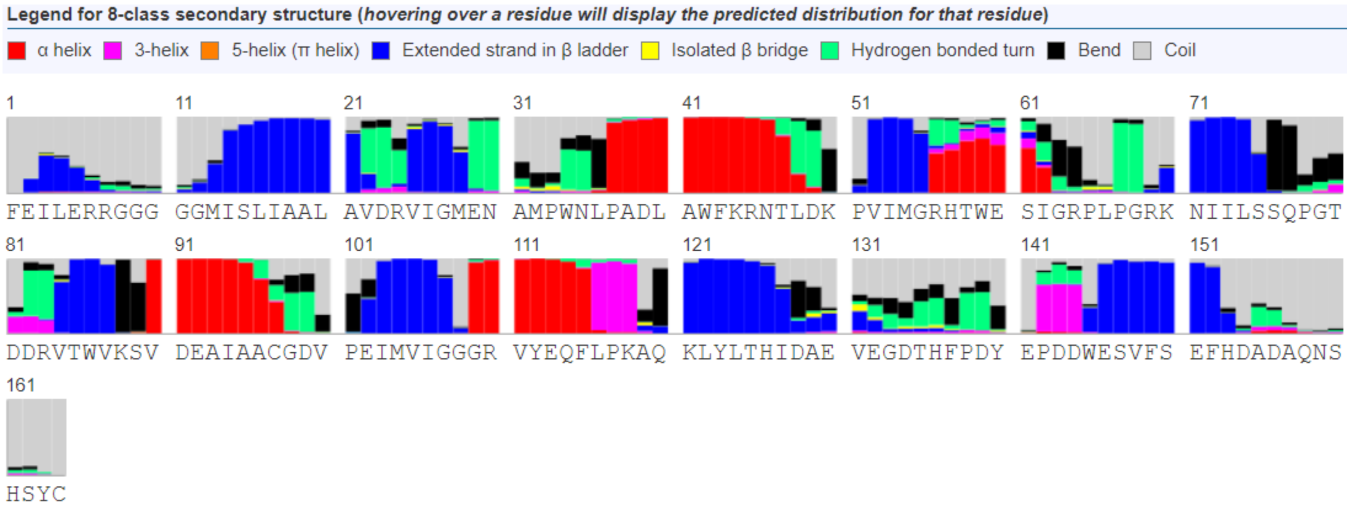

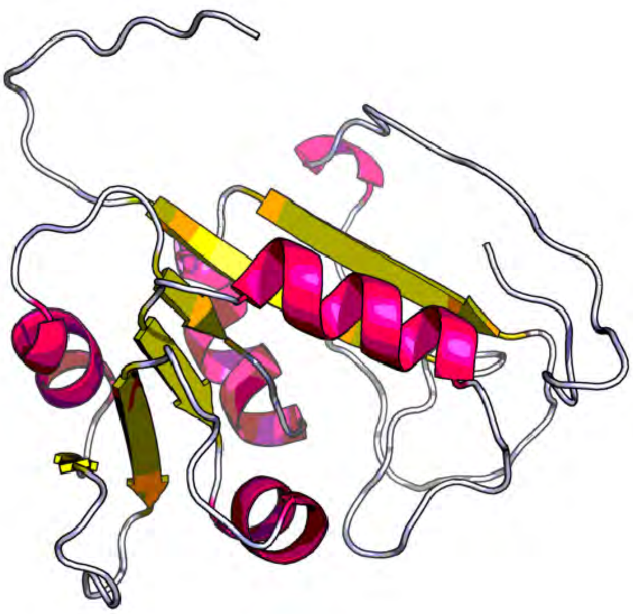

Template: PDB 1rx4A

CP site: Leu156

Target sequence:

LERRGGGGGMISLIAALAVDRVIGMENAMPWNLPADLAWFKRNTLDKPVIMGRHTWE
SIGRPLPGRKNIILSSQPGTDDRVTWVKSVDEAIAACGDVPEIMVIGGGRVYEQFLPKAQK
LYLTHIDAEVEGDTHFPDYEPDDWESVFSEFHDADAQNSHSYCFEI

Summary

- The input predicted as **1** domain(s)
- Best template: **1dreA**, p-value **1.16e-09**
- Overall uGDT (GDT): **161** (**98**)
- **164**(**100%**) residues are modeled
- **8**(**4%**) positions predicted as disordered
- Secondary struct: **17%**H, **31%**E, **51%**C
- Solvent access: **37%**E, **30%**M, **32%**B

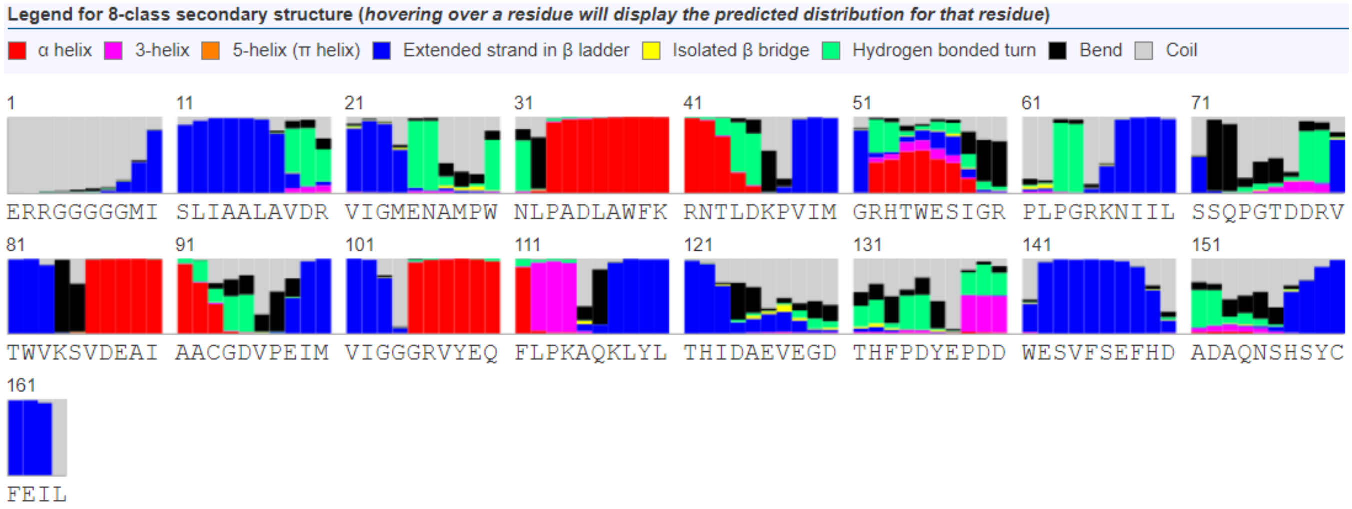

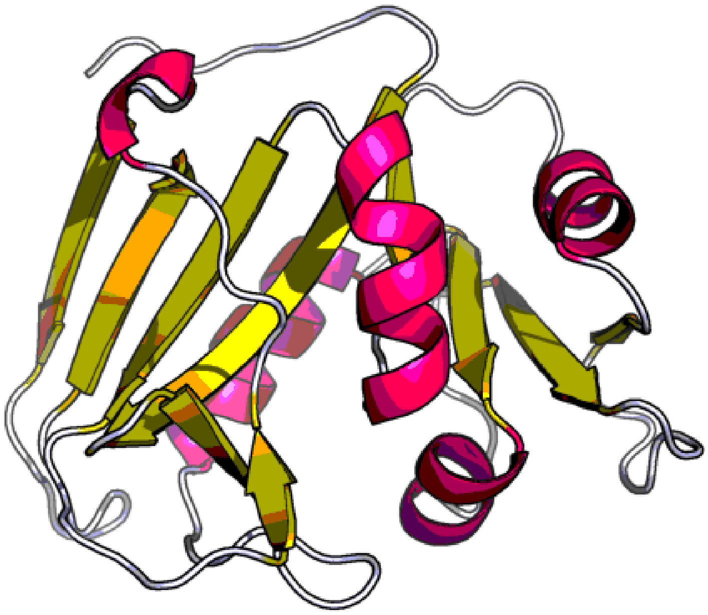

Template: PDB 1rx4A

CP site: Glu157

Target sequence:

ERRGGGGGMISLIAALAVDRVIGMENAMPWNLPADLAWFKRNTLDKPVIMGRHTWES
IGRPLPGRKNIILSSQPGTDDRVTWVKSVDEAIAACGDVPEIMVIGGGRVYEQFLPKAQKL
YLTHIDAEVEGDTHFPDYEPDDWESVFSEFHDADAQNSHSYCFEIL

Summary

- The input predicted as **1** domain(s)
- Best template: **1dreA**, p-value **3.81e-09**
- Overall uGDT (GDT): **157** (**95**)
- **164**(**100%**) residues are modeled
- **15**(**9%**) positions predicted as disordered
- Secondary struct: **18%**H, **30%**E, **50%**C
- Solvent access: **39%**E, **28%**M, **31%**B

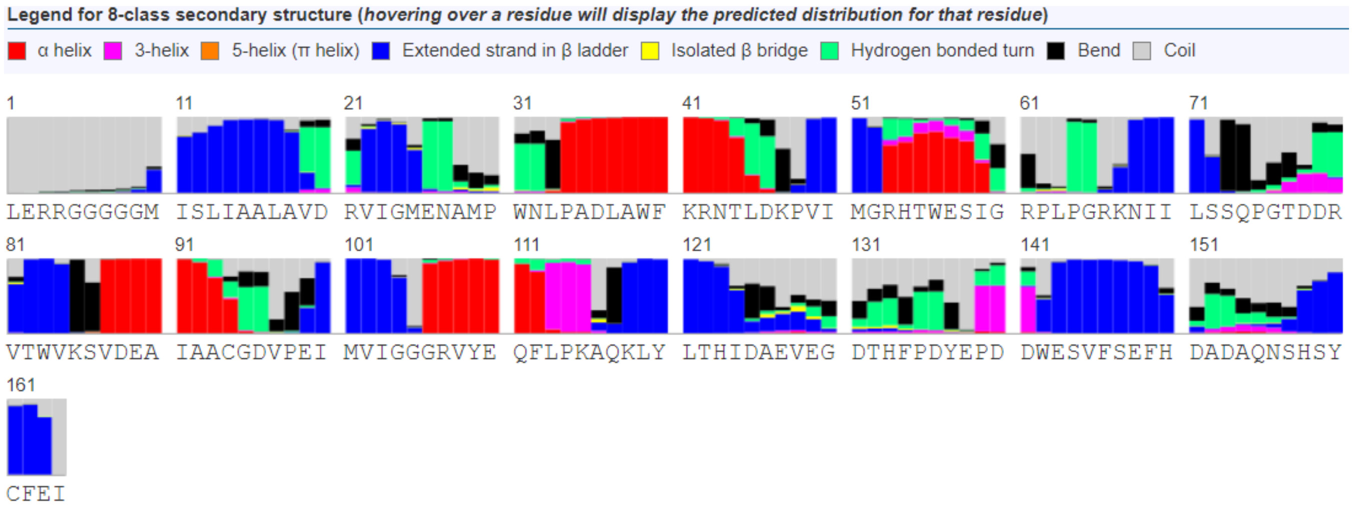

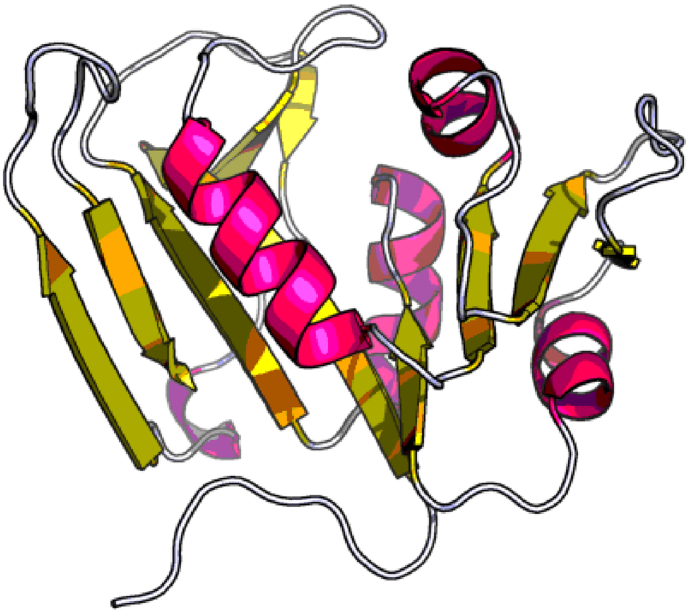

Template: PDB 1rx4A

CP site: Arg158

Target sequence:

RRGGGGGMISLIAALAVDRVIGMENAMPWNLPADLAWFKRNTLDKPVIMGRHTWESI
GRPLPGRKNIILSSQPGTDDRVTWVKSVDEAIAACGDVPEIMVIGGGRVYEQFLPKAQKL
YLTHIDAEVEGDTHFPDYEPDDWESVFSEFHDADAQNSHSYCFEILE

Summary

- •The input predicted as **1** domain(s)
- •Best template: **1dreA**, p-value **1.69e-09**
- •Overall uGDT (GDT): **159** (**97**)
- •**164**(**100%**) residues are modeled
- •**7**(**4%**) positions predicted as disordered
- •Secondary struct: **18%**H, **32%**E, **48%**C
- •Solvent access: **34%**E, **35%**M, **30%**B

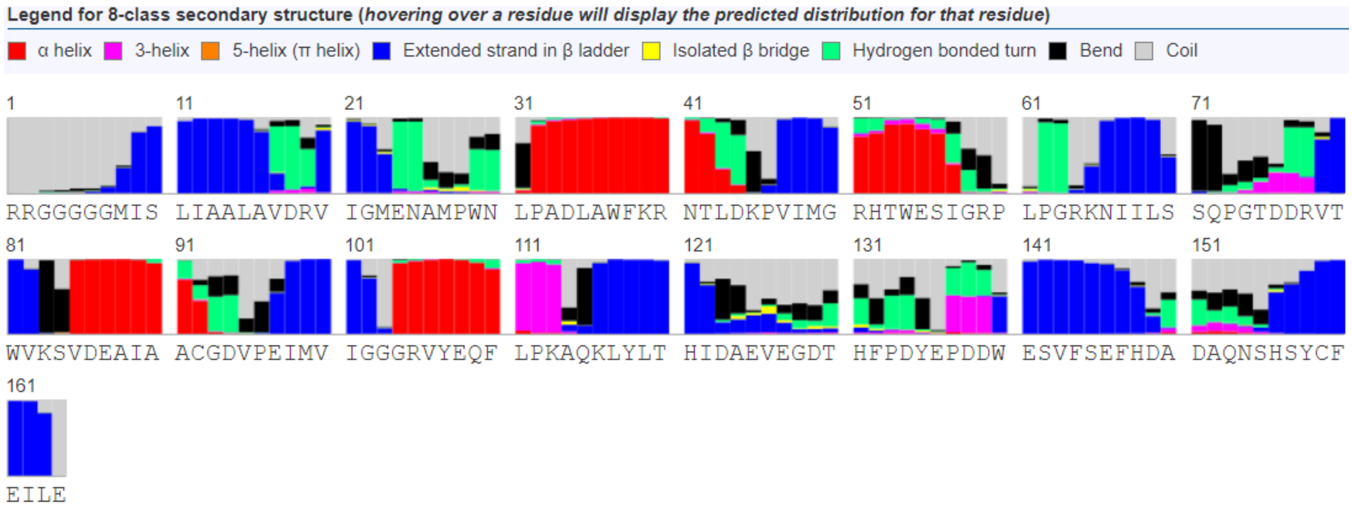

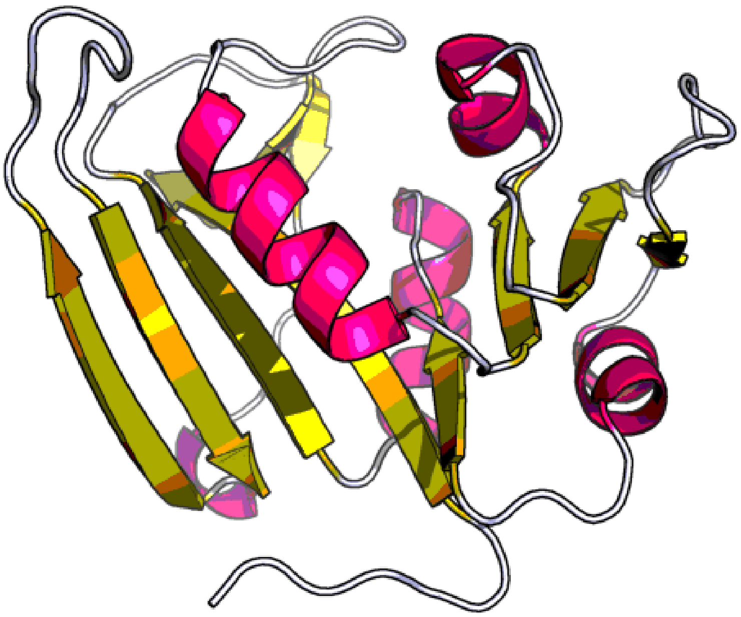

Template: PDB 1rx4A

CP site: Arg159

Target sequence:

RGGGGGMISLIAALAVDRVIGMENAMPWNLPADLAWFKRNTLDKPVIMGRHTWESIG
RPLPGRKNIILSSQPGTDDRVTWVKSVDEAIAACGDVPEIMVIGGGRVYEQFLPKAQKLYL
THIDAEVEGDTHFPDYEPDDWESVFSEFHDADAQNSHSYCFEILER

Summary

- The input predicted as **1** domain(s)
- Best template: **1dreA**, p-value **1.62e-09**
- Overall uGDT (GDT): **161** (**98**)
- **164**(**100%**) residues are modeled
- **6**(**3%**) positions predicted as disordered
- Secondary struct: **18%**H, **32%**E, **48%**C
- Solvent access: **34%**E, **34%**M, **31%**B

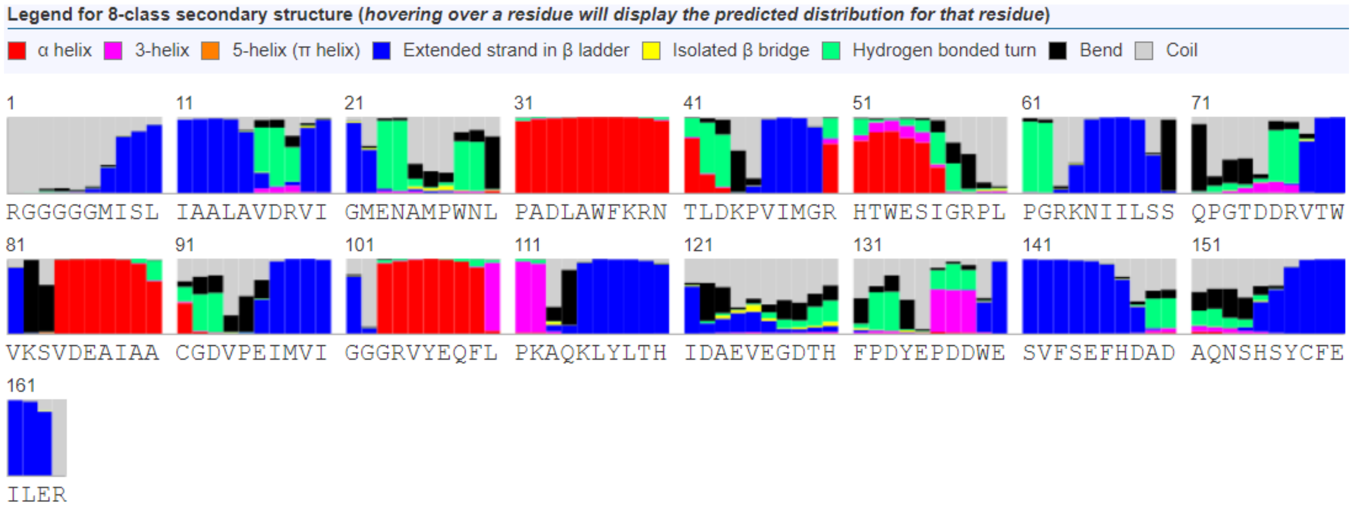

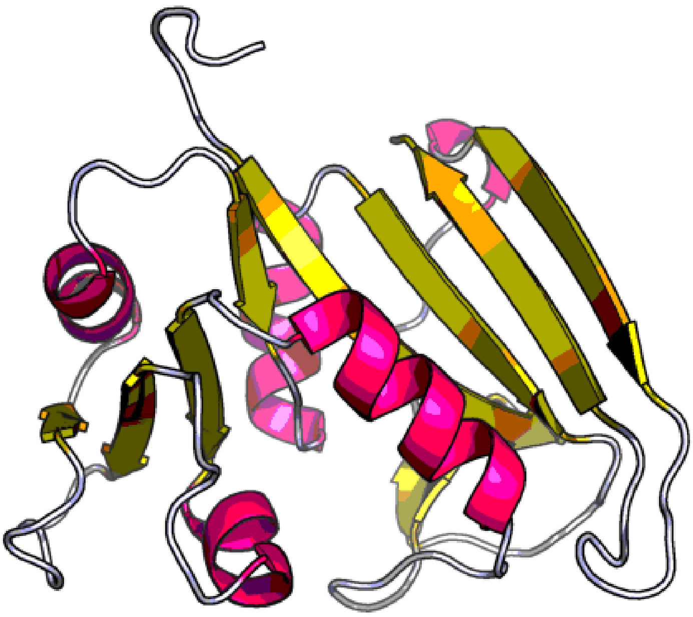

Models constructed by Robetta for viable circular permutants of the dihydrofolate reductase (PDB:1rx4)

References

Robetta

- Srivatsan Raman, Robert Vernon, James Thompson, Michael Tyka, Ruslan Sadreyev,Jimin Pei, David Kim, Elizabeth Kellogg, Frank DiMaio, Oliver Lange, Lisa Kinch, Will Sheffler, Bong-Hyun Kim, Rhiju Das, Nick V. Grishin, and David Baker. (2009) Structure prediction for CASP8 with all-atom refinement using Rosetta. Proteins 77 Suppl 9:89-99.
- Rohl CA and Baker D. (2002) De novo determination of protein backbone structure from residual dipolar couplings using Rosetta. J Am Chem Soc 124:2723-9
- Bowers PM, Strauss CE, Baker D. (2000) De novo protein structure determination using sparse NMR data. J Biomol NMR 18(4):311-8
- Kim DE, Chivian D, Baker D. (2004) Protein structure prediction and analysis using the Robetta server. Nuclei c Acids Res. 32 Suppl 2:W526-31 (WEB SERVER ISSUE)

HHpred (Söding lab)

- Söding J. (2005) Protein homology detection by HMM-HMM comparison. Bioinformatics 21, 951-960. doi:10.1093/bioinformatics/bti125.

RaptorX (Xu lab)

- Morten Källberg, Haipeng Wang, Sheng Wang, Jian Peng, Zhiyong Wang, Hui Lu & Jinbo Xu. Template-based protein structure modeling using the RaptorX web server. Nature Protocols 7, 1511-1522, 2012.

Sparks-X (Zhou lab)

- Yuedong Yang, Eshel Faraggi, Huiying Zhao, Yaoqi Zhou. Improving protein fold recognition and template-based modeling by employing probabilistic-based matching between predicted one-dimensional structural properties of the query and corresponding native properties of templates. Bioinformatics 27:2076-82(2011)

Template: PDB 1rx4A

CP site: Gly15

Target sequence:

GMENAMPWNLPADLAWFKRNTLDKPVIMGRHTWESIGRPLPGRKNIILSSQPGTDDRVTW
VKSVDEAIAACGDVPEIMVIGGGRVYEQFLPKAQKLYLTHIDAEVEGDTHFPDYEPDDWE
SVFSEFHDADAQNSHSYCFEILERRMISLIAALAVDRVI

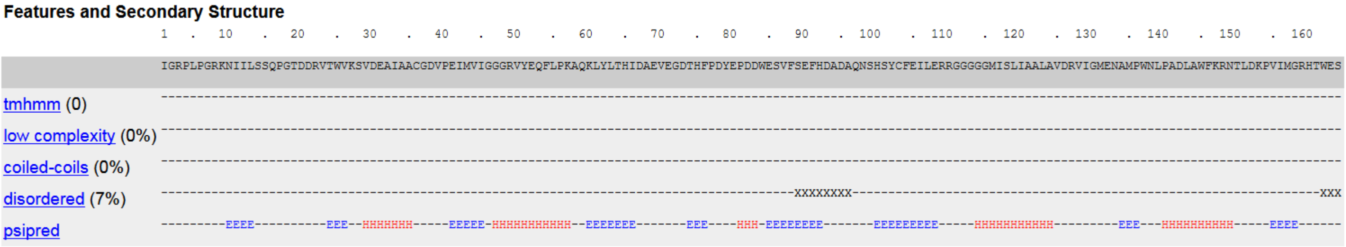

Ginzu Domain Prediction 1

Domain	Span	Source	Reference Parent	Parent Span	Confidence	Annotations
domain 1	1-164	alignment	4qi9A_301	1-158	0.8594	--

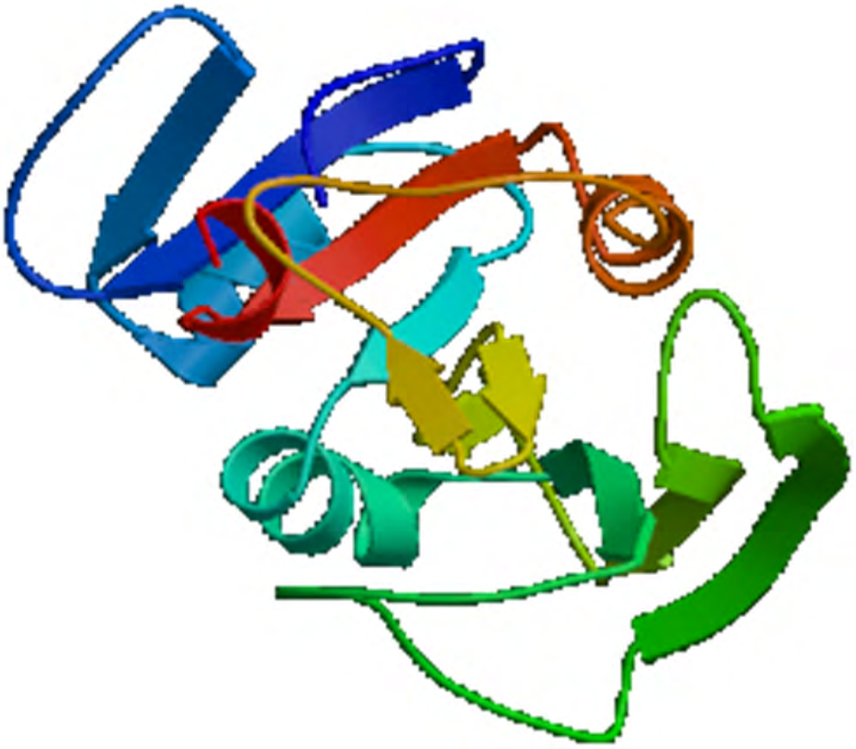

Template: PDB 1rx4A

CP site: Met16

Target sequence:

MENAMPWNLPADLAWFKRNTLDKPVIMGRHTWESIGRPLPGRKNIILSSQPGTDDRVTWV
KSVDEAIAACGDVPEIMVIGGGRVYEQFLPKAQKLYLTHIDAEVEGDTHFPDYEPDDWES
VFSEFHDADAQNSHSYCFEILERRMISLIAALAVDRVIG

Features and Secondary Structure

```
                     1  .  10   .  20   .  30   .  40   .  50   .  60   .  70   .  80   .  90   .  100  .  110  .  120  .  130  .  140  .  150  .
                     MENAMPWNLPADLAWFKRNTLDKPVIMGRHTWESIGRPLPGRKNIILSSQPGTDDRVTWVKSVDEAIAACGDVPEIMVIGGGRVYEQFLPKAQKLYLTHIDAEVEGDTHFPDYEPDDWESVFSEFHDADAQNSHSYCFEILERRMISLIAALAVDRVIG
tmhmm (0)            ---------------------------------------------------------------------------------------------------------------------------------------------------------------
low complexity (0%)  ---------------------------------------------------------------------------------------------------------------------------------------------------------------
coiled-coils (0%)    ---------------------------------------------------------------------------------------------------------------------------------------------------------------
disordered (8%)      ------------------------------------------------------------------------------------------------------------------------XXXXXXXXXXX-X--------------------------
psipred              ---------HHHHHHHHHHH----EEEE--EE-----------EEEEE--------EEEEE-HHHHHHH-----EEEEE--HHHHHHHHHH--EEEEEEE--------------HHH-EEEEEEE----------EEEEEEE-----------------
```

Ginzu Domain Prediction 1 ▲

Domain	Span	Source	Reference Parent	Parent Span	Confidence	Annotations
domain 1	1-159	alignment	3tq8A_202	1-171	0.9015	OXIDOREDUCTASE/OXIDOREDUCTASE INHIBITOR

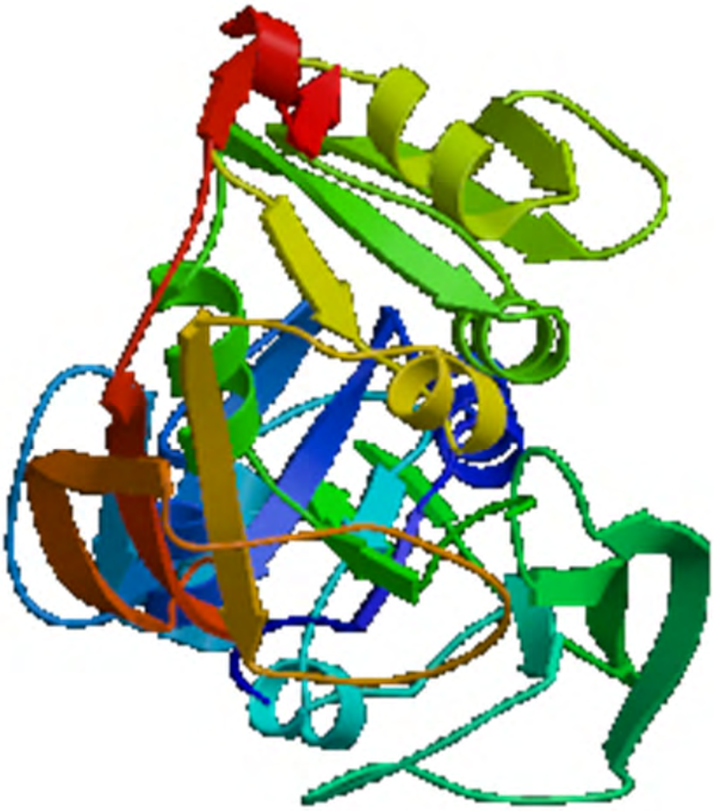

Template: PDB 1rx4A

CP site: Asp27

Target sequence:

DLAWFKRNTLDKPVIMGRHTWESIGRPLPGRKNIILSSQPGTDDRVTWVKSVDEAIAACG
DVPEIMVIGGGRVYEQFLPKAQKLYLTHIDAEVEGDTHFPDYEPDDWESVFSEFHDADAQ
NSHSYCFEILERRMISLIAALAVDRVIGMENAMPWNLPA

Features and Secondary Structure

	1 . 10 . 20 . 30 . 40 . 50 . 60 . 70 . 80 . 90 . 100 . 110 . 120 . 130 . 140 . 150 .
	DLAWFKRNTLDKPVIMGRHTWESIGRPLPGRKNIILSSQPGTDDRVTWVKSVDEAIAACGDVPEIMVIGGGRVYEQFLPKAQKLYLTHIDAEVEGDTHFPDYEPDDWESVFSEFHDADAQNSHSYCFEILERRMISLIAALAVDRVIGMENAMPWNLPA
tmhmm (0)	---
low complexity (0%)	---
coiled-coils (0%)	---
disordered (7%)	---X-XXXXXXXXX--------------------------------------X
psipred	-HHHHHHH-----EEEEE-------------EEEEEE--------EEEE--HHHHHHHH----EEEE--HHHHHHHHHHH--EEEEEEE-----EEEE-----HHH-EEEEEEE----------EEEEEEEE---------------------------

Ginzu Domain Prediction 1 ▲

Domain	Span	Source	Reference Parent	Parent Span	Confidence	Annotations
domain 1	1-159	alignment	4qi9A_301	1-158	0.8365	--

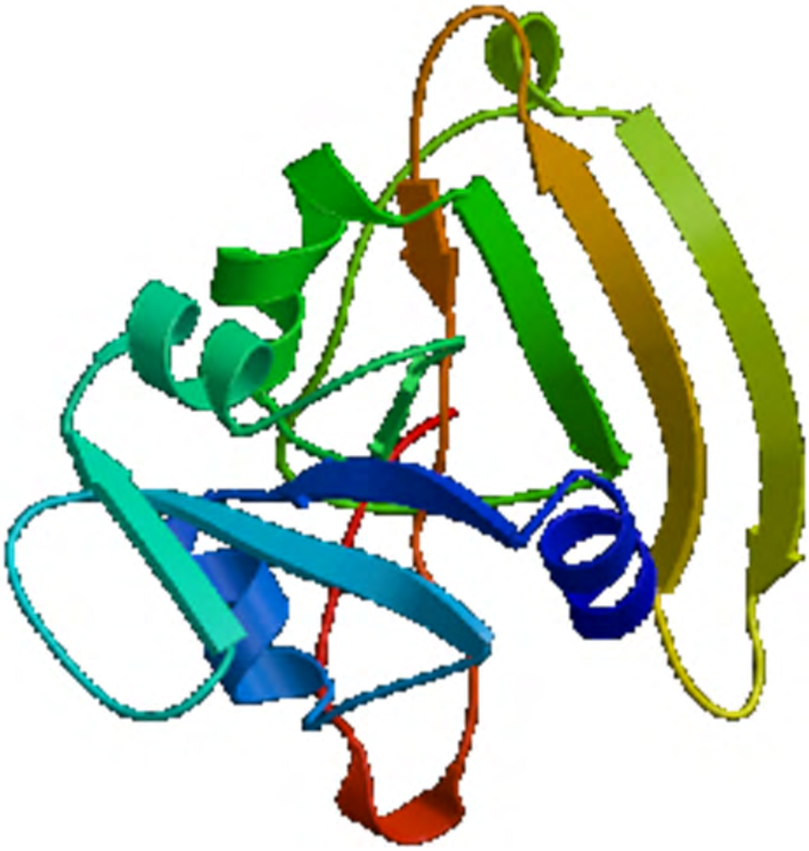

Template: PDB 1rx4A

CP site: Leu28

Target sequence:

```
LAWFKRNTLDKPVIMGRHTWESIGRPLPGRKNIILSSQPGTDDRVTWVKSVDEAIAACGD
VPEIMVIGGGRVYEQFLPKAQKLYLTHIDAEVEGDTHFPDYEPDDWESVFSEFHDADAQN
SHSYCFEILERRMISLIAALAVDRVIGMENAMPWNLPAD
```

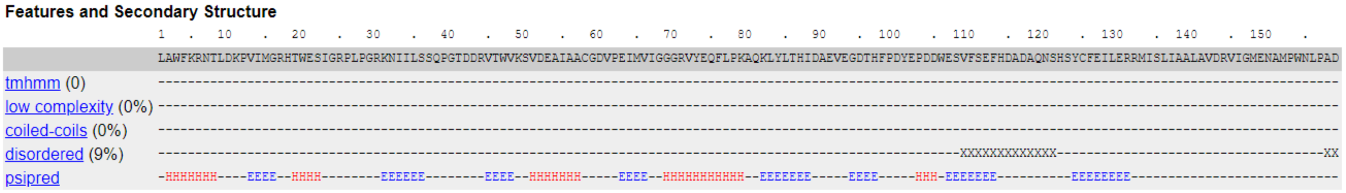

Ginzu Domain Prediction 1

Domain	Span	Source	Reference Parent	Parent Span	Confidence	Annotations
domain 1	1-159	alignment	4qi9A_301	1-158	0.8302	--

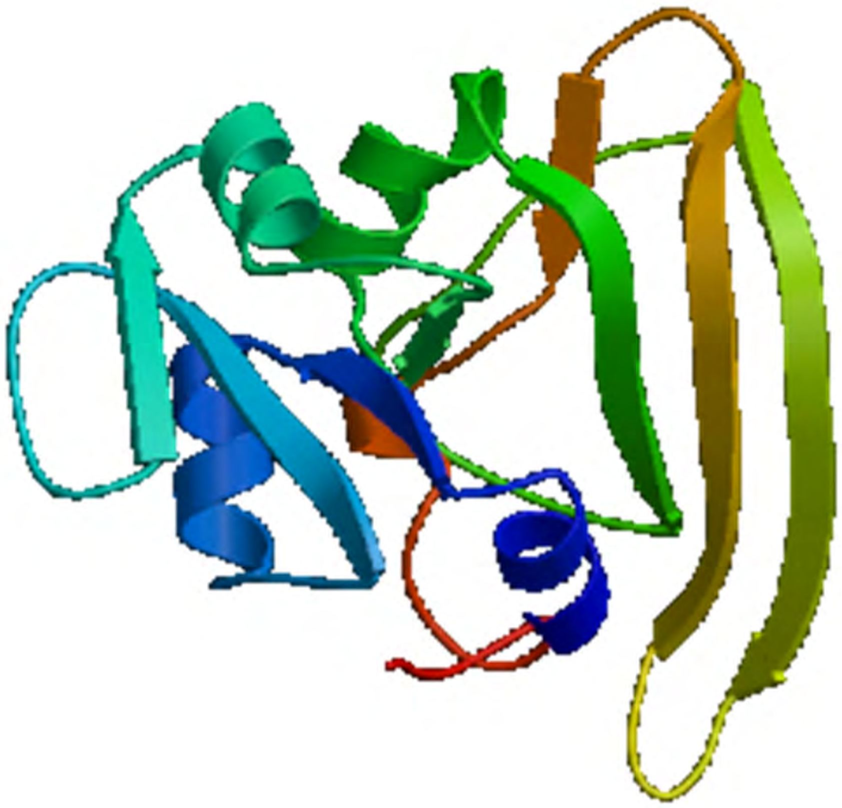

Template: PDB 1rx4A

CP site: Ala29

Target sequence:

```
AWFKRNTLDKPVIMGRHTWESIGRPLPGRKNIILSSQPGTDDRVTWVKSVDEAIAACGDV
PEIMVIGGGRVYEQFLPKAQKLYLTHIDAEVEGDTHFPDYEPDDWESVFSEFHDADAQNS
HSYCFEILERRMISLIAALAVDRVIGMENAMPWNLPADL
```

Features and Secondary Structure

```
                         1   .   10    .   20    .   30    .   40    .   50    .   60    .   70    .   80    .   90    .  100    .  110    .  120    .  130    .  140    .  150    .
                         AWFKRNTLDKPVIMGRHTWESIGRPLPGRKNIILSSQPGTDDRVTWVKSVDEAIAACGDVPEIMVIGGGRVYEQFLPKAQKLYLTHIDAEVEGDTHFPDYEPDDWESVFSEFHDADAQNSHSYCFEILERRMISLIAALAVDRVIGMENAMPWNLPADL
tmhmm (0)                ---------------------------------------------------------------------------------------------------------------------------------------------------------------
low complexity (0%)      ---------------------------------------------------------------------------------------------------------------------------------------------------------------
coiled-coils (0%)        ---------------------------------------------------------------------------------------------------------------------------------------------------------------
disordered (8%)          ------------------------------------------------------------------------------------------------------------XXXXXXXXXXX---------------------------------------X
psipred                  --HHHH-----EEEE--HHHH--------EEEEEE--------EEEE--HHHHHHHH----EEEE--HHHHHHHHHHH--EEEEEEE-----EEEE-----HHH-EEEEEEE---------EEEEEEEE------------------------------
```

Ginzu Domain Prediction 1 ▲

Domain	Span	Source	Reference Parent	Parent Span	Confidence	Annotations
domain 1	1-159	alignment	4qi9A_301	1-158	0.8239	--

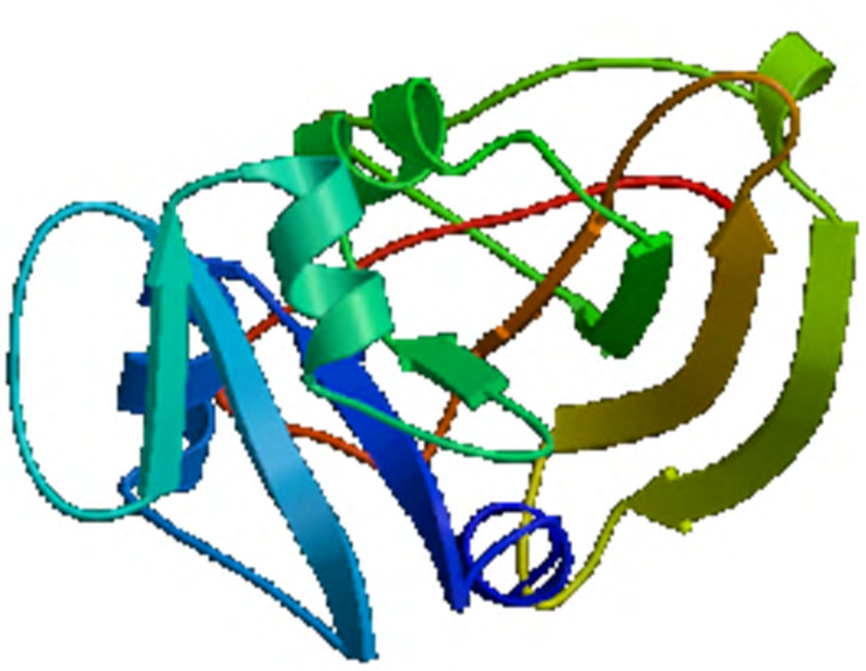

Template: PDB 1rx4A

CP site: Asp37

Target sequence:

DKPVIMGRHTWESIGRPLPGRKNIILSSQPGTDDRVTWVKSVDEAIAACGDVPEIMVIGG
GRVYEQFLPKAQKLYLTHIDAEVEGDTHFPDYEPDDWESVFSEFHDADAQNSHSYCFEIL
ERRMISLIAALAVDRVIGMENAMPWNLPADLAWFKRNTL

Features and Secondary Structure

	1 . 10 . 20 . 30 . 40 . 50 . 60 . 70 . 80 . 90 . 100 . 110 . 120 . 130 . 140 . 150 .
	DKPVIMGRHTWESIGRPLPGRKNIILSSQPGTDDRVTWVKSVDEAIAACGDVPEIMVIGGGRVYEQFLPKAQKLYLTHIDAEVEGDTHFPDYEPDDWESVFSEFHDADAQNSHSYCFEILERRMISLIAALAVDRVIGMENAMPWNLPADLAWFKRNTL
tmhmm (0)	---
low complexity (0%)	---
coiled-coils (0%)	---
disordered (5%)	---XXXXXXXX--
psipred	---EEE--HHHHH--------EEEEEE---------EEE--HHHHHHH-----EEEEE--HHHHHHHHHH--EEEEEEE-------------HHH-EEEEEEE---------EEEEEE-----------------------------HHHHHHHHHH--

Ginzu Domain Prediction 1 ▲

Domain	Span	Source	Reference Parent	Parent Span	Confidence	Annotations
domain 1	1-159	alignment	4qi9A_307	1-158	0.7736	--

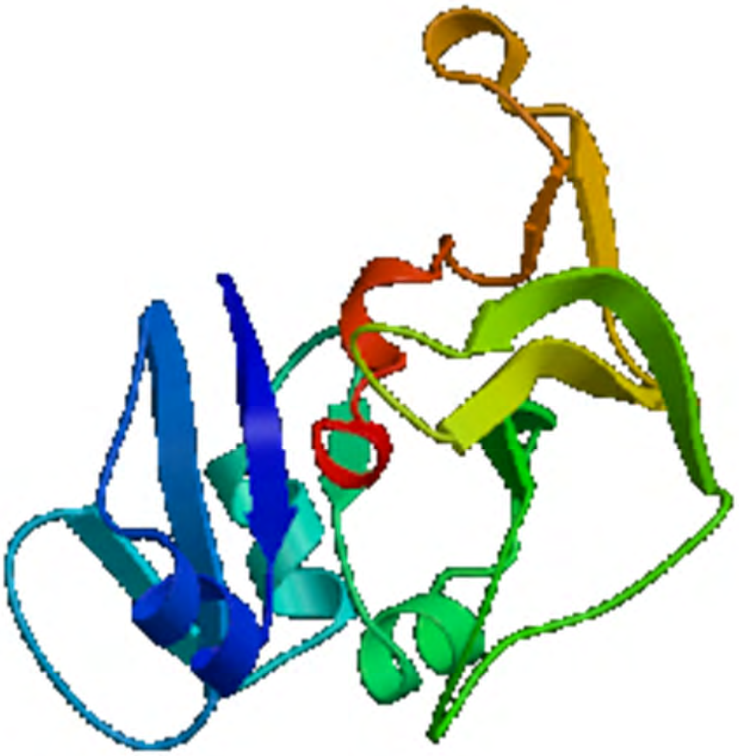

Template: PDB 1rx4A

CP site: Lys38

Target sequence:

KPVIMGRHTWESIGRPLPGRKNIILSSQPGTDDRVTWVKSVDEAIAACGDVPEIMVIGGG
RVYEQFLPKAQKLYLTHIDAEVEGDTHFPDYEPDDWESVFSEFHDADAQNSHSYCFEILE
RRMISLIAALAVDRVIGMENAMPWNLPADLAWFKRNTLD

Features and Secondary Structure

```
                       1   .   10    .   20    .   30    .   40    .   50    .   60    .   70    .   80    .   90    .  100    .  110    .  120    .  130    .  140    .  150    .
                       KPVIMGRHTWESIGRPLPGRKNIILSSQPGTDDRVTWVKSVDEAIAACGDVPEIMVIGGGRVYEQFLPKAQKLYLTHIDAEVEGDTHFPDYEPDDWESVFSEFHDADAQNSHSYCFEILERRMISLIAALAVDRVIGMENAMPWNLPADLAWFKRNTLD
tmhmm (0)              ---------------------------------------------------------------------------------------------------------------------------------------------------------------
low complexity (0%)    ---------------------------------------------------------------------------------------------------------------------------------------------------------------
coiled-coils (0%)      ---------------------------------------------------------------------------------------------------------------------------------------------------------------
disordered (6%)        ----------------------------------------------------------------------------------------------------XXXXXXXX--------------------------------------------------X
psipred                --EEEE--HHHH--------EEEEEE---------EEE--HHHHHHH-----EEEEE--HHHHHHHHHH--EEEEEEE--------------HHH-EEEEEEE----------EEEEEE---------------------------HHHHHHHHHH---
```

Ginzu Domain Prediction 1 ▲

Domain	Span	Source	Reference Parent	Parent Span	Confidence	Annotations
domain 1	1-159	alignment	4qi9A_309	1-158	0.7668	--

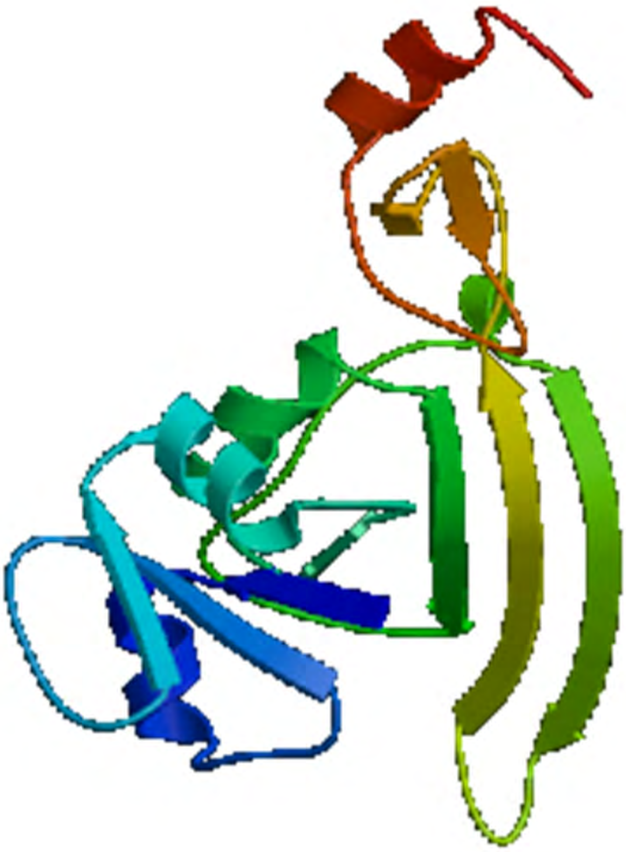

Template: PDB 1rx4A

CP site: Pro39

Target sequence:

PVIMGRHTWESIGRPLPGRKNIILSSQPGTDDRVTWVKSVDEAIAACGDVPEIMVIGGGR
VYEQFLPKAQKLYLTHIDAEVEGDTHFPDYEPDDWESVFSEFHDADAQNSHSYCFEILER
RMISLIAALAVDRVIGMENAMPWNLPADLAWFKRNTLDK

Features and Secondary Structure

	1 . 10 . 20 . 30 . 40 . 50 . 60 . 70 . 80 . 90 . 100 . 110 . 120 . 130 . 140 . 150 .
	PVIMGRHTWESIGRPLPGRKNIILSSQPGTDDRVTWVKSVDEAIAACGDVPEIMVIGGGRVYEQFLPKAQKLYLTHIDAEVEGDTHFPDYEPDDWESVFSEFHDADAQNSHSYCFEILERRMISLIAALAVDRVIGMENAMPWNLPADLAWFKRNTLDK
tmhmm (0)	---
low complexity (0%)	---
coiled-coils (0%)	---
disordered (6%)	---XXXXXXXX---X
psipred	-EEE---HHHH--------EEEEEE---------EEEE-HHHHHHH-----EEEEE--HHHHHHHHHH--EEEEEEE--------------HHH-EEEEEEE----------EEEEEE---------------EE----------HHHHHHHHHH----

Ginzu Domain Prediction 1 ▲

Domain	Span	Source	Reference Parent	Parent Span	Confidence	Annotations
domain 1	1-159	alignment	4qi9A_309	1-158	0.7610	--

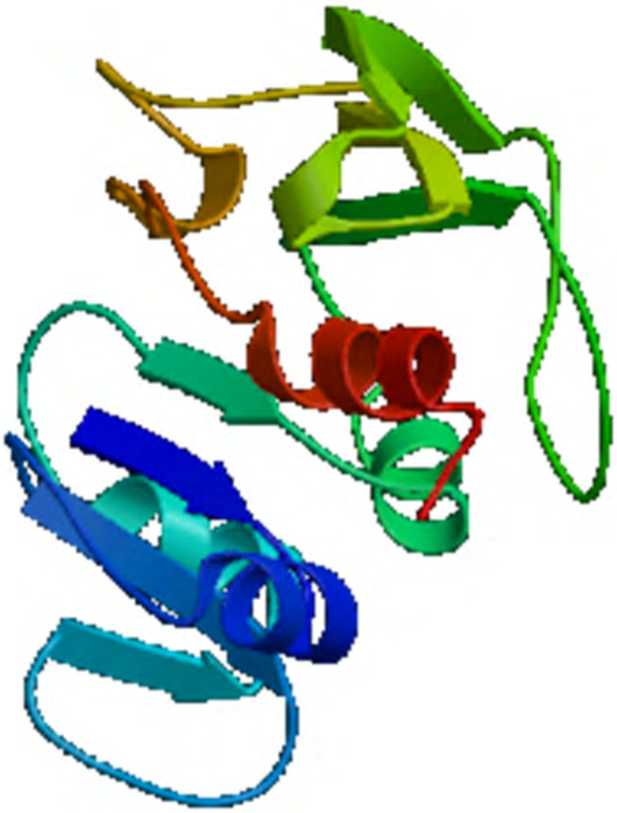

Template: PDB 1rx4A

CP site: Ile50

Target sequence:

```
IGRPLPGRKNIILSSQPGTDDRVTWVKSVDEAIAACGDVPEIMVIGGGRVYEQFLPKAQK
LYLTHIDAEVEGDTHFPDYEPDDWESVFSEFHDADAQNSHSYCFEILERRMISLIAALAV
DRVIGMENAMPWNLPADLAWFKRNTLDKPVIMGRHTWES
```

Features and Secondary Structure

```
                    1  .  10  .  20  .  30  .  40  .  50  .  60  .  70  .  80  .  90  .  100  .  110  .  120  .  130  .  140  .  150  .
                    IGRPLPGRKNIILSSQPGTDDRVTWVKSVDEAIAACGDVPEIMVIGGGRVYEQFLPKAQKLYLTHIDAEVEGDTHFPDYEPDDWESVFSEFHDADAQNSHSYCFEILERRMISLIAALAVDRVIGMENAMPWNLPADLAWFKRNTLDKPVIMGRHTWES
tmhmm (0)           ---------------------------------------------------------------------------------------------------------------------------------------------------------------
low complexity (0%) ---------------------------------------------------------------------------------------------------------------------------------------------------------------
coiled-coils (0%)   ---------------------------------------------------------------------------------------------------------------------------------------------------------------
disordered (7%)     ----------------------------------------------------------------------------------------XXXXXXXX------------------------------------------------------------XXX
psipred             ---------EEEE----------EEE--HHHHHHH-----EEEEE-HHHHHHHHHHH--EEEEEEE--------------HHH-EEEEEE------------EEEE--------HHHH----------------HHHHHHHHHH-----EEEE------
```

Ginzu Domain Prediction 1 ▲

Domain	Span	Source	Reference Parent	Parent Span	Confidence	Annotations
domain 1	1-159	alignment	4qi9B_202	1-159	0.9056	--

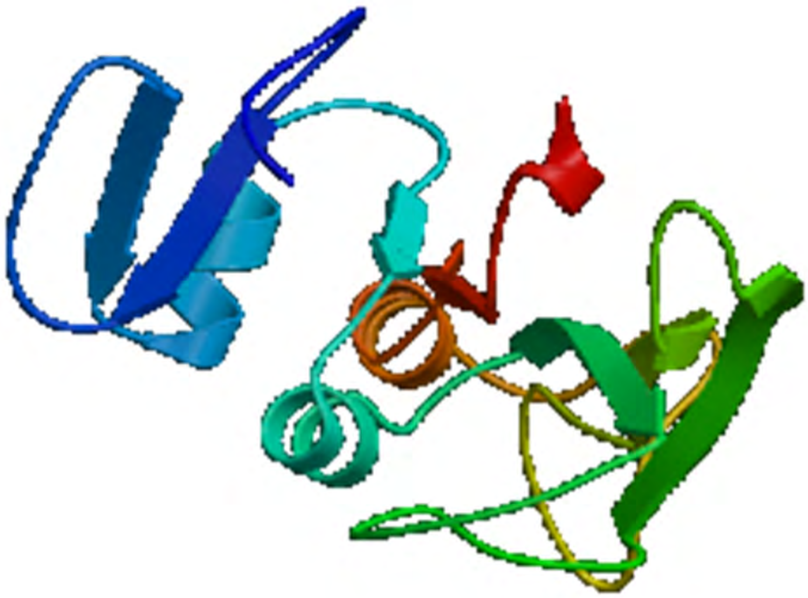

Template: PDB 1rx4A

CP site: Gly51

Target sequence:

```
GRPLPGRKNIILSSQPGTDDRVTWVKSVDEAIAACGDVPEIMVIGGGRVYEQFLPKAQKL
YLTHIDAEVEGDTHFPDYEPDDWESVFSEFHDADAQNSHSYCFEILERRMISLIAALAVD
RVIGMENAMPWNLPADLAWFKRNTLDKPVIMGRHTWESI
```

Features and Secondary Structure

```
                    1   .   10   .   20   .   30   .   40   .   50   .   60   .   70   .   80   .   90   .  100   .  110   .  120   .  130   .  140   .  150   .
                    GRPLPGRKNIILSSQPGTDDRVTWVKSVDEAIAACGDVPEIMVIGGGRVYEQFLPKAQKLYLTHIDAEVEGDTHFPDYEPDDWESVFSEFHDADAQNSHSYCFEILERRMISLIAALAVDRVIGMENAMPWNLPADLAWFKRNTLDKPVIMGRHTWESI
tmhmm (0)           ---------------------------------------------------------------------------------------------------------------------------------------------------------------
low complexity (0%) ---------------------------------------------------------------------------------------------------------------------------------------------------------------
coiled-coils (0%)   ---------------------------------------------------------------------------------------------------------------------------------------------------------------
disordered (6%)     ---------------------------------------------------------------------------------------XXXXXXXX--------------------------------------------------------------XX
psipred             --------EEEE----------EEE--HHHHHHHHH----EEEE-HHHHHHHHHHH--EEEEEEE--------------HHH-EEEEEE------------EEE--------HHHHH----------------HHHHHHHHHH-----EEEE-------
```

Ginzu Domain Prediction 1 ▲

Domain	Span	Source	Reference Parent	Parent Span	Confidence	Annotations
domain 1	1-159	alignment	3tq8A_202	1-171	0.8972	OXIDOREDUCTASE/OXIDOREDUCTASE INHIBITOR

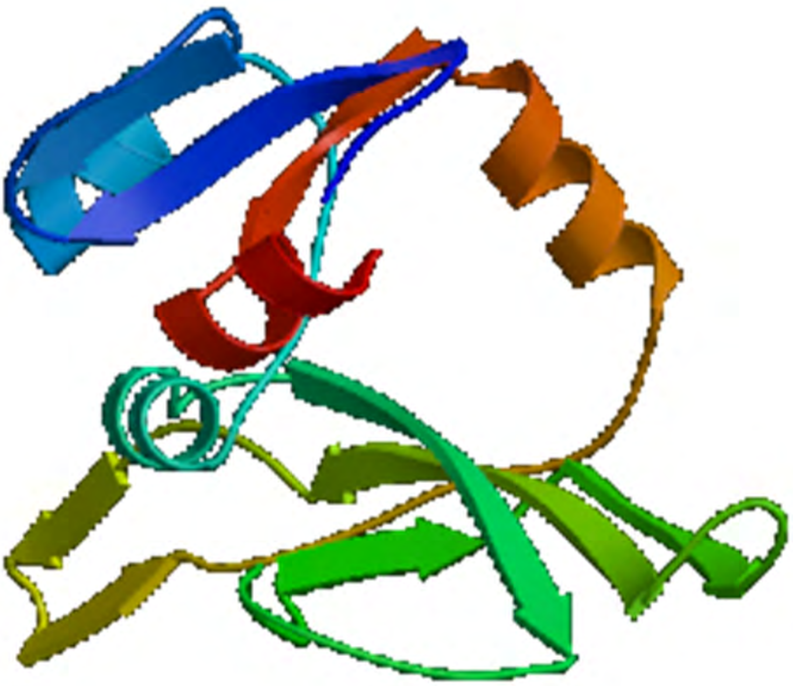

Template: PDB 1rx4A

CP site: Pro55

Target sequence:

```
PGRKNIILSSQPGTDDRVTWVKSVDEAIAACGDVPEIMVIGGGRVYEQFLPKAQKLYLTH
IDAEVEGDTHFPDYEPDDWESVFSEFHDADAQNSHSYCFEILERRMISLIAALAVDRVIG
MENAMPWNLPADLAWFKRNTLDKPVIMGRHTWESIGRPL
```

Features and Secondary Structure

	1 . 10 . 20 . 30 . 40 . 50 . 60 . 70 . 80 . 90 . 100 . 110 . 120 . 130 . 140 . 150 .
	PGRKNIILSSQPGTDDRVTWVKSVDEAIAACGDVPEIMVIGGGRVYEQFLPKAQKLYLTHIDAEVEGDTHFPDYEPDDWESVFSEFHDADAQNSHSYCFEILERRMISLIAALAVDRVIGMENAMPWNLPADLAWFKRNTLDKPVIMGRHTWESIGRPL
tmhmm (0)	---
low complexity (0%)	---
coiled-coils (0%)	---
disordered (6%)	X--X-XXXXXXXX--
psipred	----EEEE----------EEE--HHHHHHHH-----EEEE-HHHHHHHHHHH--EEEEEEE--------EE----HHH-EEEEEE-----------EEHH---------HHHHHHHH------EE----HHHHHHHHH-----EEEEE---HHH-----

Ginzu Domain Prediction 1 ▲

Domain	Span	Source	Reference Parent	Parent Span	Confidence	Annotations
domain 1	1-159	alignment	4qi9A_309	1-158	0.8750	--

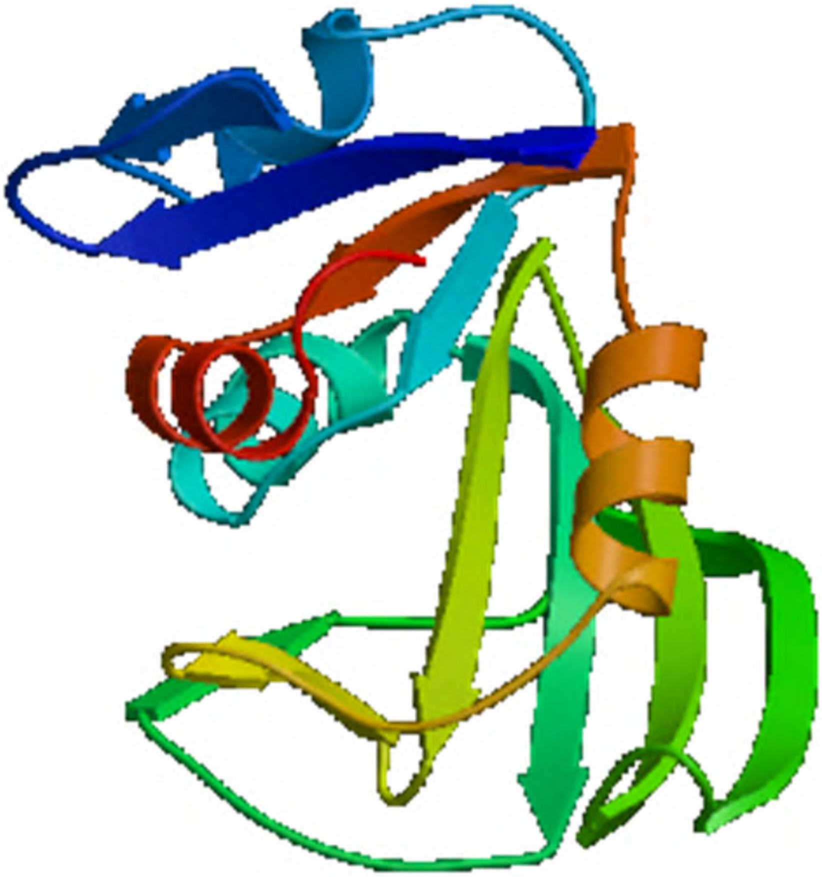

Template: PDB 1rx4A

CP site: Gly56

Target sequence:

GRKNIILSSQPGTDDRVTWVKSVDEAIAACGDVPEIMVIGGGRVYEQFLPKAQKLYLTHI
DAEVEGDTHFPDYEPDDWESVFSEFHDADAQNSHSYCFEILERRMISLIAALAVDRVIGM
ENAMPWNLPADLAWFKRNTLDKPVIMGRHTWESIGRPLP

Features and Secondary Structure

	1 . 10 . 20 . 30 . 40 . 50 . 60 . 70 . 80 . 90 . 100 . 110 . 120 . 130 . 140 . 150 .
	GRKNIILSSQPGTDDRVTWVKSVDEAIAACGDVPEIMVIGGGRVYEQFLPKAQKLYLTHIDAEVEGDTHFPDYEPDDWESVFSEFHDADAQNSHSYCFEILERRMISLIAALAVDRVIGMENAMPWNLPADLAWFKRNTLDKPVIMGRHTWESIGRPLP
tmhmm (0)	---
low complexity (0%)	---
coiled-coils (0%)	---
disordered (7%)	--X-XXXXXXXXX---X
psipred	---EEEE----------EEE--HHHHHHHH-----EEEE-HHHHHHHHHHH--EEEEEEE--EEEEEEEEE------EEEEEEE------------EE-------HHHHHHH-HHHH-----------HHHHHHHHH-----EEEEE---HHH------

Ginzu Domain Prediction 1 ▲

Domain	Span	Source	Reference Parent	Parent Span	Confidence	Annotations
domain 1	1-159	alignment	3tq8A_202	1-171	0.9123	OXIDOREDUCTASE/OXIDOREDUCTASE INHIBITOR

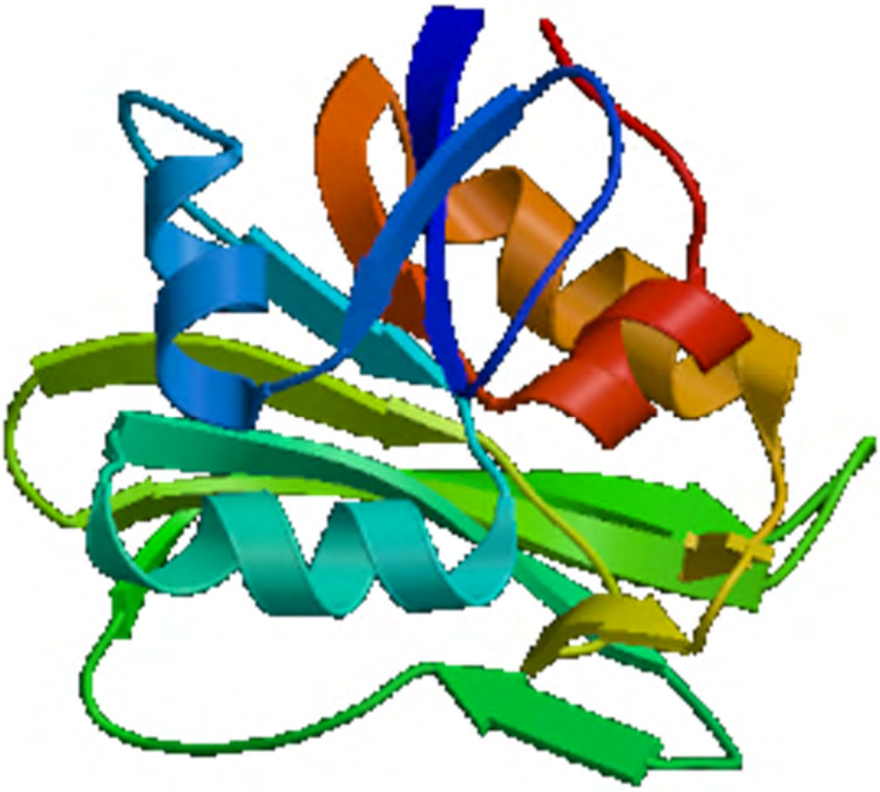

Template: PDB 1rx4A

CP site: Ser64

Target sequence:

SQPGTDDRVTWVKSVDEAIAACGDVPEIMVIGGGRVYEQFLPKAQKLYLTHIDAEVEGDT
HFPDYEPDDWESVFSEFHDADAQNSHSYCFEILERRMISLIAALAVDRVIGMENAMPWNL
PADLAWFKRNTLDKPVIMGRHTWESIGRPLPGRKNIILS

Features and Secondary Structure

	1 . 10 . 20 . 30 . 40 . 50 . 60 . 70 . 80 . 90 . 100 . 110 . 120 . 130 . 140 . 150 .
	SQPGTDDRVTWVKSVDEAIAACGDVPEIMVIGGGRVYEQFLPKAQKLYLTHIDAEVEGDTHFPDYEPDDWESVFSEFHDADAQNSHSYCFEILERRMISLIAALAVDRVIGMENAMPWNLPADLAWFKRNTLDKPVIMGRHTWESIGRPLPGRKNIILS
tmhmm (0)	---
low complexity (0%)	---
coiled-coils (0%)	---
disordered (9%)	XX--XXXXXXXXXXXXX--
psipred	--------EEEE--HHHHHHHH----EEEEE-HHHHHHHHHHH--EEEEEEE--------------HHH----------------------------HHHHHH-----EE----------HHHHHHHHHH-----EEE--HHHHH--------EEEEE-

Ginzu Domain Prediction 1 ▲

Domain	Span	Source	Reference Parent	Parent Span	Confidence	Annotations
domain 1	1-159	alignment	3tq8A_202	1-171	0.9016	OXIDOREDUCTASE/OXIDOREDUCTASE INHIBITOR

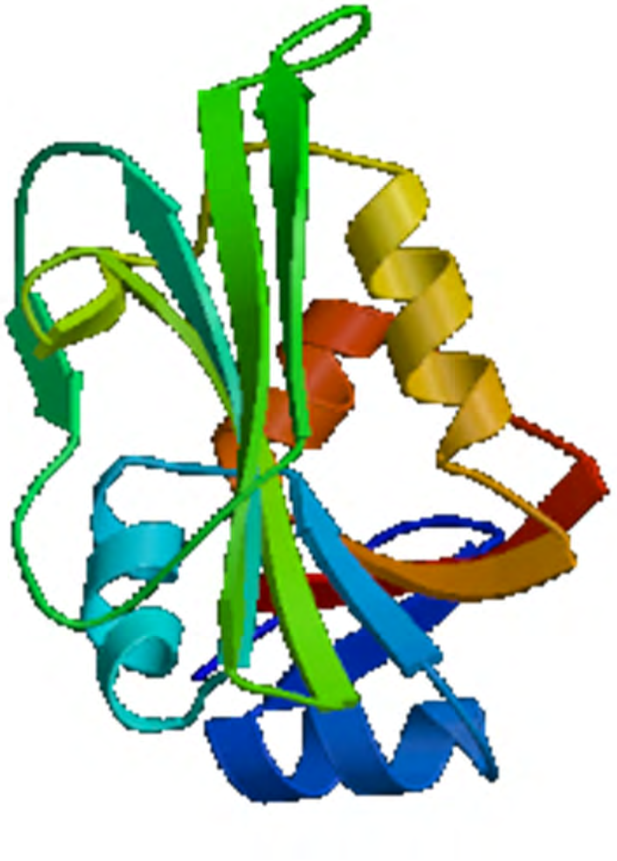

Template: PDB 1rx4A

CP site: Thr73

Target sequence:

TWVKSVDEAIAACGDVPEIMVIGGGRVYEQFLPKAQKLYLTHIDAEVEGDTHFPDYEPDD
WESVFSEFHDADAQNSHSYCFEILERRMISLIAALAVDRVIGMENAMPWNLPADLAWFKR
NTLDKPVIMGRHTWESIGRPLPGRKNIILSSQPGTDDRV

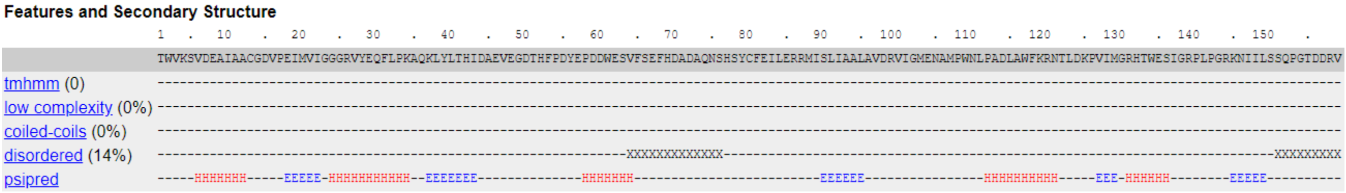

Ginzu Domain Prediction 1

Domain	Span	Source	Reference Parent	Parent Span	Confidence	Annotations
domain 1	1-159	alignment	4qi9B_202	1-159	0.8700	--

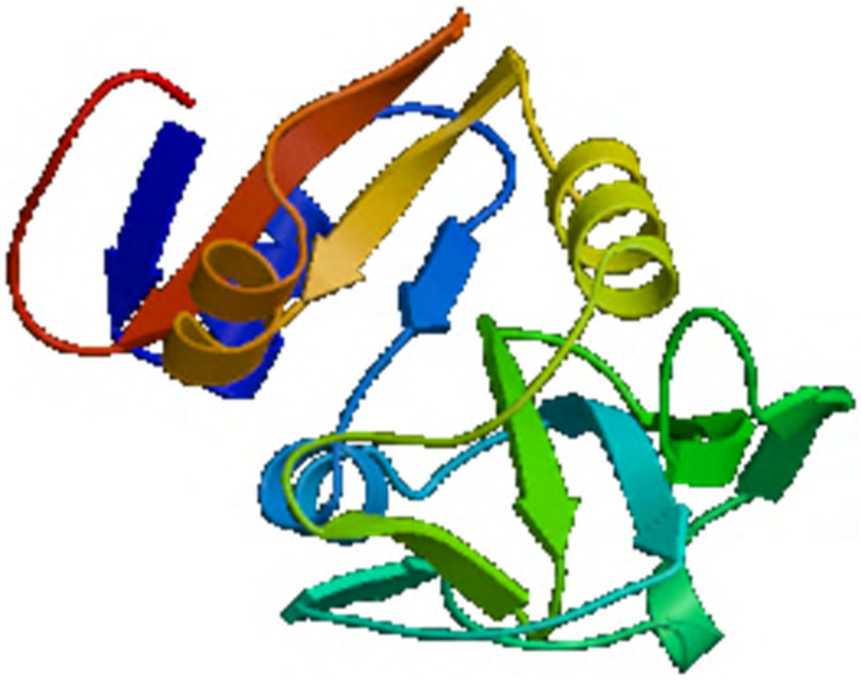

Template: PDB 1rx4A

CP site: Trp74

Target sequence:

WVKSVDEAIAACGDVPEIMVIGGGRVYEQFLPKAQKLYLTHIDAEVEGDTHFPDYEPDDW
ESVFSEFHDADAQNSHSYCFEILERRMISLIAALAVDRVIGMENAMPWNLPADLAWFKRN
TLDKPVIMGRHTWESIGRPLPGRKNIILSSQPGTDDRVT

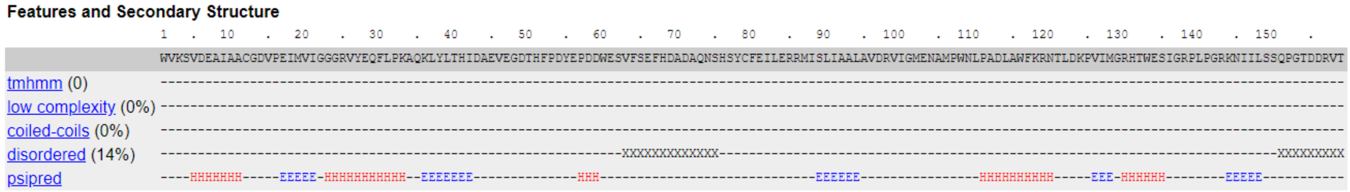

Ginzu Domain Prediction 1

Domain	Span	Source	Reference Parent	Parent Span	Confidence	Annotations
domain 1	1-159	alignment	4qi9B_202	1-159	0.8467	--

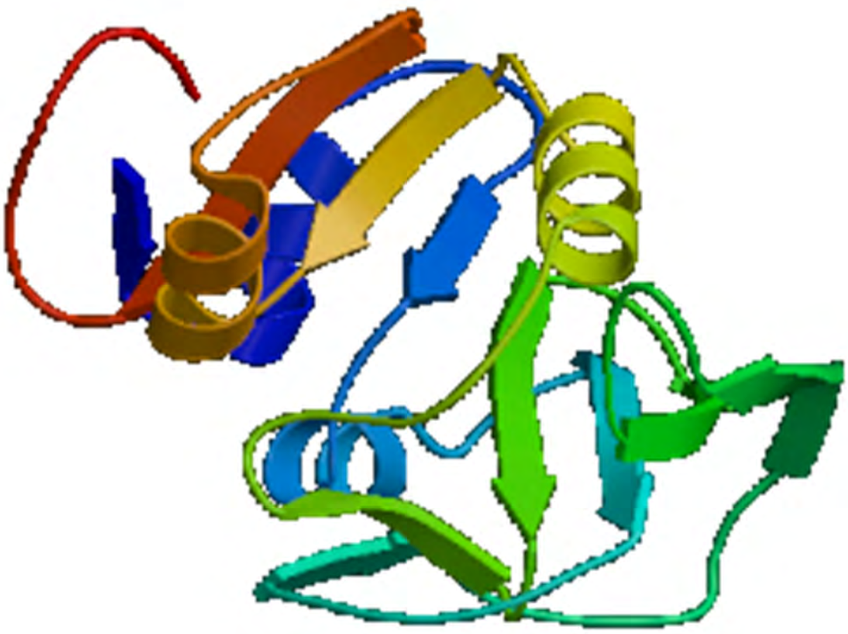

Template: PDB 1rx4A

CP site: Ser77

Target sequence:

SVDEAIAACGDVPEIMVIGGGRVYEQFLPKAQKLYLTHIDAEVEGDTHFPDYEPDDWESV
FSEFHDADAQNSHSYCFEILERRMISLIAALAVDRVIGMENAMPWNLPADLAWFKRNTLD
KPVIMGRHTWESIGRPLPGRKNIILSSQPGTDDRVTWVK

Features and Secondary Structure

	1 . 10 . 20 . 30 . 40 . 50 . 60 . 70 . 80 . 90 . 100 . 110 . 120 . 130 . 140 . 150 .
	SVDEAIAACGDVPEIMVIGGGRVYEQFLPKAQKLYLTHIDAEVEGDTHFPDYEPDDWESVFSEFHDADAQNSHSYCFEILERRMISLIAALAVDRVIGMENAMPWNLPADLAWFKRNTLDKPVIMGRHTWESIGRPLPGRKNIILSSQPGTDDRVTWVK
tmhmm (0)	---
low complexity (0%)	---
coiled-coils (0%)	---
disordered (9%)	---XXXXXXXXXXXXX---X-----
psipred	-HHHHHHH-----EEEEE-HHHHHHHHHHH--EEEEEEE--------------HHH-----EE----------------------EEEEEE----------------HHHHHHHHHH-----EEE-HHHHHH--------EEEEE----------EEE-

Ginzu Domain Prediction 1 ▲

Domain	Span	Source	Reference Parent	Parent Span	Confidence	Annotations
domain 1	1-159	alignment	3tq8A_202	1-171	0.8737	OXIDOREDUCTASE/OXIDOREDUCTASE INHIBITOR

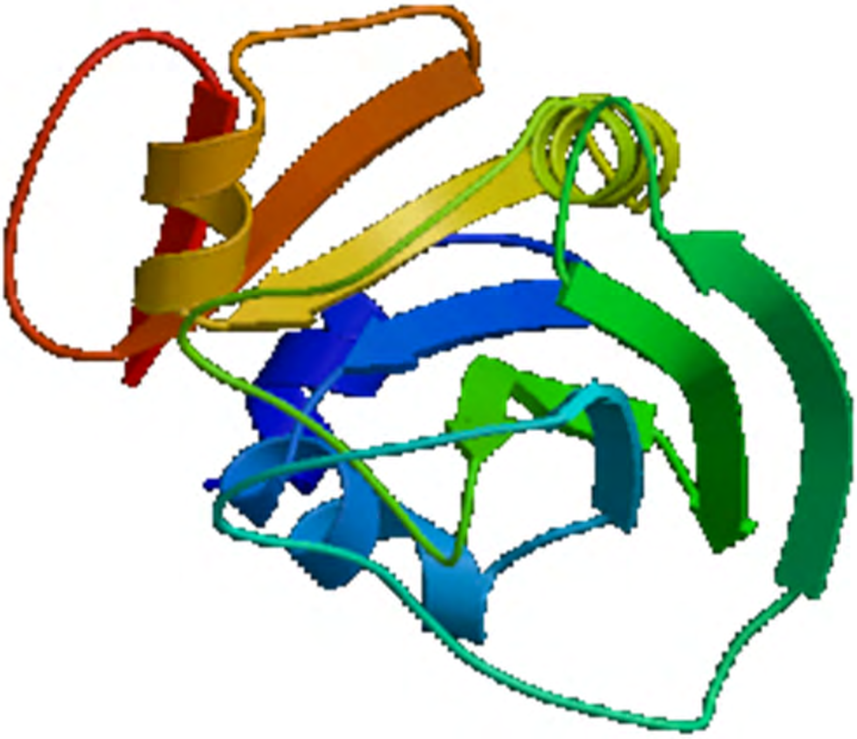

Template: PDB 1rx4A

CP site: Val88

Target sequence:

VPEIMVIGGGRVYEQFLPKAQKLYLTHIDAEVEGDTHFPDYEPDDWESVFSEFHDADAQN
SHSYCFEILERRMISLIAALAVDRVIGMENAMPWNLPADLAWFKRNTLDKPVIMGRHTWE
SIGRPLPGRKNIILSSQPGTDDRVTWVKSVDEAIAACGD

Features and Secondary Structure

	1 . 10 . 20 . 30 . 40 . 50 . 60 . 70 . 80 . 90 . 100 . 110 . 120 . 130 . 140 . 150 .
	VPEIMVIGGGRVYEQFLPKAQKLYLTHIDAEVEGDTHFPDYEPDDWESVFSEFHDADAQNSHSYCFEILERRMISLIAALAVDRVIGMENAMPWNLPADLAWFKRNTLDKPVIMGRHTWESIGRPLPGRKNIILSSQPGTDDRVTWVKSVDEAIAACGD
tmhmm (0)	---
low complexity (0%)	---
coiled-coils (0%)	---
disordered (8%)	--XXXXXXXXXXXXX--
psipred	--EEEEEE-HHHHHHHHHH--EEEEEEE--------EE----HHH------------------------------EEEEE----------EE----HHHHHHHHHH-----EEE---HHH----------EEEE----------EEE--HHHHHHHH--

Ginzu Domain Prediction 1 ▲

Domain	Span	Source	Reference Parent	Parent Span	Confidence	Annotations
domain 1	1-159	alignment	5uioB_201	1-160	1.0000	--

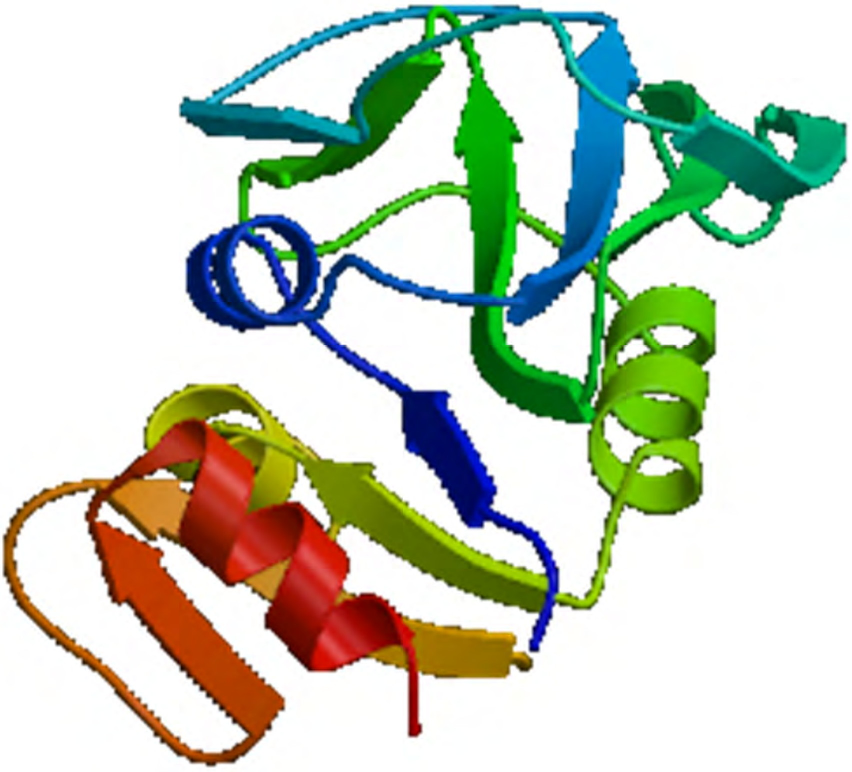

Template: PDB 1rx4A

CP site: Pro89

Target sequence:

PEIMVIGGGRVYEQFLPKAQKLYLTHIDAEVEGDTHFPDYEPDDWESVFSEFHDADAQNS
HSYCFEILERRMISLIAALAVDRVIGMENAMPWNLPADLAWFKRNTLDKPVIMGRHTWES
IGRPLPGRKNIILSSQPGTDDRVTWVKSVDEAIAACGDV

Features and Secondary Structure

	1 . 10 . 20 . 30 . 40 . 50 . 60 . 70 . 80 . 90 . 100 . 110 . 120 . 130 . 140 . 150 .
	PEIMVIGGGRVYEQFLPKAQKLYLTHIDAEVEGDTHFPDYEPDDWESVFSEFHDADAQNSHSYCFEILERRMISLIAALAVDRVIGMENAMPWNLPADLAWFKRNTLDKPVIMGRHTWESIGRPLPGRKNIILSSQPGTDDRVTWVKSVDEAIAACGDV
tmhmm (0)	---
low complexity (0%)	---
coiled-coils (0%)	---
disordered (8%)	---XXXXXXXXXXXXX---
psipred	-EEEEE-HHHHHHHHHHH--EEEEEEE--------HH----HHH------------------------------EEEEE----------EE----HHHHHHHHHH-----EEE---HHH----------EEEE----------EEE--HHHHHHHHH--

Ginzu Domain Prediction 1 ▲

Domain	Span	Source	Reference Parent	Parent Span	Confidence	Annotations
domain 1	1-159	alignment	5dxvA_201	1-149	0.3915	--

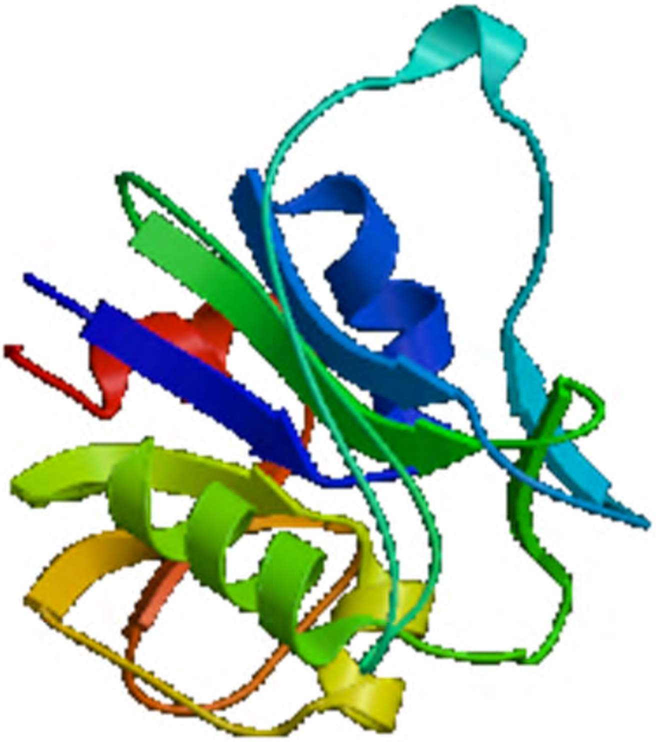

Template: PDB 1rx4A

CP site: Glu90

Target sequence:

EIMVIGGGRVYEQFLPKAQKLYLTHIDAEVEGDTHFPDYEPDDWESVFSEFHDADAQNSH
SYCFEILERRMISLIAALAVDRVIGMENAMPWNLPADLAWFKRNTLDKPVIMGRHTWESI
GRPLPGRKNIILSSQPGTDDRVTWVKSVDEAIAACGDVP

Features and Secondary Structure

```
                      1   .  10   .  20   .  30   .  40   .  50   .  60   .  70   .  80   .  90   . 100   . 110   . 120   . 130   . 140   . 150   .
                      EIMVIGGGRVYEQFLPKAQKLYLTHIDAEVEGDTHFPDYEPDDWESVFSEFHDADAQNSHSYCFEILERRMISLIAALAVDRVIGMENAMPWNLPADLAWFKRNTLDKPVIMGRHTWESIGRPLPGRKNIILSSQPGTDDRVTWVKSVDEAIAACGDVP
tmhmm (0)             ---------------------------------------------------------------------------------------------------------------------------------------------------------------
low complexity (0%)   ---------------------------------------------------------------------------------------------------------------------------------------------------------------
coiled-coils (0%)     ---------------------------------------------------------------------------------------------------------------------------------------------------------------
disordered (9%)       ----------------------------------------------XXXXXXXXXXXXX---------------------------------------------------------------------------------------------------X
psipred               -EEEE--HHHHHHHHHH--EEEEEEE-------EE-----HHH------------------------------EEEEE----------------HHHHHHHHHH-----EEE---HHH----------EEEE----------EEE--HHHHHHHHH---
```

Ginzu Domain Prediction 1

Domain	Span	Source	Reference Parent	Parent Span	Confidence	Annotations
domain 1	1-159	alignment	5dxvA_201	1-149	0.4266	--

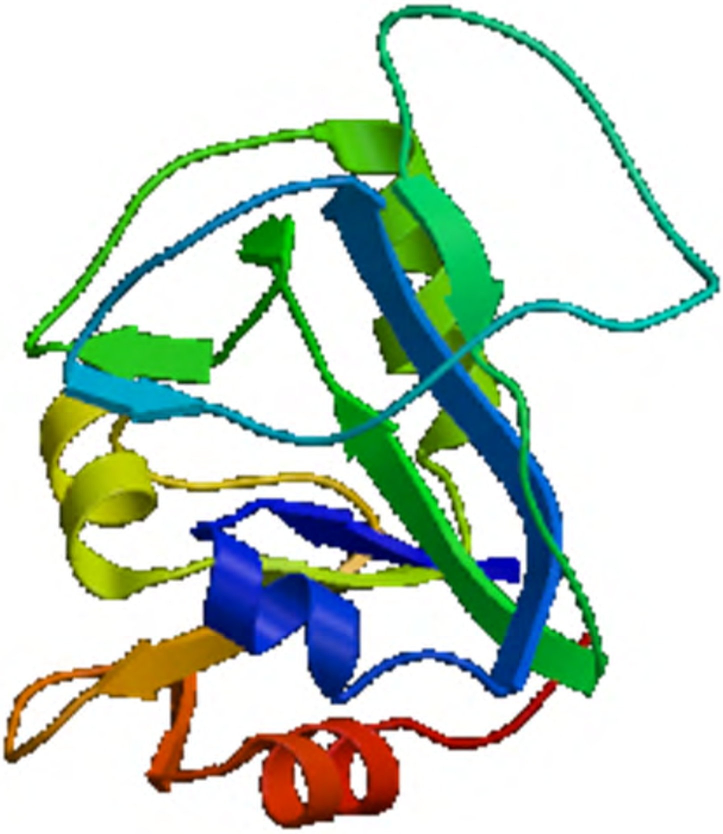

Template: PDB 1rx4A

CP site: Asp127

Target sequence:

```
DYEPDDWESVFSEFHDADAQNSHSYCFEILERRMISLIAALAVDRVIGMENAMPWNLPAD
LAWFKRNTLDKPVIMGRHTWESIGRPLPGRKNIILSSQPGTDDRVTWVKSVDEAIAACGD
VPEIMVIGGGRVYEQFLPKAQKLYLTHIDAEVEGDTHFP
```

Features and Secondary Structure

```
                       1 . 10 . 20 . 30 . 40 . 50 . 60 . 70 . 80 . 90 . 100 . 110 . 120 . 130 . 140 . 150 .
                       HDADAQNSHSYCFEILERRMISLIAALAVDRVIGMENAMPWNLPADLAWFKRNTLDKPVIMGRHTWESIGRPLPGRKNIILSSQPGTDDRVTWVKSVDEAIAACGDVPEIMVIGGGRVYEQFLPKAQKLYLTHIDAEVEGDTHFPDYEPDDWESVFSEF
tmhmm (0)              ---------------------------------------------------------------------------------------------------------------------------------------------------------------
low complexity (0%)    ---------------------------------------------------------------------------------------------------------------------------------------------------------------
coiled-coils (0%)      ---------------------------------------------------------------------------------------------------------------------------------------------------------------
disordered (8%)        XXXXXXXXXXXX---------------------------------------------------------------------------------------------------------------------------------------------------
psipred                -----------HHHH----EEEEEEEE----------------HHHHHHHHHHH---EEEE---EEEEE--------EEEEE--------EEEEE-HHHHHHH-----EEEEE-HHHHHHHHHHH--EEEEEEE-------EE-----HHH-EEEEEE-
```

Ginzu Domain Prediction 1 ▲

Domain	Span	Source	Reference Parent	Parent Span	Confidence	Annotations
domain 1	1-159	alignment	3jvxA_301	1-166	0.8622	OXIDOREDUCTASE

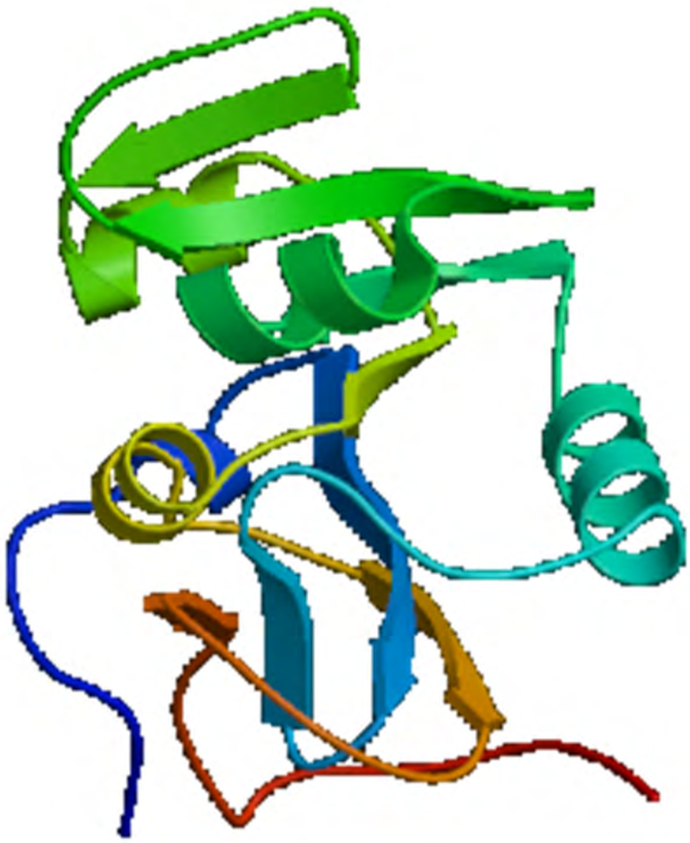

Template: PDB 1rx4A

CP site: Tyr128

Target sequence:

YEPDDWESVFSEFHDADAQNSHSYCFEILERRMISLIAALAVDRVIGMENAMPWNLPADL
AWFKRNTLDKPVIMGRHTWESIGRPLPGRKNIILSSQPGTDDRVTWVKSVDEAIAACGDV
PEIMVIGGGRVYEQFLPKAQKLYLTHIDAEVEGDTHFPD

Features and Secondary Structure

```
                      1   .   10   .   20   .   30   .   40   .   50   .   60   .   70   .   80   .   90   .  100   .  110   .  120   .  130   .  140   .  150   .
                      DADAQNSHSYCFEILERRMISLIAALAVDRVIGMENAMPWNLPADLAWFKRNTLDKPVIMGRHTWESIGRPLPGRKNIILSSQPGTDDRVTWVKSVDEAIAACGDVPEIMVIGGGRVYEQFLPKAQKLYLTHIDAEVEGDTHFPDYEPDDWESVFSEFH
tmhmm (0)             ---------------------------------------------------------------------------------------------------------------------------------------------------------------
low complexity (0%)   ---------------------------------------------------------------------------------------------------------------------------------------------------------------
coiled-coils (0%)     ---------------------------------------------------------------------------------------------------------------------------------------------------------------
disordered (8%)       XXXXX-XX--------------------------------------------------------------------------------------------------------------------------------------------------XXXXX
psipred               ----------HHHH----EEEEEEEE----------------HHHHHHHHHHH---EEEE---EEEEE--------EEEEE--------EEEEE-HHHHHHH-----EEEEE-HHHHHHHHHHH--EEEEEEE-------EE-----HHH-EEEEEEE-
```

Ginzu Domain Prediction 1

Domain	Span	Source	Reference Parent	Parent Span	Confidence	Annotations
domain 1	1-159	alignment	3jvxA_301	1-166	0.8684	OXIDOREDUCTASE

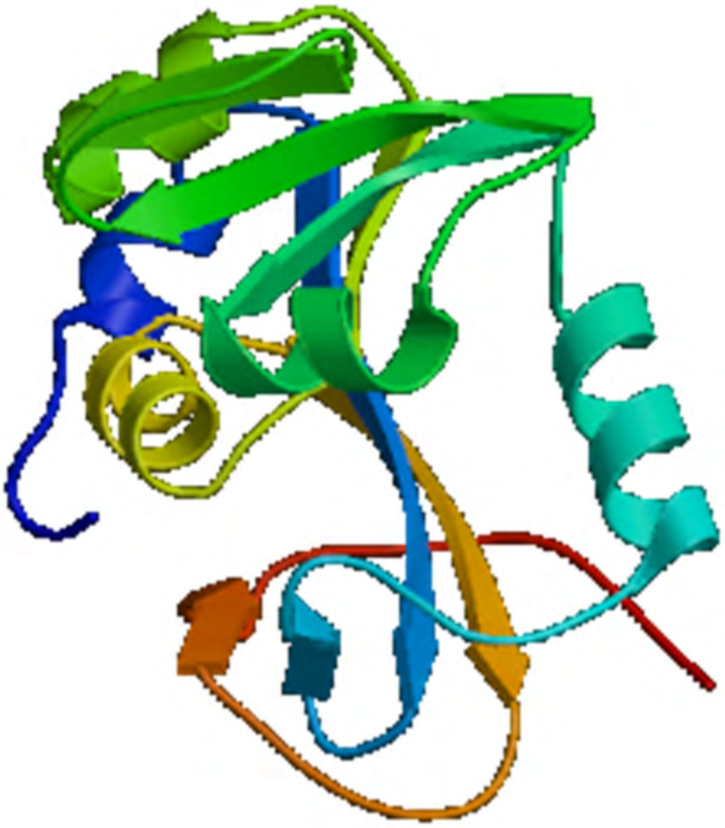

Template: PDB 1rx4A

CP site: Glu129

Target sequence:

EPDDWESVFSEFHDADAQNSHSYCFEILERRMISLIAALAVDRVIGMENAMPWNLPADLA
WFKRNTLDKPVIMGRHTWESIGRPLPGRKNIILSSQPGTDDRVTWVKSVDEAIAACGDVP
EIMVIGGGRVYEQFLPKAQKLYLTHIDAEVEGDTHFPD

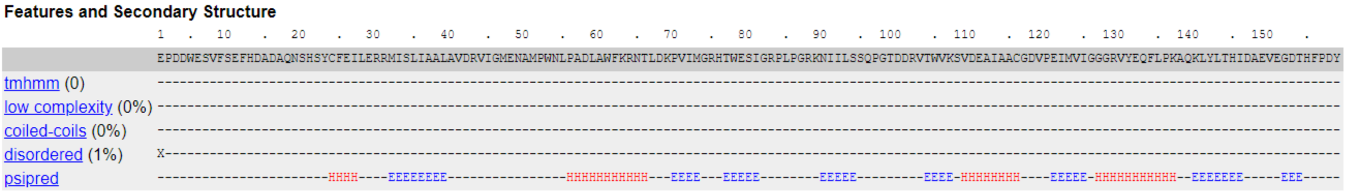

Ginzu Domain Prediction 1

Domain	Span	Source	Reference Parent	Parent Span	Confidence	Annotations
domain 1	1-159	alignment	3jvxA_301	1-166	0.7914	OXIDOREDUCTASE

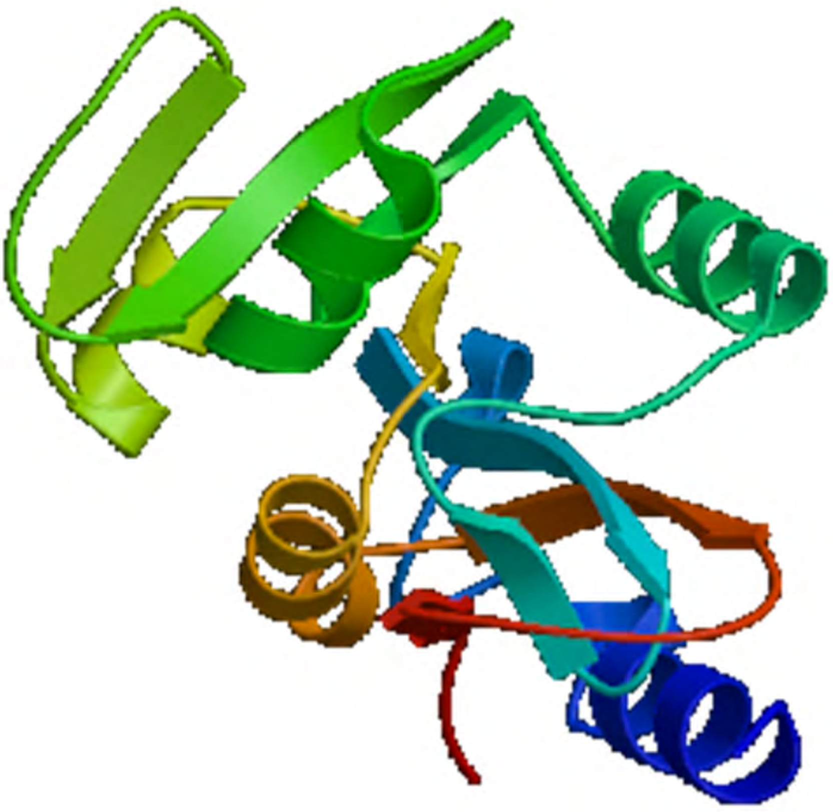

Template: PDB 1rx4A

CP site: Pro130

Target sequence:

PDDWESVFSEFHDADAQNSHSYCFEILERRMISLIAALAVDRVIGMENAMPWNLPADLAW
FKRNTLDKPVIMGRHTWESIGRPLPGRKNIILSSQPGTDDRVTWVKSVDEAIAACGDVPE
IMVIGGGRVYEQFLPKAQKLYLTHIDAEVEGDTHFPDE

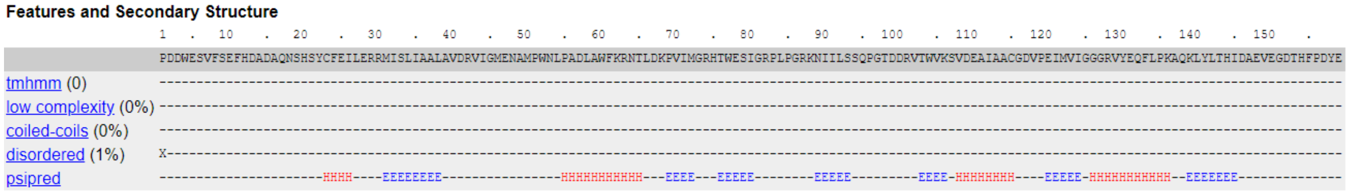

Ginzu Domain Prediction 1

Domain	Span	Source	Reference Parent	Parent Span	Confidence	Annotations
domain 1	1-159	alignment	4m7uA_202	1-170	0.7966	--

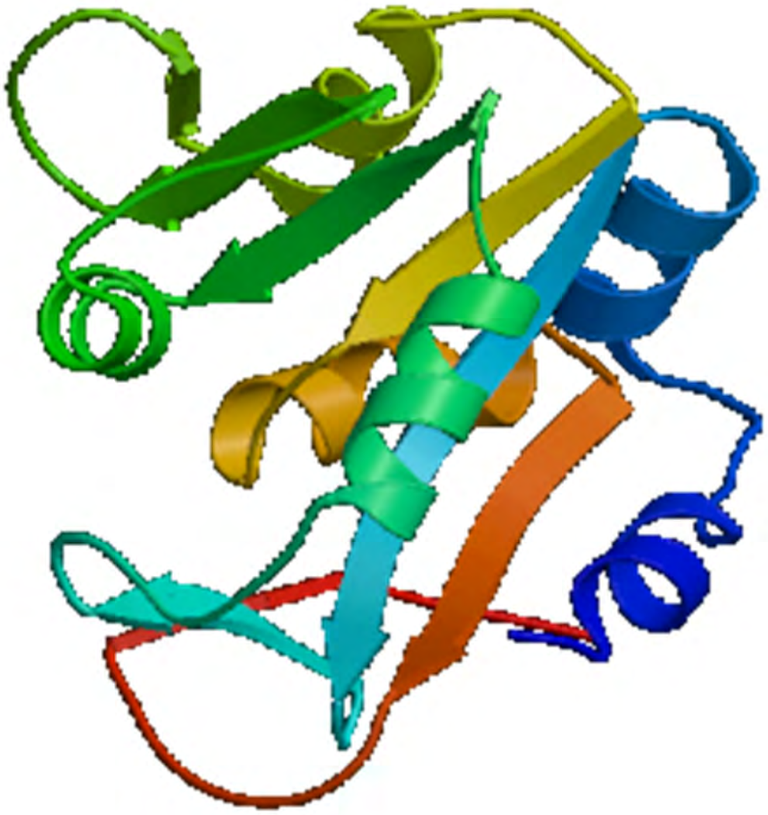

Template: PDB 1rx4A

CP site: Asp131

Target sequence:

DDWESVFSEFHDADAQNSHSYCFEILERRMISLIAALAVDRVIGMENAMPWNLPADLAWF
KRNTLDKPVIMGRHTWESIGRPLPGRKNIILSSQPGTDDRVTWVKSVDEAIAACGDVPEI
MVIGGGRVYEQFLPKAQKLYLTHIDAEVEGDTHFPDEP

Features and Secondary Structure

```
                    1 . 10 . 20 . 30 . 40 . 50 . 60 . 70 . 80 . 90 . 100 . 110 . 120 . 130 . 140 . 150 .
                    DDWESVFSEFHDADAQNSHSYCFEILERRMISLIAALAVDRVIGMENAMPWNLPADLAWFKRNTLDKPVIMGRHTWESIGRPLPGRKNIILSSQPGTDDRVTWVKSVDEAIAACGDVPEIMVIGGGRVYEQFLPKAQKLYLTHIDAEVEGDTHFPDYEP
tmhmm (0)           ---------------------------------------------------------------------------------------------------------------------------------------------------------------
low complexity (0%) ---------------------------------------------------------------------------------------------------------------------------------------------------------------
coiled-coils (0%)   ---------------------------------------------------------------------------------------------------------------------------------------------------------------
disordered (1%)     X--------------------------------------------------------------------------------------------------------------------------------------------------------------
psipred             ---------------------HHHH----EEEEEEEE-----EE---------HHHHHHHHHHH---EEEE---EEEEE--------EEEEE---------EEEE-HHHHHHHH----EEEEE-HHHHHHHHHHH--EEEEEEE------EE-------
```

Ginzu Domain Prediction 1 ▲

Domain	Span	Source	Reference Parent	Parent Span	Confidence	Annotations
domain 1	1-159	alignment	3jvxA_301	1-166	0.8035	OXIDOREDUCTASE

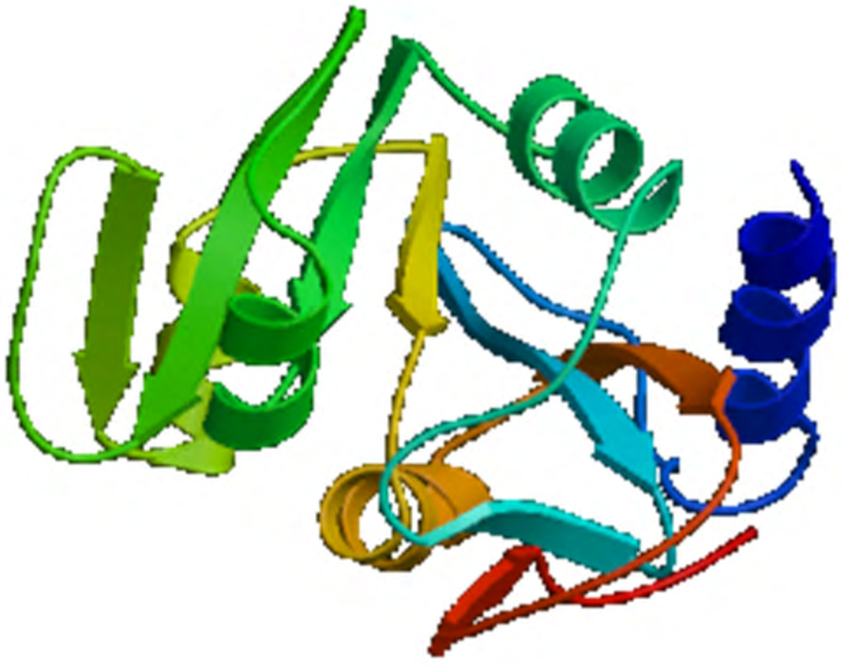

Template: PDB 1rx4A

CP site: Asp132

Target sequence:

DWESVFSEFHDADAQNSHSYCFEILERRMISLIAALAVDRVIGMENAMPWNLPADLAWFK
RNTLDKPVIMGRHTWESIGRPLPGRKNIILSSQPGTDDRVTWVKSVDEAIAACGDVPEIM
VIGGGRVYEQFLPKAQKLYLTHIDAEVEGDTHFPDEPD

Features and Secondary Structure

```
                      1   .   10   .   20   .   30   .   40   .   50   .   60   .   70   .   80   .   90   .  100   .  110   .  120   .  130   .  140   .  150   .
                      DWESVFSEFHDADAQNSHSYCFEILERRMISLIAALAVDRVIGMENAMPWNLPADLAWFKRNTLDKPVIMGRHTWESIGRPLPGRKNIILSSQPGTDDRVTWVKSVDEAIAACGDVPEIMVIGGGRVYEQFLPKAQKLYLTHIDAEVEGDTHFPDYEPD
tmhmm (0)             ---------------------------------------------------------------------------------------------------------------------------------------------------------------
low complexity (0%)   ---------------------------------------------------------------------------------------------------------------------------------------------------------------
coiled-coils (0%)     ---------------------------------------------------------------------------------------------------------------------------------------------------------------
disordered (1%)       X-------------------------------------------------------------------------------------------------------------------------------------------------------------X
psipred               --------------------HHHH----EEEEEEEE-----EE---------HHHHHHHHHHH---EEEE---EEEEE--------EEEEE---------EEEE-HHHHHHHH----EEEEE-HHHHHHHHHHH--EEEEEEE------EE--------
```

Ginzu Domain Prediction 1 ▲

Domain	Span	Source	Reference Parent	Parent Span	Confidence	Annotations
domain 1	1-159	alignment	3jvxA_301	1-166	0.8097	OXIDOREDUCTASE

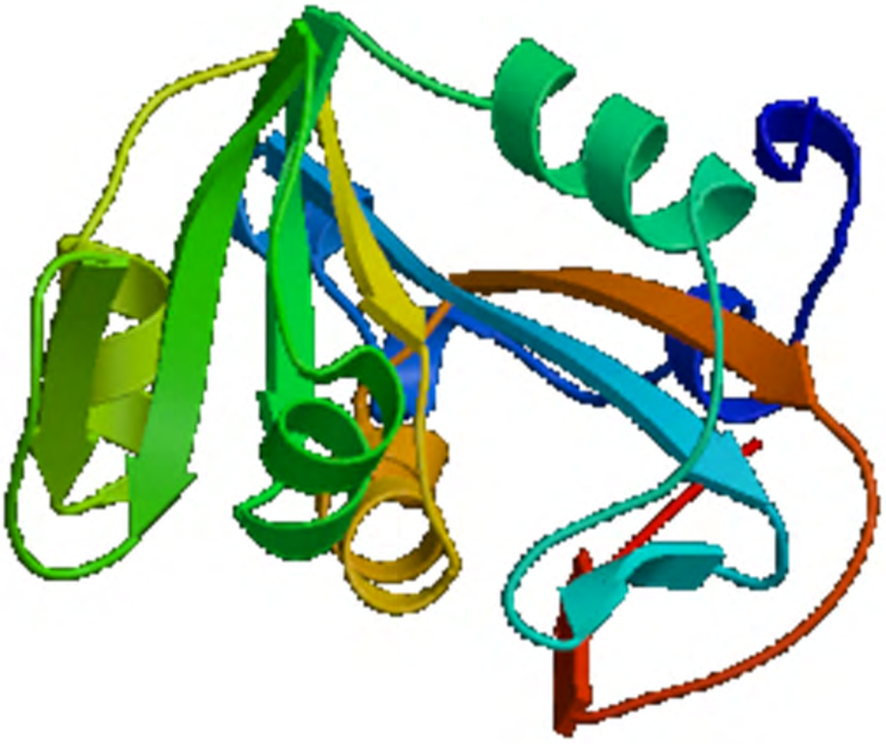

Template: PDB 1rx4A

CP site: Trp133

Target sequence:

WESVFSEFHDADAQNSHSYCFEILERRMISLIAALAVDRVIGMENAMPWNLPADLAWFKR
NTLDKPVIMGRHTWESIGRPLPGRKNIILSSQPGTDDRVTWVKSVDEAIAACGDVPEIMV
IGGGRVYEQFLPKAQKLYLTHIDAEVEGDTHFPDEPDD

Features and Secondary Structure

	1 . 10 . 20 . 30 . 40 . 50 . 60 . 70 . 80 . 90 . 100 . 110 . 120 . 130 . 140 . 150 .
	WESVFSEFHDADAQNSHSYCFEILERRMISLIAALAVDRVIGMENAMPWNLPADLAWFKRNTLDKPVIMGRHTWESIGRPLPGRKNIILSSQPGTDDRVTWVKSVDEAIAACGDVPEIMVIGGGRVYEQFLPKAQKLYLTHIDAEVEGDTHFPDYEPDD
tmhmm (0)	---
low complexity (0%)	---
coiled-coils (0%)	---
disordered (2%)	X--XX
psipred	------HHHH---------HHH-----EEEEEEEE-----EE---------HHHHHHHHHHH---EEEE---EEEEE--------EEEEE---------EEEE-HHHHHHHH----EEEEE-HHHHHHHHHHH--EEEEEEE------EE---------

Ginzu Domain Prediction 1 ▲

Domain	Span	Source	Reference Parent	Parent Span	Confidence	Annotations
domain 1	1-159	alignment	3jvxA_301	1-166	0.8160	OXIDOREDUCTASE

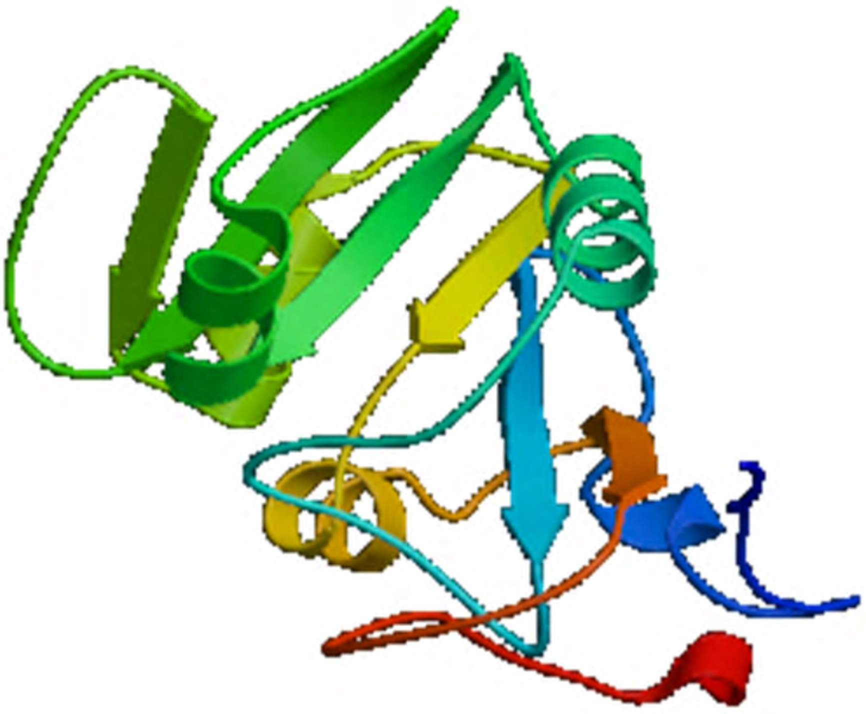

Template: PDB 1rx4A

CP site: Glu134

Target sequence:

ESVFSEFHDADAQNSHSYCFEILERRMISLIAALAVDRVIGMENAMPWNLPADLAWFKRN
TLDKPVIMGRHTWESIGRPLPGRKNIILSSQPGTDDRVTWVKSVDEAIAACGDVPEIMVI
GGGRVYEQFLPKAQKLYLTHIDAEVEGDTHFPDYEPDDW

Features and Secondary Structure

```
                   1    .   10    .   20    .   30    .   40    .   50    .   60    .   70    .   80    .   90    .  100    .  110    .  120    .  130    .  140    .  150    .
                   ESVFSEFHDADAQNSHSYCFEILERRMISLIAALAVDRVIGMENAMPWNLPADLAWFKRNTLDKPVIMGRHTWESIGRPLPGRKNIILSSQPGTDDRVTWVKSVDEAIAACGDVPEIMVIGGGRVYEQFLPKAQKLYLTHIDAEVEGDTHFPDYEPDDW
tmhmm (0)          ---------------------------------------------------------------------------------------------------------------------------------------------------------------
low complexity (0%) ---------------------------------------------------------------------------------------------------------------------------------------------------------------
coiled-coils (0%)  ---------------------------------------------------------------------------------------------------------------------------------------------------------------
disordered (2%)    XX------------------------------------------------------------------------------------------------------------------------------------------------------------X
psipred            -----HHHH---------HHHH----EEEEEEEE-----EE---------HHHHHHHHHHH---EEEE---EEEEE--------EEEEE--------EEEEE-HHHHHHH-----EEEEE-HHHHHHHHHHH--EEEEEEE------EEE-----HHH-
```

Ginzu Domain Prediction 1 ▲

Domain	Span	Source	Reference Parent	Parent Span	Confidence	Annotations
domain 1	1-159	alignment	1j3jA_202	1-221	0.8229	OXIDOREDUCTASE, TRANSFERASE

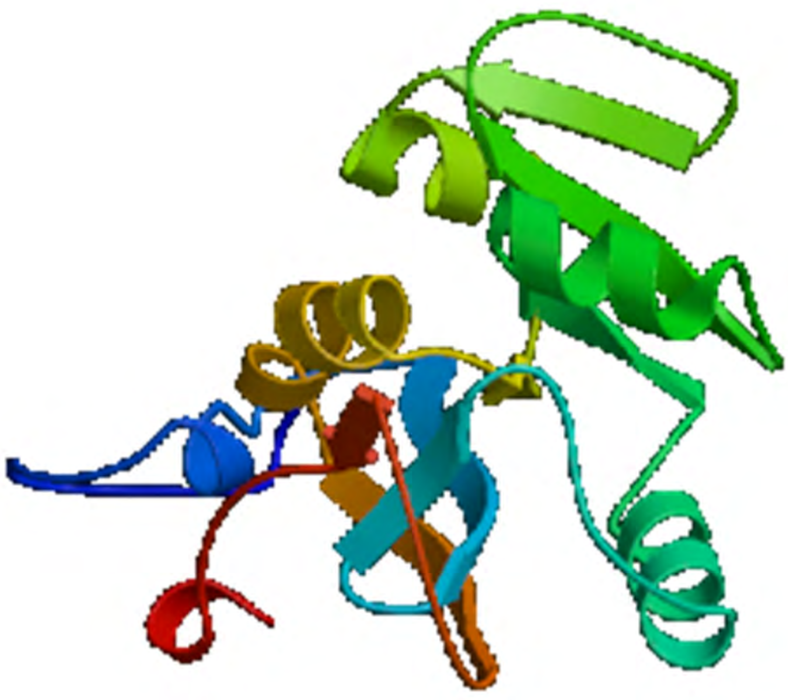

Template: PDB 1rx4A

CP site: Ser135

Target sequence:

```
SVFSEFHDADAQNSHSYCFEILERRMISLIAALAVDRVIGMENAMPWNLPADLAWFKRNT
LDKPVIMGRHTWESIGRPLPGRKNIILSSQPGTDDRVTWVKSVDEAIAACGDVPEIMVIG
GGRVYEQFLPKAQKLYLTHIDAEVEGDTHFPDYEPDDWE
```

Features and Secondary Structure

```
                    1 . 10 . 20 . 30 . 40 . 50 . 60 . 70 . 80 . 90 . 100 . 110 . 120 . 130 . 140 . 150 .
                    SVFSEFHDADAQNSHSYCFEILERRMISLIAALAVDRVIGMENAMPWNLPADLAWFKRNTLDKPVIMGRHTWESIGRPLPGRKNIILSSQPGTDDRVTWVKSVDEAIAACGDVPEIMVIGGGRVYEQFLPKAQKLYLTHIDAEVEGDTHFPDYEPDDWE
tmhmm (0)           ---------------------------------------------------------------------------------------------------------------------------------------------------------------
low complexity (0%) ---------------------------------------------------------------------------------------------------------------------------------------------------------------
coiled-coils (0%)   ---------------------------------------------------------------------------------------------------------------------------------------------------------------
disordered (2%)     X------------------------------------------------------------------------------------------------------------------------------------------------------------XX
psipred             ----HHHH---------HHHH----EEEEEEEE-----EE---------HHHHHHHHHHH---EEEE---EEEEE--------EEEEE--------EEEEE-HHHHHHH-----EEEEE-HHHHHHHHHHH--EEEEEEE------EEE-----HHH--
```

Ginzu Domain Prediction 1 ▲

Domain	Span	Source	Reference Parent	Parent Span	Confidence	Annotations
domain 1	1-159	alignment	4m7uA_202	1-170	0.8291	--

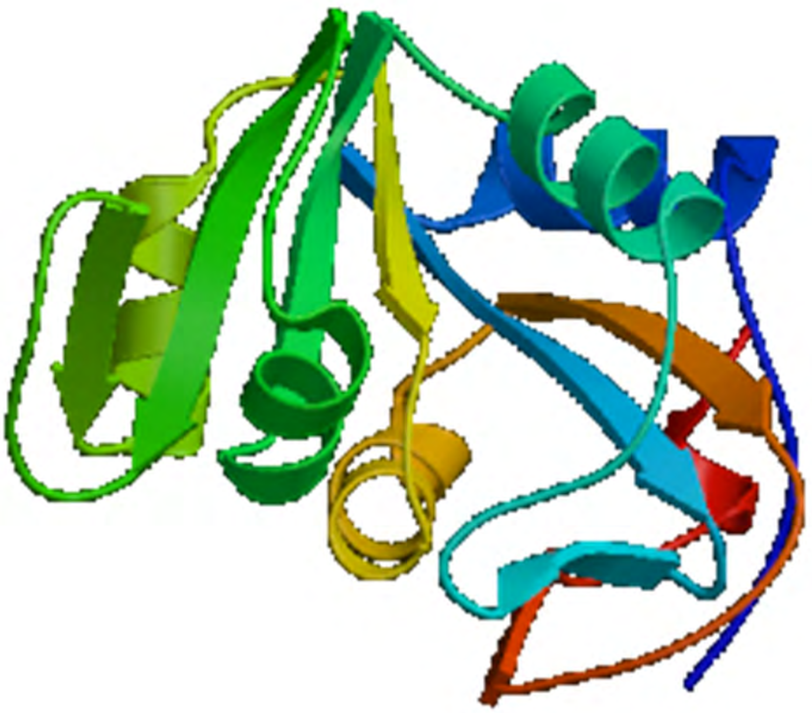

Template: PDB 1rx4A

CP site: Glu139

Target sequence:

EFHDADAQNSHSYCFEILERRMISLIAALAVDRVIGMENAMPWNLPADLAWFKRNTLDKP
VIMGRHTWESIGRPLPGRKNIILSSQPGTDDRVTWVKSVDEAIAACGDVPEIMVIGGGRV
YEQFLPKAQKLYLTHIDAEVEGDTHFPDYEPDDWESVFS

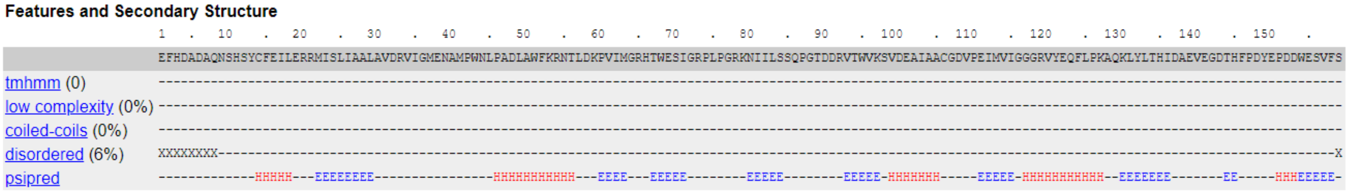

Ginzu Domain Prediction 1 ▲

Domain	Span	Source	Reference Parent	Parent Span	Confidence	Annotations
domain 1	1-159	alignment	3jvxA_301	1-166	0.8538	OXIDOREDUCTASE

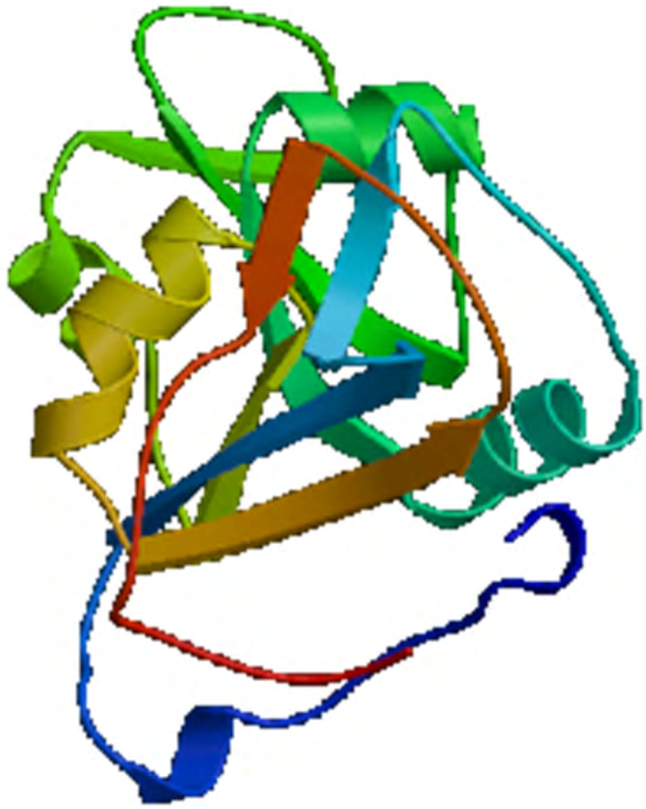

Template: PDB 1rx4A

CP site: Phe140

Target sequence:

```
FHDADAQNSHSYCFEILERRMISLIAALAVDRVIGMENAMPWNLPADLAWFKRNTLDKPV
IMGRHTWESIGRPLPGRKNIILSSQPGTDDRVTWVKSVDEAIAACGDVPEIMVIGGGRVY
EQFLPKAQKLYLTHIDAEVEGDTHFPDYEPDDWESVFSE
```

Features and Secondary Structure

```
                    1 . 10 . 20 . 30 . 40 . 50 . 60 . 70 . 80 . 90 . 100 . 110 . 120 . 130 . 140 . 150 .
                    FHDADAQNSHSYCFEILERRMISLIAALAVDRVIGMENAMPWNLPADLAWFKRNTLDKPVIMGRHTWESIGRPLPGRKNIILSSQPGTDDRVTWVKSVDEAIAACGDVPEIMVIGGGRVYEQFLPKAQKLYLTHIDAEVEGDTHFPDYEPDDWESVFSE
tmhmm (0)           ---------------------------------------------------------------------------------------------------------------------------------------------------------------
low complexity (0%) ---------------------------------------------------------------------------------------------------------------------------------------------------------------
coiled-coils (0%)   ---------------------------------------------------------------------------------------------------------------------------------------------------------------
disordered (9%)     XXXXXXXXXXXX------------------------------------------------------------------------------------------------------------------------------------------------XXX
psipred             ------------HHHHH---EEEEEEEE----------------HHHHHHHHHHH---EEEE---EEEEE--------EEEEE--------EEEEE-HHHHHHH-----EEEEE-HHHHHHHHHHH--EEEEEEE-------EE-----HHH-EEEEE-
```

Ginzu Domain Prediction 1 ▲

Domain	Span	Source	Reference Parent	Parent Span	Confidence	Annotations
domain 1	1-159	alignment	4m7uA_202	1-170	0.8601	--

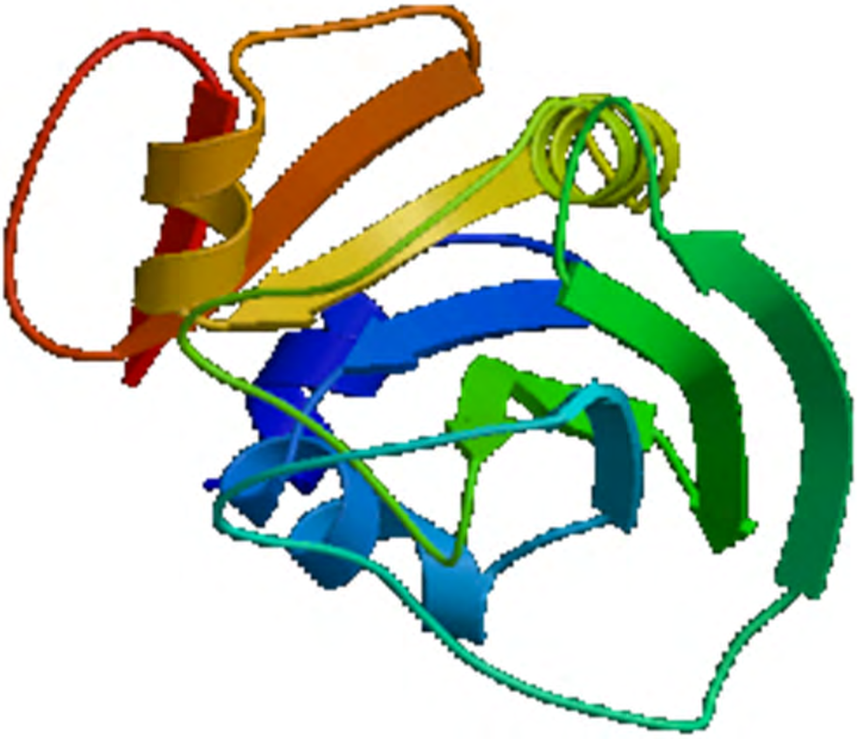

Template: PDB 1rx4A

CP site: His141

Target sequence:

HDADAQNSHSYCFEILERRMISLIAALAVDRVIGMENAMPWNLPADLAWFKRNTLDKPVI
MGRHTWESIGRPLPGRKNIILSSQPGTDDRVTWVKSVDEAIAACGDVPEIMVIGGGRVYE
QFLPKAQKLYLTHIDAEVEGDTHFPDYEPDDWESVFSEF

Features and Secondary Structure

	1 . 10 . 20 . 30 . 40 . 50 . 60 . 70 . 80 . 90 . 100 . 110 . 120 . 130 . 140 . 150 .
	HDADAQNSHSYCFEILERRMISLIAALAVDRVIGMENAMPWNLPADLAWFKRNTLDKPVIMGRHTWESIGRPLPGRKNIILSSQPGTDDRVTWVKSVDEAIAACGDVPEIMVIGGGRVYEQFLPKAQKLYLTHIDAEVEGDTHFPDYEPDDWESVFSEF
tmhmm (0)	---
low complexity (0%)	---
coiled-coils (0%)	---
disordered (8%)	XXXXXXXXXXXX---
psipred	-----------HHHH----EEEEEEEE----------------HHHHHHHHHHH---EEEE---EEEEE--------EEEEE--------EEEEE-HHHHHHH-----EEEEE-HHHHHHHHHHH--EEEEEEE-------EE-----HHH-EEEEEE-

Ginzu Domain Prediction 1 ▲

Domain	Span	Source	Reference Parent	Parent Span	Confidence	Annotations
domain 1	1-159	alignment	3jvxA_301	1-166	0.8622	OXIDOREDUCTASE

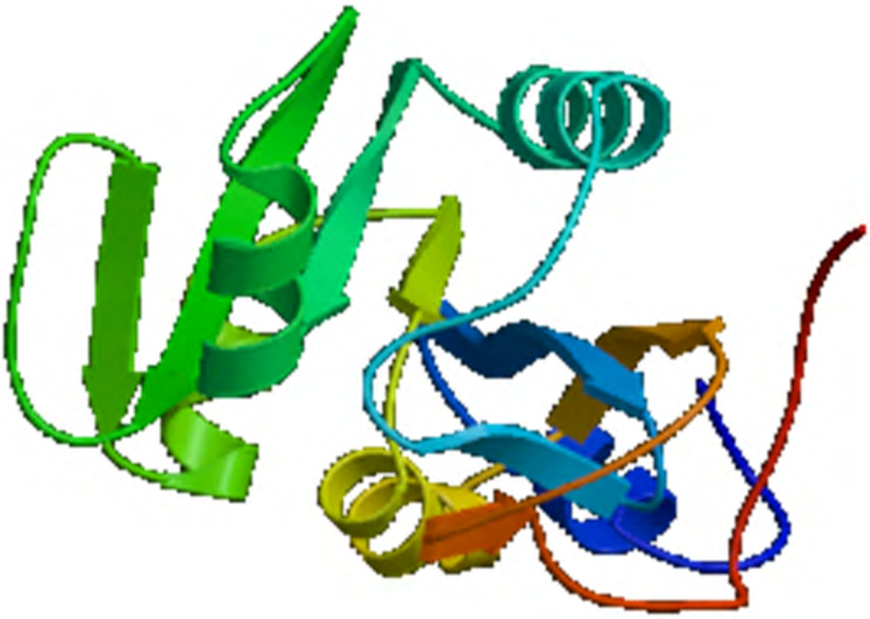

Template: PDB 1rx4A

CP site: Asp142

Target sequence:

```
DADAQNSHSYCFEILERRMISLIAALAVDRVIGMENAMPWNLPADLAWFKRNTLDKPVIM
GRHTWESIGRPLPGRKNIILSSQPGTDDRVTWVKSVDEAIAACGDVPEIMVIGGGRVYEQ
FLPKAQKLYLTHIDAEVEGDTHFPDYEPDDWESVFSEFH
```

Features and Secondary Structure

```
                       1 . 10 . 20 . 30 . 40 . 50 . 60 . 70 . 80 . 90 . 100 . 110 . 120 . 130 . 140 . 150 .
                       DADAQNSHSYCFEILERRMISLIAALAVDRVIGMENAMPWNLPADLAWFKRNTLDKPVIMGRHTWESIGRPLPGRKNIILSSQPGTDDRVTWVKSVDEAIAACGDVPEIMVIGGGRVYEQFLPKAQKLYLTHIDAEVEGDTHFPDYEPDDWESVFSEFH
tmhmm (0)              ---------------------------------------------------------------------------------------------------------------------------------------------------------------
low complexity (0%)    ---------------------------------------------------------------------------------------------------------------------------------------------------------------
coiled-coils (0%)      ---------------------------------------------------------------------------------------------------------------------------------------------------------------
disordered (8%)        XXXXX-XX--------------------------------------------------------------------------------------------------------------------------------------------------XXXXX
psipred                ----------HHHH----EEEEEEEE----------------HHHHHHHHHHH---EEEE---EEEEE--------EEEEE--------EEEEE-HHHHHHH-----EEEEE-HHHHHHHHHHH--EEEEEEE-------EE-----HHH-EEEEEEE-
```

Ginzu Domain Prediction 1 ▲

Domain	Span	Source	Reference Parent	Parent Span	Confidence	Annotations
domain 1	1-159	alignment	3jvxA_301	1-166	0.8684	OXIDOREDUCTASE

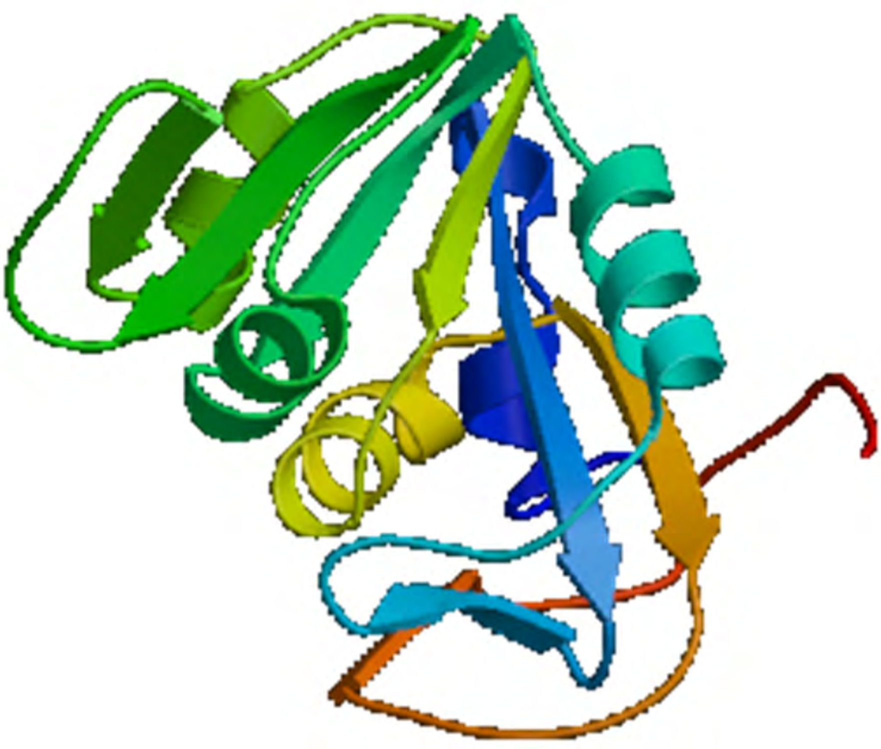

Template: PDB 1rx4A

CP site: Ala143

Target sequence:

ADAQNSHSYCFEILERRMISLIAALAVDRVIGMENAMPWNLPADLAWFKRNTLDKPVIMG
RHTWESIGRPLPGRKNIILSSQPGTDDRVTWVKSVDEAIAACGDVPEIMVIGGGRVYEQF
LPKAQKLYLTHIDAEVEGDTHFPDYEPDDWESVFSEFHD

Features and Secondary Structure

	1 . 10 . 20 . 30 . 40 . 50 . 60 . 70 . 80 . 90 . 100 . 110 . 120 . 130 . 140 . 150 .
	ADAQNSHSYCFEILERRMISLIAALAVDRVIGMENAMPWNLPADLAWFKRNTLDKPVIMGRHTWESIGRPLPGRKNIILSSQPGTDDRVTWVKSVDEAIAACGDVPEIMVIGGGRVYEQFLPKAQKLYLTHIDAEVEGDTHFPDYEPDDWESVFSEFHD
tmhmm (0)	---
low complexity (0%)	---
coiled-coils (0%)	---
disordered (8%)	XXXXXX-X---XXXXXX
psipred	---------HHHH----EEEEEEEE----------------HHHHHHHHHHH---EEEE---EEEEE--------EEEEE--------EEEEE-HHHHHHH-----EEEEE-HHHHHHHHHHH--EEEEEEE-------EE-----HHH-EEEEEEE--

Ginzu Domain Prediction 1 ▲

Domain	Span	Source	Reference Parent	Parent Span	Confidence	Annotations
domain 1	1-159	alignment	3jvxA_301	1-166	0.8695	OXIDOREDUCTASE

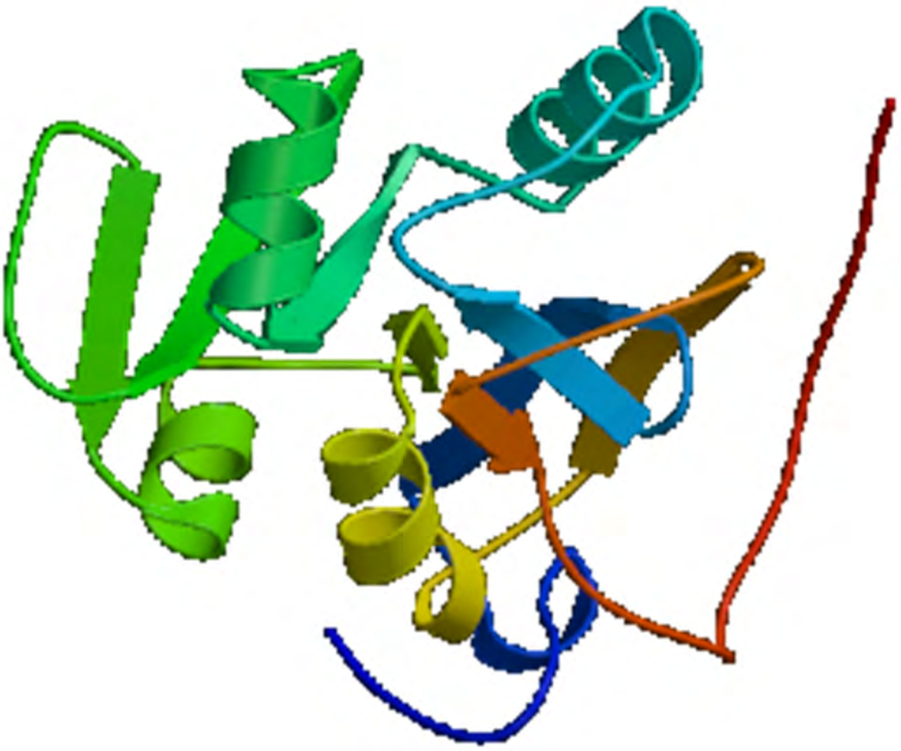

Template: PDB 1rx4A

CP site: Asp144

Target sequence:

DAQNSHSYCFEILERRMISLIAALAVDRVIGMENAMPWNLPADLAWFKRNTLDKPVIMGR
HTWESIGRPLPGRKNIILSSQPGTDDRVTWVKSVDEAIAACGDVPEIMVIGGGRVYEQFL
PKAQKLYLTHIDAEVEGDTHFPDYEPDDWESVFSEFHDA

Features and Secondary Structure

```
                     1   .  10   .  20   .  30   .  40   .  50   .  60   .  70   .  80   .  90   . 100   . 110   . 120   . 130   . 140   . 150   .
                     DAQNSHSYCFEILERRMISLIAALAVDRVIGMENAMPWNLPADLAWFKRNTLDKPVIMGRHTWESIGRPLPGRKNIILSSQPGTDDRVTWVKSVDEAIAACGDVPEIMVIGGGRVYEQFLPKAQKLYLTHIDAEVEGDTHFPDYEPDDWESVFSEFHDA
tmhmm (0)            ---------------------------------------------------------------------------------------------------------------------------------------------------------------
low complexity (0%)  ---------------------------------------------------------------------------------------------------------------------------------------------------------------
coiled-coils (0%)    ---------------------------------------------------------------------------------------------------------------------------------------------------------------
disordered (9%)      XXXXXX-XX------------------------------------------------------------------------------------------------------------------------------------------------XXXXXX
psipred              -------HHHHHH---EEEEEEEE----------------HHHHHHHHHHH---EEEE---EEEEE--------EEEEE--------EEEEE-HHHHHHH-----EEEEE-HHHHHHHHHHH--EEEEEEE-------EE-----HHH-EEEEEEE---
```

Ginzu Domain Prediction 1 ▲

Domain	Span	Source	Reference Parent	Parent Span	Confidence	Annotations
domain 1	1-159	alignment	3jvxA_301	1-166	0.8758	OXIDOREDUCTASE

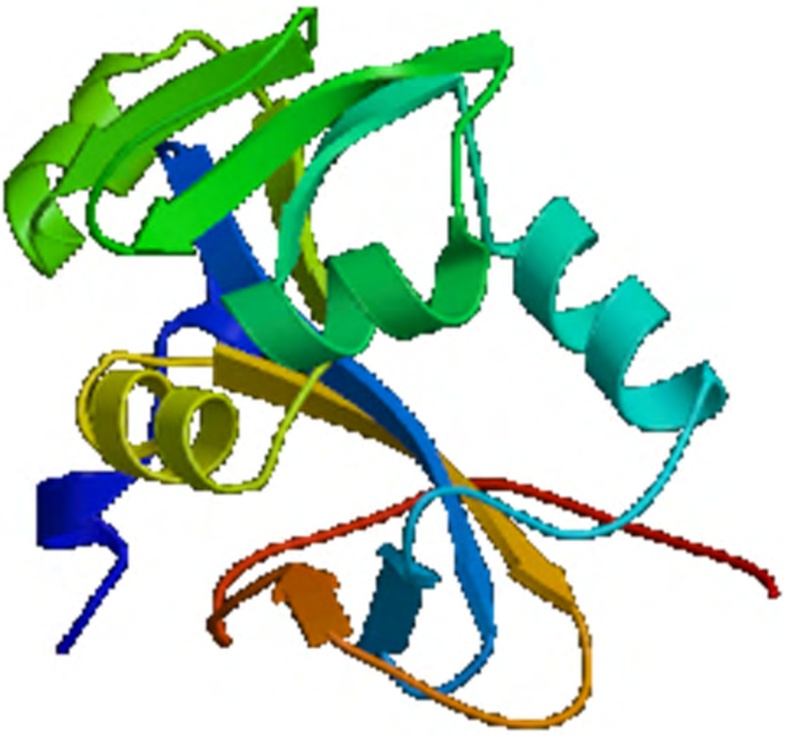

Template: PDB 1rx4A

CP site: Ala145

Target sequence:

AQNSHSYCFEILERRMISLIAALAVDRVIGMENAMPWNLPADLAWFKRNTLDKPVIMGRH
TWESIGRPLPGRKNIILSSQPGTDDRVTWVKSVDEAIAACGDVPEIMVIGGGRVYEQFLP
KAQKLYLTHIDAEVEGDTHFPDYEPDDWESVFSEFHDAD

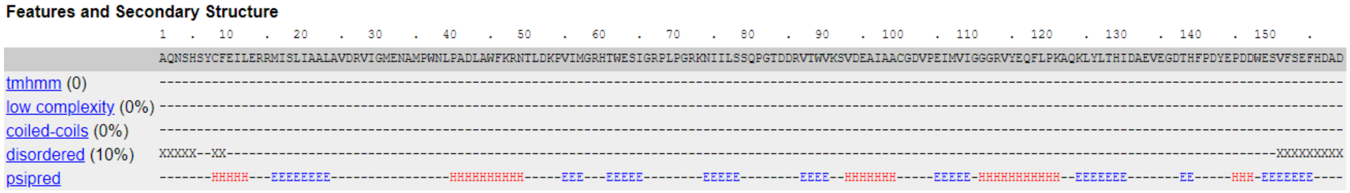

Ginzu Domain Prediction 1

Domain	Span	Source	Reference Parent	Parent Span	Confidence	Annotations
domain 1	1-159	alignment	3jvxA_301	1-166	0.8810	OXIDOREDUCTASE

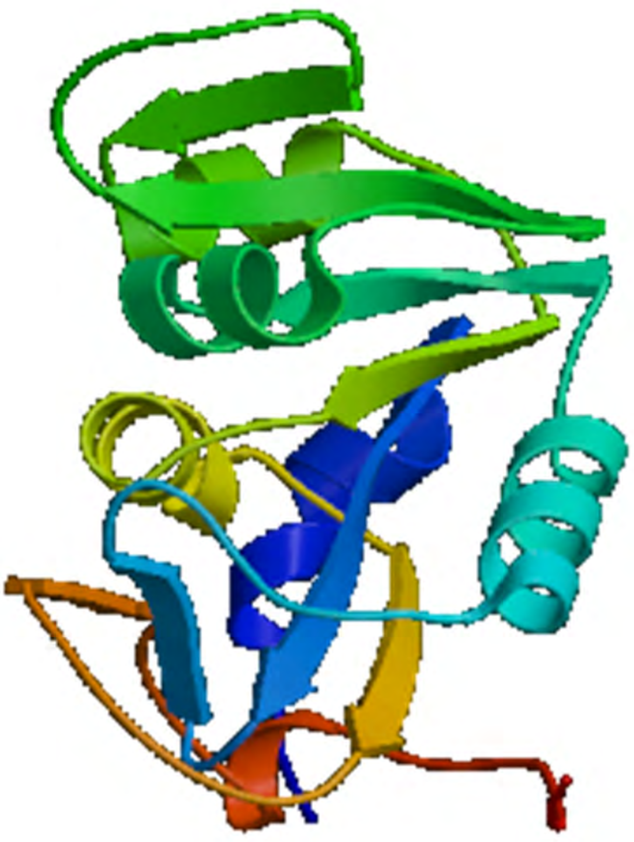

Template: PDB 1rx4A

CP site: Gln146

Target sequence:

```
QNSHSYCFEILERRMISLIAALAVDRVIGMENAMPWNLPADLAWFKRNTLDKPVIMGRHT
WESIGRPLPGRKNIILSSQPGTDDRVTWVKSVDEAIAACGDVPEIMVIGGGRVYEQFLPK
AQKLYLTHIDAEVEGDTHFPDYEPDDWESVFSEFHDADA
```

Features and Secondary Structure

	1 . 10 . 20 . 30 . 40 . 50 . 60 . 70 . 80 . 90 . 100 . 110 . 120 . 130 . 140 . 150 .
	QNSHSYCFEILERRMISLIAALAVDRVIGMENAMPWNLPADLAWFKRNTLDKPVIMGRHTWESIGRPLPGRKNIILSSQPGTDDRVTWVKSVDEAIAACGDVPEIMVIGGGRVYEQFLPKAQKLYLTHIDAEVEGDTHFPDYEPDDWESVFSEFHDADA
tmhmm (0)	---
low complexity (0%)	---
coiled-coils (0%)	---
disordered (9%)	XXXX---X--XXXXXXXXX
psipred	-----HHHHH----EEEEEEEE----------------HHHHHHHHHH----EEE---EEEE----------EEEEE--------EEEEE-HHHHHHH-----EEEEE-HHHHHHHHHHH--EEEEEEE-------EE-----HHH-EEEEEEE-----

Ginzu Domain Prediction 1

Domain	Span	Source	Reference Parent	Parent Span	Confidence	Annotations
domain 1	1-159	alignment	3jvxA_301	1-166	0.8758	OXIDOREDUCTASE

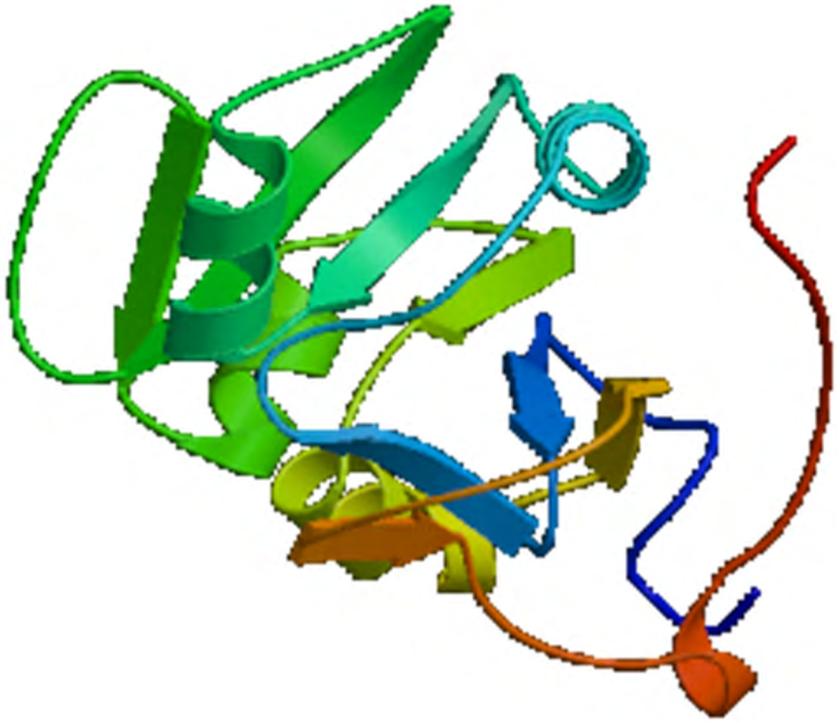

Template: PDB 1rx4A

CP site: Asn147

Target sequence:

NSHSYCFEILERRMISLIAALAVDRVIGMENAMPWNLPADLAWFKRNTLDKPVIMGRHTW
ESIGRPLPGRKNIILSSQPGTDDRVTWVKSVDEAIAACGDVPEIMVIGGGRVYEQFLPKA
QKLYLTHIDAEVEGDTHFPDYEPDDWESVFSEFHDADAQ

Features and Secondary Structure

	1 . 10 . 20 . 30 . 40 . 50 . 60 . 70 . 80 . 90 . 100 . 110 . 120 . 130 . 140 . 150 .
	NSHSYCFEILERRMISLIAALAVDRVIGMENAMPWNLPADLAWFKRNTLDKPVIMGRHTWESIGRPLPGRKNIILSSQPGTDDRVTWVKSVDEAIAACGDVPEIMVIGGGRVYEQFLPKAQKLYLTHIDAEVEGDTHFPDYEPDDWESVFSEFHDADAQ
tmhmm (0)	---
low complexity (0%)	---
coiled-coils (0%)	---
disordered (9%)	XXX---XXXXXXXXXXX
psipred	----HHHHH----EEEEEEEE----------------HHHHHHHHHHH---EEEE---EEEE---------EEEEE--------EEEEE-HHHHHHH-----EEEEE-HHHHHHHHHHH--EEEEEEE--------------HHH-EEEEEEE------

Ginzu Domain Prediction 1 ▲

Domain	Span	Source	Reference Parent	Parent Span	Confidence	Annotations
domain 1	1-159	alignment	3jvxA_301	1-166	0.8779	OXIDOREDUCTASE

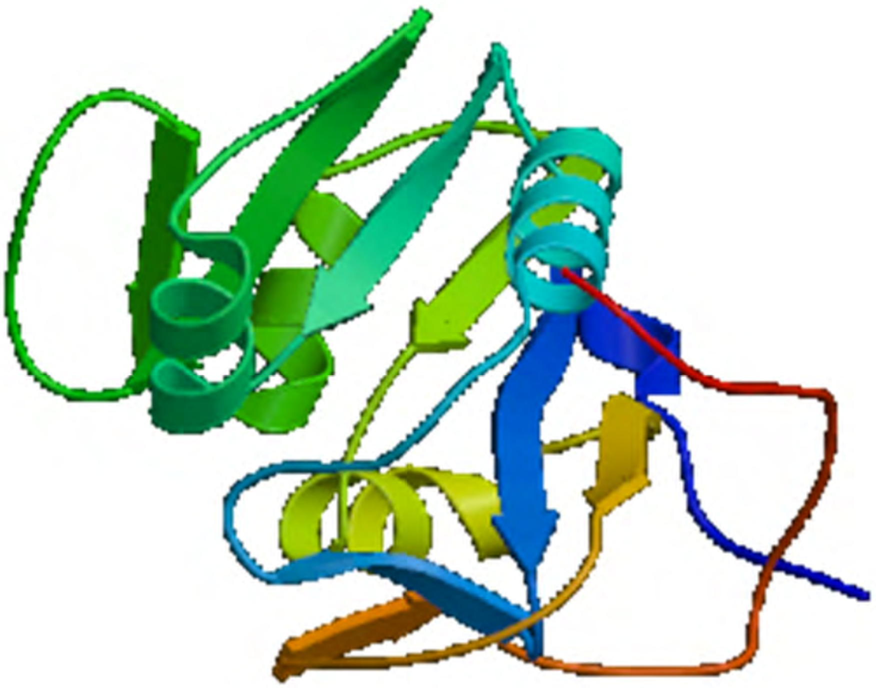

Supplement: Supplementary file 1 — Additional file 1: Data S1. Modeling results of SWISS-MODEL, RaptorX, and Robetta for viable circular permutants of the DHFR. [file 12859_2021_4403_MOESM1_ESM.pdf]
