# Supplementary material for: CirPred, the first structure modeling and linker design system for circularly permuted proteins
Source: BMC Bioinformatics. 2021 Oct 12;22(Suppl 10):494. doi: 10.1186/s12859-021-04403-1 (PMC8513176; doi:10.1186/s12859-021-04403-1)
Supplement: Supplementary file 2 — Additional file 2: Fig. S1. Co-linear modeling quality of the CirPred and several state-of-the-art modeling methods. [file 12859_2021_4403_MOESM2_ESM.pdf]

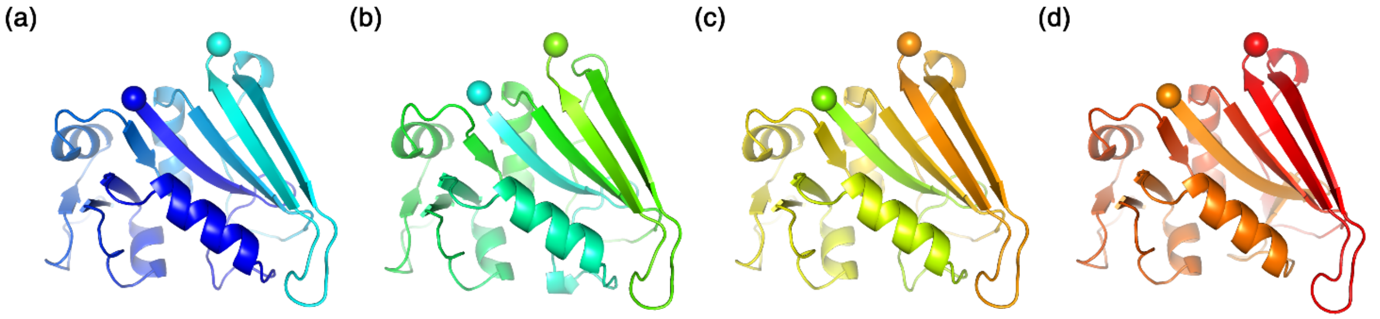

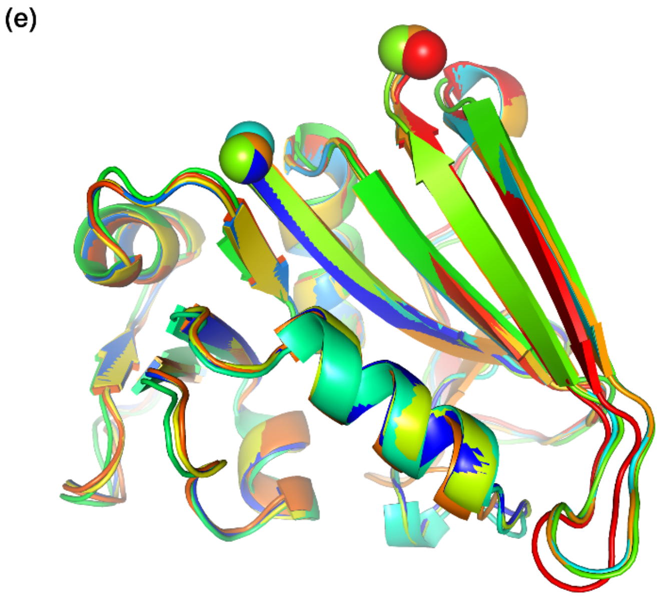

(f)

RMSD (Å)	Native	CirPred	SWISS-MODEL	RaptorX
Native	–	0.732	0.149	1.362
CirPred	0.732	–	0.723	1.545
SWISS-MODEL	0.149	0.723	–	1.378
RaptorX	1.362	1.545	1.378	–
Sum (Å)	2.243	3.000	2.250	4.285
Average (Å)	0.748	1.000	0.750	1.428

Fig. S1. Co-linear modeling quality of the CirPred and several state-of-the-art modeling methods. Although the CirPred is designed for circularly-permuted proteins, it can be applied to traditional co-linear structure modeling, simply by setting the CP site of the query template protein as residue 1. Here the dihydrofolate reductase (DHFR) was used as the template to construct models of the DHFR itself by CirPred and two widely-used comparative structure modeling methods, SWISS-MODEL [18] and RaptorX [19]. **(a)** Crystal structure of the native DHFR protein (PDB 1rx4). **(b)** Model constructed by CirPred. **(c)** Model constructed by SWISS-MODEL. **(d)** Model constructed by RaptorX. **(e)** Superimposition of all the above structures. **(f)** Root-mean-square distances (RMSD) between all pairs of the above structures. In this example, on average, the co-linear model made by CirPred showed only 1 Å difference from the native structure or models made by other methods.
