# Supplementary material for: CirPred, the first structure modeling and linker design system for circularly permuted proteins
Source: BMC Bioinformatics. 2021 Oct 12;22(Suppl 10):494. doi: 10.1186/s12859-021-04403-1 (PMC8513176; doi:10.1186/s12859-021-04403-1)
Supplement: Supplementary file 4 — Additional file 4: Fig. S2. Models constructed by CirPred for circular permutants with high identity, low identity, or large sizes. [file 12859_2021_4403_MOESM4_ESM.pdf]

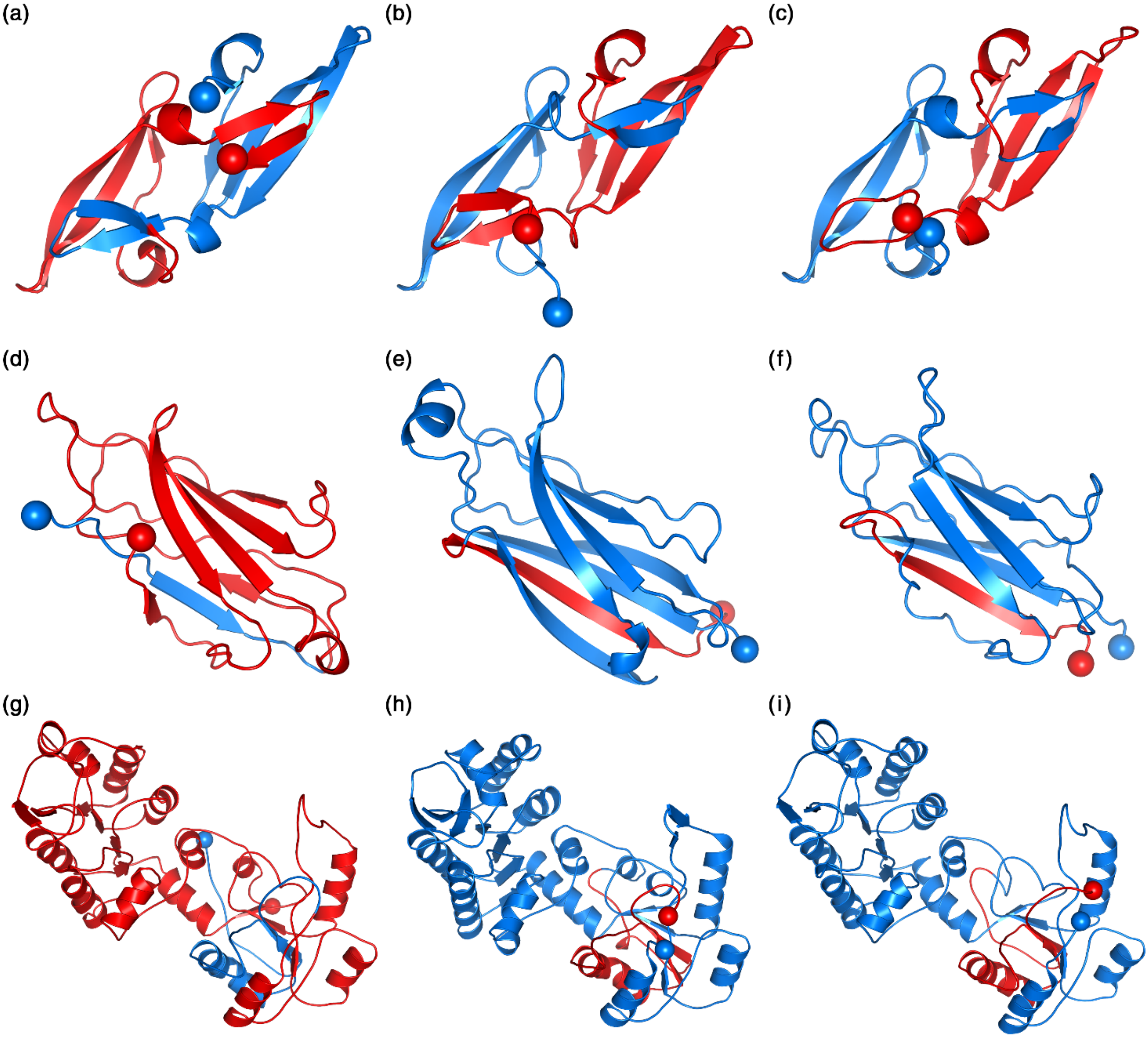

Fig. S2. Models constructed by CirPred for circular permutants with high identity, low identity, or large sizes. The proposed method is robust even when the sequence identity between the target and template proteins is low or when those proteins are large. In these three sets of examples, the CPM models constructed by CirPred are highly similar to the actual structures of the CPMs. All the alignment ratio and RMSD values between the actual structures and the CirPred models were >95% and <3.2 Å, respectively. Note that images shown here are not at the same scale; the proteins in **g**–**i** are four times larger than those in **a**–**c**. **(a)** Solution structure of the cyanovirin-N, a potent HIV-inactivating protein from *Nostoc ellipsosporum* (101 residues; PDB 2ezm). **(b)** Solution structure of the engineered CPM50 of **a** (PDB 1n02). The sequence identity between **a** and **b** is 90.1%. **(c)** The model of **b** constructed by CirPred using **a** as the template. **(d)** Crystal structure of the RIM2 C_2A-domain from *Rattus norvegicus* (129 residues; PDB 2bwq). **(e)** Crystal structure of the human Ca^{2+}-phospholipid binding domain from cytosolic phospholipase A2 (126 residues; PDB 1rlw). The sequence identity between **d** and **e** is 19.5%. **(f)** The model of **e** constructed by CirPred using **d** as the template. **(g)** Crystal structure of the yeast phosphoglycerate kinase (416 residues; PDB 3pgk). **(h)** Crystal structure of the engineered CPM72 of **g** (PDB 1fw8). **(i)** The model of **h** constructed by CirPred using **g** as the template.
