# Supplementary material for: CirPred, the first structure modeling and linker design system for circularly permuted proteins
Source: BMC Bioinformatics. 2021 Oct 12;22(Suppl 10):494. doi: 10.1186/s12859-021-04403-1 (PMC8513176; doi:10.1186/s12859-021-04403-1)
Supplement: Supplementary file 5 — Additional file 5: Tables S1, S2. Performance of CirPred for proteins of various sizes and various CP site positions. [file 12859_2021_4403_MOESM5_ESM.pdf]

Table S1. Performance of CirPred for proteins of various sizes.

Target size (residues)	Alignment ratio (%)	RMSD (Å)	Number of CP pairs
≥ 300	83.8	3.794	56
200 – 300	91.6	2.720	649
100 – 200	80.0	3.111	524
< 100	90.7	2.336	339

Table S2. Performance of CirPred for various CP site positions.

Relative distance from the termini (%)	Alignment ratio (%)	RMSD (Å)	Number of CP pairs
80 – 100	89.1	2.596	861
60 – 80	84.0	2.952	251
40 – 60	87.2	2.935	210
20 – 40	89.7	2.938	160
< 20	74.0	3.918	86

Here we describe the position of a CP site by its relative distance from the native termini using this equation,

$$d_p = \left(1 - \frac{\left|\frac{size}{2} - p\right|}{\frac{size}{2}}\right) \times 100\%$$

where d_p is the relative distance of CP site p, and *size* means the protein size. According to this, the d_p value of a CP site located at the N- or C-terminus is 0%, whereas one located right at the center of a sequence has d_p = 100%.
