# Supplementary material for: CirPred, the first structure modeling and linker design system for circularly permuted proteins
Source: BMC Bioinformatics. 2021 Oct 12;22(Suppl 10):494. doi: 10.1186/s12859-021-04403-1 (PMC8513176; doi:10.1186/s12859-021-04403-1)
Supplement: Supplementary file 8 — Additional file 8: Fig. S3, Table S3. Performance of the linker design algorithm of CirPred for proteins with various termini distances. [file 12859_2021_4403_MOESM8_ESM.pdf]

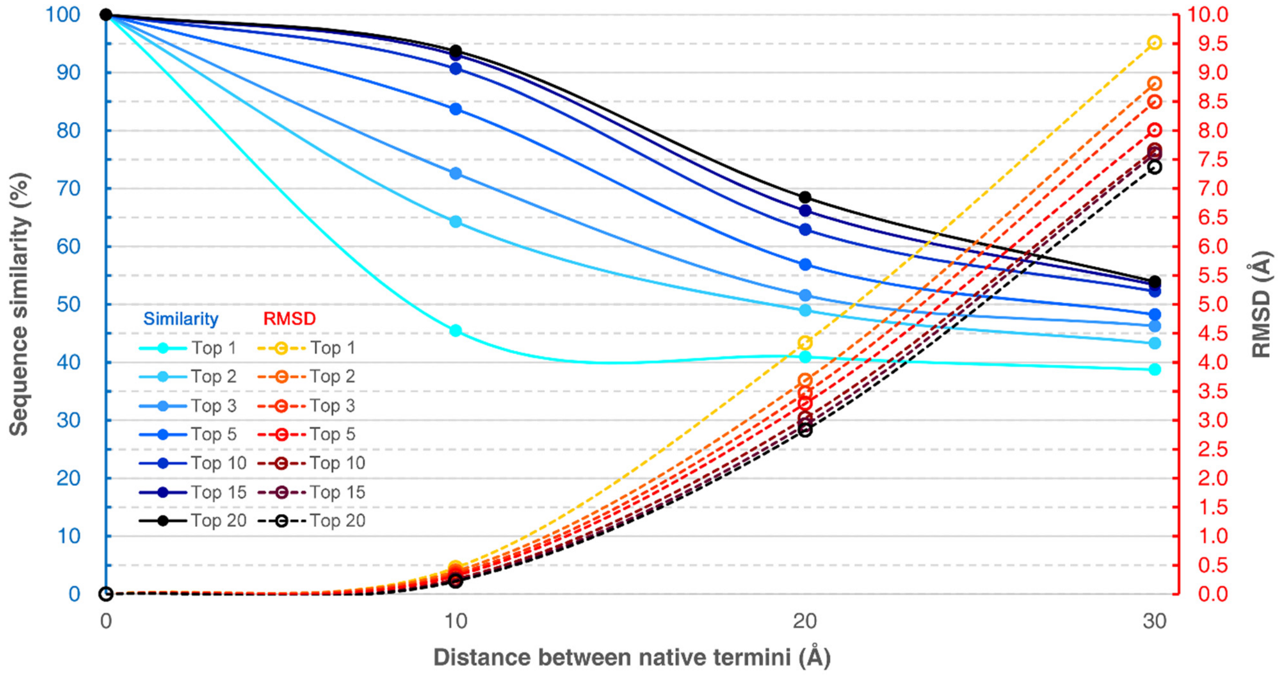

Fig. S3. Performance of the linker design algorithm of CirPred for proteins with various termini distances. This plot summarizes the average sequence similarity and RMSD values between the designed and actual linkers for proteins with different distances of native N- and C-termini in the CPDB linker dataset. For an input protein, the proposed linker design algorithm outputs several candidate designed linkers ordered by potential energy. The algorithm performed best for proteins with termini distances ≤10 Å. Although the performance seemed to decrease as the termini distance increased, by retrieving more designed candidate linkers for a given protein, the chance to find native-like linkers for that protein would significantly increase.

Table S3. Performance of the linker design algorithm of CirPred for proteins with various termini distances.

Distance of termini (Å)	Sequence similarity[a] (%)	RMSD[a] (Å)
≤10	90.7	0.258
10–20	62.9	3.038
20–30	52.3	7.663
Average	68.6	3.653

[a]The average sequence similarity and RMSD between the linkers designed by CirPred and the native linkers for proteins in the CPDB linker dataset. These measure values were obtained by retrieving the top 10 optimal candidate linkers designed by CirPred.
