# Supplementary material for: CirPred, the first structure modeling and linker design system for circularly permuted proteins
Source: BMC Bioinformatics. 2021 Oct 12;22(Suppl 10):494. doi: 10.1186/s12859-021-04403-1 (PMC8513176; doi:10.1186/s12859-021-04403-1)
Supplement: Supplementary file 10 — Additional file 10: Discussion. Advanced discussions on the utilization, limitation, and future developments of the CirPred method. [file 12859_2021_4403_MOESM10_ESM.pdf]

Advanced Discussions

On modeling 3D domain-swapped circular permutants

It was observed that, when comparing the structure of an actual CPM and the model generated by conventional comparative modeling methods, most differences occurred after the CP site, especially for proteins that seemed to comprise multiple domains (see **Fig. 2c–e** and **Data S1** for examples). This problem might be solved by MD simulations with multiple rounds of high-temperature annealing. However, there was no guarantee; even if there were, the time cost would be too high either for implementing a fast responding web server or for the experiments of this study, where over 10 thousand such simulations would be performed. Therefore, we developed the "CP-site-hinge" algorithm to refine the model after the comparative modeling step. In this algorithm, the optimal orientation between the two proportions of a protein delimited by the CP site was identified, according to simulated potential energy, by screening many rotated orientations with the CP site fixed like a hinge. In addition to reducing the computation time and generally improving the accuracy of the created model, this refinement step enabled CirPred to predict the 3D domain-swapped CPM structure of βB2-crystallin correctly. Although this case demonstrated that CirPred has the potential to detect 3D domain swapping induced by CP, there are two noteworthy things. First, the "hinge" between the swapping domains of this crystallin was coincidently the same as the CP site. It is not sure yet whether this refinement will generally apply to all DS plus CP cases. Second, this refinement strategy tended to report closed conformations since it identified optimal conformations according to simulated potential energy. If the hinge loop of a protein is long enough to allow the protein to adopt either an open or a closed conformation, the closed conformation will be favored in terms of potential energy without the presence of a binding partner in the modeling system. This problem may be solved by introducing the binding partner of the target protein into the modeling space or by predicting the quaternary structure of the target before modeling; the procedure of the protein complex version of $(PS)^2$ [21] may be applicable.

Improving viable CP site prediction

CPred is a viable CP cutting site predicting method we developed for proteins with known structures [16, 17]. The machine learning model of its current version was trained in 2011 when known inviable CP sites were scarce (only ~200 cases). Although the CPred provides a score to help estimate the viability of a CP site, its accuracy is far from being perfect. The number of known inviable CP sites (unfoldable, insoluble, or unstable) has remarkably increased during the last decade. For improving CP viability prediction, many new data can be used to re-train the machine learning model of CPred now. Additionally, the **CirPred** algorithm may be integrated into the pipeline of CPred to enhance its accuracy. Recently, Chuang *et al.* used CPred to predict viable CP sites as they untied a knotted SPOUT RNA methyltransferase (PDB entry 1ns5) by CP [42]. They chose eight viable and one inviable CP sites predicted by CPred and conducted molecular cloning, protein purification, and structural analyses. As shown in the following table, among the predicted viable CP sites, Gly82 turned out to be soluble and stable (shown in blue), and four of them were soluble after refolding treatments (light blue rows). Arg121, the predicted inviable CP site, was indeed insoluble (the red one).

CP site	CPred score	CirPred DOPE score	DOPE rank	Experimental results [42]
Pro72	0.947	-18686	3	insoluble
Gly82	0.856	-18623	4	soluble, stable
Lys92	0.871	-18618	5	insoluble
Arg104	0.982	-18596	6	insoluble
Gln118	0.678	-18587	7	soluble after refolding
Arg121	0.078	-18534	9	insoluble
Met124	0.867	-18774	1	soluble after refolding
Ser128	0.951	-18706	2	soluble after refolding
Asn132	0.629	-18553	8	soluble after refolding

The experimentally-verified inviable CP site Arg121 received an extremely low CPred score, representing a true negative prediction. It was suggested in the paper of CPred that users should select viable CP sites with scores > 0.85 for reliability [16]. Agreed with this suggestion, Gln118 and Asn132 received < 0.7 CPred scores and were soluble only after refolding. Regarding those CP sites with scores > 0.85, there was no automatic way to determine which ones could lead to viable CPMs.

To preliminarily test the potential of integrating CPred and CirPred, we used CirPred to generate CPM models and calculate energy scores for the nine CP sites. The above table shows that the inviable CP site Arg121 registered the worst (highest) DOPE energy score. Meanwhile, the DOPE scores of Met124, Ser128, Pro72, and Gly82 were better (lower) than those of the others, implying they might be viable CP sites. Among these four sites with high CPred scores and low CirPred DOPE scores, three were experimentally-verified viable. The CPM of Gly82 was soluble, and its crystal structure had been solved by Chuang *et al.* (PDB 6ahw) [42]. The structural alignment rate and RMSD between the CirPred model of Gly82 CPM and its crystal structure were 86.1% and 0.872 Å, respectively. This high structural similarity demonstrated the quality of CirPred modeling. Moreover, the CPMs of Met124 and Ser128 were soluble after refolding, meeting the definition of viable CP sites by the CPred [16, 17]. To sum up, from the nine selected CP sites, combining the information produced by CPred and CirPred reduced the number of candidate viable CP sites to four, among which three were viable, indicating CirPred could help cut down the cost of CP-based protein engineering.

Limitation of the implemented web server

Currently, the implemented web server requires the user to provide the structure of the native protein. For situations where the native protein structure is not determined yet, the server will not be directly applicable; however, the user can alternatively input a model of the native protein prepared by conventional structure modeling systems. Since the current CirPred server can deal with just one polypeptide, it may not be suitable for modeling protein complexes or proteins with a ligand, especially when the binding partner or ligand contributes much to the conformational stability of the target structure.

Future work

For enabling the CirPred server to find templates for target proteins without known native structures, the CP-homolog search algorithm CPSARST [25] will be re-implemented in combination with the $(PS)^2$ template search algorithm. Moreover, a variable gap penalty scheme will be implemented to improve the sequence alignment between target and template proteins that share low sequence homology.

Although CirPred was developed for circularly-permuted proteins, it is actually suitable for regular co-linear protein structure modeling (**Additional file 2**). To make CirPred more compatible with co-linear modeling, we will re-implement an efficient co-linear protein structural similarity search algorithm (*e.g.*, *i*SARST [29]) along with the $(PS)^2$ search algorithm to identify suitable co-linear templates from protein structure databases. Besides, multiple templates for one target will be supported to increase the accuracy of the constructed model.

For improving the linker design procedure, more training data of CPMs will be retrieved from literature or by surveying PDB using CPSARST [25]. New features will also be designed to enhance the machine-learning-based linker residue predictor.

Modeling complex structures will be implemented using the $(PS)^2$ strategy and recent protein-protein docking algorithms. In addition to completing the function of CirPred as a comparative modeling system, it will enhance the ability of CirPred in detecting the DS phenomenon caused by CP. This ability will be utilized to identify naturally occurring "CP + DS" cases. Studying those cases not only helps scientists reveal the mechanisms of protein molecular evolution but will also provide valuable information for designing novel engineered proteins and biomaterials.
