# Supplementary material for: CirPred, the first structure modeling and linker design system for circularly permuted proteins
Source: BMC Bioinformatics. 2021 Oct 12;22(Suppl 10):494. doi: 10.1186/s12859-021-04403-1 (PMC8513176; doi:10.1186/s12859-021-04403-1)
Supplement: Supplementary file 11 — Additional file 11: Table S4. Amino acid propensities of the CPDB linker dataset. [file 12859_2021_4403_MOESM11_ESM.pdf]

Table S4. Amino acid propensities of the CPDB linker dataset.

Rank	Amino acid	Propensity (%)
1	Ser	8.42
2	Glu	8.04
3	Gly	7.82
4	Lys	7.72
5	Leu	7.20
6	Ala	7.12
7	Thr	6.81
8	Asp	6.67
9	Asn	6.08
10	Gln	4.56
11	Arg	4.51
12	Phe	4.41
13	Val	4.07
14	Pro	3.83
15	His	3.10
16	Ile	3.09
17	Tyr	2.56
18	Met	1.87
19	Trp	1.49
20	Cys	0.66

These propensity data obtained from the CPDB linker dataset (**Additional file 6**) were used to assign temporary linkers for the proposed linker design protocol to redesign linkers for proteins in Dataset S (**Additional file 9**); in that experiment, the CPDB linker dataset was the training set, and Dataset S was the independent test set. The same data were also utilized in the implemented web server of CirPred (Mode 3: Linker Design). However, in the 500-round independent test performed by random divisions of the CPDB linker dataset, these propensities were not applied; instead, in each round of the independent test, propensity data were obtained from the training set of that round. The training and independent test sets of the 500 rounds are available in **Additional file 7**.
