# Supplementary material for: CirPred, the first structure modeling and linker design system for circularly permuted proteins
Source: BMC Bioinformatics. 2021 Oct 12;22(Suppl 10):494. doi: 10.1186/s12859-021-04403-1 (PMC8513176; doi:10.1186/s12859-021-04403-1)
Supplement: Supplementary file 12 — Additional file 12: Fig. S4. Relation between the length of linkers and the distance of the termini they bridged. [file 12859_2021_4403_MOESM12_ESM.pdf]

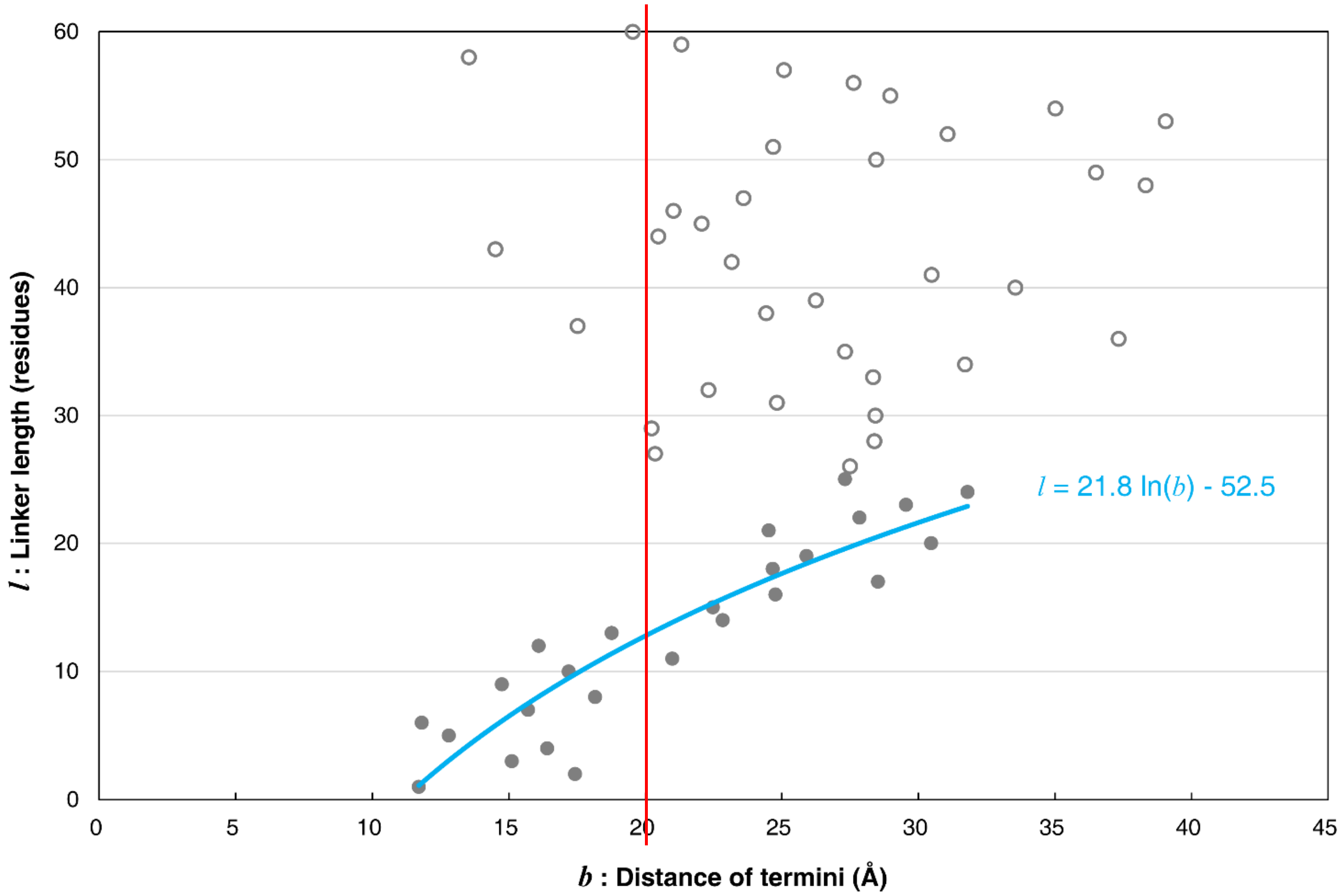

Fig. S4. Relation between the length of linkers and the distance of the termini they bridged. This plot illustrates, for proteins with different distances of N- and C-termini (*b*; the horizontal axis), how long a linker may be required to connect the termini (*l*; the vertical axis). Proteins examined here were from the CP pair dataset (**Additional file 3**). Each dot was made by proteins possessing linkers of the same length and termini of varying distances. For example, a dot (l_t, b_t) on this plot represents that several proteins possessed a linker of length l_t, and the average distance of these proteins' termini was b_t. Proteins with a linker length ≤26 residues are drawn as solid dots (●) or otherwise drawn as hollow dots (○). The equation shown in the plot belongs to the trend curve of the solid dots. The red line indicates that it should be careful to use this equation to estimate the length of a linker when the termini distance of the protein is >20 Å. For proteins with such large termini distances, an alternative algorithm for estimating the linker length can be applied (see the "**Length estimate of the linker**" subsection of **Methods**).
